# Transcription factor PagLBD21 functions as a repressor of secondary xylem development in *Populus*

**DOI:** 10.48130/FR-2022-0019

**Published:** 2022-12-21

**Authors:** Hao Li, Shiguang Yin, Linjing Wang, Na Xu, Lijun Liu

**Affiliations:** State Forestry and Grassland Administration Key Laboratory of Silviculture in downstream areas of the Yellow River, College of Forestry, Shandong Agricultural University, Taian 271018, Shandong, China

**Keywords:** *Populus*, Secondary growth, LBD domain transcription factor, RNA-seq, DAP-seq

## Abstract

During secondary growth in trees, xylem cells differentiated from cambium cells begin to synthesize secondary cell walls that are primarily composed of cellulose, hemicellulose and lignin, and are deposited between primary cell walls and plasma membranes, leading to wood formation. Identification of regulatory genes functioning during this developmental process is valuable for increasing wood production. In this study, we functionally characterized an LBD (LATERAL ORGAN BOUNDARIES DOMAIN) transcription factor PagLBD21 as a repressor of secondary xylem development in *Populus*. Compared to wild type plants, transgenic plants overexpressing *PagLBD21* (*PagLBD21OE*) exhibited decreased xylem widths in cross-sections. Consistent with the functional analysis, RNA sequencing (RNA-seq) analysis revealed that genes functioning in xylem development and secondary cell wall biosynthesis pathways were significantly down-regulated in *PagLBD21OE* plants. We also performed DNA affinity purification followed by sequencing (DAP-seq) to identify genome-wide target genes of PagLBD21. Furthermore, we compared the RNA-seq and DAP-seq datasets of PagLBD21 and PagLBD3, and the results showed that there was a significant overlap between their target genes, suggesting these two LBD transcription factors are functionally redundant during secondary growth.

## INTRODUCTION

Wood is the world's most abundant renewable resource used for timber, pulp, and energy. The process of wood formation is known as secondary growth, includes cell differentiation, cell expansion, secondary cell wall (SCW) biosynthesis, and programmed cell death. SCW is a specialized cell wall, consisting of three major components, cellulose, lignin, and hemicellulose, deposited inside of primary cell wall, The differentiation of secondary xylem from cambium cells and SCW deposition are key steps in determining wood yield and quality.

Transcriptional regulation is critical for secondary growth, and many transcription factors have been demonstrated as key regulators of different stages of secondary growth. For instance, several class I KNOX transcription factors in *Populus* are key regulators of vascular cell maintenance and differentiation during secondary growth^[1−4]^. Several class III HD-Zip transcription factors in *Populus* are important for vascular cambium initiation and xylem differentiation^[5−8]^. As shown in *Arabidopsis*, many NAC and MYB transcription factors have been demonstrated as master regulators of SCW biosynthesis in *Populus*^[9−16]^. Modulating the expression of these regulatory genes could dramatically change the wood property and yield.

LBD (LATERAL ORGAN BOUNDARIES DOMAIN) proteins belong to a plant-specific transcription factor family that participate in various plant developmental processes^[17,18]^. The LBD transcription factors contain a conserved LOB (Lateral Organ Boundaries) domain at the N-terminal responsible for DNA binding activity and a variable C-terminal responsible for activation/repression of target gene transcription. The conserved LBD domain can be further divided into three subdomains, including a C block (CX2CX6CX3C), a GAS block (Gly-Ala-Ser), and a leucine-zipper-like coiled-coil motif (LX6LX3LX6L). A conserved proline amino acid in the GAS block is critical for the DNA binding activity and biological function of LBD18 in *Arabidopsis*^[19]^. Several studies have demonstrated that LBD transcription factors play important roles in secondary growth. For example, in *Arabidopsis* root, two pairs of LBD homologous genes (*LBD3* and* LBD4*, *LBD1* and* LBD11*) act redundantly downstream of cytokinin to promote radial growth and function in maintenance of cambial stem cells, overexpression of these LBD genes leads to rapid secondary growth in root^[20]^. Another two LBD genes, *LBD18* and *LBD30*, positively regulate tracheary element (TE) differentiation in *Arabidopsis*, overexpression of either *LBD18* or *LBD30* induce transdifferentiation of nonvascular cells into TE-like cells^[21]^. CcLBD25 functions as a key regulator of haustorium development in the parasitic plant dodder, and down-regulation of CcLBD25 interferes with the haustorium penetration and formation of vascular connections to host plants^[22]^. In *Populus*, PtaLBD1 is a positive regulator of phloem development and overexpression of PtaLBD1 significantly enhances wood growth^[23]^. Similar to PtaLBD1, another LBD transcription factor PagLBD3 also plays important roles in regulating cambial cell differentiation into secondary phloem and xylem in *Populus*^[24]^. There are 58 LBD transcription factors in *Populus*^[24,25]^, but the function of most is unknown.

In this study, we functionally characterized an LBD transcription factor, PagLBD21, which acted as a repressor of xylem development in hybrid poplar (*Populus alba* X* P. glandulosa*) clone 84K. We also identified differentially expressed genes (DEGs) in *PagLBD21OE* plants using RNA-seq and PagLBD21 target genes using DAP-seq. Comparative study found that genes regulated by PagLBD21 and PagLBD3 were significantly overlapped during secondary growth. Our results provided valuable information for further dissecting the regulatory network of wood formation.

## MATERIALS AND METHODS

### Plant materials and growth conditions

*Populus alba* x* Populus tremula* var. *glandulosa* clone 84K was used in this study. All plants were propagated *via* tissue culture and transferred to soil for phenotype analysis and sequencing. Plants were grown in a phytotron at 26 °C under a 16 h light/8 h dark photoperiod.

### Plasmid construction and transformation

The *LBD21* cDNA in poplar^[26,27]^ was amplified with primers *LBD21-31-Kpn*I*-5'* and *LBD21-31-Xba*I*-3'* (Supplemental Table S1). The *pEASY-BLUNT* Simple Cloning Kit (TransGene Biotech) was used to recombine the PCR products. *Kpn*I and *Xba*I, the restriction endonuclease, were used to digest the recombinant *pEASY-BLUNT* and *PzP211-35S-PolyA* vectors. After running agarose gel electrophoresis and collecting two aim sequences, they were connected by T4 ligase (Takara). This construct was introduced in *Agrobacterium Tumefaciens* (GV3101) and used for transformation by leaves and stem dipping. The OD_600_ value of the bacterial liquid is 0.3 to 0.45, and the immersion time (15 to 20 min) was the more ideal infection condition. After that, the leaves and stem segments were placed in differentiation medium containing 50 mg/L kanamycin, and 50 mg/L cefotaminate for about one month, with succession transfers every ten days. Until the new shoots grew, the differentiation solid medium containing 0.5 mg/L 6-BA, 0.002 mg/L TDZ, and 0.1 mg/L NAA was replaced with rooting solid medium (0.01 mg/L NAA, 0.1 mg/L IBA). All the plant tissue culture was performed on the half of the Murashige and Skoog medium.

### Protein alignment

All the data was retrieved from Phytozome and DNA sequencing results. Protein alignment was performed using DNAMAN software. Sequences were aligned with Multiple Sequence Aligment.

### Hand sectioning and staining

Plants planted in the soil for a month were used for slice observation. The number of internodes was counted starting from the first visible internode and counting down to the surface of the soil. The target internode was cut into thin slices by Gillette blades, placed in 0.1% phloroglucinol solution or 0.05% toluidine blue O (Sangon Biotech) for about 5−10 min, and temporary tablets would be made for microscopic observation (OLYMPUS BX53). 0.1% phloroglucinol solution was configured using anhydrous ethanol 10 mL, concentrated hydrochloric acid 1.6 mL and phloroglucinol 0.01 g.

### RNA isolation, cDNA preparation and quantitative real-time PCR

From 2-month-old wild-type (WT) plants, samples of the following tissues were taken in order to analyze the tissue expression pattern of PagLBD3: leaf, leaf petiole, root, whole stem 1^st^ to 4^th ^internodes, whole stem 9^th^ to 12^th^ internodes, bark, secondary phloem, and secondary xylem. To obtain secondary phloem and secondary xylem samples, we peeled the bark and scraped the corresponding side of the phloem or xylem. All materials were ground into a fine powder using liquid nitrogen, and total RNA was extracted using the cetyltrimethylammonium bromide (CTAB) method. Then DNase I (Takara, 2270) and purified columns (Takara MiniBEST Plant RNA Extraction Kit, 9769) were used to get high quality RNA. The RNA purity and concentration were measured by Nanodrop 2000.

The same amount of RNA was used for cDNA reverse transcription. HiScript II Q Select RT SuperMix (+gDNA wiper) (Vazyme, R223–01) was used to synthesize cDNA, while 2×ChamQ SYBR Color qPCR Master Mix (Vazyme, Q411–02) was used for qPCR. *Actin* was used as the internal control to normalize gene expression level with the 2^−ΔΔCq^ method. Supplemental Table S1 contains the list of the primers used for qPCR. The qPCR was run with a minimum of three duplicates.

### RNA-seq analysis

The 7^th^−12^th^ internodes of two-month-old WT and *PagLBD21-OE* transgenic plants were harvested at 10 am for RNA extraction. Illumina Hiseq X 10 platform was used with paired-end 150 bp mode. Cleaned sequencing reads were mapped to *P. trichocarpa* v3.0 genome assembly with HISAT2 using default parameters, and normalized to the fragments per kilobase of exon per million mapped fragment (FPKM) by packages edgeR^[28]^. Differentially expressed genes (DEGs) were identified as previously described^[4]^

*P. trichocarpa* v3.0 Gene Ontology (GO) annotation and the BIOCONDUCTOR package GOstats^[29]^ were used in the analysis, and p-value ≤ 0.01 was seen as significantly enriched.

### DAP-seq and data analysis

To prepare the genomic DNA library, the genomic DNA of 7^th^−12^th^ internodes of 2-month-old WT plants were extracted by CTAB methods, sonicated to 200−500 bp, and purified with 3 M sodium acetate. At the same time, glutathione *S*-transferase (GST) and GST-PagLBD21 proteins were prepared. The *pColdIII* vector (Takara) ligated with the GST and GST-PagLBD21 amplification products respectively. The correct vectors were transformed into the *E.coli* BL21 (DE3) cell line to induce protein at 15 °C. Then the proteins were purified and then DNA affinity purification (DAP) was performed with the genomic DNA library. The GST and GST-PagLBD21 protein-bound DNA fragments were eluted and amplified for sequencing. Three biological replicates were prepared. Data analysis was performed with IDR pipeline as previously described^[4]^. Peak annotation was performed with CHIPPEAKANNO^[30]^.

### Data availability

The RNA-seq and DAP-seq assembly of *PagLBD21* is available in the CNCB database under accession number CRA007846.

## RESULTS

### Identification of *PagLBD21* gene

**Figure 1:**
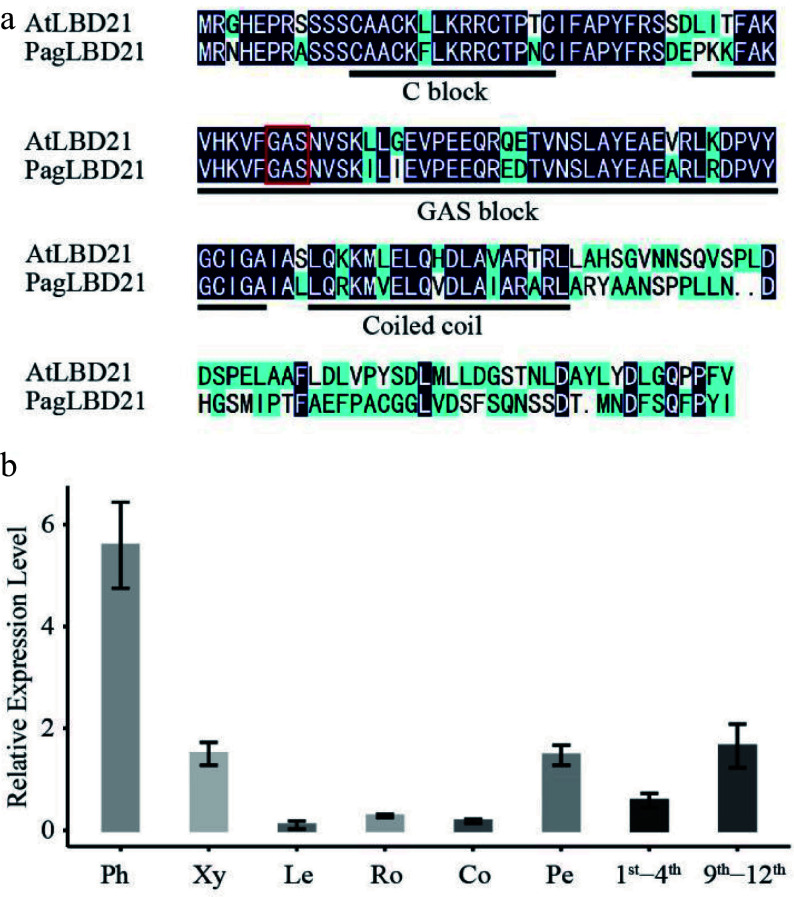
Characterization of *PagLBD21* in *Populus*. (a) Amino acid sequence alignment of *Populus* PagLBD21 and *Arabidopsis* AtLBD21. Black and blue colors indicate identical and similar amino acids, respectively. The red box represents the core amino acid of GAS block. (b) RT-qPCR of *PagLBD21* in different tissues using wild type. Each value is the mean ± standard error (SEM) of three replicates (n = 3 technical repetitions). Ph, phloem; Xy, xylem; Le, leaves; Ro, root; Co, cortex; Pe, petiole; 1^st^−4^th^, the stem of 1^st^ to 4^th^ internodes; 9^th^−12^th^, the stem of 9^th^ to 12^th^ internodes.

We identified a *PagLBD21* (Potri.010G186000) gene displaying significantly higher expression in secondary phloem than in secondary xylem from transcriptome analysis^[31]^ (Supplemental Fig. S1). The LBD family was classified into two classes based on sequence analysis and N-terminal domain structure^[17]^. PagLBD21 belonged to class I, which contains a conserved LOB (LATERAL ORGAN BOUNDARIES) domain in the N-terminal (Fig. 1a). Phylogenetic analysis showed that PagLBD21 had a close distance to the previously reported PagLBD3^[24]^. To further analyze the expression pattern of *PagLBD21* in the wood forming zone, we searched the AspWood database and found it belongs to e1 cluster^[32]^, which displayed high expression from secondary phloem to expanding xylem and declined significantly in matured xylem (Supplemental Fig. S1b). We also performed RT-qPCR to examine the expression of *PagLBD21* in different tissues of poplar (Fig. 1b). Consistently, *PagLBD21* was expressed highest in the phloem, followed by xylem. Notably, *PagLBD21* expression level in the 9^th^−12^th^ internodes was higher than in the 1^st^−4^th^. Together, these results suggested *PagLBD21* participating in regulating secondary growth.

### Phenotypes of *PagLBD21* overexpression plants

**Figure 2:**
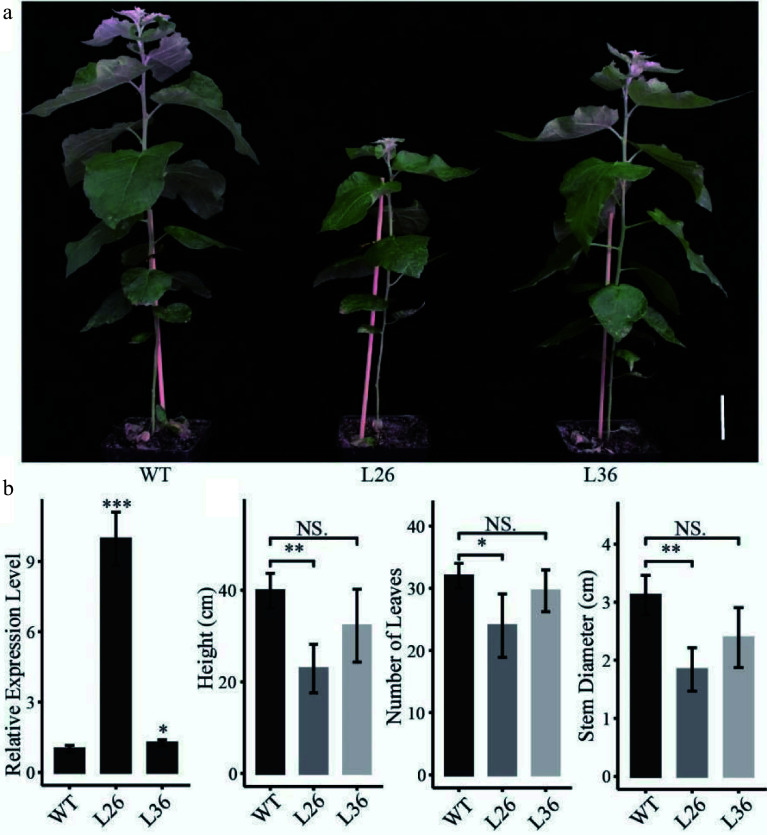
Effects of *PagLBD21* overexpression (*PagLBD21OE*) in *Populus*. (a) Morphological phenotypes of 2-month-old *PagLBD21OE* transgenic lines compared to wild type (WT). Scale bar = 5 cm. The WT, L26 and L36 represent wild-type, the overexpression line of number 26, the overexpression line of number 36, respectively. (b) RT-qPCR of *PagLBD21* in *PagLBD21OE* transgenic lines and WT. (c) The comparison of WT and *PagLBD21OE* transgenic lines in plants' height, stem diameter, and number of leaves. Each value is the mean ± SD of three replicates (n = 3 biological replicates). Student's t-test was used: *, P < 0.05; **, P < 0.01; NS, nonsignificant.

To determine the function of *PagLBD21* in *Populus*, we generated transgenic plants overexpressing* PagLBD21* (*PagLBD21OE*) in *Populus*. Eleven independent *PagLBD21OE* transgenic lines were obtained. We selected two transgenic lines L26 and L36, which displayed mild growth changes, for further analysis. RT-qPCR showed that *PagLBD21* expressed significantly higher in both L26 and L36 lines than in non-transgenic plants (WT) (Fig. 2b). Compared to WT, *PagLBD21OE* plants had shorter stems and smaller stem diameter, and fewer leaves (Fig. 2a & b).

### *PagLBD21* represses xylem development

**Figure 3:**
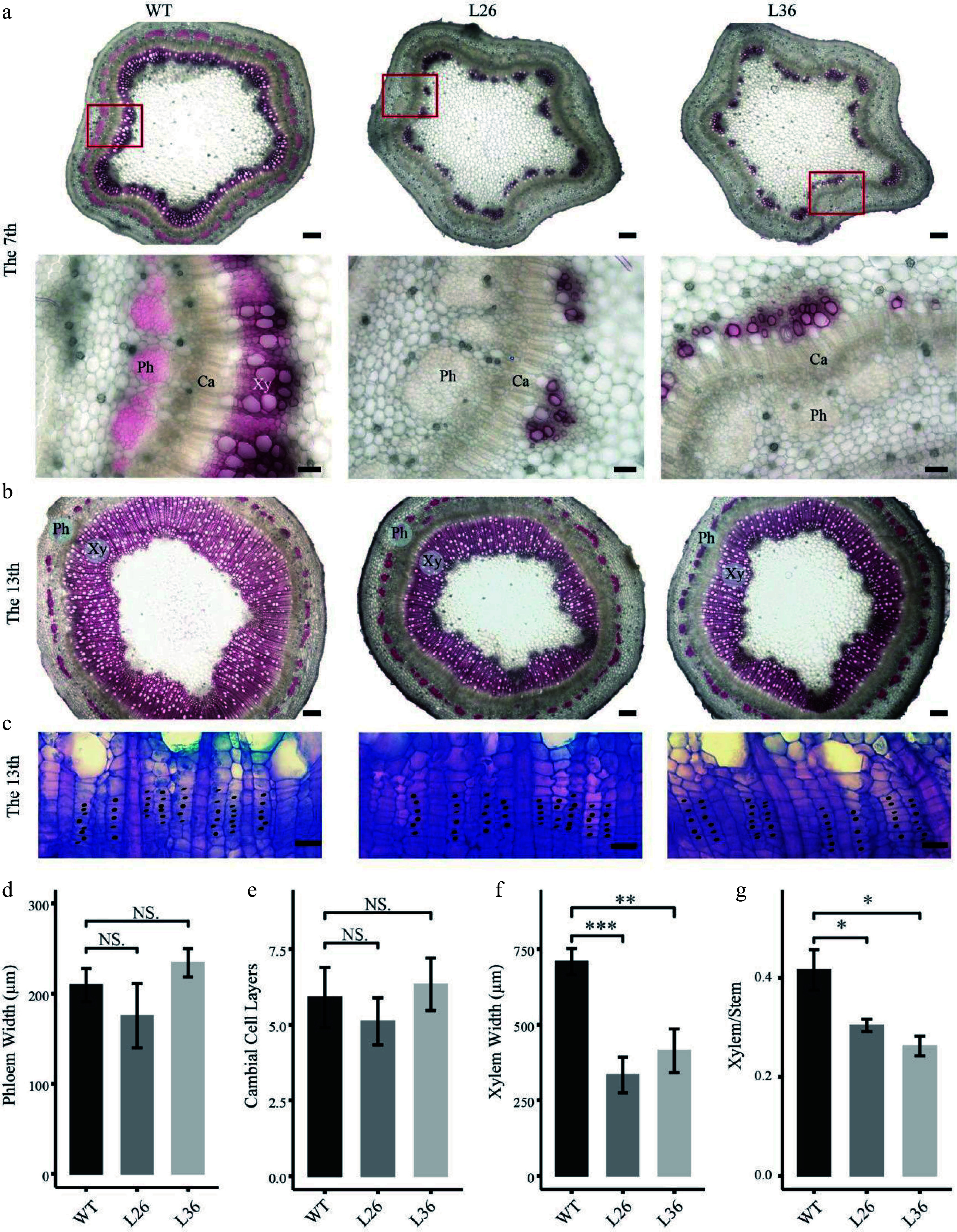
Effects of *PagLBD21* overexpression (*PagLBD21OE*) on secondary growth in *Populus*. Stem cross sections of (a) 7^th^ and (b), (c) 13^th^ internodes of WT and *PagLBD21OE* plants. Scale bar: 200 μm for (b) and the upper panel of (a), 50 μm for the lower panel of (a), 20 μm for (c). The red-boxed area in the upper panels is depicted in detail in the lower panels. Xy, xylem. Ph, phloem. Ca, cambium. Detailed observation of the cambial zone (c) was stained by toluidine blue O, cambial cells were marked with black dots. The cambial cell layers (e) were analyzed according to (c). The phloem widths (d), xylem widths (f), and xylem widths divided by the radius (Xylem/Stem) (g) in 13^th^ internodes were measured by ImageJ. Each value is the mean ± SD of three replicates (n = 3). Student’s t-test was used: *, P < 0.05; **, P < 0.01; ***, P < 0.001; NS, nonsignificant.

As the *PagLBD21OE* plants displayed smaller stem diameters, we speculated that *PagLBD21* negatively regulated stem secondary growth in plants. To test this hypothesis, we performed freehand sectioning of plant stems and stained with the lignin-specific dye phloroglucinol. We found that xylem development was clearly repressed in *PagLBD21OE* plants (Fig. 3). In the 7^th^ internode, the WT plants formed a completed xylem ring while the *PagLBD21OE* plants still displayed discontinuous xylem (Fig. 3a); in the 13^th ^internode, the width of secondary xylem region in *PagLBD21OE* plants was significantly narrower than in WT (Fig. 3b); the cambium zone did not exhibit clear changes (Fig. 3c). Further quantification analysis showed that the width of the phloem region and number of cambium cells were similar to WT while the xylem region was significantly reduced (Fig. 3d−f). Correspondingly, the ratio of xylem region in the whole stem (xylem/stem) was significantly reduced (Fig. 3g). Collectively, our results suggested that PagLBD21 is a repressor of xylem development.

### Transcriptome analysis of *PagLBD21OE* plants

To profile genes regulated by overexpressing *PagLBD21* during secondary growth, we performed RNA-seq with stem internodes 7^th^ to 12^th^ collected from 2-month-old *PagLBD21OE* (L36) and WT plants (MATERIALS AND METHODS). In total, 1421 significantly differentially expressed genes (DEGs) were identified between *PagLBD21OE* and WT plants (P-value < 0.05), with 722 and 699 up- and down-regulated DEGs in* PagLBD21OE* plants, respectively (Supplemental Table S2).

**Figure 4:**
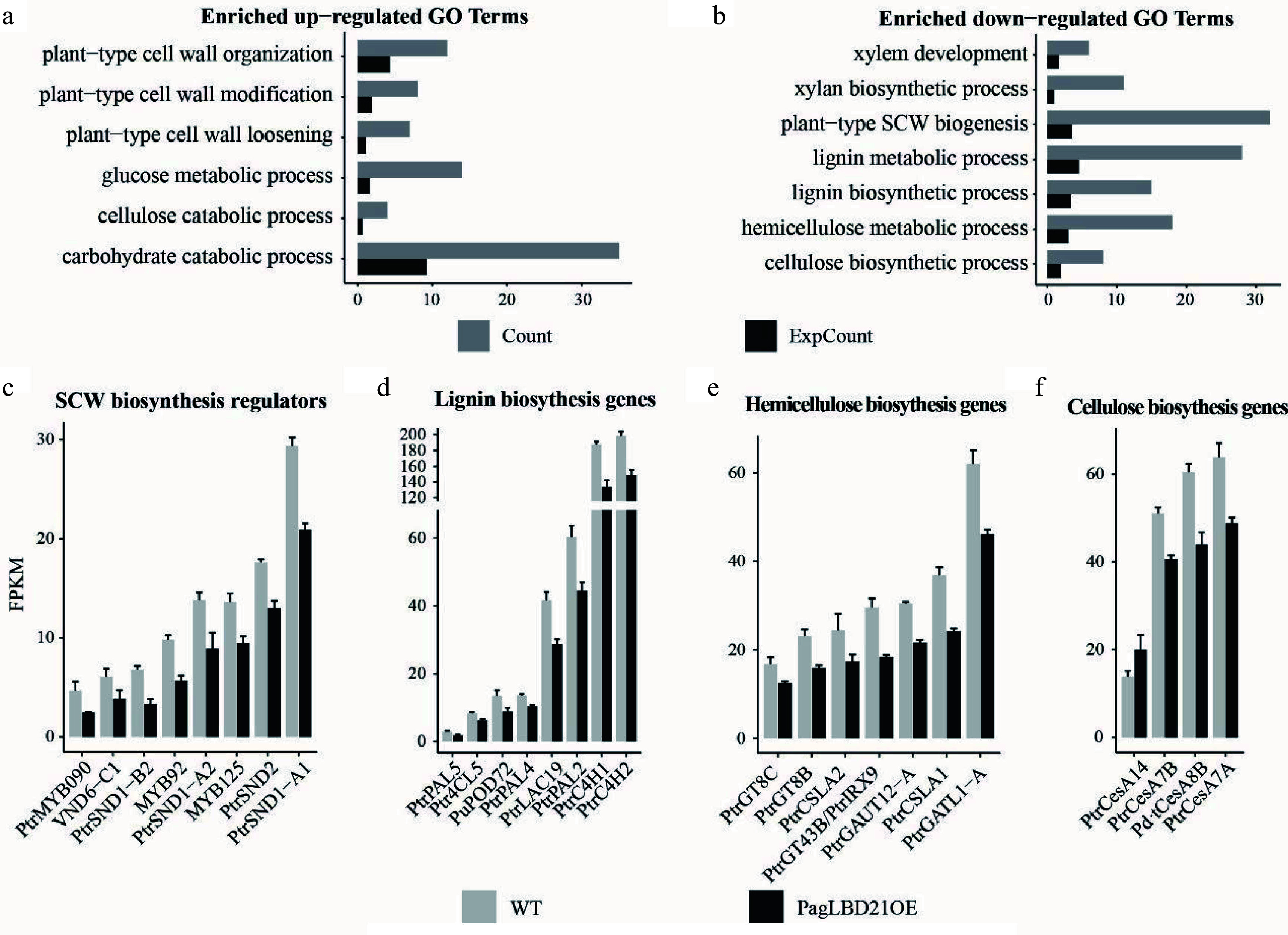
Transcriptome analysis of *PagLBD21OE* plant. (a), (b) Enriched Gene Ontology (GO) categories of (a) up-regulated and (b) down-regulated differentially expressed genes (DEGs) in *PagLBD21OE* line 36. Count, the number of genes in a GO term, ExpCount, the expected gene number. Expression of representative genes that function in (c) SCW biosynthesis regulatory pathway, (d) lignin biosynthesis, (e) hemicellulose biosynthesis, and (f) cellulose biosynthesis. The fragments per kilobase of exon per million mapped fragments (FPKM) for each gene from RNA-seq experiments were shown. Each value is the mean + standard error (SD) of three replicates (n = 3 technical repetitions). (n = 3 biological replicates).

Gene Ontology (GO) analysis was performed to characterize the function of DEGs. We found that the up-regulated DEGs were significantly enriched in GO categories including plant-type primary cell wall biogenesis, plant-type cell wall loosening, and plant-type cell wall modification (Fig. 4a, Supplemental Table S3); meanwhile, the down-regulated DEGs were significantly enriched in GO categories including cell differentiation, xylem development, and plant-type secondary cell wall biogenesis (Fig. 4b, Supplemental Table S3). These results were consistent with the observation that differentiation of the cambium cells to xylem was inhibited in *PagLBD21OE* plants.

Consistent with the functional characterization, many genes encoding key regulators and enzymes responsible for secondary cell wall biosynthesis were among the down-regulated DEGs in *PagLBD21OE* plants. For instance, positive regulators of secondary cell wall biosynthesis including *SND1-A1/A2/B2*, *VND6-C1*, *SND2*, *MYB92*, *MYB090*, *MYB125* were significantly down-regulated in *PagLBD21OE* plants (Fig. 4c), and most genes encoding key enzymes responsible for secondary cell wall cellulose, lignin, and hemicellulose biosynthesis were also significantly down-regulated in *PagLBD21OE* (Fig. 4d−f). Together, the RNA-seq data suggested that PagLBD21 inhibited xylem development partially through repressing secondary cell wall biosynthesis.

### Identification of genome-wide binding targets of PagLBD21 through DAP-seq

To further dissect the molecular function of PagLBD21, we performed DAP-seq to identify its genome-wide binding targets as previously reported^[24]^ (MATERIALS AND METHODS). The genomic 'input' DNA libraries were sequenced in parallel as a control for data analysis. Three biological replicates were prepared for both PagLBD21 DAP-seq and 'input' control.

**Figure 5:**
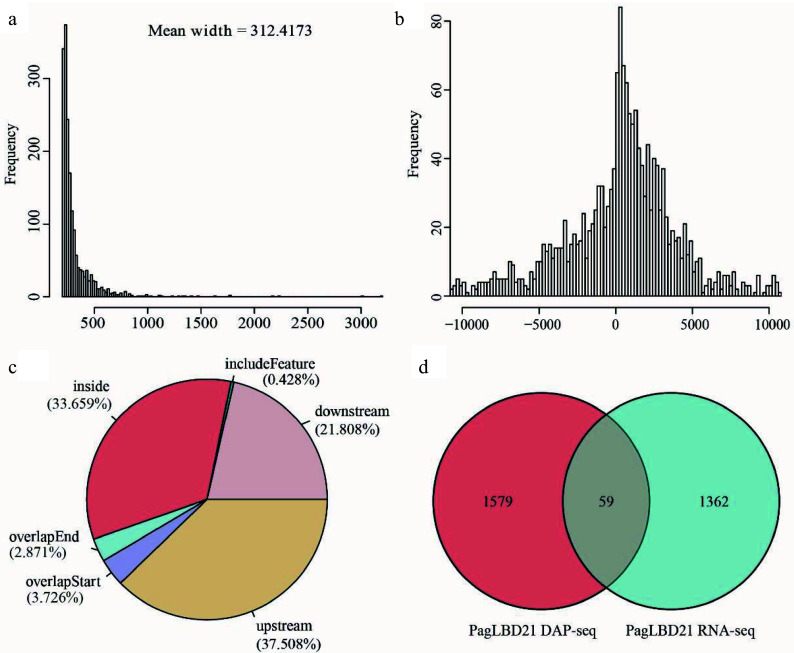
Analysis of genome-wide targets of PagLBD21 through DAP-seq. (a) The mean width of PagLBD21 binding sites identified in DAP-seq. (b) Genome-wide distribution of PagLBD21 binding sites is centered on gene transcriptional start sites (TSS). (c) The distribution of PagLBD21 binding sites relative to gene features. (d) Venn diagrams of target genes of DAP-seq data (left) and DEGs of RNA-seq data (right) of PagLBD21.

We analyzed the DAP-seq data with IDR pipeline and used MACS2 as the peak caller^[4,33]^. In total, we obtained 1790 peaks as PagLBD21 binding sites which associated with 1639 unique target genes (Supplemental Table S4). The average width of PagLBD21 binding site was 312 bp (Fig. 5a). The genome-wide distribution of PagLBD21 binding sites was enriched around the transcription start site (TSS) of the target gene (Fig. 5b). Further inspection of the location of the binding sites relative to target genes showed that 37.508% was upstream of gene TSS and 3.726% was overlapped with gene TSS (Fig. 5c).

To identify genes that are possibly regulated by PagLBD21 directly, we compared the DEGs of RNA-seq data and target genes of DAP-seq data. Results showed there were 59 overlapped genes between these two datasets (Fig. 5d), including 25 up-regulated and 34 down-regulated genes (Supplemental Table S5), respectively.

### Comparison study of target genes of PagLBD3 and PagLBD21

We previously reported another LBD gene, *PagLBD3*, which participated in regulating secondary growth in *Populus*^[24]^. Characterization of *PagLBD21OE* and *PagLBD3OE* plants showed partially similar phenotype, such as decreased xylem width. We hypothesize that these two LBD genes may target a group of common genes during secondary growth. Therefore, we performed pair-wise comparisons of the RNA-seq and DAP-seq data of PagLBD21 and PagLBD3. As shown in Table 1, there were 793, 260, 373, and 655 overlapped genes between PagLBD21 DAP-seq & PagLBD3 DAP-seq, PagLBD21 DAP-seq & PagLBD3 RNA-seq, PagLBD21 RNA-seq & PagLBD3 DAP-seq, and PagLBD21 RNA-seq & PagLBD3 RNA-seq, respectively. Notably, all these overlaps were significantly higher than random distribution in HYPGEOMDIST test.

**Table 1:** Overlapping study of DAP-seq and RNA-seq related genes between PagLBD21 and PagLBD3.

	LBD21 DAP-seq	LBD21 RNA-seq	LBD3 DAP-seq	LBD3 RNA-seq
LBD21 DAP-seq	1,638	−	−	−
LBD21 RNA-seq	59	1,421	−	−
LBD3 DAP-seq	739	373	7,955	−
LBD3 RNA-seq	260	655	1,782	6,468

As the number of both PagLBD3 related DEGs and target genes were about five times of that related to PagLBD21, the common genes in PagLBD21 DAP-seq & PagLBD3 DAP-seq and PagLBD21 RNA-seq & PagLBD3 RNA-seq only accounts for 9% (739 out of 7,955) and 10% (655 out of 6,468) of PagLBD3 DAP-seq derived target genes and RNA-seq derived DEGs, respectively. However, the common genes in PagLBD21 DAP-seq & PagLBD3 DAP-seq and PagLBD21 RNA-seq & PagLBD3 RNA-seq accounts for 45% (739 out of 1,638) and 46% (655 out of 1,421) of PagLBD21 DAP-seq derived target genes and RNA-seq derived DEGs, respectively. Detailed inspection found that many key regulators of secondary growth were among the common targets of PagLBD21 DAP-seq and PagLBD3 DAP-seq, such as auxin transporter genes (*PtPIN6b*, *PtAUX8LAX4*, and *PtPIN3a*)^[34,35]^, *PtrMYB152* which encodes a positive regulator of lignin biosynthesis^[36]^, and *PtLOG7b* which encodes a key enzyme during cytokinin biosynthesis^[34,37]^ (Supplemental Table S6). Functional analysis showed that in the common DEGs in PagLBD21 RNA-seq & PagLBD3 RNA-seq, plant-type cell wall loosening, and plant-type cell wall modification categories were enriched in up-regulated genes, while cell differentiation, xylem development, and plant-type secondary cell wall biogenesis categories were enriched in down-regulated genes (Supplemental Table S7). Collectively, these results suggested that PagLBD21 and PagLBD3 targeted a common group of genes during secondary growth.

## DISCUSSION

Extensive secondary growth is a prominent feature of forest tree species. The differentiation of secondary phloem and secondary xylem from cambium cells are key steps of secondary growth, and the secondary vascular system plays essential roles in long distance transportation of water and nutrients. In this study, we identified an LBD transcription factor, PagLBD21, as an important regulator of secondary xylem development in *Populus*. Expression analysis found that *PagLBD21* expressed across the secondary phloem, cambium zone, and secondary xylem, but showed a higher level on the phloem side (Supplemental Fig. S1)^[31,32]^. Overexpression of *PagLBD21* (*PagLBD21OE*) reduced plant height and stem diameter (Fig. 2). Stem cross-section analysis showed that the lignification process in secondary xylem was delayed in *PagLBD21OE* plants, and the xylem width was significantly reduced in the 13^th^ internode (Fig. 3). Meanwhile, the phloem widths did not change significantly (Fig. 3). These results indicated the xylem development was suppressed by *PagLBD21* overexpression, which may cause the suppression of plant growth in general.

To further investigate the influences of PagLBD21 on secondary growth, we performed RNA-seq to identify differentially expressed genes (DEGs) in *PagLBD21OE* compared to WT plants. Because the *PagLBD21OE* L36 plants we used for RNA-seq displayed low expression (Fig. 2, Supplemental Table S2), we only identified 1,421 DEGs between *PagLBD21OE* and WT plants. However, GO enrichment analysis showed that biological pathways such as cell differentiation, xylem development, and plant-type secondary cell wall biogenesis were significantly enriched in down-regulated DEGs (Fig. 4a & b), furthermore, a group of key regulators and secondary cell wall biosynthesis genes were significantly down-regulated in *PagLBD21OE* plants, supporting the conclusion that *PagLBD21* overexpression suppressed xylem development. We also performed DAP-seq to identify PagLBD21 genome-wide binding sites and identified 1,639 unique target genes associated with PagLBD21 binding sites (Fig. 5). Distribution pattern analysis suggested the PagLBD21 DAP-seq data was specific. However, there were only 59 genes overlapped between DEGs from RNA-seq and target genes from DAP-seq (Fig. 5d), which may due to the low expression level of the transgenic line used for RNA-seq and low sequencing depth of DAP-seq.

Previously, we have reported another LBD transcription factor, PagLBD3, as an important regulator of secondary growth in *Populus*^[24]^. The overexpression plants of *PagLBD21* and *PagLBD3* displayed similar changes in many aspects, such as dwarf, hard rooting, and repression of xylem development. However, the phenotype of *PagLBD21OE* plants was not as strong as *PagLBD3OE* plants. There were also differences between *PagLBD21* and *PagLBD3* overexpressing plants: *PagLBD21OE* plants reduced stem diameter while *PagLBD3OE* plants increased stem diameter; *PagLBD21OE* plants had only reduced xylem width but regular xylem boundaries while *PagLBD3OE* plants had reduced xylem width with irregularly lignification and wider phloem and cortex. The phenotype differences may partially be caused by different expression levels of transgenic genes: the *PagLBD21OE* line used were weak lines with low expression level while *PagLBD3OE* line used were strong lines with high expression level.

We also compared the RNA-seq and DAP-seq datasets of PagLBD21 and PagLBD3. Results showed that there was a significant overlap between PagLBD21 DAP-seq & PagLBD3 DAP-seq, PagLBD21 DAP-seq & PagLBD3 RNA-seq, PagLBD21 RNA-seq & PagLBD3 DAP-seq, and PagLBD21 RNA-seq & PagLBD3 RNA-seq (Table 1), respectively. As the datasets related to PagLBD21 were much smaller than that related to PagLBD3, the common genes only account for a small percentage of PagLBD3 datasets. These results made it difficult to compare the differences between PagLBD21 and PagLBD3 signaling pathways. However, many genes encoding key regulators for secondary growth, such as auxin transporters, cytokinin biosynthesis, regulators for lignin biosynthesis, and the majority of SCW biosynthesis genes were targeted or regulated by both PagLBD21 and PagLBD3, indicating that PagLBD21 and PagLBD3 targeted a common group of genes during secondary growth. Our results provided information to investigate the functions of LBD family genes, as well as to further dissect the regulatory mechanisms underlying tree secondary growth and wood formation.

## SUPPLEMENTARY DATA

Supplementary data to this article can be found online.
