# Supplementary material for: Transcription factor PagLBD21 functions as a repressor of secondary xylem development in Populus
Source: For Res (Fayettev). 2022 Dec 21;2:19. doi: 10.48130/FR-2022-0019 (PMC11524276; doi:10.48130/FR-2022-0019)
Supplement: Supplementary file 1 — Supplementary data to this article can be found online. [file FR-2022-0019-S1.zip › 10.48130_FR-2022-0019-Suppl-TableS1.pdf]

SupplementalTable S1. Primers used for gene cloning and qPCR.

Primer name	Accession number
LBD21-31-KpnI-5’	
LBD21-31-XbaI-3’	
LBD21-31-qPCR-5’	Potri.010G186000.1
LBD21-31-qPCR-3’	
PzP211-35s-PolyA-F for cloning	
PzP211-35s-PolyA-R for cloning	
Actin-F for qRT-PCR	Potri.006G192700.1
Actin-R for qRT-PCR	

Primer sequences (5'—3')

GGTACCATGAGGAACCACGAGCCTCGT

TCTAGATTAAAATATATATGGGAATTGGCTG

GTGAAGACACGGTGAATT

TATCTGATGAATTCTGGCTAA

ACAGTGGTCCCAAAGATGGA

CCCAATGCCATAATACTCAA

AAGATTCCGTTGTCCAGAGGTCCT

GAACATAGTAGAGCCACCACTGAGAAC
