# Supplementary material for: Transcription factor PagLBD21 functions as a repressor of secondary xylem development in Populus
Source: For Res (Fayettev). 2022 Dec 21;2:19. doi: 10.48130/FR-2022-0019 (PMC11524276; doi:10.48130/FR-2022-0019)
Supplement: Supplementary file 1 — Supplementary data to this article can be found online. [file FR-2022-0019-S1.zip › 10.48130_FR-2022-0019-Suppl-TableS2.pdf]

Supplemental Table S2. DEGs between PagLBD21OE and WT

genes	logFC	logCPM	PValue	FDR	up/down	WT	WT	WT
Potri.006	-3.87797	3.737086	7.49E-37	3.22E-32	-1	489	244	234
Potri.009	-0.61921	3.222226	0.001644	0.049759	-1	150	146	137
Potri.012	-0.46393	5.302108	0.001627	0.049307	-1	754	496	541
Potri.006	-0.69881	2.541187	0.00162	0.049164	-1	99	93	81
Potri.017	-0.34837	8.335004	0.00161	0.048925	-1	4628	4818	4643
Potri.014	-0.47334	4.217706	0.001598	0.048737	-1	304	258	276
Potri.001	-0.61748	2.506131	0.00156	0.047688	-1	82	89	89
Potri.016	-0.99925	2.007843	0.001553	0.047514	-1	60	50	90
Potri.004	-0.6862	2.399933	0.001537	0.047254	-1	72	95	78
Potri.T11	-2.76475	2.736297	2.65E-28	2.27E-24	-1	151	159	128
Potri.016	-0.45462	5.986484	0.001517	0.046821	-1	884	1060	899
Potri.009	-0.37531	6.400678	0.001517	0.046821	-1	1481	1114	1142
Potri.013	-1.27496	4.790957	8.22E-24	5.88E-20	-1	543	506	470
Potri.005	-0.60486	2.679938	0.001491	0.046207	-1	103	83	108
Potri.002	-0.36205	6.787399	0.001481	0.046018	-1	1792	1583	1479
Potri.004	-2.07942	3.413293	2.82E-22	1.52E-18	-1	213	241	203
Potri.010	-3.05849	1.666163	1.53E-20	6.55E-17	-1	69	81	57
Potri.001	-0.49446	4.094973	0.001451	0.045363	-1	312	231	233
Potri.006	-0.70112	2.389603	0.001447	0.045292	-1	102	62	82
Potri.006	-0.51823	4.102917	0.001444	0.045275	-1	326	197	264
Potri.018	-0.32683	7.833673	0.001445	0.045275	-1	3410	3185	3310
Potri.001	-1.2656	7.13349	3.00E-20	1.17E-16	-1	3065	2420	2283
Potri.007	-2.18874	2.189752	5.02E-20	1.80E-16	-1	94	89	96
Potri.001	-1.99106	5.031189	9.95E-20	3.29E-16	-1	622	787	607
Potri.016	-0.38953	5.025612	0.001432	0.045063	-1	541	430	468
Potri.010	-0.90361	1.610713	0.00141	0.044624	-1	59	46	42
Potri.017	-0.54321	3.735662	0.001403	0.044471	-1	232	206	172
Potri.008	-2.31162	5.917547	1.47E-18	4.22E-15	-1	1304	1397	1204
Potri.005	-0.58965	7.501352	0.001395	0.044345	-1	4118	1912	2587
Potri.018	-0.62701	3.209869	0.001386	0.044218	-1	116	153	159
Potri.018	-2.22333	2.191267	1.36E-17	3.25E-14	-1	84	93	103
Potri.004	-0.37456	6.123059	0.001353	0.043381	-1	1131	1001	939
Potri.007	-0.47172	4.35802	0.001348	0.043284	-1	359	274	293
Potri.009	-2.47474	3.388679	3.68E-17	7.53E-14	-1	195	295	184
Potri.006	-0.53989	3.958022	0.001327	0.042746	-1	267	249	196
Potri.001	-0.53298	3.08634	0.001325	0.042728	-1	128	125	131
Potri.001	-0.61114	3.032776	0.001311	0.04237	-1	120	147	110
Potri.003	-0.54563	3.727711	0.001295	0.04196	-1	202	217	186
Potri.016	-0.84362	2.400486	0.001269	0.041212	-1	71	85	99
Potri.008	-0.92918	1.548146	0.001262	0.041153	-1	48	35	58
Potri.012	-1.14766	5.867865	5.54E-16	7.93E-13	-1	1132	1044	958
Potri.010	-0.41868	4.543823	0.00123	0.040439	-1	349	324	361
Potri.009	-2.23261	2.770109	2.49E-15	3.35E-12	-1	131	169	124
Potri.003	-0.73101	3.046863	0.001223	0.040327	-1	119	132	142
Potri.008	-0.70548	2.9262	0.001222	0.040298	-1	164	93	105
Potri.009	-0.51958	3.396068	0.001213	0.040163	-1	160	161	155
Potri.010	-0.77616	2.012043	0.001211	0.040141	-1	81	59	51
Potri.008	-0.51994	4.86078	0.001204	0.039955	-1	556	435	345
Potri.005	-0.44271	4.27048	0.001195	0.039872	-1	312	250	300
Potri.005	-0.39338	5.782048	0.001197	0.039872	-1	952	748	742
Potri.003	-0.81174	3.692501	0.001194	0.039871	-1	319	147	176

Potri.003	-0.3737	6.481266	0.00119	0.039798	-1	1335	1285	1308
Potri.006	-0.76862	1.912504	0.001188	0.039784	-1	68	61	48
Potri.005	-0.48778	4.206288	0.001188	0.039784	-1	311	224	301
Potri.019	-0.37741	5.729314	0.00118	0.039608	-1	852	752	733
Potri.007	-0.50417	4.266085	0.001179	0.039588	-1	327	277	271
Potri.001	-0.84518	1.410782	0.001171	0.039407	-1	47	38	40
Potri.009	-0.76114	2.580112	0.001172	0.039407	-1	103	112	70
Potri.013	-0.5108	3.804479	0.00117	0.039407	-1	260	196	180
Potri.014	-0.61616	3.453228	0.001166	0.039374	-1	147	187	173
Potri.017	-0.43943	4.323194	0.001167	0.039374	-1	344	261	290
Potri.005	-0.46274	6.261908	0.001156	0.039048	-1	1124	1067	1269
Potri.003	-0.52846	3.591735	0.001144	0.038692	-1	190	178	180
Potri.007	-1.79363	3.332394	1.58E-14	1.79E-11	-1	192	220	184
Potri.013	-0.46587	6.037852	0.001117	0.037942	-1	1039	1012	917
Potri.006	-0.44256	5.182526	0.001113	0.037852	-1	674	473	489
Potri.004	-1.09533	5.898313	2.82E-14	2.95E-11	-1	1113	1071	978
Potri.003	-0.63878	3.297433	0.001102	0.03764	-1	163	169	127
Potri.004	-0.36646	7.261809	0.001098	0.037536	-1	2193	2285	2250
Potri.009	-0.74618	3.219596	0.001096	0.037523	-1	193	110	147
Potri.001	-3.13512	3.626244	3.92E-14	4.01E-11	-1	214	399	228
Potri.010	-0.48642	5.948115	0.001087	0.03734	-1	795	1027	966
Potri.001	-0.36696	5.948762	0.001083	0.037244	-1	1029	833	856
Potri.008	-0.41681	4.755199	0.001081	0.037201	-1	403	409	385
Potri.018	-4.34427	1.948238	6.85E-14	6.84E-11	-1	126	27	120
Potri.006	-3.67759	0.815196	1.14E-13	1.11E-10	-1	44	47	23
Potri.014	-1.34138	5.469652	2.29E-13	2.14E-10	-1	1109	565	821
Potri.010	-0.40018	5.627239	0.001069	0.036992	-1	859	640	699
Potri.009	-3.61904	1.269807	4.07E-13	3.72E-10	-1	28	72	59
Potri.001	-0.44593	4.017667	0.001053	0.036534	-1	252	227	243
Potri.001	-1.42728	2.83498	4.39E-13	3.93E-10	-1	141	129	125
Potri.016	-0.72581	3.269722	0.001049	0.036462	-1	128	190	140
Potri.002	-0.60124	3.169002	0.001041	0.036244	-1	175	127	115
Potri.008	-0.57658	3.140712	0.001028	0.035839	-1	140	141	123
Potri.001	-0.37551	6.817241	0.001028	0.035839	-1	1936	1515	1536
Potri.004	-0.73841	2.406566	0.001019	0.035614	-1	100	80	71
Potri.019	-0.37564	6.969283	0.001018	0.035607	-1	1766	1857	1882
Potri.001	-0.41992	6.90144	0.001013	0.035476	-1	2006	1522	1826
Potri.016	-0.4446	7.564282	0.001011	0.035452	-1	3638	2368	2577
Potri.001	-0.44444	4.678353	0.001011	0.035452	-1	393	350	402
Potri.005	-0.7328	3.362914	0.001002	0.035217	-1	178	123	193
Potri.019	-0.42078	7.496723	0.000999	0.0352	-1	3267	2199	2652
Potri.009	-0.42169	5.289645	0.000997	0.035168	-1	606	582	553
Potri.009	-0.39685	5.447107	0.000997	0.035168	-1	722	573	639
Potri.010	-0.5491	3.533453	0.000995	0.035151	-1	201	176	153
Potri.001	-0.87554	3.380117	0.000993	0.035117	-1	147	175	193
Potri.002	-0.95884	1.332196	0.000989	0.035029	-1	41	36	44
Potri.002	-0.46794	4.020839	0.000983	0.034876	-1	271	243	215
Potri.010	-0.71312	2.4152	0.000972	0.034544	-1	82	84	84
Potri.T07	-0.39643	7.209608	0.000972	0.034544	-1	2112	2111	2325
Potri.016	-0.46345	4.352118	0.00097	0.034528	-1	331	269	318
Potri.005	-0.57828	3.714096	0.000967	0.03447	-1	255	167	187
Potri.001	-0.52087	5.051128	0.000967	0.03447	-1	538	519	461
Potri.013	-0.67898	2.827031	0.000949	0.033952	-1	106	117	109
Potri.004	-0.54051	3.909573	0.000939	0.033674	-1	257	195	237

Potri.010	-0.40841	5.408198	0.000931	0.033465	-1	639	635	608
Potri.009	-0.74952	2.184992	0.000923	0.033266	-1	72	74	68
Potri.004	-0.66726	2.997528	0.000919	0.033221	-1	140	109	126
Potri.019	-0.83944	1.968901	0.000903	0.032814	-1	55	57	75
Potri.008	-0.65462	2.369551	0.000903	0.032814	-1	91	72	76
Potri.007	-0.90591	2.168169	0.0009	0.032764	-1	83	80	57
Potri.015	-0.35277	6.422748	0.000895	0.032611	-1	1339	1200	1215
Potri.010	-0.42069	5.687065	0.000894	0.032604	-1	896	637	773
Potri.001	-0.46181	4.649842	0.000865	0.031761	-1	464	330	340
Potri.015	-1.93473	2.126491	2.45E-12	1.81E-09	-1	72	106	79
Potri.010	-0.38866	6.235802	0.000849	0.031301	-1	1326	985	1035
Potri.019	-1.07574	4.440191	2.44E-12	1.81E-09	-1	469	338	338
Potri.007	-0.40551	6.00418	0.000825	0.030602	-1	944	1025	874
Potri.013	-0.3821	7.79998	0.000816	0.030315	-1	3628	3013	3224
Potri.006	-0.61531	3.709977	0.000812	0.030254	-1	196	191	222
Potri.001	-3.23355	1.153929	2.78E-12	1.99E-09	-1	36	67	40
Potri.006	-0.76579	2.684201	0.000805	0.030047	-1	96	82	129
Potri.005	-2.85167	1.17118	3.13E-12	2.16E-09	-1	36	54	51
Potri.003	-0.71845	2.830597	0.000785	0.029428	-1	117	106	114
Potri.015	-0.46265	4.480355	0.000784	0.029428	-1	324	311	366
Potri.006	-0.50712	4.054521	0.000773	0.029145	-1	307	232	218
Potri.015	-0.44237	5.570131	0.000769	0.029031	-1	832	647	659
Potri.001	-0.50759	3.888072	0.000761	0.028803	-1	258	185	230
Potri.010	-0.46284	4.228686	0.000759	0.028738	-1	310	277	255
Potri.015	-0.4636	4.287848	0.000757	0.028703	-1	319	260	299
Potri.004	-0.59169	3.08855	0.000735	0.027917	-1	147	116	129
Potri.004	-1.54461	3.528254	3.29E-12	2.21E-09	-1	194	239	223
Potri.010	-0.39049	5.500803	0.000722	0.027518	-1	725	609	668
Potri.017	-0.45856	5.00461	0.000717	0.02734	-1	456	504	479
Potri.004	-0.50155	4.157757	0.000715	0.027283	-1	267	259	282
Potri.019	-0.34939	9.08361	0.000715	0.027283	-1	8029	7540	8151
Potri.016	-0.73962	2.807925	0.000709	0.027143	-1	99	137	96
Potri.006	-0.35506	7.789403	0.000691	0.026558	-1	3579	2951	3184
Potri.003	-0.48615	4.308343	0.00069	0.026521	-1	324	276	296
Potri.019	-1.51747	3.171228	4.42E-12	2.92E-09	-1	144	194	169
Potri.004	-0.42303	4.986564	0.000685	0.026391	-1	543	439	433
Potri.006	-1.26879	2.892251	4.74E-12	3.04E-09	-1	156	125	119
Potri.015	-0.41593	5.794331	0.000681	0.026288	-1	947	724	808
Potri.T08	-0.39833	5.344357	0.000673	0.026003	-1	610	604	581
Potri.010	-0.59125	5.402578	0.000663	0.025736	-1	587	826	552
Potri.008	-3.10096	1.456521	5.25E-12	3.27E-09	-1	87	64	28
Potri.011	-3.76003	0.873483	7.02E-12	4.31E-09	-1	24	60	35
Potri.001	-0.57131	4.041254	0.000643	0.025104	-1	303	192	269
Potri.005	-0.7276	2.213966	0.000641	0.02506	-1	67	79	71
Potri.008	-1.67363	2.282093	1.42E-11	8.22E-09	-1	86	92	99
Potri.007	-0.47456	4.889154	0.000606	0.023849	-1	486	451	402
Potri.004	-0.79784	2.702841	0.000599	0.023632	-1	120	69	126
Potri.015	-0.76043	4.656962	0.000593	0.023447	-1	318	513	388
Potri.014	-0.86717	2.275699	0.000591	0.02338	-1	90	60	86
Potri.002	-0.8084	2.161945	0.000589	0.023329	-1	78	64	72
Potri.001	-0.82616	1.74707	0.000584	0.023167	-1	54	50	55
Potri.003	-0.43239	5.061458	0.000583	0.023147	-1	502	482	505
Potri.014	-0.48258	4.585692	0.000581	0.02309	-1	360	354	370
Potri.007	-0.66512	4.439421	0.00057	0.022728	-1	351	386	292

Potri.003	-0.51647	4.126087	0.00057	0.022728	-1	318	232	248
Potri.011	-0.51258	4.120704	0.000566	0.022628	-1	255	270	264
Potri.014	-0.48756	4.974276	0.000556	0.022247	-1	538	438	453
Potri.005	-0.86009	4.118291	0.000547	0.022008	-1	224	365	269
Potri.019	-0.47819	5.243822	0.000543	0.021896	-1	715	472	539
Potri.014	-1.49337	2.411654	3.61E-11	1.99E-08	-1	92	89	114
Potri.002	-1.35071	2.892329	3.58E-11	1.99E-08	-1	129	120	156
Potri.001	-0.54611	5.968187	0.000527	0.021384	-1	852	1112	913
Potri.015	-1.55698	2.28397	7.84E-11	4.21E-08	-1	81	89	102
Potri.009	-0.7391	2.855223	0.000525	0.021343	-1	96	135	112
Potri.007	-1.42187	3.865865	8.54E-11	4.46E-08	-1	239	322	252
Potri.004	-0.57997	3.389362	0.000524	0.021309	-1	154	175	152
Potri.004	-0.53103	3.695942	0.000524	0.021309	-1	205	199	186
Potri.010	-0.62971	3.079683	0.00051	0.020906	-1	154	108	132
Potri.013	-0.48036	4.049937	0.000509	0.020886	-1	274	226	247
Potri.006	-0.42279	6.865276	0.000505	0.020735	-1	2024	1484	1723
Potri.006	-0.55188	3.48138	0.000504	0.020709	-1	174	178	158
Potri.007	-0.59394	3.006476	0.000501	0.020621	-1	125	127	117
Potri.012	-0.47323	4.174821	0.000499	0.020561	-1	303	238	273
Potri.011	-0.51966	4.318475	0.000492	0.020353	-1	290	290	328
Potri.016	-0.39699	5.781256	0.000492	0.020353	-1	827	809	796
Potri.001	-0.37485	7.09345	0.00049	0.02034	-1	2302	1879	1854
Potri.016	-6.77297	-0.49202	1.85E-10	9.13E-08	-1	17	17	8
Potri.019	-0.49031	4.374596	0.000481	0.020097	-1	297	319	320
Potri.005	-0.7166	3.347345	0.000478	0.02004	-1	186	130	171
Potri.018	-0.77088	2.652302	0.000475	0.019922	-1	96	123	81
Potri.003	-0.74221	2.484828	0.000474	0.019913	-1	95	97	73
Potri.014	-0.61413	4.93841	0.000472	0.019883	-1	552	421	472
Potri.T09	-3.52523	0.112361	4.62E-10	2.05E-07	-1	15	29	21
Potri.004	-0.39941	6.407688	0.000469	0.019826	-1	1353	1121	1296
Potri.001	-0.40877	6.6977	0.000468	0.019803	-1	1529	1504	1577
Potri.003	-0.437	4.766147	0.000466	0.01973	-1	448	378	391
Potri.002	-0.51027	4.954981	0.000464	0.019687	-1	460	486	466
Potri.015	-0.69634	3.03347	0.000458	0.019503	-1	124	140	122
Potri.012	-0.64652	6.521623	0.000458	0.019503	-1	2082	973	1374
Potri.006	-0.60873	3.688349	0.000459	0.019503	-1	189	215	194
Potri.003	-0.80638	2.289623	0.000453	0.019346	-1	96	66	73
Potri.008	-0.56579	4.302195	0.000452	0.019326	-1	385	250	282
Potri.007	-0.94813	2.240228	0.000438	0.01879	-1	64	79	90
Potri.010	-0.58436	3.404225	0.000434	0.018645	-1	157	161	169
Potri.004	-0.77593	2.345836	0.000429	0.018455	-1	103	67	73
Potri.002	-1.00118	5.622178	5.33E-10	2.26E-07	-1	885	844	825
Potri.008	-0.65897	2.992311	0.000424	0.018302	-1	115	139	117
Potri.001	-1.09081	3.592691	6.86E-10	2.89E-07	-1	193	205	231
Potri.002	-0.84611	3.422114	0.000417	0.018015	-1	255	136	143
Potri.003	-0.52717	3.734729	0.000414	0.01791	-1	213	198	195
Potri.005	-0.94076	1.870048	0.000408	0.017723	-1	47	63	68
Potri.013	-1.49272	1.733532	1.40E-09	5.58E-07	-1	63	58	60
Potri.001	-0.49661	4.130852	0.0004	0.017437	-1	302	233	260
Potri.014	-0.76166	3.881969	0.000397	0.017311	-1	325	160	237
Potri.003	-0.40514	5.436004	0.000397	0.017311	-1	709	583	631
Potri.015	-0.49959	4.063239	0.000394	0.017235	-1	255	253	248
Potri.008	-0.64845	3.090625	0.000393	0.017208	-1	128	137	132
Potri.014	-0.89093	2.414861	0.00039	0.017116	-1	82	86	93

Potri.010	-1.31428	4.374748	1.47E-09	5.81E-07	-1	497	232	425
Potri.002	-0.62827	3.077539	0.000387	0.017029	-1	153	116	124
Potri.005	-0.55581	4.206249	0.000386	0.017029	-1	308	260	283
Potri.008	-0.60685	3.246997	0.000377	0.016687	-1	149	151	139
Potri.016	-0.44206	5.728275	0.000378	0.016687	-1	836	808	733
Potri.014	-0.8407	2.100781	0.000372	0.016486	-1	88	58	61
Potri.012	-1.84566	2.482419	1.87E-09	7.23E-07	-1	99	133	96
Potri.015	-0.81113	3.064708	0.000363	0.016179	-1	166	116	127
Potri.001	-0.61881	3.462328	0.000353	0.015778	-1	169	185	158
Potri.015	-1.26801	4.170743	2.60E-09	9.79E-07	-1	280	419	277
Potri.005	-0.6001	4.164586	0.000341	0.015381	-1	246	285	301
Potri.001	-0.50902	6.058827	0.000341	0.015381	-1	999	1105	938
Potri.014	-1.44014	2.08857	3.76E-09	1.32E-06	-1	78	65	89
Potri.002	-0.40023	6.475172	0.000334	0.015207	-1	1365	1279	1301
Potri.001	-0.46319	4.520176	0.000333	0.015167	-1	376	334	322
Potri.010	-0.66743	2.821807	0.000327	0.014967	-1	105	102	123
Potri.013	-0.4919	4.861902	0.000325	0.014903	-1	511	442	370
Potri.005	-0.81174	2.05911	0.000321	0.014773	-1	76	61	62
Potri.008	-0.48234	4.425752	0.00032	0.014757	-1	371	295	307
Potri.001	-0.58717	4.850383	0.000316	0.014595	-1	394	509	434
Potri.004	-0.69537	2.62804	0.000309	0.01439	-1	118	85	88
Potri.002	-1.03671	3.210023	6.62E-09	2.11E-06	-1	166	144	166
Potri.001	-0.63427	3.175363	0.000297	0.013946	-1	165	122	135
Potri.006	-0.39348	7.403529	0.000294	0.013817	-1	2488	2384	2620
Potri.005	-1.42841	2.24758	6.88E-09	2.17E-06	-1	77	80	102
Potri.007	-1.21678	3.316892	1.04E-08	3.12E-06	-1	158	210	163
Potri.004	-1.02315	3.408474	1.35E-08	3.96E-06	-1	218	142	188
Potri.001	-0.87856	3.029737	0.000284	0.013415	-1	125	135	143
Potri.009	-0.7956	2.217801	0.000284	0.013415	-1	80	70	72
Potri.001	-1.98146	0.808612	1.42E-08	4.15E-06	-1	30	33	35
Potri.005	-0.52244	4.253255	0.000278	0.013207	-1	290	286	293
Potri.006	-0.40875	6.091339	0.000277	0.013169	-1	1099	977	958
Potri.007	-0.43665	5.151958	0.000274	0.013082	-1	576	511	504
Potri.001	-0.6622	3.444799	0.000274	0.013072	-1	200	159	155
Potri.006	-0.56201	3.801044	0.000274	0.013072	-1	256	206	181
Potri.007	-1.02564	2.880539	1.45E-08	4.20E-06	-1	132	120	124
Potri.004	-1.24339	4.841806	1.60E-08	4.57E-06	-1	421	628	502
Potri.016	-0.41361	7.785919	0.000262	0.01267	-1	3699	2840	3333
Potri.006	-0.78457	2.708084	0.000256	0.012441	-1	105	91	118
Potri.001	-1.22544	3.052928	1.73E-08	4.91E-06	-1	180	132	133
Potri.002	-0.81062	2.74085	0.000251	0.012245	-1	90	127	105
Potri.007	-1.17343	4.510188	1.79E-08	5.05E-06	-1	326	468	419
Potri.001	-0.62352	3.206022	0.000247	0.012065	-1	154	131	144
Potri.004	-0.67172	3.285445	0.000245	0.011977	-1	152	135	172
Potri.017	-0.5066	4.687404	0.00024	0.011794	-1	365	372	433
Potri.008	-0.64399	4.477002	0.000238	0.011732	-1	434	241	384
Potri.010	-0.80233	2.39233	0.000238	0.011728	-1	73	91	87
Potri.002	-0.74814	2.908518	0.000236	0.011646	-1	131	127	101
Potri.006	-0.38945	8.085736	0.000235	0.011615	-1	4050	4065	3896
Potri.016	-0.40979	7.163566	0.000234	0.011573	-1	2379	2075	1941
Potri.019	-2.04492	3.680571	2.04E-08	5.69E-06	-1	216	377	193
Potri.016	-1.16842	2.511931	2.69E-08	7.23E-06	-1	98	86	114
Potri.005	-1.60457	2.129072	2.78E-08	7.36E-06	-1	108	71	68
Potri.008	-0.4338	7.062808	0.000226	0.011225	-1	1946	2155	1875

Potri.019	-2.0381	2.316408	2.81E-08	7.39E-06	-1	72	142	84
Potri.004	-1.45228	1.929719	3.01E-08	7.84E-06	-1	86	55	67
Potri.001	-1.9027	0.908729	3.05E-08	7.87E-06	-1	44	27	34
Potri.008	-0.63904	3.452282	0.000222	0.011132	-1	167	191	153
Potri.003	-0.61805	4.076674	0.000222	0.011131	-1	346	224	225
Potri.014	-0.47088	4.714936	0.000222	0.011131	-1	422	358	405
Potri.017	-0.37239	7.942812	0.000221	0.011103	-1	3855	3491	3498
Potri.003	-1.33626	2.823601	3.08E-08	7.87E-06	-1	128	110	147
Potri.005	-0.60545	3.537509	0.000216	0.010904	-1	187	160	192
Potri.007	-0.95837	1.519281	0.000215	0.010897	-1	50	42	47
Potri.014	-1.09296	3.211246	3.08E-08	7.87E-06	-1	187	117	180
Potri.018	-0.42874	6.156778	0.000206	0.01047	-1	1069	1060	1057
Potri.005	-0.72692	3.806899	0.000199	0.010165	-1	214	227	230
Potri.009	-0.70447	3.262482	0.000196	0.010059	-1	192	133	133
Potri.003	-0.46742	5.655082	0.000195	0.009992	-1	821	657	802
Potri.019	-0.67426	3.128257	0.00019	0.00981	-1	169	115	129
Potri.010	-0.49172	5.165801	0.000186	0.009629	-1	668	496	475
Potri.005	-1.45525	3.45393	3.80E-08	9.42E-06	-1	170	234	208
Potri.013	-0.42816	5.967918	0.000182	0.009439	-1	1052	882	870
Potri.011	-0.92493	2.100934	0.000181	0.009403	-1	63	60	87
Potri.011	-0.65298	4.073855	0.00018	0.009396	-1	359	210	233
Potri.001	-0.57569	4.831037	0.000181	0.009396	-1	523	371	434
Potri.007	-0.46798	5.204825	0.00018	0.009396	-1	553	582	526
Potri.002	-1.04507	2.713601	4.17E-08	1.02E-05	-1	131	107	98
Potri.005	-0.42442	7.614746	0.000176	0.009226	-1	3248	2702	2836
Potri.001	-0.51955	5.873937	0.000172	0.009027	-1	805	1019	852
Potri.005	-1.55181	2.523132	4.62E-08	1.10E-05	-1	92	122	108
Potri.013	-0.69383	6.827092	0.000165	0.00871	-1	1780	1738	1944
Potri.012	-0.6953	3.560658	0.000164	0.008652	-1	223	168	172
Potri.006	-0.47533	4.98555	0.000162	0.00859	-1	458	477	493
Potri.001	-0.75869	2.329501	0.00016	0.008516	-1	79	78	81
Potri.012	-0.60164	3.273356	0.00016	0.008506	-1	158	149	140
Potri.007	-0.4826	6.227142	0.000159	0.008484	-1	1033	1145	1211
Potri.003	-0.61867	6.308178	0.000158	0.008439	-1	1619	943	1211
Potri.002	-0.71892	2.876799	0.000156	0.008383	-1	114	100	134
Potri.005	-0.51265	5.427702	0.000155	0.008375	-1	769	546	662
Potri.003	-0.72716	3.450757	0.000153	0.008271	-1	234	159	134
Potri.001	-2.37458	0.275866	4.69E-08	1.11E-05	-1	24	20	24
Potri.007	-0.56334	5.786903	0.000153	0.008254	-1	801	1009	742
Potri.010	-0.66664	8.025793	0.000152	0.008207	-1	3827	4425	4174
Potri.006	-0.43383	5.592608	0.000152	0.008207	-1	756	715	687
Potri.007	-0.48344	4.796896	0.000151	0.008187	-1	466	410	384
Potri.006	-1.22662	2.908646	5.01E-08	1.17E-05	-1	160	94	148
Potri.011	-0.99695	1.547481	0.00015	0.008156	-1	47	41	55
Potri.001	-0.63476	3.57167	0.00015	0.008156	-1	204	182	171
Potri.009	-1.114	6.062991	6.59E-08	1.51E-05	-1	949	1380	1202
Potri.011	-0.74288	3.111409	0.000147	0.008053	-1	176	107	133
Potri.005	-0.96249	1.851921	0.000143	0.007894	-1	77	44	57
Potri.014	-1.48352	2.996196	8.04E-08	1.76E-05	-1	107	183	154
Potri.001	-0.4958	5.797962	0.000136	0.007523	-1	869	831	833
Potri.005	-0.58483	4.696125	0.000135	0.007475	-1	407	357	442
Potri.006	-0.50468	4.733937	0.000134	0.007472	-1	430	370	412
Potri.001	-0.46957	4.776263	0.000134	0.007472	-1	444	403	389
Potri.005	-1.73172	2.551642	8.60E-08	1.86E-05	-1	125	61	155

Potri.001	-0.51172	4.595624	0.000132	0.007402	-1	376	367	358
Potri.007	-0.50809	4.508454	0.000131	0.007363	-1	358	308	370
Potri.013	-0.4471	7.393191	0.000131	0.007363	-1	2461	2443	2648
Potri.009	-1.80422	3.004138	1.02E-07	2.16E-05	-1	100	199	171
Potri.010	-0.6309	3.704769	0.000127	0.00719	-1	213	212	185
Potri.004	-1.13119	4.601444	1.26E-07	2.62E-05	-1	642	294	372
Potri.009	-0.66464	3.301246	0.000124	0.007071	-1	155	161	147
Potri.018	-0.57368	4.755013	0.000122	0.006981	-1	534	332	396
Potri.018	-0.50297	4.535215	0.000121	0.006938	-1	410	331	316
Potri.010	-4.64696	-0.63094	1.41E-07	2.88E-05	-1	13	17	6
Potri.008	-1.16406	3.736152	1.53E-07	3.11E-05	-1	191	288	225
Potri.T11	-0.86897	3.006554	0.000116	0.006644	-1	106	157	131
Potri.003	-0.50984	5.842655	0.000114	0.006569	-1	811	872	933
Potri.014	-0.4238	6.60115	0.000113	0.006556	-1	1451	1454	1426
Potri.010	-0.60711	5.576769	0.000113	0.006532	-1	713	696	828
Potri.015	-0.65846	3.695195	0.000111	0.006489	-1	177	217	214
Potri.010	-2.29565	0.64994	1.67E-07	3.31E-05	-1	22	42	26
Potri.014	-1.11171	3.82359	1.76E-07	3.43E-05	-1	219	275	248
Potri.002	-0.46859	6.088222	0.000107	0.006322	-1	1079	1062	935
Potri.006	-0.59253	3.882952	0.000105	0.006188	-1	235	228	221
Potri.007	-0.5271	4.118495	0.000104	0.006177	-1	280	262	251
Potri.008	-0.44543	6.491986	0.000104	0.006159	-1	1521	1256	1280
Potri.013	-1.05501	5.035228	2.15E-07	4.11E-05	-1	793	364	581
Potri.010	-1.18662	4.602913	2.32E-07	4.36E-05	-1	311	493	491
Potri.T05	-0.6433	6.326917	0.000102	0.006147	-1	1306	1336	1168
Potri.001	-1.01874	2.474847	2.32E-07	4.36E-05	-1	96	97	88
Potri.014	-1.45533	3.534043	2.48E-07	4.57E-05	-1	146	202	298
Potri.002	-0.60368	4.136589	9.60E-05	0.005819	-1	245	277	295
Potri.006	-2.06377	3.719631	2.63E-07	4.79E-05	-1	167	400	239
Potri.008	-0.66442	3.230829	9.30E-05	0.005702	-1	170	145	127
Potri.003	-0.63823	4.632977	9.31E-05	0.005702	-1	398	400	372
Potri.003	-0.70998	3.208474	9.15E-05	0.005623	-1	136	166	136
Potri.009	-0.5774	4.154284	8.97E-05	0.005518	-1	330	230	268
Potri.013	-0.45573	7.946559	8.80E-05	0.00544	-1	4244	3099	3838
Potri.002	-1.46237	1.486327	2.63E-07	4.79E-05	-1	45	46	59
Potri.013	-2.4245	0.093273	3.02E-07	5.43E-05	-1	21	21	17
Potri.003	-0.65944	2.968201	8.70E-05	0.005401	-1	129	112	125
Potri.016	-0.50465	5.0246	8.70E-05	0.005401	-1	511	514	457
Potri.002	-0.79191	3.990281	8.52E-05	0.005327	-1	228	285	261
Potri.008	-0.95817	1.866137	8.38E-05	0.005274	-1	66	62	51
Potri.006	-1.12007	3.374216	3.89E-07	6.71E-05	-1	233	117	198
Potri.005	-1.55039	1.260528	4.03E-07	6.90E-05	-1	44	48	37
Potri.017	-1.35149	2.40108	4.06E-07	6.91E-05	-1	107	103	76
Potri.012	-0.49605	4.786454	8.30E-05	0.005247	-1	420	403	429
Potri.012	-0.3932	10.06187	8.16E-05	0.00521	-1	16456	15243	15695
Potri.001	-1.24878	5.238923	4.14E-07	7.00E-05	-1	559	749	742
Potri.010	-0.89192	3.626745	7.80E-05	0.004998	-1	159	238	216
Potri.013	-0.70233	4.087148	7.81E-05	0.004998	-1	303	194	319
Potri.018	-0.66843	4.561609	7.81E-05	0.004998	-1	315	441	360
Potri.016	-0.76664	7.76737	7.59E-05	0.004905	-1	4343	3191	3237
Potri.001	-0.50504	5.018607	7.54E-05	0.004884	-1	593	440	451
Potri.019	-2.38374	0.180286	4.19E-07	7.06E-05	-1	21	21	21
Potri.006	-0.70617	5.461544	7.40E-05	0.004815	-1	727	637	761
Potri.007	-2.20053	0.575166	4.87E-07	8.02E-05	-1	18	32	34

Potri.010	-0.70568	6.412507	7.30E-05	0.004783	-1	1009	1386	1683
Potri.008	-0.44044	7.807973	7.32E-05	0.004783	-1	3740	2945	3414
Potri.008	-0.78517	2.915856	7.13E-05	0.004712	-1	141	106	118
Potri.004	-1.87603	1.029771	5.53E-07	9.00E-05	-1	33	51	30
Potri.007	-0.4995	8.251177	6.98E-05	0.004642	-1	4527	4769	4601
Potri.019	-0.77054	2.70206	6.83E-05	0.004551	-1	98	108	105
Potri.T05	-0.74152	3.104253	6.76E-05	0.004518	-1	140	150	121
Potri.019	-0.41213	9.366868	6.66E-05	0.004476	-1	10781	8991	9728
Potri.015	-0.73372	2.783581	6.52E-05	0.004397	-1	113	112	102
Potri.013	-0.8561	4.916857	6.48E-05	0.004379	-1	570	526	417
Potri.003	-0.7836	3.562829	6.47E-05	0.004373	-1	191	194	189
Potri.017	-0.91837	1.99254	6.33E-05	0.004298	-1	63	67	64
Potri.017	-1.45618	1.31499	7.39E-07	0.000115	-1	41	50	41
Potri.015	-0.46369	6.565037	6.23E-05	0.004239	-1	1590	1284	1416
Potri.009	-0.49926	4.815857	6.18E-05	0.004211	-1	426	410	443
Potri.005	-0.54551	6.056945	6.11E-05	0.004169	-1	1207	858	1027
Potri.001	-0.52937	4.7834	6.00E-05	0.004119	-1	461	368	436
Potri.004	-1.53456	1.146047	8.59E-07	0.000129	-1	36	45	37
Potri.010	-2.31751	0.129618	9.82E-07	0.000144	-1	18	22	20
Potri.005	-0.64177	5.302218	5.68E-05	0.003931	-1	771	471	640
Potri.010	-0.49043	5.414903	5.30E-05	0.003693	-1	763	565	619
Potri.001	-0.57302	5.75023	5.28E-05	0.003691	-1	959	770	785
Potri.001	-1.33603	2.698117	9.93E-07	0.000145	-1	157	124	73
Potri.016	-0.53141	5.084979	5.24E-05	0.003678	-1	510	507	539
Potri.003	-1.11215	2.393036	1.10E-06	0.000159	-1	119	74	79
Potri.014	-0.84672	2.541504	5.15E-05	0.003629	-1	111	77	96
Potri.T13	-0.98467	1.763424	5.13E-05	0.003621	-1	56	51	60
Potri.001	-1.46901	1.099163	1.24E-06	0.000173	-1	43	30	40
Potri.001	-1.1439	2.932477	1.25E-06	0.000174	-1	107	152	139
Potri.013	-0.49151	6.050316	4.85E-05	0.00347	-1	1143	864	1020
Potri.002	-0.56269	5.344383	4.80E-05	0.003445	-1	666	682	535
Potri.008	-0.63895	3.954555	4.67E-05	0.003371	-1	247	253	228
Potri.013	-0.69388	3.145737	4.65E-05	0.003371	-1	155	136	128
Potri.001	-0.56195	4.096389	4.57E-05	0.003314	-1	294	233	263
Potri.002	-1.48547	1.312806	1.42E-06	0.000193	-1	53	33	47
Potri.T05	-0.57638	6.323684	4.46E-05	0.003255	-1	1145	1393	1181
Potri.002	-1.28948	1.616772	1.60E-06	0.000212	-1	55	52	53
Potri.002	-0.71326	3.178956	4.23E-05	0.003097	-1	141	148	141
Potri.009	-0.56834	5.525684	4.11E-05	0.003026	-1	683	761	690
Potri.005	-0.75999	3.214344	4.01E-05	0.002969	-1	158	141	148
Potri.002	-0.49515	5.464674	4.02E-05	0.002969	-1	759	576	681
Potri.007	-0.9743	4.291315	3.99E-05	0.002968	-1	270	405	321
Potri.001	-0.80805	2.41324	3.90E-05	0.00291	-1	88	80	88
Potri.010	-1.50726	1.230516	1.72E-06	0.000225	-1	36	47	42
Potri.017	-0.94864	3.286415	3.52E-05	0.002659	-1	185	110	199
Potri.008	-0.65809	8.914153	3.48E-05	0.002637	-1	8203	7057	7824
Potri.013	-0.60348	5.364709	3.47E-05	0.002637	-1	560	730	631
Potri.010	-0.52803	6.416446	3.42E-05	0.002606	-1	1402	1371	1159
Potri.014	-1.05836	3.301683	1.79E-06	0.000233	-1	148	186	174
Potri.005	-5.91741	-1.11553	1.86E-06	0.00024	-1	8	9	6
Potri.013	-1.02506	2.14368	1.89E-06	0.000244	-1	79	76	67
Potri.007	-0.52221	4.910651	3.29E-05	0.002546	-1	474	418	485
Potri.008	-1.13513	2.379356	1.91E-06	0.000245	-1	106	80	84
Potri.011	-0.9941	2.614251	3.21E-05	0.002498	-1	141	72	98

Potri.006	-1.12559	4.1793	2.02E-06	0.000256	-1	235	379	337
Potri.017	-1.77243	1.961314	2.27E-06	0.000286	-1	47	103	72
Potri.007	-0.71259	5.324201	3.13E-05	0.002445	-1	561	726	636
Potri.014	-0.7715	6.525677	3.11E-05	0.00244	-1	1481	1328	1715
Potri.009	-2.21168	0.353832	2.65E-06	0.000326	-1	23	17	31
Potri.019	-0.56209	4.969769	2.88E-05	0.002276	-1	466	510	471
Potri.004	-1.14755	3.958255	2.80E-06	0.00034	-1	409	236	190
Potri.001	-1.32983	1.260367	2.93E-06	0.000353	-1	42	38	44
Potri.002	-1.18194	2.393768	3.07E-06	0.000368	-1	86	113	75
Potri.002	-1.1342	1.876436	3.08E-06	0.000368	-1	56	64	67
Potri.019	-1.36685	1.397053	3.46E-06	0.000405	-1	45	56	37
Potri.008	-0.66695	3.951674	2.52E-05	0.002045	-1	248	248	236
Potri.015	-1.42592	1.101525	3.63E-06	0.000422	-1	35	43	34
Potri.004	-1.04601	4.13805	3.65E-06	0.000422	-1	443	190	294
Potri.004	-2.96626	-0.544	4.12E-06	0.000464	-1	11	15	10
Potri.001	-0.48413	5.782281	2.44E-05	0.002014	-1	871	846	781
Potri.014	-1.11453	2.543277	4.21E-06	0.000473	-1	127	71	105
Potri.018	-0.57081	4.160259	2.33E-05	0.001939	-1	283	264	279
Potri.004	-0.45979	8.518899	2.33E-05	0.001938	-1	5792	5156	5642
Potri.001	-0.54976	4.871831	2.29E-05	0.00192	-1	466	438	446
Potri.010	-1.30725	1.607983	4.44E-06	0.000496	-1	67	52	41
Potri.006	-0.92868	2.748867	2.22E-05	0.001871	-1	103	113	118
Potri.012	-0.68774	3.903128	2.22E-05	0.001871	-1	265	193	256
Potri.008	-0.5279	6.416613	2.08E-05	0.001765	-1	1319	1430	1175
Potri.006	-0.8525	2.942446	1.93E-05	0.001652	-1	111	127	138
Potri.004	-0.63226	4.003632	1.93E-05	0.001652	-1	252	252	248
Potri.008	-0.70146	3.16113	1.90E-05	0.001634	-1	148	133	143
Potri.004	-0.67759	3.654733	1.83E-05	0.001581	-1	209	176	212
Potri.010	-1.21689	4.890178	5.22E-06	0.000567	-1	363	654	573
Potri.002	-0.54215	4.643873	1.77E-05	0.00154	-1	411	378	361
Potri.002	-1.13043	1.94376	5.40E-06	0.000582	-1	57	74	65
Potri.005	-2.58858	-0.05375	6.03E-06	0.000635	-1	11	24	18
Potri.019	-0.78849	3.422752	1.57E-05	0.001398	-1	216	137	171
Potri.002	-0.79686	3.300972	1.43E-05	0.001281	-1	144	158	176
Potri.008	-1.63217	0.560878	8.21E-06	0.000824	-1	28	27	22
Potri.007	-0.97186	6.028766	1.34E-05	0.001208	-1	845	1342	1147
Potri.003	-0.49397	7.255287	1.33E-05	0.001205	-1	2622	2021	2350
Potri.013	-0.74385	4.10534	1.32E-05	0.001196	-1	363	257	218
Potri.001	-0.50673	6.64245	1.31E-05	0.001191	-1	1585	1532	1453
Potri.002	-0.95283	2.371896	1.29E-05	0.001178	-1	103	73	82
Potri.007	-1.37689	1.109266	9.03E-06	0.000888	-1	45	31	36
Potri.006	-0.80242	2.78556	1.26E-05	0.00116	-1	125	93	116
Potri.001	-0.79479	3.241011	1.26E-05	0.001156	-1	156	152	151
Potri.002	-0.80174	3.090196	1.22E-05	0.001123	-1	148	123	143
Potri.015	-0.91693	2.963801	1.21E-05	0.001115	-1	110	156	121
Potri.001	-0.82224	3.163225	1.21E-05	0.001115	-1	165	110	164
Potri.005	-1.3125	1.859122	9.36E-06	0.00091	-1	48	68	75
Potri.011	-0.49131	5.873835	1.16E-05	0.001082	-1	964	873	834
Potri.019	-2.88557	-0.60102	9.51E-06	0.00092	-1	10	9	15
Potri.001	-0.63718	5.290515	1.10E-05	0.001027	-1	769	591	502
Potri.018	-1.91472	0.450021	9.99E-06	0.000952	-1	36	15	23
Potri.006	-0.59197	5.156244	1.08E-05	0.001012	-1	655	525	492
Potri.003	-1.00448	2.090178	1.04E-05	0.000984	-1	84	70	59
Potri.014	-1.20073	1.532929	1.08E-05	0.001015	-1	54	41	53

Potri.011	-0.87764	3.08206	9.92E-06	0.000947	-1	177	120	124
Potri.004	-0.96946	3.339124	9.85E-06	0.000946	-1	127	170	213
Potri.003	-0.8864	3.667253	9.89E-06	0.000946	-1	181	244	206
Potri.019	-0.63282	4.850596	9.75E-06	0.000941	-1	506	389	470
Potri.014	-1.31084	2.194533	1.10E-05	0.00103	-1	63	108	72
Potri.010	-1.25987	1.895245	1.16E-05	0.001082	-1	48	69	77
Potri.013	-0.97462	4.996132	9.27E-06	0.000903	-1	592	565	486
Potri.016	-0.78437	2.917517	9.25E-06	0.000903	-1	120	121	123
Potri.011	-0.88768	3.711531	9.12E-06	0.000892	-1	303	193	164
Potri.009	-0.60024	6.418584	9.11E-06	0.000892	-1	1352	1448	1210
Potri.001	-1.27653	1.389494	1.27E-05	0.001161	-1	43	36	56
Potri.005	-0.52822	5.247956	8.90E-06	0.000877	-1	588	573	582
Potri.010	-0.59619	4.666745	8.67E-06	0.000862	-1	434	380	373
Potri.002	-0.76727	6.419735	8.39E-06	0.000837	-1	1234	1330	1620
Potri.010	-2.63127	-0.54423	1.43E-05	0.00128	-1	10	13	12
Potri.002	-0.80196	5.847191	8.05E-06	0.000809	-1	1023	912	916
Potri.011	-1.1034	2.607957	1.43E-05	0.00128	-1	77	118	118
Potri.004	-1.3047	1.302025	1.59E-05	0.00141	-1	37	48	42
Potri.004	-0.8293	5.811825	6.80E-06	0.000697	-1	750	1092	933
Potri.007	-0.63465	3.967555	6.46E-06	0.000673	-1	248	250	236
Potri.018	-1.05274	2.751579	1.69E-05	0.001485	-1	106	134	104
Potri.001	-0.97827	3.169961	5.89E-06	0.000624	-1	141	175	139
Potri.010	-0.61436	5.306756	5.79E-06	0.000617	-1	650	623	588
Potri.010	-0.54821	6.453177	5.66E-06	0.000608	-1	1271	1409	1362
Potri.012	-0.79176	2.982573	5.49E-06	0.000591	-1	144	120	119
Potri.009	-1.07793	2.978093	1.72E-05	0.001501	-1	108	170	127
Potri.008	-0.69611	5.981349	4.88E-06	0.000534	-1	1285	779	1002
Potri.010	-0.80785	3.451392	4.80E-06	0.000527	-1	188	157	190
Potri.005	-0.49454	7.551986	4.72E-06	0.00052	-1	2884	2786	2887
Potri.006	-0.78506	5.045792	4.71E-06	0.00052	-1	711	418	508
Potri.002	-0.58163	6.121173	4.54E-06	0.000503	-1	1307	895	1067
Potri.012	-0.88689	3.038988	4.51E-06	0.000501	-1	123	144	139
Potri.T16	-4.00788	-1.07524	2.24E-05	0.001876	-1	9	9	5
Potri.010	-0.84388	2.806642	4.26E-06	0.000477	-1	124	113	105
Potri.008	-1.49634	0.624521	2.45E-05	0.002021	-1	26	26	27
Potri.013	-0.48965	9.560726	4.00E-06	0.000455	-1	12079	10905	11477
Potri.011	-0.83459	3.565951	3.94E-06	0.00045	-1	252	156	179
Potri.010	-1.74895	0.262042	2.47E-05	0.002025	-1	22	20	20
Potri.009	-0.65071	5.839315	3.71E-06	0.000426	-1	1145	806	790
Potri.005	-0.53521	6.367033	3.70E-06	0.000426	-1	1241	1299	1258
Potri.016	-1.4077	0.951807	2.48E-05	0.002037	-1	40	36	24
Potri.004	-1.11312	2.045407	2.49E-05	0.002039	-1	98	62	52
Potri.006	-2.33619	1.504841	2.51E-05	0.002045	-1	67	68	38
Potri.002	-1.3295	1.169068	2.52E-05	0.002045	-1	46	43	27
Potri.001	-1.53774	2.348601	2.71E-05	0.002179	-1	70	119	94
Potri.004	-1.94835	1.735985	2.73E-05	0.002187	-1	34	107	52
Potri.016	-1.04324	3.040725	2.81E-05	0.002237	-1	123	164	134
Potri.003	-1.24835	1.23633	2.87E-05	0.002274	-1	46	43	31
Potri.018	-0.61351	5.231204	2.68E-06	0.000327	-1	590	510	665
Potri.006	-4.00124	-1.07632	3.10E-05	0.002436	-1	12	6	5
Potri.001	-0.49736	6.742809	2.64E-06	0.000326	-1	1740	1538	1615
Potri.016	-0.6171	6.944742	2.49E-06	0.000308	-1	1936	1943	1928
Potri.006	-0.87064	3.504264	2.47E-06	0.000306	-1	204	176	184
Potri.017	-1.91247	0.306669	3.14E-05	0.002451	-1	29	13	24

Potri.002	-1.63641	0.383082	3.20E-05	0.002494	-1	21	22	24
Potri.010	-0.57923	5.471732	2.01E-06	0.000256	-1	816	611	650
Potri.008	-0.53798	6.877833	2.00E-06	0.000255	-1	1796	1744	1884
Potri.009	-1.04324	2.908734	3.24E-05	0.002515	-1	181	114	93
Potri.006	-0.69206	4.4656	1.90E-06	0.000244	-1	381	331	346
Potri.002	-1.51245	1.131792	3.38E-05	0.002585	-1	27	48	41
Potri.019	-1.63289	0.441463	4.22E-05	0.003097	-1	18	24	28
Potri.002	-0.90149	3.129557	1.54E-06	0.000206	-1	173	138	126
Potri.002	-0.68999	3.891161	1.50E-06	0.000201	-1	265	215	228
Potri.001	-0.82292	3.440419	1.46E-06	0.000198	-1	177	173	182
Potri.019	-1.27682	1.254077	4.52E-05	0.003296	-1	36	36	50
Potri.015	-1.86098	0.324279	4.53E-05	0.0033	-1	16	28	22
Potri.005	-1.30999	1.384681	4.56E-05	0.003312	-1	37	56	42
Potri.014	-0.67851	6.054964	1.33E-06	0.000182	-1	1374	913	925
Potri.012	-1.29413	1.233997	4.73E-05	0.003407	-1	46	28	47
Potri.004	-0.57524	6.201165	1.26E-06	0.000176	-1	1257	966	1216
Potri.018	-1.73519	0.246635	4.86E-05	0.003476	-1	17	25	19
Potri.012	-1.08738	6.406832	4.89E-05	0.003483	-1	988	1909	1539
Potri.016	-0.71134	5.909298	1.22E-06	0.000172	-1	1021	981	903
Potri.001	-0.95512	3.063317	1.17E-06	0.000167	-1	127	130	163
Potri.009	-0.56398	7.197263	1.15E-06	0.000165	-1	2148	2334	2325
Potri.015	-2.93066	-0.85263	5.19E-05	0.003652	-1	10	9	8
Potri.001	-2.64354	-0.33865	5.82E-05	0.004016	-1	12	7	23
Potri.007	-0.53861	9.579096	9.64E-07	0.000142	-1	11803	11890	11630
Potri.017	-0.67689	5.511981	9.21E-07	0.000137	-1	861	650	682
Potri.014	-1.21168	1.508555	5.98E-05	0.004113	-1	37	48	60
Potri.017	-0.65637	4.548705	8.08E-07	0.000123	-1	437	324	353
Potri.001	-0.97416	6.029428	7.76E-07	0.000118	-1	902	1297	1144
Potri.004	-1.08339	1.585227	6.70E-05	0.004491	-1	56	52	42
Potri.010	-1.73882	0.324406	7.02E-05	0.004661	-1	19	16	30
Potri.010	-0.84061	4.070334	6.48E-07	0.000102	-1	314	219	302
Potri.001	-0.91616	3.010772	6.27E-07	9.90E-05	-1	157	122	124
Potri.016	-2.25633	-0.43817	7.06E-05	0.004672	-1	12	15	10
Potri.001	-0.53862	7.015724	4.93E-07	8.08E-05	-1	2233	1837	1924
Potri.001	-1.73687	0.247019	7.35E-05	0.0048	-1	15	22	24
Potri.009	-0.59484	6.413811	4.77E-07	7.89E-05	-1	1451	1258	1295
Potri.001	-1.4818	0.509622	7.43E-05	0.004829	-1	21	28	23
Potri.015	-1.31197	1.961807	8.35E-05	0.005268	-1	42	89	74
Potri.006	-0.54645	7.189445	3.70E-07	6.49E-05	-1	2255	2272	2222
Potri.017	-1.79316	0.823251	8.66E-05	0.005397	-1	19	51	26
Potri.001	-1.7138	0.380301	8.76E-05	0.005431	-1	33	14	21
Potri.002	-0.60142	6.238102	3.00E-07	5.42E-05	-1	1294	1146	1110
Potri.006	-1.29499	0.8714	9.34E-05	0.005715	-1	33	34	25
Potri.014	-1.28503	1.04704	9.58E-05	0.005819	-1	39	24	42
Potri.012	-1.03014	2.605467	9.67E-05	0.005849	-1	151	89	72
Potri.019	-0.68445	8.091765	2.18E-07	4.12E-05	-1	5460	3398	4369
Potri.008	-0.63263	5.733476	1.80E-07	3.50E-05	-1	868	845	803
Potri.014	-2.04707	-0.17839	0.000108	0.006338	-1	12	22	11
Potri.002	-0.60408	6.42603	1.75E-07	3.43E-05	-1	1479	1214	1358
Potri.008	-1.23125	1.221529	0.000108	0.006349	-1	35	36	47
Potri.004	-0.73593	7.018647	1.56E-07	3.14E-05	-1	2444	1937	1962
Potri.008	-1.42306	2.767864	0.000112	0.006512	-1	70	180	122
Potri.001	-2.41269	-0.49038	0.000116	0.006644	-1	15	14	7
Potri.010	-1.56991	0.277301	0.00012	0.006867	-1	23	22	16

Potri.018	-1.66886	0.130396	0.000123	0.006997	-1	15	19	21
Potri.016	-1.06702	1.443565	0.000133	0.007443	-1	58	42	35
Potri.003	-2.85443	-0.01313	0.000139	0.0077	-1	7	30	19
Potri.008	-0.63917	6.461415	8.04E-08	1.76E-05	-1	1310	1386	1474
Potri.016	-0.75911	4.413281	7.63E-08	1.69E-05	-1	379	339	320
Potri.001	-0.9754	2.800004	7.23E-08	1.62E-05	-1	124	116	111
Potri.T02	-5.31642	-1.48883	0.000147	0.00806	-1	4	7	4
Potri.010	-0.99277	3.842518	5.85E-08	1.34E-05	-1	212	267	253
Potri.019	-0.63427	8.842316	5.45E-08	1.26E-05	-1	8676	6484	6748
Potri.001	-3.74542	-1.24369	0.00015	0.008165	-1	4	9	6
Potri.001	-2.17225	-0.48958	0.000153	0.008254	-1	10	10	15
Potri.015	-1.12086	3.074609	0.000165	0.00871	-1	243	94	110
Potri.001	-0.84924	6.274699	4.63E-08	1.10E-05	-1	1354	1225	1301
Potri.015	-0.88067	4.577392	4.32E-08	1.05E-05	-1	360	448	385
Potri.001	-0.79283	4.083285	4.34E-08	1.05E-05	-1	286	256	288
Potri.012	-0.60049	7.625639	3.97E-08	9.74E-06	-1	3178	2980	3125
Potri.005	-0.77161	5.05625	3.83E-08	9.47E-06	-1	588	476	568
Potri.008	-5.39476	-1.43806	0.000184	0.009532	-1	9	4	3
Potri.010	-5.3181	-1.48876	0.000194	0.009981	-1	4	8	3
Potri.010	-1.25931	1.369541	0.000211	0.010722	-1	35	62	35
Potri.T15	-3.66734	-1.28955	0.000223	0.011148	-1	4	8	6
Potri.008	-1.86292	0.10947	0.000225	0.011214	-1	27	10	19
Potri.008	-0.6071	6.705963	3.01E-08	7.84E-06	-1	1827	1606	1491
Potri.009	-1.04745	1.431863	0.000226	0.011256	-1	47	32	54
Potri.T07	-0.86985	3.387269	2.74E-08	7.31E-06	-1	198	155	167
Potri.008	-1.73518	0.398686	0.000229	0.011386	-1	25	26	18
Potri.003	-0.72407	4.783236	2.24E-08	6.13E-06	-1	451	459	419
Potri.002	-1.6261	0.65853	0.000247	0.012065	-1	34	36	13
Potri.010	-5.21119	-1.54437	0.000265	0.012774	-1	7	4	3
Potri.009	-0.62437	6.029115	1.55E-08	4.47E-06	-1	1078	1024	983
Potri.010	-2.56505	-0.81776	0.000272	0.013043	-1	6	11	10
Potri.013	-2.29723	-0.57225	0.000281	0.013325	-1	10	8	15
Potri.014	-1.11794	1.287278	0.000287	0.013516	-1	35	40	46
Potri.001	-1.81756	0.371985	0.000291	0.013712	-1	13	35	20
Potri.006	-0.67645	6.036515	9.22E-09	2.77E-06	-1	1165	1040	945
Potri.001	-0.83854	4.049635	7.56E-09	2.34E-06	-1	307	239	276
Potri.002	-0.94476	4.443899	7.01E-09	2.20E-06	-1	338	409	357
Potri.007	-2.25107	-0.43628	0.000293	0.013763	-1	9	12	16
Potri.001	-1.04052	1.336503	0.000306	0.014268	-1	53	38	33
Potri.013	-1.5755	0.146643	0.000322	0.014773	-1	21	17	17
Potri.009	-0.99866	4.873567	4.33E-09	1.47E-06	-1	473	499	539
Potri.003	-1.98732	-0.4646	0.000332	0.015167	-1	14	11	10
Potri.018	-0.6802	5.343605	4.16E-09	1.44E-06	-1	690	622	633
Potri.003	-1.94296	-0.24628	0.000337	0.015298	-1	14	8	20
Potri.016	-0.69125	6.017547	3.20E-09	1.18E-06	-1	1009	1037	1063
Potri.012	-0.75041	7.573216	2.96E-09	1.09E-06	-1	3864	2522	3001
Potri.017	-0.88944	4.024208	2.65E-09	9.89E-07	-1	267	291	256
Potri.001	-2.46939	-0.65892	0.000344	0.015469	-1	7	16	8
Potri.011	-1.04649	1.101579	0.000368	0.016369	-1	38	34	32
Potri.009	-1.03091	2.585886	0.000405	0.017621	-1	66	93	144
Potri.008	-0.9154	4.667412	7.54E-10	3.11E-07	-1	427	435	423
Potri.013	-1.11286	1.819942	0.000417	0.018015	-1	60	60	59
Potri.008	-1.88153	-0.07363	0.000428	0.018438	-1	12	24	12
Potri.004	-1.65583	-0.03688	0.000463	0.019654	-1	20	16	12

Potri.013	-1.19111	0.950472	0.000478	0.02004	-1	42	21	33
Potri.010	-0.97079	4.153341	3.96E-10	1.79E-07	-1	296	324	288
Potri.019	-0.91412	6.396962	3.66E-10	1.67E-07	-1	1889	1124	1317
Potri.013	-0.84192	5.68138	2.82E-10	1.35E-07	-1	989	824	758
Potri.001	-1.75497	1.132156	0.000483	0.020156	-1	31	53	37
Potri.012	-1.00703	2.186514	0.000483	0.020161	-1	56	67	104
Potri.001	-0.85923	4.969299	2.74E-10	1.32E-07	-1	507	493	565
Potri.014	-0.97808	4.548095	1.74E-10	8.70E-08	-1	378	417	403
Potri.T09	-2.16269	-0.65855	0.000496	0.020466	-1	8	12	10
Potri.002	-1.40571	0.45553	0.000515	0.021046	-1	17	25	26
Potri.009	-1.35589	0.576843	0.000524	0.021309	-1	17	29	28
Potri.018	-1.1878	1.200011	0.00053	0.021481	-1	58	29	29
Potri.006	-1.658	0.26481	0.000536	0.021684	-1	11	22	28
Potri.004	-1.80445	-0.11552	0.000541	0.021822	-1	17	14	15
Potri.014	-2.64216	-1.03375	0.000617	0.024241	-1	5	8	9
Potri.010	-1.90286	-0.3863	0.000636	0.02491	-1	12	14	11
Potri.006	-1.01221	1.676076	0.00064	0.02506	-1	61	48	49
Potri.010	-0.81492	5.705719	1.03E-11	6.25E-09	-1	878	858	851
Potri.011	-1.14012	0.880536	0.000649	0.025281	-1	37	21	32
Potri.005	-1.09486	0.954334	0.000658	0.025595	-1	31	28	35
Potri.005	-1.37263	0.384274	0.000688	0.026483	-1	20	26	18
Potri.008	-3.49266	-1.38606	0.000726	0.027654	-1	5	3	8
Potri.010	-1.87425	-0.41097	0.000777	0.029245	-1	8	15	13
Potri.003	-1.45942	0.321644	0.000784	0.029428	-1	25	21	16
Potri.011	-1.21635	0.710051	0.000792	0.029654	-1	25	28	27
Potri.005	-2.64211	-1.03449	0.000835	0.0309	-1	7	10	5
Potri.018	-1.99314	-0.6023	0.000884	0.03228	-1	14	6	11
Potri.008	-1.29373	0.757237	0.000915	0.033175	-1	20	36	28
Potri.010	-1.03155	1.353985	0.000919	0.033221	-1	40	30	55
Potri.005	-1.0059	1.18914	0.00094	0.033687	-1	37	29	44
Potri.010	-1.09825	2.221478	0.001049	0.036462	-1	52	86	99
Potri.003	-1.18805	1.141213	0.001061	0.036783	-1	55	28	28
Potri.006	-1.33382	0.308012	0.001074	0.037077	-1	26	20	14
Potri.019	-1.95865	-0.13475	0.001075	0.037082	-1	9	27	10
Potri.004	-1.1232	0.841036	0.001078	0.03715	-1	36	26	25
Potri.017	-2.94121	-1.20025	0.00111	0.037823	-1	8	8	3
Potri.002	-1.11685	0.796944	0.001112	0.03785	-1	33	19	32
Potri.011	-3.5021	-1.38597	0.001172	0.039407	-1	5	9	2
Potri.005	-1.03446	1.229319	0.001179	0.039588	-1	36	27	51
Potri.001	-1.74266	-0.48962	0.001198	0.039872	-1	10	11	12
Potri.006	-2.12308	-0.68872	0.001236	0.040569	-1	5	10	14
Potri.T17	-5.00992	-1.65814	0.001266	0.041193	-1	2	7	3
Potri.006	-1.20452	0.522737	0.001304	0.042205	-1	20	26	23
Potri.005	-1.32498	0.181878	0.001331	0.042801	-1	16	18	20
Potri.010	-1.39334	0.095728	0.001351	0.043345	-1	15	21	15
Potri.T01	-1.19943	0.52356	0.001402	0.044458	-1	21	26	22
Potri.017	-1.32692	1.336388	0.001427	0.044997	-1	57	28	47
Potri.004	-2.32841	-0.95798	0.001431	0.045063	-1	6	8	9
Potri.001	-1.4308	0.414602	0.001476	0.045971	-1	12	24	30
Potri.006	-1.11863	1.472404	0.001506	0.04657	-1	26	57	55
Potri.011	-1.41834	0.24748	0.001542	0.047275	-1	21	23	14
Potri.005	-2.20926	-0.62875	0.001604	0.048792	-1	9	17	5
Potri.001	-1.26445	1.164938	0.001602	0.048792	-1	38	50	26
Potri.017	-2.06115	-0.72157	0.001653	0.049956	-1	12	12	4

Potri.001	0.452777	3.779103	0.001651	0.049927	1	152	151	145
Potri.007	0.399344	5.045312	0.001631	0.04941	1	357	353	396
Potri.016	0.393239	5.075607	0.001623	0.049239	1	410	391	334
Potri.002	0.331392	8.048187	0.001619	0.049163	1	3439	2761	2979
Potri.005	1.574129	8.183179	9.66E-35	2.07E-30	1	2256	1831	1644
Potri.004	1.885981	4.768346	1.54E-28	1.65E-24	1	142	142	167
Potri.003	0.427972	4.376439	0.001603	0.048792	1	233	223	231
Potri.001	0.897917	1.79113	0.001599	0.048741	1	36	20	35
Potri.T09	0.625151	3.05125	0.001594	0.048663	1	93	67	91
Potri.013	0.531919	4.453812	0.001578	0.048201	1	285	166	249
Potri.014	0.385142	5.569818	0.001549	0.047423	1	597	508	503
Potri.014	0.339452	7.34508	0.001546	0.047352	1	1921	1897	1780
Potri.010	4.591356	1.307321	1.52E-28	1.65E-24	1	1	1	5
Potri.008	0.352955	8.358402	0.001542	0.047275	1	3895	3398	3965
Potri.T15	0.916757	1.222347	0.001542	0.047275	1	14	21	24
Potri.001	0.382987	5.760247	0.001538	0.047254	1	667	618	550
Potri.001	0.444605	4.146866	0.001538	0.047254	1	195	204	182
Potri.012	0.418418	6.140818	0.001521	0.046894	1	834	722	801
Potri.006	0.680397	2.872806	0.001522	0.046894	1	64	91	60
Potri.016	0.375119	5.799928	0.001509	0.046618	1	624	639	624
Potri.019	1.506338	4.324928	2.82E-23	1.73E-19	1	159	124	124
Potri.001	0.962608	0.94099	0.001499	0.046428	1	18	15	14
Potri.003	0.42403	8.086047	0.001489	0.04619	1	2984	3011	3049
Potri.011	0.680005	2.530105	0.001489	0.04619	1	52	49	68
Potri.005	0.435832	5.00973	0.001485	0.046116	1	449	307	316
Potri.001	0.582783	3.504078	0.00148	0.046018	1	124	96	131
Potri.012	1.422251	5.735009	6.67E-21	3.18E-17	1	423	429	278
Potri.002	0.369958	7.472915	0.001472	0.045895	1	1996	1971	2073
Potri.001	0.429698	5.856927	0.001457	0.045431	1	587	629	702
Potri.003	0.435659	4.89606	0.001453	0.045376	1	340	331	313
Potri.009	0.431541	4.841118	0.001449	0.045341	1	326	291	332
Potri.011	0.765219	2.509178	0.001441	0.045255	1	64	64	33
Potri.T04	0.470272	4.867264	0.001439	0.045221	1	354	271	329
Potri.012	0.403044	5.071477	0.001432	0.045063	1	363	392	369
Potri.013	1.309051	4.395954	8.29E-19	2.54E-15	1	170	160	141
Potri.013	0.488695	3.694903	0.001426	0.044997	1	137	145	134
Potri.014	0.880326	1.771705	0.001427	0.044997	1	27	29	34
Potri.010	0.365912	6.617758	0.001422	0.044952	1	1224	1057	1072
Potri.006	0.377501	5.728214	0.001422	0.044952	1	699	564	540
Potri.006	0.48723	4.303926	0.001404	0.044478	1	228	187	224
Potri.006	0.503237	3.521256	0.001399	0.044431	1	116	123	127
Potri.014	1.10746	7.197441	3.09E-18	8.31E-15	1	1346	1275	1016
Potri.009	0.335679	6.649222	0.001393	0.044325	1	1223	1094	1147
Potri.010	0.368176	6.211808	0.001392	0.044325	1	812	856	849
Potri.007	0.383587	6.942497	0.001393	0.044325	1	1487	1392	1288
Potri.019	0.547174	3.267458	0.001391	0.044325	1	101	110	90
Potri.016	1.469188	4.15084	5.99E-18	1.51E-14	1	108	131	126
Potri.008	0.353074	7.892937	0.001375	0.043948	1	2667	2820	2643
Potri.002	0.419856	5.099774	0.001365	0.043646	1	357	393	388
Potri.001	0.840638	2.080979	0.001356	0.043425	1	52	40	23
Potri.006	1.59234	4.418865	1.50E-17	3.40E-14	1	123	134	156
Potri.006	0.395762	5.640654	0.001339	0.043013	1	577	502	600
Potri.011	0.384432	9.410314	0.001329	0.042752	1	8038	7042	7984
Potri.010	0.492235	4.724016	0.001328	0.042746	1	241	304	304

Potri.006	1.208376	6.277807	3.97E-17	7.54E-14	1	694	473	669
Potri.001	0.357723	7.550933	0.001314	0.042423	1	2194	2092	2127
Potri.014	0.454242	4.609343	0.001311	0.04237	1	242	294	261
Potri.013	2.122919	3.332147	4.04E-17	7.54E-14	1	27	51	66
Potri.008	0.330655	8.480291	0.001294	0.041958	1	4608	3792	3985
Potri.003	0.424437	5.331467	0.001283	0.041608	1	506	426	410
Potri.009	1.303156	4.166692	1.88E-16	3.23E-13	1	135	130	137
Potri.014	1.805943	4.036965	2.19E-16	3.62E-13	1	84	108	90
Potri.003	0.493314	4.463943	0.001267	0.041193	1	213	252	244
Potri.016	0.381638	7.715296	0.001263	0.041153	1	2349	2569	2189
Potri.017	2.414556	2.119063	4.44E-16	6.82E-13	1	19	16	17
Potri.001	0.365672	6.23052	0.00126	0.041112	1	995	749	825
Potri.014	1.348714	3.885336	5.01E-16	7.42E-13	1	95	122	105
Potri.001	0.478603	4.295262	0.00125	0.040875	1	239	191	208
Potri.015	0.397551	5.119405	0.001244	0.040732	1	437	367	366
Potri.018	0.532966	4.201472	0.001241	0.040668	1	182	231	168
Potri.001	0.381551	5.999307	0.001238	0.040606	1	700	721	740
Potri.013	2.700747	1.293088	9.90E-16	1.37E-12	1	6	11	7
Potri.005	0.361505	7.251505	0.001236	0.040569	1	1926	1649	1641
Potri.001	0.388254	6.603889	0.001236	0.040569	1	1163	1162	961
Potri.002	0.405572	6.22026	0.001225	0.040361	1	856	895	747
Potri.015	0.785063	2.410629	0.00122	0.040267	1	54	44	51
Potri.011	0.475018	4.587244	0.001218	0.040242	1	252	257	271
Potri.005	0.945694	1.556319	0.001218	0.040242	1	31	20	24
Potri.017	0.631297	3.020163	0.001215	0.040201	1	94	71	80
Potri.002	0.754938	2.126267	0.001206	0.039998	1	43	34	46
Potri.019	0.967293	2.176073	0.001203	0.03995	1	29	44	42
Potri.019	0.545945	5.258999	0.001201	0.03994	1	475	343	399
Potri.004	1.390594	3.978307	6.19E-15	8.06E-12	1	101	95	141
Potri.001	1.401929	4.1197	7.88E-15	9.96E-12	1	147	102	123
Potri.010	0.488105	3.947327	0.001198	0.039872	1	184	158	156
Potri.003	0.359125	8.237402	0.001191	0.0398	1	3333	3500	3464
Potri.017	0.680839	3.950798	0.001181	0.039608	1	198	123	142
Potri.T10	1.031965	7.174474	1.38E-14	1.64E-11	1	1367	1346	994
Potri.006	1.860249	2.627492	1.48E-14	1.72E-11	1	29	41	32
Potri.015	0.360591	9.28061	0.001144	0.038692	1	7770	6477	7063
Potri.018	0.358558	6.213251	0.001132	0.038344	1	881	782	873
Potri.001	0.432314	7.627102	0.001123	0.038114	1	2284	1828	2464
Potri.013	0.434665	4.420175	0.001118	0.037942	1	271	200	238
Potri.T12	1.119137	4.327276	1.99E-14	2.19E-11	1	199	133	162
Potri.003	0.714954	2.180504	0.001112	0.03785	1	48	49	33
Potri.001	0.357534	7.456117	0.001108	0.037788	1	1982	1993	2023
Potri.010	0.482057	3.648668	0.001099	0.037536	1	151	131	123
Potri.010	0.816288	2.443342	0.001096	0.037523	1	65	28	58
Potri.006	0.398711	8.035484	0.001092	0.037453	1	3401	2368	3108
Potri.018	0.483892	5.718975	0.001088	0.03734	1	553	603	551
Potri.009	1.589317	4.583169	1.62E-13	1.54E-10	1	183	170	114
Potri.017	0.38167	7.098138	0.001073	0.03707	1	1473	1509	1649
Potri.011	0.518838	3.636046	0.001071	0.037034	1	141	138	116
Potri.004	0.365313	5.650322	0.001065	0.036906	1	601	536	574
Potri.002	0.491959	4.185942	0.001053	0.036534	1	181	195	209
Potri.012	0.353265	7.081593	0.001038	0.036167	1	1568	1521	1548
Potri.014	0.485251	3.832298	0.001024	0.035758	1	155	154	150
Potri.010	0.540252	3.990556	0.001018	0.035607	1	182	176	144

Potri.006	0.380875	6.956897	0.001003	0.035217	1	1423	1256	1531
Potri.009	0.392964	5.394928	0.001002	0.035217	1	479	486	450
Potri.011	0.507296	4.393614	0.001002	0.035217	1	216	200	257
Potri.011	1.072531	4.928778	5.64E-13	4.95E-10	1	314	228	226
Potri.010	0.734503	2.113047	0.000988	0.035013	1	47	31	45
Potri.002	0.369786	6.431849	0.000979	0.034755	1	998	942	996
Potri.014	0.716847	2.594026	0.000968	0.034471	1	70	46	59
Potri.010	0.422723	5.086451	0.000961	0.034295	1	400	327	404
Potri.013	0.407173	5.808473	0.000956	0.034155	1	602	640	631
Potri.T15	0.96347	1.289277	0.000949	0.033952	1	26	16	19
Potri.006	1.083573	7.480539	6.95E-13	5.86E-10	1	1782	1226	1487
Potri.004	0.466315	6.565357	0.000939	0.033674	1	953	1161	974
Potri.001	0.48874	5.424982	0.000934	0.033551	1	402	539	443
Potri.011	0.378828	6.118421	0.000925	0.03329	1	777	784	790
Potri.013	0.595917	3.251936	0.000925	0.03329	1	100	96	96
Potri.009	0.345689	8.068403	0.000923	0.033266	1	3126	2961	3136
Potri.014	0.431059	5.093975	0.000923	0.033266	1	443	346	347
Potri.T09	0.590084	2.987081	0.000923	0.033266	1	86	85	72
Potri.001	1.647527	2.300543	6.86E-13	5.86E-10	1	37	26	28
Potri.016	0.419679	6.878467	0.000917	0.033207	1	1185	1406	1318
Potri.003	1.116424	5.097493	1.04E-12	8.44E-10	1	248	287	303
Potri.007	0.667056	2.626518	0.000905	0.032817	1	75	48	60
Potri.011	0.553241	4.925446	0.000904	0.032814	1	293	259	404
Potri.008	0.36979	8.408872	0.00089	0.032472	1	3714	3784	4052
Potri.010	2.173831	1.657517	1.75E-12	1.34E-09	1	18	13	12
Potri.011	0.483562	4.726721	0.00088	0.032154	1	334	269	258
Potri.010	0.521613	3.874811	0.000878	0.032106	1	187	144	137
Potri.001	0.70638	2.368082	0.000874	0.032016	1	50	58	41
Potri.002	0.866997	1.455615	0.000874	0.032016	1	28	24	20
Potri.005	0.42525	5.70001	0.000871	0.031949	1	612	604	513
Potri.008	0.590781	2.930927	0.000864	0.031761	1	72	80	81
Potri.010	0.878702	1.461434	0.000863	0.031717	1	30	22	20
Potri.011	0.498864	3.630816	0.000855	0.031458	1	145	135	117
Potri.018	0.403673	6.674691	0.000839	0.03095	1	1138	1134	1154
Potri.005	0.538155	4.233637	0.000838	0.03095	1	178	201	214
Potri.003	0.364787	8.639643	0.000836	0.030901	1	4491	4684	4411
Potri.013	0.474356	6.529862	0.000833	0.03086	1	955	1067	985
Potri.013	0.448935	5.00264	0.000826	0.030602	1	390	359	307
Potri.005	0.437787	5.369126	0.000817	0.030339	1	439	448	477
Potri.010	0.417691	8.739681	0.000814	0.030268	1	5309	4740	4270
Potri.002	0.702798	2.297421	0.000814	0.030268	1	50	51	41
Potri.001	1.053435	5.345599	2.75E-12	1.99E-09	1	349	267	415
Potri.008	0.353775	7.431759	0.000796	0.029747	1	1985	1968	1956
Potri.007	0.376592	6.647199	0.000795	0.029742	1	1318	1040	1057
Potri.011	1.699005	3.142872	2.85E-12	2.01E-09	1	32	69	58
Potri.001	0.432116	5.133544	0.000788	0.029531	1	360	410	389
Potri.004	1.523209	2.594316	3.17E-12	2.16E-09	1	36	49	34
Potri.011	0.668442	2.7793	0.000778	0.029245	1	68	58	77
Potri.006	0.350799	8.837468	0.000775	0.029208	1	5253	5191	5236
Potri.012	0.432621	7.467332	0.000768	0.028993	1	1911	2178	1772
Potri.008	0.472392	4.749487	0.000767	0.028993	1	289	325	260
Potri.002	0.358724	8.170725	0.000757	0.028703	1	3346	3153	3353
Potri.016	0.388414	5.886369	0.000731	0.027803	1	691	627	679
Potri.006	0.437533	6.090905	0.000729	0.027736	1	925	638	708

Potri.018	0.632036	2.687348	0.000712	0.02722	1	64	65	64
Potri.012	0.397492	5.886351	0.000706	0.027064	1	707	599	686
Potri.006	0.424604	4.918064	0.000703	0.026971	1	348	317	339
Potri.016	0.426749	5.170773	0.000694	0.026649	1	413	381	402
Potri.001	0.55523	7.331455	0.000676	0.026114	1	1306	1965	1772
Potri.014	0.449733	4.713861	0.000672	0.026002	1	307	263	293
Potri.007	0.629007	5.219232	0.000671	0.025981	1	361	354	421
Potri.013	0.963724	1.218732	0.000665	0.025792	1	27	13	18
Potri.003	0.551493	3.322974	0.000661	0.025659	1	111	96	106
Potri.001	0.47671	7.254934	0.000656	0.025513	1	1883	1392	1728
Potri.004	0.436052	5.917241	0.000652	0.025396	1	699	708	594
Potri.008	0.477795	4.478563	0.000647	0.025255	1	264	207	254
Potri.009	0.661557	5.367752	0.000641	0.025061	1	313	505	416
Potri.001	1.644531	3.056134	1.09E-11	6.52E-09	1	33	57	64
Potri.012	3.873874	0.010597	1.29E-11	7.61E-09	1	0	2	2
Potri.015	0.464953	4.5529	0.000633	0.024818	1	287	221	260
Potri.001	0.475133	4.242958	0.000612	0.024032	1	204	199	211
Potri.008	0.576804	3.881871	0.000605	0.023827	1	162	162	134
Potri.011	0.37224	6.648399	0.0006	0.02364	1	1150	1104	1154
Potri.015	0.437549	5.173999	0.000594	0.023452	1	389	421	381
Potri.013	0.463193	4.669728	0.000592	0.023433	1	334	257	244
Potri.005	0.440408	9.598244	0.000583	0.023147	1	8647	8476	8532
Potri.003	1.682148	2.579024	2.09E-11	1.20E-08	1	38	34	37
Potri.004	0.392011	6.149288	0.000576	0.022931	1	795	752	844
Potri.017	0.377522	6.204594	0.000557	0.022264	1	853	814	833
Potri.007	0.569052	4.933789	0.000549	0.022035	1	253	376	321
Potri.014	0.715266	2.353442	0.00055	0.022035	1	51	50	46
Potri.003	0.717787	2.553515	0.000549	0.022035	1	70	43	57
Potri.004	1.368168	2.447354	2.84E-11	1.61E-08	1	34	44	38
Potri.013	0.509432	3.884336	0.000544	0.021896	1	153	158	160
Potri.009	0.529299	4.288445	0.000544	0.021896	1	247	191	185
Potri.013	0.53875	3.737291	0.000538	0.021747	1	139	145	136
Potri.018	0.458864	4.789359	0.000536	0.021684	1	285	311	307
Potri.004	1.358355	2.936321	4.91E-11	2.67E-08	1	68	40	58
Potri.010	0.37962	6.58094	0.000522	0.021288	1	1162	1030	1057
Potri.001	0.459215	5.264679	0.00052	0.021242	1	417	443	398
Potri.002	2.113311	1.410955	8.62E-11	4.46E-08	1	14	19	4
Potri.016	0.521063	6.867225	0.000513	0.020995	1	1463	1042	1254
Potri.009	0.58276	5.260216	0.000511	0.020931	1	333	495	358
Potri.003	0.513105	5.876288	0.000499	0.020554	1	548	730	597
Potri.005	0.488963	5.057097	0.000497	0.020517	1	400	339	341
Potri.002	2.050728	2.754744	1.41E-10	7.12E-08	1	48	13	41
Potri.001	0.371656	6.759631	0.000495	0.020448	1	1267	1210	1207
Potri.003	0.478484	4.570491	0.000491	0.020353	1	253	266	251
Potri.T12	0.884945	1.744171	0.000488	0.020278	1	29	27	32
Potri.006	0.409493	8.166672	0.000486	0.020243	1	3199	2888	3542
Potri.005	0.415951	6.205676	0.000485	0.02021	1	780	819	860
Potri.004	1.144507	3.794704	2.88E-10	1.35E-07	1	114	96	125
Potri.004	1.326267	2.416477	2.86E-10	1.35E-07	1	39	44	33
Potri.003	0.445044	4.961539	0.000482	0.020138	1	385	304	340
Potri.001	0.417121	6.401422	0.00048	0.020079	1	895	925	996
Potri.009	1.479104	2.89806	4.05E-10	1.81E-07	1	50	47	54
Potri.010	0.415332	5.662371	0.000473	0.019909	1	650	495	552
Potri.008	0.586247	6.374797	0.000472	0.019883	1	867	683	1039

Potri.006	0.377252	7.827916	0.000469	0.019828	1	2925	2270	2548
Potri.016	0.577558	4.166571	0.000466	0.01973	1	158	206	192
Potri.008	1.953578	1.217331	4.76E-10	2.09E-07	1	11	15	9
Potri.004	0.372054	7.140819	0.000456	0.019428	1	1650	1566	1583
Potri.009	0.426847	7.105002	0.000452	0.019326	1	1729	1340	1529
Potri.009	0.492277	4.110327	0.000451	0.019314	1	215	162	181
Potri.014	0.523777	5.928301	0.000448	0.019217	1	659	577	711
Potri.018	0.835255	6.688023	0.000434	0.018645	1	615	1320	912
Potri.001	0.409783	6.311947	0.000427	0.018432	1	854	915	885
Potri.006	0.482449	5.81842	0.000412	0.01785	1	570	612	647
Potri.015	1.184355	4.008206	9.63E-10	3.90E-07	1	128	153	100
Potri.008	0.466466	5.250619	0.00041	0.017803	1	387	453	400
Potri.018	0.545604	3.632107	0.000404	0.017592	1	158	118	115
Potri.011	0.531257	4.058126	0.000393	0.017208	1	189	193	146
Potri.010	0.410252	5.431301	0.000392	0.017179	1	496	449	498
Potri.004	0.469699	5.133715	0.000387	0.017048	1	349	376	417
Potri.014	0.498645	3.892198	0.000381	0.016825	1	175	153	149
Potri.014	0.506833	4.197044	0.000378	0.016687	1	208	184	196
Potri.006	0.573246	3.299414	0.000373	0.016533	1	105	92	108
Potri.002	0.4515	7.192395	0.000369	0.016377	1	1623	1654	1538
Potri.007	0.825414	2.160488	0.000365	0.01624	1	38	35	49
Potri.001	0.614754	3.237927	0.000362	0.016153	1	117	92	79
Potri.010	0.747285	2.524039	0.000362	0.016153	1	62	49	53
Potri.011	0.368054	8.166746	0.00036	0.016077	1	3488	3073	3244
Potri.003	0.854616	2.960937	0.000352	0.01575	1	65	80	67
Potri.008	0.718475	2.557839	0.000348	0.015598	1	61	48	61
Potri.013	0.396749	6.288164	0.000347	0.015566	1	911	885	834
Potri.016	0.757453	2.185391	0.000345	0.015517	1	42	38	48
Potri.015	0.679357	3.03226	0.000342	0.015414	1	89	71	82
Potri.004	0.413506	5.563949	0.000341	0.015381	1	558	518	505
Potri.010	0.417242	5.986752	0.00034	0.015371	1	713	737	664
Potri.019	0.491772	4.726769	0.00034	0.015371	1	320	283	254
Potri.008	0.466073	8.253555	0.000338	0.01532	1	3266	3581	3133
Potri.003	0.436218	5.77246	0.000337	0.015298	1	629	561	621
Potri.008	0.53808	3.95444	0.000337	0.015298	1	183	130	178
Potri.005	0.538842	5.438231	0.000334	0.015192	1	406	494	470
Potri.014	1.215104	4.779126	4.30E-09	1.47E-06	1	155	202	281
Potri.004	0.77229	2.222986	0.000331	0.015113	1	51	38	42
Potri.008	0.576597	3.757658	0.000325	0.014903	1	162	138	121
Potri.011	0.612897	4.215865	0.000324	0.014879	1	159	204	204
Potri.013	1.17666	2.483799	5.41E-09	1.80E-06	1	47	49	35
Potri.012	0.403907	5.983261	0.000321	0.014773	1	802	645	682
Potri.014	0.512021	4.136093	0.000321	0.014773	1	185	200	176
Potri.014	1.059434	3.227658	6.44E-09	2.06E-06	1	96	75	64
Potri.005	0.683452	2.979434	0.000315	0.014595	1	89	70	74
Potri.001	0.732351	2.73283	0.000315	0.014589	1	64	63	64
Potri.003	0.441681	6.066027	0.000314	0.014582	1	683	764	760
Potri.002	0.399319	6.036152	0.000313	0.014523	1	779	680	749
Potri.017	0.505384	4.041594	0.00031	0.01439	1	207	166	156
Potri.010	0.653894	2.826793	0.000309	0.01439	1	87	60	65
Potri.015	0.489853	9.550862	0.000306	0.014268	1	7545	7865	8873
Potri.004	0.421877	6.73898	0.000302	0.01411	1	1230	1121	1211
Potri.008	0.364899	9.41889	0.0003	0.014021	1	8014	7499	7846
Potri.008	0.507127	4.40572	0.000299	0.014006	1	250	200	231

Potri.005	0.92963	3.913721	0.000296	0.013889	1	87	192	118
Potri.010	0.427954	8.698427	0.000286	0.013505	1	4968	4276	4607
Potri.014	0.568116	4.347507	0.000284	0.013415	1	193	242	199
Potri.005	0.932895	1.845866	0.000282	0.013389	1	35	27	31
Potri.014	0.437847	5.973639	0.000279	0.013275	1	685	702	690
Potri.018	0.550226	4.240019	0.000273	0.013055	1	185	220	188
Potri.009	0.512772	5.209402	0.000272	0.013032	1	404	446	335
Potri.019	0.8566	2.524467	0.00027	0.012958	1	71	48	38
Potri.002	0.623793	4.087572	0.000268	0.01288	1	219	144	159
Potri.002	0.437132	5.682819	0.000267	0.012871	1	577	564	558
Potri.010	0.599384	3.015242	0.000268	0.012871	1	86	76	85
Potri.002	0.74723	2.351124	0.000265	0.012774	1	53	39	53
Potri.003	0.562497	4.750973	0.000261	0.01264	1	295	248	303
Potri.005	0.411214	6.376942	0.00026	0.012614	1	1077	870	845
Potri.001	0.476094	4.644343	0.000258	0.012517	1	310	246	259
Potri.011	0.544633	4.000386	0.000254	0.012336	1	157	178	168
Potri.006	0.538173	3.944156	0.000245	0.011987	1	153	172	160
Potri.005	0.50152	5.493455	0.000243	0.011897	1	515	495	443
Potri.018	1.299021	3.988258	1.81E-08	5.07E-06	1	118	109	128
Potri.014	0.537689	5.836482	0.000241	0.011806	1	629	521	668
Potri.005	0.392352	7.730171	0.000236	0.011654	1	2525	2312	2334
Potri.017	1.170851	2.481422	2.48E-08	6.69E-06	1	40	36	55
Potri.017	0.598333	4.237994	0.000225	0.011219	1	160	215	204
Potri.010	0.417902	6.592317	0.000222	0.011136	1	1043	1087	1085
Potri.010	0.401511	7.104893	0.00022	0.011063	1	1560	1483	1582
Potri.014	0.499522	6.38417	0.00022	0.011063	1	892	1020	780
Potri.015	0.584047	4.844092	0.00022	0.011063	1	273	347	270
Potri.004	0.548275	4.245746	0.000213	0.010825	1	189	225	182
Potri.001	0.646784	4.457837	0.000211	0.010722	1	188	275	198
Potri.001	0.447768	5.041034	0.000209	0.010648	1	370	364	349
Potri.001	0.654147	3.362225	0.000204	0.010422	1	92	121	94
Potri.015	0.412207	8.55983	0.000201	0.010267	1	4459	4215	3972
Potri.004	0.819551	2.045405	0.0002	0.010244	1	47	34	32
Potri.013	1.429892	3.453524	3.40E-08	8.54E-06	1	55	115	57
Potri.013	0.504702	5.050641	0.000194	0.009981	1	343	357	364
Potri.009	0.596565	3.731915	0.000193	0.009918	1	126	143	139
Potri.008	0.46604	7.40018	0.000191	0.009847	1	1747	1860	1913
Potri.003	0.624865	4.902444	0.000191	0.009847	1	310	338	266
Potri.002	1.866223	0.806414	3.77E-08	9.42E-06	1	13	8	6
Potri.004	0.854937	1.844672	0.000174	0.009137	1	33	31	32
Potri.010	0.996241	2.268434	0.000172	0.009027	1	38	52	32
Potri.001	1.807077	2.044657	4.50E-08	1.08E-05	1	18	33	18
Potri.013	0.596446	3.649077	0.000164	0.008659	1	136	138	112
Potri.003	0.673304	6.57688	0.000161	0.008531	1	1173	760	956
Potri.001	0.464471	6.936235	0.000161	0.008524	1	1391	1359	1264
Potri.008	0.587305	4.15357	0.000159	0.008456	1	209	164	180
Potri.005	0.487298	5.375106	0.000158	0.008439	1	465	457	423
Potri.002	0.44091	10.73989	0.000157	0.008405	1	20123	17258	19345
Potri.008	0.521554	5.574727	0.000157	0.008405	1	573	405	552
Potri.001	0.563243	9.416116	0.000157	0.008405	1	6668	8151	6615
Potri.014	0.781881	6.229861	0.000157	0.008405	1	510	893	725
Potri.003	0.566342	5.053538	0.000156	0.008383	1	343	307	391
Potri.011	0.431127	8.419147	0.00015	0.008156	1	3948	3754	3678
Potri.014	0.786005	2.384515	0.00015	0.008156	1	52	37	57

Potri.016	1.332625	2.229572	6.78E-08	1.54E-05	1	47	22	33
Potri.003	0.727536	4.582709	0.000147	0.008053	1	324	156	229
Potri.002	1.380237	2.168092	6.87E-08	1.55E-05	1	39	32	24
Potri.006	0.911964	2.512193	0.000142	0.00784	1	51	37	63
Potri.018	0.531021	3.866443	0.00014	0.007747	1	162	145	155
Potri.010	0.444224	5.628315	0.000135	0.007472	1	613	537	486
Potri.001	0.523858	4.027918	0.000134	0.007472	1	187	168	164
Potri.004	2.097763	0.762528	8.45E-08	1.84E-05	1	12	7	4
Potri.010	0.642759	4.283066	0.00013	0.007316	1	228	220	143
Potri.001	1.566473	1.362085	1.22E-07	2.55E-05	1	18	9	21
Potri.006	0.497026	5.916648	0.000128	0.007229	1	604	717	624
Potri.009	0.663261	3.844056	0.000128	0.007221	1	136	172	121
Potri.005	0.423756	5.87862	0.000127	0.00719	1	701	599	661
Potri.019	0.489437	5.909619	0.000126	0.007161	1	638	684	623
Potri.012	1.829029	0.831006	1.28E-07	2.64E-05	1	8	11	9
Potri.001	0.545504	6.194318	0.000123	0.006997	1	704	857	750
Potri.009	0.590242	4.90983	0.00012	0.006887	1	329	244	362
Potri.008	0.468141	6.239946	0.000119	0.006804	1	845	840	786
Potri.011	0.421061	6.637059	0.000117	0.006721	1	1155	1118	1046
Potri.003	0.505143	4.961525	0.000116	0.006644	1	339	343	319
Potri.015	0.601946	5.306915	0.000114	0.006569	1	457	296	476
Potri.001	0.814917	4.48763	0.000113	0.006532	1	280	156	201
Potri.004	1.555424	1.517432	1.55E-07	3.14E-05	1	19	19	16
Potri.005	0.442141	6.097579	0.000112	0.006499	1	858	668	745
Potri.010	0.686777	4.312175	0.000111	0.006459	1	251	142	202
Potri.019	0.421916	6.751264	0.00011	0.006406	1	1321	1104	1175
Potri.006	0.411116	9.127985	0.000108	0.006349	1	6635	5975	6158
Potri.015	2.55174	0.285522	1.76E-07	3.43E-05	1	3	6	3
Potri.002	1.665896	1.222537	1.95E-07	3.73E-05	1	15	20	6
Potri.009	0.562323	5.489876	0.000105	0.00622	1	496	402	517
Potri.015	0.80007	2.355327	0.000105	0.006209	1	49	41	52
Potri.001	0.431516	6.237034	0.000103	0.006159	1	893	823	790
Potri.002	0.534797	4.163427	0.000103	0.006159	1	204	181	183
Potri.009	0.677233	3.611712	0.000103	0.006159	1	134	127	103
Potri.006	0.764131	3.300169	0.000104	0.006159	1	108	84	90
Potri.007	0.622637	3.571014	0.000103	0.006147	1	149	110	104
Potri.003	0.482309	5.807377	0.000102	0.006109	1	739	516	576
Potri.014	0.474778	5.053428	9.94E-05	0.005981	1	390	369	323
Potri.014	0.572474	4.379669	9.93E-05	0.005981	1	268	195	190
Potri.018	0.491285	6.544314	9.75E-05	0.005879	1	1017	1052	953
Potri.011	0.49975	6.193739	9.75E-05	0.005879	1	872	801	695
Potri.009	0.428868	7.699385	9.61E-05	0.005819	1	2388	2304	2221
Potri.013	0.463704	6.293571	9.55E-05	0.005807	1	823	908	833
Potri.009	0.709677	4.219195	9.52E-05	0.005802	1	177	149	222
Potri.001	0.490762	5.307058	9.45E-05	0.005763	1	462	417	404
Potri.007	0.579898	4.28217	9.36E-05	0.005716	1	214	169	223
Potri.015	0.526182	5.83415	8.95E-05	0.005515	1	604	638	577
Potri.009	0.496155	5.508836	8.83E-05	0.00545	1	465	523	479
Potri.004	1.068642	3.34319	3.50E-07	6.21E-05	1	74	80	98
Potri.010	0.596111	3.880543	8.67E-05	0.005397	1	166	122	167
Potri.014	1.224587	3.60906	3.58E-07	6.33E-05	1	58	102	120
Potri.006	0.725828	3.509005	8.48E-05	0.005309	1	100	137	93
Potri.012	0.621414	3.416599	8.43E-05	0.005288	1	117	92	116
Potri.003	0.585213	5.299015	8.41E-05	0.005283	1	383	487	351

Potri.013	0.503894	6.285602	8.26E-05	0.005235	1	771	886	849
Potri.001	0.481387	5.070261	8.20E-05	0.00521	1	383	375	333
Potri.016	0.545251	5.539262	8.19E-05	0.00521	1	564	443	471
Potri.007	0.570421	3.959921	8.18E-05	0.00521	1	179	143	164
Potri.009	0.532368	4.381339	7.94E-05	0.005072	1	236	203	223
Potri.013	0.453424	7.431134	7.69E-05	0.004947	1	2138	1788	1775
Potri.014	0.881839	2.083408	7.69E-05	0.004947	1	47	35	31
Potri.001	1.970611	0.67842	4.14E-07	7.00E-05	1	9	6	8
Potri.010	0.46297	6.301856	7.59E-05	0.004905	1	896	872	818
Potri.T07	0.766202	2.876006	7.49E-05	0.004858	1	82	63	64
Potri.012	0.513016	5.929205	7.38E-05	0.004811	1	586	663	700
Potri.012	0.574282	5.438353	7.31E-05	0.004783	1	446	494	414
Potri.005	0.482065	9.350557	7.23E-05	0.004747	1	6821	7453	6934
Potri.015	0.857361	2.654572	7.23E-05	0.004747	1	75	53	44
Potri.017	0.490713	4.650171	7.18E-05	0.004733	1	303	253	257
Potri.016	0.581455	3.803649	7.19E-05	0.004733	1	165	126	143
Potri.006	0.513294	6.939333	7.06E-05	0.004672	1	1485	1167	1306
Potri.013	1.281626	2.261207	6.88E-07	0.000108	1	28	29	49
Potri.006	0.71698	3.098252	6.83E-05	0.004551	1	82	78	89
Potri.004	0.441284	8.158738	6.72E-05	0.004497	1	3317	2937	3215
Potri.008	1.024784	2.678576	7.10E-07	0.00011	1	48	64	49
Potri.009	0.578482	3.893613	6.69E-05	0.004491	1	179	132	152
Potri.002	0.435473	7.086488	6.64E-05	0.004469	1	1682	1317	1523
Potri.010	0.625783	4.396234	6.42E-05	0.004349	1	189	244	207
Potri.006	0.584004	4.338763	6.11E-05	0.004169	1	201	227	199
Potri.009	0.885876	3.92364	6.04E-05	0.004138	1	108	162	140
Potri.010	0.482504	5.915652	5.87E-05	0.004037	1	745	545	680
Potri.T09	0.974996	2.510547	5.82E-05	0.004016	1	52	44	51
Potri.018	0.504742	5.283986	5.65E-05	0.003915	1	418	447	387
Potri.001	0.529609	6.166571	5.52E-05	0.003831	1	759	803	726
Potri.003	0.788922	7.346334	5.46E-05	0.003794	1	1152	1962	1491
Potri.004	0.99788	4.709529	5.35E-05	0.003722	1	296	172	216
Potri.006	1.605001	1.794669	1.02E-06	0.000149	1	15	33	16
Potri.005	1.337761	2.493063	1.07E-06	0.000156	1	41	36	45
Potri.014	0.578346	4.117608	5.08E-05	0.003597	1	189	176	175
Potri.006	0.619749	4.253755	5.08E-05	0.003597	1	193	185	205
Potri.018	0.642177	4.363848	5.08E-05	0.003597	1	203	220	200
Potri.006	1.593324	2.979732	1.19E-06	0.000169	1	57	36	58
Potri.011	0.842869	3.023052	4.88E-05	0.003482	1	66	76	81
Potri.T02	1.070686	2.31681	1.31E-06	0.000181	1	39	51	32
Potri.005	0.562517	4.934894	4.78E-05	0.003438	1	306	349	303
Potri.012	2.968527	-0.43186	1.32E-06	0.000182	1	3	1	1
Potri.006	0.808398	2.574704	4.67E-05	0.003371	1	71	49	46
Potri.018	1.76164	0.582888	1.35E-06	0.000185	1	9	10	5
Potri.019	1.663274	0.849964	1.38E-06	0.000188	1	10	15	6
Potri.014	0.4318	6.962008	4.23E-05	0.003097	1	1556	1287	1310
Potri.003	0.711347	4.369904	4.09E-05	0.003012	1	251	167	194
Potri.014	0.709614	3.013388	4.07E-05	0.003004	1	74	84	77
Potri.008	0.560288	5.68733	4.00E-05	0.002969	1	495	587	534
Potri.013	0.863441	2.759221	3.96E-05	0.002946	1	46	68	69
Potri.013	0.595559	4.670609	3.89E-05	0.00291	1	252	301	233
Potri.005	0.653488	3.453069	3.90E-05	0.00291	1	106	117	105
Potri.006	0.672234	3.497479	3.77E-05	0.002824	1	109	100	127
Potri.008	0.418131	8.870749	3.72E-05	0.002796	1	5595	4950	5120

Potri.014	0.754381	3.203291	3.53E-05	0.002659	1	89	75	100
Potri.012	0.510321	6.134953	3.50E-05	0.002643	1	714	759	781
Potri.001	0.5732	5.439324	3.48E-05	0.002637	1	501	483	376
Potri.007	0.619756	7.082529	3.40E-05	0.002592	1	1211	1635	1297
Potri.009	0.482667	6.966715	3.37E-05	0.00258	1	1275	1394	1389
Potri.006	0.557292	5.017406	3.35E-05	0.00257	1	326	365	326
Potri.T00	0.644822	3.889743	3.34E-05	0.002569	1	157	139	152
Potri.007	0.470095	7.148335	3.32E-05	0.002558	1	1542	1448	1647
Potri.001	0.478013	8.48924	3.24E-05	0.002515	1	3900	4064	3741
Potri.005	0.539118	5.122583	3.11E-05	0.00244	1	446	308	357
Potri.001	0.719771	3.519537	2.98E-05	0.002349	1	135	91	110
Potri.014	0.98539	2.285671	2.94E-05	0.002319	1	40	49	35
Potri.006	0.497241	5.719551	2.85E-05	0.002266	1	587	575	540
Potri.004	0.542924	4.659701	2.81E-05	0.002237	1	283	258	259
Potri.001	0.800196	4.21518	2.80E-05	0.002236	1	202	129	197
Potri.001	0.536277	6.907206	2.75E-05	0.002196	1	1325	1081	1420
Potri.016	0.561831	5.241539	2.69E-05	0.002163	1	358	406	421
Potri.005	0.855954	3.450504	2.62E-05	0.002115	1	82	115	102
Potri.006	0.500677	5.221102	2.58E-05	0.002087	1	432	365	407
Potri.007	0.635169	5.220669	2.56E-05	0.002072	1	323	447	360
Potri.013	0.463471	7.41792	2.55E-05	0.002068	1	1900	1900	1805
Potri.014	1.56496	0.932387	3.49E-06	0.000408	1	18	8	9
Potri.014	0.516654	7.285768	2.52E-05	0.002045	1	1952	1450	1631
Potri.003	1.065518	1.87097	3.78E-06	0.000433	1	32	25	32
Potri.018	0.479511	7.142851	2.46E-05	0.002024	1	1569	1445	1591
Potri.002	0.542527	5.338238	2.34E-05	0.001942	1	404	434	441
Potri.016	0.648359	4.641332	2.33E-05	0.001938	1	259	269	227
Potri.012	0.604393	5.414095	2.32E-05	0.001936	1	400	490	422
Potri.002	0.854925	2.880068	2.23E-05	0.001874	1	68	62	71
Potri.009	0.958524	2.094333	2.17E-05	0.001838	1	44	33	33
Potri.008	0.563573	6.869962	2.12E-05	0.001794	1	1141	1351	1176
Potri.001	0.507613	5.569049	2.06E-05	0.001756	1	542	501	485
Potri.007	0.636551	4.559169	2.07E-05	0.001756	1	209	250	255
Potri.001	0.889653	2.992651	1.94E-05	0.001652	1	70	73	71
Potri.009	0.50014	5.243756	1.89E-05	0.001624	1	426	396	400
Potri.002	0.629985	6.569268	1.87E-05	0.001611	1	1035	901	975
Potri.003	0.455269	9.420116	1.81E-05	0.00157	1	7454	7603	7467
Potri.012	0.50745	6.528601	1.74E-05	0.001518	1	1055	923	999
Potri.012	0.567155	5.827308	1.73E-05	0.001509	1	636	615	533
Potri.012	0.513748	5.334047	1.70E-05	0.001488	1	470	395	431
Potri.013	0.534304	5.201606	1.69E-05	0.001485	1	437	348	388
Potri.T01	0.682868	5.49821	1.67E-05	0.00147	1	530	406	421
Potri.006	0.668007	6.379124	1.63E-05	0.001437	1	857	983	663
Potri.007	0.823691	4.417879	1.59E-05	0.001411	1	190	259	148
Potri.001	1.028422	6.339934	6.91E-06	0.000705	1	515	961	579
Potri.014	0.560526	5.004431	1.48E-05	0.001317	1	343	310	356
Potri.003	1.003412	2.225195	7.34E-06	0.000748	1	37	36	45
Potri.001	1.163527	2.061858	8.02E-06	0.000809	1	26	32	39
Potri.001	0.546731	5.224015	1.39E-05	0.001249	1	409	401	372
Potri.014	0.669176	4.550684	1.38E-05	0.001247	1	266	217	222
Potri.012	0.734508	5.93996	1.29E-05	0.001178	1	498	742	543
Potri.003	0.602275	5.094114	1.19E-05	0.001104	1	374	361	321
Potri.015	0.505534	6.51433	1.07E-05	0.001012	1	970	1032	939
Potri.008	0.546186	7.085225	1.04E-05	0.000984	1	1612	1289	1419

Potri.017	1.18499	3.018621	1.04E-05	0.000984	1	82	37	73
Potri.018	0.582757	6.50895	1.01E-05	0.000962	1	883	1084	865
Potri.004	0.827749	3.893578	9.87E-06	0.000946	1	104	151	157
Potri.001	0.903522	3.415256	9.82E-06	0.000946	1	90	117	79
Potri.010	0.613786	6.953209	9.50E-06	0.00092	1	1225	1498	1084
Potri.014	0.542691	6.903925	8.84E-06	0.000873	1	1211	1385	1195
Potri.004	0.591571	5.04312	8.71E-06	0.000862	1	394	303	330
Potri.002	0.864	7.432394	8.69E-06	0.000862	1	1153	1972	1602
Potri.004	0.60662	4.807921	8.35E-06	0.000836	1	290	274	299
Potri.013	0.798832	5.157561	8.37E-06	0.000836	1	299	391	319
Potri.017	0.55476	5.181967	8.04E-06	0.000809	1	364	381	397
Potri.017	0.616913	4.967373	7.89E-06	0.000799	1	290	342	325
Potri.011	0.797354	2.940422	7.42E-06	0.000753	1	70	79	66
Potri.004	1.692012	0.632118	1.47E-05	0.001317	1	14	10	2
Potri.008	0.707553	4.755209	6.88E-06	0.000704	1	283	208	308
Potri.004	0.790802	4.516605	6.76E-06	0.000695	1	199	228	222
Potri.002	0.462894	7.266368	6.67E-06	0.000687	1	1840	1582	1639
Potri.002	0.471667	7.373167	6.66E-06	0.000687	1	1858	1700	1863
Potri.005	0.494577	7.126896	6.61E-06	0.000684	1	1632	1479	1422
Potri.006	0.584493	4.959733	6.61E-06	0.000684	1	344	325	300
Potri.006	0.929966	4.488131	6.53E-06	0.000679	1	233	137	233
Potri.005	0.8859	2.579631	6.39E-06	0.000668	1	47	54	59
Potri.007	0.752355	3.355161	6.24E-06	0.000653	1	101	96	97
Potri.008	0.610595	4.406739	6.16E-06	0.000647	1	218	229	204
Potri.001	0.707213	6.579802	5.96E-06	0.000629	1	764	1096	952
Potri.001	0.496566	6.832116	5.91E-06	0.000625	1	1313	1215	1162
Potri.001	0.51275	6.153491	5.85E-06	0.000622	1	784	724	779
Potri.002	0.543862	5.407209	5.75E-06	0.000614	1	455	433	456
Potri.009	0.662854	4.060452	5.73E-06	0.000613	1	191	154	157
Potri.010	0.984428	2.733993	5.31E-06	0.000574	1	76	44	52
Potri.009	0.585436	4.995533	5.26E-06	0.00057	1	336	326	330
Potri.004	1.233569	1.397149	1.83E-05	0.001581	1	19	20	19
Potri.014	0.550951	5.06178	5.18E-06	0.000565	1	364	359	331
Potri.003	0.699018	6.800049	5.08E-06	0.000555	1	1015	1254	1030
Potri.005	0.835975	3.956708	4.55E-06	0.000503	1	131	125	175
Potri.011	0.566079	6.106496	4.26E-06	0.000477	1	844	632	699
Potri.004	2.106492	0.062683	2.36E-05	0.001951	1	5	4	4
Potri.001	0.466772	7.820235	4.08E-06	0.000461	1	2595	2375	2440
Potri.001	0.566291	5.865582	4.06E-06	0.000461	1	727	520	594
Potri.006	0.571194	5.660598	4.07E-06	0.000461	1	522	490	572
Potri.003	0.676997	4.285084	3.69E-06	0.000426	1	188	212	181
Potri.014	0.622866	5.219058	3.58E-06	0.000417	1	387	376	378
Potri.009	0.513513	7.975802	3.44E-06	0.000404	1	2669	2801	2610
Potri.008	0.546057	5.344242	3.41E-06	0.000402	1	452	438	396
Potri.019	0.499173	6.715776	3.40E-06	0.000401	1	1232	1078	1093
Potri.001	0.522728	7.039502	3.34E-06	0.000395	1	1406	1348	1455
Potri.001	0.557111	6.938344	3.27E-06	0.000388	1	1224	1321	1316
Potri.010	0.708616	4.89776	3.12E-06	0.000371	1	362	264	259
Potri.011	0.603194	4.416322	3.03E-06	0.000363	1	239	211	209
Potri.011	0.870242	3.316949	2.94E-06	0.000354	1	83	108	80
Potri.002	0.531165	5.943155	2.86E-06	0.000346	1	743	621	603
Potri.001	0.755259	3.312036	2.86E-06	0.000346	1	100	93	92
Potri.004	0.647534	6.195357	2.68E-06	0.000327	1	750	852	620
Potri.003	0.473581	8.917812	2.65E-06	0.000326	1	5599	5090	5134

Potri.009	0.540768	6.421683	2.46E-06	0.000306	1	998	811	921
Potri.006	0.641397	5.397664	2.47E-06	0.000306	1	391	444	443
Potri.009	0.762583	3.477223	2.36E-06	0.000295	1	112	107	100
Potri.011	0.948361	3.224493	2.35E-06	0.000295	1	74	103	68
Potri.009	0.632914	5.404899	2.19E-06	0.000277	1	482	390	425
Potri.008	0.830599	3.05757	2.02E-06	0.000256	1	90	73	68
Potri.019	3.501099	-0.95512	3.31E-05	0.002555	1	0	0	2
Potri.014	1.346539	1.503015	3.37E-05	0.00258	1	14	21	24
Potri.013	0.661162	4.606342	1.83E-06	0.000238	1	238	255	239
Potri.014	2.51727	-0.54057	3.54E-05	0.002665	1	2	2	2
Potri.002	0.909062	2.836213	1.70E-06	0.000223	1	60	69	61
Potri.001	0.588708	7.345785	1.67E-06	0.000219	1	1680	1770	1611
Potri.010	0.655169	4.425667	1.62E-06	0.000213	1	243	181	226
Potri.016	0.737958	3.77441	1.62E-06	0.000213	1	136	126	135
Potri.012	0.541208	6.794984	1.55E-06	0.000206	1	1221	1175	1132
Potri.006	0.759921	4.926295	1.55E-06	0.000206	1	259	342	273
Potri.003	0.552013	6.367706	1.50E-06	0.000201	1	877	910	821
Potri.019	0.817301	3.441403	1.48E-06	0.0002	1	124	80	101
Potri.015	0.90904	2.757504	1.32E-06	0.000182	1	62	64	54
Potri.006	0.939677	3.274996	1.31E-06	0.000181	1	77	103	75
Potri.006	1.378534	1.152349	4.79E-05	0.003442	1	14	11	20
Potri.010	0.710086	4.8712	1.22E-06	0.000172	1	258	318	284
Potri.007	0.717803	7.392472	1.22E-06	0.000172	1	1856	1220	1904
Potri.014	0.825607	3.090588	1.20E-06	0.000171	1	73	79	84
Potri.013	1.743446	1.697948	4.91E-05	0.003494	1	10	19	26
Potri.002	0.517821	8.454837	1.10E-06	0.000159	1	4029	3443	3810
Potri.007	1.429796	0.690058	5.23E-05	0.003675	1	13	9	9
Potri.001	1.005888	2.519795	5.26E-05	0.003688	1	45	62	38
Potri.008	1.321274	1.819899	5.29E-05	0.003691	1	32	14	30
Potri.009	0.597398	7.102626	9.61E-07	0.000142	1	1516	1524	1228
Potri.016	0.601879	6.558235	9.44E-07	0.00014	1	923	1016	972
Potri.004	0.504944	8.736081	8.86E-07	0.000132	1	4843	4387	4542
Potri.010	0.515896	8.794318	8.82E-07	0.000132	1	5414	4572	4324
Potri.006	0.852554	3.638804	8.60E-07	0.000129	1	113	118	112
Potri.004	0.689231	6.698208	8.14E-07	0.000123	1	897	1057	1131
Potri.010	0.864234	5.419343	7.74E-07	0.000118	1	401	332	450
Potri.006	0.644365	6.768116	7.56E-07	0.000116	1	1053	1244	1009
Potri.015	0.643881	7.145608	7.50E-07	0.000115	1	1384	1607	1307
Potri.004	0.921895	2.582208	7.43E-07	0.000115	1	53	55	50
Potri.011	2.529404	-0.53817	6.25E-05	0.00425	1	2	2	2
Potri.001	0.691354	4.032509	6.81E-07	0.000107	1	165	160	160
Potri.010	0.567286	7.538353	6.01E-07	9.52E-05	1	1881	2040	1910
Potri.005	0.606765	5.42366	5.81E-07	9.24E-05	1	476	403	448
Potri.005	0.741174	3.641734	5.66E-07	9.04E-05	1	137	115	110
Potri.012	0.797817	3.772911	5.64E-07	9.04E-05	1	140	112	135
Potri.012	0.632683	6.334907	5.62E-07	9.03E-05	1	786	884	791
Potri.002	0.717939	3.963313	5.60E-07	9.03E-05	1	171	137	150
Potri.001	0.597357	5.799112	5.57E-07	9.02E-05	1	594	589	543
Potri.005	0.609214	10.39563	5.20E-07	8.49E-05	1	15449	11616	14731
Potri.010	0.771331	3.946148	4.57E-07	7.58E-05	1	162	135	145
Potri.009	0.906187	4.463189	4.39E-07	7.32E-05	1	182	211	201
Potri.014	0.550702	7.765199	4.25E-07	7.10E-05	1	2553	2124	2230
Potri.004	0.689627	5.400433	4.21E-07	7.06E-05	1	422	461	374
Potri.016	1.507077	0.360871	7.65E-05	0.004935	1	7	9	7

Potri.013	1.820076	-0.13234	8.21E-05	0.00521	1	5	4	4
Potri.003	1.420785	0.554033	8.35E-05	0.005268	1	10	7	11
Potri.001	0.735158	5.876774	3.99E-07	6.86E-05	1	514	675	521
Potri.011	1.018515	2.770018	8.39E-05	0.005274	1	65	40	68
Potri.002	0.599504	5.379166	3.80E-07	6.59E-05	1	424	424	439
Potri.001	0.820627	4.471962	3.76E-07	6.55E-05	1	270	165	193
Potri.004	0.982763	3.650102	3.75E-07	6.54E-05	1	119	103	105
Potri.018	0.846578	5.153065	3.61E-07	6.36E-05	1	299	408	278
Potri.013	1.055542	1.413418	8.54E-05	0.005331	1	23	23	18
Potri.014	0.555657	8.2608	3.41E-07	6.07E-05	1	3481	2881	3351
Potri.002	0.745864	7.455433	3.22E-07	5.76E-05	1	1485	1945	1656
Potri.005	0.95249	2.781292	2.76E-07	4.99E-05	1	69	47	64
Potri.007	2.563311	-0.51199	8.78E-05	0.005435	1	2	2	2
Potri.001	0.643183	5.744253	2.58E-07	4.73E-05	1	633	536	467
Potri.008	0.585206	7.249599	2.46E-07	4.56E-05	1	1680	1632	1439
Potri.004	0.587352	9.066446	2.38E-07	4.43E-05	1	5890	5176	5678
Potri.007	0.547159	7.837176	2.36E-07	4.41E-05	1	2701	2127	2447
Potri.017	1.207868	0.911815	0.000103	0.006159	1	16	14	11
Potri.016	0.711798	5.193853	2.18E-07	4.12E-05	1	324	381	371
Potri.013	1.367312	0.606101	0.000104	0.006159	1	13	11	6
Potri.012	1.274614	1.020006	0.000108	0.006326	1	15	19	9
Potri.007	0.924785	3.357035	1.93E-07	3.72E-05	1	94	81	98
Potri.005	0.70106	10.04928	1.90E-07	3.69E-05	1	12163	8385	11124
Potri.016	3.265383	-0.74634	0.000109	0.006389	1	0	3	0
Potri.001	0.755798	6.44516	1.65E-07	3.27E-05	1	1084	629	837
Potri.013	0.61874	5.124112	1.60E-07	3.20E-05	1	401	337	335
Potri.001	0.886539	4.177162	1.57E-07	3.14E-05	1	157	167	168
Potri.003	0.648803	7.585939	1.39E-07	2.86E-05	1	1824	2157	1836
Potri.001	1.525735	0.918472	0.000125	0.007107	1	12	7	16
Potri.010	5.682523	-1.33278	0.000129	0.007239	1	0	0	0
Potri.016	0.999797	3.444303	1.12E-07	2.34E-05	1	90	93	97
Potri.016	0.550653	7.198006	1.04E-07	2.20E-05	1	1666	1504	1484
Potri.008	0.80407	4.843758	1.03E-07	2.18E-05	1	247	317	246
Potri.001	1.341658	1.801856	0.000131	0.007363	1	31	22	21
Potri.017	0.689259	4.469137	9.57E-08	2.04E-05	1	247	204	209
Potri.005	0.737561	4.289473	9.20E-08	1.97E-05	1	194	182	193
Potri.003	0.813329	6.112858	8.90E-08	1.92E-05	1	783	542	643
Potri.003	5.488767	-1.43455	0.000135	0.007472	1	0	0	0
Potri.001	0.985569	3.380787	7.27E-08	1.62E-05	1	96	82	92
Potri.008	0.796616	6.741656	6.88E-08	1.55E-05	1	1010	1120	914
Potri.014	1.850453	-0.1109	0.000145	0.007973	1	4	5	4
Potri.019	1.272334	1.055919	0.000147	0.008062	1	10	18	16
Potri.012	0.949661	4.711794	5.16E-08	1.20E-05	1	244	222	230
Potri.008	0.776973	10.20572	4.96E-08	1.16E-05	1	13053	11655	9380
Potri.019	2.561512	-0.19358	0.00017	0.008941	1	2	5	1
Potri.006	1.904007	-0.38259	0.000178	0.00931	1	5	3	2
Potri.008	1.249481	1.154535	0.00018	0.009396	1	14	22	12
Potri.013	0.892495	6.591797	3.34E-08	8.44E-06	1	721	824	1074
Potri.008	0.710965	5.00379	3.15E-08	8.00E-06	1	307	330	308
Potri.017	1.025984	1.838813	0.000217	0.010961	1	19	37	32
Potri.013	1.606807	0.119639	0.000226	0.011225	1	5	7	6
Potri.004	1.376181	0.530178	0.00023	0.011387	1	5	12	11
Potri.005	0.901551	5.212703	2.27E-08	6.16E-06	1	376	373	259
Potri.009	0.828579	4.76927	2.20E-08	6.05E-06	1	243	283	236

Potri.001	0.731313	4.44416	2.06E-08	5.72E-06	1	222	196	218
Potri.006	1.007919	1.670261	0.000233	0.011539	1	27	22	30
Potri.001	1.155891	1.588301	0.000241	0.011827	1	35	14	21
Potri.011	1.057643	1.163375	0.000252	0.012264	1	15	22	16
Potri.006	0.821184	4.098018	1.23E-08	3.63E-06	1	177	144	160
Potri.008	0.856411	4.515398	9.19E-09	2.77E-06	1	197	206	228
Potri.011	0.950466	4.08766	9.21E-09	2.77E-06	1	175	145	131
Potri.006	0.680814	7.571514	8.79E-09	2.70E-06	1	1955	1659	2093
Potri.014	0.666096	6.298924	7.14E-09	2.22E-06	1	845	757	774
Potri.006	0.706336	5.70307	6.18E-09	1.99E-06	1	562	453	531
Potri.001	0.861806	4.542708	6.10E-09	1.98E-06	1	199	231	211
Potri.001	0.995222	5.430854	5.81E-09	1.91E-06	1	393	356	374
Potri.005	0.585568	8.338319	5.71E-09	1.89E-06	1	3585	3192	3338
Potri.004	0.64259	6.454205	5.40E-09	1.80E-06	1	917	909	843
Potri.019	1.606988	0.421502	0.000321	0.014773	1	8	10	5
Potri.006	0.794398	4.674602	4.21E-09	1.45E-06	1	253	257	216
Potri.010	0.940053	3.493362	3.99E-09	1.39E-06	1	118	97	84
Potri.005	0.794099	5.16942	3.57E-09	1.27E-06	1	394	325	309
Potri.002	0.766393	6.42309	3.40E-09	1.22E-06	1	824	780	872
Potri.008	0.685812	5.908139	3.35E-09	1.21E-06	1	642	600	554
Potri.001	0.816923	6.304792	3.27E-09	1.19E-06	1	721	841	664
Potri.003	0.760154	9.0103	2.54E-09	9.65E-07	1	4383	5110	5379
Potri.009	0.731245	6.099768	2.23E-09	8.55E-07	1	770	580	668
Potri.009	0.722035	5.910298	1.74E-09	6.81E-07	1	665	567	542
Potri.008	1.694969	0.028657	0.000389	0.017094	1	6	4	6
Potri.010	0.757716	5.785026	1.35E-09	5.43E-07	1	627	480	497
Potri.019	1.205535	2.281221	0.00041	0.017805	1	36	46	30
Potri.002	0.625938	8.202611	7.72E-10	3.16E-07	1	3224	2855	2975
Potri.007	0.950114	4.197883	7.47E-10	3.11E-07	1	173	174	139
Potri.004	0.804029	5.124847	5.24E-10	2.25E-07	1	364	318	309
Potri.005	0.739379	5.528423	4.85E-10	2.10E-07	1	454	450	442
Potri.006	1.823733	-0.43346	0.000472	0.019883	1	5	1	4
Potri.010	0.838172	4.608944	3.06E-10	1.41E-07	1	236	198	247
Potri.002	0.768995	6.275182	1.91E-10	9.31E-08	1	756	748	728
Potri.008	1.069037	1.244425	0.000488	0.020283	1	18	20	18
Potri.T16	0.689785	8.712988	9.39E-11	4.80E-08	1	4136	4211	4171
Potri.004	0.830755	5.922182	8.48E-11	4.46E-08	1	538	576	583
Potri.018	1.013358	1.634227	0.000526	0.021366	1	29	15	33
Potri.001	5.204082	-1.59757	0.00055	0.022035	1	0	0	0
Potri.003	1.072245	0.980459	0.000577	0.022949	1	15	10	21
Potri.008	0.857258	5.846469	5.15E-12	3.25E-09	1	491	552	547
Potri.001	3.7253	-1.33555	0.000683	0.026328	1	0	0	1
Potri.008	0.824999	7.258946	4.58E-12	2.98E-09	1	1489	1318	1508
Potri.002	1.736032	-0.19655	0.000798	0.029786	1	9	0	4
Potri.003	1.433217	0.933893	0.000812	0.030254	1	5	8	24
Potri.011	1.018253	1.417133	0.000851	0.031361	1	14	27	24
Potri.010	0.74014	9.102887	1.51E-12	1.18E-09	1	5500	5392	5177
Potri.001	0.896228	5.205854	1.31E-12	1.04E-09	1	326	332	345
Potri.017	0.804246	7.618499	8.10E-13	6.69E-10	1	2000	1630	1966
Potri.015	1.099684	0.81667	0.000991	0.035047	1	11	18	11
Potri.001	1.152303	0.783716	0.001081	0.037201	1	10	19	9
Potri.007	1.14463	0.630272	0.001096	0.037523	1	11	9	14
Potri.018	0.98888	6.169799	2.14E-14	2.30E-11	1	650	581	651
Potri.012	1.052703	1.470458	0.001125	0.038141	1	25	18	24

Potri.004	0.843453	7.707678	1.28E-14	1.57E-11	1	1968	1960	1906
Potri.010	1.020813	1.209087	0.0012	0.039925	1	24	12	20
Potri.010	1.480555	0.043815	0.001229	0.040439	1	6	6	6
Potri.013	1.668608	0.610926	0.001254	0.040982	1	10	10	6
Potri.017	5.205367	-1.59753	0.001255	0.040982	1	0	0	0
Potri.017	2.56553	-0.68844	0.001264	0.041153	1	2	1	2
Potri.005	0.934934	5.432813	3.70E-16	5.88E-13	1	425	363	370
Potri.006	1.51074	-0.24003	0.001269	0.041212	1	5	4	5
Potri.001	0.948165	7.088853	6.55E-17	1.17E-13	1	1323	1205	1104
Potri.003	2.555404	-0.92061	0.001322	0.042667	1	2	2	0
Potri.017	0.914815	9.743442	2.12E-17	4.55E-14	1	7821	7382	7982
Potri.018	3.572855	-1.43313	0.001357	0.043438	1	0	0	1
Potri.019	1.247836	0.297813	0.001383	0.044167	1	13	7	5
Potri.002	3.557894	-1.43449	0.0014	0.044431	1	0	0	1
Potri.011	1.556609	-0.13028	0.001435	0.045124	1	4	7	4
Potri.018	1.519665	-0.15404	0.001445	0.045275	1	6	1	8
Potri.013	1.390217	0.116796	0.001456	0.045431	1	5	7	8
Potri.003	1.292308	0.170408	0.00148	0.046018	1	6	7	9
Potri.T13	4.970792	-1.71794	0.001505	0.046558	1	0	0	0
Potri.003	1.179006	0.405268	0.001531	0.047152	1	6	11	11

PagLBD21C	PagLBD21C	PagLBD21C	Pfam	Panther	KOG	ec	KO	GO
26	21	13	PF03181	PTHR31236,PTHR31236:SF2				
91	105	67	PF13499	PTHR10891	KOG0027		K13448	
433	393	378	PF11820	PTHR33128				
49	67	41	PF03094	PTHR31942,PTHR31942:SF20			K08472	GO:001602
3478	3331	3576		PTHR21091,PTHR2109		1.5.1.20		
160	224	181	PF02338	PTHR12419,PTHR1241		3.4.19.12		
49	56	54	PF14215	PTHR13902,PTHR13902:SF3				
42	29	22	PF05678	PTHR33179,PTHR33179:SF7				
48	48	47	PF14416,P	PTHR32285,PTHR32285:SF48				
19	29	12	PF02458	PTHR31623,PTHR3162		2.3.1.160		GO:001674
683	671	591	PF14416,P	PTHR32285,PTHR32285:SF7				
791	914	989	PF00168	PTHR31208,PTHR31208:SF3				GO:000551
185	198	205	PF07731,P	PTHR11709,PTHR1170		1.10.3.2	K05909	GO:005511
60	54	67		PTHR24115,PTHR24115:SF449				GO:000701
1078	1283	1172		PTHR35301,PTHR35301:SF1				
64	42	39	PF05199,P	PTHR10668,PTHR1066		1.1.3.20	K17756	GO:005511
6	12	5	PF02458	PTHR31623,PTHR3162		2.3.1.160		GO:001674
165	146	204	PF08449	PTHR10778	KOG1582		K15277	GO:005508
42	53	46	PF00560	PTHR27004	KOG0472	2.7.11.1		GO:000551
163	165	184	PF07690,P	PTHR23500	KOG0254		K08150	GO:005508
2260	2507	2647	PF14569,P	PTHR13301,PTHR1330		2.4.1.12		GO:003024
1018	1108	876	PF00249	PTHR10641,PTHR10641:SF484			K09422	
21	22	14	PF00067	PTHR24286	KOG0157			GO:005511
181	189	103	PF00314	PTHR31048,PTHR31048:SF32				
341	336	349	PF00249	PTHR12802	KOG0724			
33	17	23	PF14226,P	PTHR10209	KOG0143	1.14.11.20		GO:005511
141	135	114	PF00571	PTHR11911				
348	243	135	PF13561	PTHR24322	KOG0725			
1741	1617	1935		PTHR34113,PTHR34113:SF3				
88	82	91		PTHR39113,PTHR39113:SF1				
24	13	19	PF02365	PTHR31744,PTHR31744:SF6				GO:000635
717	784	712		PTHR31348,PTHR31348:SF2				
184	185	257	PF05678	PTHR33179,PTHR33179:SF4				
53	37	23	PF00657	PTHR22835,PTHR2283		3.1.1.3		GO:001678
164	125	169		PTHR10774,PTHR10774:SF95				
74	87	88	PF11820	PTHR33128,PTHR33128:SF10				
74	74	84		PTHR34947,PTHR34947:SF2				
103	123	165	PF06398,P	PTHR10024,PTHR10024:SF243				GO:000551
58	34	41	PF00447	PTHR10015	KOG0627		K09419	GO:004356
24	27	18	PF00892	PTHR31218,PTHR31218:SF33				GO:001602
459	490	369	PF03766,P	PTHR31775				
217	254	255		PTHR33709,PTHR33709:SF3				
35	34	15	PF00234	PTHR33076,PTHR33076:SF4				
96	64	61		PTHR10460,PTHR10460:SF10				
63	79	64	PF02458	PTHR31623,PTHR3162		2.3.1.160		GO:001674
86	111	115	PF03134,P	PTHR12300,PTHR12300:SF43				
30	39	35	PF08276,P	PTHR27002	KOG1187	2.7.11.1		GO:004854
286	318	262	PF13419	PTHR12725	KOG3109	3.1.3.74	K07025	
198	192	203	PF00808	PTHR10252,PTHR10252:SF48			K08066	
553	616	565	PF00046	PTHR24326	KOG0484			GO:000367
98	145	94	PF00847	PTHR31677,PTHR31677:SF17			K09286	GO:000635

953	977	907	PF04110	PTHR10969	KOG1654		K08341	GO:000573
34	31	32	PF00481	PTHR13832	KOG0698	3.1.3.16	K14803	GO:000382
176	170	212	PF02458	PTHR31642,PTHR31642:SF5				GO:001674
556	580	545	PF12697	PTHR10992,PTHR1099		3.1.1.1		
192	220	163	PF02893	PTHR31969,PTHR31969:SF1				
18	25	22	PF01477,P	PTHR11771,PTHR1177		1.13.11.1	K00454	GO:000551
56	56	45	PF00388	PTHR13593	KOG4306	3.1.4.11,4.6.1.13		
123	144	150	PF06955,P	PTHR31062,PTHR3106		2.4.1.207		GO:004804
103	84	125	PF00412	PTHR24206	KOG1700		K09377	GO:000827
186	217	214	PF05623	PTHR32010,PTHR32010:SF8				
863	793	688	PF00141	PTHR31235,PTHR3123		1.11.1.7	K00430	GO:005511
102	141	113	PF05142	PTHR31604,PTHR31604:SF2				
71	53	36	PF02458	PTHR31642,PTHR3164		2.3.1.84		GO:001674
760	663	579	PF08477	PTHR24072	KOG0393		K04392	GO:000726
322	379	424	PF13306,P	PTHR24006	KOG4341			GO:000551
501	494	384		PTHR36040,PTHR36040:SF2				
80	114	82	PF06232	PTHR31718,PTHR31718:SF9				
1588	1672	1638	PF00241	PTHR11913	KOG1735		K05765	GO:000562
88	98	62	PF00170,P	PTHR22952,PTHR22952:SF179			K14431	GO:004356
39	42	8	PF00232	PTHR10353,PTHR1035		3.2.1.21		GO:000597
630	657	585	PF13632	PTHR32044		2.4.1.32	K13680	
637	636	697		PTHR34280,PTHR34280:SF2				
250	279	314	PF13414	PTHR23083,PTHR23083:SF444				
5	5	2	PF03055	PTHR10543,PTHR1054		1.13.11.51		
2	5	1	PF03181	PTHR31236,PTHR31236:SF2				
286	346	279	PF00462	PTHR10168	KOG1752	1.8.1.9,1	K03676	GO:004545
526	491	536	PF00249	PTHR24078	KOG0724			
4	3	5	PF00067	PTHR24298	KOG0156	1.14.13.8	K13083	GO:005511
156	159	182	PF01490	PTHR22950	KOG1303			
48	53	36	PF03492	PTHR31009,PTHR3100		2.1.1.278	K18848	GO:000816
100	83	77	PF01190	PTHR33210				
83	91	82		PTHR33736,PTHR33736:SF1				
87	71	96		PTHR36375,PTHR36375:SF1				
1047	1215	1337	PF02225,P	PTHR10795,PTHR1079		3.4.14.10		GO:000650
49	54	37	PF01657,P	PTHR27002	KOG1187	2.7.11.1		GO:000646
1313	1282	1391	PF00026	PTHR13683	KOG1339	3.4.23.12		GO:000650
1001	1329	1426	PF00083	PTHR11662	KOG2533	3.6.3.20	K13783	GO:005508
1945	2038	1867	PF01424,P	PTHR15672	KOG2953			GO:000367
265	229	295	PF14510,P	PTHR19241,PTHR1924		3.6.3.25		GO:001688
116	80	80	PF02701	PTHR31992,PTHR31992:SF12				GO:000635
1605	1928	2137	PF03765,P	PTHR23324	KOG1471			
408	436	371		PTHR10593,PTHR10593:SF27				
470	440	461		PTHR33738,PTHR33738:SF1				
114	122	102	PF00249	PTHR31003,PTHR31003:SF4				
122	83	56	PF01439	PTHR33543				GO:004687
25	17	16		PTHR35466,PTHR35466:SF2				
164	158	171	PF03188	PTHR23130,PTHR23130:SF88				GO:001602
38	61	44	PF00378	PTHR11941,PTHR11941:SF84				GO:000815
1619	1514	1528	PF08267,P	PTHR30519,PTHR3051		2.1.1.14	K00549	GO:000865
202	225	195	PF05678	PTHR33179,PTHR33179:SF9				
109	148	123	PF12937	PTHR24006				GO:000551
365	356	264	PF13410,P	PTHR11260	KOG0406	2.5.1.18	K00799	GO:000551
67	75	52	PF01439	PTHR33543				GO:004687
164	146	131	PF03479	PTHR31100,PTHR31100:SF16				

465	411	452	PF04788	PTHR31300,PTHR31300:SF8				
39	48	32	PF00249	PTHR10641	KOG0048		K09422	
72	90	58	PF00847	PTHR31677,PTHR31677:SF25				GO:000635
36	26	36		PTHR15852,PTHR15852:SF15				
43	48	51	PF03141	PTHR10108,PTHR10108:SF817				GO:000816
23	52	35	PF13921	PTHR10641	KOG0048		K09422	
880	917	956	PF00097,F	PTHR11685	KOG1812			GO:004687
499	501	611	PF13639	PTHR22937	KOG0320			GO:000827
250	260	257	PF00892	PTHR31218,PTHR31218:SF64				GO:001602
22	23	18	PF04577	PTHR20961	KOG4698	2.4.1.255	K18207	GO:001675
747	790	849	PF10358	PTHR31182,PTHR31182:SF3				
170	182	153	PF02458	PTHR31147,PTHR3114		2.3.1.162		GO:001674
638	649	731	PF01501	PTHR11183	KOG1950	2.4.1.17		GO:001675
2041	2257	2814	PF00612,F	PTHR32295,PTHR32295:SF18				GO:000551
142	129	100		PTHR37078,PTHR37078:SF1				
5	8	1	PF00232	PTHR10353,PTHR1035		3.2.1.21		GO:000597
54	65	50	PF12734	PTHR31568				
10	7	1	PF01501	PTHR11183	KOG1950	2.4.1.123	K18819	GO:001675
65	79	47	PF06955,F	PTHR31062,PTHR3106		2.4.1.207	K08235	GO:004804
208	218	257	PF02358	PTHR10788,PTHR1078		3.1.3.12	K01087	GO:000599
165	154	178	PF13639,F	PTHR21319	KOG1940	6.3.2.19	K10144	GO:000827
482	538	445	PF04832	PTHR11220				
146	143	153		PTHR31096,PTHR3109		2.7.7.59		
163	198	212	PF00149,F	PTHR22953,PTHR2295		3.1.4.1		GO:001678
184	181	232		PTHR33133,PTHR33133:SF3				
66	78	100	PF00786	PTHR23177				
85	76	49	PF00657	PTHR22835,PTHR2283		3.1.1.3		GO:001678
437	460	535	PF00221	PTHR10362,PTHR1036		4.3.1.24	K10775	
334	337	311	PF13639	PTHR22763	KOG0320			GO:000827
163	204	168	PF12678	PTHR22763,PTHR22763:SF34				GO:000827
5536	5908	6019	PF08267,F	PTHR30519,PTHR3051		2.1.1.14	K00549	GO:000865
59	67	61	PF06127	PTHR34205,PTHR34205:SF2				
2138	2345	2636	PF08263,F	PTHR27008	KOG1187	2.7.11.1		GO:000646
184	229	185	PF10469	PTHR13360,PTHR13360:SF1				
63	58	45	PF04535	PTHR11615,PTHR11615:SF159				
296	348	343	PF00170	PTHR22952,PTHR22952:SF182				GO:004356
47	56	52	PF00847	PTHR31194,PTHR31194:SF4				GO:000635
522	539	680		PTHR34361,PTHR34361:SF2				
413	428	436	PF14299	PTHR32278,PTHR32278:SF3				
426	422	378	PF03352	PTHR31116,PTHR3111		3.2.2.20	K01246	GO:000872
7	7	5						
3	3	2	PF08031,F	PTHR32448		1.3.3.8		GO:005511
136	179	165	PF13837	PTHR21654,PTHR21654:SF10				
38	42	43						
36	23	22	PF00005,F	PTHR19241,PTHR19241:SF180				GO:001688
326	296	277	PF02183,F	PTHR24326	KOG0483		K09338	GO:004356
55	54	60	PF03106	PTHR31429,PTHR31429:SF16				GO:004356
226	279	172	PF02458	PTHR31642,PTHR3164		2.3.1.188	K15400	GO:001674
51	38	31	PF01370	PTHR32487,PTHR3248		1.3.99.6,1.3.1.3		GO:005066
47	27	40	PF04640	PTHR31065,PTHR31065:SF12				
22	32	30	PF07714	PTHR27001	KOG1187	2.7.11.1,2.7.10.2		GO:000646
325	375	334	PF13193,F	PTHR24096,PTHR2409		6.2.1.34,	K01904	GO:000815
194	251	286	PF00067	PTHR24282	KOG0157			GO:005511
242	209	153	PF02365	PTHR31989,PTHR31989:SF23				GO:000635

143	185	194	PF13499	PTHR23056	KOG0034			
181	176	161	PF13962	PTHR24177,PTHR24177:SF11				
244	329	385	PF00010	PTHR12565	KOG3116,KOG4304			GO:004698
170	173	100	PF00067	PTHR24296	KOG0157	1.14.15.3	K15402	GO:005511
375	350	430	PF13923	PTHR12313	KOG0823	6.3.2.19	K10666	
37	29	32	PF14226,F	PTHR10209	KOG0143		K05282	GO:005511
50	40	59	PF00005,F	PTHR19241,PTHR19241:SF258				GO:001688
688	587	574	PF03791,F	PTHR11850	KOG0773			GO:000563
21	28	38	PF02171	PTHR22891,PTHR22891:SF4				GO:000367
59	58	77	PF07731,F	PTHR11709,PTHR1170		1.10.3.2	K05909	GO:005511
104	112	68	PF00067	PTHR24296	KOG0157	1.14.15.3	K15402	GO:005511
97	105	100	PF01823	PTHR33199,PTHR33199:SF8				
109	144	130						
78	68	92	PF00221	PTHR10362,PTHR1036		4.3.1.24	K10775	
148	163	191	PF03479	PTHR31500,PTHR31500:SF22				
1229	1246	1157	PF00076	PTHR24012	KOG0148		K13201	GO:000367
103	117	106		PTHR36740				
78	74	77	PF00484	PTHR11002	KOG1578	4.2.1.1		GO:000827
168	179	202	PF00249	PTHR12802	KOG0724			
208	202	183	PF04852	PTHR31165,PTHR31165:SF9				
518	598	619	PF07732,F	PTHR11709,PTHR1170		1.10.3.2	K05909	GO:000550
1374	1416	1562	PF05920,F	PTHR11850	KOG0773			GO:000635
0	0	0	PF13193,F	PTHR24095,PTHR24095:SF189				GO:000815
211	210	204	PF04640	PTHR31065,PTHR31065:SF8				
104	101	70	PF07649	PTHR13871,PTHR1387		2.7.1.107		GO:005511
56	54	55		PTHR26312,PTHR26312:SF75				
54	49	45	PF07714	PTHR24347,PTHR2434		2.7.11.1		GO:000646
320	339	216	PF13202	PTHR10891,PTHR10891:SF575				GO:000550
2	1	2	PF01734	PTHR32176	KOG0513			GO:000662
873	924	874	PF03283	PTHR21562,PTHR21562:SF13				GO:001678
1087	1126	1039	PF10539	PTHR10857,PTHR10857:SF54				
251	271	321	PF08079,F	PTHR11524	KOG3184		K02937	
329	330	268	PF13716	PTHR11106	KOG2633			
74	88	61						
816	941	857	PF00685	PTHR11783	KOG1584	2.8.2.27		GO:000814
136	124	107	PF13499	PTHR10891	KOG0027			
50	33	42		PTHR33672,PTHR3367		2.3.3.16		
210	181	184		PTHR33124,PTHR33124:SF12				
41	46	26		PTHR33088				
87	116	102	PF03552	PTHR13301,PTHR1330		2.4.1.12		GO:003024
45	47	40	PF12776	PTHR31704,PTHR31704:SF4				
447	436	304	PF14368	PTHR33044,PTHR33044:SF36				
63	81	77	PF04110	PTHR10969	KOG1654		K08341	GO:000573
87	107	83	PF00571	PTHR13780,PTHR13780:SF39				
87	114	73	PF13912	PTHR26374,PTHR26374:SF208				
114	125	156	PF12638	PTHR31750,PTHR31750:SF4				
29	34	24	PF14368	PTHR33044,PTHR33044:SF36				
20	22	18	PF03398	PTHR12161	KOG2027			
156	189	182	PF06943	PTHR31747,PTHR31747:SF1				
152	119	122	PF00249	PTHR10641	KOG0048		K09422	
423	459	477	PF01490	PTHR22950	KOG1303			
162	177	162	PF04674	PTHR31279,PTHR31279:SF4				
85	83	69	PF01490	PTHR22950	KOG1303			
33	63	36	PF00314	PTHR31048,PTHR31048:SF10				

158	133	138		PTHR33738,PTHR33738:SF2				
84	80	73		PTHR33098,PTHR33098:SF8				
165	147	232	PF05678	PTHR33179,PTHR33179:SF5				
81	79	111	PF13639	PTHR14155,PTHR14155:SF145				GO:000827
576	538	521		PTHR31789,PTHR31789:SF5				
28	35	45	PF00076	PTHR24012	KOG0149			GO:000367
33	38	14	PF02365	PTHR31744,PTHR31744:SF3				GO:000635
75	91	50	PF03766,F	PTHR31775				
115	101	96						
127	148	106	PF01643	PTHR31727,PTHR3172		3.1.2.14	K10781	GO:001679
172	192	151	PF00141	PTHR31388,PTHR3138		1.11.1.7	K00430	GO:005511
713	699	587		PTHR33181,PTHR33181:SF4				
20	33	27	PF04678	PTHR13462	KOG2966			
942	936	919	PF07883	PTHR31238,PTHR31238:SF18				
228	243	229	PF01842	PTHR31096,PTHR3109		2.7.7.59		GO:001659
60	65	70	PF13639	PTHR14155,PTHR14155:SF178				GO:000827
287	267	326	PF14531,F	PTHR24347	KOG0583	2.7.11.1		GO:000716
28	42	36	PF02365	PTHR31744,PTHR31744:SF4				GO:000635
218	220	212	PF03372	PTHR14859,PTHR14859:SF2				
307	238	292	PF03999	PTHR19321	KOG4302		K16732	GO:000801
49	54	65	PF13499,F	PTHR10891	KOG0027		K13448	
65	87	65	PF02365	PTHR31989,PTHR31989:SF45				GO:000635
73	97	84	PF04110	PTHR10969	KOG1654		K08341	GO:000573
1642	1762	1958	PF02469	PTHR32077,PTHR32077:SF7				
31	35	24	PF03106	PTHR31221,PTHR31221:SF37				GO:004356
83	71	60	PF01425	PTHR11895	KOG1211	3.5.1.99		GO:001688
88	81	82	PF08263,F	PTHR27008	KOG1187	2.7.11.1		GO:000551
93	61	50	PF03106	PTHR31221,PTHR31221:SF19				GO:004356
27	46	47	PF10250	PTHR31818,PTHR31818:SF0				
9	8	6	PF03321	PTHR31901,PTHR31901:SF12			K14487	
175	214	178	PF08596	PTHR10241	KOG1983			
624	726	795	PF07731,F	PTHR11709,PTHR1170		1.10.3.2	K05909	GO:005511
361	379	359	PF02365	PTHR31079,PTHR31079:SF5				GO:000635
111	109	82		PTHR35282,PTHR35282:SF2				
124	140	143	PF00249	PTHR10641	KOG0048		K09422	
58	48	67	PF00249	PTHR10641	KOG0048		K09422	
263	191	159	PF03015,F	PTHR11011	KOG1221	1.2.1.50,	K13356	GO:008001
2045	2257	2645	PF01490	PTHR22950	KOG1303		K13946	
68	54	48	PF00909	PTHR11730	KOG0682		K03320	GO:001602
38	77	63	PF00067	PTHR24298	KOG0156	1.14.13.21		GO:005511
59	48	66	PF01740,F	PTHR11814,PTHR11814:SF60			K17471	GO:001602
210	154	140		PTHR33088				
93	88	79	PF00153	PTHR24089	KOG0762		K15109	
78	108	84	PF14368	PTHR33214,PTHR33214:SF6				
240	267	267	PF03009	PTHR22958	KOG2421	3.1.4.46		GO:000808
211	204	215	PF03479	PTHR31100,PTHR31100:SF3				
48	45	42	PF13947,F	PTHR27009	KOG1187	3.1.4.46		GO:003024
74	50	76	PF01490	PTHR22950	KOG1303			
2746	2746	3122	PF13632,F	PTHR13301,PTHR1330		2.4.1.12	K10999	
1513	1497	1484		PTHR13902,PTHR1390		2.7.11.1		
84	67	26	PF01439	PTHR33543				GO:004687
44	39	41		PTHR36063				
26	34	15	PF00892	PTHR31218,PTHR31218:SF12				GO:001602
1320	1409	1429	PF14416,F	PTHR32285,PTHR32285:SF10				GO:005082

25	31	12	PF07883	PTHR31238,PTHR31238:SF44				
15	24	32	PF01619	PTHR13914	KOG0186	1.5.5.2	K00318	GO:000656
6	9	11	PF12681	PTHR10374	KOG2944			
94	114	100		PTHR10374,PTHR10374:SF11				
160	167	154	PF13837	PTHR21654,PTHR21654:SF10				
232	278	292	PF03141	PTHR10108,PTHR10108:SF812				GO:000816
2423	2648	2783	PF00201	PTHR11926,PTHR11926:SF343				GO:001675
64	34	44	PF01486,F	PTHR11945	KOG0014			GO:000635
121	104	106	PF05705	PTHR12265	KOG2521			
17	20	30	PF13639	PTHR14155,PTHR14155:SF94				GO:000827
61	72	79	PF04043,F	PTHR31707,PTHR3170		3.1.1.11		GO:000485
654	725	849	PF03168	PTHR31234,PTHR31234:SF6				
161	112	105	PF01419	PTHR23244,PTHR23244:SF249				GO:008002
94	91	76		PTHR35290,PTHR35290:SF2				
534	506	499		PTHR34959,PTHR34959:SF1				
81	84	76	PF10033	PTHR13430	KOG4573		K08331	
355	344	388	PF12043	PTHR31390,PTHR31390:SF2				
94	71	43	PF00249	PTHR10641	KOG0048		K09422	
577	689	685	PF03096	PTHR11034	KOG2931			
30	31	43	PF14531,F	PTHR27002	KOG1187	2.7.11.1	K04733	
145	164	165	PF00069,F	PTHR27007	KOG1187	2.7.11.1		GO:000646
262	326	241	PF14259					
379	368	379	PF05911	PTHR31580,PTHR31580:SF5				
48	55	49		PTHR33124,PTHR33124:SF8				
1982	2199	1928	PF00067	PTHR24298,PTHR24298:SF73			K09755	GO:005511
573	564	622	PF02469	PTHR32077,PTHR32077:SF10				
28	52	23	PF00249	PTHR10641	KOG0048		K09422	
1277	1104	755	PF01048	PTHR21234,PTHR2123		3.2.2.9		GO:000911
95	136	93	PF00704	PTHR11177		3.2.1.14,	K01183	GO:000597
304	326	335	PF14364,F	PTHR33098,PTHR33098:SF1				
37	44	51	PF00690,F	PTHR24093,PTHR2409		3.6.3.8	K01537	GO:000551
83	99	94	PF07816	PTHR33095,PTHR33095:SF10				
669	797	821	PF08263,F	PTHR27000	KOG0472	2.7.11.1		GO:000551
823	785	662	PF00854	PTHR11654,PTHR11654:SF146				GO:001602
67	64	67	PF15365	PTHR33670,PTHR33670:SF2				
422	470	398		PTHR34936,PTHR34936:SF1				
94	102	100	PF08264,F	PTHR11946,PTHR1194		6.1.1.4	K01869	GO:000641
3	4	5		PTHR36016				
534	567	522	PF04669	PTHR31444,PTHR31444:SF8				
2851	2609	1839	PF14368	PTHR33122,PTHR33122:SF6				
490	501	505	PF03168	PTHR31234,PTHR31234:SF6				
242	296	307		PTHR35274,PTHR35274:SF1				
45	64	51	PF13193,F	PTHR24095,PTHR24095:SF189				GO:000815
20	25	22		PTHR36615,PTHR36615:SF1				
91	135	110	PF05577	PTHR11010,PTHR1101		3.4.16.2	K01285	GO:000823
566	591	372	PF00067	PTHR24296	KOG0157	1.14.14.1	K15401	GO:005511
62	84	86		PTHR16223,PTHR16223:SF21				
27	28	30	PF01740,F	PTHR11814,PTHR11814:SF103			K17469	GO:001602
61	52	36	PF03106	PTHR31221,PTHR31221:SF7				GO:004356
611	556	510	PF04398	PTHR31676,PTHR31676:SF12				
274	203	276	PF04759	PTHR31696,PTHR31696:SF15				
283	255	260	PF07732,F	PTHR11709,PTHR1170		1.10.3.2	K05909	GO:000550
249	288	300	PF05000,F	PTHR19376,PTHR1937		2.7.7.6	K16250	GO:000635
37	33	25	PF01565,F	PTHR13878,PTHR1387		1.5.99.12	K00279	GO:005511

234	262	227	PF11820	PTHR33128,PTHR33128:SF13				
220	234	228	PF03676	PTHR13465	KOG2819			
1685	1840	1668		PTHR33474,PTHR33474:SF2				
59	42	25	PF03015,F	PTHR11011	KOG1221	1.2.1.84	K13356	GO:008001
138	116	114	PF01823	PTHR33199,PTHR33199:SF4				
175	157	221	PF00067	PTHR24296	KOG0157			GO:005511
78	108	88	PF00400	PTHR22844	KOG0271,K	2.7.11.7		GO:000551
247	252	291	PF02386	PTHR31064				GO:005508
222	231	244	PF01733	PTHR10332	KOG1479		K15014	GO:001602
0	1	0	PF00249	PTHR10641	KOG0048		K09422	
106	112	77	PF01553	PTHR15486,PTHR1548		2.3.1.15	K13508	GO:001674
77	57	69	PF13561	PTHR24322	KOG0725	1.3.1.9	K00208	
609	535	580	PF03999	PTHR19321	KOG4302		K16732	GO:000801
925	1010	1100	PF01501	PTHR13778,PTHR1377		2.4.1.43		GO:001675
476	522	374	PF02309	PTHR31734,PTHR31734:SF11			K14484	GO:000635
123	121	118	PF04043	PTHR31080,PTHR3108		3.1.1.11		GO:000485
7	5	5	PF00249	PTHR31314,PTHR31314:SF10				
120	125	76		PTHR33348,PTHR33348:SF3				
713	693	673	PF02309	PTHR31734,PTHR31734:SF6			K14484	GO:000635
154	139	131	PF07714	PTHR27001	KOG1187	2.7.11.1		GO:000646
165	160	191	PF05920,F	PTHR11850	KOG0773			GO:000635
820	891	1084	PF06955,F	PTHR31062,PTHR3106		2.4.1.207	K08235	GO:004804
276	263	232	PF13347	PTHR19432,PTHR19432:SF38			K15378	
191	211	133	PF01553	PTHR15486,PTHR1548		2.3.1.15,	K13508	GO:001674
879	805	585	PF01734	PTHR32176	KOG0513	3.1.1.26		GO:000662
37	46	47	PF00249	PTHR10641	KOG0048		K09422	
87	80	54	PF07690	PTHR11654,PTHR11654:SF90			K14638	GO:005508
173	163	169	PF05920,F	PTHR11850	KOG0773			GO:000635
88	67	25	PF08659	PTHR24322	KOG0725	1.1.1.206	K08081	
74	95	92	PF07911	PTHR33108,PTHR33108:SF2				
254	262	185	PF00249	PTHR10641	KOG0048		K09422	
79	77	96	PF01925	PTHR14255,PTHR14255:SF6				GO:001602
155	185	178		PTHR27000,PTHR27000:SF167				
2531	2473	2599						
12	19	20		PTHR24078,PTHR24078:SF255				
4	3	3	PF01501	PTHR11183	KOG1950	2.4.1.123	K18819	GO:001675
69	71	77		PTHR14237		2.8.1.9		
322	343	313	PF04525	PTHR31087,PTHR31087:SF11				
159	155	104	PF00171	PTHR11699,PTHR1169		1.2.1.3	K00128	GO:005511
27	35	24		PTHR33181,PTHR33181:SF7				
81	71	82	PF00083	PTHR23500	KOG0254		K08150	GO:005508
18	10	13	PF04640	PTHR31065,PTHR31065:SF12				
49	27	28	PF12708	PTHR31375,PTHR3137		3.2.1.15	K01184	
272	281	279	PF12734	PTHR31568,PTHR31568:SF15				
10801	11404	11613	PF08100,F	PTHR11746	KOG3178	2.1.1.68	K13066	GO:004698
357	281	165	PF00067	PTHR24296	KOG0157	1.14.14.1	K15401	GO:005511
127	95	88	PF00314	PTHR31048,PTHR31048:SF5				
137	164	168	PF03106	PTHR31221,PTHR31221:SF12			K13424	GO:004356
226	223	212	PF03055	PTHR10543	KOG1285	1.13.11.7	K17913	
2348	2137	1347	PF14368	PTHR33122,PTHR33122:SF6				
306	328	342	PF02984,F	PTHR10177	KOG0656		K14505	GO:000563
4	7	0	PF07883	PTHR31238,PTHR31238:SF44				
485	425	299	PF00141	PTHR31235,PTHR3123		1.11.1.7	K00430	GO:005511
4	7	6	PF03195	PTHR31301,PTHR31301:SF22				

777	741	848	PF00394,P	PTHR11709,PTHR1170		1.10.3.2	K05909	GO:005511
2094	2227	2656	PF00221	PTHR10362,PTHR1036		4.3.1.24	K10775	
78	62	57	PF13947,P	PTHR27005	KOG1187	2.7.11.1		GO:003024
7	14	8	PF00403	PTHR22814	KOG1603			GO:004687
3311	3030	2855	PF00067	PTHR24298	KOG0156		K09755	GO:005511
52	65	54	PF02365	PTHR31744,PTHR31744:SF10				GO:000635
86	69	75	PF00786	PTHR23177,PTHR23177:SF23				
6484	7269	6978	PF00067	PTHR24298	KOG0156	1.14.13.1	K00487	GO:005511
61	66	57	PF07690	PTHR23515,PTHR23515:SF3			K02575	GO:005508
256	348	172						
112	123	76	PF03763	PTHR31775				
35	32	29	PF03169	PTHR31645,PTHR31645:SF1				GO:005508
11	19	15	PF00657	PTHR22835,PTHR2283		3.1.1.3		GO:001678
915	1053	937		PTHR13833				
267	288	294	PF00069	PTHR27001	KOG1187	2.7.11.1		GO:000646
671	707	591	PF07687,P	PTHR11014,PTHR11014:SF62			K14664	GO:001678
248	254	320	PF02365	PTHR31989,PTHR31989:SF39				GO:000635
15	10	13						
3	7	1	PF02458	PTHR31623,PTHR3162		2.3.1.160		GO:001674
335	325	468	PF02309,P	PTHR31384,PTHR31384:SF10			K14486	GO:000635
403	421	471	PF03822,P	PTHR24343	KOG0583	2.7.11.1	K07198	GO:000716
391	579	618	PF10250	PTHR31741,PTHR31741:SF11				
41	53	36	PF05577	PTHR11010,PTHR1101		3.4.16.2	K01285	GO:000823
359	320	329	PF00083	PTHR11662	KOG2532	3.6.3.27	K08193	GO:005508
41	33	43	PF09425,P	PTHR33077,PTHR33077:SF6			K13464	
55	48	44	PF00135	PTHR23024	KOG1515	3.1.1.1	K14493	
21	31	27	PF07714	PTHR27003	KOG1187	2.7.11.1		GO:000646
10	13	15	PF01627	PTHR28242	KOG4747		K14490	GO:000487
61	64	44	PF04535	PTHR11615,PTHR11615:SF120				
687	625	697	PF00010	PTHR16223,PTHR16223:SF38				GO:004698
403	419	370	PF00141	PTHR31235,PTHR3123		1.11.1.7	K00430	GO:005511
134	170	134	PF04045	PTHR12058	KOG2826		K05758	GO:003431
87	74	81	PF03195	PTHR31301,PTHR31301:SF2				
156	159	186	PF01740,P	PTHR11814,PTHR11814:SF85			K17471	GO:001602
17	17	10	PF00173,P	PTHR19370,PTHR1937		1.7.1.1	K10534	GO:005511
775	847	720	PF06886	PTHR14326				GO:006023
21	26	14	PF13905	PTHR13871	KOG2501	1.8.1.8		
86	70	90	PF00892	PTHR31218,PTHR31218:SF10				GO:001602
429	510	411	PF07719,P	PTHR11071,PTHR1107		5.2.1.8	K05864	GO:000645
97	80	69	PF13716	PTHR11106	KOG2633			
432	425	480	PF01419	PTHR23244,PTHR23244:SF249				GO:008002
171	198	106	PF12695	PTHR10992	KOG1454	3.1.1.23		
43	42	52	PF06200,P	PTHR33077,PTHR33077:SF6			K13464	
16	16	9	PF00582	PTHR31964,PTHR31964:SF55				GO:000695
78	93	67	PF00010	PTHR12565,PTHR12565:SF128				GO:004698
5302	4723	3546	PF14368	PTHR33122,PTHR33122:SF6				
398	383	410	PF04669	PTHR31444,PTHR3144		2.1.1.112	K18801	
833	928	788	PF04045	PTHR12058	KOG2826		K05758	GO:003431
97	70	61	PF00403	PTHR22814	KOG1603			GO:004687
0	0	0	PF00394,P	PTHR11709,PTHR1170		1.10.3.2	K05909	GO:005511
35	32	35	PF00010	PTHR13935,PTHR13935:SF53				GO:004698
292	279	328		PTHR32011,PTHR32011:SF2				
49	37	28	PF04535	PTHR11615,PTHR11615:SF113				
48	42	55	PF13637,P	PTHR24177,PTHR24177:SF19				

139	171	100	PF12710,P	PTHR15486,PTHR1548		2.3.1.198	K13508	GO:001674
24	25	12		PTHR33597,PTHR33597:SF2				
428	357	314	PF00141	PTHR31388,PTHR3138		1.11.1.7	K00430	GO:005511
884	989	593	PF06404	PTHR33285,PTHR33285:SF3				GO:000828
8	5	1	PF00646	PTHR31482,PTHR31482:SF2				GO:000551
289	335	296		PTHR16223,PTHR16223:SF41				
112	144	91	PF00407	PTHR31338,PTHR31338:SF16				GO:000960
17	13	16		PTHR34948,PTHR3494		3.6.1.3,3.6.1.25		
41	42	30	PF00249	PTHR10641	KOG0048		K09422	
22	26	32	PF05804,P	PTHR22849,PTHR2284		6.3.2.19		GO:001656
18	15	17	PF07731,P	PTHR11709,PTHR1170		1.10.3.2	K05909	GO:005511
148	159	124	PF00854	PTHR11654,PTHR11654:SF73				GO:001602
9	14	16	PF04564	PTHR23315,PTHR2331		6.3.2.19		GO:001656
113	136	168	PF12481	PTHR11772,PTHR11772:SF15				
3	1	0		PTHR23315,PTHR2331		6.3.2.19		
543	529	603	PF13639	PTHR14155,PTHR14155:SF83				GO:000827
38	54	38	PF13912	PTHR26374,PTHR26374:SF208				
173	168	180	PF06749	PTHR31769,PTHR31769:SF9				
3637	3424	4259	PF13632,P	PTHR13301,PTHR1330		2.4.1.12	K10999	
279	314	270	PF13768	PTHR10338,PTHR10338:SF114				
25	17	18	PF14009	PTHR33052,PTHR33052:SF23				
60	63	41	PF02309	PTHR31734,PTHR31734:SF38			K14484	GO:000635
133	119	163	PF00271,P	PTHR24075		3.6.4.13	K12854	GO:001678
826	882	845	PF14416,P	PTHR32285,PTHR32285:SF62				
52	77	67	PF03105	PTHR10783	KOG1161			
161	150	143	PF04669	PTHR31444,PTHR3144		2.1.1.112	K18801	
84	77	83						
97	130	123	PF00232	PTHR10353,PTHR1035		3.2.1.21	K05350	GO:000597
273	228	140	PF00141	PTHR31388,PTHR3138		1.11.1.7	K00430	GO:005511
243	250	246	PF13639	PTHR14155	KOG1493		K19041	GO:000827
25	33	26	PF00249	PTHR10641	KOG0048		K09422	
4	3	1	PF01501	PTHR11183	KOG1950	2.4.1.17	K00750	GO:001675
96	100	86	PF04720	PTHR31579,PTHR31579:SF17				
86	95	77	PF03106	PTHR31221,PTHR31221:SF37				GO:004356
10	6	7	PF01490	PTHR22950	KOG1303			
636	574	383	PF00657	PTHR22835,PTHR2283		3.1.1.3		GO:001678
1546	1558	1527	PF02458	PTHR31642,PTHR3164		2.3.1.133	K13065	GO:001674
158	153	154	PF14531	PTHR24349	KOG0032	2.7.11.1,4.1.1.49		
1014	1041	953	PF00651	PTHR24413,PTHR24413:SF43			K10523	GO:000551
44	46	34	PF00847	PTHR31729,PTHR31729:SF2			K09286	GO:000635
16	14	10	PF04564,P	PTHR27003	KOG1187	2.7.11.1		GO:001656
55	63	61		PTHR34670,PTHR34670:SF3				
92	87	68	PF03766,P	PTHR31775				
66	90	66		PTHR24078,PTHR24078:SF279				
62	65	66	PF00249	PTHR10641,PTHR10641:SF665				
73	75	84	PF07690	PTHR11654,PTHR11654:SF121			K14638	GO:005508
28	27	17	PF14368	PTHR33044,PTHR33044:SF34				
574	603	601	PF02365	PTHR31989,PTHR31989:SF39				GO:000635
2	1	1	PF00847	PTHR31985,PTHR31985:SF28				GO:000635
343	413	359	PF01650	PTHR12000,PTHR1200		3.4.22.34	K01369	GO:000823
5	6	7	PF03016	PTHR11062	KOG1021	2.4.2.41	K18789	
329	383	322	PF03763	PTHR31471,PTHR31471:SF14				
36	30	33	PF05558	PTHR38522,PTHR38522:SF2				GO:005171
24	15	21	PF00447	PTHR10015	KOG0627		K09419	GO:004356

81	59	73	PF14244,P	PTHR24177,PTHR24177:SF19				
76	83	87	PF00854	PTHR11654,PTHR11654:SF177				GO:001602
121	110	89	PF02519	PTHR31374,PTHR31374:SF10			K14488	GO:000973
217	278	332	PF00612,P	PTHR32295,PTHR32295:SF18				GO:000551
32	37	23	PF01764	PTHR21493,PTHR21493:SF124				GO:000662
27	29	20	PF13912	PTHR26374,PTHR26374:SF207				
368	209	196	PF01048	PTHR21234,PTHR2123		3.2.2.9		GO:000911
69	61	68	PF00657	PTHR14209	KOG3035	3.1.1.2		GO:001678
101	122	108	PF13962	PTHR24177,PTHR24177:SF19				
882	840	751	PF00412	PTHR24206	KOG1700		K09377	GO:000827
19	16	17						
361	355	419	PF00170	PTHR22952,PTHR22952:SF182				GO:004356
264	230	239	PF07876	PTHR33178,PTHR33178:SF2				
661	978	672	PF01486,P	PTHR11945	KOG0014			GO:000635
2	1	2	PF11883,P	PTHR27002	KOG1187	2.7.11.1		GO:000467
618	531	366	PF00462	PTHR10168	KOG1752	1.8.1.9	K03676	GO:004545
49	49	39	PF00005,P	PTHR19241,PTHR1924		3.6.3.25		GO:001688
21	13	14	PF00249	PTHR10641	KOG0048		K09422	
572	471	423	PF02458	PTHR31147,PTHR3114		2.3.1.162		GO:001674
134	146	164	PF13855	PTHR32093				GO:000551
56	63	36	PF02801,P	PTHR11712,PTHR1171		2.3.1.179		
90	62	64	PF04398	PTHR31676,PTHR31676:SF20				
425	330	381	PF04535	PTHR11615,PTHR11615:SF143				
833	892	873	PF14416,P	PTHR32285,PTHR32285:SF62				
59	77	71		PTHR33919,PTHR33919:SF2				
70	63	47	PF00005,P	PTHR19241	KOG0061			GO:001688
495	558	716		PTHR33739,PTHR33739:SF5				
70	111	106	PF08541,P	PTHR31561,PTHR3156		2.3.1.199	K15397	GO:001674
1738	1822	2153	PF04669	PTHR31444,PTHR31444:SF8				
293	335	251	PF00999	PTHR32468,PTHR32468:SF10				GO:005508
656	632	750	PF14531	PTHR24362	KOG0589	2.7.11.1	K08857	
65	80	61		PTHR35301,PTHR35301:SF1				
0	0	1	PF00141	PTHR31388,PTHR3138		1.11.1.7	K00430	GO:005511
63	60	55		PTHR10992,PTHR10992:SF740				
8	12	6	PF00249	PTHR31314				
7878	7374	7696	PF00067	PTHR24298	KOG0156	1.14.13.1	K00487	GO:005511
96	112	98	PF12796,P	PTHR24177,PTHR24177:SF11				
8	5	4	PF13499	PTHR10891	KOG0027		K13448	
575	532	513	PF00249	PTHR10641,PTHR10641:SF484			K09422	
816	780	865	PF01501	PTHR11183	KOG1950	2.4.1.17		GO:001675
13	11	11						
31	26	34	PF00067	PTHR24286,PTHR24286:SF76			K01723	GO:005511
20	11	0	PF02365	PTHR31744,PTHR31744:SF6				GO:000635
13	14	16	PF13912	PTHR26374				
36	42	13	PF13668	PTHR31694,PTHR31694:SF6				
21	17	9	PF14226,P	PTHR10209	KOG0143	1.14.11.9	K05278	GO:005511
70	79	42	PF13561	PTHR24322	KOG0725	1.3.1.9	K00208	
17	17	13	PF00067	PTHR24298,PTHR2429		1.14.13.89		GO:005511
340	362	380	PF03105	PTHR10783	KOG1161			
0	0	1	PF03227	PTHR13234	KOG3160		K08059	
1051	1089	1104	PF01501	PTHR32116,PTHR3211		2.4.1.43	K13648	GO:001675
1244	1257	1036	PF00031	PTHR11413,PTHR11413:SF56				GO:000486
110	103	74	PF00230	PTHR19139	KOG0223		K09872	GO:001602
7	7	2	PF00407	PTHR31907,PTHR31907:SF4				GO:000960

4	8	8	PF03195	PTHR31529,PTHR31529:SF2				
391	427	482	PF01490	PTHR22950	KOG1303		K13946	
1047	1246	1217	PF00394,P	PTHR11709,PTHR1170		1.10.3.2	K05909	GO:005511
70	46	58	PF08100,P	PTHR11746	KOG3178	2.1.1.128		GO:004698
207	227	177	PF13920	PTHR15315	KOG1039			
17	12	9	PF03106	PTHR31221,PTHR31221:SF7				GO:004356
6	7	8	PF13639	PTHR14155,PTHR14155:SF179			K19038	GO:000827
69	86	63	PF01490	PTHR22950	KOG1303		K15015	
135	140	135	PF00651,P	PTHR32370,PTHR32370:SF30				GO:000551
83	80	120	PF01490	PTHR22950	KOG1303			
20	11	16	PF00249	PTHR10641	KOG0048		K09422	
3	3	11	PF03168	PTHR31852,PTHR31852:SF1				
22	11	18	PF13921	PTHR10641	KOG0048		K09422	
595	663	606	PF04674	PTHR31279,PTHR31279:SF16				
10	19	17	PF07690	PTHR24064	KOG0255			GO:005508
685	728	745	PF00564	PTHR31066,PTHR31066:SF4				GO:000551
5	4	8		PTHR33388,PTHR33388:SF4				
810	720	429	PF00657	PTHR22835,PTHR2283		3.1.1.3		GO:001678
622	570	460		PTHR34670,PTHR34670:SF3				
69	73	61	PF00561	PTHR10992	KOG1454	3.7.1.9		
1335	1421	1581	PF03151	PTHR11132	KOG1441		K15285	
2	0	1	PF00484	PTHR11002	KOG1578	4.2.1.1	K01673	GO:000827
1	4	1						
7718	7589	7471	PF01490	PTHR22950	KOG1303			
421	478	377						
14	20	25	PF00010	PTHR12565	KOG4304		K12126	GO:004698
206	225	229	PF07714,P	PTHR27007	KOG1187	2.7.11.1		GO:000646
603	587	405	PF02458	PTHR31642,PTHR3164		2.3.1.188	K15400	GO:001674
27	16	23	PF04770	PTHR31948,PTHR31948:SF12				
7	6	5	PF03081	PTHR12542	KOG2344		K07195	GO:000688
163	132	139		PTHR31439				
75	60	64	PF07716	PTHR23334,PTHR23334:SF25				GO:004356
0	3	4		PTHR34355,PTHR3435		3.4.19.12		
1152	1301	1413	PF07839	PTHR33923,PTHR3392		2.7.11.17		GO:000551
3	8	6	PF07690	PTHR24064,PTHR24064:SF274			K08176	GO:005508
772	921	785	PF07795	PTHR33431,PTHR33431:SF3				
7	8	9	PF00067	PTHR24298	KOG0156			GO:005511
24	32	22	PF02365	PTHR31744,PTHR31744:SF3				GO:000635
1386	1426	1527	PF01650	PTHR12000,PTHR1200		3.4.22.34	K01369	GO:000823
9	10	7	PF03634	PTHR31072,PTHR31072:SF18				
8	5	6	PF10533,P	PTHR31282,PTHR31282:SF6				GO:004356
745	748	691	PF00046	PTHR24326	KOG0484			GO:000367
7	14	14	PF06955,P	PTHR31062,PTHR3106		2.4.1.207	K08235	GO:004804
15	14	11	PF00155	PTHR11751,PTHR1175		4.4.1.14	K01762	GO:003017
53	45	43	PF02365	PTHR31744,PTHR31744:SF7				GO:000635
2398	2583	2679	PF13347	PTHR19432,PTHR19432:SF38			K15378	
513	526	479	PF01490	PTHR22950	KOG1303		K15015	
2	4	4	PF00201	PTHR11926,PTHR1192		2.4.1.218	K08237	GO:001675
720	904	873	PF04674	PTHR31279,PTHR31279:SF3				
19	9	19	PF00122,P	PTHR24092,PTHR2409		3.6.3.1	K14802	GO:004687
1139	1392	1012		PTHR33306,PTHR33306:SF7				
45	59	27		PTHR34366,PTHR34366:SF2				
5	0	1	PF03016	PTHR11062	KOG1021			
6	7	6	PF14389,P	PTHR23054,PTHR23054:SF22				

5	6	5	PF00497,F	PTHR18966	KOG1052		K05387	GO:001602
20	17	23	PF14009	PTHR33148,PTHR33148:SF2				
5	2	0	PF03254	PTHR31889,PTHR3188		2.4.1.69	K13681	GO:004254
773	852	895	PF00394,F	PTHR11709,PTHR1170		1.10.3.2	K05909	GO:005511
184	214	175	PF07058	PTHR31246,PTHR31246:SF5				GO:000801
59	50	58		PTHR13780,PTHR13780:SF39				
0	0	0		PTHR23155,PTHR23155:SF497				
126	119	100	PF01061,F	PTHR19241,PTHR19241:SF180				GO:001602
3885	4772	4520	PF00759	PTHR22298,PTHR2229		3.2.1.4		GO:000597
0	0	1	PF08031,F	PTHR32448,PTHR3244		1.3.3.8		GO:005511
2	4	1	PF11883,F	PTHR27002	KOG1187	2.7.11.1		GO:000467
72	59	57	PF03083	PTHR10791	KOG1623		K15382	GO:001602
684	797	525	PF00544	PTHR31683,PTHR3168		4.2.2.2	K01728	
211	215	182	PF00141	PTHR31235		1.11.1.7	K00430	GO:005511
159	143	146	PF00249	PTHR10641	KOG0048		K09422	
1857	2015	1860	PF04043	PTHR31080,PTHR3108		3.1.1.11		GO:000485
289	339	264	PF04043,F	PTHR31707,PTHR3170		3.1.1.11		GO:000485
0	0	0	PF00067	PTHR24296	KOG0157			GO:005511
0	0	0						
14	17	21						
0	0	1	PF01501	PTHR11183,PTHR1118		2.4.1.123		GO:001675
5	7	2	PF00067	PTHR24298,PTHR2429		1.14.13.68		GO:005511
932	988	1109	PF13632	PTHR32044		2.4.1.32	K13680	
21	19	20	PF13912	PTHR26374,PTHR26374:SF208				
84	88	94	PF01740	PTHR11814,PTHR11814:SF103				GO:005508
9	10	0	PF00249	PTHR10641	KOG0048		K09422	
255	243	256	PF00249	PTHR10641	KOG0048		K09422	
6	8	11	PF01490	PTHR22950	KOG1303			
0	0	0		PTHR33320,PTHR33320:SF10				
593	633	650	PF05278	PTHR21704,PTHR21704:SF19				
0	2	2	PF08263	PTHR32093				
1	5	0	PF00847	PTHR31985,PTHR31985:SF28				GO:000635
24	14	14	PF00332	PTHR32227,PTHR3222		3.2.1.39		GO:000597
6	8	4	PF01453,F	PTHR27002	KOG1187	2.7.11.1		GO:000646
613	627	601	PF10604	PTHR33789,PTHR33789:SF5				
125	158	147		PTHR31342,PTHR31342:SF9				
181	200	157	PF03819	PTHR14552,PTHR1455		3.6.1.12	K16904	
5	1	1		PTHR34808,PTHR34808:SF2				
19	22	15	PF13833	PTHR10891	KOG0028			
9	3	5	PF02984,F	PTHR10177	KOG0656			GO:000563
264	258	184	PF00860	PTHR11119,PTHR11119:SF51				GO:005508
3	0	5	PF00249	PTHR10641	KOG0048		K09422	
370	385	381	PF13639	PTHR22937,PTHR22937:SF53				GO:000827
2	2	6	PF02887,F	PTHR11817,PTHR1181		2.7.1.40	K00873	GO:003095
586	579	644	PF03360	PTHR10896	KOG1476	2.4.2.24		GO:001602
1659	1668	1870	PF02183,F	PTHR24326	KOG0483			GO:004356
131	127	155	PF00005,F	PTHR19241	KOG0061	3.6.3.36		GO:001688
0	3	2	PF00232	PTHR10353,PTHR1035		3.2.1.21	K01188	GO:000597
16	12	19	PF13962	PTHR24177,PTHR24177:SF19				
48	44	48	PF03018	PTHR21495,PTHR21495:SF80				
200	251	187	PF08541,F	PTHR31561,PTHR3156		2.3.1.199	K15397	GO:001674
25	39	13	PF03514	PTHR31636,PTHR31636:SF19				
5	4	3		PTHR10641,PTHR10641:SF656			K09422	
5	4	5	PF02365	PTHR31719,PTHR31719:SF9				GO:000635

12	14	13	PF04515	PTHR12385	KOG1362			
145	159	130	PF00892	PTHR31218,PTHR31218:SF61				GO:001602
686	728	718	PF00069	PTHR13902,PTHR1390		2.7.11.1	K08867	GO:000646
428	503	406	PF13639,F	PTHR22763,PTHR2276		6.3.2.19	K11982	GO:000827
22	8	3	PF00234	PTHR33076,PTHR33076:SF4				
38	38	30	PF01471,F	PTHR10201,PTHR1020		3.4.24.23		GO:003101
225	289	298	PF03009	PTHR22958	KOG2421	3.1.4.46	K18696	GO:000808
188	215	167	PF00005,F	PTHR19241,PTHR19241:SF258				GO:001688
3	3	0	PF01556	PTHR24078,PTHR24078:SF266				
7	5	12	PF14543,F	PTHR13683	KOG1339	3.4.23.12		GO:000650
9	11	7						
18	13	16	PF00026	PTHR13683	KOG1339	3.4.23.12		GO:000650
7	6	5	PF00201	PTHR11926	KOG1192	2.4.1.271		GO:001675
9	1	2	PF00201	PTHR11926,PTHR11926:SF343				GO:001675
3	0	0	PF00005,F	PTHR24221,PTHR2422		3.6.3.44	K05658	GO:001688
5	2	2	PF13302	PTHR13256,PTHR1325		2.3.1.57		GO:000808
31	12	30	PF08263,F	PTHR27004	KOG0531,K	2.7.11.1		GO:000551
435	498	445	PF03105,F	PTHR10783,PTHR10783:SF53				GO:001602
12	9	17	PF13676,F	PTHR11017	KOG4658			GO:000716
14	17	10	PF00249	PTHR10641	KOG0048		K09422	
9	4	10	PF03169	PTHR31645,PTHR31645:SF1				GO:005508
1	0	0						
1	5	3						
4	3	14	PF00494	PTHR31480	KOG4411	2.5.1.32	K02291	GO:001674
16	8	8	PF11883,F	PTHR27002	KOG1187	2.7.11.1		GO:000467
1	2	0	PF00067	PTHR24298	KOG0156			GO:005511
1	4	2		PTHR33743,PTHR33743:SF5				
14	10	8	PF00504	PTHR14154,PTHR14154:SF5				
21	20	16	PF03195	PTHR31529,PTHR31529:SF6				
20	18	13	PF13639	PTHR14155			K10664	GO:000827
48	23	33	PF00450	PTHR11802,PTHR1180		3.4.16.6	K16297	GO:000650
18	9	18	PF00106	PTHR24320	KOG1208	1.1.1.330		GO:001649
9	6	7	PF00201	PTHR11926	KOG1192	2.4.1.324		GO:001675
3	3	5	PF14416,F	PTHR32285,PTHR32285:SF38				
16	13	8	PF00010	PTHR12565,PTHR12565:SF172				GO:004698
1	0	1	PF01344	PTHR24412,PTHR24412:SF230				GO:000551
10	15	11		PTHR33974,PTHR33974:SF2				
0	0	1	PF10354	PTHR11538,PTHR11538:SF42			K19307	
18	15	19	PF01477,F	PTHR11771,PTHR1177		1.13.11.5	K15718	GO:000551
2	4	3	PF00168,F	PTHR23180	KOG1030		K12486	GO:000551
2	2	2		PTHR37210,PTHR37210:SF2				
0	0	0	PF14226,F	PTHR10209	KOG0143		K06892	GO:005511
5	11	12	PF00854	PTHR11654	KOG1237		K14638	GO:001602
6	9	5	PF03372	PTHR11200	KOG0565	3.1.3.56		
6	7	5	PF14009	PTHR33148,PTHR33148:SF3				
7	14	7	PF13855,F	PTHR23155,PTHR23155:SF497				GO:000551
27	14	7	PF12708	PTHR31375,PTHR3137		3.2.1.15	K01184	
3	1	0	PF00201	PTHR11926,PTHR11926:SF343				GO:001675
6	7	10	PF00295	PTHR31375,PTHR3137		3.2.1.15		GO:000597
22	18	20	PF13193,F	PTHR24096,PTHR2409		6.2.1.34,	K01904	GO:000815
11	6	3	PF08100,F	PTHR11746	KOG3178			GO:004698
3	3	0	PF03405	PTHR31155,PTHR3115		1.14.19.2	K03921	GO:005511
18	20	6	PF00005,F	PTHR19241,PTHR1924		3.6.3.29		GO:001688
2	2	2		PTHR23155,PTHR23155:SF518				

182	198	195	PF01595	PTHR12064	KOG2118		K16302	
437	431	503	PF03900,P	PTHR11557	KOG2892	2.5.1.61	K01749	GO:003301
419	467	513	PF01762,P	PTHR11214	KOG2287	2.4.1.134		GO:001602
3440	3416	3950	PF03807,P	PTHR13812,PTHR1381		1.2.1.70	K02492	GO:005511
5083	4664	6224	PF00232	PTHR10353,PTHR1035		3.2.1.21	K01188	GO:000597
441	451	685	PF03330,P	PTHR31867				
279	260	329	PF00106	PTHR24322	KOG0725	1.3.1.9	K00208	GO:001649
52	66	40	PF12695	PTHR10655	KOG2112	3.1.1.5	K06130	
103	105	156	PF01851	PTHR10943				
290	280	376	PF03330,P	PTHR31867,PTHR31867:SF28				
571	628	773	PF13812,P	PTHR24015,PTHR24015:SF366				
2074	2130	2451		PTHR31469,PTHR31469:SF2				
53	45	69	PF02458	PTHR31625,PTHR3162		2.3.1.115		GO:001674
4389	4689	4371	PF06298	PTHR34790,PTHR34790:SF1			K02723	GO:003014
34	35	36						
699	809	731	PF07647	PTHR33915				GO:000551
245	233	264	PF00504	PTHR21649,PTHR21649:SF13			K08908	GO:001602
906	836	1221	PF00205,P	PTHR18968,PTHR1896		2.2.1.6	K01652	GO:003097
115	98	111	PF00249	PTHR10641	KOG0048		K09422	
742	798	755	PF01678	PTHR31689,PTHR3168		5.1.1.7	K01778	GO:000908
365	308	409		PTHR22835,PTHR2283		3.1.1.3		
32	25	29	PF02535	PTHR11040	KOG1558		K14709	GO:005508
4048	4005	3278	PF01157	PTHR20981	KOG1732		K02889	GO:000641
68	92	95	PF07883	PTHR31238,PTHR31238:SF41				
402	470	481	PF00582,P	PTHR27001	KOG1187	2.7.11.1		GO:000695
133	153	209	PF14416,P	PTHR32285,PTHR32285:SF14				
935	945	953	PF00550	PTHR20863,PTHR20863:SF28				
2081	2443	2835	PF14310,P	PTHR30620,PTHR3062		3.2.1.37		GO:000597
855	779	789	PF00268	PTHR23409		1.17.4.1	K10808	GO:005511
374	369	510	PF02492	PTHR13748,PTHR13748:SF43				
361	454	384	PF00080	PTHR10003	KOG0441	1.15.1.1	K04565	GO:005511
76	80	101	PF13410,P	PTHR11260	KOG0406	2.5.1.18	K00799	GO:000551
321	419	503	PF03470,P	PTHR21596,PTHR21596:SF12				GO:003104
437	487	472	PF00022	PTHR11937,PTHR11937:SF235			K11340	
338	321	438	PF13840,P	PTHR21499	KOG0455	1.1.1.3,2	K12524	GO:001659
166	198	184	PF07983	PTHR32227,PTHR32227:SF91				
66	44	45	PF03195	PTHR31529,PTHR31529:SF3				
1387	1356	1289	PF06298	PTHR34790,PTHR34790:SF1			K02723	GO:003014
657	722	814	PF01656,P	PTHR11564,PTHR1156		3.6.5.4	K03106	GO:000661
308	275	252	PF05421	PTHR36049,PTHR36049:SF3				
145	162	181		PTHR35410,PTHR35410:SF1				
2343	2208	2800	PF01764	PTHR21493	KOG4569	3.1.1.3		GO:000662
1284	1375	1438	PF00483	PTHR22572	KOG1322	2.7.7.27	K00975	GO:001677
952	1000	1104	PF03283	PTHR21562,PTHR21562:SF5				GO:001678
1698	1815	1564	PF05479	PTHR36814,PTHR36814:SF1			K02701	GO:004265
142	139	131	PF12680	PTHR33698,PTHR3369		5.3.3.1		
339	327	281	PF00332	PTHR32227,PTHR3222		3.2.1.39		GO:000597
2963	3352	3453	PF00012	PTHR19375,PTHR19375:SF232			K03283	
489	457	483	PF01765	PTHR20982	KOG4759		K02838	GO:000641
57	57	79		PTHR37911,PTHR37911:SF1				
416	291	466	PF00201	PTHR11926,PTHR1192		2.4.1.115		GO:001675
631	643	802	PF00173,P	PTHR19353	KOG4232	1.14.19.3	K13076	GO:000662
9994	9396	8693	PF00504	PTHR21649,PTHR21649:SF26			K08912	GO:001602
364	339	424	PF13174,P	PTHR26312,PTHR26312:SF70				GO:000551

1216	1174	1586	PF01699	PTHR31503,PTHR31503:SF1			K07300	GO:005508
2318	2410	3006	PF00400,F	PTHR19876,PTHR19876:SF6			K17302	GO:000551
324	341	363	PF07690	PTHR23500	KOG0254		K08145	GO:005508
165	203	229	PF03330,F	PTHR31692,PTHR31692:SF5				
4743	4834	4974	PF00004	PTHR32429	KOG0651			GO:000552
490	512	690	PF13632	PTHR13301,PTHR1330		2.4.1.12		
241	336	358	PF07731,F	PTHR11709,PTHR1170		1.10.3.2	K05909	GO:005511
231	264	444	PF00759	PTHR22298,PTHR2229		3.2.1.4		GO:000597
292	351	295						
2564	3041	3112	PF02518,F	PTHR11528,PTHR11528:SF49			K04079	GO:005108
75	64	124	PF03330,F	PTHR31867				
979	1019	1094	PF05368	PTHR14194	KOG1203			
260	230	282	PF01507	PTHR23293	KOG0189	1.8.4.10	K05907	GO:000815
244	247	344	PF05340	PTHR31659,PTHR31659:SF0				
426	463	557	PF05879	PTHR11649,PTHR11649:SF65				
229	289	274	PF00201	PTHR11926,PTHR1192		2.4.1.273		GO:001675
793	865	993	PF00106	PTHR24322	KOG0725	1.3.1.9	K00208	GO:001649
58	43	47						
2130	2111	2011	PF00646,F	PTHR24006			K03875	GO:000551
1343	1351	1331	PF00266	PTHR21152	KOG2862	2.6.1.51,	K00830	
1080	971	1048	PF06480,F	PTHR23076,PTHR23076:SF60			K03798	GO:001602
92	88	59	PF04770	PTHR31948,PTHR31948:SF16				
371	331	312	PF00572	PTHR11545	KOG3203		K02871	GO:000641
39	60	36	PF03641	PTHR31223,PTHR31223:SF19			K06966	
93	110	154	PF08263,F	PTHR27000	KOG1187	2.7.11.1		GO:000551
75	59	60	PF14686,F	PTHR32018		4.2.2.23	K18195	
63	48	103	PF00657	PTHR22835,PTHR2283		3.1.1.3		GO:001678
390	553	731	PF03810,F	PTHR11223			K14290	GO:000853
268	243	321	PF00067	PTHR24298	KOG0156			GO:005511
223	317	385	PF02704	PTHR23201,PTHR23201:SF8				
223	231	198	PF01789	PTHR31407,PTHR31407:SF4			K02717	GO:001989
3742	4094	4605	PF00012	PTHR19375,PTHR19375:SF228			K09490	
159	221	318	PF14383,F	PTHR31680,PTHR31680:SF4				
2136	2251	2735	PF00085,F	PTHR23293	KOG0189	1.8.4.10,	K05907	GO:004545
128	89	132	PF04043	PTHR31080,PTHR3108		3.1.1.11		GO:000485
8955	7929	8653	PF01593	PTHR10742,PTHR1074		1.5.3.14,1.5.3.15		GO:005511
972	979	1098	PF00390,F	PTHR23406,PTHR2340		1.1.1.40	K00029	GO:005511
2904	2788	2585	PF00504	PTHR21649,PTHR21649:SF28			K08917	GO:001602
287	289	320	PF00187,F	PTHR22595,PTHR22595:SF62				GO:000806
308	314	382	PF14577,F	PTHR33232				
68	63	69	PF13460	PTHR14194,PTHR14194:SF44				
2402	2356	2449	PF00070	PTHR22912,PTHR2291		1.8.1.9		GO:005511
160	183	187	PF06886	PTHR31358,PTHR31358:SF6				
82	73	93	PF00854	PTHR11654,PTHR11654:SF73				GO:001602
3606	3514	3793	PF03330,F	PTHR31867,PTHR31867:SF9				
796	806	629	PF01241	PTHR34195,PTHR34195:SF2			K02698	GO:001602
561	341	402	PF01370	PTHR10366,PTHR10366:SF375				GO:005066
1766	1801	2114	PF05193,F	PTHR11851,PTHR1185		1.10.2.2	K17732	
166	194	170	PF09585	PTHR34784				
660	685	720	PF06273	PTHR32091,PTHR32091:SF5				
272	236	264		PTHR35994				
1832	1766	1958	PF13288,F	PTHR30525,PTHR3052		1.1.1.267	K00099	GO:000551
171	212	221	PF04851,F	PTHR24031,PTHR2403		3.6.4.13	K17679	GO:001678
203	197	287	PF16499	PTHR11452	KOG2366	3.2.1.22		

1707	1664	1764	PF00892	PTHR31218,PTHR31218:SF29				GO:001602
560	546	639	PF00348	PTHR12001	KOG0776	2.5.1.29	K13789	GO:000829
317	260	320		PTHR33156,PTHR33156:SF10				
416	481	618	PF08031,F	PTHR32448,PTHR3244		1.3.3.8		GO:005511
53	68	71	PF07649,F	PTHR13871	KOG2501	1.8.1.8	K17609	GO:005511
1129	1251	1175	PF02453	PTHR10994	KOG1792			
95	69	105	PF08880,F	PTHR31602,PTHR31602:SF17				GO:000635
448	436	538	PF00450	PTHR11802,PTHR1180		3.4.16.6	K16297	GO:000650
708	846	780	PF00067	PTHR24298	KOG0156	1.14.13.2	K05280	GO:005511
26	38	48	PF04578,F	PTHR31325,PTHR31325:SF0				
2315	2815	3823	PF14547	PTHR31731				
1432	1303	1267	PF00389,F	PTHR10996	KOG0069	1.1.1.81,	K15893	GO:005511
613	597	619	PF01016	PTHR15893	KOG4600		K02899	GO:000641
946	951	970	PF08669,F	PTHR13847,PTHR1384		2.1.2.10	K00605	GO:000654
111	130	175	PF13041	PTHR24015,PTHR24015:SF572				
3547	3699	3741	PF00076	PTHR24012				GO:000367
404	477	555	PF05922,F	PTHR10795,PTHR1079		3.4.14.10		GO:000650
117	100	126	PF00231	PTHR11693	KOG1531	3.6.3.14	K02115	GO:004696
84	74	110	PF03018	PTHR21495,PTHR21495:SF59				
1574	1545	1811	PF10417,F	PTHR10681	KOG0852	1.11.1.15	K03386	GO:005511
475	526	720	PF00067	PTHR24298	KOG0156	1.14.13.88		GO:005511
98	84	89		PTHR37231,PTHR37231:SF1				
400	406	516	PF00657	PTHR22835,PTHR2283		3.1.1.3		GO:001678
4600	4606	4807	PF00121	PTHR21139	KOG1643	5.3.1.1	K01803	GO:000815
75	45	62	PF00583	PTHR23091,PTHR23091:SF239				GO:000808
305	364	462	PF08263,F	PTHR27000	KOG1187	2.7.11.1		GO:000646
212	222	192	PF00466	PTHR11560,PTHR11560:SF8			K02864	GO:004225
79	74	75	PF04755	PTHR31906,PTHR31906:SF11				
45	39	39	PF10664	PTHR36900,PTHR36900:SF1				GO:005511
607	742	836	PF01554	PTHR11206	KOG1347			GO:005508
98	114	118	PF04548,F	PTHR10903,PTHR1090		3.6.5.2		GO:000552
45	35	44	PF13812,F	PTHR24015,PTHR24015:SF736				
159	180	187	PF08659	PTHR24322	KOG0725		K11147	
1475	1433	1332	PF06596	PTHR34455,PTHR34455:SF1				GO:001602
227	254	333	PF00483	PTHR22572	KOG1322	2.7.7.27	K00975	GO:001677
5080	5736	5620	PF03141	PTHR10108,PTHR1010		2.1.1.157		GO:000816
1429	1330	1148	PF04398	PTHR31676,PTHR31676:SF10				
455	394	501	PF10184	PTHR36334,PTHR36334:SF1				
612	565	553	PF00125,F	PTHR23430	KOG1756		K11251	GO:000367
6542	5583	5692	PF13410,F	PTHR11260	KOG1422	1.8.5.1		GO:000551
62	81	74	PF00628,F	PTHR12505				GO:000551
676	611	717	PF00314	PTHR31048,PTHR31048:SF7				
2215	2400	2475	PF03144,F	PTHR23115,PTHR2311		3.6.5.3	K03231	GO:000552
1355	1394	1387		PTHR33597,PTHR33597:SF2				
167	151	171	PF04725	PTHR34369,PTHR34369:SF2			K03541	GO:004265
458	486	528	PF06814	PTHR21229,PTHR21229:SF15				GO:001602
87	114	122	PF02519	PTHR31929			K14488	GO:000973
101	83	119	PF08031,F	PTHR32448,PTHR3244		1.3.3.8		GO:005511
6051	6007	6719	PF03952,F	PTHR11902,PTHR1190		4.2.1.11	K01689	GO:000609
2565	2269	2594	PF01134	PTHR10617,PTHR1061		1.3.1.83	K10960	GO:005066
384	380	373	PF05368	PTHR14194	KOG1203	1.3.1.75	K19073	
3960	3896	3975	PF01789	PTHR31407,PTHR31407:SF6			K02717	GO:001989
750	877	823	PF00150	PTHR31451,PTHR3145		3.2.1.78	K19355	GO:000597
869	891	1115	PF08541,F	PTHR31561,PTHR3156		2.3.1.199	K15397	GO:001674

90	84	107	PF02458	PTHR31642,PTHR3164	2.3.1.99	K13065	GO:001674
707	841	916	PF07983	PTHR32227,PTHR32227:SF75			
413	442	406	PF01966	PTHR11845 KOG3197		K07023	
420	544	547	PF00271,F	PTHR24031 KOG0331	3.6.4.13		GO:000552
2362	2219	2424	PF08263	PTHR27000 KOG4579	3.5.1.98		
388	337	377	PF00380	PTHR21569 KOG1753		K02996	GO:000641
372	540	757	PF02140,F	PTHR23421,PTHR2342	3.2.1.23		GO:003024
33	35	38	PF00561	PTHR10992 KOG4178	3.3.2.10		
147	146	136		PTHR34687,PTHR34687:SF1			
1746	2058	2745	PF00544	PTHR31683,PTHR3168	4.2.2.2	K01728	
883	842	806	PF00333,F	PTHR13718 KOG0877		K02988	GO:000641
276	298	374	PF00026	PTHR13683 KOG1339	3.4.23.12		GO:000650
711	530	598		PTHR34686,PTHR34686:SF1			
129	139	190	PF08241,F	PTHR10108 KOG1269	2.1.1.41	K00559	GO:000816
16	25	19	PF00520	PTHR10217 KOG0498		K05391	GO:005508
282	361	350	PF01554	PTHR11206 KOG1347		K03327	GO:005508
236	257	310	PF00298,F	PTHR11661 KOG3257		K02867	GO:000641
175	198	271	PF14416,F	PTHR32285,PTHR32285:SF65			
1348	1350	1439	PF01073,F	PTHR11540	1.1.1.37	K00026	GO:005511
457	484	578	PF00408,F	PTHR22573,PTHR2257	5.4.2.2,5	K01835	GO:007170
362	339	373	PF07816	PTHR33095,PTHR33095:SF14			
11572	11391	9532	PF01789	PTHR31407,PTHR31407:SF6		K02717	GO:001989
139	96	91	PF07883	PTHR31238,PTHR31238:SF21			
954	970	1019		PTHR36391,PTHR36391:SF1			
940	1041	1067	PF01501	PTHR32116,PTHR3211	2.4.1.43	K13648	GO:001675
437	431	463	PF00831	PTHR10916 KOG3436		K02904	GO:000641
60	81	86		PTHR36359,PTHR36359:SF1			
78	78	106	PF05199,F	PTHR11552,PTHR11552:SF69		K15403	GO:005511
89	89	104	PF03171,F	PTHR10209 KOG0143			GO:005511
214	195	220	PF02163	PTHR31412,PTHR31412:SF0			GO:000650
307	242	289		PTHR35115,PTHR35115:SF1			
192	167	214	PF13671	PTHR11055 KOG0635	2.7.1.25	K00860	
383	396	386	PF00378	PTHR11941 KOG1684	3.1.2.4	K05605	GO:000815
126	152	119	PF00657	PTHR22835,PTHR2283	3.1.1.3		GO:001678
1194	1271	1504	PF00162	PTHR11406,PTHR1140	2.7.2.3	K00927	GO:000609
556	561	502	PF07722,F	PTHR11405,PTHR1140	6.3.5.5		GO:001678
52	50	49	PF13181	PTHR36326,PTHR36326:SF1			GO:000551
1378	1484	2210	PF13632,F	PTHR13301,PTHR1330	2.4.1.12	K10999	
541	592	542	PF00076	PTHR24012 KOG0131		K13126	GO:000367
727	851	954	PF00118	PTHR11353,PTHR11353:SF80		K04077	GO:000552
403	424	599	PF13632,F	PTHR13301,PTHR1330	2.4.1.12	K10999	
152	98	142		PTHR33210,PTHR33210:SF6			
1456	1446	1567	PF00285	PTHR11739,PTHR1173	2.3.3.16,	K01647	GO:004691
282	357	371	PF04043	PTHR31707,PTHR31707:SF43			GO:000485
38	60	55	PF00432	PTHR11764,PTHR1176	5.4.99.8		GO:000382
3936	4107	3935	PF03719,F	PTHR13718 KOG0877		K02981	GO:000641
959	983	1147	PF02140,F	PTHR23421,PTHR2342	3.2.1.23		GO:003024
232	181	283	PF00657	PTHR22835,PTHR2283	3.1.1.3		GO:001678
79	88	107	PF07731,F	PTHR11709,PTHR1170	1.10.3.2	K05909	GO:005511
451	430	426	PF00111	PTHR19370,PTHR19370:SF109			GO:005153
1040	1206	1296	PF00118	PTHR11353,PTHR11353:SF80		K04077	GO:000552
97	116	186	PF01964	PTHR30557,PTHR3055	4.1.99.17	K03147	GO:005153
683	656	777	PF02309	PTHR31734,PTHR31734:SF30		K14484	GO:000635
1385	1179	1054	PF14368	PTHR33122,PTHR33122:SF8			

2856	3073	3487	PF06955,F	PTHR31062,PTHR3106		2.4.1.207	K08235	GO:004804
261	275	244	PF01132,F	PTHR30053,PTHR30053:SF3			K02356	GO:000641
48	36	44	PF00504	PTHR21649,PTHR21649:SF7			K08907	GO:001602
1755	1927	2155	PF01073	PTHR10366	KOG1429	4.1.1.35	K12449	GO:005511
1690	1787	2325	PF13632	PTHR32044,PTHR3204		2.4.1.32	K13680	
236	257	240	PF00070	PTHR22912		1.6.5.9	K17871	GO:005511
657	895	1092	PF02469	PTHR32382,PTHR32382:SF13				
1762	1309	1756	PF00067	PTHR24298	KOG0156	1.14.13.21		GO:005511
1032	1144	1138	PF00180	PTHR11835,PTHR1183		1.1.1.85	K00052	GO:005511
855	800	738	PF00970,F	PTHR19370,PTHR1937		1.6.2.2	K00326	GO:005511
312	257	240		PTHR34283,PTHR34283:SF1				
495	514	606	PF01118,F	PTHR10174	KOG4777	1.2.1.11	K00133	GO:005511
176	172	185	PF00450	PTHR11802,PTHR1180		3.4.16.6	K16297	GO:000650
236	231	248	PF13193,F	PTHR24095	KOG1177	6.2.1.26	K14760	GO:000815
548	602	650	PF00575	PTHR10724,PTHR10724:SF7			K02945	GO:000367
448	513	528	PF00450	PTHR11802	KOG1282	3.4.16.5	K16298	GO:000650
196	222	213	PF01041	PTHR11751	KOG0257			
254	227	303		PTHR35744,PTHR35744:SF1				
131	135	160	PF12068					
2205	2071	1876	PF14159	PTHR33222,PTHR33222:SF4				
60	79	64	PF01734	PTHR32176	KOG0513	3.1.1.26		GO:000662
140	127	145	PF08241	PTHR10108	KOG1269			GO:000816
67	83	109	PF08144	PTHR13389	KOG2050		K14844	GO:000372
3718	4139	3989	PF07991,F	PTHR21371,PTHR2137		1.1.1.86	K00053	GO:005511
95	96	173	PF00664,F	PTHR24223,PTHR2422		3.6.3.44		GO:005508
73	83	107	PF00122,F	PTHR24092,PTHR2409		3.6.3.1	K14802	GO:004687
971	1100	1182	PF06026	PTHR11934	KOG3075	5.3.1.6	K01807	GO:000905
67	67	69	PF00316	PTHR11556,PTHR1155		3.1.3.11	K03841	GO:004257
114	98	152	PF02225,F	PTHR10795,PTHR1079		3.4.21.25		GO:000650
588	649	741	PF14551,F	PTHR11630		3.6.4.12	K02541	GO:000626
798	882	975	PF12708	PTHR31375,PTHR3137		3.2.1.15		
309	390	433	PF09739	PTHR13489,PTHR13489:SF0				
4574	4387	3938	PF02531	PTHR31982,PTHR31982:SF4			K02692	GO:001597
638	810	856	PF14497,F	PTHR11260	KOG1627		K03233	GO:000551
208	225	234	PF14310,F	PTHR30620,PTHR3062		3.2.1.55,3.2.1.37		GO:000597
539	567	779	PF00657	PTHR22835,PTHR2283		3.2.1.51		GO:001678
369	467	573	PF01095	PTHR31321,PTHR3132		3.1.1.11	K01051	GO:004254
76	69	64	PF03107,F	PTHR13871,PTHR13871:SF51				GO:005511
193	214	179	PF00595,F	PTHR32060,PTHR3206		3.4.21.102		GO:000551
293	236	287	PF01554	PTHR11206	KOG1347		K03327	GO:005508
91	87	100	PF04770	PTHR31948,PTHR31948:SF18				
805	860	973	PF01625	PTHR10173	KOG1635	1.8.4.11	K07304	GO:005511
250	234	267	PF12049	PTHR33102,PTHR33102:SF2				
144	133	182	PF04116,F	PTHR11863,PTHR11863:SF41			K15404	GO:005511
134	103	112	PF07690	PTHR23500	KOG0254			GO:005508
73	111	115	PF00170	PTHR13301,PTHR13301:SF52				GO:004356
889	958	973	PF00160	PTHR11071	KOG0880	5.2.1.8	K03768	GO:000645
892	879	956	PF00281,F	PTHR11994	KOG0398		K02931	
221	240	241	PF00046,F	PTHR24326	KOG0483		K09338	GO:000367
99	100	113		PTHR33312,PTHR33312:SF5				
11301	11052	9564		PTHR33403,PTHR33403:SF1			K18635	
1561	1465	1433	PF03951,F	PTHR20852,PTHR2085		6.3.1.2	K01915	GO:000680
8637	9422	10192	PF03144,F	PTHR23115,PTHR2311		3.6.5.3	K03231	GO:000552
281	269	358	PF00400	PTHR22844,PTHR2284		2.7.11.7		GO:000551

195	282	243	PF15699	PTHR33669,PTHR33669:SF2				
6076	5975	5319	PF00504	PTHR21649,PTHR21649:SF7			K08907	GO:001602
289	315	279	PF01459	PTHR10802	KOG3296		K11518	GO:005508
67	42	57	PF04720	PTHR31579,PTHR31579:SF10				
755	890	1006	PF02518,P	PTHR11528,PTHR11528:SF41			K09487	GO:005108
255	294	267		PTHR11716,PTHR1171		3.1.1.4	K01047	GO:001604
551	505	528	PF03171,P	PTHR10209	KOG0143	1.14.11.9		GO:005511
67	103	96		PTHR31288,PTHR31288:SF8				
282	239	225	PF13326	PTHR34041,PTHR34041:SF1			K08902	GO:001020
747	695	711	PF00282	PTHR11999	KOG0629	4.1.1.22	K01590	GO:003017
111	113	127	PF06418	PTHR11550,PTHR1155		6.3.4.2		GO:000622
77	70	81		PTHR35697,PTHR35697:SF1				
444	372	348	PF00111	PTHR19370,PTHR19370:SF120			K02639	GO:005153
1118	1108	1244	PF00349,P	PTHR19443,PTHR1944		2.7.1.1	K00844	GO:001677
361	349	348	PF14432,P	PTHR24015,PTHR24015:SF552				
208	222	261	PF12708	PTHR31375,PTHR3137		3.2.1.15	K01213	
214	210	238	PF05577	PTHR11010,PTHR1101		3.4.16.2	K01285	GO:000823
717	568	636	PF00132,P	PTHR23416	KOG4750	2.3.1.30	K00640	GO:000900
170	258	403	PF00575	PTHR11741,PTHR11741:SF3			K02357	GO:000367
803	667	1010	PF05922,P	PTHR10795,PTHR1079		3.4.14.10		GO:000650
2921	3004	2877	PF02672,P	PTHR10836	KOG0657	1.2.1.13	K05298	GO:005511
90	93	94	PF01263	PTHR10091	KOG1604	5.1.3.3	K01785	GO:001685
253	267	308	PF03129,P	PTHR11451,PTHR1145		6.1.1.15	K01881	GO:000643
1297	1298	1442	PF02518,P	PTHR11528,PTHR11528:SF41			K09487	GO:005108
1828	1800	2110	PF00984,P	PTHR11374,PTHR1137		1.1.1.22	K00012	GO:005511
1221	1186	1161	PF00347	PTHR11655	KOG3254		K02933	GO:001984
394	471	389	PF00076	PTHR24012	KOG0131			GO:000367
265	274	280	PF01535,P	PTHR24015,PTHR24015:SF579				
314	295	369	PF16198,P	PTHR13767	KOG2529	5.4.99.25	K03177	GO:000639
438	440	509	PF03765,P	PTHR23324	KOG1471			
141	151	163	PF00230	PTHR19139	KOG0223		K09874	GO:001602
5477	5032	5222	PF00504	PTHR21649,PTHR21649:SF4			K08910	GO:001602
56	59	72	PF07498	PTHR34449,PTHR34449:SF1				GO:000635
185	175	221	PF08100,P	PTHR11746	KOG3178			GO:004698
445	415	562		PTHR33621,PTHR33621:SF2				
164	209	208	PF01336,P	PTHR22594,PTHR2259		6.1.1.12	K01876	GO:000367
2383	2543	2222	PF07885	PTHR11003,PTHR11003:SF135			K05389	
441	516	360	PF13414	PTHR36326,PTHR36326:SF1				
28	34	31	PF14686,P	PTHR32018,PTHR3201		4.2.2.23	K18195	
54	51	58	PF02365	PTHR31989,PTHR31989:SF6				GO:000635
80	89	59	PF00111	PTHR19370,PTHR19370:SF127			K02639	GO:005153
84	48	97	PF00232	PTHR10353		3.2.1.21,	K01188	GO:000597
158	197	193	PF04536	PTHR30373,PTHR3037		3.1.3.2		
1115	1210	2010	PF15044,P	PTHR12601,PTHR12601:SF18			K03255	
1633	1504	2074	PF03144,P	PTHR23115,PTHR2311		3.6.5.3	K02358	GO:000552
273	211	293	PF00067	PTHR24298	KOG0156		K00517	GO:005511
630	575	557	PF01370	PTHR10366,PTHR10366:SF278				GO:005066
24957	24674	22182	PF00504	PTHR21649,PTHR21649:SF8			K08913	GO:001602
643	753	653	PF04043	PTHR31080,PTHR3108		3.1.1.11		GO:000485
10478	10556	8635	PF02507	PTHR34939,PTHR3493		1.97.1.12	K02694	GO:001597
1274	910	1282	PF01370	PTHR32487,PTHR3248		1.3.1.3		GO:005066
500	394	553	PF00560,P	PTHR27000	KOG4579	3.5.1.98		GO:000551
5028	4734	4574	PF00504	PTHR21649,PTHR21649:SF11			K08914	GO:001602
74	79	83	PF14215	PTHR13902,PTHR13902:SF64				

87	80	72	PF01095	PTHR31321,PTHR3132		3.1.1.11		GO:004254
398	331	356	PF00155	PTHR11808	KOG0053	4.4.1.11	K01761	GO:003017
92	60	79						
68	86	114	PF07883	PTHR31238,PTHR31238:SF21				
191	215	220	PF00696	PTHR23342	KOG1154	2.7.2.8	K00930	
669	674	740	PF05761	PTHR12103	KOG2469	3.1.3.5		
206	242	252	PF11891	PTHR31620,PTHR31620:SF9				
36	21	36	PF03171,F	PTHR10209	KOG0143	4.2.3.19		GO:005511
250	308	307	PF00464	PTHR11680,PTHR1168		2.1.2.1	K00600	GO:001674
36	48	50	PF00320	PTHR10071,PTHR10071:SF197				GO:004356
859	818	905	PF00487,F	PTHR32100,PTHR3210		1.14.99.3	K10257	GO:000662
208	226	204	PF03435	PTHR12286,PTHR1228		1.5.1.43		GO:005511
785	803	876	PF00132	PTHR13061,PTHR1306		1.6.5.3		
909	788	862	PF00829	PTHR21349	KOG1686		K02888	GO:000584
37	28	29	PF04043	PTHR31080,PTHR3108		3.1.1.11		GO:000485
1083	1117	965	PF00366	PTHR10744	KOG1740		K02961	GO:000641
456	450	407		PTHR33156,PTHR33156:SF7				
1047	1153	999	PF00132,F	PTHR22572,PTHR22572		2.7.7.22,	K00966	
1295	1447	1425	PF00291	PTHR10314,PTHR1031		4.2.1.20	K01696	
374	448	516	PF03953,F	PTHR11588,PTHR1158		2.4.2.1	K07375	GO:000392
560	523	662	PF02458	PTHR31896,PTHR3189		2.3.1.99		GO:001674
299	262	489						
47	36	67						
860	960	1070	PF00571,F	PTHR11911,PTHR1191		5.3.1.13	K06041	GO:003024
259	265	372	PF03181	PTHR31458,PTHR31458:SF3				
1322	1558	1642	PF16940	PTHR34935				
7915	7783	7624	PF00504	PTHR21649,PTHR21649:SF6			K08915	GO:001602
14	32	22	PF03214	PTHR31903,PTHR3190		5.4.99.30		GO:003024
36	40	47		PTHR34463				
532	615	822	PF05303,F	PTHR12601,PTHR12601:SF6				
78	67	87	PF00581	PTHR12231,PTHR12231:SF150				
1035	1078	1049	PF00467	PTHR12903	KOG1708		K02895	GO:000641
230	282	259	PF00380	PTHR21569	KOG1753		K02996	GO:000641
193	193	157	PF01063	PTHR11825	KOG0975	2.6.1.88,	K00826	GO:000815
121	128	202		PTHR31344,PTHR31344:SF4				
158	168	197	PF00651,F	PTHR32370,PTHR32370:SF37				GO:000551
738	767	884	PF01053	PTHR11808,PTHR1180		4.4.1.11		GO:003017
452	458	499	PF00856,F	PTHR13271	KOG1337	2.1.1.127		GO:000551
266	288	355	PF00696	PTHR21499,PTHR2149		2.7.2.4	K00928	
1377	1374	1222	PF00155	PTHR11879,PTHR1187		2.6.1.1	K00811	GO:003017
1128	988	1007	PF01241	PTHR34195,PTHR34195:SF1			K08905	GO:001602
2920	2967	2820	PF00274	PTHR11627,PTHR1162		4.1.2.13	K01623	GO:000609
1088	1089	1145	PF00291	PTHR10314	KOG1252	2.5.1.47	K01738	
289	221	332		PTHR33083,PTHR33083:SF5				
497	522	676	PF13802,F	PTHR22762,PTHR2276		3.2.1.20	K01187	GO:000597
295	280	271	PF05922,F	PTHR10795,PTHR1079		3.4.14.10		GO:000650
904	747	800	PF00081,F	PTHR11404	KOG0876	1.15.1.1	K04564	GO:005511
575	654	721	PF00450	PTHR11802	KOG1283	3.4.16.5	K09646	GO:000650
117	193	190		PTHR36748				
204	208	232	PF00861	PTHR12899			K02881	GO:000641
166	220	237	PF02704	PTHR23201,PTHR23201:SF4				
161	174	179	PF00155	PTHR11879,PTHR1187		2.6.1.1	K00811	GO:003017
156	149	163	PF00425	PTHR11236	KOG1223	5.4.4.2	K02552	GO:000905
571	578	573		PTHR35753,PTHR35753:SF1				

1127	1123	1087	PF00297	PTHR11229	KOG3141		K02906	GO:000641
462	481	484	PF00291	PTHR10314,PTHR1031		4.2.1.20	K01696	
549	632	847	PF00026	PTHR13683	KOG1339	3.4.23.12		GO:000650
210	245	220	PF00013	PTHR10288	KOG2190		K13162	GO:000372
280	326	290	PF12327,F	PTHR30314,PTHR3031		3.6.5.6	K03531	GO:000392
2564	2257	2460	PF02812,F	PTHR11606,PTHR1160		1.4.1.3	K00261	GO:005511
66	56	73	PF13410,F	PTHR32419	KOG2903		K07393	GO:000551
19	26	41	PF07731,F	PTHR11709,PTHR1170		1.10.3.2	K05909	GO:005511
1155	1089	1090	PF01564	PTHR11558	KOG1562	2.5.1.16	K00797	
92	102	140	PF01208	PTHR21091,PTHR2109		4.1.1.37	K01599	GO:000677
868	844	904	PF13489,F	PTHR10108	KOG1269	2.1.1.103	K05929	GO:000816
699	609	577	PF01625	PTHR10173	KOG1635	1.8.4.11	K07304	GO:005511
7964	9047	10971	PF02518,F	PTHR11528,PTHR11528:SF45			K09487	GO:005108
87	84	121	PF02861,F	PTHR11638,PTHR11638:SF116			K03695	GO:001953
331	352	387	PF00814	PTHR11735,PTHR1173		2.6.99.4	K01409	
194	189	225	PF00254	PTHR10516	KOG0552	5.2.1.8		GO:000645
1854	1840	1560		PTHR36389,PTHR36389:SF1				
90	75	77	PF07883	PTHR31238,PTHR31238:SF39				
113	148	123	PF08449	PTHR10778	KOG1581		K15275	GO:005508
4024	4157	3833		PTHR36311,PTHR3631		1.97.1.12	K14332	
85	110	114	PF01501	PTHR32116,PTHR3211		2.4.1.43	K13648	GO:001675
215	207	224	PF05368	PTHR14194	KOG1203			
1796	1959	1961	PF13473	PTHR34192,PTHR34192:SF2			K02638	
318	310	300		PTHR36793,PTHR36793:SF1				
290	263	331		PTHR35742,PTHR35742:SF1				
264	169	283	PF07859	PTHR23024	KOG1515	4.2.1.105,3.1.1.1		GO:001678
827	847	897	PF00175	PTHR19370	KOG0534	1.12.98.4		GO:005511
115	79	75	PF00314	PTHR31048,PTHR31048:SF36				
544	569	553	PF13847	PTHR10108,PTHR10108:SF791				
1122	997	970	PF01250	PTHR21011,PTHR21011:SF1			K02990	GO:001984
2540	2041	2976	PF00067	PTHR24298,PTHR2429		1.14.13.21		GO:005511
296	317	676	PF01190	PTHR23201				
63	45	77	PF00230	PTHR19139	KOG0223		K09872	GO:001602
118	60	111		PTHR33210,PTHR33210:SF6				
213	278	266		PTHR36398,PTHR36398:SF1				
226	274	345	PF07766	PTHR14009	KOG1043			
295	248	373	PF08534	PTHR10681	KOG0855	1.11.1.15	K03564	GO:001649
85	106	243	PF08263	PTHR32093	KOG4579			
128	96	153	PF00396,F	PTHR12411	KOG1542	3.4.22.14		GO:000823
76	82	83	PF11883,F	PTHR27002	KOG1187	2.7.11.1		GO:000467
443	463	421	PF00248	PTHR11732	KOG1575	1.1.1.65		
13	16	10		PTHR35476,PTHR35476:SF2				
79	97	96		PTHR36014,PTHR36014:SF1				
27	26	24	PF04690	PTHR31675,PTHR31675:SF6				GO:000727
26	34	33	PF01370,F	PTHR11011	KOG1221	1.2.1.84	K13356	GO:005066
1658	1713	1870		PTHR11177,PTHR11177:SF167			K17525	
250	280	411	PF00005,F	PTHR24221,PTHR2422		3.6.3.44	K05658	GO:001688
115	121	125	PF13920,F	PTHR12183	KOG4172			
785	684	770	PF00828	PTHR12934	KOG0846		K02876	GO:001593
98	109	107		PTHR10168,PTHR10168:SF94				
345	388	384	PF07137	PTHR33970,PTHR3397		1.10.99.3	K09839	GO:005511
151	156	178	PF00202	PTHR11986	KOG1401	2.6.1.11	K00818	GO:003017
160	155	188	PF13364,F	PTHR23421,PTHR2342		3.2.1.23		GO:003024
6212	6210	7204	PF00162	PTHR11406,PTHR1140		2.7.2.3	K00927	GO:000609

111	149	159	PF00657	PTHR22835,PTHR2283		3.1.1.3		GO:001678
873	1007	1148	PF01734	PTHR32241	KOG0513			GO:000662
674	602	613	PF13414	PTHR36326,PTHR36326:SF1				
2078	1977	1934	PF00175	PTHR19384,PTHR1938		1.18.1.2,	K02641	GO:005511
1677	1806	1851	PF07991,F	PTHR10996,PTHR1099		1.1.1.95	K00058	GO:001659
469	422	516	PF00390,F	PTHR23406,PTHR2340		1.1.1.40	K00029	GO:005511
192	197	270		PTHR33971,PTHR33971:SF1				
1990	2028	2000	PF16363	PTHR10366,PTHR1036		4.2.1.47	K01711	
5168	5181	4922	PF00504	PTHR21649,PTHR21649:SF11			K08914	GO:001602
469	523	514	PF00067	PTHR24298	KOG0156			GO:005511
141	170	208	PF07731,F	PTHR11709,PTHR1170		1.10.3.2	K05909	GO:005511
58	78	96	PF07517	PTHR30612,PTHR30612:SF4				GO:001703
675	817	762	PF00150	PTHR31451,PTHR3145		3.2.1.78	K19355	GO:000597
369	327	396	PF00230	PTHR19139	KOG0223		K09873	GO:001602
297	215	348	PF13193,F	PTHR24095,PTHR2409		6.2.1.1		GO:000815
1792	1728	1661	PF16320,PF00542		KOG1715		K02935	GO:000641
530	574	540	PF01612	PTHR13620	KOG4373	3.6.4.12		GO:000840
179	186	143	PF14497,F	PTHR12782	KOG3029	5.3.99.3	K05309	GO:000551
533	503	558	PF01196	PTHR14413	KOG3280		K02879	GO:000641
520	561	575	PF14870	PTHR32010,PTHR32010:SF6				
2406	2442	2392	PF00106	PTHR24322	KOG1208	1.3.1.33,	K00218	GO:001649
38	29	30	PF03016	PTHR11062	KOG1021	2.4.2.41		
2300	2308	2089	PF14159	PTHR33222,PTHR33222:SF4				
51	61	63	PF03552	PTHR13301,PTHR1330		2.4.1.12		GO:003024
2075	1950	1981	PF00101	PTHR31262		4.1.1.39	K01602	
528	551	677	PF00390,F	PTHR23406,PTHR2340		1.1.1.39	K00028	GO:005511
415	354	336		PTHR34678,PTHR34678:SF1			K19034	
651	632	589	PF00334	PTHR11349	KOG0888	2.7.4.6	K00940	GO:000624
87	110	146	PF05922,F	PTHR10795,PTHR1079		3.4.21.25		GO:000650
63	76	61	PF00168,F	PTHR10336,PTHR1033		3.1.4.11	K05857	GO:000551
1673	1504	1933	PF14306,F	PTHR11055,PTHR1105		2.7.7.4	K13811	GO:000478
704	635	693	PF16113	PTHR11941	KOG1680	4.1.3.36	K01661	
348	359	336	PF00542	PTHR24089	KOG1715		K02935	GO:000641
95	113	167	PF01225,F	PTHR23135,PTHR2313		6.3.2.37,6.3.2.7		GO:000905
492	567	562	PF14551,F	PTHR11630,PTHR1163		3.6.4.12	K02212	GO:000626
1174	1200	1878	PF07731,F	PTHR11709,PTHR1170		1.10.3.3		GO:005511
8893	9464	10702	PF03283	PTHR21562,PTHR21562:SF4				GO:001678
1145	1258	1578	PF09261,F	PTHR11607,PTHR1160		3.2.1.24	K01191	GO:000827
805	916	749		PTHR34946				
509	566	662	PF08156,F	PTHR10894,PTHR10894:SF0			K14564	
541	533	511		PTHR37182				
594	547	907	PF03181	PTHR31236,PTHR31236:SF2				
1348	1135	1241	PF13410,F	PTHR11260	KOG0406	2.5.1.18	K00799	GO:000551
345	298	351						
1575	1149	1227	PF04725	PTHR34369,PTHR34369:SF2			K03541	GO:004265
468	482	442	PF04852	PTHR31165,PTHR31165:SF10				
81	63	78	PF00168,F	PTHR10067,PTHR1006		4.1.1.65	K01613	GO:000551
52	64	90	PF03352	PTHR31116,PTHR3111		3.2.2.20	K01246	GO:000872
527	578	511	PF13225	PTHR33591,PTHR3359		5.2.1.14		
376	371	296	PF03195	PTHR31304,PTHR31304:SF5				
970	936	888		PTHR36315,PTHR36315:SF1				
465	574	461	PF12708	PTHR31339,PTHR31339:SF7				
1195	1345	1386	PF00202	PTHR11986,PTHR1198		5.4.3.8	K01845	GO:003017
1696	1815	2417	PF01565	PTHR10801,PTHR1080		1.3.1.72	K09828	GO:005511

156	94	156	PF01477,F	PTHR11771,PTHR1177		1.13.11.1	K00454	GO:000551
1250	1335	1411	PF00171	PTHR11699,PTHR1169		1.2.1.9	K00131	GO:005511
199	244	249	PF08246,F	PTHR12411	KOG1543	3.4.22.6	K16290	GO:000823
173	134	197	PF00160	PTHR11071,PTHR1107		5.2.1.8		GO:000645
1770	1777	1936	PF07649,F	PTHR13871	KOG2501	1.8.1.8	K17609	GO:005511
1638	1800	1754	PF14204,F	PTHR23410	KOG0875		K02932	GO:000641
422	448	582	PF00010	PTHR12565,PTHR12565:SF149				GO:004698
2981	2309	2849	PF01370	PTHR32487,PTHR3248		1.3.1.3		GO:005066
349	394	495	PF16499	PTHR11452	KOG2366	3.2.1.22	K07407	
616	413	624	PF02458	PTHR31642,PTHR3164		2.3.1.196		GO:001674
506	501	570	PF00160	PTHR11071	KOG0884	5.2.1.8		GO:000645
405	457	523	PF00067	PTHR24298	KOG0156			GO:005511
115	109	127	PF00298,F	PTHR11661	KOG3257			GO:000641
28	27	24	PF00067	PTHR24298	KOG0156		K00517	GO:005511
332	410	485	PF03352	PTHR31116,PTHR3111		3.2.2.20	K01246	GO:000872
279	311	475	PF07731,F	PTHR11709,PTHR1170		1.10.3.3		GO:005511
2034	2205	2293	PF03953,F	PTHR11588,PTHR1158		2.4.2.1	K07375	GO:000392
2236	2322	2493	PF00390,F	PTHR23406,PTHR2340		1.1.1.39	K00028	GO:005511
2024	1847	2105	PF02502	PTHR30345,PTHR3034		5.3.1.6		GO:001685
463	468	427	PF06596	PTHR34455,PTHR34455:SF1				GO:001602
290	299	492	PF08263,F	PTHR27000	KOG1187	2.7.11.1		GO:000551
86	97	95		PTHR35697,PTHR35697:SF1				
126	160	180	PF00067	PTHR24286	KOG0157	1.14.13.1	K12637	GO:005511
307	313	312	PF00805	PTHR14136				
1432	1320	1589	PF00067	PTHR24298	KOG0156	1.14.13.21		GO:005511
1589	1501	1791	PF01070	PTHR10578,PTHR1057		1.1.3.15	K11517	GO:001649
929	978	1160	PF01566	PTHR11706,PTHR11706:SF33			K03322	GO:001602
532	621	690	PF04398	PTHR31676,PTHR31676:SF8				
210	244	292		PTHR31681,PTHR31681:SF4				
85	102	132	PF05664	PTHR31280,PTHR31280:SF1				
491	441	461	PF00201	PTHR11926	KOG1192			GO:001675
32	40	57	PF12142,F	PTHR11474,PTHR1147		1.10.3.1	K00422	GO:005511
472	466	510	PF13301	PTHR34679,PTHR34679:SF2				
1860	1672	1483	PF01597	PTHR11715	KOG3373		K02437	
202	221	304	PF16363	PTHR10366,PTHR1036		4.2.1.47	K01711	
1022	1022	954	PF05757	PTHR33399,PTHR33399:SF3			K08901	GO:001989
8	23	23	PF00544,F	PTHR31683,PTHR3168		4.2.2.2	K01728	GO:003057
2955	3218	3435	PF00118	PTHR11353,PTHR11353:SF8			K04077	GO:000552
821	786	939	PF04043,F	PTHR31707,PTHR3170		3.1.1.11	K01051	GO:000485
750	728	726	PF01680	PTHR31829		4.3.3.6	K06215	GO:004282
299	267	306	PF03168	PTHR31852,PTHR31852:SF25				
461	517	679	PF00759	PTHR22298,PTHR2229		3.2.1.4		GO:000597
3233	3654	3972	PF00118	PTHR11353,PTHR11353:SF11				GO:000552
564	566	631	PF00076	PTHR24012	KOG0106,KOG0131,KOG0144			GO:000367
1445	1409	1653	PF00213	PTHR11910	KOG1662	3.6.3.14	K02113	GO:004693
1662	1888	2142	PF14310,F	PTHR30620,PTHR3062		3.2.1.55,	K15920	GO:000597
1686	1642	2024	PF01073,F	PTHR11540		1.1.1.37	K00026	GO:005511
364	432	561	PF03352	PTHR31116,PTHR3111		3.2.2.20	K01246	GO:000872
284	330	324	PF08031,F	PTHR32448,PTHR3244		1.3.3.8		GO:005511
159	160	146	PF00468	PTHR14503,PTHR14503:SF3			K02914	GO:000641
808	870	985		PTHR11177,PTHR11177:SF167			K17525	
136	140	176	PF00657	PTHR22835,PTHR2283		3.1.1.3		GO:001678
1103	1025	1139	PF00085,F	PTHR23293	KOG0189	1.8.4.10,	K05907	GO:004545
6259	6804	7561	PF00012	PTHR19375,PTHR19375:SF147				

1226	1128	1363	PF03629	PTHR31988,PTHR3198		3.1.1.72		
649	557	668	PF08534	PTHR10681	KOG0855	1.11.1.15	K03564	GO:001649
148	158	203	PF14543	PTHR13683	KOG1339	3.4.23.12		GO:000650
153	132	160	PF00396,F	PTHR12411	KOG1543,K	3.4.22.14		GO:000823
693	555	627	PF01789	PTHR31407,PTHR31407:SF20				GO:001989
109	141	135	PF00403	PTHR22814	KOG1603			GO:004687
10	4	11	PF00187,F	PTHR22595	KOG4742	3.2.1.14	K01183	GO:000806
29	51	63	PF14432,F	PTHR24015,PTHR24015:SF107				
350	322	417		PTHR35299,PTHR35299:SF2				
7	13	14	PF00432	PTHR11764,PTHR1176		5.4.99.39		GO:000382
92	121	123	PF06480,F	PTHR23076,PTHR23076:SF60				GO:001602
2544	2259	2322	PF03767	PTHR31284,PTHR3128		3.1.3.2		GO:000399
308	299	351	PF00067	PTHR24286	KOG0157		K09588	GO:005511
211	180	230	PF12854,F	PTHR24015,PTHR24015:SF437				
1597	1456	1763	PF00076	PTHR24012	KOG0131		K11294	GO:000367
496	451	443	PF04752	PTHR12192	KOG3182			
1156	1134	1302	PF03144,F	PTHR23115,PTHR2311		3.6.5.3	K02358	GO:000552
154	155	194	PF02037,F	PTHR31407,PTHR31407:SF5				
98	117	102	PF08659	PTHR24322	KOG0725	1.1.1.223,1.1.1.243		
162	155	142	PF00656	PTHR31810,PTHR31810:SF2				GO:000650
29	29	53	PF00026	PTHR13683	KOG1339	3.4.23.12		GO:000650
374	472	482	PF00657	PTHR22835,PTHR2283		3.2.1.51		GO:001678
2173	2680	2817		PTHR37209,PTHR37209:SF2				
127	126	140		PTHR14241,PTHR14241:SF18				
26	64	88	PF08263,F	PTHR27000	KOG1187	2.7.11.1		GO:000551
4890	5246	4965	PF00044	PTHR10836,PTHR1083		1.2.1.12,1.2.1.13		GO:005511
22	22	35	PF00201	PTHR11926	KOG1192	2.4.1.115		GO:001675
68	111	96		PTHR31032,PTHR31032:SF1				
35	69	75	PF00076	PTHR24012	KOG0149			GO:000367
2042	1996	2004	PF01636	PTHR34273,PTHR3427		2.7.1.100		
1312	1269	1582	PF00076	PTHR24012	KOG0131		K11294	GO:000367
5979	6193	6116	PF00274	PTHR11627,PTHR1162		4.1.2.13	K01623	GO:000609
6160	6252	6714	PF00004	PTHR32429	KOG0651			GO:000552
211	157	213						
1585	1390	1713	PF13193,F	PTHR24095,PTHR24095:SF189				GO:000815
796	667	538	PF14368	PTHR33122,PTHR33122:SF8				
1646	1551	1658	PF02915	PTHR31053,PTHR3105		1.14.13.8	K04035	GO:005511
2182	1991	2131	PF13489,F	PTHR10108	KOG1269	2.1.1.103	K05929	GO:000816
90	93	98	PF08276,F	PTHR27002	KOG1187	2.7.11.1		GO:004854
16	10	8	PF00685	PTHR11783	KOG1584	2.8.2.24		GO:000814
217	245	274	PF02434	PTHR10811	KOG2246	2.4.1.222		GO:001675
2650	2701	2764	PF03951,F	PTHR20852,PTHR2085		6.3.1.2	K01915	GO:000680
651	576	663	PF13326	PTHR34041,PTHR34041:SF1			K08902	GO:001020
176	183	208	PF01789	PTHR31407,PTHR31407:SF15				GO:001989
190	187	255	PF00657	PTHR22835,PTHR2283		3.1.1.3		GO:001678
1185	1250	1145	PF12847	PTHR10108	KOG1270	2.1.1.11	K03428	
224	214	268	PF00076	PTHR24012	KOG0148			GO:000367
830	817	797	PF07731,F	PTHR11709	KOG1263	1.10.3.3	K00423	GO:005511
20050	20501	18852	PF00504	PTHR21649,PTHR21649:SF26			K08912	GO:001602
237	199	271	PF14577,F	PTHR33232				
247	365	444	PF01190	PTHR23201,PTHR23201:SF16				
3217	3120	3103	PF00504	PTHR21649,PTHR21649:SF8			K08913	GO:001602
652	652	594	PF08041	PTHR34951				GO:000951
21	17	24	PF05691	PTHR31268,PTHR3126		2.4.1.82		

11	15	18	PF00168	PTHR10024,PTHR10024:SF172				GO:000551
19	22	30	PF00875,P	PTHR11455,PTHR1145		4.1.99.13	K02295	
896	864	920	PF00572	PTHR11545	KOG3203			GO:000641
71	110	150	PF13802,P	PTHR22762,PTHR2276		3.2.1.20	K01187	GO:000597
561	608	664	PF00759	PTHR22298,PTHR2229		3.2.1.4		GO:000597
369	335	325	PF00171	PTHR11699,PTHR1169		1.2.1.5	K00128	GO:005511
242	153	208	PF00197	PTHR33107,PTHR33107:SF5				GO:000486
591	504	571	PF04752	PTHR12192	KOG3182			
37	48	40	PF00301	PTHR10909				GO:000550
4423	4505	4410	PF00044,P	PTHR10836	KOG0657	1.2.1.12,	K05298	GO:005511
2849	2567	2603	PF00504	PTHR14154,PTHR14154:SF10			K03542	
103	113	110	PF00230	PTHR19139	KOG0223		K09872	GO:001602
15	5	15		PTHR35697,PTHR35697:SF1				
808	741	837	PF05694	PTHR23300			K17285	GO:000843
2262	2170	2237	PF00316	PTHR11556	KOG1458	3.1.3.37	K01100	GO:004257
8002	8134	7351	PF05757	PTHR33399,PTHR33399:SF3			K08901	GO:001989
3103	3319	3522	PF01293	PTHR30031,PTHR3003		4.1.1.49,	K01610	GO:000609
30	33	26	PF00201	PTHR11926,PTHR11926:SF298				GO:001675
491	603	566	PF06955,P	PTHR31062,PTHR3106		2.4.1.207	K08235	GO:004804
23	25	25	PF08541,P	PTHR31561,PTHR3156		2.3.1.199	K15397	GO:001674
28	41	29	PF03098	PTHR11903	KOG2408			
179	137	169	PF04193	PTHR16201	KOG2913			
16566	16571	14704	PF00504	PTHR21649,PTHR21649:SF26			K08912	GO:001602
17	5	8	PF13360	PTHR32303,PTHR3230		1.1.2.6		
1240	1298	1471	PF04043,P	PTHR31707,PTHR3170		3.1.1.11	K01051	GO:000485
494	517	530	PF10674	PTHR35319,PTHR35319:SF2				
308	305	237	PF00334	PTHR11349,PTHR1134		2.7.4.6		GO:000624
2728	2890	2966	PF00118	PTHR11353,PTHR11353:SF8			K04077	GO:000552
18	31	47	PF01477,P	PTHR11771,PTHR1177		1.13.11.1	K00454	GO:000551
12	2	4	PF02727,P	PTHR10638,PTHR1063		1.4.3.21	K00276	GO:005511
198	138	189	PF00656	PTHR31810	KOG1546			GO:000650
2011	2165	2209		PTHR31250,PTHR31250:SF10				
442	406	484	PF00466	PTHR11560,PTHR11560:SF8			K02864	GO:004225
28	63	87		PTHR35480	KOG0978,KOG4593			
332	321	342	PF00450	PTHR11802,PTHR1180		3.4.16.6	K16297	GO:000650
306	269	314	PF00150	PTHR31451,PTHR3145		3.2.1.78	K19355	GO:000597
801	1129	1318	PF03953,P	PTHR11588,PTHR1158		2.4.2.1	K07375	GO:000392
5	2	9	PF00319,P	PTHR11945	KOG0014			GO:004698
130	160	214	PF05199,P	PTHR11552,PTHR11552:SF69			K15403	GO:005511
1857	1621	1460	PF01641	PTHR10173	KOG0856	1.8.4.12	K07305	GO:005511
9	21	15	PF00106	PTHR24322,PTHR24322:SF442				GO:001649
26	30	45	PF00657	PTHR22835,PTHR2283		3.1.1.3		GO:001678
472	457	321	PF06549	PTHR31425,PTHR31425:SF3				
20509	16798	17000	PF00407	PTHR31338,PTHR31338:SF15				GO:000960
23	5	18	PF00187,P	PTHR22595	KOG4742	3.2.1.14	K01183	GO:000806
13	10	13	PF03016	PTHR11062,PTHR1106		2.4.2.41		
36	26	46	PF12854,P	PTHR24015,PTHR24015:SF492				
1284	1668	1646	PF03330,P	PTHR31867,PTHR31867:SF22				
489	455	508	PF13847,P	PTHR10108,PTHR1010		2.1.1.79		
58	60	51	PF00254	PTHR10516	KOG0544	5.2.1.8		GO:000645
23	13	16	PF00067	PTHR24298	KOG0156			GO:005511
23	19	27	PF02458	PTHR31625,PTHR3162		2.3.1.115		GO:001674
530	506	736	PF01740,P	PTHR11814,PTHR11814:SF65			K17470	GO:001602
419	460	390		PTHR34940,PTHR34940:SF1				

312	346	331	PF00027,F	PTHR10217,PTHR10217:SF476				GO:005508
58	36	55	PF00106	PTHR24320	KOG1208	1.1.1.330		GO:001649
58	36	51	PF11744	PTHR31086,PTHR31086:SF22				GO:001574
34	32	38	PF14416,F	PTHR32285,PTHR32285:SF42				
234	250	314	PF02325	PTHR33219,PTHR33219:SF4			K02221	GO:001602
372	370	328	PF00445	PTHR11240	KOG1642	3.1.27.1	K01166	GO:003389
213	265	342	PF02469	PTHR32382,PTHR32382:SF4				
2534	2826	3239	PF04862	PTHR31265,PTHR31265:SF3				
1214	1168	1140	PF14159	PTHR33222,PTHR33222:SF9				
734	722	910	PF00573	PTHR10746	KOG1624		K02926	GO:000641
313	350	437	PF09118,F	PTHR32208,PTHR3220		1.1.3.9		
522	617	981	PF09478,F	PTHR22298,PTHR2229		3.2.1.4		GO:003024
4510	4786	4921	PF00127	PTHR34192,PTHR34192:SF2			K02638	GO:000905
1222	1273	1418	PF01078	PTHR32039,PTHR3203		6.6.1.1	K03405	GO:001685
32	14	20	PF00657	PTHR22835,PTHR2283		3.1.1.3		GO:001678
384	359	439	PF05834	PTHR13789,PTHR1378		5.5.1.18	K06444	GO:001670
162	189	186	PF10604	PTHR31907,PTHR31907:SF1				
528	480	663	PF01740,F	PTHR11814,PTHR11814:SF65			K17470	GO:001602
1392	1119	1438	PF00044	PTHR10836,PTHR1083		1.2.1.12,1.2.1.13		GO:005511
788	918	1008	PF02518,F	PTHR11528,PTHR11528:SF41			K09487	GO:005108
1303	1179	1194	PF02668	PTHR10696,PTHR10696:SF21				GO:005511
7467	7625	8676	PF06955,F	PTHR31062,PTHR3106		2.4.1.207	K08235	GO:004804
1088	977	1058	PF00230	PTHR19139	KOG0223			GO:001602
852	831	1061	PF03460,F	PTHR11493,PTHR1149		1.8.7.1	K00392	GO:005511
18	22	9	PF00249	PTHR10641	KOG0048		K09422	
708	886	947	PF14215	PTHR13902,PTHR13902:SF23				
122	58	60	PF00187,F	PTHR22595	KOG4742	3.2.1.14		GO:000806
3987	4307	4817	PF02780,F	PTHR11624,PTHR1162		2.2.1.2,2	K00615	GO:000815
276	329	274	PF05498	PTHR33136,PTHR33136:SF7				
443	581	601	PF06955,F	PTHR31062,PTHR3106		2.4.1.207	K08235	GO:004804
709	659	739	PF03059	PTHR32266,PTHR3226		2.5.1.43	K05953	GO:003041
10	14	10	PF00646	PTHR33736,PTHR33736:SF5				GO:000551
352	398	391	PF03195	PTHR31301,PTHR31301:SF24				
1258	1142	1158	PF00504	PTHR21649,PTHR21649:SF20			K08912	GO:001602
24	46	41	PF04667	PTHR34804	KOG4076			
6159	6171	6629	PF01134	PTHR10617,PTHR1061		1.3.1.83	K10960	GO:005066
792	1012	1042	PF00282	PTHR11999,PTHR1199		4.1.1.15	K01580	GO:003017
56	47	42	PF16113	PTHR11941	KOG1684	3.1.2.4	K05605	
3	7	3	PF13632	PTHR13301,PTHR1330		2.4.2.24		
28	33	30	PF04640	PTHR31065,PTHR31065:SF14				
880	900	928	PF14845,F	PTHR22600	KOG2499	3.2.1.52	K12373	GO:000597
2	5	10		PTHR33264,PTHR33264:SF6				
2131	2206	2857	PF00544	PTHR31683,PTHR3168		4.2.2.2	K01728	
16	12	13	PF00582	PTHR31964,PTHR31964:SF37				GO:000695
30	33	32	PF01370	PTHR10366	KOG1502	1.3.1.77		GO:005066
48	39	37	PF00106	PTHR24322	KOG1208	1.3.1.33	K00218	GO:001649
8186	7953	9050	PF00438,F	PTHR11964,PTHR1196		2.5.1.6	K00789	GO:000655
527	554	677	PF01964	PTHR30557,PTHR3055		4.1.99.17	K03147	GO:005153
3070	2950	3113	PF03951,F	PTHR20852,PTHR2085		6.3.1.2	K01915	GO:000680
25	25	31	PF13639	PTHR14155,PTHR14155:SF78			K19041	GO:000827
24	24	32	PF00111	PTHR23426	KOG3309			GO:005153
18	27	26		PTHR33597,PTHR33597:SF1				
1120	993	1398	PF04862	PTHR31265,PTHR31265:SF3				
56	26	48	PF13837	PTHR33492,PTHR33492:SF2				

3310	3181	3318	PF12338,F	PTHR31262,PTHR3126		4.1.1.39	K01602	
26	34	47						
8	19	21						
45	12	20	PF00657	PTHR22835,PTHR2283		3.1.1.3		GO:001678
2	9	2	PF04833	PTHR31673,PTHR31673:SF3				GO:003122
4	6	20	PF12854,F	PTHR24015,PTHR24015:SF496				
673	669	730	PF00141	PTHR31356,PTHR3135		1.11.1.11	K00434	GO:005511
14	12	12						
2248	2097	2203	PF00106	PTHR24322	KOG1208	1.3.1.33	K00218	GO:001649
4	6	14	PF00010	PTHR11969,PTHR11969:SF22				GO:004698
13789	13445	13700	PF12338,F	PTHR31262,PTHR3126		4.1.1.39	K01602	
9	2	4	PF00403	PTHR22814	KOG1603			GO:004687
16	20	20		PTHR33833,PTHR33833:SF1				
5	2	8	PF08879	PTHR34680				
19	9	14	PF00046,F	PTHR24326	KOG0484		K09338	GO:000367
11	13	17	PF00931	PTHR23155	KOG4658			GO:004353
14	12	24	PF00657	PTHR22835,PTHR2283		3.1.1.3		GO:001678
16	21	14	PF01764	PTHR21493	KOG4569	3.1.1.3		GO:000662
4	4	3	PF16113	PTHR11941	KOG1684	3.1.2.4		
20	19	21	PF14008,F	PTHR22953	KOG1378	3.1.3.2		GO:001678

Best.hit.arabi.sym	arabi.defline
AT5G25610ATRD22,RD	BURP domain-containing protein
AT2G15680.1	Calcium-binding EF-hand family protein
AT3G48660.1	Protein of unknown function (DUF 3339)
AT2G39200ATMLO12,M	Seven transmembrane MLO family protein
AT2G44160MTHFR2	methylenetetrahydrofolate reductase 2
AT3G02070.1	Cysteine proteinases superfamily protein
AT5G53900.2	Serine/threonine-protein kinase WNK (With No Lysine)-related
AT4G39720.1	VQ motif-containing protein
AT5G15900TBL19	TRICHOME BIREFRINGENCE-LIKE 19
AT1G24430.1	HXXXD-type acyl-transferase family protein
AT5G01360TBL3	Plant protein of unknown function (DUF828)
AT5G12300.1	Calcium-dependent lipid-binding (CaLB domain) family protein
AT2G30210LAC3	laccase 3
AT5G27550.1	P-loop containing nucleoside triphosphate hydrolases superfamil
AT3G24770CLE41	CLAVATA3/ESR-RELATED 41
AT4G19380.1	Long-chain fatty alcohol dehydrogenase family protein
AT1G24430.1	HXXXD-type acyl-transferase family protein
AT4G23010ATUTR2,UT	UDP-galactose transporter 2
AT2G25470AtRLP21,R	receptor like protein 21
AT4G16480ATINT4,IN	inositol transporter 4
AT5G17420ATCESA7,C	Cellulose synthase family protein
AT3G46130ATMYB48,A	myb domain protein 48
AT5G36110CYP716A1	cytochrome P450, family 716, subfamily A, polypeptide 1
AT1G20030.2	Pathogenesis-related thaumatin superfamily protein
AT3G09600.1	Homeodomain-like superfamily protein
AT1G06620.1	2-oxoglutarate (2OG) and Fe(II)-dependent oxygenase superfamily
AT5G10860.1	Cystathionine beta-synthase (CBS) family protein
AT1G54870.1	NAD(P)-binding Rossmann-fold superfamily protein
AT1G42430.1	
AT3G42725.1	Putative membrane lipoprotein
AT2G24430ANAC038,A	NAC domain containing protein 38
AT5G39360EDL2	EID1-like 2
AT5G65170.1	VQ motif-containing protein
AT5G22810.1	GDSL-like Lipase/Acylhydrolase superfamily protein
AT3G08550ABI8,ELD1	elongation defective 1 protein / ELD1 protein
AT3G27027.1	Protein of unknown function (DUF 3339)
AT3G13130.1	
AT3G57880.1	Calcium-dependent lipid-binding (CaLB domain) plant phosphoribo
AT2G41690AT-HSFB3,	heat shock transcription factor B3
AT5G07050.1	nodulin MtN21 /EamA-like transporter family protein
AT5G23750.2	Remorin family protein
AT1G16860.1	Ubiquitin-specific protease family C19-related protein
AT2G18370.1	Bifunctional inhibitor/lipid-transfer protein/seed storage 2S a
AT3G49290ABIL2	ABL interactor-like protein 2
AT3G26040.1	HXXXD-type acyl-transferase family protein
AT1G74520ATHVA22A,	HVA22 homologue A
AT4G21380ARK3,RK3	receptor kinase 3
AT2G32150.1	Haloacid dehalogenase-like hydrolase (HAD) superfamily protein
AT1G56170ATHAP5B,H	nuclear factor Y, subunit C2
AT4G35550ATWOX13,H	WUSCHEL related homeobox 13
AT5G51190.1	Integrase-type DNA-binding superfamily protein

AT4G16520	ATG8F	Ubiquitin-like superfamily protein
AT2G25620	AtDBP1,DB	DNA-binding protein phosphatase 1
AT1G27620.1		HXXXD-type acyl-transferase family protein
AT3G10870	ATMES17,M	methyl esterase 17
AT5G08350.1		GRAM domain-containing protein / ABA-responsive protein-related
AT3G45140	ATLOX2,LC	lipoxygenase 2
AT4G38690.1		PLC-like phosphodiesterases superfamily protein
AT4G25810	XTH23,XTR	xyloglucan endotransglycosylase 6
AT1G01780.1		GATA type zinc finger transcription factor family protein
AT1G03610.1		Protein of unknown function (DUF789)
AT1G71695.1		Peroxidase superfamily protein
AT5G12330	LRP1	Lateral root primordium (LRP) protein-related
AT1G32910.1		HXXXD-type acyl-transferase family protein
AT4G35020	ARAC3,ATR	RAC-like 3
AT1G15740.1		Leucine-rich repeat family protein
AT3G45160.1		Putative membrane lipoprotein
AT4G39730.1		Lipase/lipooxygenase, PLAT/LH2 family protein
AT2G16700	ADF5,ATAD	actin depolymerizing factor 5
AT1G08320	bZIP21,TG	bZIP transcription factor family protein
AT3G60130	BGLU16	beta glucosidase 16
AT5G03760	ATCSLA09,	Nucleotide-diphospho-sugar transferases superfamily protein
AT3G01860.1		
AT4G28600	NPGR2	no pollen germination related 2
AT3G63520	ATCCD1,AT	carotenoid cleavage dioxygenase 1
AT5G25610	ATRD22,RD	BURP domain-containing protein
AT3G62950.1		Thioredoxin superfamily protein
AT5G05790.1		Duplicated homeodomain-like superfamily protein
AT5G07990	CYP75B1,D	Cytochrome P450 superfamily protein
AT5G40780	LHT1	lysine histidine transporter 1
AT5G55250	IAMT1	IAA carboxylmethyltransferase 1
AT5G47635.1		Pollen Ole e 1 allergen and extensin family protein
AT2G27310.1		F-box family protein
AT3G55420.1		
AT4G10550.1		Subtilase family protein
AT4G05200	CRK25	cysteine-rich RLK (RECEPTOR-like protein kinase) 25
AT2G03200.1		Eukaryotic aspartyl protease family protein
AT3G47420	ATPS3,PS3	phosphate starvation-induced gene 3
AT3G56680.1		Single-stranded nucleic acid binding R3H protein
AT1G59870	ABCG36,AT	ABC-2 and Plant PDR ABC-type transporter family protein
AT3G50410	OBP1	OBF binding protein 1
AT1G22530	PATL2	PATELLIN 2
AT1G08290	WIP3	WIP domain protein 3
AT5G59080.1		
AT1G25550.1		myb-like transcription factor family protein
AT3G09390	ATMT-1,AT	metallothionein 2A
AT1G77400.1		
AT4G18260.1		Cytochrome b561/ferric reductase transmembrane protein family
AT1G65520	ATECI1,EC	delta(3), delta(2)-enoyl CoA isomerase 1
AT5G17920	ATCIMS,AT	Cobalamin-independent synthase family protein
AT3G56880.1		VQ motif-containing protein
AT5G67140.1		F-box/RNI-like superfamily protein
AT1G78380	ATGSTU19,	glutathione S-transferase TAU 19
AT3G09390	ATMT-1,AT	metallothionein 2A
AT4G35390	AGF1	AT-hook protein of GA feedback 1

AT1G79420.1		Protein of unknown function (DUF620)
AT5G57620	AtMYB36,M	myb domain protein 36
AT1G28360	ATERF12,E	ERF domain protein 12
AT5G17840.1		DnaJ/Hsp40 cysteine-rich domain superfamily protein
AT2G03480	QUL2	QUASIMODO2 LIKE 2
AT4G33450	ATMYB69,M	myb domain protein 69
AT4G19670.2		RING/U-box superfamily protein
AT2G37150.1		RING/U-box superfamily protein
AT3G28050.1		nodulin MtN21 /EamA-like transporter family protein
AT3G18170.1		Glycosyltransferase family 61 protein
AT3G11760.1		
AT2G25150.1		HXXXD-type acyl-transferase family protein
AT3G18660	GUX1,PGSI	plant glycogenin-like starch initiation protein 1
AT3G59690	IQD13	IQ-domain 13
AT5G44640	BGLU13	beta glucosidase 13
AT5G04080.1		
AT1G56600	AtGolS2,G	galactinol synthase 2
AT1G32170	XTH30,XTR	xyloglucan endotransglucosylase/hydrolase 30
AT5G51460	ATTPPA	Haloacid dehalogenase-like hydrolase (HAD) superfamily protein
AT5G25560.1		CHY-type/CTCHY-type/RING-type Zinc finger protein
AT1G17100.1		SOUL heme-binding family protein
AT2G36840.1		ACT-like superfamily protein
AT1G13750.1		Purple acid phosphatases superfamily protein
AT5G61340.1		
AT4G04900	RIC10	ROP-interactive CRIB motif-containing protein 10
AT5G37690.1		SGNH hydrolase-type esterase superfamily protein
AT2G37040	ATPAL1,PA	PHE ammonia lyase 1
AT5G15790.2		RING/U-box superfamily protein
AT5G15790.2		RING/U-box superfamily protein
AT5G17920	ATCIMS,AT	Cobalamin-independent synthase family protein
AT3G09085.1		Protein of unknown function (DUF962)
AT2G36570.1		Leucine-rich repeat protein kinase family protein
AT3G16230.2		Predicted eukaryotic LigT
AT1G03700.1		Uncharacterised protein family (UPF0497)
AT1G75390	AtbZIP44,	basic leucine-zipper 44
AT5G25190.1		Integrase-type DNA-binding superfamily protein
AT3G49490.1		
AT4G19840	ATPP2-A1,	phloem protein 2-A1
AT1G13635.2		DNA glycosylase superfamily protein
AT5G44440.1		FAD-binding Berberine family protein
AT2G33550.1		Homeodomain-like superfamily protein
AT5G27550.1		P-loop containing nucleoside triphosphate hydrolases superfamil
AT3G55090.1		ABC-2 type transporter family protein
AT4G37790	HAT22	Homeobox-leucine zipper protein family
AT4G04450	AtWRKY42,	WRKY family transcription factor
AT5G41040.1		HXXXD-type acyl-transferase family protein
AT4G24220	AWI31,VEP	NAD(P)-binding Rossmann-fold superfamily protein
AT1G21000.2		PLATZ transcription factor family protein
AT5G47070.1		Protein kinase superfamily protein
AT3G21240	4CL2,AT4C	4-coumarate:CoA ligase 2
AT1G75130	CYP721A1	cytochrome P450, family 721, subfamily A, polypeptide 1
AT2G18060	ANAC037,V	vascular related NAC-domain protein 1

AT3G18430.1		Calcium-binding EF-hand family protein
AT3G54070.1		Ankyrin repeat family protein
AT3G23690.1		basic helix-loop-helix (bHLH) DNA-binding superfamily protein
AT5G23190	CYP86B1	cytochrome P450, family 86, subfamily B, polypeptide 1
AT4G03510	ATRMA1,RM	RING membrane-anchor 1
AT4G25420	AT2301,AT	2-oxoglutarate (2OG) and Fe(II)-dependent oxygenase superfamily
AT2G37360.1		ABC-2 type transporter family protein
AT1G62990	IXR11,KNA	KNOTTED-like homeobox of Arabidopsis thaliana 7
AT1G31280	AGO2	Argonaute family protein
AT2G29130	ATLAC2,LA	laccase 2
AT5G23190	CYP86B1	cytochrome P450, family 86, subfamily B, polypeptide 1
AT1G28380	NSL1	MAC/Perforin domain-containing protein
AT1G66140	ZFP4	zinc finger protein 4
AT2G37040	ATPAL1,PA	PHE ammonia lyase 1
AT3G04590.2		AT hook motif DNA-binding family protein
AT1G17370	UBP1B	oligouridylate binding protein 1B
AT2G38570.1		
AT4G33580	ATBCA5,BC	beta carbonic anhydrase 5
AT1G18330	EPR1,RVE7	Homeodomain-like superfamily protein
AT2G42610	LSH10	Protein of unknown function (DUF640)
AT2G38080	ATLMCO4,I	Laccase/Diphenol oxidase family protein
AT2G35940	BLH1,EDA2	BEL1-like homeodomain 1
AT1G66120.1		AMP-dependent synthetase and ligase family protein
AT4G17900.1		PLATZ transcription factor family protein
AT1G20990.1		Cysteine/Histidine-rich C1 domain family protein
AT1G80130.1		Tetratricopeptide repeat (TPR)-like superfamily protein
AT3G45240	ATSNAK2,G	geminivirus rep interacting kinase 1
AT5G42380	CML37,CML	calmodulin like 37
AT2G26560	PLA IIA,P	phospholipase A 2A
AT4G19420.1		Pectinacetylesterase family protein
AT5G42050.1		DCD (Development and Cell Death) domain protein
AT1G80750.1		Ribosomal protein L30/L7 family protein
AT4G35750.1		SEC14 cytosolic factor family protein / phosphoglyceride transf
AT1G74100	ATSOT16,A	sulfotransferase 16
AT2G43290	MSS3	Calcium-binding EF-hand family protein
AT4G20190.1		
AT3G17100.2		sequence-specific DNA binding transcription factors
AT4G24010	ATCSLG1,C	cellulose synthase like G1
AT2G24960.2		
AT3G22600.1		Bifunctional inhibitor/lipid-transfer protein/seed storage 2S a
AT3G15580	APG8H,ATG	Ubiquitin-like superfamily protein
AT4G27460.1		Cystathionine beta-synthase (CBS) family protein
AT1G27730	STZ,ZAT10	salt tolerance zinc finger
AT4G22920	ATNYE1,NY	non-yellowing 1
AT2G48130.1		Bifunctional inhibitor/lipid-transfer protein/seed storage 2S a
AT2G14830.1		Regulator of Vps4 activity in the MVB pathway protein
AT1G32540	LOL1	lsd one like 1
AT4G37260	ATMYB73,M	myb domain protein 73
AT5G41800.1		Transmembrane amino acid transporter family protein
AT2G35150	EXL1	EXORDIUM like 1
AT1G08230	ATGAT1,GA	Transmembrane amino acid transporter family protein
AT1G75030	ATLP-3,TL	thaumatin-like protein 3

AT2G39855.2
AT2G20500.1
AT5G65170.1 VQ motif-containing protein
AT4G40070.1 RING/U-box superfamily protein
AT2G38630.1 Transducin/WD40 repeat-like superfamily protein
AT2G46780.1 RNA-binding (RRM/RBD/RNP motifs) family protein
AT3G18400anac058,NNAC domain containing protein 58
AT5G23750.2 Remorin family protein

AT1G08510FATB fatty acyl-ACP thioesterases B
AT5G66390.1 Peroxidase superfamily protein
AT5G43150.1
AT5G42610.1 Protein of unknown function (DUF607)
AT3G62020GLP10 germin-like protein 10
AT3G01990ACR6 ACT domain repeat 6
AT5G43420.1 RING/U-box superfamily protein
AT5G58380CIPK10,PKSOS3-interacting protein 1
AT4G28530anac074,NNAC domain containing protein 74
AT3G21530.1 DNAse I-like superfamily protein
AT1G27920MAP65-8 microtubule-associated protein 65-8
AT1G66400CML23 calmodulin like 23
AT2G46770ANAC043,ENAC (No Apical Meristem) domain transcriptional regulator super
AT4G16520ATG8F Ubiquitin-like superfamily protein
AT5G03170ATFLA11,FFASCICLIN-like arabinogalactan-protein 11
AT4G18170ATWRKY28,WRKY DNA-binding protein 28
AT5G64440AtFAAH,FAfatty acid amide hydrolase
AT2G01210.1 Leucine-rich repeat protein kinase family protein
AT4G18170ATWRKY28,WRKY DNA-binding protein 28
AT1G20550.1 O-fucosyltransferase family protein
AT2G14960GH3.1 Auxin-responsive GH3 family protein
AT4G35560.1 Transducin/WD40 repeat-like superfamily protein
AT5G60020ATLAC17,Llaccase 17
AT4G28500ANAC073,NNAC domain containing protein 73

AT4G38620ATMYB4,MYmyb domain protein 4
AT5G10280ATMYB64,Amyb domain protein 92
AT4G33790CER4,FAR3Jojoba acyl CoA reductase-related male sterility protein
AT2G38120AUX1,MAP1Transmembrane amino acid transporter family protein
AT2G38290AMT2,AMT2ammonium transporter 2
AT2G46660CYP78A6 cytochrome P450, family 78, subfamily A, polypeptide 6
AT3G51895AST12,SULsulfate transporter 3;1
AT5G09520.1 hydroxyproline-rich glycoprotein family protein
AT1G79900ATMBAC2,BMitochondrial substrate carrier family protein
AT3G18280.1 Bifunctional inhibitor/lipid-transfer protein/seed storage 2S a
AT3G02040SRG3 senescence-related gene 3
AT4G14465AHL20 AT-hook motif nuclear-localized protein 20
AT1G66920.2 Protein kinase superfamily protein
AT1G77380AAP3,ATAAamino acid permease 3
AT5G17420ATCESA7,CCellulose synthase family protein
AT5G58350WNK4,ZIK2with no lysine (K) kinase 4
AT3G09390ATMT-1,ATmetallothionein 2A

AT4G08300.1 nodulin MtN21 /EamA-like transporter family protein
AT3G55990ESK1,TBL2Plant protein of unknown function (DUF828)

AT3G05950.1		RmlC-like cupins superfamily protein
AT5G38710.1		Methylenetetrahydrofolate reductase family protein
AT1G80160.1		Lactoylglutathione lyase / glyoxalase I family protein
AT2G32090.1		Lactoylglutathione lyase / glyoxalase I family protein
AT2G33550.1		Homeodomain-like superfamily protein
AT4G00750.1		S-adenosyl-L-methionine-dependent methyltransferases superfamil
AT3G16520	UGT88A1	UDP-glucosyl transferase 88A1
AT5G65070	AGL69,FCL	K-box region and MADS-box transcription factor family protein
AT3G19970.1		alpha/beta-Hydrolases superfamily protein
AT4G33565.1		RING/U-box superfamily protein
AT3G59010	PME61	pectin methylesterase 61
AT5G11890.1		
AT1G19715.3		Mannose-binding lectin superfamily protein
AT2G16385.1		
AT5G14090.1		
AT3G18770.1		Autophagy-related protein 13
AT2G33360.1		Protein of unknown function (DUF3527)
AT1G34670	AtMYB93,M	myb domain protein 93
AT5G56750	NDL1	N-MYC downregulated-like 1
AT5G38280	PR5K	PR5-like receptor kinase
AT5G06740.1		Concanavalin A-like lectin protein kinase family protein
AT5G32450.1		RNA binding (RRM/RBD/RNP motifs) family protein
AT3G05270.2		Plant protein of unknown function (DUF869)
AT2G43060	IBH1	ILI1 binding bHLH 1
AT4G36220	CYP84A1,F	ferulic acid 5-hydroxylase 1
AT5G03170	ATFLA11,F	FASCICLIN-like arabinogalactan-protein 11
AT1G34670	AtMYB93,M	myb domain protein 93
AT4G24340.1		Phosphorylase superfamily protein
AT5G24090	ATCHIA,CH	chitinase A
AT2G26110.1		Protein of unknown function (DUF761)
AT2G41560	ACA4	autoinhibited Ca(2+)-ATPase, isoform 4
AT1G70420.1		Protein of unknown function (DUF1645)
AT5G23400.1		Leucine-rich repeat (LRR) family protein
AT5G19640.1		Major facilitator superfamily protein
AT3G21570.1		
AT1G20460.1		
AT1G09620.1		ATP binding;leucine-tRNA ligases;aminoacyl-tRNA ligases;nucleot
AT2G31085	CLE6	CLAVATA3/ESR-RELATED 6
AT5G67210.1		Protein of unknown function (DUF579)
AT5G05960.1		Bifunctional inhibitor/lipid-transfer protein/seed storage 2S a
AT5G11890.1		
AT1G03820.1		
AT1G66120.1		AMP-dependent synthetase and ligase family protein
AT5G22860.1		Serine carboxypeptidase S28 family protein
AT5G58860	CYP86,CYP	cytochrome P450, family 86, subfamily A, polypeptide 1
AT1G61660.1		basic helix-loop-helix (bHLH) DNA-binding superfamily protein
AT5G10180	AST68,SUL	slufate transporter 2;1
AT2G46130	ATWRKY43,	WRKY DNA-binding protein 43
AT5G19860.1		Protein of unknown function, DUF538
AT1G21050.1		Protein of unknown function, DUF617
AT2G38080	ATLMCO4,I	Laccase/Diphenol oxidase family protein
AT1G63020	NRPD1,NRP	nuclear RNA polymerase D1A
AT1G75450	ATCKX5,AT	cytokinin oxidase 5

AT3G27030.1
AT3G51130.1

AT4G33790CER4,FAR3Jojoba acyl CoA reductase-related male sterility protein
AT1G14780.1 MAC/Perforin domain-containing protein
AT2G27690CYP94C1 cytochrome P450, family 94, subfamily C, polypeptide 1
AT3G18950.1 Transducin/WD40 repeat-like superfamily protein
AT4G10310ATHKT1,HKhigh-affinity K+ transporter 1
AT1G02630.1 Nucleoside transporter family protein
AT3G12720ATMYB67,Amyb domain protein 67
AT5G06090ATGPAT7,Gglycerol-3-phosphate acyltransferase 7
AT2G05990ENR1,MOD1NAD(P)-binding Rossmann-fold superfamily protein
AT1G27920MAP65-8 microtubule-associated protein 65-8
AT1G19300ATGATL1,GNucleotide-diphospho-sugar transferases superfamily protein
AT5G43700ATAUX2-11AUX/IAA transcriptional regulator family protein
AT5G62350.1 Plant invertase/pectin methylesterase inhibitor superfamily pro
AT1G14600.1 Homeodomain-like superfamily protein
AT3G50610.1
AT4G28640IAA11 indole-3-acetic acid inducible 11
AT2G26290ARSK1 root-specific kinase 1
AT2G23760BLH4,SAW2BEL1-like homeodomain 4
AT1G14720ATXTH28,Exyloglucan endotransglucosylase/hydrolase 28
AT1G22710ATSUC2,SUsucrose-proton symporter 2
AT3G11430ATGPAT5,Gglycerol-3-phosphate acyltransferase 5
AT2G26560PLA IIA,Pphospholipase A 2A
AT3G13890ATMYB26,Mmyb domain protein 26
AT1G32450NRT1.5 nitrate transporter 1.5
AT2G16400BLH7 BEL1-like homeodomain 7
AT2G29150.1 NAD(P)-binding Rossmann-fold superfamily protein
AT3G22540.1 Protein of unknown function (DUF1677)
AT4G12350AtMYB42,Mmyb domain protein 42
AT2G25737.1 Sulfite exporter TauE/SafE family protein
AT4G06744.1 Leucine-rich repeat (LRR) family protein

AT1G75250ATRL6,RL6RAD-like 6
AT1G56600AtGolS2,Ggalactinol synthase 2
AT2G23520.1 Pyridoxal phosphate (PLP)-dependent transferases superfamily pr
AT5G01750.2 Protein of unknown function (DUF567)
AT1G44170ALDH3H1,Aaldehyde dehydrogenase 3H1
AT5G43150.1
AT4G16480ATINT4,INinositol transporter 4
AT1G21000.2 PLATZ transcription factor family protein
AT3G59850.1 Pectin lyase-like superfamily protein

AT5G54160ATOMT1,OMO-methyltransferase 1
AT5G58860CYP86,CYPcytochrome P450, family 86, subfamily A, polypeptide 1
AT5G02140.1 Pathogenesis-related thaumatin superfamily protein
AT2G38470ATWRKY33,WRKY DNA-binding protein 33
AT4G32810ATCCD8,CCcarotenoid cleavage dioxygenase 8
AT3G53980.1 Bifunctional inhibitor/lipid-transfer protein/seed storage 2S a
AT4G34160CYCD3,CYCCYCLIN D3;1
AT3G05950.1 RmlC-like cupins superfamily protein
AT2G37130.1 Peroxidase superfamily protein
AT5G66870ASL1,LBD3ASYMMETRIC LEAVES 2-like 1

AT5G05390	LAC12	laccase 12
AT2G37040	ATPAL1,PA	PHE ammonia lyase 1
AT5G02070.1		Protein kinase family protein
AT1G06330.1		Heavy metal transport/detoxification superfamily protein
AT4G36220	CYP84A1,F	ferulic acid 5-hydroxylase 1
AT5G18270	ANAC087	Arabidopsis NAC domain containing protein 87
AT5G16490	RIC4	ROP-interactive CRIB motif-containing protein 4
AT2G30490	ATC4H,C4H	cinnamate-4-hydroxylase
AT1G12940	ATNRT2.5,	nitrate transporter2.5

AT5G23750.1		Remorin family protein
AT1G65730	YSL7	YELLOW STRIPE like 7
AT5G37690.1		SGNH hydrolase-type esterase superfamily protein
AT5G62865.1		
AT2G28250	NCRK	Protein kinase superfamily protein
AT1G44350	ILL6	IAA-leucine resistant (ILR)-like gene 6
AT2G46770	ANAC043,E	NAC (No Apical Meristem) domain transcriptional regulator super

AT1G24430.1		HXXXD-type acyl-transferase family protein
AT1G19850	ARF5,IAA2	Transcriptional factor B3 family protein / auxin-responsive fac
AT3G17510	CIPK1,SnR	CBL-interacting protein kinase 1
AT2G44500.1		O-fucosyltransferase family protein
AT5G22860.1		Serine carboxypeptidase S28 family protein
AT2G38060	PHT4;2	phosphate transporter 4;2
AT5G13220	JAS1,JAZ1	jasmonate-zim-domain protein 10
AT3G63010	ATGID1B,G	alpha/beta-Hydrolases superfamily protein
AT1G79620.1		Leucine-rich repeat protein kinase family protein
AT3G16360	AHP4	HPT phosphotransmitter 4
AT5G44550.1		Uncharacterised protein family (UPF0497)
AT4G02590	UNE12	basic helix-loop-helix (bHLH) DNA-binding superfamily protein
AT5G42180.1		Peroxidase superfamily protein
AT2G33385	arpc2b	actin-related protein C2B
AT2G40470	ASL11,LBD	LOB domain-containing protein 15
AT3G15990	SULTR3;4	sulfate transporter 3;4
AT1G77760	GNR1,NIA1	nitrate reductase 1
AT5G37478.1		TPX2 (targeting protein for Xklp2) protein family
AT1G60420.1		DC1 domain-containing protein
AT3G30340.1		nodulin MtN21 /EamA-like transporter family protein
AT2G15790	CYP40,SQN	peptidyl-prolyl cis-trans isomerase / cyclophilin-40 (CYP40) /
AT4G35750.1		SEC14 cytosolic factor family protein / phosphoglyceride transf
AT1G19715.3		Mannose-binding lectin superfamily protein
AT2G18360.1		alpha/beta-Hydrolases superfamily protein
AT5G13220	JAS1,JAZ1	jasmonate-zim-domain protein 10
AT3G25930.1		Adenine nucleotide alpha hydrolases-like superfamily protein
AT1G25330.1		basic helix-loop-helix (bHLH) DNA-binding superfamily protein
AT5G05960.1		Bifunctional inhibitor/lipid-transfer protein/seed storage 2S a
AT1G33800.1		Protein of unknown function (DUF579)
AT2G33385	arpc2b	actin-related protein C2B
AT1G29000.1		Heavy metal transport/detoxification superfamily protein
AT5G09360	LAC14	laccase 14
AT4G20970.1		basic helix-loop-helix (bHLH) DNA-binding superfamily protein
AT5G67020.1		
AT4G15610.1		Uncharacterised protein family (UPF0497)
AT3G54070.1		Ankyrin repeat family protein

AT2G38110	ATGPAT6,G	glycerol-3-phosphate acyltransferase 6
AT1G58120.1		
AT5G66390.1		Peroxidase superfamily protein
AT3G49780	ATPSK3 (F	phytosulfokine 4 precursor
AT2G32560.1		F-box family protein
AT1G05710.5		basic helix-loop-helix (bHLH) DNA-binding superfamily protein
AT1G70880.1		Polyketide cyclase/dehydrase and lipid transport superfamily pr
AT2G11890.1		adenylate cyclases
AT1G34670	AtMYB93,M	myb domain protein 93
AT3G18710	ATPUB29,P	plant U-box 29
AT2G30210	LAC3	laccase 3
AT5G46050	ATPTR3,PT	peptide transporter 3
AT1G60190.1		ARM repeat superfamily protein
AT4G27450.1		Aluminium induced protein with YGL and LRDR motifs
AT2G27430.1		ARM repeat superfamily protein
AT5G55970.1		RING/U-box superfamily protein
AT1G27730	STZ,ZAT10	salt tolerance zinc finger
AT4G31130.1		Protein of unknown function (DUF1218)
AT4G18780	ATCESA8,C	cellulose synthase family protein
AT1G19110.1		inter-alpha-trypsin inhibitor heavy chain-related
AT3G03280.1		
AT4G32280	IAA29	indole-3-acetic acid inducible 29
AT1G20960	emb1507	U5 small nuclear ribonucleoprotein helicase, putative
AT2G40320	TBL33	TRICHOME BIREFRINGENCE-LIKE 33
AT2G26660	ATSPX2,SP	SPX domain gene 2
AT1G09610.1		Protein of unknown function (DUF579)
AT1G61820	BGLU46	beta glucosidase 46
AT1G68850.1		Peroxidase superfamily protein
AT1G72220.1		RING/U-box superfamily protein
AT5G65790	ATMYB68,M	myb domain protein 68
AT1G08990	PGSIP5	plant glycogenin-like starch initiation protein 5
AT4G32480.1		Protein of unknown function (DUF506)
AT4G18170	ATWRKY28,	WRKY DNA-binding protein 28
AT1G08230	ATGAT1,GA	Transmembrane amino acid transporter family protein
AT2G23540.1		GDSL-like Lipase/Acylhydrolase superfamily protein
AT5G48930	HCT	hydroxycinnamoyl-CoA shikimate/quinate hydroxycinnamoyl transfe
AT3G04530	ATPPCK2,P	phosphoenolpyruvate carboxylase kinase 2
AT1G55760.1		BTB/POZ domain-containing protein
AT1G46768	RAP2.1	related to AP2 1
AT5G65500.1		U-box domain-containing protein kinase family protein
AT5G02090.1		
AT5G23750.1		Remorin family protein
AT1G75250	ATRL6,RL6	RAD-like 6
AT1G17950	ATMYB52,B	myb domain protein 52
AT1G32450	NRT1.5	nitrate transporter 1.5
AT2G48140	EDA4	Bifunctional inhibitor/lipid-transfer protein/seed storage 2S a
AT2G46770	ANAC043,E	NAC (No Apical Meristem) domain transcriptional regulator super
AT1G33760.1		Integrase-type DNA-binding superfamily protein
AT1G62710	BETA-VPE,	beta vacuolar processing enzyme
AT5G20260.1		Exostosin family protein
AT3G57540.1		Remorin family protein
AT4G20260	ATPCAP1,P	plasma-membrane associated cation-binding protein 1
AT5G45710	AT-HSFA4C	winged-helix DNA-binding transcription factor family protein

AT3G54070.1	Ankyrin repeat family protein
AT1G59740.1	Major facilitator superfamily protein
AT5G50760.1	SAUR-like auxin-responsive protein family
AT3G59690 IQD13	IQ-domain 13
AT3G61680.1	alpha/beta-Hydrolases superfamily protein
AT2G37430.1	C2H2 and C2HC zinc fingers superfamily protein
AT4G24350.1	Phosphorylase superfamily protein
AT3G11210.1	SGNH hydrolase-type esterase superfamily protein
AT3G54070.1	Ankyrin repeat family protein
AT1G10200 WLIM1	GATA type zinc finger transcription factor family protein
AT3G15518.1	
AT1G75390 AtbZIP44,	basic leucine-zipper 44
AT3G17210 ATHS1,HS1	heat stable protein 1
AT2G22540 AGL22,SVP	K-box region and MADS-box transcription factor family protein
AT4G27300.1	S-locus lectin protein kinase family protein
AT2G30540.1	Thioredoxin superfamily protein
AT1G53270.1	ABC-2 type transporter family protein
AT4G21440 ATM4,ATMY	MYB-like 102
AT1G28680.1	HXXXD-type acyl-transferase family protein
AT4G28380.1	Leucine-rich repeat (LRR) family protein
AT1G74960 ATKAS2,FA	fatty acid biosynthesis 1
AT4G24130.1	Protein of unknown function, DUF538
AT5G54980.1	Uncharacterised protein family (UPF0497)
AT2G40320 TBL33	TRICHOME BIREFRINGENCE-LIKE 33
AT5G07330.1	
AT5G19410.1	ABC-2 type transporter family protein
AT3G23590 MED33A,RF	REF4-related 1
AT1G04220 KCS2	3-ketoacyl-CoA synthase 2
AT5G67210.1	Protein of unknown function (DUF579)
AT3G53720 ATCHX20,C	cation/H+ exchanger 20
AT3G04810 ATNEK2,NE	NIMA-related kinase 2
AT3G24770 CLE41	CLAVATA3/ESR-RELATED 41
AT1G14550.1	Peroxidase superfamily protein
AT3G54240.1	alpha/beta-Hydrolases superfamily protein
AT2G40260.1	Homeodomain-like superfamily protein
AT2G30490 ATC4H,C4H	cinnamate-4-hydroxylase
AT3G18670.1	Ankyrin repeat family protein
AT1G24620.1	EF hand calcium-binding protein family
AT3G46130 ATMYB48,A	myb domain protein 48
AT3G18660 GUX1,PGSI	plant glycogenin-like starch initiation protein 1
AT5G42650 AOS,CYP74	allene oxide synthase
AT2G24430 ANAC038,A	NAC domain containing protein 38
AT1G02040.1	C2H2-type zinc finger family protein
AT3G62730.1	
AT5G08640 ATFLS1,FL	flavonol synthase 1
AT2G05990 ENR1,MOD1	NAD(P)-binding Rossmann-fold superfamily protein
AT4G37370 CYP81D8	cytochrome P450, family 81, subfamily D, polypeptide 8
AT2G26660 ATSPX2,SP	SPX domain gene 2
AT1G07080.1	Thioredoxin superfamily protein
AT5G54690 GAUT12,IR	galacturonosyltransferase 12
AT5G05110.1	Cystatin/monellin family protein
AT2G37170 PIP2;2,PI	plasma membrane intrinsic protein 2
AT1G70840 MLP31	MLP-like protein 31

AT2G42440.1		Lateral organ boundaries (LOB) domain family protein
AT2G38120	AUX1,MAP1	Transmembrane amino acid transporter family protein
AT5G05390	LAC12	laccase 12
AT4G35160.1		O-methyltransferase family protein
AT5G01520.1		RING/U-box superfamily protein
AT2G46130	ATWRKY43,	WRKY DNA-binding protein 43
AT2G42360.1		RING/U-box superfamily protein
AT5G16740.1		Transmembrane amino acid transporter family protein
AT1G03010.1		Phototropic-responsive NPH3 family protein
AT5G41800.1		Transmembrane amino acid transporter family protein
AT5G16770	AtMYB9,MY	myb domain protein 9
AT1G65690.1		Late embryogenesis abundant (LEA) hydroxyproline-rich glycoprot
AT1G17950	ATMYB52,B	myb domain protein 52
AT5G64260	EXL2	EXORDIUM like 2
AT1G73220	1-Oct,AtC	organic cation/carnitine transporter1
AT4G05150.1		Octicosapeptide/Phox/Bem1p family protein
AT2G20080.1		
AT1G74460.1		GDSL-like Lipase/Acylhydrolase superfamily protein
AT2G37750.1		
AT4G24140.1		alpha/beta-Hydrolases superfamily protein
AT2G28315.1		Nucleotide/sugar transporter family protein
AT5G14740	BETA CA2,	carbonic anhydrase 2
AT2G20142.1		Toll-Interleukin-Resistance (TIR) domain family protein
AT5G09220	AAP2	amino acid permease 2
AT1G24625	ZFP7	zinc finger protein 7
AT1G09530	PAP3,PIF3	phytochrome interacting factor 3
AT2G32800	AP4.3A	protein kinase family protein
AT5G41040.1		HXXXD-type acyl-transferase family protein
AT3G28917	MIF2	mini zinc finger 2
AT4G31540	ATEXO70G1	exocyst subunit exo70 family protein G1
AT2G40390.1		
AT4G35040	bZIP19	Basic-leucine zipper (bZIP) transcription factor family protein
AT5G01225.1		
AT2G38800.1		Plant calmodulin-binding protein-related
AT2G32830	PHT1;5,PH	phosphate transporter 1;5
AT5G22930.1		Protein of unknown function (DUF1635)
AT4G12300	CYP706A4	cytochrome P450, family 706, subfamily A, polypeptide 4
AT3G18400	anac058,N	NAC domain containing protein 58
AT4G32940	GAMMA-VPE	gamma vacuolar processing enzyme
AT1G67260	TCP1	TCP family transcription factor
AT2G30590	WRKY21	WRKY DNA-binding protein 21
AT4G35550	ATWOX13,H	WUSCHEL related homeobox 13
AT4G25810	XTH23,XTR	xyloglucan endotransglycosylase 6
AT4G37770	ACS8	1-amino-cyclopropane-1-carboxylate synthase 8
AT5G61430	ANAC100,A	NAC domain containing protein 100
AT1G22710	ATSUC2,SU	sucrose-proton symporter 2
AT3G28960.1		Transmembrane amino acid transporter family protein
AT4G01070	GT72B1,UG	UDP-Glycosyltransferase superfamily protein
AT4G08950	EXO	Phosphate-responsive 1 family protein
AT5G04930	ALA1	aminophospholipid ATPase 1
AT2G21180.1		
AT5G49350.1		Glycine-rich protein family
AT1G68470.1		Exostosin family protein
AT2G39690.1		Protein of unknown function, DUF547

AT2G29110	ATGLR2.8,	glutamate receptor 2.8
AT3G10120.1		
AT2G03220	ATFT1,ATF	fucosyltransferase 1
AT2G40370	LAC5	laccase 5
AT4G17220	ATMAP70-5	microtubule-associated proteins 70-5
AT5G53750.1		CBS domain-containing protein
AT2G39350.1		ABC-2 type transporter family protein
AT1G71380	ATCEL3,AT	cellulase 3
AT5G44400.1		FAD-binding Berberine family protein
AT4G27290.1		S-locus lectin protein kinase family protein
AT5G50790.1		Nodulin MtN3 family protein
AT3G27400.1		Pectin lyase-like superfamily protein
AT1G05260	RCI3,RCI3	Peroxidase superfamily protein
AT4G12350	AtMYB42,M	myb domain protein 42
AT5G62350.1		Plant invertase/pectin methylesterase inhibitor superfamily pro
AT5G09760.1		Plant invertase/pectin methylesterase inhibitor superfamily
AT1G69500	CYP704B1	cytochrome P450, family 704, subfamily B, polypeptide 1
AT1G56600	AtGolS2,G	galactinol synthase 2
AT3G26320	CYP71B36	cytochrome P450, family 71, subfamily B, polypeptide 36
AT5G03760	ATCSLA09,	Nucleotide-diphospho-sugar transferases superfamily protein
AT1G27730	STZ,ZAT10	salt tolerance zinc finger
AT5G10180	AST68,SUL	slufate transporter 2;1
AT3G12720	ATMYB67,A	myb domain protein 67
AT1G63910	AtMYB103,	myb domain protein 103
AT5G16740.1		Transmembrane amino acid transporter family protein
AT5G10695.1		
AT4G38560.2		Arabidopsis phospholipase-like protein (PEARLI 4) family
AT3G22800.1		Leucine-rich repeat (LRR) family protein
AT1G33760.1		Integrase-type DNA-binding superfamily protein
AT2G16230.1		O-Glycosyl hydrolases family 17 protein
AT4G21390	B120	S-locus lectin protein kinase family protein
AT4G32870.1		Polyketide cyclase/dehydrase and lipid transport superfamily pr
AT1G07120.1		
AT3G25400.1		
AT4G33467.2		
AT4G27280.1		Calcium-binding EF-hand family protein
AT4G03270	CYCD6;1	Cyclin D6;1
AT1G49960.1		Xanthine/uracil permease family protein
AT3G12720	ATMYB67,A	myb domain protein 67
AT5G24870.1		RING/U-box superfamily protein
AT5G56350.1		Pyruvate kinase family protein
AT2G37090	IRX9	Nucleotide-diphospho-sugar transferases superfamily protein
AT3G01470	ATHB-1,AT	homeobox 1
AT1G17840	ABCG11,AT	white-brown complex homolog protein 11
AT3G60120	BGLU27	beta glucosidase 27
AT3G54070.1		Ankyrin repeat family protein
AT2G21100.1		Disease resistance-responsive (dirigent-like protein) family pr
AT2G26640	KCS11	3-ketoacyl-CoA synthase 11
AT1G50420	SCL-3,SCL	scarecrow-like 3
AT1G61110	anac025,N	NAC domain containing protein 25

AT3G03700.1		Plasma-membrane choline transporter family protein
AT1G70260.1		nodulin MtN21 /EamA-like transporter family protein
AT5G58350	WNK4,ZIK2	with no lysine (K) kinase 4
AT5G56340	ATCRT1	RING/U-box superfamily protein
AT2G18370.1		Bifunctional inhibitor/lipid-transfer protein/seed storage 2S a
AT1G24140.1		Matrixin family protein
AT3G02040	SRG3	senescence-related gene 3
AT2G37360.1		ABC-2 type transporter family protein
AT2G20560.1		DNAJ heat shock family protein
AT1G03220.1		Eukaryotic aspartyl protease family protein
AT5G10770.1		Eukaryotic aspartyl protease family protein
AT4G15550	IAGLU	indole-3-acetate beta-D-glucosyltransferase
AT3G16520	UGT88A1	UDP-glucosyl transferase 88A1
AT1G02520	PGP11	P-glycoprotein 11
AT2G32030.1		Acyl-CoA N-acyltransferases (NAT) superfamily protein
AT1G58190	AtRLP9,RL	receptor like protein 9
AT3G23430	ATPHO1,PH	phosphate 1
AT1G27170.1		transmembrane receptors;ATP binding
AT3G23250	ATMYB15,A	myb domain protein 15
AT1G65730	YSL7	YELLOW STRIPE like 7
AT5G17230	PSY	PHYTOENE SYNTHASE
AT4G27300.1		S-locus lectin protein kinase family protein
AT3G52970	CYP76G1	cytochrome P450, family 76, subfamily G, polypeptide 1
AT3G22840	ELIP,ELIP	Chlorophyll A-B binding family protein
AT5G06080	LBD33	LOB domain-containing protein 33
AT1G72200.1		RING/U-box superfamily protein
AT3G07990	SCPL27	serine carboxypeptidase-like 27
AT4G24050.1		NAD(P)-binding Rossmann-fold superfamily protein
AT1G22400	ATUGT85A1	UDP-Glycosyltransferase superfamily protein
AT3G12060	TBL1	Plant protein of unknown function (DUF828)
AT3G28857.1		basic helix-loop-helix (bHLH) DNA-binding family protein
AT3G27150.1		Galactose oxidase/kelch repeat superfamily protein
AT3G21710.2		
AT1G55790.1		Domain of unknown function (DUF2431)
AT1G55020	ATLOX1,LO	lipoxygenase 1
AT3G07940.1		Calcium-dependent ARF-type GTPase activating protein family
AT2G25625.2		
AT3G13610.1		2-oxoglutarate (2OG) and Fe(II)-dependent oxygenase superfamily
AT1G52190.1		Major facilitator superfamily protein
AT5G65090	BST1,DER4	DNAse I-like superfamily protein
AT2G01340	At17.1	
AT4G27220.1		NB-ARC domain-containing disease resistance protein
AT3G59850.1		Pectin lyase-like superfamily protein
AT3G16520	UGT88A1	UDP-glucosyl transferase 88A1
AT5G14650.1		Pectin lyase-like superfamily protein
AT3G21240	4CL2,AT4C	4-coumarate:CoA ligase 2
AT4G35160.1		O-methyltransferase family protein
AT1G43800.1		Plant stearoyl-acyl-carrier-protein desaturase family protein
AT1G53270.1		ABC-2 type transporter family protein
AT1G58602.2		LRR and NB-ARC domains-containing disease resistance protein

AT2G14520.1	CBS domain-containing protein with a domain of unknown function
AT5G08280HEMC	hydroxymethylbilane synthase
AT4G21060.2	Galactosyltransferase family protein
AT1G58290HEMA1	Glutamyl-tRNA reductase family protein
AT5G24550BGLU32	beta glucosidase 32
AT1G69530AT-EXP1,A	expansin A1
AT2G05990ENR1,MOD1	NAD(P)-binding Rossmann-fold superfamily protein
AT1G52700.1	alpha/beta-Hydrolases superfamily protein
AT2G20580ATRPN1A,R	26S proteasome regulatory subunit S2 1A
AT2G03090ATEXP15,A	expansin A15
AT2G31400GUN1	genomes uncoupled 1
AT2G04280.1	
AT3G29635.1	HXXXD-type acyl-transferase family protein
AT1G67740PSBY,YCF3	photosystem II BY

AT1G15760.1	Sterile alpha motif (SAM) domain-containing protein
AT3G61470LHCA2	photosystem I light harvesting complex gene 2
AT3G48560AHAS,ALS,	chlorsulfuron/imidazolinone resistant 1
AT5G35550ATMYB123,	Duplicated homeodomain-like superfamily protein
AT3G53580.1	diaminopimelate epimerase family protein
AT3G04290ATLTL1,LT	Li-tolerant lipase 1
AT3G12750ZIP1	zinc transporter 1 precursor
AT1G09690.1	Translation protein SH3-like family protein
AT3G05950.1	RmlC-like cupins superfamily protein
AT1G77280.1	Protein kinase protein with adenine nucleotide alpha hydrolases
AT5G58600PMR5,TBL4	Plant protein of unknown function (DUF828)
AT4G25050ACP4	acyl carrier protein 4
AT1G78060.1	Glycosyl hydrolase family protein
AT3G27060ATTSO2,TS	Ferritin/ribonucleotide reductase-like family protein
AT1G80480PTAC17	plastid transcriptionally active 17
AT2G28190CSD2,CZSC	copper/zinc superoxide dismutase 2
AT1G78380ATGSTU19,	glutathione S-transferase TAU 19
AT5G23570ATSGS3,SG	XS domain-containing protein / XS zinc finger domain-containing
AT1G18450ARP4,ATAR	actin-related protein 4
AT4G19710AK-HSDH,A	aspartate kinase-homoserine dehydrogenase ii
AT3G58100PDCB5	plasmodesmata callose-binding protein 5
AT2G45420LBD18	LOB domain-containing protein 18
AT1G67740PSBY,YCF3	photosystem II BY
AT5G03940 54CP,CPSR	chloroplast signal recognition particle 54 kDa subunit
AT4G16410.1	

AT5G42930.1	alpha/beta-Hydrolases superfamily protein
AT4G39210APL3	Glucose-1-phosphate adenylyltransferase family protein
AT5G26670.1	Pectinacetylesterase family protein
AT5G64040PSAN	photosystem I reaction center subunit PSI-N, chloroplast, putat
AT1G71480.1	Nuclear transport factor 2 (NTF2) family protein
AT3G57270BG1	beta-1,3-glucanase 1
AT5G02500AT-HSC70-	heat shock cognate protein 70-1
AT3G63190AtcpRRF,c	ribosome recycling factor, chloroplast precursor
AT3G14900.1	
AT5G54010.1	UDP-Glycosyltransferase superfamily protein
AT2G46210.1	Fatty acid/sphingolipid desaturase
AT2G34430LHB1B1,LH	light-harvesting chlorophyll-protein complex II subunit B1
AT3G18420.1	Protein prenylyltransferase superfamily protein

AT3G51860	ATCAX3,AT	cation exchanger 3
AT1G52360.1		Coatomer, beta\' subunit
AT2G48020.1		Major facilitator superfamily protein
AT4G28250	ATEXPB3,A	expansin B3
AT2G39730	RCA	rubisco activase
AT4G23990	ATCSLG3,C	cellulose synthase like G3
AT5G03260	LAC11	laccase 11
AT4G02290	AtGH9B13,	glycosyl hydrolase 9B13
AT3G27750.1		
AT5G56000	AtHsp90.4	HEAT SHOCK PROTEIN 81.4
AT3G29030	ATEXP5,AT	expansin A5
AT3G46780	PTAC16	plastid transcriptionally active 16
AT4G21990	APR3,ATAP	APS reductase 3
AT5G58930.1		Protein of unknown function (DUF740)
AT5G45160.1		Root hair defective 3 GTP-binding protein (RHD3)
AT2G22590.1		UDP-Glycosyltransferase superfamily protein
AT2G05990	ENR1,MOD1	NAD(P)-binding Rossmann-fold superfamily protein
AT1G77000	ATSKP2;2,	RNI-like superfamily protein
AT2G13360	AGT,AGT1,	alanine:glyoxylate aminotransferase
AT2G30950	FTSH2,VAR	FtsH extracellular protease family
AT3G28920	AtHB34,HB	homeobox protein 34
AT1G78630	emb1473	Ribosomal protein L13 family protein
AT5G06300.1		Putative lysine decarboxylase family protein
AT3G47570.1		Leucine-rich repeat protein kinase family protein
AT1G09890.1		Rhamnogalacturonate lyase family protein
AT5G33370.1		GDSL-like Lipase/Acylhydrolase superfamily protein
AT5G17020	ATCRM1,AT	exportin 1A
AT1G11600	CYP77B1	cytochrome P450, family 77, subfamily B, polypeptide 1
AT1G74670.1		Gibberellin-regulated family protein
AT3G55330	PPL1	PsbP-like protein 1
AT5G42020	BIP,BIP2	Heat shock protein 70 (Hsp 70) family protein
AT1G74160.1		
AT4G21990	APR3,ATAP	APS reductase 3
AT5G20740.1		Plant invertase/pectin methylesterase inhibitor superfamily pro
AT5G13700	APAO,ATPA	polyamine oxidase 1
AT5G25880	ATNADP-ME	NADP-malic enzyme 3
AT1G15820	CP24,LHCB	light harvesting complex photosystem II subunit 6
AT3G04720	HEL,PR-4,	pathogenesis-related 4
AT3G01680.1		
AT1G72640.1		NAD(P)-binding Rossmann-fold superfamily protein
AT2G17420	ATNTRA,NT	NADPH-dependent thioredoxin reductase A
AT1G70100.3		
AT5G46050	ATPTR3,PT	peptide transporter 3
AT2G37640	ATEXP3,AT	Barwin-like endoglucanases superfamily protein
AT1G30380	PSAK	photosystem I subunit K
AT4G39230.1		NmrA-like negative transcriptional regulator family protein
AT3G02090	MPPBETA	Insulinase (Peptidase family M16) protein
AT1G29040.1		
AT4G38710.2		glycine-rich protein
AT1G21600	PTAC6	plastid transcriptionally active 6
AT5G62790	DXR,PDE12	1-deoxy-D-xylulose 5-phosphate reductoisomerase
AT5G08610.1		P-loop containing nucleoside triphosphate hydrolases superfamil
AT3G26380.1		Melibiase family protein

AT2G37460.1		nodulin MtN21 /EamA-like transporter family protein
AT4G38460	GGR	geranylgeranyl reductase
AT2G33847.1		
AT1G11770.1		FAD-binding Berberine family protein
AT1G60420.1		DC1 domain-containing protein
AT2G46170.1		Reticulon family protein
AT2G22840	AtGRF1,GR	growth-regulating factor 1
AT3G17180	scpl33	serine carboxypeptidase-like 33
AT5G07990	CYP75B1,D	Cytochrome P450 superfamily protein
AT5G45470.1		Protein of unknown function (DUF594)
AT2G10940.1		Bifunctional inhibitor/lipid-transfer protein/seed storage 2S a
AT1G68010	ATHPR1,HP	hydroxypyruvate reductase
AT5G40950	RPL27	ribosomal protein large subunit 27
AT1G11860.3		Glycine cleavage T-protein family
AT3G04760.1		Pentatricopeptide repeat (PPR-like) superfamily protein
AT4G39260	ATGRP8,CC	cold, circadian rhythm, and RNA binding 1
AT5G67090.1		Subtilisin-like serine endopeptidase family protein
AT4G04640	ATPC1	ATPase, F1 complex, gamma subunit protein
AT3G13650.1		Disease resistance-responsive (dirigent-like protein) family pr
AT5G06290	2-Cys Prx	2-cysteine peroxiredoxin B
AT5G07990	CYP75B1,D	Cytochrome P450 superfamily protein
AT3G50685.1		
AT1G29670.1		GDSL-like Lipase/Acylhydrolase superfamily protein
AT3G55440	ATCTIMC,C	triosephosphate isomerase
AT4G37580	COP3,HLS1	Acyl-CoA N-acyltransferases (NAT) superfamily protein
AT4G20940.1		Leucine-rich receptor-like protein kinase family protein
AT5G13510.1		Ribosomal protein L10 family protein
AT1G51110.1		Plastid-lipid associated protein PAP / fibrillin family protein
AT4G37925	NDH-M	subunit NDH-M of NAD(P)H:plastoquinone dehydrogenase complex
AT4G38380.1		MATE efflux family protein
AT4G02510	ATTOC159,	translocon at the outer envelope membrane of chloroplasts 159
AT3G22670.1		Pentatricopeptide repeat (PPR) superfamily protein
AT4G05530	IBR1,SDRA	indole-3-butyric acid response 1
AT2G06520	PSBX	photosystem II subunit X
AT1G27680	APL2	ADPGLC-PPase large subunit
AT4G18030.1		S-adenosyl-L-methionine-dependent methyltransferases superfamil
AT1G56580	SVB	Protein of unknown function, DUF538
AT1G65230.1		Uncharacterized conserved protein (DUF2358)
AT1G54690	G-H2AX,GA	gamma histone variant H2AX
AT1G75270	DHAR2	dehydroascorbate reductase 2
AT4G22140	EBS	PHD finger family protein / bromo-adjacent homology (BAH) domai
AT2G28790.1		Pathogenesis-related thaumatin superfamily protein
AT1G07930.1		GTP binding Elongation factor Tu family protein
AT1G58120.1		
AT1G79040	PSBR	photosystem II subunit R
AT1G72480.1		Lung seven transmembrane receptor family protein
AT4G34770.1		SAUR-like auxin-responsive protein family
AT1G34575.1		FAD-binding Berberine family protein
AT2G36530	ENO2,LOS2	Enolase
AT1G74470.1		Pyridine nucleotide-disulphide oxidoreductase family protein
AT5G18660	PCB2	NAD(P)-binding Rossmann-fold superfamily protein
AT1G06680	OE23,OEE2	photosystem II subunit P-1
AT5G01930.1		Glycosyl hydrolase superfamily protein
AT2G26250	FDH,KCS10	3-ketoacyl-CoA synthase 10

AT5G48930	HCT	hydroxycinnamoyl-CoA shikimate/quinate hydroxycinnamoyl transfe
AT1G18650	PDCB3	plasmodesmata callose-binding protein 3
AT2G23820.2		Metal-dependent phosphohydrolase
AT3G22330	ATRH53,PM	putative mitochondrial RNA helicase 2
AT3G20820.1		Leucine-rich repeat (LRR) family protein
AT1G74970	RPS9,TWN3	ribosomal protein S9
AT5G63810	BGAL10	beta-galactosidase 10
AT3G05600.1		alpha/beta-Hydrolases superfamily protein
AT5G43260.1		chaperone protein dnaJ-related
AT4G13710.1		Pectin lyase-like superfamily protein
AT2G33800.1		Ribosomal protein S5 family protein
AT3G25700.1		Eukaryotic aspartyl protease family protein
AT4G37300	MEE59	maternal effect embryo arrest 59
AT5G13710	CPH,SMT1	sterol methyltransferase 1
AT3G17700	ATCNGC20,	cyclic nucleotide-binding transporter 1
AT3G03620.1		MATE efflux family protein
AT1G32990	PRPL11	plastid ribosomal protein l11
AT5G19160	TBL11	TRICHOME BIREFRINGENCE-LIKE 11
AT1G53240	mMDH1	Lactate/malate dehydrogenase family protein
AT5G51820	ATPGMP,PG	phosphoglucomutase
AT3G62630.1		Protein of unknown function (DUF1645)
AT1G06680	OE23,OEE2	photosystem II subunit P-1
AT1G72610	ATGER1,GE	germin-like protein 1
AT5G08060.1		
AT3G02350	GAUT9	galacturonosyltransferase 9
AT5G65220.1		Ribosomal L29 family protein
AT2G48070	RPH1	resistance to phytophthora 1
AT1G72970	EDA17,HTH	Glucose-methanol-choline (GMC) oxidoreductase family protein
AT3G19000.1		2-oxoglutarate (2OG) and Fe(II)-dependent oxygenase superfamily
AT5G35220	EGY1	Peptidase M50 family protein
AT4G34090.1		
AT3G03900	APK3	adenosine-5\'-phosphosulfate (APS) kinase 3
AT4G31810.1		ATP-dependent caseinolytic (Clp) protease/crotonase family prot
AT1G29670.1		GDSL-like Lipase/Acylhydrolase superfamily protein
AT3G12780	PGK1	phosphoglycerate kinase 1
AT3G27740	CARA	carbamoyl phosphate synthetase A
AT5G48850	ATSDI1	Tetratricopeptide repeat (TPR)-like superfamily protein
AT5G05170	ATCESA3,A	Cellulose synthase family protein
AT3G52150.2		RNA-binding (RRM/RBD/RNP motifs) family protein
AT3G23990	HSP60,HSP	heat shock protein 60
AT5G64740	CESA6,E11	cellulose synthase 6
AT2G42840	PDF1	protodermal factor 1
AT2G44350	ATCS,CSY4	Citrate synthase family protein
AT5G38610.1		Plant invertase/pectin methylesterase inhibitor superfamily pro
AT2G07050	CAS1	cycloartenol synthase 1
AT3G57490.1		Ribosomal protein S5 family protein
AT4G36360	BGAL3	beta-galactosidase 3
AT5G45950.1		GDSL-like Lipase/Acylhydrolase superfamily protein
AT5G03260	LAC11	laccase 11
AT1G32550.1		2Fe-2S ferredoxin-like superfamily protein
AT3G23990	HSP60,HSP	heat shock protein 60
AT2G29630	PY,THIC	thiaminC
AT4G14550	IAA14,SLR	indole-3-acetic acid inducible 14
AT2G37870.1		Bifunctional inhibitor/lipid-transfer protein/seed storage 2S a

AT2G36870	XTH32	xyloglucan endotransglucosylase/hydrolase 32
AT3G08740.1		elongation factor P (EF-P) family protein
AT3G54890	LHCA1	photosystem I light harvesting complex gene 1
AT1G08200	AXS2	UDP-D-apiose/UDP-D-xylose synthase 2
AT5G22740	ATCSLA02,	cellulose synthase-like A02
AT2G29990	NDA2	alternative NAD(P)H dehydrogenase 2
AT4G12730	FLA2	FASCICLIN-like arabinogalactan 2
AT5G07990	CYP75B1, D	Cytochrome P450 superfamily protein
AT1G80560	ATIMD2, IM	isopropylmalate dehydrogenase 2
AT5G20080.1		FAD/NAD(P)-binding oxidoreductase
AT5G24660	LSU2	response to low sulfur 2
AT1G14810.1		semialdehyde dehydrogenase family protein
AT4G30610	BRS1, SCPL	alpha/beta-Hydrolases superfamily protein
AT1G30520	AAE14	acyl-activating enzyme 14
AT5G30510	ARRPS1, RP	ribosomal protein S1
AT3G45010	scpl48	serine carboxypeptidase-like 48
AT1G77670.1		Pyridoxal phosphate (PLP)-dependent transferases superfamily pr
AT5G52010.1		C2H2-like zinc finger protein
AT5G52580.2		RabGAP/TBC domain-containing protein
AT4G01150.1		
AT2G26560	PLA IIA, P	phospholipase A 2A
AT3G01660.1		S-adenosyl-L-methionine-dependent methyltransferases superfamil
AT3G16810	APUM24, PU	pumilio 24
AT3G58610.3		ketol-acid reductoisomerase
AT3G21250	ATMRP6, MR	multidrug resistance-associated protein 6
AT1G59820	ALA3	aminophospholipid ATPase 3
AT3G04790.1		Ribose 5-phosphate isomerase, type A protein
AT3G54050	HCEF1	high cyclic electron flow 1
AT5G51750	ATSBT1.3,	subtilase 1.3
AT5G46280	MCM3	Minichromosome maintenance (MCM2/3/5) family protein
AT1G48100.1		Pectin lyase-like superfamily protein
AT2G40550	ETG1	E2F target gene 1
AT1G03130	PSAD-2	photosystem I subunit D-2
AT1G09640.1		Translation elongation factor EF1B, gamma chain
AT5G49360	ATBXL1, BX	beta-xylosidase 1
AT1G09390.1		GDSL-like Lipase/Acylhydrolase superfamily protein
AT5G19730.1		Pectin lyase-like superfamily protein
AT2G16050.1		Cysteine/Histidine-rich C1 domain family protein
AT4G17740.1		Peptidase S41 family protein
AT3G21690.1		MATE efflux family protein
AT1G75240	AtHB33, HB	homeobox protein 33
AT4G25130	PMSR4	peptide met sulfoxide reductase 4
AT4G29400.1		Protein of unknown function (DUF3531)
AT1G02205	CER1	Fatty acid hydroxylase superfamily
AT1G54730.2		Major facilitator superfamily protein
AT3G58120	ATBZIP61,	Basic-leucine zipper (bZIP) transcription factor family protein
AT5G13120	ATCYP20-2	cyclophilin 20-2
AT4G01310.1		Ribosomal L5P family protein
AT5G15150	ATHB-3, AT	homeobox 3
AT5G26230.1		
AT1G26355	SP1L1	SPIRAL1-like1
AT5G37600	ATGLN1;1,	glutamine synthase clone R1
AT1G07930.1		GTP binding Elongation factor Tu family protein
AT3G06880.2		Transducin/WD40 repeat-like superfamily protein

AT3G54890	LHCA1	photosystem I light harvesting complex gene 1
AT3G20000	TOM40	translocase of the outer mitochondrial membrane 40
AT2G38820.2		Protein of unknown function (DUF506)
AT3G07770	AtHsp90-6	HEAT SHOCK PROTEIN 89.1
AT2G06925	ATSPLA2-A	Phospholipase A2 family protein
AT3G19000.1		2-oxoglutarate (2OG) and Fe(II)-dependent oxygenase superfamily
AT1G35510.1		O-fucosyltransferase family protein
AT1G03600	PSB27	photosystem II family protein
AT1G43710	emb1075	Pyridoxal phosphate (PLP)-dependent transferases superfamily pr
AT1G30820.1		CTP synthase family protein
AT1G43790	TED6	tracheary element differentiation-related 6
AT1G60950	ATFD2,FED	2Fe-2S ferredoxin-like superfamily protein
AT1G47840	HXK3	hexokinase 3
AT1G31920.1		Tetratricopeptide repeat (TPR)-like superfamily protein
AT1G23460.1		Pectin lyase-like superfamily protein
AT2G24280.1		alpha/beta-Hydrolases superfamily protein
AT4G35640	ATSERAT3;	serine acetyltransferase 3;2
AT4G29060	emb2726	elongation factor Ts family protein
AT5G67090.1		Subtilisin-like serine endopeptidase family protein
AT1G42970	GAPB	glyceraldehyde-3-phosphate dehydrogenase B subunit
AT3G47800.1		Galactose mutarotase-like superfamily protein
AT5G52520	OVA6,PROR	Class II aaRS and biotin synthetases superfamily protein
AT2G04030	AtHsp90.5	Chaperone protein htpG family protein
AT5G15490.1		UDP-glucose 6-dehydrogenase family protein
AT1G05190	emb2394	Ribosomal protein L6 family
AT4G24770	ATRBP31,A	31-kDa RNA binding protein
AT1G61870	PPR336	pentatricopeptide repeat 336
AT5G14460.1		Pseudouridine synthase family protein
AT1G55690.1		Sec14p-like phosphatidylinositol transfer family protein
AT1G80760	NIP6,NIP6	NOD26-like intrinsic protein 6;1
AT3G47470	CAB4,LHCA	light-harvesting chlorophyll-protein complex I subunit A4
AT4G18740.1		Rho termination factor
AT4G35160.1		O-methyltransferase family protein
AT3G03130.1		
AT4G33760.1		tRNA synthetase class II (D, K and N) family protein
AT5G55630	ATKCO1,AT	Outward rectifying potassium channel protein
AT1G04770.1		Tetratricopeptide repeat (TPR)-like superfamily protein
AT4G24430.1		Rhamnogalacturonate lyase family protein
AT1G65910	anac028,N	NAC domain containing protein 28
AT4G14890.1		2Fe-2S ferredoxin-like superfamily protein
AT2G44480	BGLU17	beta glucosidase 17
AT1G54780	TLP18.3	thylakoid lumen 18.3 kDa protein
AT1G15290.1		Tetratricopeptide repeat (TPR)-like superfamily protein
AT4G20360	ATRAB8D,A	RAB GTPase homolog E1B
AT5G04660	CYP77A4	cytochrome P450, family 77, subfamily A, polypeptide 4
AT3G63140	CSP41A	chloroplast stem-loop binding protein of 41 kDa
AT2G05070	LHCB2,LHC	photosystem II light harvesting complex gene 2.2
AT1G14890.1		Plant invertase/pectin methylesterase inhibitor superfamily pro
AT1G31330	PSAF	photosystem I subunit F
AT4G24220	AWI31,VEP	NAD(P)-binding Rossmann-fold superfamily protein
AT3G20820.1		Leucine-rich repeat (LRR) family protein
AT5G54270	LHCB3,LHC	light-harvesting chlorophyll B-binding protein 3
AT1G60060.1		Serine/threonine-protein kinase WNK (With No Lysine)-related

AT1G05310.1		Pectin lyase-like superfamily protein
AT1G64660	ATMGL,MGL	methionine gamma-lyase

AT5G20630	ATGER3,GE	germin 3
AT3G57560	NAGK	N-acetyl-l-glutamate kinase
AT5G48960.1		HAD-superfamily hydrolase, subfamily IG, 5\'-nucleotidase
AT5G12470.1		Protein of unknown function (DUF3411)
AT3G19000.1		2-oxoglutarate (2OG) and Fe(II)-dependent oxygenase superfamily
AT4G37930	SHM1,SHMT	serine transhydroxymethyltransferase 1
AT4G32890	GATA9	GATA transcription factor 9
AT5G05580	FAD8	fatty acid desaturase 8
AT1G50450.1		Saccharopine dehydrogenase
AT1G19580	GAMMA CA1	gamma carbonic anhydrase 1
AT1G35680.1		Ribosomal protein L21
AT4G25250.1		Plant invertase/pectin methylesterase inhibitor superfamily pro
AT1G79850	CS17,PRPS	ribosomal protein S17
AT2G15000.2		
AT2G39770	CYT1,EMB1	Glucose-1-phosphate adenylyltransferase family protein
AT4G27070	TSB2	tryptophan synthase beta-subunit 2
AT5G23860	TUB8	tubulin beta 8
AT5G23940	DCR,EMB30	HXXXD-type acyl-transferase family protein

AT3G54690.1		Sugar isomerase (SIS) family protein
AT1G70370	PG2	polygalacturonase 2
AT1G06950	ATTIC110,	translocon at the inner envelope membrane of chloroplasts 110
AT3G08940	LHCB4.2	light harvesting complex photosystem II
AT5G13090.1		
AT4G29020.2		glycine-rich protein
AT3G52140.2		tetratricopeptide repeat (TPR)-containing protein
AT4G24750.1		Rhodanese/Cell cycle control phosphatase superfamily protein
AT5G54600.1		Translation protein SH3-like family protein
AT1G74970	RPS9,TWN3	ribosomal protein S9
AT1G10070	ATBCAT-2,	branched-chain amino acid transaminase 2
AT2G42320.2		nucleolar protein gar2-related
AT5G10250	DOT3	Phototropic-responsive NPH3 family protein
AT1G64660	ATMGL,MGL	methionine gamma-lyase
AT3G07670.1		Rubisco methyltransferase family protein
AT5G14060	CARAB-AK-	Aspartate kinase family protein
AT4G31990	AAT3,ASP5	aspartate aminotransferase 5
AT1G55670	PSAG	photosystem I subunit G
AT4G38970	FBA2	fructose-bisphosphate aldolase 2
AT2G43750	ACS1,ATCS	O-acetylserine (thiol) lyase B
AT5G22430.1		Pollen Ole e 1 allergen and extensin family protein
AT5G11720.1		Glycosyl hydrolases family 31 protein
AT2G04160	AIR3	Subtilisin-like serine endopeptidase family protein
AT5G51100	FSD2	Fe superoxide dismutase 2
AT2G27920	SCPL51	serine carboxypeptidase-like 51
AT5G16030.1		
AT1G48350.1		Ribosomal L18p/L5e family protein
AT5G59845.1		Gibberellin-regulated family protein
AT4G31990	AAT3,ASP5	aspartate aminotransferase 5
AT1G74710	ATICS1,ED	ADC synthase superfamily protein
AT5G07020.1		proline-rich family protein

AT2G43030.1		Ribosomal protein L3 family protein
AT4G27070	TSB2	tryptophan synthase beta-subunit 2
AT3G52500.1		Eukaryotic aspartyl protease family protein
AT2G22600.1		RNA-binding KH domain-containing protein
AT2G36250	ATFTSZ2-1	Tubulin/FtsZ family protein
AT5G18170	GDH1	glutamate dehydrogenase 1
AT5G44000.1		Glutathione S-transferase family protein
AT5G60020	ATLAC17,L	laccase 17
AT1G70310	SPDS2	spermidine synthase 2
AT2G40490	HEME2	Uroporphyrinogen decarboxylase
AT1G48600	AtPMEAMT,	S-adenosyl-L-methionine-dependent methyltransferases superfamil
AT5G61640	ATMSRA1,P	peptidemethionine sulfoxide reductase 1
AT4G24190	AtHsp90-7	Chaperone protein htpG family protein
AT1G74310	ATHSP101,	heat shock protein 101
AT4G22720.1		Actin-like ATPase superfamily protein
AT3G10060.1		FKBP-like peptidyl-prolyl cis-trans isomerase family protein
AT5G02160.1		
AT1G09560	GLP5	germin-like protein 5
AT1G14360	ATUTR3,UT	UDP-galactose transporter 3
AT1G08380	PSAO	photosystem I subunit O
AT3G25140	GAUT8,QUA	Nucleotide-diphospho-sugar transferases superfamily protein
AT3G46780	PTAC16	plastid transcriptionally active 16
AT1G76100	PETE1	plastocyanin 1
AT5G08540.1		
AT4G02530.1		chloroplast thylakoid lumen protein
AT1G19190.1		alpha/beta-Hydrolases superfamily protein
AT1G15140.1		FAD/NAD(P)-binding oxidoreductase
AT4G11650	ATOSM34,O	osmotin 34
AT4G33110.2		S-adenosyl-L-methionine-dependent methyltransferases superfamil
AT1G64510.1		Translation elongation factor EF1B/ribosomal protein S6 family
AT5G07990	CYP75B1,D	Cytochrome P450 superfamily protein
AT4G38770	ATPRP4,PR	proline-rich protein 4
AT4G00430	PIP1;4,PI	plasma membrane intrinsic protein 1;4
AT2G42840	PDF1	protodermal factor 1
AT5G42765.1		
AT3G11560.4		LETM1-like protein
AT3G26060	ATPRX Q	Thioredoxin superfamily protein
AT4G13340.1		Leucine-rich repeat (LRR) family protein
AT5G43060.1		Granulin repeat cysteine protease family protein
AT4G27290.1		S-locus lectin protein kinase family protein
AT1G06690.1		NAD(P)-linked oxidoreductase superfamily protein
AT2G02880.1		mucin-related
AT2G26340.1		
AT2G26580	YAB5	plant-specific transcription factor YABBY family protein
AT3G44540	FAR4	fatty acid reductase 4
AT4G01050	TROL	thylakoid rhodanese-like
AT4G25960	PGP2	P-glycoprotein 2
AT2G38185.1		RING/U-box superfamily protein
AT3G25920	RPL15	ribosomal protein L15
AT1G22630.1		
AT1G08550	AVDE1,NPQ	non-photochemical quenching 1
AT1G80600	WIN1	HOPW1-1-interacting 1
AT5G20710	BGAL7	beta-galactosidase 7
AT3G12780	PGK1	phosphoglycerate kinase 1

AT2G04570.1 GDSL-like Lipase/Acylhydrolase superfamily protein
AT3G54950PLA IIIA, patatin-like protein 6
AT1G04770.1 Tetratricopeptide repeat (TPR)-like superfamily protein
AT5G66190ATLFNR1,F ferredoxin-NADP(+)-oxidoreductase 1
AT4G34200 EDA9 D-3-phosphoglycerate dehydrogenase
AT5G25880ATNADP-ME NADP-malic enzyme 3
AT5G39570.1
AT3G51160GMD2,MUR1 NAD(P)-binding Rossmann-fold superfamily protein
AT5G54270LHCB3,LHC light-harvesting chlorophyll B-binding protein 3
AT5G09970 CYP78A7 cytochrome P450, family 78, subfamily A, polypeptide 7
AT5G60020ATLAC17,L laccase 17
AT4G01800AGY1,Atcp Albino or Glassy Yellow 1
AT5G01930.1 Glycosyl hydrolase superfamily protein
AT2G36830GAMMA-TIP gamma tonoplast intrinsic protein
AT1G55320 AAE18 acyl-activating enzyme 18
AT3G27830RPL12,RPL ribosomal protein L12-A
AT4G13870ATWEX,ATW Werner syndrome-like exonuclease
AT5G42150.1 Glutathione S-transferase family protein
AT3G54210.1 Ribosomal protein L17 family protein
AT5G23120 HCF136 photosystem II stability/assembly factor, chloroplast (HCF136)
AT1G03630POR C,POR protochlorophyllide oxidoreductase C
AT3G07620.1 Exostosin family protein
AT4G01150.1
AT4G23990ATCSLG3,C cellulose synthase like G3
AT5G38420.1 Ribulose bisphosphate carboxylase (small chain) family protein
AT2G13560 NAD-ME1 NAD-dependent malic enzyme 1
AT3G56910 PSRP5 plastid-specific 50S ribosomal protein 5
AT5G63310ATNDPK2,N nucleoside diphosphate kinase 2
AT4G00230 XSP1 xylem serine peptidase 1
AT3G08510ATPLC2,PL phospholipase C 2
AT3G22890 APS1 ATP sulfurylase 1
AT1G60550DHNS,ECHI enoyl-CoA hydratase/isomerase D
AT4G36420.1 Ribosomal protein L12 family protein
AT1G63680ATMURE,MU acid-amino acid ligases;ligases;ATP binding;ATP binding;ligases
AT2G16440 MCM4 Minichromosome maintenance (MCM2/3/5) family protein
AT1G76160 sks5 SKU5 similar 5
AT4G19410.1 Pectinacetylesterase family protein
AT5G13980.1 Glycosyl hydrolase family 38 protein
AT3G48550.1
AT1G56110 NOP56 homolog of nucleolar protein NOP56
AT1G21500.1
AT5G25610ATRD22,RD BURP domain-containing protein
AT5G02790 GSTL3 Glutathione S-transferase family protein

AT1G79040 PSBR photosystem II subunit R
AT2G42610 LSH10 Protein of unknown function (DUF640)
AT5G57190 PSD2 phosphatidylserine decarboxylase 2
AT5G44680.1 DNA glycosylase superfamily protein
AT1G64680.1
AT3G49940 LBD38 LOB domain-containing protein 38
AT1G18730 NDF6 NDH dependent flow 6
AT3G61490.3 Pectin lyase-like superfamily protein
AT3G48730 GSA2 glutamate-1-semialdehyde 2,1-aminomutase 2
AT3G19820CBB1,DIM, cell elongation protein / DWARF1 / DIMINUTO (DIM)

AT3G45140ATLOX2,LC lipoxygenase 2
AT2G24270ALDH11A3 aldehyde dehydrogenase 11A3
AT4G35350XCP1 xylem cysteine peptidase 1
AT3G15520.1 Cyclophilin-like peptidyl-prolyl cis-trans isomerase family pro
AT1G60420.1 DC1 domain-containing protein
AT5G39740OLI7,RPL5 ribosomal protein L5 B
AT4G34530CIB1 cryptochrome-interacting basic-helix-loop-helix 1
AT4G24220AWI31,VEP NAD(P)-binding Rossmann-fold superfamily protein
AT5G08370AGAL2,AtA alpha-galactosidase 2
AT5G17540.1 HXXXD-type acyl-transferase family protein
AT3G01480ATCYP38,C cyclophilin 38
AT5G10610CYP81K1 cytochrome P450, family 81, subfamily K, polypeptide 1
AT1G32990PRPL11 plastid ribosomal protein l11
AT1G11600CYP77B1 cytochrome P450, family 77, subfamily B, polypeptide 1
AT5G44680.1 DNA glycosylase superfamily protein
AT4G22010sks4 SKU5 similar 4
AT1G20010TUB5 tubulin beta-5 chain
AT4G00570NAD-ME2 NAD-dependent malic enzyme 2
AT3G04880DRT102 DNA-damage-repair/toleration protein (DRT102)
AT2G06520PSBX photosystem II subunit X
AT2G26330ER,QRP1 Leucine-rich receptor-like protein kinase family protein
AT5G48920TED7 tracheary element differentiation-related 7
AT4G36380ROT3 Cytochrome P450 superfamily protein
AT2G44920.2 Tetratricopeptide repeat (TPR)-like superfamily protein
AT5G07990CYP75B1,D Cytochrome P450 superfamily protein
AT3G14420.2 Aldolase-type TIM barrel family protein
AT1G15960ATNRAMP6, NRAMP metal ion transporter 6
AT3G07470.1 Protein of unknown function, DUF538
AT2G29660.1 zinc finger (C2H2 type) family protein
AT2G33420.1 Protein of unknown function (DUF810)
AT2G29730UGT71D1 UDP-glucosyl transferase 71D1
4,GO:0004097,GO:0016491,GO:0008152
AT3G61870.1
AT1G32470.1 Single hybrid motif superfamily protein
AT3G51160GMD2,MUR1 NAD(P)-binding Rossmann-fold superfamily protein
AT4G05180PSBQ,PSBQ photosystem II subunit Q-2
AT5G15110.1 Pectate lyase family protein
AT3G13470.1 TCP-1/cpn60 chaperonin family protein
AT3G14310ATPME3,PM pectin methylesterase 3
AT5G01410ATPDX1,AT Aldolase-type TIM barrel family protein
AT1G45688.1
AT2G32990AtGH9B8,G glycosyl hydrolase 9B8
AT2G28000CH-CPN60A chaperonin-60alpha
AT1G60000.1 RNA-binding (RRM/RBD/RNP motifs) family protein
AT4G09650ATPD ATP synthase delta-subunit gene
AT5G64570ATBXL4,XY beta-D-xylosidase 4
AT1G53240mMDH1 Lactate/malate dehydrogenase family protein
AT5G44680.1 DNA glycosylase superfamily protein
AT1G30700.1 FAD-binding Berberine family protein
AT1G29070.1 Ribosomal protein L34
AT4G01050TROL thylakoid rhodanese-like
AT3G16370.1 GDSL-like Lipase/Acylhydrolase superfamily protein
AT4G21990APR3,ATAP APS reductase 3
AT5G49910cpHsc70-2 chloroplast heat shock protein 70-2

AT4G34215.1	Domain of unknown function (DUF303)
AT3G26060ATPRX Q	Thioredoxin superfamily protein
AT3G52500.1	Eukaryotic aspartyl protease family protein
AT1G09850XBCP3	xylem bark cysteine peptidase 3
AT3G63540.1	Mog1/PsbP/DUF1795-like photosystem II reaction center PsbP fami
AT1G23000.1	Heavy metal transport/detoxification superfamily protein
AT3G54420ATCHITIV,	homolog of carrot EP3-3 chitinase
AT4G01030.1	pentatricopeptide (PPR) repeat-containing protein
AT3G04550.1	
AT1G78955CAMS1	camelliol C synthase 1
AT2G30950FTSH2,VAR	FtsH extracellular protease family
AT4G25150.1	HAD superfamily, subfamily IIIB acid phosphatase
AT5G05690CBB3,CPD,	Cytochrome P450 superfamily protein
AT4G16390.1	pentatricopeptide (PPR) repeat-containing protein
AT4G24770ATRBP31,A	31-kDa RNA binding protein
AT4G31290.1	ChaC-like family protein
AT4G20360ATRAB8D,A	RAB GTPase homolog E1B
AT3G04260PTAC3	plastid transcriptionally active 3
AT2G47140.1	NAD(P)-binding Rossmann-fold superfamily protein
AT5G04200AtMC9,MC9	metacaspase 9
AT5G02190ATASP38,E	Eukaryotic aspartyl protease family protein
AT3G26430.1	GDSL-like Lipase/Acylhydrolase superfamily protein
AT2G23130AGP17,ATA	arabinogalactan protein 17
AT4G13030.1	P-loop containing nucleoside triphosphate hydrolases superfamil
AT1G08590.1	Leucine-rich receptor-like protein kinase family protein
AT3G26650GAPA,GAPA	glyceraldehyde 3-phosphate dehydrogenase A subunit
AT2G18570.1	UDP-Glycosyltransferase superfamily protein
AT4G22890PGR5-LIKE	PGR5-LIKE A
AT3G54770.1	RNA-binding (RRM/RBD/RNP motifs) family protein
AT1G49820ATMTK,MTK	S-methyl-5-thioribose kinase
AT2G37220.1	RNA-binding (RRM/RBD/RNP motifs) family protein
AT4G38970FBA2	fructose-bisphosphate aldolase 2
AT2G39730RCA	rubisco activase
AT5G21430.1	Chaperone DnaJ-domain superfamily protein
AT1G65880BZO1	benzoyloxyglucosinolate 1
AT2G37870.1	Bifunctional inhibitor/lipid-transfer protein/seed storage 2S a
AT3G56940ACSF,CHL2	dicarboxylate diiron protein, putative (Crd1)
AT1G48600AtPMEAMT,	S-adenosyl-L-methionine-dependent methyltransferases superfamil
AT4G21390B120	S-locus lectin protein kinase family protein
AT1G18590ATSOT17,A	sulfotransferase 17
AT5G41460.1	Protein of unknown function (DUF604)
AT5G35630ATGSL1,GL	glutamine synthetase 2
AT1G03600PSB27	photosystem II family protein
AT4G15510.1	Photosystem II reaction center PsbP family protein
AT5G45670.1	GDSL-like Lipase/Acylhydrolase superfamily protein
AT4G25080CHLM	magnesium-protoporphyrin IX methyltransferase
AT1G47490ATRBP47C,	RNA-binding protein 47C
AT5G21105.1	Plant L-ascorbate oxidase
AT2G34430LHB1B1,LH	light-harvesting chlorophyll-protein complex II subunit B1
AT3G01680.1	
AT4G38770ATPRP4,PR	proline-rich protein 4
AT2G05100LHCB2,LHC	photosystem II light harvesting complex gene 2.1
AT2G26500.2	cytochrome b6f complex subunit (petM), putative
AT3G57520AtSIP2,SI	seed imbibition 2

AT3G03680.1		C2 calcium/lipid-binding plant phosphoribosyltransferase family
AT3G15620	UVR3	DNA photolyase family protein
AT1G78630	emb1473	Ribosomal protein L13 family protein
AT5G11720.1		Glycosyl hydrolases family 31 protein
AT1G19940	AtGH9B5,G	glycosyl hydrolase 9B5
AT4G36250	ALDH3F1	aldehyde dehydrogenase 3F1
AT1G17860.1		Kunitz family trypsin and protease inhibitor protein
AT4G31290.1		ChaC-like family protein
AT1G54500.1		Rubredoxin-like superfamily protein
AT3G26650	GAPA,GAPA	glyceraldehyde 3-phosphate dehydrogenase A subunit
AT1G44575	NPQ4,PSBS	Chlorophyll A-B binding family protein
AT2G16850	PIP2;8,PI	plasma membrane intrinsic protein 2;8
AT5G48920	TED7	tracheary element differentiation-related 7
AT4G14030	SBP1	selenium-binding protein 1
AT3G55800	SBPASE	sedoheptulose-bisphosphatase
AT4G05180	PSBQ,PSBQ	photosystem II subunit Q-2
AT4G37870	PCK1,PEPC	phosphoenolpyruvate carboxykinase 1
AT5G12890.1		UDP-Glycosyltransferase superfamily protein
AT2G36870	XTH32	xyloglucan endotransglucosylase/hydrolase 32
AT1G71160	KCS7	3-ketoacyl-CoA synthase 7
AT1G73680	ALPHA DOX	alpha dioxygenase
AT4G36850.1		PQ-loop repeat family protein / transmembrane family protein
AT2G34430	LHB1B1,LH	light-harvesting chlorophyll-protein complex II subunit B1
AT3G14310	ATPME3,PM	pectin methylesterase 3
AT5G58250.1		
AT4G23900.1		Nucleoside diphosphate kinase family protein
AT3G13470.1		TCP-1/cpn60 chaperonin family protein
AT3G45140	ATLOX2,LO	lipoxygenase 2
AT1G31690.1		Copper amine oxidase family protein
AT5G04200	AtMC9,MC9	metacaspase 9
AT3G52870.1		IQ calmodulin-binding motif family protein
AT5G13510.1		Ribosomal protein L10 family protein
AT2G34780	EMB1611,M	maternal effect embryo arrest 22
AT3G02110	scpl25	serine carboxypeptidase-like 25
AT5G66460.1		Glycosyl hydrolase superfamily protein
AT5G12250	TUB6	beta-6 tubulin
AT1G24260	AGL9,SEP3	K-box region and MADS-box transcription factor family protein
AT1G72970	EDA17,HTH	Glucose-methanol-choline (GMC) oxidoreductase family protein
AT4G04830	ATMSRB5,M	methionine sulfoxide reductase B5
AT1G01800.1		NAD(P)-binding Rossmann-fold superfamily protein
AT5G33370.1		GDSL-like Lipase/Acylhydrolase superfamily protein
AT1G74730.1		Protein of unknown function (DUF1118)
AT1G14930.1		Polyketide cyclase/dehydrase and lipid transport superfamily pr
AT3G54420	ATCHITIV,	homolog of carrot EP3-3 chitinase
AT5G03795.1		Exostosin family protein
AT5G66520.1		Tetratricopeptide repeat (TPR)-like superfamily protein
AT2G40610	ATEXP8,AT	expansin A8
AT3G23510.1		Cyclopropane-fatty-acyl-phospholipid synthase
AT2G43560.1		FKBP-like peptidyl-prolyl cis-trans isomerase family protein
AT5G06900	CYP93D1	cytochrome P450, family 93, subfamily D, polypeptide 1
AT5G39080.1		HXXXD-type acyl-transferase family protein
AT1G22150	SULTR1;3	sulfate transporter 1;3
AT1G51400.1		Photosystem II 5 kD protein

AT5G54250	ATCNGC4,C	cyclic nucleotide-gated cation channel 4
AT5G02540.1		NAD(P)-binding Rossmann-fold superfamily protein
AT1G08440.1		Aluminium activated malate transporter family protein
AT1G29050	TBL38	TRICHOME BIREFRINGENCE-LIKE 38
AT5G21920	ATYLMG2,Y	YGGT family protein
AT1G26820	RNS3	ribonuclease 3
AT5G55730	FLA1	FASCICLIN-like arabinogalactan 1
AT5G11420.1		Protein of unknown function, DUF642
AT2G46820	PSAP,PSI-	photosystem I P subunit
AT1G07320	RPL4	ribosomal protein L4
AT1G14430.1		glyoxal oxidase-related protein
AT1G64390	AtGH9C2,G	glycosyl hydrolase 9C2
AT1G76100	PETE1	plastocyanin 1
AT5G45930	CHL I2,CH	magnesium chelatase i2
AT1G29670.1		GDSL-like Lipase/Acylhydrolase superfamily protein
AT5G57030	LUT2	Lycopene beta/epsilon cyclase protein
AT1G24020	MLP423	MLP-like protein 423
AT1G22150	SULTR1;3	sulfate transporter 1;3
AT1G12900	GAPA-2	glyceraldehyde 3-phosphate dehydrogenase A subunit 2
AT2G04030	AtHsp90.5	Chaperone protein htpG family protein
AT3G21360.1		2-oxoglutarate (2OG) and Fe(II)-dependent oxygenase superfamily
AT5G13870	EXGT-A4,X	xyloglucan endotransglucosylase/hydrolase 5
AT3G61430	ATPIP1,PI	plasma membrane intrinsic protein 1A
AT5G04590	SIR	sulfite reductase
AT3G01140	AtMYB106,	myb domain protein 106
AT1G60060.1		Serine/threonine-protein kinase WNK (With No Lysine)-related
AT3G54420	ATCHITIV,	homolog of carrot EP3-3 chitinase
AT2G45290.1		Transketolase
AT5G67070	RALFL34	ralf-like 34
AT1G11545	XTH8	xyloglucan endotransglucosylase/hydrolase 8
AT1G09240	ATNAS3,NA	nicotianamine synthase 3
AT2G36090.1		F-box family protein
AT3G11090	LBD21	LOB domain-containing protein 21
AT2G34430	LHB1B1,LH	light-harvesting chlorophyll-protein complex II subunit B1
AT4G16146.1		cAMP-regulated phosphoprotein 19-related protein
AT1G74470.1		Pyridine nucleotide-disulphide oxidoreductase family protein
AT5G17330	GAD,GAD1	glutamate decarboxylase
AT5G65940	CHY1	beta-hydroxyisobutyryl-CoA hydrolase 1
AT2G33100	ATCSLD1,C	cellulose synthase-like D1
AT1G31040.1		PLATZ transcription factor family protein
AT1G65590	ATHEX1,HE	beta-hexosaminidase 3
AT5G14690.1		
AT1G67750.1		Pectate lyase family protein
AT1G44760.1		Adenine nucleotide alpha hydrolases-like superfamily protein
AT5G42800	DFR,M318,	dihydroflavonol 4-reductase
AT5G54190	PORA	protochlorophyllide oxidoreductase A
AT1G02500	AtSAM1,MA	S-adenosylmethionine synthetase 1
AT2G29630	PY,THIC	thiaminC
AT5G37600	ATGLN1;1,	glutamine synthase clone R1
AT5G05810	ATL43	RING/U-box superfamily protein
AT3G16250	NDF4	NDH-dependent cyclic electron flow 1
AT1G58122	CPuORF45	conserved peptide upstream open reading frame 45
AT5G11420.1		Protein of unknown function, DUF642
AT2G35640.1		Homeodomain-like superfamily protein

AT1G67090	RBCS1A	ribulose bisphosphate carboxylase small chain 1A

AT1G29670.1		GDSL-like Lipase/Acylhydrolase superfamily protein
AT5G60920	COB	COBRA-like extracellular glycosyl-phosphatidyl inositol-anchore
AT2G29760	OTP81	Tetratricopeptide repeat (TPR)-like superfamily protein
AT4G09010	APX4,TL29	ascorbate peroxidase 4
AT2G31280	CPUORF7	conserved peptide upstream open reading frame 7
AT5G54190	PORA	protochlorophyllide oxidoreductase A
AT5G46690	bHLH071	beta HLH protein 71
AT1G67090	RBCS1A	ribulose bisphosphate carboxylase small chain 1A
AT3G24450.1		Heavy metal transport/detoxification superfamily protein
AT5G60030.1		
AT4G00310	EDA8,MEE4	Putative membrane lipoprotein
AT4G04890	PDF2	protodermal factor 2
AT4G10780.1		LRR and NB-ARC domains-containing disease resistance protein
AT1G71691.2		GDSL-like Lipase/Acylhydrolase superfamily protein
AT3G14360.1		alpha/beta-Hydrolases superfamily protein
AT5G65940	CHY1	beta-hydroxyisobutyryl-CoA hydrolase 1
AT3G52820	ATPAP22,P	purple acid phosphatase 22

y protein

protein

syltransferase family protein

lbumin superfamily protein

.y protein

r protein

fer family protein

albumin superfamily protein

albumin superfamily protein

family protein

lbumin superfamily protein

:y protein

:ide binding;ATP binding;aminoacyl-tRNA ligases

ılbumin superfamily protein

›tein

·otein

ılbumin superfamily protein

family protein

tor AUX/IAA-related

rotamase
er family protein

lbumin superfamily protein

otein

erase

lbumin superfamily protein
family protein

:ein family

l

›tein

·otein

·otein

ılbumin superfamily protein

ʳ protein

ı (DUF21)

;-like domain

; protein-related

:ive / PSI-N, putative (PSAN)

›tein

.y protein

ılbumin superfamily protein

rotein

ı

.y protein

.n-containing protein

erase

protein

tein

otein

lbumin superfamily protein

·otein

.y protein

l

r protein

rotein

otein

r protein

otein

.y protein

.y protein
' protein

›tein

.ly protein

.y protein

ılbumin superfamily protein

.y protein

, protein

rotein

protein

ed protein family
