# Supplementary material for: Transcription factor PagLBD21 functions as a repressor of secondary xylem development in Populus
Source: For Res (Fayettev). 2022 Dec 21;2:19. doi: 10.48130/FR-2022-0019 (PMC11524276; doi:10.48130/FR-2022-0019)
Supplement: Supplementary file 1 — Supplementary data to this article can be found online. [file FR-2022-0019-S1.zip › 10.48130_FR-2022-0019-Suppl-TableS3.pdf]

Supplemental Table S3. Gene Ontology (GO) analysis of the up- or down -regulated DEGs ir

GOBPID	Pvalue	OddsRatio	ExpCount	Count	Size	Term	Genes	up- or do
GO:004549	2.88E-09	14.2311	0.978847	11	52	xylan bio	Potri.001	down-regu
GO:001008	0.006395	3.842842	1.65651	6	88	xylem dev	Potri.001	down-regu
GO:000969	4.77E-21	6.53233	8.263727	46	439	phenylpro	Potri.001	down-regu
GO:001041	1.92E-09	6.68903	3.049485	18	162	hemicellu	Potri.001	down-regu
GO:000980	2.08E-06	4.777818	3.425964	15	182	lignin bi	Potri.001	down-regu
GO:000980	2.22E-14	7.109888	4.555403	28	242	lignin me	Potri.001	down-regu
GO:004548	0.004324	2.661065	4.310691	11	229	pectin me	Potri.001	down-regu
GO:000983	3.36E-21	11.10476	3.576556	32	190	plant-typ	Potri.001	down-regu
GO:000973	0.009596	1.762147	13.45915	23	715	response	Potri.001	down-regu
GO:003024	0.000796	4.389605	1.957694	8	104	cellulose	Potri.004	down-regu
GO:003015	0.005195	1.621045	23.64292	37	1256	cell diff	Potri.001	down-regu
GO:004254	7.20E-21	6.296806	8.734327	47	464	cell wall	Potri.001	down-regu
GO:004403	3.86E-08	6.715528	2.522413	15	134	cell wall	Potri.001	down-regu
GO:000983	8.04E-19	6.861416	6.644865	39	353	plant-typ	Potri.001	down-regu
GO:004455	4.57E-15	5.449486	7.717832	37	410	secondary	Potri.001	down-regu
GO:007059	2.84E-08	6.88988	2.465941	15	131	cell wall	Potri.001	down-regu
GO:001605	1.36E-05	2.668126	10.72967	27	570	carbohydr	Potri.001	down-regu
GO:200065	1.42E-07	10.80366	1.110615	10	59	regulatio	Potri.001	down-regu
GO:007155	1.10E-08	2.978544	14.55094	40	773	cell wall	Potri.001	down-regu
GO:001038	3.91E-10	5.528526	4.649523	23	247	cell wall	Potri.001	down-regu
GO:000969	3.88E-16	6.337526	6.362505	35	338	phenylpro	Potri.001	down-regu
GO:001974	1.66E-19	4.534952	15.2286	60	809	secondary	Potri.001	down-regu
GO:004627	1.45E-10	13.83497	1.185911	13	63	lignin ca	Potri.005	down-regu
GO:004627	1.45E-10	13.83497	1.185911	13	63	phenylpro	Potri.005	down-regu
GO:007166	3.02E-15	4.97295	9.336694	41	496	plant-typ	Potri.001	down-regu
GO:190333	8.53E-07	8.674958	1.336503	10	71	regulatio	Potri.001	down-regu
GO:200028	0.003324	4.439803	1.449446	6	77	regulatio	Potri.002	down-regu
GO:004549	9.36E-09	9.340648	1.637686	13	87	xylan met	Potri.001	down-regu
GO:000701	0.009989	3.090909	2.371821	7	126	actin fil	Potri.004	down-regu
GO:000333	4.92E-06	5.579858	2.371821	12	126	amino aci	Potri.001	down-regu
GO:000686	2.71E-05	4.639608	2.804773	12	149	amino aci	Potri.001	down-regu
GO:005127	0.001615	3.900344	2.183582	8	116	beta-gluc	Potri.004	down-regu
GO:005127	0.002123	3.387226	2.804773	9	149	beta-gluc	Potri.004	down-regu
GO:000597	6.01E-05	1.842949	31.02192	54	1648	carbohydr	Potri.001	down-regu
GO:190503	2.90E-05	4.605837	2.823597	12	150	carboxyli	Potri.001	down-regu
GO:004403	3.01E-08	4.306043	5.835434	23	310	cell wall	Potri.001	down-regu
GO:007155	1.02E-15	3.485209	21.08286	65	1120	cell wall	Potri.001	down-regu
GO:003463	1.3E-06	3.538257	6.682513	22	355	cellular	Potri.001	down-regu
GO:004426	2.92E-07	2.81009	13.36503	35	710	cellular	Potri.001	down-regu
GO:007058	3.86E-08	6.715528	2.522413	15	134	cellular	Potri.001	down-regu
GO:004886	0.001905	1.67186	26.16533	42	1390	cellular	Potri.001	down-regu
GO:000607	0.000418	2.693892	6.626041	17	352	cellular	Potri.003	down-regu
GO:003369	1.19E-07	4.295141	5.327186	21	283	cellular	Potri.001	down-regu
GO:004426	7.69E-08	3.389275	9.242574	29	491	cellular	Potri.001	down-regu
GO:003024	0.001188	3.706231	2.578885	9	137	cellulose	Potri.004	down-regu
GO:004522	2.34E-09	2.975226	16.0945	44	855	external	Potri.001	down-regu
GO:001039	0.004612	2.636701	4.348339	11	231	galacturo	Potri.001	down-regu
GO:004404	0.000432	2.685786	6.644865	17	353	glucan me	Potri.003	down-regu
GO:001976	0.00592	2.687984	3.87774	10	206	glucosino	Potri.001	down-regu
GO:001975	0.00592	2.687984	3.87774	10	206	glycosino	Potri.001	down-regu
GO:004274	0.008966	2.881608	2.898893	8	154	hydrogen	Potri.002	down-regu

GO:001569	0.007575	2.754163	3.40714	9	181	inorganic	Potri.001	down-regu
GO:001087	0.006282	2.406884	5.176594	12	275	lipid loc	Potri.001	down-regu
GO:000686	0.002352	2.743181	4.574227	12	243	lipid tra	Potri.001	down-regu
GO:007233	0.00982	1.949491	8.997862	17	478	monocarbo	Potri.001	down-regu
GO:000685	0.00222	3.694339	2.296526	8	122	oligopept	Potri.001	down-regu
GO:190382	2.90E-05	4.605837	2.823597	12	150	organic a	Potri.001	down-regu
GO:001584	8.15E-05	3.417055	4.668347	15	248	organic a	Potri.001	down-regu
GO:004548	0.004273	4.202466	1.524742	6	81	pectin bi	Potri.001	down-regu
GO:000027	6.99E-07	3.683572	6.437801	22	342	polysacch	Potri.001	down-regu
GO:000597	1.37E-06	2.656449	13.66621	34	726	polysacch	Potri.001	down-regu
GO:000989	0.009338	1.652194	18.10867	29	962	positive	Potri.001	down-regu
GO:003132	0.009734	1.662109	17.37453	28	923	positive	Potri.001	down-regu
GO:190350	0.008654	1.734513	14.87094	25	790	positive	Potri.001	down-regu
GO:190268	0.008654	1.734513	14.87094	25	790	positive	Potri.001	down-regu
GO:004589	0.008029	1.746216	14.77682	25	785	positive	Potri.001	down-regu
GO:004408	0.000736	3.003126	4.555403	13	242	regulatio	Potri.001	down-regu
GO:000973	0.001625	1.749169	22.02406	37	1170	response	Potri.001	down-regu
GO:000110	6.26E-06	2.463116	14.66388	34	779	response	Potri.001	down-regu
GO:009730	0.002136	1.719216	22.38171	37	1189	response	Potri.001	down-regu
GO:001003	0.000502	1.677813	33.80787	54	1796	response	Potri.001	down-regu
GO:003399	3.50E-05	1.832575	34.20317	59	1817	response	Potri.001	down-regu
GO:000697	1.31E-06	2.207488	24.24529	50	1288	response	Potri.001	down-regu
GO:000965	5.60E-06	2.17654	22.00523	45	1169	response	Potri.001	down-regu
GO:000941	6.67E-06	2.492562	14.06151	33	747	response	Potri.001	down-regu
GO:000941	4.50E-06	2.543598	13.79798	33	733	response	Potri.001	down-regu
GO:000961	1.39E-06	2.978198	10.05201	28	534	response	Potri.001	down-regu
GO:001614	0.00592	2.687984	3.87774	10	206	S-glycosi	Potri.001	down-regu
GO:000962	0.00965	2.842482	2.936541	8	156	systemic	Potri.001	down-regu
GO:005508	0.001112	1.682043	28.5748	46	1518	transmemb	Potri.001	down-regu
GO:001034	9.97E-13	27.73883	0.658839	12	35	suberin b	Potri.001	down-regu
GO:001943	5.44E-11	1.956399	84.04907	143	4465	aromatic	Potri.001	down-regu
GO:190136	8.39E-10	1.859684	89.03742	145	4730	organic c	Potri.001	down-regu
GO:000635	8.33E-09	1.935775	61.46029	107	3265	regulatio	Potri.001	down-regu
GO:190350	8.74E-09	1.933728	61.51677	107	3268	regulatio	Potri.001	down-regu
GO:200114	8.74E-09	1.933728	61.51677	107	3268	regulatio	Potri.001	down-regu
GO:000635	4.54E-08	1.854923	65.0745	109	3457	transcrip	Potri.001	down-regu
GO:009765	4.74E-08	1.853059	65.13097	109	3460	nucleic a	Potri.001	down-regu
GO:003277	5.67E-08	1.845638	65.35686	109	3472	RNA biosy	Potri.001	down-regu
GO:005125	7.59E-08	1.842946	64.1333	107	3407	regulatio	Potri.001	down-regu
GO:200011	1.07E-07	1.810238	67.78515	111	3601	regulatio	Potri.001	down-regu
GO:001055	1.31E-07	1.802079	68.04869	111	3615	regulatio	Potri.001	down-regu
GO:001921	2.89E-07	1.78636	65.88393	107	3500	regulatio	Potri.001	down-regu
GO:003132	3.18E-07	1.753796	71.70054	114	3809	regulatio	Potri.001	down-regu
GO:000988	3.90E-07	1.741764	72.81115	115	3868	regulatio	Potri.001	down-regu
GO:004424	4.19E-07	1.565562	146.6012	200	7788	cellular	Potri.001	down-regu
GO:000905	8.48E-07	1.537702	155.2978	208	8250	biosynthe	Potri.001	down-regu
GO:190157	1.35E-06	1.528048	152.606	204	8107	organic s	Potri.001	down-regu
GO:008009	6.17E-06	1.614609	81.30077	120	4319	regulatio	Potri.001	down-regu
GO:190170	8.03E-06	1.688926	60.85793	95	3233	response	Potri.001	down-regu
GO:003465	8.08E-06	1.636253	72.47232	109	3850	nucleobas	Potri.001	down-regu
GO:003132	1.13E-05	1.579879	85.78088	124	4557	regulatio	Potri.001	down-regu
GO:005117	1.29E-05	1.598098	78.32658	115	4161	regulatio	Potri.001	down-regu
GO:001055	2.14E-05	12.62795	0.583543	6	31	response	Potri.001	down-regu
GO:001046	2.20E-05	1.589559	75.01356	110	3985	regulatio	Potri.001	down-regu

GO:001813	3.13E-05	1.56067	79.90779	115	4245	heterocyc	Potri.001	down-regu
GO:000905	3.29E-05	1.502297	103.5319	142	5500	macromole	Potri.001	down-regu
GO:001041	3.68E-05	16.41934	0.395304	5	21	glucurono	Potri.001	down-regu
GO:001041	3.68E-05	16.41934	0.395304	5	21	glucurono	Potri.001	down-regu
GO:003464	5.53E-05	1.487589	101.9695	139	5417	cellular	Potri.001	down-regu
GO:000972	6.65E-05	1.616778	57.58255	87	3059	response	Potri.001	down-regu
GO:006025	7.18E-05	1.515636	84.95262	119	4513	regulatio	Potri.001	down-regu
GO:000971	0.000105	1.592424	58.35434	87	3100	response	Potri.001	down-regu
GO:001003	0.000167	1.51697	72.77351	103	3866	response	Potri.001	down-regu
GO:001922	0.000229	1.450751	94.43991	127	5017	regulatio	Potri.001	down-regu
GO:004222	0.000264	1.43208	101.8942	135	5413	response	Potri.001	down-regu
GO:000672	0.000405	1.375253	134.8362	170	7163	cellular	Potri.001	down-regu
GO:190133	0.000512	26.18995	0.169416	3	9	negative	Potri.002	down-regu
GO:190235	0.000512	26.18995	0.169416	3	9	sulfate t	Potri.001	down-regu
GO:001011	0.000577	12.3413	0.395304	4	21	photoprot	Potri.002	down-regu
GO:190136	0.000846	1.345402	139.6928	173	7421	organic c	Potri.001	down-regu
GO:000980	0.000978	19.64118	0.207064	3	11	cinnamic	Potri.008	down-regu
GO:000980	0.000978	19.64118	0.207064	3	11	cinnamic	Potri.008	down-regu
GO:000827	0.001546	9.120047	0.508247	4	27	sulfate t	Potri.001	down-regu
GO:003526	0.001649	15.71193	0.244712	3	13	multicell	Potri.002	down-regu
GO:004408	0.001807	1.539913	40.77274	60	2166	cellular	Potri.001	down-regu
GO:000995	0.001857	6.249695	0.884727	5	47	positive	Potri.003	down-regu
GO:001614	0.002031	8.389898	0.545895	4	29	S-glycosi	Potri.001	down-regu
GO:001975	0.002031	8.389898	0.545895	4	29	glycosino	Potri.001	down-regu
GO:001976	0.002031	8.389898	0.545895	4	29	glucosino	Potri.001	down-regu
GO:190134	0.002069	14.2831	0.263536	3	14	positive	Potri.005	down-regu
GO:190334	0.002069	14.2831	0.263536	3	14	positive	Potri.005	down-regu
GO:004647	0.00207	52.29762	0.075296	2	4	glyceroph	Potri.001	down-regu
GO:200002	0.002551	13.09242	0.28236	3	15	regulatio	Potri.002	down-regu
GO:000990	0.003298	7.231729	0.621191	4	33	anther de	Potri.001	down-regu
GO:001040	0.003407	34.86395	0.09412	2	5	rhamnogal	Potri.006	down-regu
GO:001960	0.003407	34.86395	0.09412	2	5	butyrate	Potri.006	down-regu
GO:000962	0.003473	1.372154	76.03005	99	4039	response	Potri.001	down-regu
GO:011012	0.004386	10.47291	0.338832	3	18	phloem lo	Potri.007	down-regu
GO:199093	0.005046	26.14711	0.112944	2	6	xylan ace	Potri.008	down-regu
GO:004624	0.005137	9.818037	0.357656	3	19	salicylic	Potri.008	down-regu
GO:000655	0.00596	9.240204	0.37648	3	20	L-phenyla	Potri.008	down-regu
GO:190134	0.00596	9.240204	0.37648	3	20	negative	Potri.005	down-regu
GO:190222	0.00596	9.240204	0.37648	3	20	erythrose	Potri.008	down-regu
GO:190333	0.00596	9.240204	0.37648	3	20	negative	Potri.005	down-regu
GO:001576	0.006857	8.726576	0.395304	3	21	disacchar	Potri.013	down-regu
GO:001577	0.006857	8.726576	0.395304	3	21	sucrose t	Potri.013	down-regu
GO:001577	0.006857	8.726576	0.395304	3	21	oligosacc	Potri.013	down-regu
GO:004265	0.006857	8.726576	0.395304	3	21	regulatio	Potri.001	down-regu
GO:003064	0.006977	20.91701	0.131768	2	7	cellular	Potri.001	down-regu
GO:004645	0.006977	20.91701	0.131768	2	7	short-cha	Potri.006	down-regu
GO:007250	0.006977	20.91701	0.131768	2	7	cellular	Potri.001	down-regu
GO:000981	0.00783	8.267013	0.414128	3	22	drought r	Potri.008	down-regu
GO:000656	0.00888	7.853407	0.432952	3	23	proline m	Potri.001	down-regu
GO:200006	0.00888	7.853407	0.432952	3	23	regulatio	Potri.002	down-regu
GO:000173	0.009187	17.43027	0.150592	2	8	establish	Potri.010	down-regu
GO:000173	0.009187	17.43027	0.150592	2	8	morphogen	Potri.010	down-regu
GO:000200	0.009187	17.43027	0.150592	2	8	morphogen	Potri.010	down-regu
GO:000991	0.009187	17.43027	0.150592	2	8	phloem su	Potri.013	down-regu

GO:003000	0.009187	17.43027	0.150592	2	8	cellular	Potri.001	down-regu
GO:004620	0.009187	17.43027	0.150592	2	8	nitric ox	Potri.002	down-regu
GO:004872	0.009187	17.43027	0.150592	2	8	tissue mo	Potri.010	down-regu
GO:009050	0.009187	17.43027	0.150592	2	8	axillary	Potri.002	down-regu
GO:007234	0.009304	5.241126	0.828255	4	44	sulfur co	Potri.001	down-regu
GO:000982	0.000132	6.985646	1.132757	7	56	plant-typ	Potri.004	up-regula
GO:000982	0.000649	4.550561	1.901413	8	94	plant-typ	Potri.004	up-regula
GO:003024	0.000113	4.285625	2.771209	11	137	cellulose	Potri.001	up-regula
GO:000973	2.03E-08	3.369008	10.29595	32	509	response	Potri.001	up-regula
GO:001605	2.39E-11	4.183529	9.264333	35	458	carbohydr	Potri.001	up-regula
GO:000600	8.73E-10	10.32711	1.638452	14	81	glucose m	Potri.002	up-regula
GO:003024	0.00426	6.717022	0.667517	4	33	cellulose	Potri.001	up-regula
GO:000983	0.003051	5.539999	0.991162	5	49	plant-typ	Potri.001	up-regula
GO:001699	1.17E-06	7.309553	1.719363	11	85	cell wall	Potri.001	up-regula
GO:000966	0.00142	2.928253	4.308522	12	213	plant-typ	Potri.004	up-regula
GO:003024	0.005259	3.523307	2.103691	7	104	cellulose	Potri.001	up-regula
GO:004403	0.00022	2.860212	6.270619	17	310	cell wall	Potri.001	up-regula
GO:007155	5.83E-07	2.600128	15.63609	38	773	cell wall	Potri.001	up-regula
GO:001038	0.004754	2.501799	4.996267	12	247	cell wall	Potri.001	up-regula
GO:007166	0.000563	2.292994	10.03299	22	496	plant-typ	Potri.001	up-regula
GO:004254	0.005064	2.480524	5.036723	12	249	cell wall	Potri.001	up-regula
GO:007155	1.17E-08	2.534211	22.65514	53	1120	cell wall	Potri.001	up-regula
GO:001605	2.67E-07	2.980993	11.52985	32	570	carbohydr	Potri.001	up-regula
GO:004427	0.003799	3.370387	2.508247	8	124	cellular	Potri.001	up-regula
GO:004426	6.15E-05	2.273683	14.36174	31	710	cellular	Potri.001	up-regula
GO:000609	1.25E-05	5.572173	2.184603	11	108	glycolyti	Potri.002	up-regula
GO:004636	0.000105	5.313427	1.860958	9	92	monosacch	Potri.004	up-regula
GO:000609	7.3E-08	5.907356	3.03417	16	150	pyruvate	Potri.002	up-regula
GO:001599	6.42E-08	7.818841	1.921641	13	95	chlorophy	Potri.001	up-regula
GO:001599	8.87E-08	6.282207	2.690298	15	133	chlorophy	Potri.001	up-regula
GO:000965	4.54E-05	2.94449	7.201098	20	356	chloropla	Potri.001	up-regula
GO:000985	0.000604	4.604247	1.881186	8	93	photoresp	Potri.001	up-regula
GO:001597	2.25E-45	10.42589	9.547523	76	472	photosynt	Potri.001	up-regula
GO:000976	3.4E-19	18.99713	1.618224	22	80	photosynt	Potri.001	up-regula
GO:001968	3.8E-29	10.47278	5.663785	47	280	photosynt	Potri.001	up-regula
GO:000976	2.39E-12	8.979077	2.649842	20	131	photosynt	Potri.001	up-regula
GO:001020	0.000117	7.131413	1.112529	7	55	photosyst	Potri.001	up-regula
GO:004614	0.000378	2.940548	5.380595	15	266	pigment b	Potri.001	up-regula
GO:004244	0.001949	2.472948	6.331302	15	313	pigment m	Potri.001	up-regula
GO:000677	2.98E-07	6.746029	2.184603	13	108	porphyrin	Potri.001	up-regula
GO:000677	5.14E-07	5.407592	3.074626	15	152	porphyrin	Potri.001	up-regula
GO:001010	4.38E-05	6.995208	1.294579	8	64	regulatio	Potri.002	up-regula
GO:000963	0.00813	2.937952	2.85212	8	141	response	Potri.004	up-regula
GO:001021	0.000649	4.550561	1.901413	8	94	response	Potri.002	up-regula
GO:000964	8.78E-07	5.569589	2.791437	14	138	response	Potri.001	up-regula
GO:000964	1.58E-08	4.306781	6.108796	24	302	response	Potri.001	up-regula
GO:001011	8.17E-05	4.905288	2.225058	10	110	response	Potri.002	up-regula
GO:000941	0.001749	2.714647	5.016495	13	248	response	Potri.001	up-regula
GO:003301	5.07E-07	6.407681	2.285742	13	113	tetrapyrr	Potri.001	up-regula
GO:003301	6.08E-07	5.329436	3.115082	15	154	tetrapyrr	Potri.001	up-regula
GO:001002	0.001938	4.274382	1.759819	7	87	thylakoid	Potri.002	up-regula
GO:004603	1.92E-05	5.298162	2.285742	11	113	ADP metab	Potri.002	up-regula
GO:190160	4.56E-11	5.428961	5.380595	26	266	alpha-ami	Potri.001	up-regula
GO:190160	4.37E-09	3.90906	8.111349	29	401	alpha-ami	Potri.001	up-regula

GO:004360	0.000115	1.898505	24.94088	45	1233	amide bio	Potri.001	up-regula
GO:000675	1.25E-05	5.572173	2.184603	11	108	ATP gener	Potri.002	up-regula
GO:004603	0.001212	2.836228	4.814217	13	238	ATP metab	Potri.002	up-regula
GO:005127	0.009402	3.134191	2.346425	7	116	beta-gluc	Potri.001	up-regula
GO:005127	0.000237	3.911427	3.013943	11	149	beta-gluc	Potri.001	up-regula
GO:190113	0.006901	1.844663	12.31873	22	609	carbohydr	Potri.001	up-regula
GO:190113	1.61E-06	2.206717	23.70698	49	1172	carbohydr	Potri.001	up-regula
GO:004639	3.05E-07	2.535129	17.76001	42	878	carboxyli	Potri.001	up-regula
GO:004545	0.000756	3.165958	4.005105	12	198	cell redo	Potri.001	up-regula
GO:004360	0.000212	1.783037	29.47191	50	1457	cellular	Potri.001	up-regula
GO:000865	5.45E-11	5.16914	5.845835	27	289	cellular	Potri.001	up-regula
GO:000906	0.00688	3.029447	2.771209	8	137	cellular	Potri.003	up-regula
GO:000652	4.38E-11	3.665415	12.01531	40	594	cellular	Potri.001	up-regula
GO:000607	0.000118	2.818155	7.120186	19	352	cellular	Potri.001	up-regula
GO:004309	0.000521	3.828751	2.791437	10	138	cellular	Potri.001	up-regula
GO:004424	0.001582	4.441351	1.699135	7	84	cellular	Potri.001	up-regula
GO:004426	9.87E-06	2.781353	9.931851	26	491	cellular	Potri.001	up-regula
GO:003459	0.001502	3.5808	2.67007	9	132	cellular	Potri.001	up-regula
GO:004233	0.000408	5.702897	1.355263	7	67	cuticle d	Potri.006	up-regula
GO:000653	6.81E-06	9.331203	1.01139	8	50	cysteine	Potri.003	up-regula
GO:000645	0.001341	4.033069	2.123919	8	105	'de novo'	Potri.001	up-regula
GO:004364	6.58E-05	4.21254	3.074626	12	152	dicarboxy	Potri.001	up-regula
GO:002290	5.08E-09	5.004775	4.8749	22	241	electron	Potri.001	up-regula
GO:004522	2.56E-06	2.401193	17.29477	39	855	external	Potri.001	up-regula
GO:000663	0.001815	2.217289	8.920461	19	441	fatty aci	Potri.001	up-regula
GO:001939	0.007596	3.704346	1.719363	6	85	fatty aci	Potri.001	up-regula
GO:004204	0.009533	4.129503	1.294579	5	64	fluid tra	Potri.004	up-regula
GO:000609	8.28E-27	5.699134	14.19992	68	702	generatio	Potri.001	up-regula
GO:000925	0.002461	2.881881	4.005105	11	198	glucan bi	Potri.001	up-regula
GO:004404	0.000122	2.809625	7.140414	19	353	glucan me	Potri.001	up-regula
GO:000908	0.001214	5.526259	1.19344	6	59	glutamine	Potri.001	up-regula
GO:000906	0.009402	3.134191	2.346425	7	116	glutamine	Potri.001	up-regula
GO:190165	0.001279	4.621859	1.638452	7	81	glycosyl	Potri.001	up-regula
GO:001931	3.72E-07	4.863286	3.843282	17	190	hexose me	Potri.002	up-regula
GO:001604	0.000982	2.237926	9.790256	21	484	lipid cat	Potri.001	up-regula
GO:003444	0.001911	3.797388	2.245286	8	111	lipid oxi	Potri.001	up-regula
GO:003278	0.000278	1.960239	19.23664	36	951	monocarbo	Potri.001	up-regula
GO:000599	3.76E-06	3.855372	5.036723	18	249	monosacch	Potri.002	up-regula
GO:000982	0.007459	3.28541	2.245286	7	111	multidime	Potri.005	up-regula
GO:000674	0.003636	5.298783	1.031618	5	51	NADPH reg	Potri.002	up-regula
GO:005508	0.000568	2.162852	12.076	25	597	nucleobas	Potri.001	up-regula
GO:000913	3.49E-07	6.06013	2.589159	14	128	nucleosid	Potri.001	up-regula
GO:000616	6.22E-07	6.28163	2.326197	13	115	nucleosid	Potri.001	up-regula
GO:000675	0.000711	2.354761	8.880005	20	439	nucleosid	Potri.001	up-regula
GO:000911	0.0006	2.389444	8.758638	20	433	nucleotid	Potri.001	up-regula
GO:004693	1.23E-06	5.876878	2.467792	13	122	nucleotid	Potri.001	up-regula
GO:000922	0.003957	5.185874	1.051846	5	52	nucleotid	Potri.004	up-regula
GO:001605	2.03E-06	2.341766	19.11527	42	945	organic a	Potri.001	up-regula
GO:001963	0.000186	2.050085	17.39591	34	860	organopho	Potri.001	up-regula
GO:004304	2.69E-05	2.04791	22.71582	44	1123	peptide b	Potri.001	up-regula
GO:000651	4.91E-05	1.945313	25.50726	47	1261	peptide m	Potri.001	up-regula
GO:001819	2.89E-08	8.43777	1.800274	13	89	peptidyl-	Potri.001	up-regula
GO:000966	0.002505	4.070308	1.84073	7	91	plastid m	Potri.002	up-regula
GO:000965	3.13E-07	3.230695	9.325017	28	461	plastid o	Potri.001	up-regula

GO:000027	1.56E-06	3.770823	5.724468	20	283	polysacchPotri.001	up-regula
GO:000597	1.23E-07	2.782108	14.68538	38	726	polysacchPotri.001	up-regula
GO:005126	0.005872	4.686468	1.152985	5	57	protein hPotri.003	up-regula
GO:004202	6.17E-05	5.728582	1.739591	9	86	protein rPotri.001	up-regula
GO:001829	1.70E-08	7.947868	2.043008	14	101	protein-cPotri.001	up-regula
GO:006500	0.005711	1.719175	17.43637	29	862	protein-cPotri.001	up-regula
GO:000913	1.92E-05	5.298162	2.285742	11	113	purine nuPotri.002	up-regula
GO:000616	0.000817	2.615489	6.412213	16	317	purine nuPotri.001	up-regula
GO:000917	1.92E-05	5.298162	2.285742	11	113	purine riPotri.002	up-regula
GO:000915	0.000436	2.783492	6.048113	16	299	purine riPotri.001	up-regula
GO:007252	0.002669	2.312507	7.201098	16	356	purine-coPotri.001	up-regula
GO:000622	0.005042	4.874245	1.112529	5	55	pyrimidinPotri.001	up-regula
GO:000622	0.005042	4.874245	1.112529	5	55	pyrimidinPotri.001	up-regula
GO:007252	0.001279	4.621859	1.638452	7	81	pyrimidinPotri.001	up-regula
GO:007252	0.003797	3.756349	1.982325	7	98	pyrimidinPotri.001	up-regula
GO:004346	6.81E-06	9.331203	1.01139	8	50	regulatioPotri.002	up-regula
GO:000961	0.004738	1.642104	22.69559	36	1122	response Potri.001	up-regula
GO:004668	2.41E-10	3.383249	13.26944	41	656	response Potri.001	up-regula
GO:000940	7.05E-12	3.457792	15.00903	47	742	response Potri.001	up-regula
GO:001003	1.13E-09	2.929494	17.47682	47	864	response Potri.001	up-regula
GO:000926	1.89E-12	3.018969	22.39218	61	1107	response Potri.001	up-regula
GO:000918	2.45E-05	5.146281	2.346425	11	116	ribonuclePotri.002	up-regula
GO:000925	0.00018	2.810466	6.756086	18	334	ribonuclePotri.001	up-regula
GO:001969	0.000286	2.698259	7.019047	18	347	ribose phPotri.001	up-regula
GO:000907	2.00E-09	10.87502	1.456402	13	72	serine faPotri.001	up-regula
GO:000906	5.13E-10	9.640182	1.860958	15	92	serine faPotri.001	up-regula
GO:004428	4.57E-10	2.602073	25.64885	61	1268	small molPotri.001	up-regula
GO:000009	3.47E-06	7.329219	1.557541	10	77	sulfur amPotri.003	up-regula
GO:000009	7.56E-05	4.954999	2.20483	10	109	sulfur amPotri.003	up-regula
GO:004427	3.09E-07	4.423253	4.69285	19	232	sulfur coPotri.001	up-regula
GO:004427	0.005446	4.778516	1.132757	5	56	sulfur coPotri.003	up-regula
GO:000679	7.44E-05	2.318628	13.1683	29	651	sulfur coPotri.001	up-regula
GO:000641	2.32E-05	2.06177	22.57423	44	1116	translatiPotri.001	up-regula
GO:000683	0.009533	4.129503	1.294579	5	64	water traPotri.004	up-regula
GO:000976	4.29E-17	39.14208	0.687745	15	34	photosyntPotri.001	up-regula
GO:000815	2.23E-14	1.91506	363.4734	455	17969	metabolicPotri.001	up-regula
GO:190156	3.57E-14	2.437288	50.44814	108	2494	organonitPotri.001	up-regula
GO:004343	4.78E-14	2.65562	37.78553	89	1868	oxoacid mPotri.001	up-regula
GO:000597	5.43E-14	2.764504	33.33542	82	1648	carbohydrPotri.001	up-regula
GO:004428	1.73E-13	2.294412	58.1347	117	2874	small molPotri.001	up-regula
GO:001975	4.14E-13	2.683729	33.33542	80	1648	carboxyliPotri.001	up-regula
GO:000608	6.68E-13	2.519878	39.60604	89	1958	organic aPotri.001	up-regula
GO:001701	3.40E-12	24.6664	0.728201	12	36	protein nPotri.001	up-regula
GO:001811	3.40E-12	24.6664	0.728201	12	36	peptidyl-Potri.001	up-regula
GO:000962	4.51E-12	2.023115	81.70009	144	4039	response Potri.001	up-regula
GO:001925	1.41E-11	21.13987	0.809112	12	40	reductivePotri.002	up-regula
GO:001968	1.96E-11	20.41025	0.82934	12	41	photosyntPotri.002	up-regula
GO:001597	8.79E-11	17.4059	0.930479	12	46	carbon fiPotri.002	up-regula
GO:000941	1.57E-10	2.499012	30.80694	70	1523	response Potri.001	up-regula
GO:000931	3.63E-10	2.445948	31.41378	70	1553	response Potri.001	up-regula
GO:000010	1.31E-08	38.08258	0.323645	7	16	sulfate aPotri.001	up-regula
GO:190157	3.06E-08	1.958442	52.00568	93	2571	organic sPotri.001	up-regula
GO:000976	4.24E-08	20.64234	0.546151	8	27	photosyntPotri.001	up-regula
GO:007170	8.12E-08	1.542624	338.8966	404	16754	organic sPotri.001	up-regula

GO:000977	1.06E-07	17.82573	0.606834	8	30	photosyntPotri.001	up-regula
GO:001003	1.26E-07	2.084114	36.32913	70	1796	response Potri.001	up-regula
GO:000905	6.56E-07	1.522336	166.8794	222	8250	biosynthePotri.001	up-regula
GO:000905	1.72E-06	1.753064	59.32814	96	2933	catabolicPotri.001	up-regula
GO:003254	2.04E-06	20.94745	0.404556	6	20	plastid tPotri.001	up-regula
GO:000609	2.08E-06	11.2	0.869795	8	43	gluconeogPotri.004	up-regula
GO:001934	2.08E-06	11.2	0.869795	8	43	cysteine Potri.003	up-regula
GO:001941	2.41E-06	97.48254	0.121367	4	6	sulfate rPotri.004	up-regula
GO:003009	4.49E-06	12.68675	0.687745	7	34	protein rPotri.002	up-regula
GO:001931	5.82E-06	9.559105	0.991162	8	49	hexose biPotri.004	up-regula
GO:000964	6.60E-06	16.29034	0.485467	6	24	response Potri.001	up-regula
GO:001020	8.54E-06	15.43245	0.505695	6	25	photosystPotri.002	up-regula
GO:011010	1.09E-05	48.7381	0.161822	4	8	ribulose Potri.004	up-regula
GO:190157	1.10E-05	1.451686	163.9868	212	8107	organic sPotri.001	up-regula
GO:004423	1.16E-05	1.413968	324.9596	378	16065	cellular Potri.001	up-regula
GO:190156	3.45E-05	1.415171	170.5204	216	8430	organonitPotri.001	up-regula
GO:004222	7.54E-05	1.458397	109.4931	147	5413	response Potri.001	up-regula
GO:000610	7.77E-05	9.770382	0.728201	6	36	malate mePotri.001	up-regula
GO:000694	0.000103	12.83993	0.485467	5	24	syncytiumPotri.004	up-regula
GO:004254	0.000124	8.881297	0.788884	6	39	regulatioPotri.002	up-regula
GO:004434	0.000124	8.881297	0.788884	6	39	cell wallPotri.001	up-regula
GO:190125	0.000186	11.08795	0.546151	5	27	chloroplaPotri.004	up-regula
GO:000909	0.000249	16.2418	0.323645	4	16	homoserinPotri.003	up-regula
GO:007012	0.000271	36.49564	0.141595	3	7	mitochondPotri.001	up-regula
GO:001043	0.000428	29.19556	0.161822	3	8	regulatioPotri.001	up-regula
GO:000678	0.000505	12.99217	0.384328	4	19	protoporpPotri.002	up-regula
GO:004650	0.000505	12.99217	0.384328	4	19	protoporpPotri.002	up-regula
GO:000657	0.000571	8.409627	0.687745	5	34	valine mePotri.009	up-regula
GO:001020	0.000621	12.17976	0.404556	4	20	photoinhiPotri.002	up-regula
GO:004315	0.000621	12.17976	0.404556	4	20	negative Potri.002	up-regula
GO:007184	0.000724	1.393298	99.66238	130	4927	cellular Potri.001	up-regula
GO:000983	0.000963	7.389314	0.768656	5	38	plant-typPotri.006	up-regula
GO:001967	0.001204	18.24544	0.222506	3	11	GDP-mannoPotri.005	up-regula
GO:001043	0.001209	97.17722	0.060683	2	3	cellular Potri.002	up-regula
GO:190342	0.001209	97.17722	0.060683	2	3	positive Potri.002	up-regula
GO:190515	0.001277	9.74254	0.485467	4	24	negative Potri.002	up-regula
GO:004254	0.001368	6.772876	0.82934	5	41	cell wallPotri.006	up-regula
GO:000654	0.001581	16.21764	0.242734	3	12	glutaminePotri.004	up-regula
GO:004423	0.001636	1.270963	311.8723	349	15418	primary mPotri.001	up-regula
GO:000654	0.00174	8.856277	0.525923	4	26	glycine mPotri.001	up-regula
GO:001946	0.002025	14.5954	0.262961	3	13	glycine dPotri.003	up-regula
GO:004856	0.002308	8.117725	0.566378	4	28	photosystPotri.002	up-regula
GO:007148	0.002308	8.117725	0.566378	4	28	cellular Potri.002	up-regula
GO:004235	0.002386	48.58703	0.080911	2	4	'de novo'Potri.005	up-regula
GO:000597	0.002539	13.26812	0.283189	3	14	glycogen Potri.005	up-regula
GO:004635	0.002539	13.26812	0.283189	3	14	mannan caPotri.005	up-regula
GO:001604	0.002761	1.357322	87.86957	113	4344	cellular Potri.001	up-regula
GO:005089	0.002929	1.257458	230.2126	264	11381	response Potri.001	up-regula
GO:001614	0.003051	5.539999	0.991162	5	49	S-glycosiPotri.001	up-regula
GO:001975	0.003051	5.539999	0.991162	5	49	glycosinoPotri.001	up-regula
GO:001976	0.003051	5.539999	0.991162	5	49	glucosinoPotri.001	up-regula
GO:004424	0.003102	1.281712	157.5341	188	7788	cellular Potri.001	up-regula
GO:000654	0.003126	12.16204	0.303417	3	15	glycine cPotri.003	up-regula
GO:001041	0.00379	11.22614	0.323645	3	16	mannan mePotri.005	up-regula

GO:000208	0.003923	32.3903	0.101139	2	5	regulatio	Potri.008	up-regula
GO:190358	0.003923	32.3903	0.101139	2	5	positive	Potri.008	up-regula
GO:190386	0.003923	32.3903	0.101139	2	5	positive	Potri.008	up-regula
GO:000678	0.00426	6.717022	0.667517	4	33	heme bios	Potri.002	up-regula
GO:005127	0.00426	6.717022	0.667517	4	33	beta-gluc	Potri.001	up-regula
GO:000662	0.004522	1.483709	39.92968	57	1974	lipid met	Potri.001	up-regula
GO:000624	0.004534	10.42393	0.343873	3	17	CTP biosy	Potri.001	up-regula
GO:000920	0.004534	10.42393	0.343873	3	17	pyrimidin	Potri.001	up-regula
GO:000920	0.004534	10.42393	0.343873	3	17	pyrimidin	Potri.001	up-regula
GO:003122	0.004534	10.42393	0.343873	3	17	arabinan	Potri.001	up-regula
GO:003122	0.004534	10.42393	0.343873	3	17	arabinan	Potri.001	up-regula
GO:004603	0.004534	10.42393	0.343873	3	17	CTP metab	Potri.001	up-regula
GO:000037	0.004752	6.49291	0.687745	4	34	Group II	Potri.001	up-regula
GO:000921	0.004752	6.49291	0.687745	4	34	pyrimidin	Potri.001	up-regula
GO:000922	0.004752	6.49291	0.687745	4	34	pyrimidin	Potri.001	up-regula
GO:000914	0.005359	9.728685	0.3641	3	18	pyrimidin	Potri.001	up-regula
GO:000998	0.005396	1.265215	443.7575	473	21938	cellular	Potri.001	up-regula
GO:004408	0.005725	1.446605	43.81342	61	2166	cellular	Potri.001	up-regula
GO:000909	0.005806	24.29193	0.121367	2	6	homoserin	Potri.013	up-regula
GO:004235	0.005806	24.29193	0.121367	2	6	GDP-L-fuc	Potri.005	up-regula
GO:004636	0.005806	24.29193	0.121367	2	6	GDP-L-fuc	Potri.005	up-regula
GO:004216	0.005848	6.086706	0.728201	4	36	heme meta	Potri.002	up-regula
GO:000909	0.00627	9.120345	0.384328	3	19	valine bi	Potri.009	up-regula
GO:000963	0.00627	9.120345	0.384328	3	19	response	Potri.001	up-regula
GO:001019	0.00627	9.120345	0.384328	3	19	nonphotoc	Potri.001	up-regula
GO:199006	0.00627	9.120345	0.384328	3	19	energy qu	Potri.001	up-regula
GO:001026	0.007266	8.583574	0.404556	3	20	somatic e	Potri.019	up-regula
GO:000622	0.00802	19.43291	0.141595	2	7	UTP biosy	Potri.001	up-regula
GO:001026	0.00802	19.43291	0.141595	2	7	response	Potri.001	up-regula
GO:004605	0.00802	19.43291	0.141595	2	7	UTP metab	Potri.001	up-regula
GO:007081	0.00802	19.43291	0.141595	2	7	hydrogen	Potri.008	up-regula
GO:003109	0.008352	8.106445	0.424784	3	21	regenerat	Potri.019	up-regula
GO:000656	0.009289	5.26332	0.82934	4	41	L-serine	Potri.005	up-regula

L36
own-regulated
lated
lated
lated
lated
lated
lated
lated
lated
lated
lated
lated
lated
lated
lated
lated
lated
lated
lated
lated
lated
lated
lated
lated
lated
lated
lated
lated
lated
lated
lated
lated
lated
lated
lated
lated
lated
lated
lated
lated
lated
lated
lated
lated
lated
lated
lated
lated
lated
lated
lated
lated

ılated
ılated
ılated
ılated
ılated
ılated
ılated
ılated
ılated
ılated
ılated
ılated
ılated
ılated
ılated
ılated
ılated
ılated
ılated
ılated
ılated
ılated
ılated
ılated
ılated
ılated
ılated
ılated
ılated
ılated
ılated
ılated
ılated
ılated
ılated
ılated
ılated
ılated
ılated
ılated
ılated
ılated
ılated
ılated
ılated
ılated
ılated
ılated
ılated
ılated
ılated
ılated
ılated
ılated

ılated
ılated
ılated
ılated
ılated
ılated
ılated
ılated
ılated
ılated
ılated
ılated
ılated
ılated
ılated
ılated
ılated
ılated
ılated
ılated
ılated
ılated
ılated
ılated
ılated
ılated
ılated
ılated
ılated
ılated
ılated
ılated
ılated
ılated
ılated
ılated
ılated
ılated
ılated
ılated
ılated
ılated
ılated
ılated
ılated
ılated
ılated
ılated
ılated
ılated
ılated
ılated
ılated
ılated

lated
lated
lated
lated
lated
ated
ated
ated
ated
ated
ated
ated
ated
ated
ated
ated
ated
ated
ated
ated
ated
ated
ated
ated
ated
ated
ated
ated
ated
ated
ated
ated
ated
ated
ated
ated
ated
ated
ated
ated
ated
ated
ated
ated
ated
ated
ated
ated
ated
ated
ated
ated
ated
ated

ated
ated
ated
ated
ated
ated
ated
ated
ated
ated
ated
ated
ated
ated
ated
ated
ated
ated
ated
ated
ated
ated
ated
ated
ated
ated
ated
ated
ated
ated
ated
ated
ated
ated
ated
ated
ated
ated
ated
ated
ated
ated
ated
ated
ated
ated
ated
ated
ated
ated
ated
ated
ated
ated

ated
ated
ated
ated
ated
ated
ated
ated
ated
ated
ated
ated
ated
ated
ated
ated
ated
ated
ated
ated
ated
ated
ated
ated
ated
ated
ated
ated
ated
ated
ated
ated
ated
ated
ated
ated
ated
ated
ated
ated
ated
ated
ated
ated
ated
ated
ated
ated
ated
ated
ated
ated
ated
ated

ated
ated
ated
ated
ated
ated
ated
ated
ated
ated
ated
ated
ated
ated
ated
ated
ated
ated
ated
ated
ated
ated
ated
ated
ated
ated
ated
ated
ated
ated
ated
ated
ated
ated
ated
ated
ated
ated
ated
ated
ated
ated
ated
ated
ated
ated
ated
ated
ated
ated
ated
ated
ated
ated

ated
ated
ated
ated
ated
ated
ated
ated
ated
ated
ated
ated
ated
ated
ated
ated
ated
ated
ated
ated
ated
ated
ated
ated
ated
ated
ated
ated
ated
ated
ated
ated
ated
