# Supplementary material for: Transcription factor PagLBD21 functions as a repressor of secondary xylem development in Populus
Source: For Res (Fayettev). 2022 Dec 21;2:19. doi: 10.48130/FR-2022-0019 (PMC11524276; doi:10.48130/FR-2022-0019)
Supplement: Supplementary file 1 — Supplementary data to this article can be found online. [file FR-2022-0019-S1.zip › 10.48130_FR-2022-0019-Suppl-TableS4.pdf]

Supplemental Table S4. Summary of PagLBD21 DAP-seq binding sites and target genes

genes	seqnames	start	end	width	strand	score	blockCoun	thick.sta
Potri.001	Chr01	929145	929398	254	*	215	139	5
Potri.001	Chr01	1854598	1854819	222	*	178	121	2
Potri.001	Chr01	2425062	2425323	262	*	198	127	4
Potri.001	Chr01	2561159	2561423	265	*	195	131	4
Potri.001	Chr01	3620015	3620238	224	*	269	126	4
Potri.001	Chr01	3625622	3625833	212	*	211	102	3
Potri.001	Chr01	3657896	3658137	242	*	176	152	4
Potri.001	Chr01	3867742	3868246	505	*	182	112	3
Potri.001	Chr01	4424567	4425087	521	*	446	124	3
Potri.001	Chr01	4467168	4467691	524	*	172	127	4
Potri.001	Chr01	4727100	4727366	267	*	175	133	4
Potri.001	Chr01	4738452	4738686	235	*	202	127	4
Potri.001	Chr01	4817439	4817822	384	*	178	195	3
Potri.001	Chr01	5083634	5083924	291	*	572	155	4
Potri.001	Chr01	5164245	5164447	203	*	177	88	4
Potri.001	Chr01	5518387	5518682	296	*	212	125	5
Potri.001	Chr01	5532327	5532761	435	*	190	119	4
Potri.001	Chr01	5594344	5594619	276	*	196	132	3
Potri.001	Chr01	5701321	5701540	220	*	174	119	3
Potri.001	Chr01	5713056	5713314	259	*	172	121	4
Potri.001	Chr01	6538430	6538652	223	*	249	101	3
Potri.001	Chr01	6611503	6611833	331	*	188	173	4
Potri.001	Chr01	6820209	6820675	467	*	235	343	4
Potri.001	Chr01	7118166	7118428	263	*	243	122	5
Potri.001	Chr01	7162426	7162900	475	*	197	326	3
Potri.001	Chr01	7165158	7165391	234	*	199	135	4
Potri.001	Chr01	7202933	7203201	269	*	192	159	4
Potri.001	Chr01	8135630	8135930	301	*	200	137	3
Potri.001	Chr01	8148327	8148541	215	*	201	120	3
Potri.001	Chr01	8565216	8565441	226	*	168	104	5
Potri.001	Chr01	9211692	9211926	235	*	192	84	4
Potri.001	Chr01	9221095	9221365	271	*	177	120	4
Potri.001	Chr01	9499008	9499299	292	*	316	128	5
Potri.001	Chr01	9531364	9531614	251	*	214	116	4
Potri.001	Chr01	9856180	9856473	294	*	174	107	3
Potri.001	Chr01	9965471	9965676	206	*	224	95	3
Potri.001	Chr01	10709156	10709372	217	*	175	140	5
Potri.001	Chr01	11014348	11014630	283	*	187	108	3
Potri.001	Chr01	11304236	11304483	248	*	236	113	4
Potri.001	Chr01	11491617	11491855	239	*	363	95	2
Potri.001	Chr01	11563680	11564504	825	*	204	407	2
Potri.001	Chr01	12180669	12180990	322	*	180	182	3
Potri.001	Chr01	12720296	12720501	206	*	181	110	4
Potri.001	Chr01	13282377	13282837	461	*	280	168	4
Potri.001	Chr01	13557019	13557261	243	*	205	124	4
Potri.001	Chr01	13980531	13980772	242	*	197	115	3
Potri.001	Chr01	14228354	14228582	229	*	223	107	5
Potri.001	Chr01	14800502	14800934	433	*	217	123	3
Potri.001	Chr01	14994392	14994681	290	*	200	139	3
Potri.001	Chr01	15234734	15234982	249	*	346	128	5
Potri.001	Chr01	15548930	15549153	224	*	169	127	5

Potri.001Chr01	15637732	15637979	248	*	178	144	4
Potri.001Chr01	15654351	15654591	241	*	209	102	5
Potri.001Chr01	16011573	16012004	432	*	318	121	3
Potri.001Chr01	16056375	16056587	213	*	168	127	3
Potri.001Chr01	16181800	16182105	306	*	191	118	3
Potri.001Chr01	16391659	16391885	227	*	180	90	3
Potri.001Chr01	17037292	17037893	602	*	169	456	3
Potri.001Chr01	17235612	17235829	218	*	187	77	2
Potri.001Chr01	17649304	17649878	575	*	419	424	2
Potri.001Chr01	17695577	17695861	285	*	181	134	4
Potri.001Chr01	18472258	18472463	206	*	197	93	3
Potri.001Chr01	18787754	18787961	208	*	273	105	3
Potri.001Chr01	18838615	18838831	217	*	213	129	5
Potri.001Chr01	19166562	19166793	232	*	169	83	5
Potri.001Chr01	19207721	19208080	360	*	203	197	4
Potri.001Chr01	19232438	19232643	206	*	379	89	3
Potri.001Chr01	19476930	19477297	368	*	200	177	3
Potri.001Chr01	19852116	19852329	214	*	293	109	3
Potri.001Chr01	20657701	20657911	211	*	248	104	3
Potri.001Chr01	20747746	20748008	263	*	259	112	4
Potri.001Chr01	20863611	20863873	263	*	230	118	5
Potri.001Chr01	20932991	20933295	305	*	251	158	5
Potri.001Chr01	20949693	20950171	479	*	340	125	4
Potri.001Chr01	21094922	21095165	244	*	224	115	3
Potri.001Chr01	21207549	21207852	304	*	237	141	4
Potri.001Chr01	21285467	21285673	207	*	273	130	3
Potri.001Chr01	21735628	21735845	218	*	264	100	5
Potri.001Chr01	21767641	21767950	310	*	201	187	3
Potri.001Chr01	21800187	21800454	268	*	285	112	4
Potri.001Chr01	22029190	22029426	237	*	207	152	3
Potri.001Chr01	22367111	22367349	239	*	235	117	4
Potri.001Chr01	22654563	22655071	509	*	183	172	3
Potri.001Chr01	22842446	22842664	219	*	230	143	4
Potri.001Chr01	23053466	23054213	748	*	168	374	3
Potri.001Chr01	24554688	24555000	313	*	244	154	5
Potri.001Chr01	25022733	25023209	477	*	351	335	4
Potri.001Chr01	25215327	25215574	248	*	214	111	4
Potri.001Chr01	25376647	25376892	246	*	295	116	5
Potri.001Chr01	25410497	25410992	496	*	222	378	4
Potri.001Chr01	26022918	26023184	267	*	248	137	5
Potri.001Chr01	26612929	26613158	230	*	530	122	3
Potri.001Chr01	26784001	26784229	229	*	174	134	3
Potri.001Chr01	26837013	26837229	217	*	201	120	3
Potri.001Chr01	27287131	27287411	281	*	228	142	4
Potri.001Chr01	27822169	27822501	333	*	349	159	5
Potri.001Chr01	28014634	28014868	235	*	208	115	4
Potri.001Chr01	28202924	28203134	211	*	445	76	3
Potri.001Chr01	28406347	28406597	251	*	208	112	3
Potri.001Chr01	28526866	28527090	225	*	178	115	3
Potri.001Chr01	28647293	28647509	217	*	199	134	4
Potri.001Chr01	28658989	28659248	260	*	193	166	4
Potri.001Chr01	28984782	28985001	220	*	175	109	5
Potri.001Chr01	29390817	29391164	348	*	294	153	2
Potri.001Chr01	29742882	29743098	217	*	228	87	5

Potri.001Chr01	31448791	31449151	361	*	203	144	3
Potri.001Chr01	32048733	32049072	340	*	176	198	3
Potri.001Chr01	33457592	33457805	214	*	342	98	3
Potri.001Chr01	33570581	33570838	258	*	181	128	3
Potri.001Chr01	33828568	33828801	234	*	215	118	4
Potri.001Chr01	33915954	33916220	267	*	212	136	3
Potri.001Chr01	33976105	33976353	249	*	171	84	3
Potri.001Chr01	34176846	34177099	254	*	225	117	4
Potri.001Chr01	34316537	34316817	281	*	208	161	4
Potri.001Chr01	35232642	35233062	421	*	192	257	4
Potri.001Chr01	35578650	35579245	596	*	170	341	3
Potri.001Chr01	35923384	35923805	422	*	205	306	4
Potri.001Chr01	36109221	36110150	930	*	193	813	3
Potri.001Chr01	36668052	36668510	459	*	186	189	3
Potri.001Chr01	36719265	36719591	327	*	187	177	3
Potri.001Chr01	36773988	36774280	293	*	179	119	3
Potri.001Chr01	37073017	37073239	223	*	181	114	4
Potri.001Chr01	37170785	37170991	207	*	214	95	3
Potri.001Chr01	37970166	37970480	315	*	296	158	5
Potri.001Chr01	38195395	38195649	255	*	223	107	5
Potri.001Chr01	38546782	38547340	559	*	204	457	4
Potri.001Chr01	38581020	38581547	528	*	172	296	3
Potri.001Chr01	38900271	38900501	231	*	306	164	6
Potri.001Chr01	39441266	39441514	249	*	172	88	4
Potri.001Chr01	39926911	39927281	371	*	169	187	3
Potri.001Chr01	40086079	40086306	228	*	177	150	3
Potri.001Chr01	40123073	40123282	210	*	322	75	3
Potri.001Chr01	40943679	40943908	230	*	175	118	5
Potri.001Chr01	41015949	41016244	296	*	527	145	3
Potri.001Chr01	41352508	41352709	202	*	175	107	4
Potri.001Chr01	41633636	41633870	235	*	415	116	3
Potri.001Chr01	41805493	41805830	338	*	232	172	5
Potri.001Chr01	42021359	42021841	483	*	216	362	4
Potri.001Chr01	42083558	42083824	267	*	215	136	3
Potri.001Chr01	42380485	42380711	227	*	269	133	4
Potri.001Chr01	42901618	42901889	272	*	191	142	5
Potri.001Chr01	43036833	43037117	285	*	277	129	4
Potri.001Chr01	43533743	43534216	474	*	183	182	4
Potri.001Chr01	43693426	43693663	238	*	181	134	4
Potri.001Chr01	43726680	43726996	317	*	361	182	2
Potri.001Chr01	43778833	43779060	228	*	208	102	3
Potri.001Chr01	43863650	43863872	223	*	192	121	4
Potri.001Chr01	43908021	43908260	240	*	182	128	4
Potri.001Chr01	44052037	44052357	321	*	344	189	4
Potri.001Chr01	44500054	44500285	232	*	205	126	4
Potri.001Chr01	45286666	45287290	625	*	545	159	3
Potri.001Chr01	45643749	45644024	276	*	168	147	3
Potri.001Chr01	45718959	45719178	220	*	462	114	3
Potri.001Chr01	45804776	45804979	204	*	243	88	3
Potri.001Chr01	46964321	46964525	205	*	242	75	3
Potri.001Chr01	47055743	47055954	212	*	169	99	5
Potri.001Chr01	47358494	47358780	287	*	179	177	3
Potri.001Chr01	47427294	47427647	354	*	299	173	4
Potri.001Chr01	47801016	47801297	282	*	251	137	5

Potri.001	Chr01	47877530	47878138	609	*	301	173	4
Potri.001	Chr01	48291364	48291605	242	*	200	159	4
Potri.001	Chr01	48748860	48749093	234	*	303	113	5
Potri.001	Chr01	48821696	48822085	390	*	239	100	3
Potri.001	Chr01	49084154	49084651	498	*	174	320	3
Potri.001	Chr01	49269866	49270070	205	*	175	102	4
Potri.001	Chr01	49440659	49440895	237	*	173	106	4
Potri.001	Chr01	49657251	49657503	253	*	249	132	3
Potri.001	Chr01	50300894	50301682	789	*	194	156	3
Potri.001	Chr01	50310104	50310381	278	*	188	94	3
Potri.001	Chr01	50407731	50408069	339	*	180	160	3
Potri.002	Chr02	76798	77162	365	*	203	188	3
Potri.002	Chr02	242397	242811	415	*	199	247	3
Potri.002	Chr02	673630	673887	258	*	274	134	5
Potri.002	Chr02	1264017	1264278	262	*	259	113	3
Potri.002	Chr02	1762232	1762521	290	*	215	142	3
Potri.002	Chr02	1824675	1824915	241	*	199	107	4
Potri.002	Chr02	1828043	1828250	208	*	343	111	3
Potri.002	Chr02	1948095	1948422	328	*	184	141	4
Potri.002	Chr02	2328005	2328302	298	*	188	125	3
Potri.002	Chr02	2373052	2373324	273	*	329	139	4
Potri.002	Chr02	2564367	2564632	266	*	174	135	3
Potri.002	Chr02	2560178	2560385	208	*	338	122	3
Potri.002	Chr02	2761988	2762205	218	*	188	106	3
Potri.002	Chr02	2804876	2805217	342	*	674	180	5
Potri.002	Chr02	3149934	3150430	497	*	193	379	4
Potri.002	Chr02	3191462	3191679	218	*	280	99	4
Potri.002	Chr02	3259870	3260141	272	*	269	131	4
Potri.002	Chr02	3484213	3484690	478	*	412	166	4
Potri.002	Chr02	3653603	3653899	297	*	190	168	3
Potri.002	Chr02	3668732	3670070	1339	*	189	963	3
Potri.002	Chr02	3751194	3751509	316	*	387	154	2
Potri.002	Chr02	3782545	3782813	269	*	208	117	4
Potri.002	Chr02	3848902	3849499	598	*	286	176	3
Potri.002	Chr02	4204158	4204448	291	*	169	145	3
Potri.002	Chr02	4726104	4726322	219	*	181	106	3
Potri.002	Chr02	4817633	4817928	296	*	175	151	4
Potri.002	Chr02	4997837	4998038	202	*	216	108	4
Potri.002	Chr02	5057873	5058122	250	*	222	132	4
Potri.002	Chr02	5256221	5256645	425	*	179	112	2
Potri.002	Chr02	5524404	5524641	238	*	461	116	3
Potri.002	Chr02	5549415	5549665	251	*	309	131	4
Potri.002	Chr02	5801365	5801595	231	*	193	97	4
Potri.002	Chr02	5973654	5974021	368	*	252	168	4
Potri.002	Chr02	6027926	6028310	385	*	178	200	3
Potri.002	Chr02	6140066	6140285	220	*	358	104	3
Potri.002	Chr02	6295734	6295952	219	*	175	112	5
Potri.002	Chr02	6542230	6542512	283	*	207	142	3
Potri.002	Chr02	6760361	6760631	271	*	171	133	3
Potri.002	Chr02	6805103	6805326	224	*	170	113	4
Potri.002	Chr02	6814253	6814484	232	*	249	126	4
Potri.002	Chr02	7002606	7002901	296	*	260	133	4
Potri.002	Chr02	7145316	7145525	210	*	191	107	4
Potri.002	Chr02	7727244	7727460	217	*	177	134	4

Potri.002	Chr02	7822629	7823108	480	*	179	242	3
Potri.002	Chr02	7838964	7839252	289	*	195	133	4
Potri.002	Chr02	7975444	7975718	275	*	219	126	4
Potri.002	Chr02	8024870	8025102	233	*	264	100	4
Potri.002	Chr02	8210564	8210874	311	*	168	145	3
Potri.002	Chr02	8322259	8322462	204	*	203	88	3
Potri.002	Chr02	9176993	9177632	640	*	168	483	3
Potri.002	Chr02	9532546	9532760	215	*	172	101	4
Potri.002	Chr02	9968017	9968303	287	*	175	140	4
Potri.002	Chr02	10237921	10238198	278	*	194	162	2
Potri.002	Chr02	10425607	10425861	255	*	168	135	3
Potri.002	Chr02	10485049	10485377	329	*	197	188	4
Potri.002	Chr02	10813182	10813451	270	*	228	146	5
Potri.002	Chr02	11632464	11632671	208	*	182	99	4
Potri.002	Chr02	11789392	11789598	207	*	173	105	4
Potri.002	Chr02	11909070	11909319	250	*	182	117	4
Potri.002	Chr02	11994767	11994992	226	*	168	76	3
Potri.002	Chr02	12458308	12458509	202	*	224	92	3
Potri.002	Chr02	12533595	12533870	276	*	175	98	3
Potri.002	Chr02	12796938	12797171	234	*	181	123	4
Potri.002	Chr02	13302694	13303015	322	*	183	166	4
Potri.002	Chr02	13622351	13622645	295	*	214	152	4
Potri.002	Chr02	14003251	14003537	287	*	261	151	5
Potri.002	Chr02	14263568	14263812	245	*	461	117	3
Potri.002	Chr02	15618534	15618824	291	*	721	140	3
Potri.002	Chr02	15739053	15739258	206	*	175	130	4
Potri.002	Chr02	15786299	15786595	297	*	312	133	5
Potri.002	Chr02	16766241	16766604	364	*	197	264	3
Potri.002	Chr02	16836837	16837061	225	*	223	160	4
Potri.002	Chr02	17125547	17125757	211	*	278	107	3
Potri.002	Chr02	17352392	17352621	230	*	196	137	5
Potri.002	Chr02	17652906	17653166	261	*	733	130	3
Potri.002	Chr02	17813769	17813982	214	*	323	108	3
Potri.002	Chr02	17888468	17888703	236	*	175	95	3
Potri.002	Chr02	18209164	18209386	223	*	237	118	4
Potri.002	Chr02	18302835	18303053	219	*	300	126	3
Potri.002	Chr02	18356805	18357546	742	*	263	239	2
Potri.002	Chr02	18363354	18364058	705	*	222	538	2
Potri.002	Chr02	18368445	18368681	237	*	267	97	2
Potri.002	Chr02	18378206	18379838	1633	*	251	666	2
Potri.002	Chr02	18385289	18386283	995	*	204	592	2
Potri.002	Chr02	18386656	18388425	1770	*	276	187	2
Potri.002	Chr02	18388713	18389284	572	*	184	235	2
Potri.002	Chr02	19442406	19442650	245	*	296	125	5
Potri.002	Chr02	19550541	19550809	269	*	178	145	4
Potri.002	Chr02	19759869	19760084	216	*	181	128	4
Potri.002	Chr02	20470878	20471281	404	*	181	100	3
Potri.002	Chr02	20569255	20569478	224	*	324	116	4
Potri.002	Chr02	20800009	20800252	244	*	189	114	4
Potri.002	Chr02	20919927	20920165	239	*	192	99	4
Potri.002	Chr02	21172016	21172253	238	*	304	128	5
Potri.002	Chr02	21522065	21522542	478	*	186	145	3
Potri.002	Chr02	21546356	21546637	282	*	188	139	5
Potri.002	Chr02	21819525	21819731	207	*	176	97	4

Potri.002Chr02	21925698	21925951	254	*	277	103	5
Potri.002Chr02	22414081	22414542	462	*	213	295	3
Potri.002Chr02	22422640	22423310	671	*	242	206	4
Potri.002Chr02	22594997	22595231	235	*	175	113	4
Potri.002Chr02	22718099	22718300	202	*	169	81	3
Potri.002Chr02	22768066	22768274	209	*	259	131	3
Potri.002Chr02	22874743	22874965	223	*	193	139	5
Potri.002Chr02	23764535	23764747	213	*	169	112	5
Potri.002Chr02	23906127	23906472	346	*	271	148	4
Potri.002Chr02	24058868	24059106	239	*	531	115	3
Potri.002Chr02	24339924	24340204	281	*	192	111	4
Potri.002Chr02	24736897	24737178	282	*	287	164	4
Potri.002Chr02	24751796	24752101	306	*	190	141	3
Potri.002Chr02	24957979	24958222	244	*	248	87	5
Potri.002Chr02	25090748	25090983	236	*	203	108	3
Potri.002Chr02	25138468	25138735	268	*	185	145	4
Potri.003Chr03	61971	62230	260	*	266	150	6
Potri.003Chr03	129656	129946	291	*	170	138	4
Potri.003Chr03	656114	656315	202	*	231	93	3
Potri.003Chr03	1495994	1496290	297	*	193	113	2
Potri.003Chr03	1513843	1514045	203	*	172	90	3
Potri.003Chr03	2551852	2552448	597	*	213	150	3
Potri.003Chr03	2712833	2713079	247	*	265	116	4
Potri.003Chr03	2903853	2904371	519	*	421	349	3
Potri.003Chr03	3018055	3018510	456	*	220	312	2
Potri.003Chr03	3022246	3022458	213	*	208	125	3
Potri.003Chr03	3376699	3377001	303	*	269	158	4
Potri.003Chr03	3491753	3492039	287	*	331	136	3
Potri.003Chr03	3506542	3506778	237	*	210	122	3
Potri.003Chr03	3650135	3650348	214	*	177	104	4
Potri.003Chr03	3826644	3826887	244	*	602	120	3
Potri.003Chr03	4444781	4445019	239	*	169	95	5
Potri.003Chr03	4461650	4461897	248	*	240	141	5
Potri.003Chr03	4755672	4755880	209	*	240	121	4
Potri.003Chr03	6161995	6162202	208	*	174	94	3
Potri.003Chr03	6267778	6268062	285	*	233	151	5
Potri.003Chr03	6423038	6423278	241	*	192	112	4
Potri.003Chr03	6766119	6766331	213	*	204	69	4
Potri.003Chr03	7196440	7196683	244	*	188	110	3
Potri.003Chr03	8381002	8381251	250	*	199	104	4
Potri.003Chr03	9386042	9386341	300	*	842	168	3
Potri.003Chr03	9389169	9389542	374	*	418	205	2
Potri.003Chr03	9566687	9567187	501	*	253	136	3
Potri.003Chr03	9569214	9569530	317	*	555	149	3
Potri.003Chr03	10182079	10182321	243	*	510	137	3
Potri.003Chr03	10359058	10359370	313	*	170	149	3
Potri.003Chr03	10535089	10535357	269	*	168	121	3
Potri.003Chr03	10615766	10616209	444	*	218	292	3
Potri.003Chr03	11126434	11126675	242	*	199	126	4
Potri.003Chr03	11573532	11573804	273	*	191	139	3
Potri.003Chr03	11619030	11619296	267	*	212	108	3
Potri.003Chr03	12027669	12028001	333	*	168	173	3
Potri.003Chr03	12303519	12303759	241	*	188	100	4
Potri.003Chr03	12427676	12427942	267	*	192	156	4

Potri.003Chr03	12735666	12735888	223	*	215	109	3
Potri.003Chr03	12742584	12742801	218	*	193	112	3
Potri.003Chr03	13043932	13044184	253	*	175	99	3
Potri.003Chr03	13055116	13055438	323	*	237	136	4
Potri.003Chr03	13104122	13104423	302	*	185	110	3
Potri.003Chr03	13158324	13158581	258	*	171	145	3
Potri.003Chr03	13183531	13183972	442	*	183	167	3
Potri.003Chr03	13697484	13697776	293	*	232	176	3
Potri.003Chr03	14060435	14060677	243	*	215	153	5
Potri.003Chr03	14190057	14190413	357	*	202	199	3
Potri.003Chr03	14267816	14268042	227	*	249	106	4
Potri.003Chr03	15105546	15105777	232	*	199	127	4
Potri.003Chr03	15333864	15334244	381	*	173	182	3
Potri.003Chr03	15380497	15381719	1223	*	228	1102	3
Potri.003Chr03	15553791	15554016	226	*	202	98	5
Potri.003Chr03	15715390	15715627	238	*	224	110	5
Potri.003Chr03	15837723	15837925	203	*	319	104	2
Potri.003Chr03	16382870	16383080	211	*	170	84	4
Potri.003Chr03	16625715	16625946	232	*	168	116	3
Potri.003Chr03	16699772	16700025	254	*	381	139	3
Potri.003Chr03	17015196	17015423	228	*	193	132	4
Potri.003Chr03	17271607	17271895	289	*	183	164	3
Potri.003Chr03	17595004	17595214	211	*	193	129	5
Potri.003Chr03	17760402	17760655	254	*	177	146	4
Potri.003Chr03	17956708	17956951	244	*	242	141	4
Potri.003Chr03	18363124	18363365	242	*	195	119	5
Potri.003Chr03	18396952	18397271	320	*	252	156	4
Potri.003Chr03	18476771	18476999	229	*	188	138	5
Potri.003Chr03	18772456	18772727	272	*	290	110	5
Potri.003Chr03	18816852	18817279	428	*	185	303	3
Potri.003Chr03	19295246	19295480	235	*	223	158	4
Potri.003Chr03	19296783	19297030	248	*	242	115	4
Potri.003Chr03	19533971	19534444	474	*	212	176	2
Potri.003Chr03	19568573	19568976	404	*	249	258	3
Potri.003Chr03	19598580	19598854	275	*	176	148	2
Potri.003Chr03	19927723	19927952	230	*	176	150	3
Potri.003Chr03	20146603	20146823	221	*	171	85	4
Potri.003Chr03	20179276	20179522	247	*	260	142	3
Potri.003Chr03	20194417	20194929	513	*	227	401	3
Potri.003Chr03	20297536	20297754	219	*	539	92	4
Potri.003Chr03	20350203	20350446	244	*	216	141	4
Potri.003Chr03	20399325	20399666	342	*	224	182	3
Potri.003Chr03	20447696	20448330	635	*	285	241	3
Potri.003Chr03	20542681	20543000	320	*	223	136	4
Potri.003Chr03	20595640	20595878	239	*	170	126	4
Potri.003Chr03	20657367	20657609	243	*	168	133	4
Potri.003Chr03	20738230	20738435	206	*	173	105	3
Potri.003Chr03	20783686	20784006	321	*	211	143	4
Potri.003Chr03	20931410	20931611	202	*	202	112	3
Potri.003Chr03	21312388	21312606	219	*	208	103	3
Potri.003Chr03	21383170	21383385	216	*	168	122	3
Potri.003Chr03	21407017	21407230	214	*	208	106	4
Potri.003Chr03	21512332	21512557	226	*	214	110	4
Potri.003Chr03	21527106	21527351	246	*	171	131	3

Potri.003	Chr03	21531997	21532286	290 *	199	111	3
Potri.004	Chr04	142186	142405	220 *	230	111	5
Potri.004	Chr04	529305	529516	212 *	192	111	4
Potri.004	Chr04	811231	811469	239 *	225	101	4
Potri.004	Chr04	1297190	1297593	404 *	210	174	2
Potri.004	Chr04	2270347	2270589	243 *	236	122	4
Potri.004	Chr04	2474541	2474752	212 *	195	120	5
Potri.004	Chr04	2589807	2590037	231 *	168	127	3
Potri.004	Chr04	2778885	2779370	486 *	183	332	3
Potri.004	Chr04	2803076	2803297	222 *	170	125	4
Potri.004	Chr04	2819772	2820118	347 *	172	185	3
Potri.004	Chr04	2912044	2912667	624 *	178	551	3
Potri.004	Chr04	3420787	3420993	207 *	229	104	5
Potri.004	Chr04	3801955	3802294	340 *	181	122	4
Potri.004	Chr04	3861446	3861764	319 *	183	134	3
Potri.004	Chr04	3869416	3869634	219 *	276	54	3
Potri.004	Chr04	3875049	3875521	473 *	217	176	3
Potri.004	Chr04	4190011	4190218	208 *	247	103	4
Potri.004	Chr04	4244015	4244260	246 *	264	115	4
Potri.004	Chr04	4328805	4329036	232 *	194	106	5
Potri.004	Chr04	4418400	4418620	221 *	197	98	5
Potri.004	Chr04	4537715	4537993	279 *	323	140	5
Potri.004	Chr04	4552978	4553189	212 *	215	77	4
Potri.004	Chr04	4750292	4750688	397 *	184	293	3
Potri.004	Chr04	5088525	5088781	257 *	176	137	3
Potri.004	Chr04	5192380	5192671	292 *	180	162	5
Potri.004	Chr04	5234097	5234310	214 *	188	94	5
Potri.004	Chr04	6106183	6106438	256 *	199	133	4
Potri.004	Chr04	6269750	6269989	240 *	286	120	5
Potri.004	Chr04	6276893	6277102	210 *	173	88	3
Potri.004	Chr04	6311719	6312076	358 *	225	180	3
Potri.004	Chr04	6778391	6778660	270 *	189	148	3
Potri.004	Chr04	6955127	6955418	292 *	193	129	4
Potri.004	Chr04	7558289	7558505	217 *	169	144	5
Potri.004	Chr04	7802923	7803174	252 *	204	124	3
Potri.004	Chr04	8208761	8209273	513 *	180	125	3
Potri.004	Chr04	8369143	8369385	243 *	187	132	4
Potri.004	Chr04	8427273	8427542	270 *	187	140	4
Potri.004	Chr04	8830799	8831089	291 *	188	150	3
Potri.004	Chr04	8906539	8906826	288 *	303	133	2
Potri.004	Chr04	9292473	9292734	262 *	168	147	5
Potri.004	Chr04	9322724	9323188	465 *	179	155	3
Potri.004	Chr04	9503758	9504004	247 *	330	100	2
Potri.004	Chr04	10287963	10288223	261 *	184	111	4
Potri.004	Chr04	10315252	10315464	213 *	371	91	3
Potri.004	Chr04	10375494	10375909	416 *	236	145	4
Potri.004	Chr04	10392733	10392951	219 *	232	122	3
Potri.004	Chr04	10447923	10448161	239 *	352	130	6
Potri.004	Chr04	11934926	11935188	263 *	178	109	4
Potri.004	Chr04	12145296	12145625	330 *	201	163	3
Potri.004	Chr04	12333541	12333764	224 *	204	83	4
Potri.004	Chr04	12472419	12472824	406 *	365	164	2
Potri.004	Chr04	12615952	12616166	215 *	203	110	4
Potri.004	Chr04	12681434	12681655	222 *	392	81	3

Potri.004	Chr04	13200702	13201094	393	*	656	234	3
Potri.004	Chr04	14309858	14310137	280	*	187	111	4
Potri.004	Chr04	14359023	14359340	318	*	375	145	2
Potri.004	Chr04	14458094	14458306	213	*	405	86	3
Potri.004	Chr04	14899532	14899829	298	*	308	141	5
Potri.004	Chr04	15267714	15267977	264	*	304	111	6
Potri.004	Chr04	16138759	16138967	209	*	346	80	3
Potri.004	Chr04	16447592	16447812	221	*	173	103	3
Potri.004	Chr04	16761882	16762123	242	*	246	144	4
Potri.004	Chr04	16997639	16997909	271	*	187	143	4
Potri.004	Chr04	17206449	17206872	424	*	202	329	4
Potri.004	Chr04	17750789	17751016	228	*	222	103	5
Potri.004	Chr04	18114535	18114799	265	*	214	110	4
Potri.004	Chr04	18158915	18159208	294	*	183	135	4
Potri.004	Chr04	18516299	18516521	223	*	193	99	4
Potri.004	Chr04	18539055	18539500	446	*	223	289	3
Potri.004	Chr04	18677754	18678269	516	*	282	87	3
Potri.004	Chr04	18790854	18791089	236	*	191	118	3
Potri.004	Chr04	19046660	19047077	418	*	268	142	3
Potri.004	Chr04	19238127	19238356	230	*	185	108	3
Potri.004	Chr04	19650423	19650642	220	*	264	138	3
Potri.004	Chr04	19910561	19910809	249	*	190	122	4
Potri.004	Chr04	20206515	20206852	338	*	308	192	3
Potri.004	Chr04	20356490	20356837	348	*	288	156	5
Potri.004	Chr04	20857561	20857830	270	*	195	143	3
Potri.004	Chr04	20887728	20888016	289	*	576	145	3
Potri.004	Chr04	21030881	21031163	283	*	205	118	4
Potri.004	Chr04	21399320	21399645	326	*	206	173	3
Potri.004	Chr04	21551332	21551541	210	*	343	105	3
Potri.004	Chr04	21615542	21615980	439	*	246	190	3
Potri.004	Chr04	22420702	22421090	389	*	207	139	3
Potri.004	Chr04	22822657	22822884	228	*	260	104	4
Potri.004	Chr04	22883573	22883870	298	*	355	146	4
Potri.004	Chr04	23314871	23315127	257	*	170	85	3
Potri.004	Chr04	23557384	23557683	300	*	177	172	3
Potri.004	Chr04	24099000	24099237	238	*	222	112	4
Potri.004	Chr04	24123136	24123368	233	*	188	140	5
Potri.004	Chr04	24170720	24170957	238	*	442	122	3
Potri.005	Chr05	3043	3260	218	*	209	120	4
Potri.005	Chr05	350362	350582	221	*	170	118	4
Potri.005	Chr05	759495	759718	224	*	187	94	4
Potri.005	Chr05	835678	835907	230	*	274	94	4
Potri.005	Chr05	1473722	1473982	261	*	203	143	3
Potri.005	Chr05	1855786	1856194	409	*	319	101	3
Potri.005	Chr05	1879402	1880096	695	*	367	545	3
Potri.005	Chr05	2229957	2230187	231	*	247	110	4
Potri.005	Chr05	3149585	3149832	248	*	203	104	3
Potri.005	Chr05	3193032	3193282	251	*	181	88	4
Potri.005	Chr05	3291262	3291464	203	*	176	111	3
Potri.005	Chr05	4019922	4020189	268	*	177	153	4
Potri.005	Chr05	4183983	4184419	437	*	218	88	2
Potri.005	Chr05	4185325	4185527	203	*	298	99	3
Potri.005	Chr05	4361239	4361465	227	*	193	108	4
Potri.005	Chr05	4535382	4535603	222	*	175	101	4

Potri.005Chr05	4542240	4542449	210 *	189	101	4
Potri.005Chr05	4712604	4713286	683 *	172	531	3
Potri.005Chr05	5278912	5279142	231 *	169	116	4
Potri.005Chr05	5413894	5414139	246 *	182	103	3
Potri.005Chr05	5598685	5598923	239 *	262	99	4
Potri.005Chr05	5688719	5688992	274 *	187	137	4
Potri.005Chr05	5844122	5844350	229 *	229	116	5
Potri.005Chr05	5992887	5993124	238 *	181	68	3
Potri.005Chr05	6084178	6084428	251 *	176	139	3
Potri.005Chr05	6186026	6186248	223 *	171	84	4
Potri.005Chr05	6352092	6352350	259 *	226	122	4
Potri.005Chr05	6476890	6477153	264 *	252	134	4
Potri.005Chr05	6900151	6900459	309 *	644	164	3
Potri.005Chr05	7165603	7165814	212 *	181	76	3
Potri.005Chr05	7378513	7378782	270 *	268	117	5
Potri.005Chr05	7436189	7436419	231 *	183	101	4
Potri.005Chr05	7693066	7693277	212 *	232	83	5
Potri.005Chr05	7890394	7890685	292 *	244	144	5
Potri.005Chr05	8363067	8363286	220 *	179	110	3
Potri.005Chr05	8404898	8405386	489 *	210	408	3
Potri.005Chr05	8427742	8427989	248 *	192	113	4
Potri.005Chr05	8845615	8846043	429 *	200	166	3
Potri.005Chr05	8914378	8914590	213 *	169	99	4
Potri.005Chr05	10895167	10895407	241 *	186	121	3
Potri.005Chr05	11232580	11232862	283 *	181	171	4
Potri.005Chr05	11266986	11267193	208 *	182	101	5
Potri.005Chr05	11533587	11533902	316 *	175	180	3
Potri.005Chr05	11607255	11607540	286 *	225	141	4
Potri.005Chr05	11709073	11709388	316 *	429	188	4
Potri.005Chr05	11810477	11810819	343 *	197	121	4
Potri.005Chr05	12000488	12000784	297 *	244	134	5
Potri.005Chr05	12279601	12279828	228 *	209	123	5
Potri.005Chr05	12344622	12345348	727 *	306	300	4
Potri.005Chr05	12743932	12744150	219 *	397	117	3
Potri.005Chr05	12752045	12752250	206 *	168	94	4
Potri.005Chr05	13239922	13240155	234 *	214	110	4
Potri.005Chr05	13425130	13425921	792 *	238	134	4
Potri.005Chr05	13493411	13493638	228 *	587	124	3
Potri.005Chr05	13504710	13504941	232 *	309	119	3
Potri.005Chr05	13547370	13547622	253 *	204	134	4
Potri.005Chr05	14524539	14524743	205 *	306	112	3
Potri.005Chr05	14814275	14814542	268 *	937	134	4
Potri.005Chr05	14825925	14826134	210 *	408	68	3
Potri.005Chr05	16038463	16038743	281 *	181	138	4
Potri.005Chr05	16086014	16086261	248 *	172	113	3
Potri.005Chr05	16263407	16263906	500 *	175	102	3
Potri.005Chr05	16498370	16498667	298 *	431	146	2
Potri.005Chr05	17008366	17008702	337 *	183	183	4
Potri.005Chr05	17718205	17718413	209 *	361	101	3
Potri.005Chr05	17897316	17897548	233 *	169	113	5
Potri.005Chr05	18577697	18578005	309 *	176	141	3
Potri.005Chr05	18610740	18611347	608 *	209	488	5
Potri.005Chr05	18664794	18665005	212 *	169	102	5
Potri.005Chr05	18956540	18956772	233 *	208	102	4

Potri.005	Chr05	19147111	19147415	305	*	404	147	5
Potri.005	Chr05	19184258	19184588	331	*	210	181	4
Potri.005	Chr05	19301372	19301675	304	*	181	148	3
Potri.005	Chr05	19331911	19332204	294	*	191	113	3
Potri.005	Chr05	19626375	19626622	248	*	327	81	2
Potri.005	Chr05	20544892	20545222	331	*	417	171	2
Potri.005	Chr05	20610020	20610280	261	*	220	119	4
Potri.005	Chr05	20755647	20755858	212	*	247	116	4
Potri.005	Chr05	20985012	20985309	298	*	180	192	3
Potri.005	Chr05	21358091	21358396	306	*	236	155	3
Potri.005	Chr05	21437309	21437533	225	*	171	99	3
Potri.005	Chr05	21462461	21462711	251	*	179	114	3
Potri.005	Chr05	21485275	21485933	659	*	223	434	3
Potri.005	Chr05	21883762	21883976	215	*	282	102	2
Potri.005	Chr05	21949144	21949412	269	*	223	141	5
Potri.005	Chr05	21982630	21982932	303	*	244	136	3
Potri.005	Chr05	22191846	22192104	259	*	171	129	3
Potri.005	Chr05	22394783	22395368	586	*	689	415	5
Potri.005	Chr05	22534380	22534634	255	*	168	109	3
Potri.005	Chr05	22580855	22581071	217	*	221	114	3
Potri.005	Chr05	22767997	22768331	335	*	168	176	5
Potri.005	Chr05	22985994	22986460	467	*	368	104	3
Potri.005	Chr05	23031405	23031721	317	*	198	158	3
Potri.005	Chr05	23576023	23576261	239	*	259	139	5
Potri.005	Chr05	23601924	23602134	211	*	175	116	4
Potri.005	Chr05	23664862	23665310	449	*	224	155	3
Potri.005	Chr05	24083689	24083920	232	*	197	79	4
Potri.005	Chr05	24205987	24206222	236	*	188	110	4
Potri.005	Chr05	24285228	24285611	384	*	174	231	3
Potri.005	Chr05	24429579	24429890	312	*	220	161	4
Potri.005	Chr05	24785100	24785339	240	*	180	112	5
Potri.005	Chr05	24877284	24877513	230	*	192	130	4
Potri.005	Chr05	24983874	24984111	238	*	183	120	4
Potri.005	Chr05	25230823	25231369	547	*	224	378	3
Potri.005	Chr05	25759918	25760137	220	*	182	93	4
Potri.006	Chr06	19546	19790	245	*	173	130	3
Potri.006	Chr06	73501	73723	223	*	169	131	5
Potri.006	Chr06	584267	584492	226	*	209	98	3
Potri.006	Chr06	674830	675082	253	*	214	130	4
Potri.006	Chr06	706350	706564	215	*	171	107	4
Potri.006	Chr06	998909	999130	222	*	172	130	4
Potri.006	Chr06	1211182	1211460	279	*	204	137	4
Potri.006	Chr06	1464065	1464367	303	*	174	176	3
Potri.006	Chr06	1628583	1628791	209	*	312	82	5
Potri.006	Chr06	1732087	1732327	241	*	238	116	4
Potri.006	Chr06	2704686	2704897	212	*	336	97	3
Potri.006	Chr06	3652600	3652801	202	*	178	111	3
Potri.006	Chr06	3656156	3656406	251	*	171	118	2
Potri.006	Chr06	3674160	3674588	429	*	173	289	3
Potri.006	Chr06	3858214	3858501	288	*	214	155	4
Potri.006	Chr06	4149558	4150011	454	*	176	135	3
Potri.006	Chr06	4496009	4496255	247	*	253	119	3
Potri.006	Chr06	4530595	4530921	327	*	185	179	3
Potri.006	Chr06	4611631	4611904	274	*	181	95	2

Potri.006	Chr06	4912751	4913009	259	*	233	160	5
Potri.006	Chr06	4993382	4993598	217	*	168	125	4
Potri.006	Chr06	5403030	5403262	233	*	196	139	4
Potri.006	Chr06	5456081	5456315	235	*	235	130	4
Potri.006	Chr06	5855120	5855419	300	*	267	165	3
Potri.006	Chr06	6097131	6097619	489	*	179	323	3
Potri.006	Chr06	6260133	6260376	244	*	216	103	4
Potri.006	Chr06	6509763	6509977	215	*	175	112	5
Potri.006	Chr06	6522769	6523039	271	*	259	121	4
Potri.006	Chr06	6538315	6538566	252	*	232	88	4
Potri.006	Chr06	6735062	6735338	277	*	168	152	3
Potri.006	Chr06	7189215	7189428	214	*	203	114	4
Potri.006	Chr06	7602058	7602351	294	*	183	142	3
Potri.006	Chr06	7753987	7754199	213	*	169	129	5
Potri.006	Chr06	8156421	8156650	230	*	171	106	3
Potri.006	Chr06	8218409	8218636	228	*	252	92	5
Potri.006	Chr06	8529653	8529974	322	*	192	156	3
Potri.006	Chr06	8705806	8706086	281	*	241	129	4
Potri.006	Chr06	9339130	9339388	259	*	193	149	3
Potri.006	Chr06	9443032	9443257	226	*	169	120	5
Potri.006	Chr06	9457540	9457780	241	*	186	104	4
Potri.006	Chr06	10440725	10440959	235	*	308	134	5
Potri.006	Chr06	10470671	10471079	409	*	203	195	3
Potri.006	Chr06	11142181	11142400	220	*	395	115	3
Potri.006	Chr06	11181180	11181449	270	*	181	116	3
Potri.006	Chr06	11383033	11383305	273	*	223	134	5
Potri.006	Chr06	11503235	11503464	230	*	175	90	5
Potri.006	Chr06	11739495	11739787	293	*	241	165	4
Potri.006	Chr06	12140513	12140752	240	*	288	153	6
Potri.006	Chr06	12772947	12773173	227	*	168	115	5
Potri.006	Chr06	12897772	12898010	239	*	170	96	4
Potri.006	Chr06	12957848	12958058	211	*	309	116	3
Potri.006	Chr06	13620623	13620861	239	*	208	107	5
Potri.006	Chr06	14057111	14057448	338	*	287	167	4
Potri.006	Chr06	15115850	15116090	241	*	195	104	5
Potri.006	Chr06	15214270	15214485	216	*	219	94	3
Potri.006	Chr06	15278442	15278658	217	*	513	138	5
Potri.006	Chr06	15988897	15989115	219	*	699	109	3
Potri.006	Chr06	16338470	16338869	400	*	226	147	2
Potri.006	Chr06	16559802	16560015	214	*	175	117	4
Potri.006	Chr06	16917221	16917455	235	*	230	95	5
Potri.006	Chr06	17170194	17170426	233	*	293	120	5
Potri.006	Chr06	17649872	17650085	214	*	265	108	3
Potri.006	Chr06	17843536	17843779	244	*	255	129	5
Potri.006	Chr06	18384987	18385317	331	*	203	136	4
Potri.006	Chr06	18431559	18431785	227	*	211	127	5
Potri.006	Chr06	18632759	18632964	206	*	402	103	3
Potri.006	Chr06	18914230	18914503	274	*	273	135	3
Potri.006	Chr06	19020137	19020395	259	*	393	140	7
Potri.006	Chr06	19392821	19393162	342	*	237	153	5
Potri.006	Chr06	19624832	19625138	307	*	237	171	3
Potri.006	Chr06	20042067	20042292	226	*	210	109	4
Potri.006	Chr06	20104158	20104396	239	*	210	108	4
Potri.006	Chr06	20917537	20917825	289	*	192	152	4

Potri.006	Chr06	21054464	21054675	212	*	169	94	5
Potri.006	Chr06	21209585	21210084	500	*	539	136	3
Potri.006	Chr06	21506471	21506884	414	*	178	235	3
Potri.006	Chr06	21740868	21741446	579	*	354	171	2
Potri.006	Chr06	22052388	22052680	293	*	254	139	4
Potri.006	Chr06	22273120	22273348	229	*	187	129	4
Potri.006	Chr06	22659554	22660043	490	*	305	336	3
Potri.006	Chr06	23468586	23468850	265	*	192	114	4
Potri.006	Chr06	23738928	23739283	356	*	272	220	4
Potri.006	Chr06	23957814	23958052	239	*	181	83	4
Potri.006	Chr06	24408878	24409099	222	*	188	115	5
Potri.006	Chr06	24640228	24640456	229	*	193	83	4
Potri.006	Chr06	24678458	24678699	242	*	180	121	5
Potri.006	Chr06	24981985	24982269	285	*	172	151	3
Potri.006	Chr06	24995211	24995418	208	*	200	66	3
Potri.006	Chr06	25124500	25125066	567	*	198	118	4
Potri.006	Chr06	25777276	25777534	259	*	175	173	4
Potri.006	Chr06	25939969	25940230	262	*	283	125	5
Potri.006	Chr06	26231450	26231668	219	*	169	76	5
Potri.006	Chr06	26473563	26473799	237	*	173	142	4
Potri.006	Chr06	26635645	26636123	479	*	209	152	3
Potri.006	Chr06	26742203	26742449	247	*	197	112	4
Potri.006	Chr06	26749817	26750098	282	*	168	168	3
Potri.006	Chr06	27446377	27446604	228	*	183	101	4
Potri.006	Chr06	27625800	27626272	473	*	219	341	3
Potri.006	Chr06	27739264	27739700	437	*	248	249	4
Potri.007	Chr07	215002	215218	217	*	189	89	4
Potri.007	Chr07	1007548	1007805	258	*	175	109	4
Potri.007	Chr07	1445906	1446148	243	*	210	135	5
Potri.007	Chr07	1640840	1641077	238	*	195	117	4
Potri.007	Chr07	2418046	2418260	215	*	216	120	4
Potri.007	Chr07	2828561	2828893	333	*	192	163	4
Potri.007	Chr07	3252335	3252665	331	*	545	163	3
Potri.007	Chr07	3370763	3370986	224	*	195	66	5
Potri.007	Chr07	3419571	3419778	208	*	168	88	5
Potri.007	Chr07	3578358	3578564	207	*	187	66	4
Potri.007	Chr07	3779481	3779747	267	*	168	103	3
Potri.007	Chr07	4771133	4771454	322	*	260	135	2
Potri.007	Chr07	5102747	5102963	217	*	195	110	5
Potri.007	Chr07	5261432	5261701	270	*	197	148	3
Potri.007	Chr07	5675940	5676165	226	*	183	119	4
Potri.007	Chr07	5974247	5974468	222	*	240	105	2
Potri.007	Chr07	6537769	6538198	430	*	273	273	2
Potri.007	Chr07	6538971	6539635	665	*	168	489	2
Potri.007	Chr07	6679545	6679752	208	*	238	121	3
Potri.007	Chr07	7247424	7247633	210	*	295	140	3
Potri.007	Chr07	7978966	7979178	213	*	267	97	3
Potri.007	Chr07	8147070	8147271	202	*	172	96	3
Potri.007	Chr07	8208911	8209304	394	*	349	285	4
Potri.007	Chr07	8227544	8227791	248	*	238	133	4
Potri.007	Chr07	8773896	8774162	267	*	294	114	6
Potri.007	Chr07	9399337	9399612	276	*	188	123	3
Potri.007	Chr07	9476536	9476806	271	*	236	140	4
Potri.007	Chr07	10285200	10285426	227	*	611	114	3

Potri.007	Chr07	10416143	10416378	236	*	239	116	5
Potri.007	Chr07	10547920	10548225	306	*	215	111	4
Potri.007	Chr07	11112375	11112619	245	*	236	95	4
Potri.007	Chr07	11157074	11157319	246	*	209	128	5
Potri.007	Chr07	11193598	11194041	444	*	168	157	3
Potri.007	Chr07	11690352	11691097	746	*	208	605	4
Potri.007	Chr07	11709005	11709440	436	*	291	243	5
Potri.007	Chr07	12137475	12137931	457	*	171	349	4
Potri.007	Chr07	12164501	12164717	217	*	200	111	3
Potri.007	Chr07	12528340	12528658	319	*	175	147	4
Potri.007	Chr07	12666281	12666788	508	*	178	329	3
Potri.007	Chr07	12664165	12664512	348	*	232	149	3
Potri.007	Chr07	12684523	12684810	288	*	174	142	3
Potri.007	Chr07	12706167	12706396	230	*	335	122	5
Potri.007	Chr07	12942033	12942311	279	*	242	157	4
Potri.007	Chr07	13002359	13002595	237	*	225	96	4
Potri.007	Chr07	13278533	13278851	319	*	172	149	3
Potri.007	Chr07	13311166	13311725	560	*	254	144	3
Potri.007	Chr07	13759072	13759306	235	*	463	128	3
Potri.007	Chr07	14210620	14210867	248	*	309	134	3
Potri.007	Chr07	14911290	14911517	228	*	175	115	4
Potri.007	Chr07	15009448	15009696	249	*	256	105	4
Potri.007	Chr07	15161595	15161873	279	*	184	124	2
Potri.007	Chr07	15183506	15183715	210	*	275	135	3
Potri.007	Chr07	15318082	15318344	263	*	274	149	3
Potri.007	Chr07	15358736	15359012	277	*	201	170	3
Potri.007	Chr07	15578567	15578807	241	*	169	139	5
Potri.008	Chr08	380068	380344	277	*	383	148	2
Potri.008	Chr08	1517054	1517272	219	*	206	128	5
Potri.008	Chr08	1778725	1779103	379	*	207	211	3
Potri.008	Chr08	1991147	1991369	223	*	174	126	3
Potri.008	Chr08	2006673	2006911	239	*	175	103	4
Potri.008	Chr08	2803550	2803896	347	*	197	173	3
Potri.008	Chr08	2815018	2815236	219	*	393	101	3
Potri.008	Chr08	3005419	3005655	237	*	190	125	4
Potri.008	Chr08	3021282	3021964	683	*	177	557	4
Potri.008	Chr08	3052261	3052598	338	*	177	198	3
Potri.008	Chr08	3167109	3167528	420	*	179	334	3
Potri.008	Chr08	3222318	3222538	221	*	174	134	3
Potri.008	Chr08	3232637	3233074	438	*	173	128	3
Potri.008	Chr08	3567336	3567589	254	*	169	115	4
Potri.008	Chr08	3616057	3616266	210	*	230	92	3
Potri.008	Chr08	3695582	3695794	213	*	377	111	3
Potri.008	Chr08	3781586	3781857	272	*	284	108	4
Potri.008	Chr08	4476890	4477540	651	*	196	377	3
Potri.008	Chr08	4516661	4516885	225	*	201	121	4
Potri.008	Chr08	4868833	4869112	280	*	191	142	3
Potri.008	Chr08	5214475	5214697	223	*	195	131	5
Potri.008	Chr08	5814300	5814599	300	*	324	150	4
Potri.008	Chr08	6070852	6071099	248	*	187	139	4
Potri.008	Chr08	6163801	6164285	485	*	210	374	5
Potri.008	Chr08	6625940	6626158	219	*	170	90	4
Potri.008	Chr08	6923411	6923620	210	*	172	97	3
Potri.008	Chr08	6934754	6935026	273	*	228	144	3

Potri.008	Chr08	7116352	7116576	225	*	238	90	3
Potri.008	Chr08	7178983	7179270	288	*	214	144	4
Potri.008	Chr08	7526344	7526602	259	*	191	129	3
Potri.008	Chr08	7694965	7695284	320	*	176	170	3
Potri.008	Chr08	7843464	7843712	249	*	228	154	4
Potri.008	Chr08	8006635	8006896	262	*	206	115	4
Potri.008	Chr08	8012196	8012437	242	*	175	133	4
Potri.008	Chr08	8024917	8025172	256	*	229	140	4
Potri.008	Chr08	8187275	8187895	621	*	183	430	3
Potri.008	Chr08	8492893	8493283	391	*	224	256	5
Potri.008	Chr08	8631360	8631653	294	*	236	164	4
Potri.008	Chr08	9127795	9128023	229	*	177	137	4
Potri.008	Chr08	9315899	9316213	315	*	177	139	4
Potri.008	Chr08	9463258	9463571	314	*	178	180	3
Potri.008	Chr08	9667604	9668102	499	*	202	347	3
Potri.008	Chr08	9867129	9867368	240	*	192	135	4
Potri.008	Chr08	10168934	10169234	301	*	211	136	4
Potri.008	Chr08	10457420	10457640	221	*	208	110	4
Potri.008	Chr08	10519890	10520114	225	*	191	112	4
Potri.008	Chr08	10604140	10604355	216	*	170	110	4
Potri.008	Chr08	10762998	10763241	244	*	184	117	4
Potri.008	Chr08	11092667	11092917	251	*	263	127	3
Potri.008	Chr08	11143788	11144006	219	*	177	129	4
Potri.008	Chr08	11910275	11910530	256	*	203	121	4
Potri.008	Chr08	11999679	11999911	233	*	179	99	3
Potri.008	Chr08	12093355	12093667	313	*	330	169	5
Potri.008	Chr08	12250450	12250685	236	*	168	113	3
Potri.008	Chr08	12401111	12401412	302	*	200	154	3
Potri.008	Chr08	12419753	12420024	272	*	225	127	4
Potri.008	Chr08	12944908	12945139	232	*	215	130	4
Potri.008	Chr08	13021089	13021310	222	*	190	107	4
Potri.008	Chr08	13902027	13902238	212	*	203	106	4
Potri.008	Chr08	14042253	14042477	225	*	285	104	4
Potri.008	Chr08	14569377	14569892	516	*	192	137	3
Potri.008	Chr08	15127483	15127814	332	*	294	169	4
Potri.008	Chr08	15334299	15334509	211	*	173	136	4
Potri.008	Chr08	15425938	15426150	213	*	176	99	4
Potri.008	Chr08	15732436	15732641	206	*	176	90	3
Potri.008	Chr08	17238863	17239184	322	*	308	151	5
Potri.008	Chr08	17483540	17484020	481	*	263	126	3
Potri.008	Chr08	17694815	17695195	381	*	222	187	3
Potri.008	Chr08	18376747	18377055	309	*	186	136	4
Potri.008	Chr08	18547209	18547692	484	*	173	391	4
Potri.008	Chr08	18779671	18780309	639	*	469	452	2
Potri.009	Chr09	188074	188275	202	*	288	138	3
Potri.009	Chr09	250842	251093	252	*	447	103	2
Potri.009	Chr09	414081	414302	222	*	554	119	3
Potri.009	Chr09	987828	988123	296	*	616	172	3
Potri.009	Chr09	991184	991712	529	*	393	351	2
Potri.009	Chr09	1270607	1271020	414	*	190	239	3
Potri.009	Chr09	1385409	1385620	212	*	299	82	3
Potri.009	Chr09	2723688	2723933	246	*	697	138	3
Potri.009	Chr09	2727338	2727635	298	*	673	147	3
Potri.009	Chr09	2730027	2730244	218	*	523	129	3

Potri.009	Chr09	3163812	3164406	595	*	171	139	3
Potri.009	Chr09	3309805	3310102	298	*	197	138	5
Potri.009	Chr09	3954995	3955216	222	*	223	117	5
Potri.009	Chr09	3984696	3984937	242	*	175	114	5
Potri.009	Chr09	4111819	4112061	243	*	254	130	4
Potri.009	Chr09	5384670	5384973	304	*	170	160	4
Potri.009	Chr09	5482353	5482584	232	*	170	139	4
Potri.009	Chr09	5494478	5494708	231	*	181	81	4
Potri.009	Chr09	5853196	5853452	257	*	180	137	3
Potri.009	Chr09	6343430	6343688	259	*	182	107	4
Potri.009	Chr09	6591488	6591822	335	*	180	144	3
Potri.009	Chr09	6860119	6860380	262	*	247	118	4
Potri.009	Chr09	7143408	7143679	272	*	555	159	3
Potri.009	Chr09	7519930	7520201	272	*	177	167	4
Potri.009	Chr09	7584982	7585234	253	*	197	139	4
Potri.009	Chr09	8620824	8621313	490	*	332	136	2
Potri.009	Chr09	9092890	9093121	232	*	224	99	4
Potri.009	Chr09	9383280	9383659	380	*	222	210	4
Potri.009	Chr09	9497050	9497371	322	*	250	195	3
Potri.009	Chr09	9895910	9896214	305	*	226	156	5
Potri.009	Chr09	10213863	10214125	263	*	192	94	4
Potri.009	Chr09	10239971	10240444	474	*	190	117	3
Potri.009	Chr09	10435910	10436209	300	*	230	153	5
Potri.009	Chr09	10521301	10521524	224	*	195	74	5
Potri.009	Chr09	11366307	11367012	706	*	262	578	4
Potri.009	Chr09	11403385	11403734	350	*	314	158	4
Potri.009	Chr09	11512645	11512881	237	*	175	104	5
Potri.009	Chr09	11793301	11793562	262	*	187	135	4
Potri.009	Chr09	11983524	11983734	211	*	212	76	3
Potri.009	Chr09	12026918	12027409	492	*	205	313	3
Potri.009	Chr09	12164492	12164899	408	*	170	197	3
Potri.009	Chr09	12341523	12341777	255	*	205	129	5
Potri.009	Chr09	12948280	12948482	203	*	183	105	3
Potri.010	Chr10	497365	497581	217	*	197	98	3
Potri.010	Chr10	678554	678782	229	*	175	126	3
Potri.010	Chr10	722688	722932	245	*	238	120	3
Potri.010	Chr10	1233077	1233325	249	*	274	104	4
Potri.010	Chr10	1823823	1824314	492	*	170	161	3
Potri.010	Chr10	1975920	1976129	210	*	200	125	3
Potri.010	Chr10	2849717	2849988	272	*	195	133	5
Potri.010	Chr10	3031885	3032157	273	*	180	150	3
Potri.010	Chr10	3544480	3544716	237	*	219	132	4
Potri.010	Chr10	3731846	3732057	212	*	520	121	4
Potri.010	Chr10	3994352	3994555	204	*	200	121	3
Potri.010	Chr10	4095017	4095269	253	*	211	141	4
Potri.010	Chr10	4213200	4213410	211	*	720	85	5
Potri.010	Chr10	4424864	4425127	264	*	237	152	5
Potri.010	Chr10	4752260	4752479	220	*	372	100	3
Potri.010	Chr10	4770043	4770267	225	*	313	136	3
Potri.010	Chr10	5229777	5230032	256	*	1035	127	3
Potri.010	Chr10	5616028	5616276	249	*	379	75	5
Potri.010	Chr10	5747999	5748440	442	*	223	191	2
Potri.010	Chr10	7325234	7325860	627	*	182	332	3
Potri.010	Chr10	7606293	7606602	310	*	222	187	4

Potri.010	Chr10	8333077	8333733	657	*	505	193	2
Potri.010	Chr10	8334683	8334895	213	*	282	105	3
Potri.010	Chr10	8934311	8934521	211	*	183	101	2
Potri.010	Chr10	9484605	9484811	207	*	171	100	4
Potri.010	Chr10	10002568	10002868	301	*	196	169	3
Potri.010	Chr10	10019919	10020132	214	*	197	112	4
Potri.010	Chr10	10140443	10140767	325	*	221	182	3
Potri.010	Chr10	10751840	10752202	363	*	177	184	3
Potri.010	Chr10	11301952	11302389	438	*	207	333	2
Potri.010	Chr10	11672674	11673043	370	*	172	289	3
Potri.010	Chr10	11758523	11758757	235	*	170	133	4
Potri.010	Chr10	11849113	11849384	272	*	208	95	4
Potri.010	Chr10	11921708	11921973	266	*	205	144	3
Potri.010	Chr10	12305197	12305720	524	*	206	381	3
Potri.010	Chr10	12506727	12507011	285	*	299	132	4
Potri.010	Chr10	12523879	12524339	461	*	193	96	4
Potri.010	Chr10	12833280	12833517	238	*	175	131	5
Potri.010	Chr10	13059681	13060640	960	*	180	533	3
Potri.010	Chr10	13109552	13109776	225	*	178	117	4
Potri.010	Chr10	13134263	13134479	217	*	192	126	3
Potri.010	Chr10	13192922	13193151	230	*	215	138	5
Potri.010	Chr10	13648842	13649046	205	*	171	99	4
Potri.010	Chr10	13736434	13736715	282	*	201	144	5
Potri.010	Chr10	14105226	14105468	243	*	240	84	5
Potri.010	Chr10	14666986	14667319	334	*	192	172	4
Potri.010	Chr10	14800777	14801185	409	*	181	196	4
Potri.010	Chr10	14858688	14858915	228	*	259	115	4
Potri.010	Chr10	15255414	15255630	217	*	170	113	4
Potri.010	Chr10	15401212	15401604	393	*	232	152	3
Potri.010	Chr10	15939303	15939546	244	*	215	152	5
Potri.010	Chr10	15942135	15942394	260	*	169	108	4
Potri.010	Chr10	16024808	16025131	324	*	225	167	4
Potri.010	Chr10	16321285	16321520	236	*	306	139	4
Potri.010	Chr10	16512627	16512855	229	*	214	122	4
Potri.010	Chr10	17008348	17008593	246	*	258	118	4
Potri.010	Chr10	17100301	17100667	367	*	172	177	3
Potri.010	Chr10	17847643	17847900	258	*	208	110	4
Potri.010	Chr10	18061438	18061673	236	*	262	101	4
Potri.010	Chr10	18069815	18070209	395	*	335	142	5
Potri.010	Chr10	18450658	18450875	218	*	169	97	3
Potri.010	Chr10	18934795	18935190	396	*	214	169	4
Potri.010	Chr10	19155725	19156085	361	*	312	207	3
Potri.010	Chr10	19349673	19349889	217	*	225	121	4
Potri.010	Chr10	19411340	19411678	339	*	263	143	4
Potri.010	Chr10	19603286	19603787	502	*	393	371	7
Potri.010	Chr10	19765326	19765557	232	*	410	121	3
Potri.010	Chr10	19862948	19863251	304	*	175	152	4
Potri.010	Chr10	19930641	19930935	295	*	169	138	3
Potri.010	Chr10	19944842	19945043	202	*	189	97	3
Potri.010	Chr10	20360992	20361237	246	*	187	129	4
Potri.010	Chr10	20656404	20656624	221	*	178	100	4
Potri.010	Chr10	20705546	20705828	283	*	236	150	4
Potri.010	Chr10	20746208	20746443	236	*	206	91	4
Potri.010	Chr10	20849709	20850172	464	*	171	135	3

Potri.010	Chr10	20955244	20955477	234	*	208	127	4
Potri.010	Chr10	21026064	21026341	278	*	170	141	3
Potri.010	Chr10	21452941	21453146	206	*	182	98	4
Potri.010	Chr10	22181052	22181261	210	*	257	90	3
Potri.010	Chr10	22407911	22408186	276	*	168	136	3
Potri.010	Chr10	22493115	22493344	230	*	216	139	3
Potri.011	Chr11	280384	280607	224	*	180	119	5
Potri.011	Chr11	1009365	1009585	221	*	481	103	3
Potri.011	Chr11	1017380	1017592	213	*	299	137	3
Potri.011	Chr11	1293925	1294164	240	*	207	131	4
Potri.011	Chr11	1778243	1778491	249	*	169	115	5
Potri.011	Chr11	3264209	3264449	241	*	197	92	3
Potri.011	Chr11	4065262	4065549	288	*	212	118	5
Potri.011	Chr11	4080175	4080404	230	*	193	123	3
Potri.011	Chr11	4098306	4098598	293	*	210	154	5
Potri.011	Chr11	4179519	4179735	217	*	173	144	3
Potri.011	Chr11	4753803	4754005	203	*	188	120	3
Potri.011	Chr11	5675391	5675644	254	*	699	123	3
Potri.011	Chr11	5810621	5810850	230	*	194	117	3
Potri.011	Chr11	6675904	6676283	380	*	226	266	3
Potri.011	Chr11	7193887	7194291	405	*	729	279	3
Potri.011	Chr11	7195749	7196120	372	*	503	219	3
Potri.011	Chr11	7197098	7197582	485	*	482	258	2
Potri.011	Chr11	7199081	7199905	825	*	277	369	2
Potri.011	Chr11	7204585	7204800	216	*	389	100	4
Potri.011	Chr11	7206727	7206931	205	*	506	137	4
Potri.011	Chr11	7216984	7217188	205	*	488	93	3
Potri.011	Chr11	7232108	7232312	205	*	215	125	3
Potri.011	Chr11	7730587	7730793	207	*	180	99	2
Potri.011	Chr11	8004416	8004657	242	*	223	86	5
Potri.011	Chr11	8281481	8281709	229	*	422	106	3
Potri.011	Chr11	8750210	8750433	224	*	206	98	5
Potri.011	Chr11	8894785	8895003	219	*	175	116	5
Potri.011	Chr11	9968424	9968745	322	*	173	184	3
Potri.011	Chr11	10768971	10769219	249	*	188	129	5
Potri.011	Chr11	11041237	11042233	997	*	183	627	2
Potri.011	Chr11	11085311	11085559	249	*	297	141	4
Potri.011	Chr11	11646597	11646832	236	*	208	118	3
Potri.011	Chr11	12064035	12064249	215	*	303	104	3
Potri.011	Chr11	12868168	12868389	222	*	177	151	4
Potri.011	Chr11	13360491	13360787	297	*	187	167	4
Potri.011	Chr11	13370910	13371148	239	*	229	127	5
Potri.011	Chr11	13389192	13389676	485	*	225	288	3
Potri.011	Chr11	13861283	13861491	209	*	380	128	3
Potri.011	Chr11	14619743	14619978	236	*	188	113	3
Potri.011	Chr11	14663973	14664237	265	*	252	159	3
Potri.011	Chr11	15559310	15559948	639	*	177	432	3
Potri.011	Chr11	15681919	15682469	551	*	184	178	3
Potri.011	Chr11	15719434	15720275	842	*	272	715	5
Potri.011	Chr11	15776698	15777122	425	*	250	144	4
Potri.011	Chr11	16055882	16056093	212	*	411	107	3
Potri.011	Chr11	16096299	16096580	282	*	179	158	3
Potri.011	Chr11	16139170	16139431	262	*	197	132	3
Potri.011	Chr11	16376771	16376989	219	*	229	97	4

Potri.011Chr11	16537079	16537513	435	*	169	293	3
Potri.011Chr11	16787551	16787780	230	*	197	100	5
Potri.011Chr11	16859757	16860001	245	*	177	106	4
Potri.011Chr11	16941564	16941860	297	*	272	166	5
Potri.011Chr11	17248568	17248796	229	*	175	108	5
Potri.011Chr11	17976364	17976604	241	*	177	132	3
Potri.011Chr11	18050016	18050691	676	*	277	498	3
Potri.012Chr12	279345	279683	339	*	187	166	3
Potri.012Chr12	429733	429953	221	*	229	105	4
Potri.012Chr12	774277	774502	226	*	198	85	3
Potri.012Chr12	878444	878674	231	*	232	121	3
Potri.012Chr12	1662939	1663170	232	*	242	103	4
Potri.012Chr12	1700840	1701206	367	*	210	229	3
Potri.012Chr12	1770217	1770546	330	*	204	177	4
Potri.012Chr12	1787499	1787713	215	*	168	124	3
Potri.012Chr12	1875727	1875929	203	*	196	82	3
Potri.012Chr12	2051145	2051446	302	*	192	191	3
Potri.012Chr12	2142008	2142234	227	*	181	125	3
Potri.012Chr12	3346451	3346697	247	*	202	143	4
Potri.012Chr12	3633292	3633510	219	*	175	91	5
Potri.012Chr12	3679420	3679644	225	*	176	125	4
Potri.012Chr12	3767955	3768175	221	*	195	115	5
Potri.012Chr12	3785404	3785650	247	*	285	105	4
Potri.012Chr12	3846984	3847217	234	*	291	116	6
Potri.012Chr12	3891244	3891507	264	*	254	133	4
Potri.012Chr12	4989235	4989446	212	*	183	75	4
Potri.012Chr12	5405245	5405472	228	*	177	126	4
Potri.012Chr12	5439591	5439877	287	*	186	146	3
Potri.012Chr12	5532896	5533125	230	*	185	118	2
Potri.012Chr12	6265498	6265770	273	*	243	152	5
Potri.012Chr12	6891658	6891862	205	*	185	132	3
Potri.012Chr12	7038069	7038330	262	*	174	121	3
Potri.012Chr12	7087561	7087907	347	*	716	200	3
Potri.012Chr12	7487675	7487882	208	*	238	97	4
Potri.012Chr12	8367004	8367382	379	*	341	202	4
Potri.012Chr12	8481537	8481822	286	*	258	160	4
Potri.012Chr12	8502904	8503165	262	*	249	128	4
Potri.012Chr12	8845202	8845403	202	*	238	84	4
Potri.012Chr12	9073318	9073533	216	*	192	84	3
Potri.012Chr12	9215974	9216704	731	*	171	572	3
Potri.012Chr12	9825741	9826156	416	*	173	270	3
Potri.012Chr12	9832325	9832546	222	*	191	132	4
Potri.012Chr12	9850644	9850876	233	*	209	95	5
Potri.012Chr12	9901810	9902091	282	*	199	143	3
Potri.012Chr12	9967460	9967687	228	*	204	105	5
Potri.012Chr12	10013792	10014124	333	*	185	134	3
Potri.012Chr12	10140422	10140680	259	*	175	119	5
Potri.012Chr12	10421124	10421343	220	*	264	92	5
Potri.012Chr12	10490328	10490562	235	*	217	123	3
Potri.012Chr12	10531993	10532249	257	*	299	133	5
Potri.012Chr12	10777583	10777798	216	*	331	124	3
Potri.012Chr12	10956219	10956847	629	*	176	474	3
Potri.012Chr12	11046450	11046815	366	*	265	196	4
Potri.012Chr12	11144365	11144651	287	*	353	137	6

Potri.012	Chr12	11245027	11245453	427	*	170	136	3
Potri.012	Chr12	11349177	11349416	240	*	238	159	4
Potri.012	Chr12	11453611	11453843	233	*	203	114	4
Potri.012	Chr12	11589057	11589322	266	*	287	120	3
Potri.012	Chr12	11684858	11685336	479	*	191	144	3
Potri.012	Chr12	12014961	12015164	204	*	183	91	3
Potri.012	Chr12	12312788	12313203	416	*	333	204	3
Potri.012	Chr12	12359956	12360182	227	*	187	70	4
Potri.012	Chr12	12374717	12374919	203	*	200	105	3
Potri.012	Chr12	12421610	12421831	222	*	198	95	3
Potri.012	Chr12	12555547	12555862	316	*	209	172	3
Potri.012	Chr12	12772752	12772972	221	*	188	98	5
Potri.012	Chr12	12872671	12872880	210	*	303	116	3
Potri.012	Chr12	12897468	12897762	295	*	209	112	3
Potri.012	Chr12	13015456	13015722	267	*	249	150	4
Potri.012	Chr12	13055843	13056237	395	*	171	185	2
Potri.012	Chr12	13079362	13079582	221	*	188	125	5
Potri.012	Chr12	13224097	13224406	310	*	182	154	3
Potri.012	Chr12	13280498	13280720	223	*	177	122	4
Potri.012	Chr12	13707896	13708149	254	*	240	165	4
Potri.012	Chr12	13761533	13761756	224	*	195	86	5
Potri.012	Chr12	13977853	13978097	245	*	197	88	3
Potri.012	Chr12	14103319	14103535	217	*	169	145	5
Potri.012	Chr12	14120146	14120661	516	*	226	200	3
Potri.012	Chr12	14198834	14199046	213	*	169	99	3
Potri.012	Chr12	14245017	14245276	260	*	199	125	4
Potri.012	Chr12	14279764	14280003	240	*	214	131	3
Potri.012	Chr12	14356166	14356442	277	*	183	131	3
Potri.012	Chr12	14376476	14376687	212	*	169	93	5
Potri.012	Chr12	14456793	14457321	529	*	196	396	3
Potri.012	Chr12	14736852	14737208	357	*	175	185	3
Potri.012	Chr12	14780382	14780601	220	*	193	95	4
Potri.012	Chr12	14964775	14965018	244	*	169	126	3
Potri.012	Chr12	14973069	14973402	334	*	195	154	3
Potri.012	Chr12	15033803	15034026	224	*	172	100	4
Potri.012	Chr12	15087189	15087401	213	*	206	119	3
Potri.012	Chr12	15116262	15116476	215	*	199	106	3
Potri.012	Chr12	15123772	15124171	400	*	240	194	3
Potri.012	Chr12	15168580	15168920	341	*	347	180	3
Potri.012	Chr12	15239700	15239936	237	*	192	128	4
Potri.012	Chr12	15380375	15380653	279	*	230	143	5
Potri.012	Chr12	15463451	15463680	230	*	223	116	4
Potri.012	Chr12	15582553	15582973	421	*	172	170	3
Potri.012	Chr12	15624082	15624313	232	*	189	146	3
Potri.012	Chr12	15706156	15706422	267	*	186	77	3
Potri.013	Chr13	586923	587127	205	*	173	110	4
Potri.013	Chr13	635381	635924	544	*	210	163	3
Potri.013	Chr13	645484	645906	423	*	192	248	3
Potri.013	Chr13	792708	793014	307	*	182	180	4
Potri.013	Chr13	1139200	1139429	230	*	270	102	5
Potri.013	Chr13	1437542	1437829	288	*	183	188	3
Potri.013	Chr13	1676299	1676684	386	*	360	148	2
Potri.013	Chr13	1700265	1700574	310	*	204	136	4
Potri.013	Chr13	1738462	1738744	283	*	208	112	3

Potri.013Chr13	2470014	2470260	247 *	212	106	4
Potri.013Chr13	2500987	2501341	355 *	236	199	3
Potri.013Chr13	2904593	2904806	214 *	178	85	4
Potri.013Chr13	3360468	3360706	239 *	219	111	3
Potri.013Chr13	3621400	3621688	289 *	277	144	4
Potri.013Chr13	3632923	3633188	266 *	216	157	4
Potri.013Chr13	3779731	3780132	402 *	187	296	3
Potri.013Chr13	3992374	3992719	346 *	281	193	4
Potri.013Chr13	4277138	4277584	447 *	180	344	3
Potri.013Chr13	4567578	4567969	392 *	188	257	3
Potri.013Chr13	5296954	5297207	254 *	642	123	3
Potri.013Chr13	5605976	5606245	270 *	200	152	4
Potri.013Chr13	5967622	5967910	289 *	203	163	4
Potri.013Chr13	5973873	5974118	246 *	212	129	3
Potri.013Chr13	6402281	6402538	258 *	402	130	2
Potri.013Chr13	6403918	6404394	477 *	678	148	3
Potri.013Chr13	6408437	6409840	1404 *	306	589	2
Potri.013Chr13	6410049	6410744	696 *	304	511	2
Potri.013Chr13	6414272	6414582	311 *	434	187	2
Potri.013Chr13	6539352	6539662	311 *	181	183	3
Potri.013Chr13	8397107	8397424	318 *	226	138	3
Potri.013Chr13	8553850	8554074	225 *	170	83	4
Potri.013Chr13	8676632	8676913	282 *	181	160	3
Potri.013Chr13	9592001	9592225	225 *	337	124	2
Potri.013Chr13	9857166	9857556	391 *	434	199	2
Potri.013Chr13	10263688	10263962	275 *	205	137	4
Potri.013Chr13	10308267	10308536	270 *	228	124	4
Potri.013Chr13	10321529	10321772	244 *	259	104	5
Potri.013Chr13	10736166	10736404	239 *	489	121	4
Potri.013Chr13	10871684	10871887	204 *	180	94	4
Potri.013Chr13	11150564	11150783	220 *	188	119	5
Potri.013Chr13	11190269	11190503	235 *	193	139	5
Potri.013Chr13	11656693	11657086	394 *	196	123	3
Potri.013Chr13	11677172	11677583	412 *	168	160	3
Potri.013Chr13	12163662	12163870	209 *	173	108	4
Potri.013Chr13	12366040	12366491	452 *	208	156	3
Potri.013Chr13	12676259	12676531	273 *	197	139	3
Potri.013Chr13	12942050	12942787	738 *	173	637	3
Potri.013Chr13	13253798	13254033	236 *	201	103	3
Potri.013Chr13	13614224	13614445	222 *	246	87	3
Potri.013Chr13	13699039	13699440	402 *	191	152	3
Potri.013Chr13	13967624	13967834	211 *	516	102	3
Potri.013Chr13	14041668	14041951	284 *	237	119	4
Potri.013Chr13	14106801	14107231	431 *	177	330	4
Potri.013Chr13	14522867	14523113	247 *	382	120	3
Potri.013Chr13	14660552	14661673	1122 *	546	140	2
Potri.013Chr13	14661984	14662610	627 *	258	237	2
Potri.013Chr13	14662902	14663136	235 *	815	112	3
Potri.013Chr13	14665264	14666214	951 *	349	183	2
Potri.013Chr13	14666392	14667221	830 *	271	209	2
Potri.013Chr13	14684602	14685046	445 *	218	352	3
Potri.013Chr13	14752511	14753353	843 *	610	664	3
Potri.013Chr13	14753839	14754066	228 *	743	106	3
Potri.013Chr13	14754849	14755531	683 *	434	510	2

Potri.013	Chr13	14756198	14756471	274	*	367	191	2
Potri.013	Chr13	14757953	14758362	410	*	737	232	3
Potri.013	Chr13	14767526	14767773	248	*	915	134	3
Potri.013	Chr13	14770079	14770661	583	*	433	380	2
Potri.013	Chr13	14772548	14772884	337	*	508	174	2
Potri.013	Chr13	14775557	14775947	391	*	430	226	2
Potri.013	Chr13	14779113	14779325	213	*	597	83	4
Potri.013	Chr13	14785259	14785561	303	*	1228	140	4
Potri.013	Chr13	14788608	14789177	570	*	277	249	2
Potri.013	Chr13	14790886	14791178	293	*	607	127	3
Potri.013	Chr13	14792096	14792313	218	*	280	114	2
Potri.013	Chr13	14793673	14795142	1470	*	318	598	2
Potri.013	Chr13	14814110	14814340	231	*	459	125	3
Potri.013	Chr13	14826626	14826967	342	*	927	150	3
Potri.013	Chr13	14828531	14828784	254	*	687	146	3
Potri.013	Chr13	14831933	14832457	525	*	863	379	3
Potri.013	Chr13	14836049	14836265	217	*	857	89	3
Potri.013	Chr13	14836649	14836865	217	*	624	131	3
Potri.013	Chr13	14838921	14839387	467	*	615	243	3
Potri.013	Chr13	14842253	14842764	512	*	441	316	2
Potri.013	Chr13	14847329	14847686	358	*	380	154	2
Potri.013	Chr13	14849115	14850094	980	*	302	384	2
Potri.013	Chr13	14851957	14852325	369	*	546	160	3
Potri.013	Chr13	14853535	14853922	388	*	470	245	2
Potri.013	Chr13	14865360	14865596	237	*	358	97	2
Potri.013	Chr13	14866330	14866592	263	*	544	113	3
Potri.013	Chr13	14869475	14869947	473	*	341	333	2
Potri.013	Chr13	14878722	14879021	300	*	1015	157	3
Potri.013	Chr13	14879676	14880782	1107	*	451	180	2
Potri.013	Chr13	14881749	14882282	534	*	1017	377	3
Potri.013	Chr13	14883095	14883314	220	*	857	74	4
Potri.013	Chr13	14896132	14896381	250	*	747	128	3
Potri.013	Chr13	14907355	14907572	218	*	392	39	3
Potri.013	Chr13	14987548	14987800	253	*	203	135	4
Potri.013	Chr13	15389644	15390011	368	*	194	212	3
Potri.013	Chr13	15451378	15451617	240	*	182	137	3
Potri.013	Chr13	15643150	15643379	230	*	180	111	5
Potri.013	Chr13	15657913	15658151	239	*	175	102	4
Potri.013	Chr13	15717746	15717984	239	*	247	117	4
Potri.013	Chr13	15836524	15836740	217	*	374	100	3
Potri.013	Chr13	15903178	15903390	213	*	174	86	3
Potri.013	Chr13	16012205	16012458	254	*	180	87	3
Potri.013	Chr13	16069810	16070054	245	*	239	132	4
Potri.013	Chr13	16164331	16164605	275	*	281	148	6
Potri.014	Chr14	735918	736142	225	*	181	133	4
Potri.014	Chr14	1320621	1320867	247	*	242	93	5
Potri.014	Chr14	1492151	1492456	306	*	357	141	6
Potri.014	Chr14	1637553	1637791	239	*	191	123	3
Potri.014	Chr14	2172135	2172369	235	*	186	124	4
Potri.014	Chr14	3331410	3331703	294	*	193	146	4
Potri.014	Chr14	3441507	3441898	392	*	468	195	4
Potri.014	Chr14	3744572	3744802	231	*	181	104	4
Potri.014	Chr14	4120073	4120774	702	*	204	183	3
Potri.014	Chr14	4240183	4240396	214	*	261	107	3

Potri.014Chr14	4247627	4247894	268	*	309	131	4
Potri.014Chr14	4256216	4256475	260	*	286	100	3
Potri.014Chr14	4261708	4261964	257	*	243	127	3
Potri.014Chr14	4504359	4504665	307	*	170	151	4
Potri.014Chr14	4654402	4654680	279	*	186	140	4
Potri.014Chr14	4749908	4750303	396	*	250	274	3
Potri.014Chr14	4771968	4772217	250	*	230	136	5
Potri.014Chr14	5356907	5357219	313	*	192	139	4
Potri.014Chr14	5539560	5539837	278	*	183	123	3
Potri.014Chr14	6232305	6232575	271	*	177	117	3
Potri.014Chr14	6386497	6386736	240	*	287	104	4
Potri.014Chr14	6431184	6431637	454	*	357	121	3
Potri.014Chr14	6549183	6549642	460	*	171	167	3
Potri.014Chr14	6598631	6599200	570	*	185	148	3
Potri.014Chr14	6975156	6975365	210	*	377	92	3
Potri.014Chr14	6983024	6983371	348	*	194	154	4
Potri.014Chr14	7117836	7118097	262	*	315	119	6
Potri.014Chr14	7291885	7292103	219	*	178	86	4
Potri.014Chr14	7987068	7987324	257	*	171	160	3
Potri.014Chr14	8147135	8147397	263	*	183	128	3
Potri.014Chr14	8232527	8232800	274	*	203	138	4
Potri.014Chr14	9201094	9201411	318	*	195	137	4
Potri.014Chr14	9345135	9345353	219	*	308	123	3
Potri.014Chr14	9716376	9716605	230	*	209	115	5
Potri.014Chr14	10022432	10022739	308	*	168	147	3
Potri.014Chr14	10405941	10406300	360	*	428	201	5
Potri.014Chr14	10418607	10418843	237	*	187	99	4
Potri.014Chr14	10635097	10635313	217	*	181	112	4
Potri.014Chr14	11046105	11046353	249	*	196	158	4
Potri.014Chr14	11077199	11077427	229	*	407	130	3
Potri.014Chr14	11132234	11132610	377	*	205	175	4
Potri.014Chr14	11503902	11504202	301	*	178	134	3
Potri.014Chr14	12212279	12212490	212	*	169	98	5
Potri.014Chr14	12397126	12397396	271	*	269	130	5
Potri.014Chr14	12601509	12601777	269	*	227	149	4
Potri.014Chr14	12728202	12728494	293	*	168	176	3
Potri.014Chr14	12946298	12946536	239	*	244	109	5
Potri.014Chr14	13145245	13145569	325	*	189	173	4
Potri.014Chr14	13563493	13563800	308	*	201	146	3
Potri.014Chr14	13668119	13668437	319	*	487	157	5
Potri.014Chr14	14101896	14102116	221	*	188	96	5
Potri.014Chr14	14566009	14566226	218	*	245	113	4
Potri.014Chr14	14599724	14600011	288	*	258	161	4
Potri.014Chr14	14775846	14776143	298	*	175	152	4
Potri.014Chr14	14837207	14837452	246	*	175	134	5
Potri.014Chr14	15179903	15180132	230	*	192	128	5
Potri.014Chr14	15933493	15933727	235	*	560	130	3
Potri.014Chr14	16521024	16521270	247	*	383	102	2
Potri.014Chr14	16649522	16649724	203	*	171	70	3
Potri.014Chr14	16978898	16980182	1285	*	179	976	2
Potri.014Chr14	16985454	16986796	1343	*	171	459	2
Potri.014Chr14	17215397	17215628	232	*	405	113	3
Potri.014Chr14	17260715	17261251	537	*	197	378	4
Potri.014Chr14	17362526	17363026	501	*	183	127	4

Potri.014	Chr14	17500991	17501207	217	*	169	89	5
Potri.014	Chr14	17619992	17620248	257	*	230	127	5
Potri.014	Chr14	17648528	17648836	309	*	209	134	5
Potri.014	Chr14	17822783	17822993	211	*	291	95	5
Potri.014	Chr14	17879127	17879399	273	*	324	150	6
Potri.014	Chr14	17942805	17943028	224	*	181	118	4
Potri.014	Chr14	18135696	18135898	203	*	250	106	3
Potri.014	Chr14	18333017	18333238	222	*	357	119	4
Potri.014	Chr14	18419621	18419980	360	*	175	226	3
Potri.015	Chr15	461873	462108	236	*	181	95	4
Potri.015	Chr15	602061	602617	557	*	176	119	3
Potri.015	Chr15	941383	941595	213	*	177	128	4
Potri.015	Chr15	1854564	1854801	238	*	272	88	4
Potri.015	Chr15	1899690	1899953	264	*	179	150	3
Potri.015	Chr15	2345890	2346330	441	*	186	155	3
Potri.015	Chr15	2635540	2635772	233	*	180	104	4
Potri.015	Chr15	2937669	2937945	277	*	193	87	3
Potri.015	Chr15	3344859	3345118	260	*	174	116	3
Potri.015	Chr15	3489693	3489965	273	*	170	155	4
Potri.015	Chr15	3500748	3501045	298	*	192	163	4
Potri.015	Chr15	3694113	3694320	208	*	186	100	3
Potri.015	Chr15	3731711	3731941	231	*	178	74	4
Potri.015	Chr15	4009026	4009277	252	*	233	123	4
Potri.015	Chr15	4087629	4087945	317	*	175	194	3
Potri.015	Chr15	4098981	4099336	356	*	229	190	3
Potri.015	Chr15	4109875	4110361	487	*	304	369	6
Potri.015	Chr15	4401989	4402233	245	*	180	99	3
Potri.015	Chr15	4558037	4558245	209	*	311	90	2
Potri.015	Chr15	4967890	4968263	374	*	652	171	6
Potri.015	Chr15	5161556	5161836	281	*	386	140	6
Potri.015	Chr15	5411884	5412132	249	*	228	114	4
Potri.015	Chr15	5584983	5585207	225	*	191	121	5
Potri.015	Chr15	5662904	5663111	208	*	168	115	4
Potri.015	Chr15	6289629	6289839	211	*	267	104	3
Potri.015	Chr15	6322481	6322828	348	*	509	161	3
Potri.015	Chr15	6326514	6326745	232	*	628	123	3
Potri.015	Chr15	6409201	6409415	215	*	288	93	3
Potri.015	Chr15	6588669	6589028	360	*	185	190	3
Potri.015	Chr15	6571580	6571788	209	*	262	117	3
Potri.015	Chr15	6682178	6682397	220	*	172	123	3
Potri.015	Chr15	6969549	6969757	209	*	226	82	4
Potri.015	Chr15	7444315	7444539	225	*	168	113	5
Potri.015	Chr15	7978405	7978679	275	*	521	141	3
Potri.015	Chr15	8041979	8042479	501	*	232	107	3
Potri.015	Chr15	8388319	8388556	238	*	201	128	4
Potri.015	Chr15	8583408	8583620	213	*	193	91	4
Potri.015	Chr15	8887117	8887348	232	*	310	135	4
Potri.015	Chr15	9031032	9031331	300	*	207	154	4
Potri.015	Chr15	9289141	9289386	246	*	182	109	4
Potri.015	Chr15	9574403	9574642	240	*	251	121	5
Potri.015	Chr15	10235085	10235307	223	*	503	123	3
Potri.015	Chr15	11321131	11321356	226	*	173	146	4
Potri.015	Chr15	11995780	11996031	252	*	170	129	4
Potri.015	Chr15	12014481	12014793	313	*	181	148	4

Potri.015	Chr15	12110429	12110659	231	*	175	140	2
Potri.015	Chr15	12313711	12313968	258	*	210	141	5
Potri.015	Chr15	13068899	13069142	244	*	306	141	6
Potri.015	Chr15	13293696	13293938	243	*	187	160	3
Potri.015	Chr15	13307371	13307642	272	*	336	150	5
Potri.015	Chr15	13331956	13332356	401	*	202	249	3
Potri.015	Chr15	13653651	13653894	244	*	215	116	3
Potri.015	Chr15	13712456	13712705	250	*	399	129	5
Potri.015	Chr15	13741175	13741425	251	*	263	137	4
Potri.015	Chr15	14300750	14301043	294	*	258	148	4
Potri.015	Chr15	14918229	14918486	258	*	192	139	3
Potri.015	Chr15	14977099	14977362	264	*	250	105	4
Potri.015	Chr15	15241519	15241885	367	*	179	192	3
Potri.016	Chr16	530065	530375	311	*	185	150	3
Potri.016	Chr16	560815	561044	230	*	276	138	3
Potri.016	Chr16	625084	625538	455	*	254	335	5
Potri.016	Chr16	817876	818089	214	*	249	138	3
Potri.016	Chr16	905345	905600	256	*	237	96	3
Potri.016	Chr16	1443761	1443995	235	*	185	146	3
Potri.016	Chr16	1446034	1446240	207	*	176	74	3
Potri.016	Chr16	1626487	1626919	433	*	225	251	3
Potri.016	Chr16	1867470	1867723	254	*	214	155	4
Potri.016	Chr16	2510795	2511102	308	*	181	129	4
Potri.016	Chr16	2885831	2886045	215	*	172	101	3
Potri.016	Chr16	2906127	2906362	236	*	209	113	5
Potri.016	Chr16	3015631	3015863	233	*	175	116	5
Potri.016	Chr16	3200780	3201012	233	*	186	111	3
Potri.016	Chr16	3327866	3328462	597	*	168	118	3
Potri.016	Chr16	3406078	3406383	306	*	264	162	5
Potri.016	Chr16	3542430	3542665	236	*	196	93	4
Potri.016	Chr16	3618240	3618617	378	*	276	134	3
Potri.016	Chr16	3796009	3796626	618	*	176	310	3
Potri.016	Chr16	3872848	3873270	423	*	193	173	3
Potri.016	Chr16	3936618	3937099	482	*	203	376	4
Potri.016	Chr16	4119864	4120176	313	*	183	170	3
Potri.016	Chr16	4267058	4267301	244	*	241	117	3
Potri.016	Chr16	4315585	4315827	243	*	253	108	3
Potri.016	Chr16	4391481	4391751	271	*	192	152	4
Potri.016	Chr16	4948225	4948497	273	*	269	124	5
Potri.016	Chr16	5040280	5040498	219	*	169	121	5
Potri.016	Chr16	5526856	5527169	314	*	191	172	3
Potri.016	Chr16	5843554	5843822	269	*	329	151	6
Potri.016	Chr16	6063642	6064470	829	*	170	153	3
Potri.016	Chr16	6098891	6099288	398	*	392	172	4
Potri.016	Chr16	6579784	6580058	275	*	210	145	3
Potri.016	Chr16	6678744	6679083	340	*	179	200	3
Potri.016	Chr16	7275824	7276179	356	*	320	165	3
Potri.016	Chr16	7289451	7289729	279	*	203	133	4
Potri.016	Chr16	7689821	7690032	212	*	327	88	3
Potri.016	Chr16	7805504	7805767	264	*	186	152	4
Potri.016	Chr16	8010657	8010894	238	*	244	151	5
Potri.016	Chr16	8130685	8130911	227	*	214	114	3
Potri.016	Chr16	8218262	8218496	235	*	458	132	3
Potri.016	Chr16	8455733	8455966	234	*	259	118	5

Potri.016	Chr16	10368226	10368481	256	*	289	117	6
Potri.016	Chr16	10424885	10425228	344	*	185	141	3
Potri.016	Chr16	11061788	11061994	207	*	325	119	3
Potri.016	Chr16	11095519	11095738	220	*	246	109	3
Potri.016	Chr16	11214967	11215202	236	*	195	122	5
Potri.016	Chr16	12148167	12148407	241	*	204	150	3
Potri.016	Chr16	12349383	12350004	622	*	483	175	4
Potri.016	Chr16	12541018	12541219	202	*	215	85	3
Potri.016	Chr16	12602222	12602572	351	*	215	262	4
Potri.016	Chr16	13014275	13014476	202	*	196	96	3
Potri.016	Chr16	13270386	13270787	402	*	175	109	5
Potri.016	Chr16	13363981	13364208	228	*	380	130	3
Potri.016	Chr16	13763101	13763601	501	*	190	356	3
Potri.016	Chr16	13771339	13771632	294	*	192	148	4
Potri.016	Chr16	13842785	13843014	230	*	208	109	3
Potri.016	Chr16	13992626	13992871	246	*	170	104	4
Potri.016	Chr16	14175180	14175403	224	*	169	131	3
Potri.016	Chr16	14186333	14186653	321	*	185	142	3
Potri.017	Chr17	363315	363550	236	*	223	106	5
Potri.017	Chr17	473359	473579	221	*	175	127	5
Potri.017	Chr17	587365	587592	228	*	173	132	4
Potri.017	Chr17	697601	697802	202	*	218	87	2
Potri.017	Chr17	804357	804607	251	*	203	114	4
Potri.017	Chr17	947259	947479	221	*	175	117	4
Potri.017	Chr17	1821661	1822277	617	*	277	134	4
Potri.017	Chr17	2039379	2039849	471	*	198	156	3
Potri.017	Chr17	2166085	2166342	258	*	373	149	3
Potri.017	Chr17	2261594	2261979	386	*	188	242	3
Potri.017	Chr17	2355644	2355852	209	*	204	84	3
Potri.017	Chr17	2579352	2579643	292	*	185	138	3
Potri.017	Chr17	2665049	2665258	210	*	183	78	3
Potri.017	Chr17	3582131	3582363	233	*	244	130	5
Potri.017	Chr17	3596169	3596677	509	*	175	156	3
Potri.017	Chr17	3706748	3707072	325	*	259	143	5
Potri.017	Chr17	4048108	4048427	320	*	198	175	3
Potri.017	Chr17	4591715	4591934	220	*	215	73	3
Potri.017	Chr17	5654502	5654924	423	*	175	255	4
Potri.017	Chr17	5707856	5708331	476	*	226	353	4
Potri.017	Chr17	5868625	5868846	222	*	460	112	3
Potri.017	Chr17	5940914	5941147	234	*	582	118	3
Potri.017	Chr17	6939831	6940100	270	*	209	99	3
Potri.017	Chr17	7300015	7300245	231	*	874	128	5
Potri.017	Chr17	7358823	7359070	248	*	541	121	3
Potri.017	Chr17	7510308	7510541	234	*	362	141	4
Potri.017	Chr17	8293044	8293296	253	*	237	138	4
Potri.017	Chr17	8769072	8769288	217	*	343	123	3
Potri.017	Chr17	9597791	9598003	213	*	171	105	3
Potri.017	Chr17	9634484	9634763	280	*	179	201	4
Potri.017	Chr17	9812455	9812689	235	*	229	124	5
Potri.017	Chr17	10055068	10055295	228	*	550	94	3
Potri.017	Chr17	10335525	10335760	236	*	175	125	5
Potri.017	Chr17	10846559	10846767	209	*	171	84	4
Potri.017	Chr17	10937777	10937996	220	*	216	129	4
Potri.017	Chr17	11031114	11031464	351	*	261	210	5

Potri.017	Chr17	11508006	11508328	323	*	198	156	3
Potri.017	Chr17	11651802	11652103	302	*	236	125	4
Potri.017	Chr17	11720425	11720638	214	*	184	97	4
Potri.017	Chr17	11995502	11995733	232	*	178	106	3
Potri.017	Chr17	12180323	12180530	208	*	215	93	4
Potri.017	Chr17	12571528	12571742	215	*	170	90	4
Potri.017	Chr17	12622159	12622473	315	*	178	143	4
Potri.017	Chr17	12856876	12857104	229	*	188	124	5
Potri.017	Chr17	13004792	13005061	270	*	208	157	3
Potri.017	Chr17	13146131	13146415	285	*	248	134	4
Potri.017	Chr17	13184785	13184991	207	*	181	91	4
Potri.017	Chr17	13334335	13334709	375	*	179	206	3
Potri.017	Chr17	13533660	13533872	213	*	303	92	3
Potri.017	Chr17	14201299	14201507	209	*	185	121	4
Potri.017	Chr17	14231431	14231738	308	*	171	138	3
Potri.017	Chr17	14240930	14241156	227	*	170	127	3
Potri.017	Chr17	14446380	14446598	219	*	189	98	3
Potri.017	Chr17	14673370	14673631	262	*	188	142	5
Potri.017	Chr17	14723996	14724203	208	*	170	97	4
Potri.017	Chr17	14743710	14743957	248	*	206	104	4
Potri.017	Chr17	14752893	14753162	270	*	174	181	3
Potri.017	Chr17	14836792	14837023	232	*	491	95	3
Potri.017	Chr17	14837449	14837653	205	*	314	92	3
Potri.017	Chr17	15101487	15102270	784	*	212	225	2
Potri.017	Chr17	15518775	15519123	349	*	173	154	3
Potri.017	Chr17	15809444	15809659	216	*	178	103	4
Potri.017	Chr17	15920269	15921049	781	*	196	340	3
Potri.018	Chr18	772358	772608	251	*	170	156	3
Potri.018	Chr18	839474	839693	220	*	175	110	5
Potri.018	Chr18	1278807	1279016	210	*	179	107	3
Potri.018	Chr18	1499901	1500118	218	*	193	91	5
Potri.018	Chr18	1748579	1748897	319	*	189	180	4
Potri.018	Chr18	2322662	2322924	263	*	175	90	3
Potri.018	Chr18	2343894	2344167	274	*	205	156	4
Potri.018	Chr18	2355474	2355775	302	*	233	174	4
Potri.018	Chr18	2437335	2437686	352	*	317	176	2
Potri.018	Chr18	2684266	2684518	253	*	181	158	4
Potri.018	Chr18	3014239	3014597	359	*	175	220	4
Potri.018	Chr18	3107667	3107892	226	*	530	127	3
Potri.018	Chr18	3229837	3230082	246	*	352	121	6
Potri.018	Chr18	3799429	3799687	259	*	924	121	3
Potri.018	Chr18	4016125	4016365	241	*	195	144	5
Potri.018	Chr18	4061342	4061794	453	*	221	183	2
Potri.018	Chr18	4112354	4112573	220	*	225	116	4
Potri.018	Chr18	4160529	4160751	223	*	297	119	4
Potri.018	Chr18	4435302	4435649	348	*	215	156	4
Potri.018	Chr18	4690898	4691105	208	*	239	139	3
Potri.018	Chr18	5031688	5031913	226	*	349	95	3
Potri.018	Chr18	5242377	5242612	236	*	898	120	4
Potri.018	Chr18	5636568	5636770	203	*	264	97	3
Potri.018	Chr18	5770252	5770719	468	*	211	338	4
Potri.018	Chr18	6207695	6207942	248	*	182	162	4
Potri.018	Chr18	6289530	6289746	217	*	181	117	4
Potri.018	Chr18	6400348	6400583	236	*	177	146	4

Potri.018	Chr18	6617839	6618084	246	*	704	135	6
Potri.018	Chr18	7356032	7356235	204	*	184	97	2
Potri.018	Chr18	7768175	7768466	292	*	189	120	2
Potri.018	Chr18	8130695	8130952	258	*	181	137	4
Potri.018	Chr18	9329858	9330060	203	*	366	109	3
Potri.018	Chr18	10091057	10091491	435	*	174	309	3
Potri.018	Chr18	10158223	10158437	215	*	175	115	4
Potri.018	Chr18	10521460	10521856	397	*	199	246	3
Potri.018	Chr18	10734364	10734723	360	*	186	227	3
Potri.018	Chr18	11169234	11169541	308	*	204	164	3
Potri.018	Chr18	11640501	11640729	229	*	269	130	4
Potri.018	Chr18	11706478	11706743	266	*	573	138	6
Potri.018	Chr18	11812258	11812488	231	*	240	126	4
Potri.018	Chr18	12559350	12559659	310	*	220	158	4
Potri.018	Chr18	12642901	12643260	360	*	180	170	3
Potri.018	Chr18	12797411	12797679	269	*	223	124	4
Potri.018	Chr18	13033689	13033899	211	*	188	114	5
Potri.018	Chr18	13215049	13215268	220	*	201	123	4
Potri.018	Chr18	13662298	13662651	354	*	187	185	4
Potri.018	Chr18	13777968	13778222	255	*	274	125	3
Potri.018	Chr18	13916082	13916664	583	*	193	422	3
Potri.018	Chr18	15181502	15181883	382	*	182	251	4
Potri.018	Chr18	15517355	15517558	204	*	188	122	5
Potri.018	Chr18	15591553	15591759	207	*	216	140	3
Potri.018	Chr18	15620341	15620542	202	*	211	101	3
Potri.018	Chr18	15739345	15739603	259	*	192	141	4
Potri.018	Chr18	15995787	15996100	314	*	263	151	4
Potri.018	Chr18	16893449	16893702	254	*	350	135	5
Potri.019	Chr19	160656	160927	272	*	323	133	5
Potri.019	Chr19	286217	286607	391	*	214	269	3
Potri.019	Chr19	457307	457518	212	*	214	92	4
Potri.019	Chr19	560449	560660	212	*	189	102	4
Potri.019	Chr19	1380794	1381007	214	*	177	111	4
Potri.019	Chr19	1431883	1432455	573	*	194	430	4
Potri.019	Chr19	1487580	1487814	235	*	188	108	5
Potri.019	Chr19	1926344	1926562	219	*	218	124	4
Potri.019	Chr19	1984778	1985045	268	*	196	106	4
Potri.019	Chr19	1998582	1998824	243	*	266	133	5
Potri.019	Chr19	2143248	2143463	216	*	253	65	3
Potri.019	Chr19	2175572	2175811	240	*	179	95	4
Potri.019	Chr19	2310559	2310857	299	*	168	113	3
Potri.019	Chr19	2425264	2425873	610	*	271	145	3
Potri.019	Chr19	2704860	2705414	555	*	182	173	3
Potri.019	Chr19	2751653	2751916	264	*	320	120	3
Potri.019	Chr19	3188226	3188439	214	*	228	59	3
Potri.019	Chr19	3192907	3193128	222	*	642	109	4
Potri.019	Chr19	3194890	3195647	758	*	292	389	2
Potri.019	Chr19	3202901	3203470	570	*	923	129	3
Potri.019	Chr19	3204251	3204503	253	*	629	118	3
Potri.019	Chr19	3347902	3348239	338	*	210	177	3
Potri.019	Chr19	3790649	3790908	260	*	316	138	6
Potri.019	Chr19	3831881	3832177	297	*	219	136	3
Potri.019	Chr19	3870187	3870464	278	*	258	143	4
Potri.019	Chr19	4765413	4765637	225	*	197	151	4

Potri.019Chr19	4775656	4775884	229 *		406	101	3
Potri.019Chr19	4984821	4985392	572 *		172	428	3
Potri.019Chr19	5166187	5166408	222 *		175	115	5
Potri.019Chr19	5185644	5185882	239 *		247	146	4
Potri.019Chr19	5215445	5215649	205 *		281	110	3
Potri.019Chr19	5248776	5249018	243 *		191	104	3
Potri.019Chr19	5320875	5321121	247 *		808	139	3
Potri.019Chr19	5545993	5546563	571 *		291	434	2
Potri.019Chr19	5559122	5559340	219 *		247	106	3
Potri.019Chr19	6691063	6691619	557 *		404	92	3
Potri.019Chr19	6933162	6933389	228 *		182	139	3
Potri.019Chr19	6959709	6959952	244 *		218	119	4
Potri.019Chr19	7262737	7262938	202 *		181	128	2
Potri.019Chr19	7535840	7536112	273 *		346	149	6
Potri.019Chr19	7814338	7814648	311 *		294	156	6
Potri.019Chr19	7832460	7832781	322 *		785	174	3
Potri.019Chr19	7834493	7834749	257 *		509	120	3
Potri.019Chr19	9847551	9847805	255 *		238	150	4
Potri.019Chr19	9943058	9943266	209 *		261	107	3
Potri.019Chr19	9986720	9987027	308 *		309	151	2
Potri.019Chr19	10011824	10012061	238 *		183	162	4
Potri.019Chr19	10242307	10242642	336 *		186	125	3
Potri.019Chr19	10496412	10496648	237 *		188	120	5
Potri.019Chr19	10681745	10682003	259 *		176	107	3
Potri.019Chr19	11359705	11359914	210 *		173	107	4
Potri.019Chr19	11772245	11772480	236 *		169	146	5
Potri.019Chr19	11801277	11801588	312 *		281	145	5
Potri.019Chr19	12251353	12251619	267 *		187	138	4
Potri.019Chr19	12260884	12261174	291 *		171	113	3
Potri.019Chr19	12331612	12331842	231 *		271	124	4
Potri.019Chr19	12958554	12958796	243 *		210	127	5
Potri.019Chr19	13002509	13002946	438 *		191	319	4
Potri.019Chr19	13062550	13062795	246 *		304	108	5
Potri.019Chr19	13316679	13316924	246 *		203	87	4
Potri.019Chr19	13374682	13374888	207 *		197	90	3
Potri.019Chr19	13567662	13567880	219 *		179	121	3
Potri.019Chr19	13674062	13674281	220 *		192	105	4
Potri.019Chr19	14453979	14454208	230 *		175	117	4
Potri.019Chr19	14853067	14853277	211 *		259	65	4
Potri.019Chr19	15187623	15187921	299 *		192	156	4
Potri.019Chr19	15371466	15371738	273 *		195	142	3
Potri.019Chr19	15897031	15897496	466 *		223	145	5
Potri.T00scaffold_	140563	140786	224 *		252	99	4
Potri.T00scaffold_	235955	236178	224 *		580	121	3
Potri.T00scaffold_	529210	529440	231 *		255	136	4
Potri.T00scaffold_	339895	340230	336 *		777	153	3
Potri.T00scaffold_	437154	437696	543 *		446	410	3
Potri.T00scaffold_	438879	439424	546 *		313	141	2
Potri.T00scaffold_	697168	697374	207 *		330	105	3
Potri.T00scaffold_	746250	746665	416 *		271	218	2
Potri.T01scaffold_	101172	101701	530 *		272	430	3
Potri.T01scaffold_	120325	120527	203 *		195	108	3
Potri.T01scaffold_	381765	381981	217 *		232	134	4
Potri.T01scaffold_	396446	396714	269 *		224	149	3

Potri.T01scaffold_	536084	536377	294	*	177	156	4
Potri.T01scaffold_	466595	466834	240	*	374	141	3
Potri.T01scaffold_	192813	193031	219	*	398	96	4
Potri.T02scaffold_	226718	227001	284	*	575	149	3
Potri.T02scaffold_	127749	127967	219	*	198	85	4
Potri.T02scaffold_	174244	174485	242	*	188	105	5
Potri.T02scaffold_	341603	341811	209	*	216	114	3
Potri.T02scaffold_	366468	366705	238	*	637	109	3
Potri.T02scaffold_	97826	98042	217	*	281	94	4
Potri.T03scaffold_	30325	30543	219	*	169	78	5
Potri.T03scaffold_	229679	229892	214	*	317	154	4
Potri.T03scaffold_	251446	251746	301	*	244	158	5
Potri.T03scaffold_	247062	247562	501	*	383	116	3
Potri.T03scaffold_	263597	263824	228	*	360	108	4
Potri.T03scaffold_	272244	272483	240	*	473	112	3
Potri.T04scaffold_	109281	109492	212	*	313	120	3
Potri.T04scaffold_	153530	153744	215	*	247	121	3
Potri.T04scaffold_	91807	92077	271	*	368	147	7
Potri.T04scaffold_	191964	192239	276	*	196	140	4
Potri.T04scaffold_	51381	51608	228	*	401	106	3
Potri.T04scaffold_	163693	163961	269	*	183	183	2
Potri.T04scaffold_	196942	197161	220	*	175	136	5
Potri.T04scaffold_	277166	277404	239	*	209	141	5
Potri.T04scaffold_	20466	20710	245	*	188	179	5
Potri.T05scaffold_	187580	187803	224	*	312	103	4
Potri.T05scaffold_	131452	131653	202	*	172	110	3
Potri.T05scaffold_	203202	203458	257	*	192	146	4
Potri.T05scaffold_	68538	68839	302	*	296	132	2
Potri.T05scaffold_	183397	183607	211	*	293	113	3
Potri.T05scaffold_	110637	110860	224	*	633	135	3
Potri.T06scaffold_	106148	106509	362	*	203	198	4
Potri.T06scaffold_	112672	112987	316	*	176	168	3
Potri.T06scaffold_	116742	116996	255	*	589	125	3
Potri.T07scaffold_	11224	11477	254	*	215	137	3
Potri.T07scaffold_	71376	71636	261	*	467	143	2
Potri.T07scaffold_	124652	124857	206	*	186	131	3
Potri.T08scaffold_	100694	100965	272	*	220	168	4
Potri.T08scaffold_	96266	96549	284	*	443	146	3
Potri.T08scaffold_	16883	17093	211	*	185	97	3
Potri.T08scaffold_	97582	97947	366	*	176	188	3
Potri.T09scaffold_	60594	60804	211	*	329	108	3
Potri.T09scaffold_	2089	2308	220	*	176	134	4
Potri.T09scaffold_	16648	16862	215	*	172	127	4
Potri.T09scaffold_	58552	58835	284	*	378	165	3
Potri.T09scaffold_	67004	67373	370	*	177	259	2
Potri.T10scaffold_	15545	17317	1773	*	198	311	2
Potri.T10scaffold_	23429	23762	334	*	338	177	2
Potri.T10scaffold_	61178	61730	553	*	756	130	3
Potri.T10scaffold_	54053	54260	208	*	281	102	3
Potri.T10scaffold_	85032	85318	287	*	210	151	5
Potri.T10scaffold_	8098	8322	225	*	427	122	3
Potri.T10scaffold_	8036	8282	247	*	205	94	3
Potri.T10scaffold_	15185	15418	234	*	208	116	5
Potri.T11scaffold_	37809	38018	210	*	221	101	3

Potri.T11scaffold_	26116	26350	235	*	195	126	5
Potri.T11scaffold_	70116	70351	236	*	410	100	5
Potri.T11scaffold_	21341	21569	229	*	341	109	4
Potri.T11scaffold_	30570	30802	233	*	233	120	5
Potri.T11scaffold_	48324	48548	225	*	193	126	4
Potri.T12scaffold_	13379	13646	268	*	294	150	5
Potri.T12scaffold_	54639	54844	206	*	176	64	3
Potri.T13scaffold_	22836	23061	226	*	243	100	3
Potri.T13scaffold_	39844	40048	205	*	186	83	3
Potri.T14scaffold_	37592	37802	211	*	258	105	4
Potri.T14scaffold_	6513	6729	217	*	190	79	3
Potri.T14scaffold_	14708	14914	207	*	195	117	5
Potri.T15scaffold_	3858	4117	260	*	252	143	3
Potri.T16scaffold_	12743	13173	431	*	196	193	3
Potri.T16scaffold_	12154	12726	573	*	221	267	2
Potri.T17scaffold_	3245	3462	218	*	170	77	4
Potri.T17scaffold_	9712	9950	239	*	183	123	4
Potri.T17scaffold_	31495	31728	234	*	380	96	3
Potri.T17scaffold_	18985	19243	259	*	205	91	4
Potri.T17scaffold_	1678	1928	251	*	208	104	4
Potri.T17scaffold_	1992	2197	206	*	250	66	3

thick.end	thick.wid	itemRgb	peak	start_pos	end_posit	feature_s	insideFea	distancet
21	17	NA	LBD21.31R	931891	937283	+	upstream	-2746
17	16	NA	LBD21.31R	1858218	1858899	-	downstrea	4301
19	16	NA	LBD21.31R	2419636	2425132	-	overlapSt	70
19	16	NA	LBD21.31R	2563817	2582977	+	upstream	-2658
26	23	NA	LBD21.31R	3608830	3616405	+	downstrea	11185
21	19	NA	LBD21.31R	3640591	3642167	+	upstream	-14969
17	14	NA	LBD21.31R	3655019	3662744	+	inside	2877
18	16	NA	LBD21.31R	3866711	3875990	+	inside	1031
44	42	NA	LBD21.31R	4424461	4425107	-	inside	540
17	14	NA	LBD21.31R	4471426	4472137	-	downstrea	4969
17	14	NA	LBD21.31R	4726501	4726914	+	downstrea	599
20	17	NA	LBD21.31R	4738951	4744301	+	upstream	-499
17	15	NA	LBD21.31R	4817225	4820458	+	inside	214
57	54	NA	LBD21.31R	5081114	5083400	-	upstream	-234
17	14	NA	LBD21.31R	5163758	5164215	+	downstrea	487
21	17	NA	LBD21.31R	5525312	5526193	+	upstream	-6925
19	16	NA	LBD21.31R	5528912	5531093	+	downstrea	3415
19	17	NA	LBD21.31R	5594633	5596573	+	upstream	-289
17	15	NA	LBD21.31R	5695547	5701591	-	inside	270
17	14	NA	LBD21.31R	5712602	5713239	-	overlapSt	183
24	22	NA	LBD21.31R	6537780	6548390	+	inside	650
18	15	NA	LBD21.31R	6608926	6610764	-	upstream	-739
23	20	NA	LBD21.31R	6817441	6820954	+	inside	2768
24	20	NA	LBD21.31R	7116809	7118715	+	inside	1357
19	17	NA	LBD21.31R	7161567	7161995	-	upstream	-431
19	16	NA	LBD21.31R	7162074	7164172	-	upstream	-986
19	16	NA	LBD21.31R	7201940	7204932	+	inside	993
20	18	NA	LBD21.31R	8136078	8139864	+	upstream	-448
20	18	NA	LBD21.31R	8144938	8152505	+	inside	3389
16	12	NA	LBD21.31R	8563785	8564939	-	upstream	-277
19	16	NA	LBD21.31R	9208104	9209012	-	upstream	-2680
17	14	NA	LBD21.31R	9228514	9229965	-	downstrea	8870
31	27	NA	LBD21.31R	9497158	9500671	+	inside	1850
21	18	NA	LBD21.31R	9532286	9534455	-	downstrea	3091
17	15	NA	LBD21.31R	9856787	9866426	+	upstream	-607
22	20	NA	LBD21.31R	9962114	9963181	+	downstrea	3357
17	13	NA	LBD21.31R	10705848	10712357	-	inside	3201
18	16	NA	LBD21.31R	11013679	11014410	-	overlapSt	62
23	20	NA	LBD21.31R	11300605	11302211	-	upstream	-2025
36	35	NA	LBD21.31R	11492791	11497064	+	upstream	-1174
20	19	NA	LBD21.31R	11542659	11544254	-	upstream	-19426
18	16	NA	LBD21.31R	12170721	12173268	+	downstrea	9948
18	15	NA	LBD21.31R	12721833	12723526	+	upstream	-1537
28	25	NA	LBD21.31R	13280030	13281057	+	downstrea	2347
20	17	NA	LBD21.31R	13556117	13556365	+	downstrea	902
19	17	NA	LBD21.31R	13976442	13981566	-	inside	1035
22	18	NA	LBD21.31R	14218530	14221206	-	upstream	-7148
21	19	NA	LBD21.31R	14797301	14801934	+	inside	3201
20	18	NA	LBD21.31R	14992433	14993839	+	downstrea	1959
34	30	NA	LBD21.31R	15234652	15237125	-	inside	2391
16	12	NA	LBD21.31R	15542075	15545257	-	upstream	-3673

17	14	NA	LBD21.31R	15639966	15640319	-	downstrea	2587
20	16	NA	LBD21.31R	15650879	15654047	-	upstream	-304
31	29	NA	LBD21.31R	16015850	16019299	+	upstream	-4277
16	14	NA	LBD21.31R	16053844	16057783	-	inside	1408
19	17	NA	LBD21.31R	16178623	16183438	-	inside	1638
18	16	NA	LBD21.31R	16389360	16390346	+	downstrea	2299
16	14	NA	LBD21.31R	17034511	17041769	+	inside	2781
18	17	NA	LBD21.31R	17234276	17239905	-	inside	4293
41	40	NA	LBD21.31R	17650350	17656084	-	downstrea	6780
18	15	NA	LBD21.31R	17698397	17700840	+	upstream	-2820
19	17	NA	LBD21.31R	18382069	18382905	+	downstrea	90189
27	25	NA	LBD21.31R	18767933	18772523	+	downstrea	19821
21	17	NA	LBD21.31R	18808260	18809389	-	upstream	-29226
16	12	NA	LBD21.31R	19160680	19162246	-	upstream	-4316
20	17	NA	LBD21.31R	19204737	19210160	-	inside	2439
37	35	NA	LBD21.31R	19240538	19240907	+	upstream	-8100
20	18	NA	LBD21.31R	19474731	19479610	-	inside	2680
29	27	NA	LBD21.31R	19852151	19852980	+	overlapSt	-35
24	22	NA	LBD21.31R	20658471	20661501	+	upstream	-770
25	22	NA	LBD21.31R	20750806	20752266	+	upstream	-3060
23	19	NA	LBD21.31R	20856453	20862070	-	upstream	-1541
25	21	NA	LBD21.31R	20933078	20936228	+	overlapSt	-87
34	31	NA	LBD21.31R	20943752	20958263	-	inside	8570
22	20	NA	LBD21.31R	21096101	21098276	-	downstrea	3354
23	20	NA	LBD21.31R	21210427	21214178	-	downstrea	6629
27	25	NA	LBD21.31R	21262213	21263432	-	upstream	-22035
26	22	NA	LBD21.31R	21731709	21738117	+	inside	3919
20	18	NA	LBD21.31R	21764257	21776257	-	inside	8616
28	25	NA	LBD21.31R	21798119	21805355	+	inside	2068
20	18	NA	LBD21.31R	22029049	22033651	-	inside	4461
23	20	NA	LBD21.31R	22363394	22367792	-	inside	681
18	16	NA	LBD21.31R	22652944	22656415	-	inside	1852
23	20	NA	LBD21.31R	22842447	22843911	-	overlapEn	1465
16	14	NA	LBD21.31R	23047696	23054875	-	inside	1409
24	20	NA	LBD21.31R	24556970	24562120	-	downstrea	7432
35	32	NA	LBD21.31R	25022582	25027843	+	inside	151
21	18	NA	LBD21.31R	25214577	25214992	-	upstream	-335
29	25	NA	LBD21.31R	25372661	25378972	-	inside	2325
22	19	NA	LBD21.31R	25408006	25410298	-	upstream	-199
24	20	NA	LBD21.31R	26016050	26017786	-	upstream	-5132
53	51	NA	LBD21.31R	26610644	26613598	-	inside	669
17	15	NA	LBD21.31R	26778088	26781152	-	upstream	-2849
20	18	NA	LBD21.31R	26837127	26838989	+	overlapSt	-114
22	19	NA	LBD21.31R	27283889	27284421	+	downstrea	3242
34	30	NA	LBD21.31R	27813645	27816590	-	upstream	-5579
20	17	NA	LBD21.31R	28008504	28009538	+	downstrea	6130
44	42	NA	LBD21.31R	28195252	28200532	-	upstream	-2392
20	18	NA	LBD21.31R	28405728	28407043	-	inside	696
17	15	NA	LBD21.31R	28524787	28536154	+	inside	2079
19	16	NA	LBD21.31R	28644983	28646106	+	downstrea	2310
19	16	NA	LBD21.31R	28656046	28658388	-	upstream	-601
17	13	NA	LBD21.31R	28983611	28985938	-	inside	1156
29	28	NA	LBD21.31R	29390781	29391222	-	inside	405
22	18	NA	LBD21.31R	29748443	29750426	-	downstrea	7544

20	18	NA	LBD21.31R	31452614	31455086	+	upstream	-3823
17	15	NA	LBD21.31R	32044646	32052144	-	inside	3411
34	32	NA	LBD21.31R	33457530	33457793	-	overlapSt	201
18	16	NA	LBD21.31R	33568408	33569446	-	upstream	-1135
21	18	NA	LBD21.31R	33830169	33836654	+	upstream	-1601
21	19	NA	LBD21.31R	33904534	33907921	-	upstream	-8033
17	15	NA	LBD21.31R	33974233	33976599	-	inside	494
22	19	NA	LBD21.31R	34176754	34177827	-	inside	981
20	17	NA	LBD21.31R	34316929	34319074	+	upstream	-392
19	16	NA	LBD21.31R	35235297	35238324	-	downstrea	5682
17	15	NA	LBD21.31R	35577723	35589740	+	inside	927
20	17	NA	LBD21.31R	35908608	35909747	-	upstream	-13637
19	17	NA	LBD21.31R	36110950	36111162	+	upstream	-1729
18	16	NA	LBD21.31R	36660993	36687274	+	inside	7059
18	16	NA	LBD21.31R	36703035	36719635	-	inside	370
17	15	NA	LBD21.31R	36770585	36775252	-	inside	1264
18	15	NA	LBD21.31R	37061055	37068329	-	upstream	-4688
21	19	NA	LBD21.31R	37165684	37172818	-	inside	2033
29	25	NA	LBD21.31R	37970473	37974990	-	overlapEn	4824
22	18	NA	LBD21.31R	38179930	38187645	-	upstream	-7750
20	17	NA	LBD21.31R	38536483	38543085	-	upstream	-3697
17	15	NA	LBD21.31R	38576579	38585411	+	inside	4441
30	25	NA	LBD21.31R	38900791	38901054	-	downstrea	783
17	14	NA	LBD21.31R	39459775	39461919	-	downstrea	20653
16	14	NA	LBD21.31R	39935036	39935322	+	upstream	-8125
17	15	NA	LBD21.31R	40072674	40074996	-	upstream	-11083
32	30	NA	LBD21.31R	40126837	40127766	-	downstrea	4693
17	13	NA	LBD21.31R	40937912	40940508	-	upstream	-3171
52	50	NA	LBD21.31R	41014240	41017948	-	inside	1999
17	14	NA	LBD21.31R	41340927	41349252	-	upstream	-3256
41	39	NA	LBD21.31R	41624735	41626633	+	downstrea	8901
23	19	NA	LBD21.31R	41808763	41810069	-	downstrea	4576
21	18	NA	LBD21.31R	42015598	42016722	-	upstream	-4637
21	19	NA	LBD21.31R	42080004	42085667	-	inside	2109
26	23	NA	LBD21.31R	42379094	42379882	-	upstream	-603
19	15	NA	LBD21.31R	42906326	42908305	+	upstream	-4708
27	24	NA	LBD21.31R	43033549	43041023	+	inside	3284
18	15	NA	LBD21.31R	43536705	43537358	-	downstrea	3615
18	15	NA	LBD21.31R	43699392	43703438	+	upstream	-5966
36	35	NA	LBD21.31R	43726632	43728235	-	inside	1555
20	18	NA	LBD21.31R	43782426	43783058	-	downstrea	4225
19	16	NA	LBD21.31R	43866229	43868094	+	upstream	-2579
18	15	NA	LBD21.31R	43906681	43908252	-	overlapSt	231
34	31	NA	LBD21.31R	44051744	44055305	-	inside	3268
20	17	NA	LBD21.31R	44498537	44503774	-	inside	3720
54	52	NA	LBD21.31R	45270331	45272211	-	upstream	-14455
16	14	NA	LBD21.31R	45654246	45654401	+	upstream	-10497
46	44	NA	LBD21.31R	45750195	45758236	-	downstrea	39277
24	22	NA	LBD21.31R	45800670	45801056	-	upstream	-3720
24	22	NA	LBD21.31R	46960726	46962464	+	downstrea	3595
16	12	NA	LBD21.31R	47055702	47057826	-	inside	2083
17	15	NA	LBD21.31R	47355398	47356346	-	upstream	-2148
29	26	NA	LBD21.31R	47420222	47423184	-	upstream	-4110
25	21	NA	LBD21.31R	47798128	47798564	+	downstrea	2888

30	27	NA	LBD21.31R	47869531	47873726	-		upstream	-3804
20	17	NA	LBD21.31R	48294396	48295061	-		downstrea	3697
30	26	NA	LBD21.31R	48744474	48747110	+		downstrea	4386
23	21	NA	LBD21.31R	48820335	48827240	+		inside	1361
17	15	NA	LBD21.31R	49081099	49084625	-		overlapSt	471
17	14	NA	LBD21.31R	49248574	49249820	+		downstrea	21292
17	14	NA	LBD21.31R	49433260	49437196	+		downstrea	7399
24	22	NA	LBD21.31R	49658499	49662020	+		upstream	-1248
19	17	NA	LBD21.31R	50301341	50304285	+		overlapSt	-447
18	16	NA	LBD21.31R	50307952	50312306	-		inside	2202
18	16	NA	LBD21.31R	50403482	50409278	-		inside	1547
20	18	NA	LBD21.31R	80364	85282	+		upstream	-3566
19	17	NA	LBD21.31R	240097	242866	-		inside	469
27	23	NA	LBD21.31R	673530	674147	-		inside	517
25	23	NA	LBD21.31R	1269779	1270145	-		downstrea	6128
21	19	NA	LBD21.31R	1762114	1764183	+		inside	118
19	16	NA	LBD21.31R	1813265	1814888	-		upstream	-9787
34	32	NA	LBD21.31R	1824453	1838295	-		inside	10252
18	15	NA	LBD21.31R	1947603	1948965	+		inside	492
18	16	NA	LBD21.31R	2325652	2333498	-		inside	5493
32	29	NA	LBD21.31R	2373460	2374844	+		upstream	-408
17	15	NA	LBD21.31R	2557989	2565187	-		inside	820
33	31	NA	LBD21.31R	2560092	2560412	+		inside	86
18	16	NA	LBD21.31R	2759390	2760947	-		upstream	-1041
67	63	NA	LBD21.31R	2804643	2807203	+		inside	233
19	16	NA	LBD21.31R	3157120	3158261	+		upstream	-7186
28	25	NA	LBD21.31R	3186386	3192465	-		inside	1003
26	23	NA	LBD21.31R	3253001	3261425	-		inside	1555
41	38	NA	LBD21.31R	3483467	3485264	+		inside	746
19	17	NA	LBD21.31R	3654028	3655628	-		downstrea	2025
18	16	NA	LBD21.31R	3665608	3675093	+		inside	3124
38	37	NA	LBD21.31R	3748149	3751459	-		overlapSt	265
20	17	NA	LBD21.31R	3782590	3787280	+		overlapSt	-45
28	26	NA	LBD21.31R	3848631	3851551	+		inside	271
16	14	NA	LBD21.31R	4202354	4203809	+		downstrea	1804
18	16	NA	LBD21.31R	4727613	4728448	-		downstrea	2344
17	14	NA	LBD21.31R	4816840	4817893	-		overlapSt	260
21	18	NA	LBD21.31R	4988728	4995830	-		upstream	-2007
22	19	NA	LBD21.31R	5058199	5066302	+		upstream	-326
17	16	NA	LBD21.31R	5242804	5253709	-		upstream	-2512
46	44	NA	LBD21.31R	5521471	5523733	-		upstream	-671
30	27	NA	LBD21.31R	5549316	5549963	+		inside	99
19	16	NA	LBD21.31R	5798611	5800222	-		upstream	-1143
25	22	NA	LBD21.31R	5971484	5974511	-		inside	857
17	15	NA	LBD21.31R	6026827	6029904	-		inside	1978
35	33	NA	LBD21.31R	6134192	6137820	-		upstream	-2246
17	13	NA	LBD21.31R	6300384	6304858	+		upstream	-4650
20	18	NA	LBD21.31R	6541365	6544162	-		inside	1932
17	15	NA	LBD21.31R	6758985	6761062	+		inside	1376
17	14	NA	LBD21.31R	6809734	6813364	+		upstream	-4631
24	21	NA	LBD21.31R	6817567	6820006	+		upstream	-3314
26	23	NA	LBD21.31R	6997442	6999188	-		upstream	-3418
19	16	NA	LBD21.31R	7140938	7145141	+		downstrea	4378
17	14	NA	LBD21.31R	7717689	7724144	-		upstream	-3100

17	15	NA	LBD21.31R	7816383	7817179	+	downstrea	6246
19	16	NA	LBD21.31R	7829783	7831956	+	downstrea	9181
21	18	NA	LBD21.31R	7974226	7975413	+	downstrea	1218
26	23	NA	LBD21.31R	8024624	8029932	+	inside	246
16	14	NA	LBD21.31R	8206493	8211920	-	inside	1356
20	18	NA	LBD21.31R	8322992	8326606	+	upstream	-733
16	14	NA	LBD21.31R	9176812	9180006	+	inside	181
17	14	NA	LBD21.31R	9529410	9530809	-	upstream	-1737
17	14	NA	LBD21.31R	9969304	9970138	+	upstream	-1287
19	18	NA	LBD21.31R	10233594	10242380	+	inside	4327
16	14	NA	LBD21.31R	10423223	10424588	-	upstream	-1019
19	16	NA	LBD21.31R	10483728	10484993	+	downstrea	1321
22	18	NA	LBD21.31R	10814710	10817717	-	downstrea	4535
18	15	NA	LBD21.31R	11637073	11641558	+	upstream	-4609
17	14	NA	LBD21.31R	11792508	11794803	+	upstream	-3116
18	15	NA	LBD21.31R	11909479	11910298	-	downstrea	1228
16	14	NA	LBD21.31R	11995106	11996200	+	upstream	-339
22	20	NA	LBD21.31R	12456399	12457606	+	downstrea	1909
17	15	NA	LBD21.31R	12532928	12540289	+	inside	667
18	15	NA	LBD21.31R	12796502	12798356	-	inside	1418
18	15	NA	LBD21.31R	13296225	13298788	+	downstrea	6469
21	18	NA	LBD21.31R	13618757	13622764	+	inside	3594
26	22	NA	LBD21.31R	14004276	14007348	+	upstream	-1025
46	44	NA	LBD21.31R	14263537	14263850	+	inside	31
72	70	NA	LBD21.31R	15622905	15627562	+	upstream	-4371
17	14	NA	LBD21.31R	15738767	15739814	-	inside	761
31	27	NA	LBD21.31R	15787688	15793426	+	upstream	-1389
19	17	NA	LBD21.31R	16766346	16767636	-	overlapEn	1395
22	19	NA	LBD21.31R	16834480	16838766	+	inside	2357
27	25	NA	LBD21.31R	17124896	17127337	+	inside	651
19	15	NA	LBD21.31R	17346654	17350721	-	upstream	-1671
73	71	NA	LBD21.31R	17656193	17656629	+	upstream	-3287
32	30	NA	LBD21.31R	17802211	17802676	+	downstrea	11558
17	15	NA	LBD21.31R	17887532	17887840	+	downstrea	936
23	20	NA	LBD21.31R	18233624	18234448	+	upstream	-24460
30	28	NA	LBD21.31R	18302824	18303082	+	inside	11
26	25	NA	LBD21.31R	18358100	18358803	-	downstrea	1998
22	21	NA	LBD21.31R	18361982	18362639	+	downstrea	1372
26	25	NA	LBD21.31R	18367804	18368815	+	inside	641
25	24	NA	LBD21.31R	18377658	18379352	-	overlapSt	1146
20	19	NA	LBD21.31R	18385207	18385666	+	overlapEn	82
27	26	NA	LBD21.31R	18387444	18388134	+	includeFe	-788
18	17	NA	LBD21.31R	18388647	18390665	+	inside	66
29	25	NA	LBD21.31R	19445909	19454497	-	downstrea	12091
17	14	NA	LBD21.31R	19561742	19564303	+	upstream	-11201
18	15	NA	LBD21.31R	19748763	19749182	-	upstream	-10687
18	16	NA	LBD21.31R	20471383	20474810	+	upstream	-505
32	29	NA	LBD21.31R	20561725	20563528	+	downstrea	7530
18	15	NA	LBD21.31R	20795929	20805938	-	inside	5929
19	16	NA	LBD21.31R	20918210	20919809	-	upstream	-118
30	26	NA	LBD21.31R	21167013	21172583	-	inside	567
18	16	NA	LBD21.31R	21519892	21525001	+	inside	2173
18	14	NA	LBD21.31R	21548048	21548636	-	downstrea	2280
17	14	NA	LBD21.31R	21819526	21822784	-	overlapEn	3259

27	23	NA	LBD21.31R	21932768	21941584	+	upstream	-7070
21	19	NA	LBD21.31R	22409931	22418405	+	inside	4150
24	21	NA	LBD21.31R	22420262	22425160	+	inside	2378
17	14	NA	LBD21.31R	22594429	22597522	+	inside	568
16	14	NA	LBD21.31R	22717022	22719775	-	inside	1676
25	23	NA	LBD21.31R	22760610	22768273	-	overlapSt	207
19	15	NA	LBD21.31R	22868559	22868732	-	upstream	-6011
16	12	NA	LBD21.31R	23765393	23767140	+	upstream	-858
27	24	NA	LBD21.31R	23903060	23906186	-	overlapSt	59
53	51	NA	LBD21.31R	24058841	24059155	+	inside	27
19	16	NA	LBD21.31R	24328370	24330177	-	upstream	-9747
28	25	NA	LBD21.31R	24736998	24739216	-	overlapEn	2319
19	17	NA	LBD21.31R	24750711	24753375	+	inside	1085
24	20	NA	LBD21.31R	24958678	24959361	+	upstream	-699
20	18	NA	LBD21.31R	25091698	25095512	+	upstream	-950
18	15	NA	LBD21.31R	25138768	25142143	+	upstream	-300
26	21	NA	LBD21.31R	69145	71538	+	upstream	-7174
17	14	NA	LBD21.31R	142308	149307	+	upstream	-12652
23	21	NA	LBD21.31R	655424	657531	+	inside	690
19	18	NA	LBD21.31R	1494392	1496547	+	inside	1602
17	15	NA	LBD21.31R	1504041	1509723	-	upstream	-4120
21	19	NA	LBD21.31R	2550013	2551810	-	upstream	-42
26	23	NA	LBD21.31R	2707918	2709233	-	upstream	-3600
42	40	NA	LBD21.31R	2889061	2891328	-	upstream	-12525
22	21	NA	LBD21.31R	3020018	3020430	+	upstream	-1963
20	18	NA	LBD21.31R	3022134	3022642	-	inside	396
26	23	NA	LBD21.31R	3374216	3375340	+	downstrea	2483
33	31	NA	LBD21.31R	3485423	3486054	+	downstrea	6330
21	19	NA	LBD21.31R	3501583	3509760	+	inside	4959
17	14	NA	LBD21.31R	3652099	3652896	+	upstream	-1964
60	58	NA	LBD21.31R	3826628	3826894	+	inside	16
16	12	NA	LBD21.31R	4436631	4441053	+	downstrea	8150
24	20	NA	LBD21.31R	4463882	4469795	+	upstream	-2232
24	21	NA	LBD21.31R	4749885	4750656	-	upstream	-5016
17	15	NA	LBD21.31R	6152297	6153934	+	downstrea	9698
23	19	NA	LBD21.31R	6271853	6272056	+	upstream	-4075
19	16	NA	LBD21.31R	6415789	6419435	+	downstrea	7249
20	17	NA	LBD21.31R	6779986	6784041	-	downstrea	17922
18	16	NA	LBD21.31R	7197769	7198429	+	upstream	-1329
19	16	NA	LBD21.31R	8387010	8387117	+	upstream	-6008
84	82	NA	LBD21.31R	9386830	9388800	+	upstream	-788
41	40	NA	LBD21.31R	9389396	9390123	+	overlapSt	-227
25	23	NA	LBD21.31R	9566251	9567033	+	overlapEn	436
55	53	NA	LBD21.31R	9567815	9569026	-	upstream	-188
51	49	NA	LBD21.31R	10183128	10183823	-	downstrea	1744
17	15	NA	LBD21.31R	10357263	10363169	+	inside	1795
16	14	NA	LBD21.31R	10534366	10538515	+	inside	723
21	19	NA	LBD21.31R	10604134	10617528	-	inside	1762
19	16	NA	LBD21.31R	11128461	11131462	+	upstream	-2027
19	17	NA	LBD21.31R	11573150	11583281	+	inside	382
21	19	NA	LBD21.31R	11614280	11621015	+	inside	4750
16	14	NA	LBD21.31R	12027557	12029242	-	inside	1573
18	15	NA	LBD21.31R	12305986	12311549	+	upstream	-2467
19	16	NA	LBD21.31R	12432595	12437661	+	upstream	-4919

21	19	NA	LBD21.31R	12729229	12730819	+	downstrea	6437
19	17	NA	LBD21.31R	12753349	12755327	-	downstrea	12743
17	15	NA	LBD21.31R	13040521	13046522	-	inside	2590
23	20	NA	LBD21.31R	13049039	13055620	-	inside	504
18	16	NA	LBD21.31R	13105319	13107221	+	upstream	-1197
17	15	NA	LBD21.31R	13156727	13175786	+	inside	1597
18	16	NA	LBD21.31R	13183164	13184572	+	inside	367
23	21	NA	LBD21.31R	13699658	13700594	+	upstream	-2174
21	17	NA	LBD21.31R	14047746	14056301	-	upstream	-4134
20	18	NA	LBD21.31R	14189779	14191428	-	inside	1371
24	21	NA	LBD21.31R	14265134	14269761	-	inside	1945
19	16	NA	LBD21.31R	15103453	15104818	-	upstream	-728
17	15	NA	LBD21.31R	15324744	15330797	-	upstream	-3067
22	20	NA	LBD21.31R	15379900	15381837	-	inside	1340
20	16	NA	LBD21.31R	15551696	15552231	+	downstrea	2095
22	18	NA	LBD21.31R	15709563	15713084	+	downstrea	5827
31	30	NA	LBD21.31R	15835933	15838778	+	inside	1790
17	14	NA	LBD21.31R	16372582	16375217	-	upstream	-7653
16	14	NA	LBD21.31R	16625709	16626938	-	inside	1223
38	36	NA	LBD21.31R	16701064	16705723	+	upstream	-1292
19	16	NA	LBD21.31R	17002532	17008723	+	downstrea	12664
18	16	NA	LBD21.31R	17270911	17274746	+	inside	696
19	15	NA	LBD21.31R	17596241	17597637	+	upstream	-1237
17	14	NA	LBD21.31R	17766081	17766461	+	upstream	-5679
24	21	NA	LBD21.31R	17950578	17950986	+	downstrea	6130
19	15	NA	LBD21.31R	18368222	18372666	+	upstream	-5098
25	22	NA	LBD21.31R	18395232	18397016	+	overlapEn	1720
18	14	NA	LBD21.31R	18479899	18482747	+	upstream	-3128
29	25	NA	LBD21.31R	18770564	18771906	-	upstream	-550
18	16	NA	LBD21.31R	18815797	18817888	+	inside	1055
22	19	NA	LBD21.31R	19294354	19294875	+	downstrea	892
24	21	NA	LBD21.31R	19297875	19299786	+	upstream	-1092
21	20	NA	LBD21.31R	19531740	19535025	-	inside	1054
24	22	NA	LBD21.31R	19567602	19575186	+	inside	971
17	16	NA	LBD21.31R	19598729	19599608	-	overlapEn	1028
17	15	NA	LBD21.31R	19927435	19928668	-	inside	945
17	14	NA	LBD21.31R	20145496	20149321	-	inside	2718
26	24	NA	LBD21.31R	20173864	20180085	-	inside	809
22	20	NA	LBD21.31R	20186834	20195032	+	inside	7583
53	50	NA	LBD21.31R	20294523	20302361	-	inside	4825
21	18	NA	LBD21.31R	20353339	20357188	+	upstream	-3136
22	20	NA	LBD21.31R	20398547	20400664	+	inside	778
28	26	NA	LBD21.31R	20447558	20452100	+	inside	138
22	19	NA	LBD21.31R	20542729	20543495	+	overlapSt	-48
17	14	NA	LBD21.31R	20603112	20606057	+	upstream	-7472
16	13	NA	LBD21.31R	20665302	20666936	+	upstream	-7935
17	15	NA	LBD21.31R	20740174	20744146	-	downstrea	5916
21	18	NA	LBD21.31R	20790141	20795989	+	upstream	-6455
20	18	NA	LBD21.31R	20931566	20932094	-	overlapEn	684
20	18	NA	LBD21.31R	21312279	21313030	-	inside	642
16	14	NA	LBD21.31R	21381434	21382712	-	upstream	-458
20	17	NA	LBD21.31R	21409350	21414541	+	upstream	-2333
21	18	NA	LBD21.31R	21514119	21516242	-	downstrea	3910
17	15	NA	LBD21.31R	21526754	21527456	+	inside	352

19	17	NA	LBD21.31R	21532013	21534183	-	overlapEn	2186
23	19	NA	LBD21.31R	140890	145444	+	inside	1296
19	16	NA	LBD21.31R	525880	527127	+	downstrea	3425
22	19	NA	LBD21.31R	808810	809677	+	downstrea	2421
21	20	NA	LBD21.31R	1296320	1297652	+	inside	870
23	20	NA	LBD21.31R	2269548	2272045	-	inside	1698
19	15	NA	LBD21.31R	2470639	2475043	+	inside	3902
16	14	NA	LBD21.31R	2570949	2589685	-	upstream	-122
18	16	NA	LBD21.31R	2774597	2781846	-	inside	2961
17	14	NA	LBD21.31R	2795406	2799775	-	upstream	-3301
17	15	NA	LBD21.31R	2817856	2822411	-	inside	2639
17	15	NA	LBD21.31R	2912268	2913050	-	overlapEn	1006
22	18	NA	LBD21.31R	3417387	3417957	-	upstream	-2830
18	15	NA	LBD21.31R	3793576	3794363	-	upstream	-7592
18	16	NA	LBD21.31R	3857938	3859961	-	upstream	-1485
27	25	NA	LBD21.31R	3868748	3870670	-	inside	1254
21	19	NA	LBD21.31R	3875028	3876013	+	inside	21
24	21	NA	LBD21.31R	4187175	4187502	+	downstrea	2836
26	23	NA	LBD21.31R	4243164	4247903	+	inside	851
19	15	NA	LBD21.31R	4325805	4327812	+	downstrea	3000
19	15	NA	LBD21.31R	4424932	4425619	-	downstrea	7219
32	28	NA	LBD21.31R	4541867	4544012	+	upstream	-4152
21	18	NA	LBD21.31R	4532918	4551085	-	upstream	-1893
18	16	NA	LBD21.31R	4751992	4757233	+	upstream	-1700
17	15	NA	LBD21.31R	5085603	5092563	+	inside	2922
18	14	NA	LBD21.31R	5191587	5192571	-	overlapSt	191
18	14	NA	LBD21.31R	5238500	5250295	+	upstream	-4403
19	16	NA	LBD21.31R	6105301	6106633	-	inside	450
28	24	NA	LBD21.31R	6269473	6273663	+	inside	277
17	15	NA	LBD21.31R	6276672	6280347	+	inside	221
22	20	NA	LBD21.31R	6300120	6308679	-	upstream	-3040
18	16	NA	LBD21.31R	6778244	6781216	+	inside	147
19	16	NA	LBD21.31R	6958715	6961637	+	upstream	-3588
16	12	NA	LBD21.31R	7568848	7572266	+	upstream	-10559
20	18	NA	LBD21.31R	7803371	7805739	-	downstrea	2816
18	16	NA	LBD21.31R	8206433	8208989	+	overlapEn	2328
18	15	NA	LBD21.31R	8367867	8368944	+	downstrea	1276
18	15	NA	LBD21.31R	8421268	8421646	+	downstrea	6005
18	16	NA	LBD21.31R	8809185	8846693	-	inside	15894
30	29	NA	LBD21.31R	8905575	8908083	+	inside	964
16	12	NA	LBD21.31R	9285507	9287297	-	upstream	-5176
17	15	NA	LBD21.31R	9308585	9323572	-	inside	848
33	32	NA	LBD21.31R	9511345	9512253	+	upstream	-7587
18	15	NA	LBD21.31R	10281961	10287827	-	upstream	-136
37	35	NA	LBD21.31R	10314112	10321954	+	inside	1140
23	20	NA	LBD21.31R	10365239	10365973	+	downstrea	10255
23	21	NA	LBD21.31R	10376122	10386705	-	upstream	-6028
35	30	NA	LBD21.31R	10454286	10454717	+	upstream	-6363
17	14	NA	LBD21.31R	11929544	11932387	-	upstream	-2539
20	18	NA	LBD21.31R	12145001	12147276	+	inside	295
20	17	NA	LBD21.31R	12337842	12338677	-	downstrea	5136
36	35	NA	LBD21.31R	12466740	12468284	-	upstream	-4135
20	17	NA	LBD21.31R	12590162	12594879	+	downstrea	25790
39	37	NA	LBD21.31R	12652576	12652938	-	upstream	-28496

65	63	NA	LBD21.31R	13124991	13125335	-	upstream	-75367
18	15	NA	LBD21.31R	14319039	14325165	+	upstream	-9181
37	36	NA	LBD21.31R	14349106	14352191	+	downstrea	9917
40	38	NA	LBD21.31R	14414010	14416706	+	downstrea	44084
30	26	NA	LBD21.31R	14903140	14906264	+	upstream	-3608
30	25	NA	LBD21.31R	15275096	15286884	+	upstream	-7382
34	32	NA	LBD21.31R	16134853	16136863	+	downstrea	3906
17	15	NA	LBD21.31R	16448141	16449147	-	downstrea	1555
24	21	NA	LBD21.31R	16762048	16763104	-	overlapEn	1222
18	15	NA	LBD21.31R	16995134	16998705	+	inside	2505
20	17	NA	LBD21.31R	17205151	17208026	+	inside	1298
22	18	NA	LBD21.31R	17748145	17751189	+	inside	2644
21	18	NA	LBD21.31R	18111861	18112040	+	downstrea	2674
18	15	NA	LBD21.31R	18167700	18173549	+	upstream	-8785
19	16	NA	LBD21.31R	18519395	18520295	+	upstream	-3096
22	20	NA	LBD21.31R	18538349	18538577	-	upstream	-478
28	26	NA	LBD21.31R	18672434	18673602	-	upstream	-4152
19	17	NA	LBD21.31R	18790261	18793854	+	inside	593
26	24	NA	LBD21.31R	19044352	19047726	+	inside	2308
18	16	NA	LBD21.31R	19238092	19239534	+	inside	35
26	24	NA	LBD21.31R	19645993	19651241	-	inside	818
19	16	NA	LBD21.31R	19911899	19912768	+	upstream	-1338
30	28	NA	LBD21.31R	20203128	20207004	-	inside	489
28	24	NA	LBD21.31R	20353847	20355967	+	downstrea	2643
19	17	NA	LBD21.31R	20855645	20857045	+	downstrea	1916
57	55	NA	LBD21.31R	20885873	20888083	+	inside	1855
20	17	NA	LBD21.31R	21024480	21031297	-	inside	416
20	18	NA	LBD21.31R	21396958	21401302	+	inside	2362
34	32	NA	LBD21.31R	21546474	21548258	+	downstrea	4858
24	22	NA	LBD21.31R	21612481	21617888	+	inside	3061
20	18	NA	LBD21.31R	22413113	22414246	-	upstream	-6456
26	23	NA	LBD21.31R	22822148	22822804	+	overlapEn	509
35	32	NA	LBD21.31R	22881087	22884413	-	inside	840
17	15	NA	LBD21.31R	23312853	23320060	+	inside	2018
17	15	NA	LBD21.31R	23554290	23559921	-	inside	2537
22	19	NA	LBD21.31R	24096936	24103665	+	inside	2064
18	14	NA	LBD21.31R	24104206	24106928	-	upstream	-16208
44	42	NA	LBD21.31R	24164253	24172484	-	inside	1764
20	17	NA	LBD21.31R	2351	8719	-	inside	5676
17	14	NA	LBD21.31R	347790	350058	+	downstrea	2572
18	15	NA	LBD21.31R	759189	767490	+	inside	306
27	24	NA	LBD21.31R	833012	833816	+	downstrea	2666
20	18	NA	LBD21.31R	1477025	1486732	+	upstream	-3303
31	29	NA	LBD21.31R	1854298	1856108	-	overlapSt	322
36	34	NA	LBD21.31R	1876628	1880313	-	inside	911
24	21	NA	LBD21.31R	2226212	2233881	-	inside	3924
20	18	NA	LBD21.31R	3149146	3149364	-	upstream	-221
18	15	NA	LBD21.31R	3189385	3195663	-	inside	2631
17	15	NA	LBD21.31R	3289322	3291712	-	inside	450
17	14	NA	LBD21.31R	4017952	4018194	-	upstream	-1728
21	20	NA	LBD21.31R	4181343	4182149	+	downstrea	2640
29	27	NA	LBD21.31R	4182330	4187153	-	inside	1828
19	16	NA	LBD21.31R	4366161	4366712	-	downstrea	5473
17	14	NA	LBD21.31R	4534405	4536336	+	inside	977

18	15	NA	LBD21.31R	4547068	4549123	+	upstream	-4828
17	15	NA	LBD21.31R	4712291	4712926	+	overlapEn	313
16	13	NA	LBD21.31R	5279980	5285296	+	upstream	-1068
18	16	NA	LBD21.31R	5408603	5417613	+	inside	5291
26	23	NA	LBD21.31R	5591213	5595844	-	upstream	-2841
18	15	NA	LBD21.31R	5704544	5706720	-	downstrea	18001
22	18	NA	LBD21.31R	5847667	5849956	+	upstream	-3545
18	16	NA	LBD21.31R	5988631	5989114	-	upstream	-3773
17	15	NA	LBD21.31R	6087657	6088835	+	upstream	-3479
17	14	NA	LBD21.31R	6187569	6188933	+	upstream	-1543
22	19	NA	LBD21.31R	6349752	6352599	+	inside	2340
25	22	NA	LBD21.31R	6474126	6478918	-	inside	2028
64	62	NA	LBD21.31R	6898522	6901736	+	inside	1629
18	16	NA	LBD21.31R	7170333	7173494	+	upstream	-4730
26	22	NA	LBD21.31R	7383725	7385275	+	upstream	-5212
18	15	NA	LBD21.31R	7437254	7439962	+	upstream	-1065
23	19	NA	LBD21.31R	7693630	7694226	-	downstrea	1160
24	20	NA	LBD21.31R	7891300	7891560	+	upstream	-906
17	15	NA	LBD21.31R	8358719	8365054	-	inside	1987
21	19	NA	LBD21.31R	8401879	8410117	+	inside	3019
19	16	NA	LBD21.31R	8431668	8432811	+	upstream	-3926
20	18	NA	LBD21.31R	8842332	8846582	+	inside	3283
16	13	NA	LBD21.31R	8914536	8917823	+	overlapSt	-158
18	16	NA	LBD21.31R	10887568	10887879	+	downstrea	7599
18	15	NA	LBD21.31R	11231800	11232786	-	overlapSt	206
18	14	NA	LBD21.31R	11265842	11266159	-	upstream	-827
17	15	NA	LBD21.31R	11531670	11534520	-	inside	933
22	19	NA	LBD21.31R	11613891	11617243	+	upstream	-6636
42	39	NA	LBD21.31R	11705721	11709426	-	inside	353
19	16	NA	LBD21.31R	11805773	11809765	+	downstrea	4704
24	20	NA	LBD21.31R	11988603	11991909	-	upstream	-8579
20	16	NA	LBD21.31R	12269399	12271223	+	downstrea	10202
30	27	NA	LBD21.31R	12331860	12348948	+	inside	12762
39	37	NA	LBD21.31R	12725031	12730565	+	downstrea	18901
16	13	NA	LBD21.31R	12770351	12773239	+	upstream	-18306
21	18	NA	LBD21.31R	13235178	13241581	-	inside	1659
23	20	NA	LBD21.31R	13425154	13425571	+	includeFe	-24
58	56	NA	LBD21.31R	13493293	13493694	+	inside	118
30	28	NA	LBD21.31R	13495859	13496047	-	upstream	-8663
20	17	NA	LBD21.31R	13548653	13549048	+	upstream	-1283
30	28	NA	LBD21.31R	14532529	14533505	+	upstream	-7990
93	90	NA	LBD21.31R	14813787	14814410	-	overlapSt	135
40	38	NA	LBD21.31R	14824857	14826149	-	inside	224
18	15	NA	LBD21.31R	16044822	16048596	+	upstream	-6359
17	15	NA	LBD21.31R	16083491	16087691	-	inside	1677
17	15	NA	LBD21.31R	16241470	16242775	-	upstream	-20632
43	42	NA	LBD21.31R	16507797	16510707	+	upstream	-9427
18	15	NA	LBD21.31R	16990684	16994688	-	upstream	-13678
36	34	NA	LBD21.31R	17714154	17715725	-	upstream	-2480
16	12	NA	LBD21.31R	17892563	17899118	-	inside	1802
17	15	NA	LBD21.31R	18573573	18578651	-	inside	954
20	16	NA	LBD21.31R	18627145	18627938	+	upstream	-16405
16	12	NA	LBD21.31R	18668951	18671728	+	upstream	-4157
20	17	NA	LBD21.31R	18958391	18961149	+	upstream	-1851

40	36	NA	LBD21.31R	19147416	19150201	+	upstream	-305
21	18	NA	LBD21.31R	19181611	19182726	-	upstream	-1532
18	16	NA	LBD21.31R	19297174	19301357	+	downstrea	4198
19	17	NA	LBD21.31R	19329500	19334579	+	inside	2411
32	31	NA	LBD21.31R	19611383	19622976	-	upstream	-3399
41	40	NA	LBD21.31R	20549534	20550472	-	downstrea	5580
22	19	NA	LBD21.31R	20600549	20603875	-	upstream	-6145
24	21	NA	LBD21.31R	20755700	20756738	+	overlapSt	-53
18	16	NA	LBD21.31R	20981407	20986758	+	inside	3605
23	21	NA	LBD21.31R	21352942	21358912	-	inside	821
17	15	NA	LBD21.31R	21433863	21438717	-	inside	1408
17	15	NA	LBD21.31R	21461159	21463172	+	inside	1302
22	20	NA	LBD21.31R	21480538	21486638	-	inside	1363
28	27	NA	LBD21.31R	21881226	21882935	+	downstrea	2536
22	18	NA	LBD21.31R	21946606	21947143	+	downstrea	2538
24	22	NA	LBD21.31R	21981629	21983177	-	inside	547
17	15	NA	LBD21.31R	22183203	22186356	-	upstream	-5490
68	64	NA	LBD21.31R	22394468	22396557	-	inside	1774
16	14	NA	LBD21.31R	22534222	22534428	+	overlapEn	158
22	20	NA	LBD21.31R	22582369	22583519	+	upstream	-1514
16	12	NA	LBD21.31R	22760031	22764264	-	upstream	-3733
36	34	NA	LBD21.31R	22985956	22986419	-	overlapSt	425
19	17	NA	LBD21.31R	23030899	23036682	+	inside	506
25	21	NA	LBD21.31R	23570628	23572383	+	downstrea	5395
17	14	NA	LBD21.31R	23600307	23604582	-	inside	2658
22	20	NA	LBD21.31R	23664913	23671039	+	overlapSt	-51
19	16	NA	LBD21.31R	24079656	24082417	-	upstream	-1272
18	15	NA	LBD21.31R	24202969	24205332	-	upstream	-655
17	15	NA	LBD21.31R	24283167	24285817	-	inside	589
22	19	NA	LBD21.31R	24438183	24444834	+	upstream	-8604
18	14	NA	LBD21.31R	24789162	24790870	+	upstream	-4062
19	16	NA	LBD21.31R	24880386	24881758	-	downstrea	4474
18	15	NA	LBD21.31R	24983374	24984664	-	inside	790
22	20	NA	LBD21.31R	25225915	25231843	-	inside	1020
18	15	NA	LBD21.31R	25755665	25758828	+	downstrea	4253
17	15	NA	LBD21.31R	27537	30044	-	downstrea	10498
16	12	NA	LBD21.31R	62848	66691	-	upstream	-6810
20	18	NA	LBD21.31R	587389	588339	+	upstream	-3122
21	18	NA	LBD21.31R	675517	676354	-	downstrea	1524
17	14	NA	LBD21.31R	703686	703967	+	downstrea	2664
17	14	NA	LBD21.31R	998795	999130	-	inside	221
20	17	NA	LBD21.31R	1211435	1212355	-	overlapEn	1173
17	15	NA	LBD21.31R	1463229	1468233	+	inside	836
31	27	NA	LBD21.31R	1626750	1628824	-	inside	241
23	20	NA	LBD21.31R	1731653	1736841	+	inside	434
33	31	NA	LBD21.31R	2689811	2705764	-	inside	1078
17	15	NA	LBD21.31R	3652919	3653326	-	downstrea	726
17	16	NA	LBD21.31R	3655493	3666168	+	inside	663
17	15	NA	LBD21.31R	3671522	3674425	-	overlapSt	265
21	18	NA	LBD21.31R	3854946	3858521	+	inside	3268
17	15	NA	LBD21.31R	4148448	4152549	-	inside	2991
25	23	NA	LBD21.31R	4499695	4504592	+	upstream	-3686
18	16	NA	LBD21.31R	4523335	4532993	-	inside	2398
18	17	NA	LBD21.31R	4611204	4615026	-	inside	3395

23	19	NA	LBD21.31R	4908071	4915141	-	inside	2390
16	13	NA	LBD21.31R	4996504	5006232	+	upstream	-3122
19	16	NA	LBD21.31R	5396646	5401181	-	upstream	-1849
23	20	NA	LBD21.31R	5451266	5458349	+	inside	4815
26	24	NA	LBD21.31R	5854711	5855455	+	inside	409
17	15	NA	LBD21.31R	6095567	6107810	+	inside	1564
21	18	NA	LBD21.31R	6260384	6264486	+	upstream	-251
17	13	NA	LBD21.31R	6510362	6512593	+	upstream	-599
25	22	NA	LBD21.31R	6517063	6518158	-	upstream	-4611
23	20	NA	LBD21.31R	6538210	6541462	-	inside	3147
16	14	NA	LBD21.31R	6736902	6738013	-	downstrea	2951
20	17	NA	LBD21.31R	7188529	7192013	+	inside	686
18	16	NA	LBD21.31R	7600982	7602379	+	inside	1076
16	12	NA	LBD21.31R	7746324	7748908	-	upstream	-5079
17	15	NA	LBD21.31R	8154913	8157233	-	inside	812
25	21	NA	LBD21.31R	8219970	8223230	+	upstream	-1561
19	17	NA	LBD21.31R	8524655	8535097	-	inside	5444
24	21	NA	LBD21.31R	8701663	8703532	-	upstream	-2274
19	17	NA	LBD21.31R	9336398	9340989	-	inside	1859
16	12	NA	LBD21.31R	9445700	9447868	+	upstream	-2668
18	15	NA	LBD21.31R	9461415	9496093	+	upstream	-3875
30	26	NA	LBD21.31R	10438380	10439692	-	upstream	-1033
20	18	NA	LBD21.31R	10470188	10470993	+	overlapEn	483
39	37	NA	LBD21.31R	11151518	11151907	+	upstream	-9337
18	16	NA	LBD21.31R	11178169	11178641	+	downstrea	3011
22	18	NA	LBD21.31R	11370750	11373810	-	upstream	-9223
17	13	NA	LBD21.31R	11502823	11503288	+	overlapEn	412
24	21	NA	LBD21.31R	11739873	11742124	-	downstrea	2629
28	23	NA	LBD21.31R	12135840	12136248	+	downstrea	4673
16	12	NA	LBD21.31R	12776413	12780760	+	upstream	-3466
17	14	NA	LBD21.31R	12897437	12901197	-	inside	3425
30	28	NA	LBD21.31R	12953016	12957023	-	upstream	-825
20	16	NA	LBD21.31R	13610915	13615203	-	upstream	-5420
28	25	NA	LBD21.31R	14061665	14068002	+	upstream	-4554
19	15	NA	LBD21.31R	15108747	15111743	-	upstream	-4107
21	19	NA	LBD21.31R	15215286	15217572	-	downstrea	3302
51	47	NA	LBD21.31R	15248067	15250270	-	upstream	-28172
69	67	NA	LBD21.31R	16002086	16002300	+	upstream	-13189
22	21	NA	LBD21.31R	16330173	16330536	+	downstrea	8297
17	14	NA	LBD21.31R	16554131	16565217	+	inside	5671
23	19	NA	LBD21.31R	16923804	16931618	+	upstream	-6583
29	25	NA	LBD21.31R	17173988	17174164	+	upstream	-3794
26	24	NA	LBD21.31R	17626093	17627487	+	downstrea	23779
25	21	NA	LBD21.31R	17846159	17847605	+	upstream	-2623
20	17	NA	LBD21.31R	18378703	18385024	-	overlapSt	37
21	17	NA	LBD21.31R	18427409	18428888	-	upstream	-2671
40	38	NA	LBD21.31R	18633690	18636357	-	downstrea	3598
27	25	NA	LBD21.31R	18911364	18919423	-	inside	5193
39	33	NA	LBD21.31R	19005679	19006954	-	upstream	-13183
23	19	NA	LBD21.31R	19392135	19393336	-	inside	515
23	21	NA	LBD21.31R	19616220	19621043	-	upstream	-3789
21	18	NA	LBD21.31R	20063876	20065794	+	upstream	-21809
21	18	NA	LBD21.31R	20100118	20103675	-	upstream	-483
19	16	NA	LBD21.31R	20907598	20912145	-	upstream	-5392

16	12	NA	LBD21.31R	21034572	21035402	-	upstream	-19062
53	51	NA	LBD21.31R	21209507	21209889	+	overlapEn	78
17	15	NA	LBD21.31R	21506066	21506903	+	inside	405
35	34	NA	LBD21.31R	21744980	21746664	-	downstrea	5796
25	22	NA	LBD21.31R	22064648	22069887	+	upstream	-12260
18	15	NA	LBD21.31R	22262176	22265633	-	upstream	-7487
30	28	NA	LBD21.31R	22659114	22660766	+	inside	440
19	16	NA	LBD21.31R	23472013	23475138	+	upstream	-3427
27	24	NA	LBD21.31R	23751412	23754666	+	upstream	-12484
18	15	NA	LBD21.31R	23959220	23963225	-	downstrea	5411
18	14	NA	LBD21.31R	24412663	24417659	+	upstream	-3785
19	16	NA	LBD21.31R	24635800	24636399	+	downstrea	4428
18	14	NA	LBD21.31R	24667224	24674234	-	upstream	-4224
17	15	NA	LBD21.31R	24983306	24984851	-	downstrea	2866
20	18	NA	LBD21.31R	24989619	24992901	-	upstream	-2310
19	16	NA	LBD21.31R	25124468	25127705	+	inside	32
17	14	NA	LBD21.31R	25774745	25775792	+	downstrea	2531
28	24	NA	LBD21.31R	25930994	25935700	-	upstream	-4269
16	12	NA	LBD21.31R	26231739	26233670	-	downstrea	2220
17	14	NA	LBD21.31R	26468721	26470660	+	downstrea	4842
20	18	NA	LBD21.31R	26632445	26633154	+	downstrea	3200
19	16	NA	LBD21.31R	26745119	26745769	-	downstrea	3566
16	14	NA	LBD21.31R	26748955	26753066	+	inside	862
18	15	NA	LBD21.31R	27444313	27447739	-	inside	1362
21	19	NA	LBD21.31R	27621859	27629894	-	inside	4094
24	21	NA	LBD21.31R	27736852	27737235	-	upstream	-2029
18	15	NA	LBD21.31R	212807	213915	-	upstream	-1087
17	14	NA	LBD21.31R	1002705	1005406	-	upstream	-2142
21	17	NA	LBD21.31R	1442351	1442648	-	upstream	-3258
19	16	NA	LBD21.31R	1639493	1640810	-	upstream	-30
21	18	NA	LBD21.31R	2415499	2419192	-	inside	1146
19	16	NA	LBD21.31R	2826103	2828296	-	upstream	-265
54	52	NA	LBD21.31R	3252284	3254824	-	inside	2489
19	15	NA	LBD21.31R	3376046	3376747	+	upstream	-5283
16	12	NA	LBD21.31R	3412336	3412617	-	upstream	-6954
18	15	NA	LBD21.31R	3566099	3570373	-	upstream	-7985
16	14	NA	LBD21.31R	3779080	3781182	-	inside	1701
26	25	NA	LBD21.31R	4759259	4760930	-	upstream	-10203
19	15	NA	LBD21.31R	5114576	5118175	+	upstream	-11829
19	17	NA	LBD21.31R	5259789	5263992	-	inside	2560
18	15	NA	LBD21.31R	5661027	5662744	+	downstrea	14913
24	23	NA	LBD21.31R	5978584	5981095	+	upstream	-4337
27	26	NA	LBD21.31R	6534123	6536798	-	upstream	-971
16	15	NA	LBD21.31R	6538623	6540372	-	inside	1401
23	21	NA	LBD21.31R	6683311	6684888	-	downstrea	5343
29	27	NA	LBD21.31R	7250907	7251317	+	upstream	-3483
26	24	NA	LBD21.31R	7974628	7975279	+	downstrea	4338
17	15	NA	LBD21.31R	8144143	8149905	+	inside	2927
34	31	NA	LBD21.31R	8205831	8210012	-	inside	1101
23	20	NA	LBD21.31R	8223603	8230240	+	inside	3941
29	24	NA	LBD21.31R	8776939	8780271	+	upstream	-3043
18	16	NA	LBD21.31R	9400119	9403174	-	downstrea	3837
23	20	NA	LBD21.31R	9468081	9472152	-	upstream	-4384
61	59	NA	LBD21.31R	10281245	10311187	+	inside	3955

23	19	NA	LBD21.31R	10413398	10415268	+	downstrea	2745
21	18	NA	LBD21.31R	10542508	10549296	-	inside	1376
23	20	NA	LBD21.31R	11109651	11113754	+	inside	2724
20	16	NA	LBD21.31R	11159989	11167075	+	upstream	-2915
16	14	NA	LBD21.31R	11193574	11194068	-	inside	470
20	17	NA	LBD21.31R	11686122	11688658	+	downstrea	4230
29	25	NA	LBD21.31R	11715514	11715907	-	downstrea	6902
17	14	NA	LBD21.31R	12138490	12140768	-	downstrea	3293
20	18	NA	LBD21.31R	12162182	12163625	-	upstream	-876
17	14	NA	LBD21.31R	12528768	12531281	-	downstrea	2941
17	15	NA	LBD21.31R	12652323	12671801	-	inside	5520
23	21	NA	LBD21.31R	12657150	12657681	+	downstrea	7015
17	15	NA	LBD21.31R	12678172	12681640	-	upstream	-2883
33	29	NA	LBD21.31R	12704588	12708177	-	inside	2010
24	21	NA	LBD21.31R	12941962	12944072	+	inside	71
22	19	NA	LBD21.31R	13002911	13007361	+	upstream	-552
17	15	NA	LBD21.31R	13277490	13281767	+	inside	1043
25	23	NA	LBD21.31R	13309004	13313082	-	inside	1916
46	44	NA	LBD21.31R	13754957	13755313	-	upstream	-3759
30	28	NA	LBD21.31R	14208454	14211053	-	inside	433
17	14	NA	LBD21.31R	14911216	14914519	+	inside	74
25	22	NA	LBD21.31R	15008447	15013847	+	inside	1001
18	17	NA	LBD21.31R	15160115	15162536	-	inside	941
27	25	NA	LBD21.31R	15180717	15180979	-	upstream	-2527
27	25	NA	LBD21.31R	15314118	15322630	+	inside	3964
20	18	NA	LBD21.31R	15358332	15361898	-	inside	3162
16	12	NA	LBD21.31R	15582983	15584617	+	upstream	-4416
38	37	NA	LBD21.31R	379940	380409	+	inside	128
20	16	NA	LBD21.31R	1518098	1518765	-	downstrea	1711
20	18	NA	LBD21.31R	1782197	1783753	-	downstrea	5028
17	15	NA	LBD21.31R	1985358	1989952	+	downstrea	5789
17	14	NA	LBD21.31R	2002373	2002860	-	upstream	-3813
19	17	NA	LBD21.31R	2800339	2810370	+	inside	3211
39	37	NA	LBD21.31R	2811985	2812920	-	upstream	-2098
19	16	NA	LBD21.31R	3005811	3006909	+	upstream	-392
17	14	NA	LBD21.31R	3017623	3018405	+	downstrea	3659
17	15	NA	LBD21.31R	3050530	3056239	-	inside	3978
17	15	NA	LBD21.31R	3162907	3164822	-	upstream	-2287
17	15	NA	LBD21.31R	3220515	3223122	-	inside	804
17	15	NA	LBD21.31R	3227297	3230214	-	upstream	-2423
16	13	NA	LBD21.31R	3564658	3566968	+	downstrea	2678
23	21	NA	LBD21.31R	3612661	3614813	-	upstream	-1244
37	35	NA	LBD21.31R	3695827	3697846	-	downstrea	2264
28	25	NA	LBD21.31R	3776560	3779286	-	upstream	-2300
19	17	NA	LBD21.31R	4476846	4478817	+	inside	44
20	17	NA	LBD21.31R	4513635	4514673	+	downstrea	3026
19	17	NA	LBD21.31R	4866197	4868458	+	downstrea	2636
19	15	NA	LBD21.31R	5212150	5216225	-	inside	1750
32	29	NA	LBD21.31R	5812936	5820235	+	inside	1364
18	15	NA	LBD21.31R	6064176	6065835	-	upstream	-5017
21	17	NA	LBD21.31R	6162096	6169028	+	inside	1705
17	14	NA	LBD21.31R	6626146	6628702	-	overlapEn	2762
17	15	NA	LBD21.31R	6924757	6925966	+	upstream	-1346
22	20	NA	LBD21.31R	6926358	6930611	-	upstream	-4143

23	21	NA	LBD21.31R	7115541	7121312	-	inside	4960
21	18	NA	LBD21.31R	7175275	7178103	-	upstream	-880
19	17	NA	LBD21.31R	7530525	7532880	+	upstream	-4181
17	15	NA	LBD21.31R	7693602	7694606	-	upstream	-359
22	19	NA	LBD21.31R	7843368	7843839	-	inside	375
20	17	NA	LBD21.31R	8006243	8008302	+	inside	392
17	14	NA	LBD21.31R	8011656	8012130	+	downstrea	540
22	19	NA	LBD21.31R	8017574	8018741	-	upstream	-6176
18	16	NA	LBD21.31R	8188054	8188848	+	upstream	-779
22	18	NA	LBD21.31R	8496777	8500194	-	downstrea	7301
23	20	NA	LBD21.31R	8631059	8632295	-	inside	935
17	14	NA	LBD21.31R	9124770	9128433	+	inside	3025
17	14	NA	LBD21.31R	9322167	9329696	+	upstream	-6268
17	15	NA	LBD21.31R	9461153	9464758	+	inside	2105
20	18	NA	LBD21.31R	9665648	9666783	+	downstrea	1956
19	16	NA	LBD21.31R	9860569	9862692	+	downstrea	6560
21	18	NA	LBD21.31R	10167044	10168070	-	upstream	-864
20	17	NA	LBD21.31R	10457421	10458550	-	overlapEn	1130
19	16	NA	LBD21.31R	10513016	10518417	-	upstream	-1473
17	14	NA	LBD21.31R	10597132	10602609	-	upstream	-1531
18	15	NA	LBD21.31R	10763527	10763694	+	upstream	-529
26	24	NA	LBD21.31R	11091933	11092256	+	downstrea	734
17	14	NA	LBD21.31R	11144480	11144996	+	upstream	-692
20	17	NA	LBD21.31R	11911824	11930638	+	upstream	-1549
17	15	NA	LBD21.31R	11997384	12003357	-	inside	3678
33	29	NA	LBD21.31R	12097990	12102875	+	upstream	-4635
16	14	NA	LBD21.31R	12241181	12250048	-	upstream	-402
20	18	NA	LBD21.31R	12401027	12405504	+	inside	84
22	19	NA	LBD21.31R	12417987	12423037	-	inside	3284
21	18	NA	LBD21.31R	12941639	12944369	+	downstrea	3269
19	16	NA	LBD21.31R	13019684	13023254	-	inside	2165
20	17	NA	LBD21.31R	13897185	13901644	-	upstream	-383
28	25	NA	LBD21.31R	14031295	14036463	-	upstream	-5790
19	17	NA	LBD21.31R	14567016	14571279	+	inside	2361
29	26	NA	LBD21.31R	15126261	15127815	-	inside	332
17	14	NA	LBD21.31R	15295465	15298041	-	upstream	-36258
17	14	NA	LBD21.31R	15392707	15395953	+	downstrea	33231
17	15	NA	LBD21.31R	15742390	15744149	-	downstrea	11713
30	26	NA	LBD21.31R	17239823	17240740	+	upstream	-960
26	24	NA	LBD21.31R	17478458	17484271	-	inside	731
22	20	NA	LBD21.31R	17670728	17686704	-	upstream	-8111
18	15	NA	LBD21.31R	18385290	18386273	+	upstream	-8543
17	14	NA	LBD21.31R	18549130	18551151	+	upstream	-1921
46	45	NA	LBD21.31R	18790426	18792329	+	upstream	-10755
28	26	NA	LBD21.31R	190781	191530	+	upstream	-2707
44	43	NA	LBD21.31R	238870	240371	+	downstrea	11972
55	53	NA	LBD21.31R	428906	430507	-	downstrea	16426
61	59	NA	LBD21.31R	989221	989641	+	upstream	-1393
39	38	NA	LBD21.31R	991605	991793	-	overlapEn	609
19	17	NA	LBD21.31R	1268585	1271680	-	inside	1073
29	27	NA	LBD21.31R	1392167	1394565	+	upstream	-6758
69	67	NA	LBD21.31R	2723693	2723921	+	includeFe	-5
67	65	NA	LBD21.31R	2727840	2728220	+	upstream	-502
52	50	NA	LBD21.31R	2730090	2730230	-	includeFe	203

17	15	NA	LBD21.31R	3163709	3164651	-	inside	839
19	15	NA	LBD21.31R	3308909	3309527	+	downstrea	896
22	18	NA	LBD21.31R	3959009	3959907	+	upstream	-4014
17	13	NA	LBD21.31R	3976470	3977823	-	upstream	-6873
25	22	NA	LBD21.31R	4112696	4116361	-	downstrea	4542
17	14	NA	LBD21.31R	5382460	5384224	+	downstrea	2210
17	14	NA	LBD21.31R	5478033	5483711	-	inside	1358
18	15	NA	LBD21.31R	5502089	5508765	+	upstream	-7611
18	16	NA	LBD21.31R	5853166	5857730	+	inside	30
18	15	NA	LBD21.31R	6340254	6342421	-	upstream	-1009
18	16	NA	LBD21.31R	6590436	6593008	-	inside	1520
24	21	NA	LBD21.31R	6851601	6861463	-	inside	1344
55	53	NA	LBD21.31R	7142744	7143769	-	inside	361
17	14	NA	LBD21.31R	7516983	7520363	-	inside	433
19	16	NA	LBD21.31R	7583980	7598628	+	inside	1002
33	32	NA	LBD21.31R	8624944	8627440	-	downstrea	6616
22	19	NA	LBD21.31R	9085740	9090282	-	upstream	-2608
22	19	NA	LBD21.31R	9383370	9387266	+	overlapSt	-90
25	23	NA	LBD21.31R	9495429	9498532	-	inside	1482
22	18	NA	LBD21.31R	9895387	9896087	+	overlapEn	523
19	16	NA	LBD21.31R	10203623	10212959	-	upstream	-904
19	17	NA	LBD21.31R	10238649	10239156	-	upstream	-815
23	19	NA	LBD21.31R	10435173	10436054	+	overlapEn	737
19	15	NA	LBD21.31R	10521412	10522134	-	overlapEn	833
26	23	NA	LBD21.31R	11362951	11367004	-	overlapSt	697
31	28	NA	LBD21.31R	11407300	11410604	+	upstream	-3915
17	13	NA	LBD21.31R	11514609	11516543	-	downstrea	3898
18	15	NA	LBD21.31R	11783112	11788425	-	upstream	-4876
21	19	NA	LBD21.31R	11981991	11983282	-	upstream	-242
20	18	NA	LBD21.31R	12025721	12030665	+	inside	1197
17	15	NA	LBD21.31R	12157574	12165568	-	inside	1076
20	16	NA	LBD21.31R	12339643	12343776	-	inside	2253
18	16	NA	LBD21.31R	12943202	12947095	+	downstrea	5078
19	17	NA	LBD21.31R	493731	500540	-	inside	3175
17	15	NA	LBD21.31R	678213	681875	-	inside	3321
23	21	NA	LBD21.31R	721754	722601	-	upstream	-87
27	24	NA	LBD21.31R	1222633	1223076	-	upstream	-10001
17	15	NA	LBD21.31R	1813262	1829947	+	inside	10561
20	18	NA	LBD21.31R	1975048	1979416	-	inside	3496
19	15	NA	LBD21.31R	2837752	2840631	-	upstream	-9086
18	16	NA	LBD21.31R	3023337	3034937	-	inside	3052
21	18	NA	LBD21.31R	3544886	3545254	+	upstream	-406
52	49	NA	LBD21.31R	3742462	3745247	-	downstrea	13401
20	18	NA	LBD21.31R	3988172	3994613	-	inside	261
21	18	NA	LBD21.31R	4089829	4097781	+	inside	5188
72	68	NA	LBD21.31R	4155808	4161440	+	downstrea	57392
23	19	NA	LBD21.31R	4421608	4427087	-	inside	2223
37	35	NA	LBD21.31R	4663851	4675020	-	upstream	-77240
31	29	NA	LBD21.31R	4853220	4858707	+	upstream	-83177
103	101	NA	LBD21.31R	5229730	5230211	+	inside	47
37	33	NA	LBD21.31R	5669271	5671886	+	upstream	-53243
22	21	NA	LBD21.31R	5747967	5748499	+	inside	32
18	16	NA	LBD21.31R	7324413	7331352	+	inside	821
22	19	NA	LBD21.31R	7610574	7611740	+	upstream	-4281

50	49	NA	LBD21.31R	8333019	8333763	+	inside	58
28	26	NA	LBD21.31R	8336268	8341257	+	upstream	-1585
18	17	NA	LBD21.31R	8926521	8936988	-	inside	2677
17	14	NA	LBD21.31R	9479907	9485010	-	inside	405
19	17	NA	LBD21.31R	10001785	10016269	+	inside	783
19	16	NA	LBD21.31R	10020206	10023357	+	upstream	-287
22	20	NA	LBD21.31R	10139477	10143131	-	inside	2688
17	15	NA	LBD21.31R	10749173	10752701	-	inside	861
20	19	NA	LBD21.31R	11303792	11304397	-	downstrea	2445
17	15	NA	LBD21.31R	11669215	11675372	-	inside	2698
17	14	NA	LBD21.31R	11747167	11755290	+	downstrea	11356
20	17	NA	LBD21.31R	11844016	11848320	-	upstream	-793
20	18	NA	LBD21.31R	11913219	11920057	-	upstream	-1651
20	18	NA	LBD21.31R	12304493	12311027	+	inside	704
29	26	NA	LBD21.31R	12503051	12507304	+	inside	3676
19	16	NA	LBD21.31R	12522077	12523362	+	downstrea	1802
17	13	NA	LBD21.31R	12829827	12830114	-	upstream	-3166
18	16	NA	LBD21.31R	13050510	13064296	-	inside	4615
17	14	NA	LBD21.31R	13116732	13120397	+	upstream	-7180
19	17	NA	LBD21.31R	13132830	13137057	+	inside	1433
21	17	NA	LBD21.31R	13194526	13197475	+	upstream	-1604
17	14	NA	LBD21.31R	13640755	13644147	-	upstream	-4695
20	16	NA	LBD21.31R	13733411	13735324	+	downstrea	3023
24	20	NA	LBD21.31R	14103926	14105188	+	downstrea	1300
19	16	NA	LBD21.31R	14675579	14679750	+	upstream	-8593
18	15	NA	LBD21.31R	14802073	14803969	-	downstrea	3192
25	22	NA	LBD21.31R	14857783	14859253	-	inside	565
17	14	NA	LBD21.31R	15256503	15257054	+	upstream	-1089
23	21	NA	LBD21.31R	15395629	15411849	-	inside	10637
21	17	NA	LBD21.31R	15938397	15939837	+	inside	906
16	13	NA	LBD21.31R	15941658	15942607	+	inside	477
22	19	NA	LBD21.31R	16019876	16024647	-	upstream	-161
30	27	NA	LBD21.31R	16319676	16321044	-	upstream	-241
21	18	NA	LBD21.31R	16512800	16517009	+	overlapSt	-173
25	22	NA	LBD21.31R	17009869	17012319	+	upstream	-1521
17	15	NA	LBD21.31R	17098655	17100780	-	inside	479
20	17	NA	LBD21.31R	17849922	17850905	-	downstrea	3262
26	23	NA	LBD21.31R	18057386	18062543	-	inside	1105
33	29	NA	LBD21.31R	18066606	18069486	+	downstrea	3209
16	14	NA	LBD21.31R	18454527	18457379	+	upstream	-3869
21	18	NA	LBD21.31R	18925431	18927898	-	upstream	-6897
31	29	NA	LBD21.31R	19155230	19162196	+	inside	495
22	19	NA	LBD21.31R	19350296	19350531	+	upstream	-623
26	23	NA	LBD21.31R	19400650	19406402	-	upstream	-4938
39	33	NA	LBD21.31R	19602693	19603725	+	overlapEn	593
41	39	NA	LBD21.31R	19761629	19769106	+	inside	3697
17	14	NA	LBD21.31R	19860664	19863362	+	inside	2284
16	14	NA	LBD21.31R	19928701	19931559	+	inside	1940
18	16	NA	LBD21.31R	19944162	19945552	-	inside	710
18	15	NA	LBD21.31R	20362434	20365631	+	upstream	-1442
17	14	NA	LBD21.31R	20659826	20659990	+	upstream	-3422
23	20	NA	LBD21.31R	20704365	20710341	-	inside	4795
20	17	NA	LBD21.31R	20747463	20750870	-	downstrea	4662
17	15	NA	LBD21.31R	20849855	20856785	+	overlapSt	-146

20	17	NA	LBD21.31R	20955472	20956297	+	overlapSt	-228
17	15	NA	LBD21.31R	21023027	21037872	-	inside	11808
18	15	NA	LBD21.31R	21453186	21453542	-	downstrea	601
25	23	NA	LBD21.31R	22185751	22186563	+	upstream	-4699
16	14	NA	LBD21.31R	22405504	22410495	-	inside	2584
21	19	NA	LBD21.31R	22492584	22495837	+	inside	531
18	14	NA	LBD21.31R	278846	281529	-	inside	1145
48	46	NA	LBD21.31R	1000639	1001969	+	downstrea	8726
29	27	NA	LBD21.31R	1018697	1020664	+	upstream	-1317
20	17	NA	LBD21.31R	1288198	1294255	-	inside	330
16	12	NA	LBD21.31R	1774559	1774945	+	downstrea	3684
19	17	NA	LBD21.31R	3254482	3259695	-	upstream	-4514
21	17	NA	LBD21.31R	4061272	4063211	-	upstream	-2051
19	17	NA	LBD21.31R	4071035	4077856	-	upstream	-2319
21	17	NA	LBD21.31R	4095391	4097498	+	downstrea	2915
17	15	NA	LBD21.31R	4183188	4183487	-	downstrea	3968
18	16	NA	LBD21.31R	4755285	4758524	+	upstream	-1482
69	67	NA	LBD21.31R	5660885	5661364	-	upstream	-14027
19	17	NA	LBD21.31R	5810438	5811395	+	inside	183
22	20	NA	LBD21.31R	6674943	6675394	+	downstrea	961
72	70	NA	LBD21.31R	7193480	7194553	-	inside	666
50	48	NA	LBD21.31R	7196318	7196635	+	upstream	-569
48	47	NA	LBD21.31R	7197641	7197790	-	downstrea	692
27	26	NA	LBD21.31R	7199187	7199534	-	includeFe	453
38	35	NA	LBD21.31R	7203770	7204270	-	upstream	-315
50	47	NA	LBD21.31R	7206842	7207063	-	overlapEn	336
48	46	NA	LBD21.31R	7215822	7217613	+	inside	1162
21	19	NA	LBD21.31R	7230312	7232353	-	inside	245
18	17	NA	LBD21.31R	7730518	7742170	+	inside	69
22	18	NA	LBD21.31R	8006037	8008475	-	downstrea	4059
42	40	NA	LBD21.31R	8288217	8297303	+	upstream	-6736
20	16	NA	LBD21.31R	8738465	8749355	-	upstream	-855
17	13	NA	LBD21.31R	8848739	8849445	-	upstream	-45340
17	15	NA	LBD21.31R	9995156	9995703	+	upstream	-26732
18	14	NA	LBD21.31R	10754371	10769666	-	inside	695
18	17	NA	LBD21.31R	11049147	11049290	+	upstream	-7910
29	26	NA	LBD21.31R	11085280	11087535	-	inside	2224
20	18	NA	LBD21.31R	11644313	11656304	-	inside	9707
30	28	NA	LBD21.31R	12063003	12064255	+	inside	1032
17	14	NA	LBD21.31R	12867984	12868432	-	inside	264
18	15	NA	LBD21.31R	13357083	13360227	-	upstream	-264
22	18	NA	LBD21.31R	13371139	13377581	+	overlapSt	-229
22	20	NA	LBD21.31R	13386596	13391770	-	inside	2578
38	36	NA	LBD21.31R	13861100	13861201	-	upstream	-82
18	16	NA	LBD21.31R	14618174	14623032	+	inside	1569
25	23	NA	LBD21.31R	14658904	14666479	-	inside	2506
17	15	NA	LBD21.31R	15556550	15561394	-	inside	2084
18	16	NA	LBD21.31R	15676763	15682883	-	inside	964
27	23	NA	LBD21.31R	15719348	15719855	+	overlapEn	86
25	22	NA	LBD21.31R	15777117	15777623	+	overlapSt	-419
41	39	NA	LBD21.31R	16057023	16060615	+	upstream	-1141
17	15	NA	LBD21.31R	16094007	16094405	-	upstream	-1894
19	17	NA	LBD21.31R	16137597	16137953	-	upstream	-1217
22	19	NA	LBD21.31R	16378944	16380645	-	downstrea	3874

16	14	NA	LBD21.31R	16536279	16541055	+		inside	800
19	15	NA	LBD21.31R	16785690	16786493	+		downstrea	1861
17	14	NA	LBD21.31R	16858895	16861452	+		inside	862
27	23	NA	LBD21.31R	16933171	16936868	-		upstream	-4696
17	13	NA	LBD21.31R	17258225	17264952	+		upstream	-9657
17	15	NA	LBD21.31R	17974662	17976325	+		downstrea	1702
27	25	NA	LBD21.31R	18049998	18051851	+		inside	18
18	16	NA	LBD21.31R	274497	275360	+		downstrea	4848
22	19	NA	LBD21.31R	428272	430274	+		inside	1461
19	17	NA	LBD21.31R	773291	773710	+		downstrea	986
23	21	NA	LBD21.31R	878985	880332	+		upstream	-541
24	21	NA	LBD21.31R	1659514	1661849	-		upstream	-1090
21	19	NA	LBD21.31R	1698979	1701858	-		inside	1018
20	17	NA	LBD21.31R	1772665	1773882	+		upstream	-2448
16	14	NA	LBD21.31R	1790897	1791558	-		downstrea	4059
19	17	NA	LBD21.31R	1875251	1878254	-		inside	2527
19	17	NA	LBD21.31R	2051060	2056456	+		inside	85
18	16	NA	LBD21.31R	2140833	2141899	+		downstrea	1175
20	17	NA	LBD21.31R	3342885	3345628	+		downstrea	3566
17	13	NA	LBD21.31R	3629109	3634234	+		inside	4183
17	14	NA	LBD21.31R	3680805	3681724	+		upstream	-1385
19	15	NA	LBD21.31R	3756392	3763955	-		upstream	-4000
28	25	NA	LBD21.31R	3780252	3780497	-		upstream	-4907
29	24	NA	LBD21.31R	3851240	3851963	+		upstream	-4256
25	22	NA	LBD21.31R	3881692	3889376	-		upstream	-1868
18	15	NA	LBD21.31R	4986633	4992453	-		inside	3218
17	14	NA	LBD21.31R	5408743	5411011	+		upstream	-3498
18	16	NA	LBD21.31R	5435316	5440099	+		inside	4275
18	17	NA	LBD21.31R	5531491	5533297	-		inside	401
24	20	NA	LBD21.31R	6257592	6258115	-		upstream	-7383
18	16	NA	LBD21.31R	6940150	6960376	+		upstream	-48492
17	15	NA	LBD21.31R	7039338	7039887	+		upstream	-1269
71	69	NA	LBD21.31R	7087527	7087980	+		inside	34
23	20	NA	LBD21.31R	7540873	7541856	-		downstrea	54181
34	31	NA	LBD21.31R	8364171	8366711	+		downstrea	2833
25	22	NA	LBD21.31R	8466107	8472367	+		downstrea	15430
24	21	NA	LBD21.31R	8499578	8501428	+		downstrea	3326
23	20	NA	LBD21.31R	8820074	8821825	-		upstream	-23377
19	17	NA	LBD21.31R	9075043	9077053	-		downstrea	3735
17	15	NA	LBD21.31R	9214832	9218388	-		inside	2414
17	15	NA	LBD21.31R	9823081	9826808	+		inside	2660
19	16	NA	LBD21.31R	9826733	9830338	-		upstream	-1987
20	16	NA	LBD21.31R	9855190	9858922	+		upstream	-4546
19	17	NA	LBD21.31R	9900642	9903747	+		inside	1168
20	16	NA	LBD21.31R	9970242	9972070	+		upstream	-2782
18	16	NA	LBD21.31R	10012685	10014370	+		inside	1107
17	13	NA	LBD21.31R	10145596	10145850	+		upstream	-5174
26	22	NA	LBD21.31R	10411438	10416356	-		upstream	-4768
21	19	NA	LBD21.31R	10488772	10492999	-		inside	2671
29	25	NA	LBD21.31R	10523812	10526089	+		downstrea	8181
33	31	NA	LBD21.31R	10796766	10799160	+		upstream	-19183
17	15	NA	LBD21.31R	10954107	10959386	+		inside	2112
26	23	NA	LBD21.31R	11050489	11051138	-		downstrea	4688
35	30	NA	LBD21.31R	11137722	11139660	+		downstrea	6643

17	15	NA	LBD21.31R	11244930	11247010	-	inside	1983
23	20	NA	LBD21.31R	11356226	11359696	+	upstream	-7049
20	17	NA	LBD21.31R	11448703	11461097	+	inside	4908
28	26	NA	LBD21.31R	11588198	11599425	-	inside	10368
19	17	NA	LBD21.31R	11682812	11684817	+	downstrea	2046
18	16	NA	LBD21.31R	12015845	12017020	+	upstream	-884
33	31	NA	LBD21.31R	12311205	12313557	-	inside	769
18	15	NA	LBD21.31R	12355907	12357365	-	upstream	-2591
20	18	NA	LBD21.31R	12371433	12376015	-	inside	1298
19	17	NA	LBD21.31R	12421577	12421883	+	inside	33
20	18	NA	LBD21.31R	12553770	12557943	+	inside	1777
18	14	NA	LBD21.31R	12766213	12768115	-	upstream	-4637
30	28	NA	LBD21.31R	12868554	12869907	-	upstream	-2764
20	18	NA	LBD21.31R	12894761	12896793	-	upstream	-675
24	21	NA	LBD21.31R	13018998	13019841	+	upstream	-3542
17	16	NA	LBD21.31R	13055362	13057881	-	inside	2038
18	14	NA	LBD21.31R	13079329	13079566	+	overlapEn	33
18	16	NA	LBD21.31R	13223079	13224502	+	inside	1018
17	14	NA	LBD21.31R	13277604	13278577	+	downstrea	2894
24	21	NA	LBD21.31R	13703467	13712962	-	inside	5066
19	15	NA	LBD21.31R	13722549	13738643	-	upstream	-22890
19	17	NA	LBD21.31R	13976282	13979025	+	inside	1571
16	12	NA	LBD21.31R	14099535	14100465	-	upstream	-2854
22	20	NA	LBD21.31R	14117032	14119973	+	downstrea	3114
16	14	NA	LBD21.31R	14196754	14206880	+	inside	2080
19	16	NA	LBD21.31R	14244690	14245079	-	overlapSt	62
21	19	NA	LBD21.31R	14281387	14285743	+	upstream	-1623
18	16	NA	LBD21.31R	14354997	14358305	-	inside	2139
16	12	NA	LBD21.31R	14376964	14378396	+	upstream	-488
19	17	NA	LBD21.31R	14456179	14460666	+	inside	614
17	15	NA	LBD21.31R	14735670	14736753	+	downstrea	1182
19	16	NA	LBD21.31R	14777432	14782106	-	inside	1724
16	14	NA	LBD21.31R	14961839	14964665	+	downstrea	2936
19	17	NA	LBD21.31R	14970440	14975257	-	inside	2188
17	14	NA	LBD21.31R	15036990	15038916	-	downstrea	5113
20	18	NA	LBD21.31R	15087035	15089363	-	inside	2174
19	17	NA	LBD21.31R	15115381	15115863	+	downstrea	881
24	22	NA	LBD21.31R	15120813	15124437	-	inside	665
34	32	NA	LBD21.31R	15170317	15170755	+	upstream	-1737
19	16	NA	LBD21.31R	15237741	15239875	-	overlapSt	175
23	19	NA	LBD21.31R	15373698	15379797	-	upstream	-578
22	19	NA	LBD21.31R	15463453	15464495	-	overlapEn	1044
17	15	NA	LBD21.31R	15582384	15584094	-	inside	1541
18	16	NA	LBD21.31R	15615822	15627778	-	inside	3696
18	16	NA	LBD21.31R	15704881	15708099	-	inside	1943
17	14	NA	LBD21.31R	592545	595758	+	upstream	-5622
21	19	NA	LBD21.31R	634239	637149	+	inside	1142
19	17	NA	LBD21.31R	642518	646495	-	inside	1011
18	15	NA	LBD21.31R	773339	783010	-	upstream	-9698
27	23	NA	LBD21.31R	1135684	1136628	-	upstream	-2572
18	16	NA	LBD21.31R	1436400	1439816	+	inside	1142
36	35	NA	LBD21.31R	1673482	1677412	+	inside	2817
20	17	NA	LBD21.31R	1714158	1716041	+	upstream	-13893
20	18	NA	LBD21.31R	1741029	1744938	+	upstream	-2567

21	18	NA	LBD21.31R	2467474	2472895	-	inside	2881
23	21	NA	LBD21.31R	2505101	2506028	+	upstream	-4114
17	14	NA	LBD21.31R	2902894	2903814	+	downstrea	1699
21	19	NA	LBD21.31R	3353107	3353361	+	downstrea	7361
27	24	NA	LBD21.31R	3621041	3622906	-	inside	1506
21	18	NA	LBD21.31R	3628925	3631600	-	upstream	-1323
18	16	NA	LBD21.31R	3771491	3782070	+	inside	8240
28	25	NA	LBD21.31R	3991611	3996963	+	inside	763
18	16	NA	LBD21.31R	4274467	4280930	+	inside	2671
18	16	NA	LBD21.31R	4567214	4567826	+	overlapEn	364
64	62	NA	LBD21.31R	5311388	5311926	-	downstrea	14972
20	17	NA	LBD21.31R	5600652	5612865	-	inside	6889
20	17	NA	LBD21.31R	5970580	5972265	+	upstream	-2958
21	19	NA	LBD21.31R	5972786	5973658	+	downstrea	1087
40	39	NA	LBD21.31R	6402922	6404074	+	upstream	-641
67	65	NA	LBD21.31R	6404076	6408104	+	overlapSt	-158
30	29	NA	LBD21.31R	6408512	6409298	+	includeFe	-75
30	29	NA	LBD21.31R	6410411	6410767	+	overlapSt	-362
43	42	NA	LBD21.31R	6413755	6414887	+	inside	517
18	16	NA	LBD21.31R	6539055	6540229	-	inside	877
22	20	NA	LBD21.31R	8382096	8385123	-	upstream	-11984
17	14	NA	LBD21.31R	8531878	8557929	-	inside	4079
18	16	NA	LBD21.31R	8652583	8677213	+	inside	24049
33	32	NA	LBD21.31R	9585458	9591300	-	upstream	-701
43	42	NA	LBD21.31R	9857154	9857592	-	inside	426
20	17	NA	LBD21.31R	10263286	10266497	-	inside	2809
22	19	NA	LBD21.31R	10293910	10304554	-	upstream	-3713
25	21	NA	LBD21.31R	10313887	10314624	+	downstrea	7642
48	45	NA	LBD21.31R	10736488	10737257	-	downstrea	1091
18	15	NA	LBD21.31R	10870739	10874037	-	inside	2353
18	14	NA	LBD21.31R	11147296	11150772	+	overlapEn	3268
19	15	NA	LBD21.31R	11160987	11170428	-	upstream	-19841
19	17	NA	LBD21.31R	11656284	11659564	+	inside	409
16	14	NA	LBD21.31R	11676610	11677298	+	overlapEn	562
17	14	NA	LBD21.31R	12162080	12164957	-	inside	1295
20	18	NA	LBD21.31R	12365534	12368041	+	inside	506
19	17	NA	LBD21.31R	12673696	12675672	-	upstream	-587
17	15	NA	LBD21.31R	12941227	12944199	+	inside	823
20	18	NA	LBD21.31R	13248572	13251015	+	downstrea	5226
24	22	NA	LBD21.31R	13611002	13611721	-	upstream	-2503
19	17	NA	LBD21.31R	13698237	13702803	+	inside	802
51	49	NA	LBD21.31R	13971000	13976252	+	upstream	-3376
23	20	NA	LBD21.31R	14038902	14040718	-	upstream	-950
17	14	NA	LBD21.31R	14100565	14110153	-	inside	3352
38	36	NA	LBD21.31R	14518609	14522174	-	upstream	-693
54	53	NA	LBD21.31R	14660556	14660867	+	includeFe	-4
25	24	NA	LBD21.31R	14661052	14661624	+	downstrea	932
81	79	NA	LBD21.31R	14663541	14663858	+	upstream	-639
34	33	NA	LBD21.31R	14665244	14665681	+	overlapEn	20
27	26	NA	LBD21.31R	14666884	14667419	-	overlapEn	1027
21	19	NA	LBD21.31R	14683663	14685457	-	inside	855
61	59	NA	LBD21.31R	14752339	14752989	-	overlapSt	478
74	72	NA	LBD21.31R	14753634	14754182	-	inside	343
43	42	NA	LBD21.31R	14754882	14755349	-	includeFe	500

36	35	NA	LBD21.31R	14755958	14756298	-	overlapSt	100
73	71	NA	LBD21.31R	14757219	14757726	+	downstrea	734
91	89	NA	LBD21.31R	14765441	14765638	-	upstream	-1888
43	42	NA	LBD21.31R	14771293	14771695	-	downstrea	1616
50	49	NA	LBD21.31R	14772455	14773204	+	inside	93
43	42	NA	LBD21.31R	14775554	14776118	-	inside	561
59	56	NA	LBD21.31R	14779861	14780816	-	downstrea	1703
122	119	NA	LBD21.31R	14783992	14784405	-	upstream	-854
27	26	NA	LBD21.31R	14788312	14789160	+	overlapEn	296
60	58	NA	LBD21.31R	14790129	14790383	-	upstream	-503
28	27	NA	LBD21.31R	14791829	14792637	+	inside	267
31	30	NA	LBD21.31R	14794072	14795129	+	includeFe	-399
45	43	NA	LBD21.31R	14814563	14814898	+	upstream	-453
92	90	NA	LBD21.31R	14825811	14825999	+	downstrea	815
68	66	NA	LBD21.31R	14829782	14829886	-	downstrea	1355
86	84	NA	LBD21.31R	14830570	14831157	+	downstrea	1363
85	83	NA	LBD21.31R	14835523	14835765	+	downstrea	526
62	60	NA	LBD21.31R	14836710	14837115	-	overlapEn	466
61	59	NA	LBD21.31R	14837889	14838632	+	downstrea	1032
44	43	NA	LBD21.31R	14844509	14845329	-	downstrea	3076
38	37	NA	LBD21.31R	14845563	14846078	+	downstrea	1766
30	29	NA	LBD21.31R	14849394	14849797	+	includeFe	-279
54	52	NA	LBD21.31R	14851611	14852408	+	inside	346
47	46	NA	LBD21.31R	14853384	14854012	+	inside	151
35	34	NA	LBD21.31R	14865069	14866243	+	inside	291
54	52	NA	LBD21.31R	14867350	14867454	+	upstream	-1020
34	33	NA	LBD21.31R	14870394	14871677	+	upstream	-919
101	99	NA	LBD21.31R	14877522	14878292	+	downstrea	1200
45	44	NA	LBD21.31R	14880122	14880328	-	includeFe	652
101	99	NA	LBD21.31R	14881679	14881918	+	overlapEn	70
85	82	NA	LBD21.31R	14882770	14884113	+	inside	325
74	72	NA	LBD21.31R	14894928	14896038	+	downstrea	1204
39	37	NA	LBD21.31R	14901898	14907618	+	inside	5457
20	17	NA	LBD21.31R	14996475	15001272	+	upstream	-8927
19	17	NA	LBD21.31R	15385225	15389973	+	overlapEn	4419
18	16	NA	LBD21.31R	15447641	15452128	-	inside	750
18	14	NA	LBD21.31R	15639115	15641031	-	upstream	-2119
17	14	NA	LBD21.31R	15656674	15657674	+	downstrea	1239
24	21	NA	LBD21.31R	15722546	15724488	+	upstream	-4800
37	35	NA	LBD21.31R	15832819	15835201	+	downstrea	3705
17	15	NA	LBD21.31R	15901561	15904411	-	inside	1233
18	16	NA	LBD21.31R	16012591	16020092	-	downstrea	7887
23	20	NA	LBD21.31R	16066967	16067796	+	downstrea	2843
28	23	NA	LBD21.31R	16162975	16169041	+	inside	1356
18	15	NA	LBD21.31R	731206	735001	-	upstream	-917
24	20	NA	LBD21.31R	1313962	1316043	-	upstream	-4578
35	30	NA	LBD21.31R	1489482	1493387	-	inside	1236
19	17	NA	LBD21.31R	1629064	1635605	-	upstream	-1948
18	15	NA	LBD21.31R	2169470	2171490	+	downstrea	2665
19	16	NA	LBD21.31R	3318499	3326082	-	upstream	-5328
46	43	NA	LBD21.31R	3435300	3440047	-	upstream	-1460
18	15	NA	LBD21.31R	3741950	3742335	+	downstrea	2622
20	18	NA	LBD21.31R	4119030	4123682	+	inside	1043
26	24	NA	LBD21.31R	4238803	4238982	+	downstrea	1380

30	27	NA	LBD21.31R	4245031	4246350	+	downstrea	2596
28	26	NA	LBD21.31R	4255330	4258195	+	inside	886
24	22	NA	LBD21.31R	4261057	4261458	+	downstrea	651
17	14	NA	LBD21.31R	4506551	4508041	-	downstrea	3682
18	15	NA	LBD21.31R	4648125	4651889	-	upstream	-2513
25	23	NA	LBD21.31R	4749118	4751720	-	inside	1812
23	19	NA	LBD21.31R	4770571	4771568	+	downstrea	1397
19	16	NA	LBD21.31R	5351230	5353789	-	upstream	-3118
18	16	NA	LBD21.31R	5536840	5541062	+	inside	2720
17	15	NA	LBD21.31R	6226331	6230401	-	upstream	-1904
28	25	NA	LBD21.31R	6385919	6393427	+	inside	578
35	33	NA	LBD21.31R	6429060	6430243	-	upstream	-941
17	15	NA	LBD21.31R	6547245	6549845	+	inside	1938
18	16	NA	LBD21.31R	6588710	6598177	-	upstream	-454
37	35	NA	LBD21.31R	6968454	6973051	-	upstream	-2105
19	16	NA	LBD21.31R	6980125	6985518	-	inside	2494
31	26	NA	LBD21.31R	7118925	7121224	+	upstream	-1089
17	14	NA	LBD21.31R	7287631	7287810	-	upstream	-4075
17	15	NA	LBD21.31R	7986961	7988446	-	inside	1378
18	16	NA	LBD21.31R	8140603	8148322	-	inside	1187
20	17	NA	LBD21.31R	8226544	8228993	-	upstream	-3534
19	16	NA	LBD21.31R	9199617	9200053	-	upstream	-1041
30	28	NA	LBD21.31R	9349076	9356575	+	upstream	-3941
20	16	NA	LBD21.31R	9716465	9717471	-	overlapEn	1095
16	14	NA	LBD21.31R	10020843	10028307	+	inside	1589
42	38	NA	LBD21.31R	10403873	10405807	-	upstream	-134
18	15	NA	LBD21.31R	10416007	10418905	-	inside	298
18	15	NA	LBD21.31R	10633908	10634354	+	downstrea	1189
19	16	NA	LBD21.31R	11047549	11051847	+	upstream	-1444
40	38	NA	LBD21.31R	11073156	11075291	-	upstream	-1908
20	17	NA	LBD21.31R	11132347	11136217	+	overlapSt	-113
17	15	NA	LBD21.31R	11498179	11507177	-	inside	3275
16	12	NA	LBD21.31R	12213368	12214398	+	upstream	-1089
26	22	NA	LBD21.31R	12397621	12399479	+	upstream	-495
22	19	NA	LBD21.31R	12601305	12605522	-	inside	4013
16	14	NA	LBD21.31R	12725170	12731865	+	inside	3032
24	20	NA	LBD21.31R	12925765	12927225	+	downstrea	20533
18	15	NA	LBD21.31R	13141970	13145599	-	inside	354
20	18	NA	LBD21.31R	13562156	13564642	-	inside	1149
48	44	NA	LBD21.31R	13667462	13667732	+	downstrea	657
18	14	NA	LBD21.31R	14100657	14103545	+	inside	1239
24	21	NA	LBD21.31R	14563846	14564802	-	upstream	-1207
25	22	NA	LBD21.31R	14599471	14600126	+	inside	253
17	14	NA	LBD21.31R	14743200	14748976	-	upstream	-26870
17	13	NA	LBD21.31R	14841817	14848896	+	upstream	-4610
19	15	NA	LBD21.31R	15176521	15179747	-	upstream	-156
56	54	NA	LBD21.31R	15925039	15932369	-	upstream	-1124
38	37	NA	LBD21.31R	16505398	16505793	-	upstream	-15231
17	15	NA	LBD21.31R	16651127	16651783	+	upstream	-1605
17	16	NA	LBD21.31R	16984035	16984962	-	downstrea	6064
17	16	NA	LBD21.31R	16984821	16985340	-	upstream	-114
40	38	NA	LBD21.31R	17226201	17227664	+	upstream	-10804
19	16	NA	LBD21.31R	17264422	17271626	+	upstream	-3707
18	15	NA	LBD21.31R	17374404	17374625	-	downstrea	12099

16	12	NA	LBD21.31R	17503418	17505261	-	downstrea	4270
23	19	NA	LBD21.31R	17612333	17615512	-	upstream	-4480
20	16	NA	LBD21.31R	17669483	17675274	+	upstream	-20955
29	25	NA	LBD21.31R	17831231	17845254	-	downstrea	22471
32	27	NA	LBD21.31R	17857165	17864759	-	upstream	-14368
18	15	NA	LBD21.31R	17946056	17948099	+	upstream	-3251
25	23	NA	LBD21.31R	18139525	18146950	-	downstrea	11254
35	32	NA	LBD21.31R	18318753	18326192	-	upstream	-6825
17	15	NA	LBD21.31R	18419460	18428607	+	inside	161
18	15	NA	LBD21.31R	458876	461241	+	downstrea	2997
17	15	NA	LBD21.31R	601409	603356	+	inside	652
17	14	NA	LBD21.31R	948915	952911	+	upstream	-7532
27	24	NA	LBD21.31R	1858280	1862197	+	upstream	-3716
17	15	NA	LBD21.31R	1899112	1904272	+	inside	578
18	16	NA	LBD21.31R	2345768	2353767	+	inside	122
18	15	NA	LBD21.31R	2637644	2638216	-	downstrea	2676
19	17	NA	LBD21.31R	2932682	2938680	-	inside	1011
17	15	NA	LBD21.31R	3344864	3346133	-	overlapEn	1274
17	14	NA	LBD21.31R	3477396	3481333	+	downstrea	12297
19	16	NA	LBD21.31R	3510981	3513035	-	downstrea	12287
18	16	NA	LBD21.31R	3695168	3696939	+	upstream	-1055
17	14	NA	LBD21.31R	3731254	3732570	+	inside	457
23	20	NA	LBD21.31R	4011094	4014183	+	upstream	-2068
17	15	NA	LBD21.31R	4073780	4080814	-	upstream	-6815
22	20	NA	LBD21.31R	4095980	4102909	+	inside	3001
30	25	NA	LBD21.31R	4113946	4119988	+	upstream	-4071
18	16	NA	LBD21.31R	4399157	4405464	+	inside	2832
31	30	NA	LBD21.31R	4554427	4560533	-	inside	2496
65	60	NA	LBD21.31R	4970325	4977849	+	upstream	-2435
38	33	NA	LBD21.31R	5169004	5170626	+	upstream	-7448
22	19	NA	LBD21.31R	5373276	5381768	-	upstream	-30116
19	15	NA	LBD21.31R	5603569	5603877	+	upstream	-18586
16	13	NA	LBD21.31R	5635620	5667458	-	inside	4554
26	24	NA	LBD21.31R	6288546	6289859	-	inside	230
50	48	NA	LBD21.31R	6322447	6322818	+	overlapEn	34
62	60	NA	LBD21.31R	6328465	6329003	+	upstream	-1951
28	26	NA	LBD21.31R	6406439	6408531	+	downstrea	2762
18	16	NA	LBD21.31R	6581611	6581823	-	upstream	-6846
26	24	NA	LBD21.31R	6581703	6587085	+	upstream	-10123
17	15	NA	LBD21.31R	6631345	6632413	-	upstream	-49765
22	19	NA	LBD21.31R	6956049	6957260	-	upstream	-12289
16	12	NA	LBD21.31R	7437985	7438842	-	upstream	-5473
52	50	NA	LBD21.31R	7977017	7979624	-	inside	1219
23	21	NA	LBD21.31R	8042378	8044079	+	overlapSt	-399
20	17	NA	LBD21.31R	8381611	8388412	-	overlapSt	93
19	16	NA	LBD21.31R	8578922	8580741	+	downstrea	4486
31	28	NA	LBD21.31R	8886714	8888635	-	inside	1518
20	17	NA	LBD21.31R	9030920	9032223	+	inside	112
18	15	NA	LBD21.31R	9283824	9286967	+	downstrea	5317
25	21	NA	LBD21.31R	9578198	9580711	+	upstream	-3795
50	48	NA	LBD21.31R	10235060	10235369	+	inside	25
17	14	NA	LBD21.31R	11320982	11321489	-	inside	358
17	14	NA	LBD21.31R	11994376	11996292	+	inside	1404
18	15	NA	LBD21.31R	12012072	12013730	-	upstream	-751

17	16	NA	LBD21.31R	12108018	12114460	-	inside	4031
21	17	NA	LBD21.31R	12317986	12322365	+	upstream	-4275
30	25	NA	LBD21.31R	13067169	13071253	-	inside	2354
18	16	NA	LBD21.31R	13290801	13293848	-	overlapSt	152
33	29	NA	LBD21.31R	13310810	13312539	-	downstrea	5168
20	18	NA	LBD21.31R	13330338	13331562	+	downstrea	1618
21	19	NA	LBD21.31R	13653206	13657755	+	inside	445
39	35	NA	LBD21.31R	13712792	13715704	+	upstream	-336
26	23	NA	LBD21.31R	13741366	13744927	-	overlapEn	3752
25	22	NA	LBD21.31R	14297144	14299677	+	downstrea	3606
19	17	NA	LBD21.31R	14911353	14916935	-	upstream	-1294
25	22	NA	LBD21.31R	14975769	14979847	-	inside	2748
17	15	NA	LBD21.31R	15243480	15246532	+	upstream	-1961
18	16	NA	LBD21.31R	528688	532529	+	inside	1377
27	25	NA	LBD21.31R	553726	558012	-	upstream	-2803
25	21	NA	LBD21.31R	626427	626889	-	downstrea	1805
24	22	NA	LBD21.31R	813721	817048	-	upstream	-828
23	21	NA	LBD21.31R	904073	905624	+	inside	1272
18	16	NA	LBD21.31R	1443146	1447104	+	inside	615
17	15	NA	LBD21.31R	1447433	1448510	+	upstream	-1399
22	20	NA	LBD21.31R	1624024	1628651	-	inside	2164
21	18	NA	LBD21.31R	1868366	1869367	-	downstrea	1897
18	15	NA	LBD21.31R	2517734	2518480	-	downstrea	7685
17	15	NA	LBD21.31R	2884652	2885784	-	upstream	-47
20	16	NA	LBD21.31R	2901630	2902712	-	upstream	-3415
17	13	NA	LBD21.31R	3015792	3016055	+	overlapSt	-161
18	16	NA	LBD21.31R	3196568	3198829	-	upstream	-1951
16	14	NA	LBD21.31R	3326362	3331791	+	inside	1504
26	22	NA	LBD21.31R	3412830	3416331	+	upstream	-6752
19	16	NA	LBD21.31R	3541745	3542526	+	overlapEn	685
27	25	NA	LBD21.31R	3614176	3617007	+	downstrea	4064
17	15	NA	LBD21.31R	3795851	3796694	-	inside	685
19	17	NA	LBD21.31R	3871632	3876702	-	inside	3854
20	17	NA	LBD21.31R	3938815	3940594	+	upstream	-2197
18	16	NA	LBD21.31R	4115461	4120192	-	inside	328
24	22	NA	LBD21.31R	4266555	4267758	-	inside	700
25	23	NA	LBD21.31R	4310269	4311084	-	upstream	-4501
19	16	NA	LBD21.31R	4391378	4394340	-	inside	2859
26	22	NA	LBD21.31R	4953185	4960792	+	upstream	-4960
16	12	NA	LBD21.31R	5045014	5046327	+	upstream	-4734
19	17	NA	LBD21.31R	5523869	5527970	-	inside	1114
32	27	NA	LBD21.31R	5842560	5843301	+	downstrea	994
17	15	NA	LBD21.31R	6061213	6064507	-	inside	865
39	36	NA	LBD21.31R	6097845	6099951	+	inside	1046
21	19	NA	LBD21.31R	6579485	6580823	+	inside	299
17	15	NA	LBD21.31R	6677891	6681323	+	inside	853
32	30	NA	LBD21.31R	7275771	7277504	-	inside	1680
20	17	NA	LBD21.31R	7298514	7298877	+	upstream	-9063
32	30	NA	LBD21.31R	7710855	7711361	+	upstream	-21034
18	15	NA	LBD21.31R	7796125	7796896	-	upstream	-8608
24	20	NA	LBD21.31R	7990864	7998006	-	upstream	-12651
21	19	NA	LBD21.31R	8101978	8102394	+	downstrea	28707
45	43	NA	LBD21.31R	8218191	8219153	+	inside	71
25	21	NA	LBD21.31R	8468303	8471131	+	upstream	-12570

28	23	NA	LBD21.31R	10360586	10364403	-	upstream	-3823
18	16	NA	LBD21.31R	10427189	10427585	-	downstrea	2700
32	30	NA	LBD21.31R	11057082	11057586	+	downstrea	4706
24	22	NA	LBD21.31R	11100066	11100463	-	downstrea	4944
19	15	NA	LBD21.31R	11212293	11213501	+	downstrea	2674
20	18	NA	LBD21.31R	12145528	12148381	+	overlapEn	2639
48	45	NA	LBD21.31R	12343379	12350042	-	inside	659
21	19	NA	LBD21.31R	12539743	12543487	+	inside	1275
21	18	NA	LBD21.31R	12596463	12599352	-	upstream	-2870
19	17	NA	LBD21.31R	13016462	13017756	+	upstream	-2187
17	13	NA	LBD21.31R	13271651	13271820	-	downstrea	1434
38	36	NA	LBD21.31R	13361396	13364190	+	overlapEn	2585
19	17	NA	LBD21.31R	13760476	13762290	+	downstrea	2625
19	16	NA	LBD21.31R	13767533	13768493	-	upstream	-2846
20	18	NA	LBD21.31R	13840606	13844757	+	inside	2179
17	14	NA	LBD21.31R	13989680	13990909	-	upstream	-1717
16	14	NA	LBD21.31R	14173867	14177417	+	inside	1313
18	16	NA	LBD21.31R	14185523	14187522	+	inside	810
22	18	NA	LBD21.31R	362085	363948	-	inside	633
17	13	NA	LBD21.31R	476715	481503	+	upstream	-3356
17	14	NA	LBD21.31R	583179	584266	+	downstrea	4186
21	20	NA	LBD21.31R	709834	712800	+	upstream	-12233
20	17	NA	LBD21.31R	804971	809233	+	upstream	-614
17	14	NA	LBD21.31R	947026	949342	+	inside	233
27	24	NA	LBD21.31R	1821367	1822834	-	inside	1173
19	17	NA	LBD21.31R	2038429	2045055	+	inside	950
37	35	NA	LBD21.31R	2186360	2187873	+	upstream	-20275
18	16	NA	LBD21.31R	2256891	2262332	+	inside	4703
20	18	NA	LBD21.31R	2353070	2353586	+	downstrea	2574
18	16	NA	LBD21.31R	2578793	2582511	+	inside	559
18	16	NA	LBD21.31R	2668624	2669811	+	upstream	-3575
24	20	NA	LBD21.31R	3583593	3583901	-	downstrea	1770
17	15	NA	LBD21.31R	3592006	3593938	+	downstrea	4163
25	21	NA	LBD21.31R	3699486	3710047	-	inside	3299
19	17	NA	LBD21.31R	4044469	4048853	-	inside	745
21	19	NA	LBD21.31R	4589135	4589335	+	downstrea	2580
17	14	NA	LBD21.31R	5642080	5644665	-	upstream	-9837
22	19	NA	LBD21.31R	5708953	5710046	+	upstream	-1097
46	44	NA	LBD21.31R	5860500	5863375	-	upstream	-5250
58	56	NA	LBD21.31R	5940893	5941428	+	inside	21
20	18	NA	LBD21.31R	6937683	6944376	+	inside	2148
87	83	NA	LBD21.31R	7261738	7264096	+	downstrea	38277
54	52	NA	LBD21.31R	7364780	7365135	-	downstrea	6312
36	33	NA	LBD21.31R	7467160	7471594	-	upstream	-38714
23	20	NA	LBD21.31R	8296712	8297045	+	upstream	-3668
34	32	NA	LBD21.31R	8764503	8766101	-	upstream	-2971
17	15	NA	LBD21.31R	9599828	9601523	-	downstrea	3732
17	14	NA	LBD21.31R	9635769	9638227	+	upstream	-1285
22	18	NA	LBD21.31R	9813697	9813831	+	upstream	-1242
55	53	NA	LBD21.31R	10065285	10065639	+	upstream	-10217
17	13	NA	LBD21.31R	10330119	10331759	-	upstream	-3766
17	14	NA	LBD21.31R	10846200	10853416	+	inside	359
21	18	NA	LBD21.31R	10947583	10953970	+	upstream	-9806
26	22	NA	LBD21.31R	11031741	11032041	+	upstream	-627

19	17	NA	LBD21.31R	11500621	11508966	-	inside	960
23	20	NA	LBD21.31R	11666996	11671264	+	upstream	-15194
18	15	NA	LBD21.31R	11725841	11733082	+	upstream	-5416
17	15	NA	LBD21.31R	11998047	11999028	+	upstream	-2545
21	18	NA	LBD21.31R	12178033	12179081	+	downstrea	2290
17	14	NA	LBD21.31R	12570826	12571681	-	overlapSt	153
17	14	NA	LBD21.31R	12620436	12626207	+	inside	1723
18	14	NA	LBD21.31R	12855403	12859226	-	inside	2350
20	18	NA	LBD21.31R	13012869	13015325	+	upstream	-8077
24	21	NA	LBD21.31R	13150818	13156037	+	upstream	-4687
18	15	NA	LBD21.31R	13184918	13185627	+	overlapSt	-133
17	15	NA	LBD21.31R	13334516	13334953	+	overlapSt	-181
30	28	NA	LBD21.31R	13528321	13532208	-	upstream	-1452
18	15	NA	LBD21.31R	14201130	14201727	+	inside	169
17	15	NA	LBD21.31R	14235060	14237031	+	upstream	-3629
17	15	NA	LBD21.31R	14239005	14241188	+	inside	1925
18	16	NA	LBD21.31R	14441145	14449013	-	inside	2633
18	14	NA	LBD21.31R	14667726	14673252	-	upstream	-118
17	14	NA	LBD21.31R	14723580	14726534	+	inside	416
20	17	NA	LBD21.31R	14741875	14746528	-	inside	2818
17	15	NA	LBD21.31R	14748601	14752776	-	upstream	-117
49	47	NA	LBD21.31R	14835641	14836690	-	upstream	-102
31	29	NA	LBD21.31R	14838177	14839672	+	upstream	-728
21	20	NA	LBD21.31R	15112186	15115017	-	downstrea	13530
17	15	NA	LBD21.31R	15517239	15520067	+	inside	1536
17	14	NA	LBD21.31R	15808899	15814020	+	inside	545
19	17	NA	LBD21.31R	15917789	15919258	-	upstream	-1011
17	15	NA	LBD21.31R	766518	772371	-	overlapSt	13
17	13	NA	LBD21.31R	836540	842809	-	inside	3335
17	15	NA	LBD21.31R	1274099	1276741	-	upstream	-2066
19	15	NA	LBD21.31R	1491298	1492943	-	upstream	-6958
18	15	NA	LBD21.31R	1743742	1745095	-	upstream	-3484
17	15	NA	LBD21.31R	2319954	2322915	-	overlapSt	253
20	17	NA	LBD21.31R	2345287	2346896	-	downstrea	3002
23	20	NA	LBD21.31R	2355148	2359228	+	inside	326
31	30	NA	LBD21.31R	2436803	2437920	+	inside	532
18	15	NA	LBD21.31R	2680940	2682556	-	upstream	-1710
17	14	NA	LBD21.31R	3014831	3018941	+	upstream	-592
53	51	NA	LBD21.31R	3119582	3122945	+	upstream	-11915
35	30	NA	LBD21.31R	3234742	3238131	+	upstream	-4905
92	90	NA	LBD21.31R	3799400	3799737	+	inside	29
19	15	NA	LBD21.31R	4017910	4024850	+	upstream	-1785
22	21	NA	LBD21.31R	4061102	4061935	-	inside	593
22	19	NA	LBD21.31R	4122359	4129934	+	upstream	-10005
29	26	NA	LBD21.31R	4159566	4159991	+	downstrea	963
21	18	NA	LBD21.31R	4416611	4417856	-	upstream	-17446
23	21	NA	LBD21.31R	4676602	4678662	-	upstream	-12236
34	32	NA	LBD21.31R	5006621	5007839	-	upstream	-23849
89	86	NA	LBD21.31R	5252831	5256854	+	upstream	-10454
26	24	NA	LBD21.31R	5636863	5637371	-	downstrea	803
21	18	NA	LBD21.31R	5764955	5771073	-	inside	821
18	15	NA	LBD21.31R	6209246	6209788	-	downstrea	2093
18	15	NA	LBD21.31R	6288391	6290320	+	inside	1139
17	14	NA	LBD21.31R	6365527	6368967	-	upstream	-31381

70	65	NA	LBD21.31R	6532988	6537493	+		downstrea	84851
18	17	NA	LBD21.31R	7374464	7374985	+		upstream	-18432
18	17	NA	LBD21.31R	7766617	7770793	+		inside	1558
18	15	NA	LBD21.31R	8133687	8142269	-		downstrea	11574
36	34	NA	LBD21.31R	9346400	9346579	+		upstream	-16542
17	15	NA	LBD21.31R	10090918	10098117	+		inside	139
17	14	NA	LBD21.31R	10187948	10189829	+		upstream	-29725
19	17	NA	LBD21.31R	10518552	10522822	-		inside	1362
18	16	NA	LBD21.31R	10732971	10739712	+		inside	1393
20	18	NA	LBD21.31R	11165781	11169792	-		inside	558
26	23	NA	LBD21.31R	11640875	11642874	-		downstrea	2373
57	52	NA	LBD21.31R	11697912	11699851	-		upstream	-6627
24	21	NA	LBD21.31R	11813066	11827727	+		upstream	-808
22	19	NA	LBD21.31R	12568154	12573071	+		upstream	-8804
18	16	NA	LBD21.31R	12643275	12644045	-		downstrea	1144
22	19	NA	LBD21.31R	12799233	12802179	-		downstrea	4768
18	14	NA	LBD21.31R	13029141	13030024	-		upstream	-3665
20	17	NA	LBD21.31R	13210914	13212861	+		downstrea	4135
18	15	NA	LBD21.31R	13654861	13655379	+		downstrea	7437
27	25	NA	LBD21.31R	13775241	13779044	+		inside	2727
19	17	NA	LBD21.31R	13916467	13916964	+		overlapSt	-385
18	15	NA	LBD21.31R	15180498	15182662	-		inside	1160
18	14	NA	LBD21.31R	15515402	15516727	+		downstrea	1953
21	19	NA	LBD21.31R	15590333	15591232	-		upstream	-321
21	19	NA	LBD21.31R	15619914	15624708	+		inside	427
19	16	NA	LBD21.31R	15734482	15740935	-		inside	1590
26	23	NA	LBD21.31R	15991778	15997918	+		inside	4009
35	31	NA	LBD21.31R	16893804	16896717	+		upstream	-355
32	28	NA	LBD21.31R	159827	164910	-		inside	4254
21	19	NA	LBD21.31R	286427	288019	-		overlapEn	1802
21	18	NA	LBD21.31R	457316	459522	+		overlapSt	-9
18	15	NA	LBD21.31R	554428	564520	+		inside	6021
17	14	NA	LBD21.31R	1380506	1384282	+		inside	288
19	16	NA	LBD21.31R	1432709	1436609	+		upstream	-826
18	14	NA	LBD21.31R	1488317	1488949	+		upstream	-737
21	18	NA	LBD21.31R	1926730	1926981	+		upstream	-386
19	16	NA	LBD21.31R	1985821	1986374	-		downstrea	1596
26	22	NA	LBD21.31R	1992431	1994565	-		upstream	-4017
25	23	NA	LBD21.31R	2145453	2153015	+		upstream	-2205
17	14	NA	LBD21.31R	2174154	2175874	+		inside	1418
16	14	NA	LBD21.31R	2309317	2313931	+		inside	1242
27	25	NA	LBD21.31R	2418959	2419646	+		downstrea	6305
18	16	NA	LBD21.31R	2701843	2705500	-		inside	640
32	30	NA	LBD21.31R	2751574	2757351	+		inside	79
22	20	NA	LBD21.31R	3188159	3189958	+		inside	67
64	61	NA	LBD21.31R	3192473	3193492	+		inside	434
29	28	NA	LBD21.31R	3194810	3195886	-		inside	996
92	90	NA	LBD21.31R	3201672	3202760	-		upstream	-141
62	60	NA	LBD21.31R	3203146	3203886	-		upstream	-365
21	19	NA	LBD21.31R	3345964	3349933	+		inside	1938
31	26	NA	LBD21.31R	3788859	3790666	-		overlapSt	17
21	19	NA	LBD21.31R	3830946	3835094	+		inside	935
25	22	NA	LBD21.31R	3877811	3882573	-		downstrea	12386
19	16	NA	LBD21.31R	4758852	4759878	+		downstrea	6561

40	38	NA	LBD21.31R	4777056	4777541	-	downstrea	1885
17	15	NA	LBD21.31R	4982509	4986316	-	inside	1495
17	13	NA	LBD21.31R	5158751	5159863	-	upstream	-6324
24	21	NA	LBD21.31R	5185638	5190979	-	inside	5335
28	26	NA	LBD21.31R	5201109	5202092	-	upstream	-13353
19	17	NA	LBD21.31R	5244852	5252021	+	inside	3924
80	78	NA	LBD21.31R	5320857	5321204	+	inside	18
29	28	NA	LBD21.31R	5539256	5540996	-	upstream	-4997
24	22	NA	LBD21.31R	5555686	5557863	-	upstream	-1259
40	38	NA	LBD21.31R	6688729	6691334	-	overlapSt	271
18	16	NA	LBD21.31R	6936306	6939889	+	upstream	-3144
21	18	NA	LBD21.31R	6951211	6956430	-	upstream	-3279
18	17	NA	LBD21.31R	7261226	7263942	+	inside	1511
34	29	NA	LBD21.31R	7540268	7542125	+	upstream	-4428
29	24	NA	LBD21.31R	7812605	7813660	-	upstream	-678
78	76	NA	LBD21.31R	7813770	7816013	-	upstream	-16447
50	48	NA	LBD21.31R	7839613	7849712	-	downstrea	15219
23	20	NA	LBD21.31R	9844880	9848021	+	inside	2671
26	24	NA	LBD21.31R	9943878	9945607	-	downstrea	2549
30	29	NA	LBD21.31R	9984160	9985937	-	upstream	-783
18	15	NA	LBD21.31R	10011999	10016353	+	overlapSt	-175
18	16	NA	LBD21.31R	10243067	10243261	-	downstrea	954
18	14	NA	LBD21.31R	10490979	10494550	-	upstream	-1862
17	15	NA	LBD21.31R	10677872	10681793	-	overlapSt	48
17	14	NA	LBD21.31R	11353130	11356983	-	upstream	-2722
16	12	NA	LBD21.31R	11770699	11773280	-	inside	1035
28	24	NA	LBD21.31R	11796617	11800728	-	upstream	-549
18	15	NA	LBD21.31R	12247192	12249694	-	upstream	-1659
17	15	NA	LBD21.31R	12258252	12261258	+	inside	2632
27	24	NA	LBD21.31R	12330740	12331554	-	upstream	-58
21	17	NA	LBD21.31R	12958757	12961224	+	overlapSt	-203
19	16	NA	LBD21.31R	13003659	13004931	+	upstream	-1150
30	26	NA	LBD21.31R	13059593	13062592	+	overlapEn	2957
20	17	NA	LBD21.31R	13306929	13311828	-	upstream	-4851
19	17	NA	LBD21.31R	13379458	13381364	-	downstrea	6682
17	15	NA	LBD21.31R	13564597	13565645	+	downstrea	3065
19	16	NA	LBD21.31R	13674704	13675219	+	upstream	-642
17	14	NA	LBD21.31R	14447339	14452432	+	downstrea	6640
25	22	NA	LBD21.31R	14872134	14872834	-	downstrea	19767
19	16	NA	LBD21.31R	15185477	15185977	-	upstream	-1646
19	17	NA	LBD21.31R	15371119	15377454	+	inside	347
22	18	NA	LBD21.31R	15897931	15898035	+	upstream	-900
25	22	NA	LBD21.31R	136612	139531	+	downstrea	3951
58	56	NA	LBD21.31R	193697	197138	-	upstream	-38817
25	22	NA	LBD21.31R	499261	503175	+	downstrea	29949
77	75	NA	LBD21.31R	336917	337153	+	downstrea	2978
44	42	NA	LBD21.31R	435395	436960	-	upstream	-194
31	30	NA	LBD21.31R	438693	439050	+	overlapEn	186
33	31	NA	LBD21.31R	703587	709004	+	upstream	-6419
27	26	NA	LBD21.31R	746690	748017	-	downstrea	1767
27	25	NA	LBD21.31R	100631	101775	+	inside	541
19	17	NA	LBD21.31R	121252	122017	+	upstream	-927
23	20	NA	LBD21.31R	373902	378083	-	upstream	-3682
22	20	NA	LBD21.31R	386255	388907	-	upstream	-7539

17	14	NA	LBD21.31R	533760	552442	+	inside	2324
37	35	NA	LBD21.31R	474713	477251	-	downstrea	10656
39	36	NA	LBD21.31R	185949	186314	-	upstream	-6499
57	55	NA	LBD21.31R	230695	231069	-	downstrea	4351
19	16	NA	LBD21.31R	125722	129365	+	inside	2027
18	14	NA	LBD21.31R	182526	191285	+	upstream	-8282
21	19	NA	LBD21.31R	336651	340385	-	upstream	-1218
63	61	NA	LBD21.31R	375524	382339	+	upstream	-9056
28	25	NA	LBD21.31R	121540	121881	-	downstrea	24055
16	12	NA	LBD21.31R	162604	166175	-	downstrea	135850
31	28	NA	LBD21.31R	286887	287644	-	downstrea	57965
24	20	NA	LBD21.31R	255064	256341	+	upstream	-3618
38	36	NA	LBD21.31R	243811	244128	+	downstrea	3251
36	33	NA	LBD21.31R	261478	261795	+	downstrea	2119
47	45	NA	LBD21.31R	279248	279568	+	upstream	-7004
31	29	NA	LBD21.31R	96849	104538	+	downstrea	12432
24	22	NA	LBD21.31R	150794	155589	-	inside	2059
36	30	NA	LBD21.31R	89468	89677	+	downstrea	2339
19	16	NA	LBD21.31R	210714	212429	+	upstream	-18750
40	38	NA	LBD21.31R	54850	60572	-	downstrea	9191
18	17	NA	LBD21.31R	165764	167397	+	upstream	-2071
17	13	NA	LBD21.31R	194889	197500	-	inside	558
20	16	NA	LBD21.31R	274982	284640	-	inside	7474
18	14	NA	LBD21.31R	21430	26858	+	upstream	-964
31	28	NA	LBD21.31R	183283	183870	+	downstrea	4297
17	15	NA	LBD21.31R	140296	143001	-	downstrea	11549
19	16	NA	LBD21.31R	199945	205851	+	inside	3257
29	28	NA	LBD21.31R	64153	65366	+	downstrea	4385
29	27	NA	LBD21.31R	194973	196597	-	downstrea	13200
63	61	NA	LBD21.31R	160954	161337	+	upstream	-50317
20	17	NA	LBD21.31R	109338	110465	+	upstream	-3190
17	15	NA	LBD21.31R	113891	116786	+	upstream	-1219
58	56	NA	LBD21.31R	116723	117018	+	inside	19
21	19	NA	LBD21.31R	7821	17171	-	inside	5947
46	45	NA	LBD21.31R	70232	71679	-	inside	303
18	16	NA	LBD21.31R	118786	126050	-	inside	1398
22	19	NA	LBD21.31R	99508	102279	-	inside	1585
44	42	NA	LBD21.31R	95718	98362	+	inside	548
18	16	NA	LBD21.31R	18047	18426	+	upstream	-1164
17	15	NA	LBD21.31R	94249	100850	-	inside	3268
32	30	NA	LBD21.31R	59887	63047	+	inside	707
17	14	NA	LBD21.31R	3275	4020	+	upstream	-1186
17	14	NA	LBD21.31R	28036	33071	+	upstream	-11388
37	35	NA	LBD21.31R	62331	65349	+	upstream	-3779
17	16	NA	LBD21.31R	66691	69240	+	inside	313
19	18	NA	LBD21.31R	6537	8055	-	upstream	-7490
33	32	NA	LBD21.31R	28859	31174	+	upstream	-5430
75	73	NA	LBD21.31R	61152	61778	+	inside	26
28	26	NA	LBD21.31R	46265	46630	+	downstrea	7788
21	17	NA	LBD21.31R	84666	85204	+	overlapEn	366
42	40	NA	LBD21.31R	8978	13165	+	upstream	-880
20	18	NA	LBD21.31R	7691	10609	-	inside	2573
20	16	NA	LBD21.31R	16205	17610	-	downstrea	2425
22	20	NA	LBD21.31R	28580	30088	+	downstrea	9229

19	15	NA	LBD21.31R	35206	35497	+	upstream	-9090
41	37	NA	LBD21.31R	70936	73058	-	downstrea	2942
34	31	NA	LBD21.31R	16345	19466	-	upstream	-1875
23	19	NA	LBD21.31R	31887	35361	-	downstrea	4791
19	16	NA	LBD21.31R	51460	53460	+	upstream	-3136
29	25	NA	LBD21.31R	11414	12885	+	downstrea	1965
17	15	NA	LBD21.31R	51414	55069	+	inside	3225
24	22	NA	LBD21.31R	12076	19649	-	upstream	-3187
18	16	NA	LBD21.31R	36651	38957	-	upstream	-887
25	22	NA	LBD21.31R	36453	38308	-	inside	716
19	17	NA	LBD21.31R	4336	7827	-	inside	1314
19	15	NA	LBD21.31R	11617	14159	+	downstrea	3091
25	23	NA	LBD21.31R	15266	15851	+	upstream	-11408
19	17	NA	LBD21.31R	13380	15387	+	upstream	-637
22	21	NA	LBD21.31R	12142	13396	-	inside	1242
17	14	NA	LBD21.31R	2196	5547	-	inside	2302
18	15	NA	LBD21.31R	4680	7778	+	downstrea	5032
38	36	NA	LBD21.31R	12232	12645	-	upstream	-18850
20	17	NA	LBD21.31R	11500	17366	-	upstream	-1619
20	17	NA	LBD21.31R	4548	5317	-	downstrea	3639
25	23	NA	LBD21.31R	327	706	-	upstream	-1286

shortestD	fromOverlappingOrNearest
2493	NearestLocation
3399	NearestLocation
70	NearestLocation
2394	NearestLocation
3610	NearestLocation
14758	NearestLocation
2877	NearestLocation
1031	NearestLocation
20	NearestLocation
3735	NearestLocation
186	NearestLocation
265	NearestLocation
214	NearestLocation
234	NearestLocation
30	NearestLocation
6630	NearestLocation
1234	NearestLocation
14	NearestLocation
51	NearestLocation
75	NearestLocation
650	NearestLocation
739	NearestLocation
279	NearestLocation
287	NearestLocation
431	NearestLocation
986	NearestLocation
993	NearestLocation
148	NearestLocation
3389	NearestLocation
277	NearestLocation
2680	NearestLocation
7149	NearestLocation
1372	NearestLocation
672	NearestLocation
314	NearestLocation
2290	NearestLocation
2985	NearestLocation
62	NearestLocation
2025	NearestLocation
936	NearestLocation
19426	NearestLocation
7401	NearestLocation
1332	NearestLocation
1320	NearestLocation
654	NearestLocation
794	NearestLocation
7148	NearestLocation
1000	NearestLocation
553	NearestLocation
82	NearestLocation
3673	NearestLocation

1987	NearestLocation
304	NearestLocation
3846	NearestLocation
1196	NearestLocation
1333	NearestLocation
1313	NearestLocation
2781	NearestLocation
1336	NearestLocation
472	NearestLocation
2536	NearestLocation
89353	NearestLocation
15231	NearestLocation
29226	NearestLocation
4316	NearestLocation
2080	NearestLocation
7895	NearestLocation
2199	NearestLocation
35	NearestLocation
560	NearestLocation
2798	NearestLocation
1541	NearestLocation
87	NearestLocation
5941	NearestLocation
936	NearestLocation
2575	NearestLocation
22035	NearestLocation
2272	NearestLocation
3384	NearestLocation
2068	NearestLocation
141	NearestLocation
443	NearestLocation
1344	NearestLocation
1	NearestLocation
662	NearestLocation
1970	NearestLocation
151	NearestLocation
335	NearestLocation
2080	NearestLocation
199	NearestLocation
5132	NearestLocation
440	NearestLocation
2849	NearestLocation
102	NearestLocation
2710	NearestLocation
5579	NearestLocation
5096	NearestLocation
2392	NearestLocation
446	NearestLocation
2079	NearestLocation
1187	NearestLocation
601	NearestLocation
937	NearestLocation
36	NearestLocation
5345	NearestLocation

3463	NearestLocation
3072	NearestLocation
12	NearestLocation
1135	NearestLocation
1368	NearestLocation
8033	NearestLocation
246	NearestLocation
92	NearestLocation
112	NearestLocation
2235	NearestLocation
927	NearestLocation
13637	NearestLocation
800	NearestLocation
7059	NearestLocation
44	NearestLocation
972	NearestLocation
4688	NearestLocation
1827	NearestLocation
7	NearestLocation
7750	NearestLocation
3697	NearestLocation
3864	NearestLocation
290	NearestLocation
18261	NearestLocation
7755	NearestLocation
11083	NearestLocation
3555	NearestLocation
3171	NearestLocation
1704	NearestLocation
3256	NearestLocation
7003	NearestLocation
2933	NearestLocation
4637	NearestLocation
1843	NearestLocation
603	NearestLocation
4437	NearestLocation
3284	NearestLocation
2489	NearestLocation
5729	NearestLocation
48	NearestLocation
3366	NearestLocation
2357	NearestLocation
8	NearestLocation
293	NearestLocation
1517	NearestLocation
14455	NearestLocation
10222	NearestLocation
31017	NearestLocation
3720	NearestLocation
1857	NearestLocation
41	NearestLocation
2148	NearestLocation
4110	NearestLocation
2452	NearestLocation

3804	NearestLocation
2791	NearestLocation
1750	NearestLocation
1361	NearestLocation
26	NearestLocation
20046	NearestLocation
3463	NearestLocation
996	NearestLocation
341	NearestLocation
1925	NearestLocation
1209	NearestLocation
3202	NearestLocation
55	NearestLocation
100	NearestLocation
5501	NearestLocation
118	NearestLocation
9787	NearestLocation
3590	NearestLocation
492	NearestLocation
2353	NearestLocation
136	NearestLocation
555	NearestLocation
27	NearestLocation
1041	NearestLocation
233	NearestLocation
6690	NearestLocation
786	NearestLocation
1284	NearestLocation
574	NearestLocation
129	NearestLocation
3124	NearestLocation
50	NearestLocation
45	NearestLocation
271	NearestLocation
349	NearestLocation
1291	NearestLocation
35	NearestLocation
2007	NearestLocation
77	NearestLocation
2512	NearestLocation
671	NearestLocation
99	NearestLocation
1143	NearestLocation
490	NearestLocation
1099	NearestLocation
2246	NearestLocation
4432	NearestLocation
865	NearestLocation
431	NearestLocation
4408	NearestLocation
3083	NearestLocation
3418	NearestLocation
175	NearestLocation
3100	NearestLocation

5450	NearestLocation
7008	NearestLocation
31	NearestLocation
246	NearestLocation
1046	NearestLocation
530	NearestLocation
181	NearestLocation
1737	NearestLocation
1001	NearestLocation
4182	NearestLocation
1019	NearestLocation
56	NearestLocation
1259	NearestLocation
4402	NearestLocation
2910	NearestLocation
160	NearestLocation
114	NearestLocation
702	NearestLocation
667	NearestLocation
436	NearestLocation
3906	NearestLocation
119	NearestLocation
739	NearestLocation
31	NearestLocation
4081	NearestLocation
286	NearestLocation
1093	NearestLocation
105	NearestLocation
1705	NearestLocation
651	NearestLocation
1671	NearestLocation
3027	NearestLocation
11093	NearestLocation
628	NearestLocation
24238	NearestLocation
11	NearestLocation
554	NearestLocation
715	NearestLocation
134	NearestLocation
486	NearestLocation
82	NearestLocation
291	NearestLocation
66	NearestLocation
3259	NearestLocation
10933	NearestLocation
10687	NearestLocation
102	NearestLocation
5727	NearestLocation
4080	NearestLocation
118	NearestLocation
330	NearestLocation
2173	NearestLocation
1411	NearestLocation
1	NearestLocation

6817 NearestLocation
3863 NearestLocation
1850 NearestLocation
568 NearestLocation
1077 NearestLocation
1 NearestLocation
6011 NearestLocation
646 NearestLocation
59 NearestLocation
27 NearestLocation
9747 NearestLocation
101 NearestLocation
1085 NearestLocation
456 NearestLocation
715 NearestLocation
33 NearestLocation
6915 NearestLocation
12362 NearestLocation
690 NearestLocation
257 NearestLocation
4120 NearestLocation
42 NearestLocation
3600 NearestLocation
12525 NearestLocation
1508 NearestLocation
112 NearestLocation
1359 NearestLocation
5699 NearestLocation
2982 NearestLocation
1751 NearestLocation
7 NearestLocation
3728 NearestLocation
1985 NearestLocation
5016 NearestLocation
8061 NearestLocation
3791 NearestLocation
3603 NearestLocation
13655 NearestLocation
1086 NearestLocation
5759 NearestLocation
489 NearestLocation
146 NearestLocation
154 NearestLocation
188 NearestLocation
807 NearestLocation
1795 NearestLocation
723 NearestLocation
1319 NearestLocation
1786 NearestLocation
382 NearestLocation
1719 NearestLocation
112 NearestLocation
2227 NearestLocation
4653 NearestLocation

4847	NearestLocation
10548	NearestLocation
2338	NearestLocation
182	NearestLocation
896	NearestLocation
1597	NearestLocation
367	NearestLocation
1882	NearestLocation
4134	NearestLocation
278	NearestLocation
1719	NearestLocation
728	NearestLocation
3067	NearestLocation
118	NearestLocation
1560	NearestLocation
2306	NearestLocation
853	NearestLocation
7653	NearestLocation
6	NearestLocation
1039	NearestLocation
6473	NearestLocation
696	NearestLocation
1027	NearestLocation
5426	NearestLocation
5722	NearestLocation
4857	NearestLocation
64	NearestLocation
2900	NearestLocation
550	NearestLocation
609	NearestLocation
371	NearestLocation
845	NearestLocation
581	NearestLocation
971	NearestLocation
125	NearestLocation
288	NearestLocation
1107	NearestLocation
563	NearestLocation
103	NearestLocation
3013	NearestLocation
2893	NearestLocation
778	NearestLocation
138	NearestLocation
48	NearestLocation
7234	NearestLocation
7693	NearestLocation
1739	NearestLocation
6135	NearestLocation
45	NearestLocation
109	NearestLocation
458	NearestLocation
2120	NearestLocation
1562	NearestLocation
105	NearestLocation

16	NearestLocation
1296	NearestLocation
2178	NearestLocation
1554	NearestLocation
59	NearestLocation
799	NearestLocation
291	NearestLocation
122	NearestLocation
2476	NearestLocation
3301	NearestLocation
1916	NearestLocation
224	NearestLocation
2830	NearestLocation
7592	NearestLocation
1485	NearestLocation
668	NearestLocation
21	NearestLocation
2509	NearestLocation
851	NearestLocation
993	NearestLocation
6312	NearestLocation
3874	NearestLocation
1893	NearestLocation
1304	NearestLocation
2922	NearestLocation
100	NearestLocation
4190	NearestLocation
195	NearestLocation
277	NearestLocation
221	NearestLocation
3040	NearestLocation
147	NearestLocation
3297	NearestLocation
10343	NearestLocation
197	NearestLocation
228	NearestLocation
199	NearestLocation
5627	NearestLocation
15604	NearestLocation
964	NearestLocation
5176	NearestLocation
384	NearestLocation
7341	NearestLocation
136	NearestLocation
1140	NearestLocation
9521	NearestLocation
6028	NearestLocation
6125	NearestLocation
2539	NearestLocation
295	NearestLocation
4078	NearestLocation
4135	NearestLocation
21073	NearestLocation
28496	NearestLocation

75367 NearestLocation
8902 NearestLocation
6832 NearestLocation
41388 NearestLocation
3311 NearestLocation
7119 NearestLocation
1896 NearestLocation
329 NearestLocation
75 NearestLocation
796 NearestLocation
1154 NearestLocation
173 NearestLocation
2495 NearestLocation
8492 NearestLocation
2874 NearestLocation
478 NearestLocation
4152 NearestLocation
593 NearestLocation
649 NearestLocation
35 NearestLocation
599 NearestLocation
1090 NearestLocation
152 NearestLocation
523 NearestLocation
516 NearestLocation
67 NearestLocation
134 NearestLocation
1657 NearestLocation
3074 NearestLocation
1908 NearestLocation
6456 NearestLocation
80 NearestLocation
543 NearestLocation
2018 NearestLocation
2238 NearestLocation
2064 NearestLocation
16208 NearestLocation
1527 NearestLocation
692 NearestLocation
304 NearestLocation
306 NearestLocation
1862 NearestLocation
3043 NearestLocation
86 NearestLocation
217 NearestLocation
3694 NearestLocation
221 NearestLocation
2381 NearestLocation
248 NearestLocation
1728 NearestLocation
1834 NearestLocation
1626 NearestLocation
4696 NearestLocation
733 NearestLocation

4619	NearestLocation
313	NearestLocation
838	NearestLocation
3474	NearestLocation
2841	NearestLocation
15552	NearestLocation
3317	NearestLocation
3773	NearestLocation
3229	NearestLocation
1321	NearestLocation
249	NearestLocation
1765	NearestLocation
1277	NearestLocation
4519	NearestLocation
4943	NearestLocation
835	NearestLocation
353	NearestLocation
615	NearestLocation
1768	NearestLocation
3019	NearestLocation
3679	NearestLocation
539	NearestLocation
54	NearestLocation
7288	NearestLocation
76	NearestLocation
827	NearestLocation
618	NearestLocation
6351	NearestLocation
38	NearestLocation
712	NearestLocation
8579	NearestLocation
8378	NearestLocation
3600	NearestLocation
13367	NearestLocation
18101	NearestLocation
1426	NearestLocation
24	NearestLocation
56	NearestLocation
8663	NearestLocation
1031	NearestLocation
7786	NearestLocation
132	NearestLocation
15	NearestLocation
6079	NearestLocation
1430	NearestLocation
20632	NearestLocation
9130	NearestLocation
13678	NearestLocation
2480	NearestLocation
1570	NearestLocation
646	NearestLocation
15798	NearestLocation
3946	NearestLocation
1619	NearestLocation

1 NearestLocation
1532 NearestLocation
15 NearestLocation
2375 NearestLocation
3399 NearestLocation
4312 NearestLocation
6145 NearestLocation
53 NearestLocation
1449 NearestLocation
516 NearestLocation
1184 NearestLocation
461 NearestLocation
705 NearestLocation
827 NearestLocation
2001 NearestLocation
245 NearestLocation
5490 NearestLocation
315 NearestLocation
48 NearestLocation
1298 NearestLocation
3733 NearestLocation
38 NearestLocation
506 NearestLocation
3640 NearestLocation
1617 NearestLocation
51 NearestLocation
1272 NearestLocation
655 NearestLocation
206 NearestLocation
8293 NearestLocation
3823 NearestLocation
2873 NearestLocation
500 NearestLocation
474 NearestLocation
1090 NearestLocation
7747 NearestLocation
6810 NearestLocation
2897 NearestLocation
435 NearestLocation
2383 NearestLocation
0 NearestLocation
25 NearestLocation
836 NearestLocation
33 NearestLocation
434 NearestLocation
867 NearestLocation
118 NearestLocation
663 NearestLocation
163 NearestLocation
20 NearestLocation
1110 NearestLocation
3440 NearestLocation
2072 NearestLocation
427 NearestLocation

2132	NearestLocation
2906	NearestLocation
1849	NearestLocation
2034	NearestLocation
36	NearestLocation
1564	NearestLocation
8	NearestLocation
385	NearestLocation
4611	NearestLocation
105	NearestLocation
1564	NearestLocation
686	NearestLocation
28	NearestLocation
5079	NearestLocation
583	NearestLocation
1334	NearestLocation
4998	NearestLocation
2274	NearestLocation
1601	NearestLocation
2443	NearestLocation
3635	NearestLocation
1033	NearestLocation
86	NearestLocation
9118	NearestLocation
2539	NearestLocation
9223	NearestLocation
53	NearestLocation
86	NearestLocation
4265	NearestLocation
3240	NearestLocation
335	NearestLocation
825	NearestLocation
5420	NearestLocation
4217	NearestLocation
4107	NearestLocation
801	NearestLocation
28172	NearestLocation
12971	NearestLocation
7934	NearestLocation
5202	NearestLocation
6349	NearestLocation
3562	NearestLocation
22385	NearestLocation
2380	NearestLocation
37	NearestLocation
2671	NearestLocation
726	NearestLocation
2866	NearestLocation
13183	NearestLocation
174	NearestLocation
3789	NearestLocation
21584	NearestLocation
483	NearestLocation
5392	NearestLocation

19062	NearestLocation
78	NearestLocation
19	NearestLocation
3534	NearestLocation
11968	NearestLocation
7487	NearestLocation
440	NearestLocation
3163	NearestLocation
12129	NearestLocation
1168	NearestLocation
3564	NearestLocation
3829	NearestLocation
4224	NearestLocation
1037	NearestLocation
2310	NearestLocation
32	NearestLocation
1484	NearestLocation
4269	NearestLocation
71	NearestLocation
2903	NearestLocation
2491	NearestLocation
2670	NearestLocation
862	NearestLocation
1135	NearestLocation
3622	NearestLocation
2029	NearestLocation
1087	NearestLocation
2142	NearestLocation
3258	NearestLocation
30	NearestLocation
932	NearestLocation
265	NearestLocation
51	NearestLocation
5060	NearestLocation
6954	NearestLocation
7985	NearestLocation
401	NearestLocation
10203	NearestLocation
11613	NearestLocation
1643	NearestLocation
13196	NearestLocation
4116	NearestLocation
971	NearestLocation
348	NearestLocation
3559	NearestLocation
3274	NearestLocation
3687	NearestLocation
2634	NearestLocation
708	NearestLocation
2449	NearestLocation
2777	NearestLocation
507	NearestLocation
4384	NearestLocation
3955	NearestLocation

875 NearestLocation
1071 NearestLocation
1135 NearestLocation
2670 NearestLocation
24 NearestLocation
1694 NearestLocation
6074 NearestLocation
559 NearestLocation
876 NearestLocation
110 NearestLocation
5013 NearestLocation
6484 NearestLocation
2883 NearestLocation
1579 NearestLocation
71 NearestLocation
316 NearestLocation
1043 NearestLocation
1357 NearestLocation
3759 NearestLocation
186 NearestLocation
74 NearestLocation
1001 NearestLocation
663 NearestLocation
2527 NearestLocation
3964 NearestLocation
404 NearestLocation
4176 NearestLocation
65 NearestLocation
826 NearestLocation
3094 NearestLocation
1195 NearestLocation
3813 NearestLocation
3211 NearestLocation
2098 NearestLocation
156 NearestLocation
2877 NearestLocation
1731 NearestLocation
2287 NearestLocation
584 NearestLocation
2423 NearestLocation
368 NearestLocation
1244 NearestLocation
33 NearestLocation
2300 NearestLocation
44 NearestLocation
1988 NearestLocation
375 NearestLocation
1528 NearestLocation
1364 NearestLocation
5017 NearestLocation
1705 NearestLocation
12 NearestLocation
1137 NearestLocation
4143 NearestLocation

811	NearestLocation
880	NearestLocation
3923	NearestLocation
359	NearestLocation
96	NearestLocation
392	NearestLocation
66	NearestLocation
6176	NearestLocation
159	NearestLocation
3494	NearestLocation
301	NearestLocation
410	NearestLocation
5954	NearestLocation
1187	NearestLocation
821	NearestLocation
4437	NearestLocation
864	NearestLocation
1	NearestLocation
1473	NearestLocation
1531	NearestLocation
286	NearestLocation
411	NearestLocation
474	NearestLocation
1294	NearestLocation
2295	NearestLocation
4323	NearestLocation
402	NearestLocation
84	NearestLocation
1766	NearestLocation
539	NearestLocation
1405	NearestLocation
383	NearestLocation
5790	NearestLocation
1387	NearestLocation
1	NearestLocation
36258	NearestLocation
29985	NearestLocation
9749	NearestLocation
639	NearestLocation
251	NearestLocation
8111	NearestLocation
8235	NearestLocation
1438	NearestLocation
10117	NearestLocation
2506	NearestLocation
10471	NearestLocation
14604	NearestLocation
1098	NearestLocation
81	NearestLocation
660	NearestLocation
6547	NearestLocation
5	NearestLocation
205	NearestLocation
14	NearestLocation

103	NearestLocation
278	NearestLocation
3793	NearestLocation
6873	NearestLocation
635	NearestLocation
446	NearestLocation
1127	NearestLocation
7381	NearestLocation
30	NearestLocation
1009	NearestLocation
1052	NearestLocation
1083	NearestLocation
90	NearestLocation
162	NearestLocation
1002	NearestLocation
3631	NearestLocation
2608	NearestLocation
90	NearestLocation
1161	NearestLocation
127	NearestLocation
904	NearestLocation
815	NearestLocation
144	NearestLocation
111	NearestLocation
8	NearestLocation
3566	NearestLocation
1728	NearestLocation
4876	NearestLocation
242	NearestLocation
1197	NearestLocation
669	NearestLocation
1880	NearestLocation
1185	NearestLocation
2959	NearestLocation
341	NearestLocation
87	NearestLocation
10001	NearestLocation
5633	NearestLocation
872	NearestLocation
9086	NearestLocation
2780	NearestLocation
170	NearestLocation
10405	NearestLocation
58	NearestLocation
2512	NearestLocation
51760	NearestLocation
1960	NearestLocation
77240	NearestLocation
82953	NearestLocation
47	NearestLocation
52995	NearestLocation
32	NearestLocation
821	NearestLocation
3972	NearestLocation

30	NearestLocation
1373	NearestLocation
2467	NearestLocation
199	NearestLocation
783	NearestLocation
74	NearestLocation
966	NearestLocation
499	NearestLocation
1403	NearestLocation
2329	NearestLocation
3233	NearestLocation
793	NearestLocation
1651	NearestLocation
704	NearestLocation
293	NearestLocation
517	NearestLocation
3166	NearestLocation
3656	NearestLocation
6956	NearestLocation
1433	NearestLocation
1375	NearestLocation
4695	NearestLocation
1110	NearestLocation
38	NearestLocation
8260	NearestLocation
888	NearestLocation
338	NearestLocation
873	NearestLocation
5583	NearestLocation
291	NearestLocation
213	NearestLocation
161	NearestLocation
241	NearestLocation
55	NearestLocation
1276	NearestLocation
113	NearestLocation
2022	NearestLocation
870	NearestLocation
329	NearestLocation
3652	NearestLocation
6897	NearestLocation
495	NearestLocation
407	NearestLocation
4938	NearestLocation
62	NearestLocation
3549	NearestLocation
111	NearestLocation
624	NearestLocation
509	NearestLocation
1197	NearestLocation
3202	NearestLocation
1181	NearestLocation
1020	NearestLocation
146	NearestLocation

5	NearestLocation
3037	NearestLocation
40	NearestLocation
4490	NearestLocation
2309	NearestLocation
531	NearestLocation
922	NearestLocation
7396	NearestLocation
1105	NearestLocation
91	NearestLocation
3298	NearestLocation
4514	NearestLocation
2051	NearestLocation
2319	NearestLocation
808	NearestLocation
3453	NearestLocation
1280	NearestLocation
14027	NearestLocation
183	NearestLocation
510	NearestLocation
262	NearestLocation
198	NearestLocation
59	NearestLocation
106	NearestLocation
315	NearestLocation
89	NearestLocation
425	NearestLocation
41	NearestLocation
69	NearestLocation
1380	NearestLocation
6508	NearestLocation
855	NearestLocation
45340	NearestLocation
26411	NearestLocation
447	NearestLocation
6914	NearestLocation
31	NearestLocation
2284	NearestLocation
6	NearestLocation
43	NearestLocation
264	NearestLocation
9	NearestLocation
2094	NearestLocation
82	NearestLocation
1569	NearestLocation
2242	NearestLocation
1446	NearestLocation
414	NearestLocation
86	NearestLocation
5	NearestLocation
930	NearestLocation
1894	NearestLocation
1217	NearestLocation
1955	NearestLocation

800	NearestLocation
1058	NearestLocation
862	NearestLocation
4696	NearestLocation
9429	NearestLocation
39	NearestLocation
18	NearestLocation
3985	NearestLocation
321	NearestLocation
567	NearestLocation
311	NearestLocation
1090	NearestLocation
652	NearestLocation
2119	NearestLocation
3184	NearestLocation
476	NearestLocation
85	NearestLocation
109	NearestLocation
823	NearestLocation
724	NearestLocation
1161	NearestLocation
4000	NearestLocation
4907	NearestLocation
4023	NearestLocation
1868	NearestLocation
2602	NearestLocation
3271	NearestLocation
222	NearestLocation
172	NearestLocation
7383	NearestLocation
48288	NearestLocation
1008	NearestLocation
34	NearestLocation
52991	NearestLocation
293	NearestLocation
9170	NearestLocation
1476	NearestLocation
23377	NearestLocation
1510	NearestLocation
1142	NearestLocation
652	NearestLocation
1987	NearestLocation
4314	NearestLocation
1168	NearestLocation
2555	NearestLocation
246	NearestLocation
4916	NearestLocation
4768	NearestLocation
1556	NearestLocation
5904	NearestLocation
18968	NearestLocation
2112	NearestLocation
3674	NearestLocation
4705	NearestLocation

97	NearestLocation
6810	NearestLocation
4908	NearestLocation
859	NearestLocation
41	NearestLocation
681	NearestLocation
354	NearestLocation
2591	NearestLocation
1096	NearestLocation
33	NearestLocation
1777	NearestLocation
4637	NearestLocation
2764	NearestLocation
675	NearestLocation
3276	NearestLocation
481	NearestLocation
16	NearestLocation
96	NearestLocation
1921	NearestLocation
4429	NearestLocation
22890	NearestLocation
928	NearestLocation
2854	NearestLocation
173	NearestLocation
2080	NearestLocation
62	NearestLocation
1384	NearestLocation
1169	NearestLocation
277	NearestLocation
614	NearestLocation
99	NearestLocation
1505	NearestLocation
110	NearestLocation
1855	NearestLocation
2964	NearestLocation
154	NearestLocation
399	NearestLocation
266	NearestLocation
1397	NearestLocation
61	NearestLocation
578	NearestLocation
2	NearestLocation
169	NearestLocation
3465	NearestLocation
1275	NearestLocation
5418	NearestLocation
1142	NearestLocation
589	NearestLocation
9698	NearestLocation
2572	NearestLocation
1142	NearestLocation
728	NearestLocation
13584	NearestLocation
2285	NearestLocation

2540 NearestLocation
3760 NearestLocation
779 NearestLocation
7107 NearestLocation
359 NearestLocation
1323 NearestLocation
1938 NearestLocation
763 NearestLocation
2671 NearestLocation
143 NearestLocation
14181 NearestLocation
5324 NearestLocation
2670 NearestLocation
215 NearestLocation
384 NearestLocation
158 NearestLocation
75 NearestLocation
23 NearestLocation
305 NearestLocation
297 NearestLocation
11984 NearestLocation
3855 NearestLocation
300 NearestLocation
701 NearestLocation
12 NearestLocation
402 NearestLocation
3713 NearestLocation
6905 NearestLocation
84 NearestLocation
945 NearestLocation
11 NearestLocation
19841 NearestLocation
409 NearestLocation
126 NearestLocation
1087 NearestLocation
506 NearestLocation
587 NearestLocation
823 NearestLocation
2783 NearestLocation
2503 NearestLocation
802 NearestLocation
3166 NearestLocation
950 NearestLocation
2922 NearestLocation
693 NearestLocation
4 NearestLocation
360 NearestLocation
405 NearestLocation
20 NearestLocation
198 NearestLocation
411 NearestLocation
172 NearestLocation
116 NearestLocation
33 NearestLocation

100	NearestLocation
227	NearestLocation
1888	NearestLocation
632	NearestLocation
93	NearestLocation
3	NearestLocation
536	NearestLocation
854	NearestLocation
17	NearestLocation
503	NearestLocation
267	NearestLocation
13	NearestLocation
223	NearestLocation
627	NearestLocation
998	NearestLocation
776	NearestLocation
284	NearestLocation
61	NearestLocation
289	NearestLocation
1745	NearestLocation
1251	NearestLocation
279	NearestLocation
83	NearestLocation
90	NearestLocation
291	NearestLocation
758	NearestLocation
447	NearestLocation
430	NearestLocation
446	NearestLocation
70	NearestLocation
325	NearestLocation
94	NearestLocation
46	NearestLocation
8675	NearestLocation
38	NearestLocation
511	NearestLocation
2119	NearestLocation
239	NearestLocation
4562	NearestLocation
1323	NearestLocation
1021	NearestLocation
133	NearestLocation
2014	NearestLocation
1356	NearestLocation
917	NearestLocation
4578	NearestLocation
931	NearestLocation
1948	NearestLocation
645	NearestLocation
5328	NearestLocation
1460	NearestLocation
2237	NearestLocation
1043	NearestLocation
1201	NearestLocation

1277	NearestLocation
886	NearestLocation
250	NearestLocation
1886	NearestLocation
2513	NearestLocation
790	NearestLocation
400	NearestLocation
3118	NearestLocation
1225	NearestLocation
1904	NearestLocation
578	NearestLocation
941	NearestLocation
203	NearestLocation
454	NearestLocation
2105	NearestLocation
2147	NearestLocation
828	NearestLocation
4075	NearestLocation
107	NearestLocation
925	NearestLocation
3534	NearestLocation
1041	NearestLocation
3723	NearestLocation
89	NearestLocation
1589	NearestLocation
134	NearestLocation
62	NearestLocation
743	NearestLocation
1196	NearestLocation
1908	NearestLocation
113	NearestLocation
2975	NearestLocation
878	NearestLocation
225	NearestLocation
204	NearestLocation
3032	NearestLocation
19073	NearestLocation
30	NearestLocation
842	NearestLocation
387	NearestLocation
1239	NearestLocation
1207	NearestLocation
115	NearestLocation
26870	NearestLocation
4365	NearestLocation
156	NearestLocation
1124	NearestLocation
15231	NearestLocation
1403	NearestLocation
3853	NearestLocation
114	NearestLocation
10573	NearestLocation
3171	NearestLocation
11378	NearestLocation

2211	NearestLocation
4480	NearestLocation
20647	NearestLocation
8238	NearestLocation
14368	NearestLocation
3028	NearestLocation
3627	NearestLocation
6825	NearestLocation
161	NearestLocation
632	NearestLocation
652	NearestLocation
7320	NearestLocation
3479	NearestLocation
578	NearestLocation
122	NearestLocation
1872	NearestLocation
735	NearestLocation
5	NearestLocation
8360	NearestLocation
9936	NearestLocation
848	NearestLocation
457	NearestLocation
1817	NearestLocation
6815	NearestLocation
3001	NearestLocation
3585	NearestLocation
2832	NearestLocation
2288	NearestLocation
2062	NearestLocation
7168	NearestLocation
30116	NearestLocation
18362	NearestLocation
4347	NearestLocation
20	NearestLocation
10	NearestLocation
1720	NearestLocation
670	NearestLocation
6846	NearestLocation
9915	NearestLocation
49765	NearestLocation
12289	NearestLocation
5473	NearestLocation
945	NearestLocation
101	NearestLocation
93	NearestLocation
2667	NearestLocation
403	NearestLocation
112	NearestLocation
2174	NearestLocation
3556	NearestLocation
25	NearestLocation
133	NearestLocation
261	NearestLocation
751	NearestLocation

2411	NearestLocation
4018	NearestLocation
1730	NearestLocation
90	NearestLocation
3168	NearestLocation
394	NearestLocation
445	NearestLocation
87	NearestLocation
59	NearestLocation
1073	NearestLocation
1294	NearestLocation
1330	NearestLocation
1595	NearestLocation
1377	NearestLocation
2803	NearestLocation
889	NearestLocation
828	NearestLocation
24	NearestLocation
615	NearestLocation
1193	NearestLocation
1732	NearestLocation
643	NearestLocation
6632	NearestLocation
47	NearestLocation
3415	NearestLocation
71	NearestLocation
1951	NearestLocation
1504	NearestLocation
6447	NearestLocation
96	NearestLocation
1233	NearestLocation
68	NearestLocation
1216	NearestLocation
1716	NearestLocation
16	NearestLocation
457	NearestLocation
4501	NearestLocation
103	NearestLocation
4688	NearestLocation
4516	NearestLocation
801	NearestLocation
253	NearestLocation
37	NearestLocation
663	NearestLocation
299	NearestLocation
853	NearestLocation
53	NearestLocation
8785	NearestLocation
20823	NearestLocation
8608	NearestLocation
12651	NearestLocation
28291	NearestLocation
71	NearestLocation
12337	NearestLocation

3823	NearestLocation
1961	NearestLocation
4202	NearestLocation
4328	NearestLocation
1466	NearestLocation
26	NearestLocation
38	NearestLocation
1275	NearestLocation
2870	NearestLocation
1986	NearestLocation
864	NearestLocation
18	NearestLocation
811	NearestLocation
2846	NearestLocation
1743	NearestLocation
1717	NearestLocation
1313	NearestLocation
810	NearestLocation
398	NearestLocation
3136	NearestLocation
3099	NearestLocation
12032	NearestLocation
364	NearestLocation
233	NearestLocation
294	NearestLocation
950	NearestLocation
20018	NearestLocation
353	NearestLocation
2058	NearestLocation
559	NearestLocation
3366	NearestLocation
1230	NearestLocation
2231	NearestLocation
2975	NearestLocation
426	NearestLocation
2380	NearestLocation
9837	NearestLocation
622	NearestLocation
5250	NearestLocation
21	NearestLocation
2148	NearestLocation
35919	NearestLocation
5710	NearestLocation
38714	NearestLocation
3416	NearestLocation
2971	NearestLocation
1825	NearestLocation
1006	NearestLocation
1008	NearestLocation
9990	NearestLocation
3766	NearestLocation
359	NearestLocation
9587	NearestLocation
277	NearestLocation

638	NearestLocation
14893	NearestLocation
5203	NearestLocation
2314	NearestLocation
1242	NearestLocation
61	NearestLocation
1723	NearestLocation
1473	NearestLocation
7808	NearestLocation
4403	NearestLocation
73	NearestLocation
181	NearestLocation
1452	NearestLocation
169	NearestLocation
3322	NearestLocation
32	NearestLocation
2415	NearestLocation
118	NearestLocation
416	NearestLocation
1835	NearestLocation
117	NearestLocation
102	NearestLocation
524	NearestLocation
9916	NearestLocation
944	NearestLocation
545	NearestLocation
1011	NearestLocation
13	NearestLocation
2934	NearestLocation
2066	NearestLocation
6958	NearestLocation
3484	NearestLocation
9	NearestLocation
1120	NearestLocation
326	NearestLocation
234	NearestLocation
1710	NearestLocation
234	NearestLocation
11690	NearestLocation
4660	NearestLocation
29	NearestLocation
1545	NearestLocation
141	NearestLocation
9786	NearestLocation
538	NearestLocation
17446	NearestLocation
12236	NearestLocation
23849	NearestLocation
10219	NearestLocation
93	NearestLocation
354	NearestLocation
1304	NearestLocation
574	NearestLocation
31381	NearestLocation

80346	NearestLocation
18229	NearestLocation
1558	NearestLocation
2735	NearestLocation
16340	NearestLocation
139	NearestLocation
29511	NearestLocation
966	NearestLocation
1393	NearestLocation
251	NearestLocation
146	NearestLocation
6627	NearestLocation
578	NearestLocation
8495	NearestLocation
15	NearestLocation
1554	NearestLocation
3665	NearestLocation
2188	NearestLocation
6919	NearestLocation
822	NearestLocation
197	NearestLocation
779	NearestLocation
628	NearestLocation
321	NearestLocation
427	NearestLocation
1332	NearestLocation
1818	NearestLocation
102	NearestLocation
829	NearestLocation
180	NearestLocation
9	NearestLocation
3860	NearestLocation
288	NearestLocation
254	NearestLocation
503	NearestLocation
168	NearestLocation
776	NearestLocation
4017	NearestLocation
1990	NearestLocation
63	NearestLocation
1242	NearestLocation
5618	NearestLocation
86	NearestLocation
79	NearestLocation
67	NearestLocation
364	NearestLocation
80	NearestLocation
141	NearestLocation
365	NearestLocation
1694	NearestLocation
17	NearestLocation
935	NearestLocation
7347	NearestLocation
5535	NearestLocation

1172	NearestLocation
924	NearestLocation
6324	NearestLocation
6	NearestLocation
13353	NearestLocation
3003	NearestLocation
18	NearestLocation
4997	NearestLocation
1259	NearestLocation
271	NearestLocation
2917	NearestLocation
3279	NearestLocation
1004	NearestLocation
4156	NearestLocation
678	NearestLocation
16447	NearestLocation
4864	NearestLocation
216	NearestLocation
612	NearestLocation
783	NearestLocation
62	NearestLocation
425	NearestLocation
1862	NearestLocation
48	NearestLocation
2722	NearestLocation
800	NearestLocation
549	NearestLocation
1659	NearestLocation
84	NearestLocation
58	NearestLocation
39	NearestLocation
713	NearestLocation
42	NearestLocation
4851	NearestLocation
4570	NearestLocation
2017	NearestLocation
423	NearestLocation
1547	NearestLocation
18857	NearestLocation
1646	NearestLocation
347	NearestLocation
435	NearestLocation
1032	NearestLocation
38817	NearestLocation
26035	NearestLocation
2742	NearestLocation
194	NearestLocation
171	NearestLocation
6213	NearestLocation
25	NearestLocation
74	NearestLocation
725	NearestLocation
3682	NearestLocation
7539	NearestLocation

2324	NearestLocation
7879	NearestLocation
6499	NearestLocation
3694	NearestLocation
1398	NearestLocation
8041	NearestLocation
1218	NearestLocation
8819	NearestLocation
23498	NearestLocation
132061	NearestLocation
56995	NearestLocation
3318	NearestLocation
2934	NearestLocation
1802	NearestLocation
6765	NearestLocation
4743	NearestLocation
1845	NearestLocation
2130	NearestLocation
18475	NearestLocation
3242	NearestLocation
1803	NearestLocation
339	NearestLocation
2184	NearestLocation
720	NearestLocation
3710	NearestLocation
8643	NearestLocation
2393	NearestLocation
3172	NearestLocation
11366	NearestLocation
50094	NearestLocation
2829	NearestLocation
904	NearestLocation
19	NearestLocation
3403	NearestLocation
43	NearestLocation
1193	NearestLocation
1186	NearestLocation
548	NearestLocation
954	NearestLocation
2903	NearestLocation
707	NearestLocation
967	NearestLocation
11174	NearestLocation
3496	NearestLocation
313	NearestLocation
7490	NearestLocation
5097	NearestLocation
26	NearestLocation
7423	NearestLocation
114	NearestLocation
656	NearestLocation
345	NearestLocation
787	NearestLocation
7721	NearestLocation

8856	NearestLocation
585	NearestLocation
1875	NearestLocation
1085	NearestLocation
2912	NearestLocation
494	NearestLocation
225	NearestLocation
3187	NearestLocation
887	NearestLocation
506	NearestLocation
1098	NearestLocation
549	NearestLocation
11149	NearestLocation
207	NearestLocation
12	NearestLocation
1049	NearestLocation
1934	NearestLocation
18850	NearestLocation
1619	NearestLocation
2620	NearestLocation
1286	NearestLocation
