# Supplementary material for: Transcription factor PagLBD21 functions as a repressor of secondary xylem development in Populus
Source: For Res (Fayettev). 2022 Dec 21;2:19. doi: 10.48130/FR-2022-0019 (PMC11524276; doi:10.48130/FR-2022-0019)
Supplement: Supplementary file 1 — Supplementary data to this article can be found online. [file FR-2022-0019-S1.zip › 10.48130_FR-2022-0019-Suppl-TableS5.pdf]

Supplemental Table S5. The overlapped genes between PagLBD21 DAP-seq target genes an

genes	seqnames	start	end	width	strand	score	blockCount	thick.sta
Potri.001	Chr01	5594344	5594619	276	*	196	132	3
Potri.001	Chr01	6611503	6611833	331	*	188	173	4
Potri.001	Chr01	7165158	7165391	234	*	199	135	4
Potri.001	Chr01	9531364	9531614	251	*	214	116	4
Potri.001	Chr01	13980531	13980772	242	*	197	115	3
Potri.001	Chr01	22029190	22029426	237	*	207	152	3
Potri.001	Chr01	34316537	34316817	281	*	208	161	4
Potri.001	Chr01	43036833	43037117	285	*	277	129	4
Potri.001	Chr01	47358494	47358780	287	*	179	177	3
Potri.002	Chr02	3653603	3653899	297	*	190	168	3
Potri.002	Chr02	6295734	6295952	219	*	175	112	5
Potri.002	Chr02	11789392	11789598	207	*	173	105	4
Potri.003	Chr03	13104122	13104423	302	*	185	110	3
Potri.003	Chr03	19295246	19295480	235	*	223	158	4
Potri.003	Chr03	19296783	19297030	248	*	242	115	4
Potri.004	Chr04	6269750	6269989	240	*	286	120	5
Potri.004	Chr04	6955127	6955418	292	*	193	129	4
Potri.004	Chr04	11934926	11935188	263	*	178	109	4
Potri.004	Chr04	12333541	12333764	224	*	204	83	4
Potri.004	Chr04	19238127	19238356	230	*	185	108	3
Potri.005	Chr05	4185325	4185527	203	*	298	99	3
Potri.005	Chr05	23601924	23602134	211	*	175	116	4
Potri.005	Chr05	24083689	24083920	232	*	197	79	4
Potri.006	Chr06	1628583	1628791	209	*	312	82	5
Potri.006	Chr06	4496009	4496255	247	*	253	119	3
Potri.006	Chr06	4530595	4530921	327	*	185	179	3
Potri.006	Chr06	6538315	6538566	252	*	232	88	4
Potri.006	Chr06	19624832	19625138	307	*	237	171	3
Potri.007	Chr07	2418046	2418260	215	*	216	120	4
Potri.007	Chr07	3252335	3252665	331	*	545	163	3
Potri.007	Chr07	12706167	12706396	230	*	335	122	5
Potri.008	Chr08	3222318	3222538	221	*	174	134	3
Potri.008	Chr08	8631360	8631653	294	*	236	164	4
Potri.008	Chr08	10457420	10457640	221	*	208	110	4
Potri.009	Chr09	5494478	5494708	231	*	181	81	4
Potri.009	Chr09	11366307	11367012	706	*	262	578	4
Potri.009	Chr09	11403385	11403734	350	*	314	158	4
Potri.009	Chr09	11793301	11793562	262	*	187	135	4
Potri.010	Chr10	1975920	1976129	210	*	200	125	3
Potri.010	Chr10	11849113	11849384	272	*	208	95	4
Potri.010	Chr10	19930641	19930935	295	*	169	138	3
Potri.011	Chr11	280384	280607	224	*	180	119	5
Potri.012	Chr12	1770217	1770546	330	*	204	177	4
Potri.012	Chr12	12872671	12872880	210	*	303	116	3
Potri.014	Chr14	735918	736142	225	*	181	133	4
Potri.014	Chr14	7117836	7118097	262	*	315	119	6
Potri.015	Chr15	2635540	2635772	233	*	180	104	4
Potri.015	Chr15	4009026	4009277	252	*	233	123	4
Potri.015	Chr15	11995780	11996031	252	*	170	129	4
Potri.017	Chr17	363315	363550	236	*	223	106	5
Potri.017	Chr17	2579352	2579643	292	*	185	138	3

Potri.017	Chr17	9634484	9634763	280	*	179	201	4
Potri.017	Chr17	10937777	10937996	220	*	216	129	4
Potri.017	Chr17	13004792	13005061	270	*	208	157	3
Potri.018	Chr18	772358	772608	251	*	170	156	3
Potri.019	Chr19	11801277	11801588	312	*	281	145	5
Potri.019	Chr19	15371466	15371738	273	*	195	142	3
Potri.T02	scaffold_	174244	174485	242	*	188	105	5
Potri.T11	scaffold_	37809	38018	210	*	221	101	3

ıd DEGs in L36

thick.end	thick.wid	start_pos	end_posit	feature_s	insideFea	distancet	shortestD	fromOverl
19	17	5594633	5596573	+	upstream	-289	14	NearestLo
18	15	6608926	6610764	-	upstream	-739	739	NearestLo
19	16	7162074	7164172	-	upstream	-986	986	NearestLo
21	18	9532286	9534455	-	downstrea	3091	672	NearestLo
19	17	13976442	13981566	-	inside	1035	794	NearestLo
20	18	22029049	22033651	-	inside	4461	141	NearestLo
20	17	34316929	34319074	+	upstream	-392	112	NearestLo
27	24	43033549	43041023	+	inside	3284	3284	NearestLo
17	15	47355398	47356346	-	upstream	-2148	2148	NearestLo
19	17	3654028	3655628	-	downstrea	2025	129	NearestLo
17	13	6300384	6304858	+	upstream	-4650	4432	NearestLo
17	14	11792508	11794803	+	upstream	-3116	2910	NearestLo
18	16	13105319	13107221	+	upstream	-1197	896	NearestLo
22	19	19294354	19294875	+	downstrea	892	371	NearestLo
24	21	19297875	19299786	+	upstream	-1092	845	NearestLo
28	24	6269473	6273663	+	inside	277	277	NearestLo
19	16	6958715	6961637	+	upstream	-3588	3297	NearestLo
17	14	11929544	11932387	-	upstream	-2539	2539	NearestLo
20	17	12337842	12338677	-	downstrea	5136	4078	NearestLo
18	16	19238092	19239534	+	inside	35	35	NearestLo
29	27	4182330	4187153	-	inside	1828	1626	NearestLo
17	14	23600307	23604582	-	inside	2658	1617	NearestLo
19	16	24079656	24082417	-	upstream	-1272	1272	NearestLo
31	27	1626750	1628824	-	inside	241	33	NearestLo
25	23	4499695	4504592	+	upstream	-3686	3440	NearestLo
18	16	4523335	4532993	-	inside	2398	2072	NearestLo
23	20	6538210	6541462	-	inside	3147	105	NearestLo
23	21	19616220	19621043	-	upstream	-3789	3789	NearestLo
21	18	2415499	2419192	-	inside	1146	932	NearestLo
54	52	3252284	3254824	-	inside	2489	51	NearestLo
33	29	12704588	12708177	-	inside	2010	1579	NearestLo
17	15	3220515	3223122	-	inside	804	584	NearestLo
23	20	8631059	8632295	-	inside	935	301	NearestLo
20	17	10457421	10458550	-	overlapEn	1130	1	NearestLo
18	15	5502089	5508765	+	upstream	-7611	7381	NearestLo
26	23	11362951	11367004	-	overlapSt	697	8	NearestLo
31	28	11407300	11410604	+	upstream	-3915	3566	NearestLo
18	15	11783112	11788425	-	upstream	-4876	4876	NearestLo
20	18	1975048	1979416	-	inside	3496	872	NearestLo
20	17	11844016	11848320	-	upstream	-793	793	NearestLo
16	14	19928701	19931559	+	inside	1940	624	NearestLo
18	14	278846	281529	-	inside	1145	922	NearestLo
20	17	1772665	1773882	+	upstream	-2448	2119	NearestLo
30	28	12868554	12869907	-	upstream	-2764	2764	NearestLo
18	15	731206	735001	-	upstream	-917	917	NearestLo
31	26	7118925	7121224	+	upstream	-1089	828	NearestLo
18	15	2637644	2638216	-	downstrea	2676	1872	NearestLo
23	20	4011094	4014183	+	upstream	-2068	1817	NearestLo
17	14	11994376	11996292	+	inside	1404	261	NearestLo
22	18	362085	363948	-	inside	633	398	NearestLo
18	16	2578793	2582511	+	inside	559	559	NearestLo

17	14	9635769	9638227	+	upstream	-1285	1006	NearestLo
21	18	10947583	10953970	+	upstream	-9806	9587	NearestLo
20	18	13012869	13015325	+	upstream	-8077	7808	NearestLo
17	15	766518	772371	-	overlapSt	13	13	NearestLo
28	24	11796617	11800728	-	upstream	-549	549	NearestLo
19	17	15371119	15377454	+	inside	347	347	NearestLo
18	14	182526	191285	+	upstream	-8282	8041	NearestLo
22	20	28580	30088	+	downstrea	9229	7721	NearestLo

logFC	logCPM	PValue	FDR	OX.WT	LLJGY.A	LLJGY.B	LLJGY.C	LLJGY.M
-0.57569	4.831037	0.000181	0.009396	-1	523	371	434	262
0.861806	4.542708	6.10E-09	1.98E-06	1	199	231	211	313
0.886539	4.177162	1.57E-07	3.14E-05	1	157	167	168	308
-0.79283	4.083285	4.34E-08	1.05E-05	-1	286	256	288	159
0.432116	5.133544	0.000788	0.029531	1	360	410	389	458
-0.37485	7.09345	0.00049	0.02034	-1	2302	1879	1854	1374
-0.84924	6.274699	4.63E-08	1.10E-05	-1	1354	1225	1301	684
0.731313	4.44416	2.06E-08	5.72E-06	1	222	196	218	312
-1.1439	2.932477	1.25E-06	0.000174	-1	107	152	139	61
-1.40571	0.45553	0.000515	0.021046	-1	17	25	26	7
-1.48547	1.312806	1.42E-06	0.000193	-1	53	33	47	17
-1.35071	2.892329	3.58E-11	1.99E-08	-1	129	120	156	50
-0.63878	3.297433	0.001102	0.03764	-1	163	169	127	80
0.482309	5.807377	0.000102	0.006109	1	739	516	576	738
0.727536	4.582709	0.000147	0.008053	1	324	156	229	398
0.830755	5.922182	8.48E-11	4.46E-08	1	538	576	583	792
-0.53103	3.695942	0.000524	0.021309	-1	205	199	186	109
0.392011	6.149288	0.000576	0.022931	1	795	752	844	954
-1.08339	1.585227	6.70E-05	0.004491	-1	56	52	42	27
-0.36646	7.261809	0.001098	0.037536	-1	2193	2285	2250	1588
1.574129	8.183179	9.66E-35	2.07E-30	1	2256	1831	1644	5083
-0.81174	2.05911	0.000321	0.014773	-1	76	61	62	28
-0.72692	3.806899	0.000199	0.010165	-1	214	227	230	161
-1.33382	0.308012	0.001074	0.037077	-1	26	20	14	9
-1.01221	1.676076	0.00064	0.02506	-1	61	48	49	31
-0.44256	5.182526	0.001113	0.037852	-1	674	473	489	322
0.398711	8.035484	0.001092	0.037453	1	3401	2368	3108	3606
-0.38945	8.085736	0.000235	0.011615	-1	4050	4065	3896	2746
0.924785	3.357035	1.93E-07	3.72E-05	1	94	81	98	179
0.825414	2.160488	0.000365	0.01624	1	38	35	49	60
0.579898	4.28217	9.36E-05	0.005716	1	214	169	223	295
0.353074	7.892937	0.001375	0.043948	1	2667	2820	2643	2963
0.776973	10.20572	4.96E-08	1.16E-05	1	13053	11655	9380	20509
-0.66442	3.230829	9.30E-05	0.005702	-1	170	145	127	74
0.663261	3.844056	0.000128	0.007221	1	136	172	121	208
-0.7956	2.217801	0.000284	0.013415	-1	80	70	72	27
-0.42169	5.289645	0.000997	0.035168	-1	606	582	553	408
0.426847	7.105002	0.000452	0.019326	1	1729	1340	1529	1690
-0.77616	2.012043	0.001211	0.040141	-1	81	59	51	30
-0.97079	4.153341	3.96E-10	1.79E-07	-1	296	324	288	145
0.488105	3.947327	0.001198	0.039872	1	184	158	156	223
-0.92493	2.100934	0.000181	0.009403	-1	63	60	87	30
-0.88689	3.038988	4.51E-06	0.000501	-1	123	144	139	65
-1.00703	2.186514	0.000483	0.020161	-1	56	67	104	38
-0.7715	6.525677	3.11E-05	0.00244	-1	1481	1328	1715	884
-1.48352	2.996196	8.04E-08	1.76E-05	-1	107	183	154	61
0.785063	2.410629	0.00122	0.040267	1	54	44	51	92
-1.93473	2.126491	2.45E-12	1.81E-09	-1	72	106	79	22
-1.12086	3.074609	0.000165	0.00871	-1	243	94	110	72
-1.35149	2.40108	4.06E-07	6.91E-05	-1	107	103	76	49
0.490713	4.650171	7.18E-05	0.004733	1	303	253	257	331

0.505384	4.041594	0.00031	0.01439	1	207	166	156	221
0.38167	7.098138	0.001073	0.03707	1	1473	1509	1649	1766
0.914815	9.743442	2.12E-17	4.55E-14	1	7821	7382	7982	13789
-1.66886	0.130396	0.000123	0.006997	-1	15	19	21	5
-0.68445	8.091765	2.18E-07	4.12E-05	-1	5460	3398	4369	2398
0.421916	6.751264	0.00011	0.006406	1	1321	1104	1175	1322
1.070686	2.31681	1.31E-06	0.000181	1	39	51	32	76
-2.76475	2.736297	2.65E-28	2.27E-24	-1	151	159	128	19

LLJGY.N	LLJGY.O	Best.hit.arabi.sym	arabi.defline
326	241	AT5G32450.1	RNA binding (RRM/RBD/RNP motifs) family prote
350	437	AT1G14430.1	glyoxal oxidase-related protein
305	237	AT4G23900.1	Nucleoside diphosphate kinase family protein
143	146	AT4G12350AtMYB42,M	myb domain protein 42
486	528	AT1G72480.1	Lung seven transmembrane receptor family prot
1416	1562	AT2G35940BLH1,EDA2	BEL1-like homeodomain 1
797	525	AT3G27400.1	Pectin lyase-like superfamily protein
346	331	AT5G54250ATCNGC4,C	cyclic nucleotide-gated cation channel 4
64	44	AT5G44550.1	Uncharacterised protein family (UPF0497)
5	12	AT1G03220.1	Eukaryotic aspartyl protease family protein
17	10	AT1G77760GNR1,NIA1	nitrate reductase 1
40	59	AT2G37360.1	ABC-2 type transporter family protein
114	82	AT4G39730.1	Lipase/lipooxygenase, PLAT/LH2 family protein
767	884	AT1G64660ATMGL,MGL	methionine gamma-lyase
331	356	AT1G64660ATMGL,MGL	methionine gamma-lyase
1012	1042	AT5G17330GAD,GAD1	glutamate decarboxylase
144	130	AT1G66140ZFP4	zinc finger protein 4
970	1019	AT5G08060.1	
16	23	AT3G28917MIF2	mini zinc finger 2
1672	1638	AT2G16700ADF5,ATAD	actin depolymerizing factor 5
4664	6224	AT5G24550BGLU32	beta glucosidase 32
42	36	AT4G28530anac074,N	NAC domain containing protein 74
112	105	AT1G19715.3	Mannose-binding lectin superfamily protein
6	7	AT1G22400ATUGT85A1	UDP-Glycosyltransferase superfamily protein
12	30	AT1G58190AtRLP9,RL	receptor like protein 9
379	424	AT1G15740.1	Leucine-rich repeat family protein
3514	3793	AT2G37640ATEXP3,AT	Barwin-like endoglucanases superfamily protei
2746	3122	AT5G17420ATCESA7,C	Cellulose synthase family protein
137	169	AT4G36850.1	PQ-loop repeat family protein / transmembrane
79	64	AT2G26560PLA IIA,P	phospholipase A 2A
280	271	AT2G04160AIR3	Subtilisin-like serine endopeptidase family p
3352	3453	AT5G02500AT-HSC70-	heat shock cognate protein 70-1
16798	17000	AT1G14930.1	Polyketide cyclase/dehydrase and lipid transp
95	92	AT3G22540.1	Protein of unknown function (DUF1677)
226	204	AT1G50450.1	Saccharopine dehydrogenase
46	47	AT1G20550.1	O-fucosyltransferase family protein
436	371	AT1G08290WIP3	WIP domain protein 3
1787	2325	AT5G22740ATCSLA02,	cellulose synthase-like A02
39	35	AT4G21380ARK3,RK3	receptor kinase 3
159	130	AT1G70260.1	nodulin MtN21 /EamA-like transporter family p
231	198	AT3G55330PPL1	PsbP-like protein 1
31	43	AT5G38280PR5K	PR5-like receptor kinase
80	61	AT3G24770CLE41	CLAVATA3/ESR-RELATED 41
38	30	AT1G24140.1	Matrixin family protein
989	593	AT3G49780ATPSK3 (F	phytosulfokine 4 precursor
52	36	AT2G46130ATWRKY43,	WRKY DNA-binding protein 43
88	59	AT3G28920AtHB34,HB	homeobox protein 34
23	18	AT3G18170.1	Glycosyltransferase family 61 protein
59	57	AT5G50790.1	Nodulin MtN3 family protein
27	28	AT3G59850.1	Pectin lyase-like superfamily protein
352	387	AT4G22720.1	Actin-like ATPase superfamily protein

240	241	AT5G15150	ATHB-3,AT	homeobox 3
1801	2114	AT3G02090	MPPBETA	Insulinase (Peptidase family M16) protein
13445	13700	AT1G67090	RBCS1A	ribulose bisphosphate carboxylase small chain
6	5	AT2G29110	ATGLR2.8,	glutamate receptor 2.8
2583	2679	AT1G22710	ATSUC2,SU	sucrose-proton symporter 2
1558	1642	AT1G06950	ATTIC110,	translocon at the inner envelope membrane of
82	83	AT4G27290.1		S-locus lectin protein kinase family protein
29	12	AT1G24430.1		HXXXD-type acyl-transferase family protein

ein

tein

n

n

family protein

protein

port superfamily protein

protein

ı 1A

chloroplasts 110
