# Supplementary material for: Transcription factor PagLBD21 functions as a repressor of secondary xylem development in Populus
Source: For Res (Fayettev). 2022 Dec 21;2:19. doi: 10.48130/FR-2022-0019 (PMC11524276; doi:10.48130/FR-2022-0019)
Supplement: Supplementary file 1 — Supplementary data to this article can be found online. [file FR-2022-0019-S1.zip › 10.48130_FR-2022-0019-Suppl-TableS6.pdf]

Supplemental Table S6. The overlapped target genes in PagLBD21 DAP-seq & PagLBD3 D

genes	seqnames.	start.x	end.x	width.x	strand.x	score	blockCoun	thick.sta
Potri.001	Chr01	929145	929398	254	*	215	139	5
Potri.001	Chr01	3620015	3620238	224	*	269	126	4
Potri.001	Chr01	3620015	3620238	224	*	269	126	4
Potri.001	Chr01	3620015	3620238	224	*	269	126	4
Potri.001	Chr01	3657896	3658137	242	*	176	152	4
Potri.001	Chr01	4424567	4425087	521	*	446	124	3
Potri.001	Chr01	4817439	4817822	384	*	178	195	3
Potri.001	Chr01	5083634	5083924	291	*	572	155	4
Potri.001	Chr01	5164245	5164447	203	*	177	88	4
Potri.001	Chr01	5518387	5518682	296	*	212	125	5
Potri.001	Chr01	5594344	5594619	276	*	196	132	3
Potri.001	Chr01	5713056	5713314	259	*	172	121	4
Potri.001	Chr01	5713056	5713314	259	*	172	121	4
Potri.001	Chr01	6538430	6538652	223	*	249	101	3
Potri.001	Chr01	6538430	6538652	223	*	249	101	3
Potri.001	Chr01	7118166	7118428	263	*	243	122	5
Potri.001	Chr01	7118166	7118428	263	*	243	122	5
Potri.001	Chr01	7202933	7203201	269	*	192	159	4
Potri.001	Chr01	7202933	7203201	269	*	192	159	4
Potri.001	Chr01	7202933	7203201	269	*	192	159	4
Potri.001	Chr01	9211692	9211926	235	*	192	84	4
Potri.001	Chr01	9499008	9499299	292	*	316	128	5
Potri.001	Chr01	9856180	9856473	294	*	174	107	3
Potri.001	Chr01	9965471	9965676	206	*	224	95	3
Potri.001	Chr01	11491617	11491855	239	*	363	95	2
Potri.001	Chr01	11563680	11564504	825	*	204	407	2
Potri.001	Chr01	12720296	12720501	206	*	181	110	4
Potri.001	Chr01	13557019	13557261	243	*	205	124	4
Potri.001	Chr01	15234734	15234982	249	*	346	128	5
Potri.001	Chr01	15234734	15234982	249	*	346	128	5
Potri.001	Chr01	15637732	15637979	248	*	178	144	4
Potri.001	Chr01	16181800	16182105	306	*	191	118	3
Potri.001	Chr01	17037292	17037893	602	*	169	456	3
Potri.001	Chr01	17649304	17649878	575	*	419	424	2
Potri.001	Chr01	18787754	18787961	208	*	273	105	3
Potri.001	Chr01	18838615	18838831	217	*	213	129	5
Potri.001	Chr01	19166562	19166793	232	*	169	83	5
Potri.001	Chr01	19852116	19852329	214	*	293	109	3
Potri.001	Chr01	20657701	20657911	211	*	248	104	3
Potri.001	Chr01	20863611	20863873	263	*	230	118	5
Potri.001	Chr01	20949693	20950171	479	*	340	125	4
Potri.001	Chr01	21094922	21095165	244	*	224	115	3
Potri.001	Chr01	21735628	21735845	218	*	264	100	5
Potri.001	Chr01	21735628	21735845	218	*	264	100	5
Potri.001	Chr01	22367111	22367349	239	*	235	117	4
Potri.001	Chr01	22842446	22842664	219	*	230	143	4
Potri.001	Chr01	23053466	23054213	748	*	168	374	3
Potri.001	Chr01	24554688	24555000	313	*	244	154	5
Potri.001	Chr01	25022733	25023209	477	*	351	335	4
Potri.001	Chr01	26612929	26613158	230	*	530	122	3
Potri.001	Chr01	27822169	27822501	333	*	349	159	5

Potri.001	Chr01	27821222	27821426	205	*	175	106	4
Potri.001	Chr01	28202924	28203134	211	*	445	76	3
Potri.001	Chr01	28204445	28204663	219	*	175	108	5
Potri.001	Chr01	28658989	28659248	260	*	193	166	4
Potri.001	Chr01	28984782	28985001	220	*	175	109	5
Potri.001	Chr01	29390817	29391164	348	*	294	153	2
Potri.001	Chr01	29742882	29743098	217	*	228	87	5
Potri.001	Chr01	31448791	31449151	361	*	203	144	3
Potri.001	Chr01	33457592	33457805	214	*	342	98	3
Potri.001	Chr01	33570581	33570838	258	*	181	128	3
Potri.001	Chr01	33976105	33976353	249	*	171	84	3
Potri.001	Chr01	34316537	34316817	281	*	208	161	4
Potri.001	Chr01	34316537	34316817	281	*	208	161	4
Potri.001	Chr01	36668052	36668510	459	*	186	189	3
Potri.001	Chr01	36719265	36719591	327	*	187	177	3
Potri.001	Chr01	36716687	36716963	277	*	179	137	3
Potri.001	Chr01	36773988	36774280	293	*	179	119	3
Potri.001	Chr01	38900271	38900501	231	*	306	164	6
Potri.001	Chr01	40123073	40123282	210	*	322	75	3
Potri.001	Chr01	40943679	40943908	230	*	175	118	5
Potri.001	Chr01	41015949	41016244	296	*	527	145	3
Potri.001	Chr01	41352508	41352709	202	*	175	107	4
Potri.001	Chr01	41633636	41633870	235	*	415	116	3
Potri.001	Chr01	41805493	41805830	338	*	232	172	5
Potri.001	Chr01	41805493	41805830	338	*	232	172	5
Potri.001	Chr01	41805493	41805830	338	*	232	172	5
Potri.001	Chr01	42021359	42021841	483	*	216	362	4
Potri.001	Chr01	43036833	43037117	285	*	277	129	4
Potri.001	Chr01	43727465	43727918	454	*	187	298	2
Potri.001	Chr01	43727465	43727918	454	*	187	298	2
Potri.001	Chr01	43727465	43727918	454	*	187	298	2
Potri.001	Chr01	43726680	43726996	317	*	361	182	2
Potri.001	Chr01	43726680	43726996	317	*	361	182	2
Potri.001	Chr01	43726680	43726996	317	*	361	182	2
Potri.001	Chr01	45718959	45719178	220	*	462	114	3
Potri.001	Chr01	45718959	45719178	220	*	462	114	3
Potri.001	Chr01	45811997	45812279	283	*	214	109	3
Potri.001	Chr01	45804776	45804979	204	*	243	88	3
Potri.001	Chr01	47427294	47427647	354	*	299	173	4
Potri.001	Chr01	47427294	47427647	354	*	299	173	4
Potri.001	Chr01	47427294	47427647	354	*	299	173	4
Potri.001	Chr01	48291364	48291605	242	*	200	159	4
Potri.001	Chr01	48748860	48749093	234	*	303	113	5
Potri.001	Chr01	48821696	48822085	390	*	239	100	3
Potri.001	Chr01	49440659	49440895	237	*	173	106	4
Potri.001	Chr01	49657251	49657503	253	*	249	132	3
Potri.002	Chr02	242397	242811	415	*	199	247	3
Potri.002	Chr02	1264017	1264278	262	*	259	113	3
Potri.002	Chr02	1270379	1270641	263	*	204	148	3
Potri.002	Chr02	1762232	1762521	290	*	215	142	3
Potri.002	Chr02	1821836	1822077	242	*	187	144	4
Potri.002	Chr02	1824675	1824915	241	*	199	107	4
Potri.002	Chr02	1828043	1828250	208	*	343	111	3
Potri.002	Chr02	2564367	2564632	266	*	174	135	3

Potri.002	Chr02	2560178	2560385	208	*	338	122	3
Potri.002	Chr02	2802450	2802702	253	*	267	134	4
Potri.002	Chr02	2804876	2805217	342	*	674	180	5
Potri.002	Chr02	3259870	3260141	272	*	269	131	4
Potri.002	Chr02	3484213	3484690	478	*	412	166	4
Potri.002	Chr02	3484213	3484690	478	*	412	166	4
Potri.002	Chr02	3668732	3670070	1339	*	189	963	3
Potri.002	Chr02	3668732	3670070	1339	*	189	963	3
Potri.002	Chr02	3751194	3751509	316	*	387	154	2
Potri.002	Chr02	3782545	3782813	269	*	208	117	4
Potri.002	Chr02	3848902	3849499	598	*	286	176	3
Potri.002	Chr02	4997837	4998038	202	*	216	108	4
Potri.002	Chr02	5524404	5524641	238	*	461	116	3
Potri.002	Chr02	5973654	5974021	368	*	252	168	4
Potri.002	Chr02	6140066	6140285	220	*	358	104	3
Potri.002	Chr02	6760361	6760631	271	*	171	133	3
Potri.002	Chr02	6760361	6760631	271	*	171	133	3
Potri.002	Chr02	6760361	6760631	271	*	171	133	3
Potri.002	Chr02	6805103	6805326	224	*	170	113	4
Potri.002	Chr02	6814253	6814484	232	*	249	126	4
Potri.002	Chr02	6814253	6814484	232	*	249	126	4
Potri.002	Chr02	7002606	7002901	296	*	260	133	4
Potri.002	Chr02	7145316	7145525	210	*	191	107	4
Potri.002	Chr02	7838964	7839252	289	*	195	133	4
Potri.002	Chr02	7838964	7839252	289	*	195	133	4
Potri.002	Chr02	8024870	8025102	233	*	264	100	4
Potri.002	Chr02	8322259	8322462	204	*	203	88	3
Potri.002	Chr02	8322259	8322462	204	*	203	88	3
Potri.002	Chr02	9176993	9177632	640	*	168	483	3
Potri.002	Chr02	9176993	9177632	640	*	168	483	3
Potri.002	Chr02	10237921	10238198	278	*	194	162	2
Potri.002	Chr02	10425607	10425861	255	*	168	135	3
Potri.002	Chr02	10485049	10485377	329	*	197	188	4
Potri.002	Chr02	11789392	11789598	207	*	173	105	4
Potri.002	Chr02	12458308	12458509	202	*	224	92	3
Potri.002	Chr02	13302694	13303015	322	*	183	166	4
Potri.002	Chr02	13622351	13622645	295	*	214	152	4
Potri.002	Chr02	14003251	14003537	287	*	261	151	5
Potri.002	Chr02	14263568	14263812	245	*	461	117	3
Potri.002	Chr02	15618534	15618824	291	*	721	140	3
Potri.002	Chr02	15786299	15786595	297	*	312	133	5
Potri.002	Chr02	16766241	16766604	364	*	197	264	3
Potri.002	Chr02	16836837	16837061	225	*	223	160	4
Potri.002	Chr02	17125547	17125757	211	*	278	107	3
Potri.002	Chr02	17651204	17651413	210	*	391	94	3
Potri.002	Chr02	17651204	17651413	210	*	391	94	3
Potri.002	Chr02	17651204	17651413	210	*	391	94	3
Potri.002	Chr02	17651204	17651413	210	*	391	94	3
Potri.002	Chr02	17657454	17657718	265	*	637	138	3
Potri.002	Chr02	17657454	17657718	265	*	637	138	3
Potri.002	Chr02	17657454	17657718	265	*	637	138	3
Potri.002	Chr02	17657454	17657718	265	*	637	138	3
Potri.002	Chr02	17652906	17653166	261	*	733	130	3
Potri.002	Chr02	17652906	17653166	261	*	733	130	3

Potri.002Chr02	17652906	17653166	261	*	733	130	3
Potri.002Chr02	17652906	17653166	261	*	733	130	3
Potri.002Chr02	17652008	17652213	206	*	198	129	3
Potri.002Chr02	17652008	17652213	206	*	198	129	3
Potri.002Chr02	17652008	17652213	206	*	198	129	3
Potri.002Chr02	17652008	17652213	206	*	198	129	3
Potri.002Chr02	17813769	17813982	214	*	323	108	3
Potri.002Chr02	18302835	18303053	219	*	300	126	3
Potri.002Chr02	18356166	18356462	297	*	255	155	2
Potri.002Chr02	18356166	18356462	297	*	255	155	2
Potri.002Chr02	18356166	18356462	297	*	255	155	2
Potri.002Chr02	18356166	18356462	297	*	255	155	2
Potri.002Chr02	18356166	18356462	297	*	255	155	2
Potri.002Chr02	18356166	18356462	297	*	255	155	2
Potri.002Chr02	18356166	18356462	297	*	255	155	2
Potri.002Chr02	18356166	18356462	297	*	255	155	2
Potri.002Chr02	18356166	18356462	297	*	255	155	2
Potri.002Chr02	18356166	18356462	297	*	255	155	2
Potri.002Chr02	18353041	18353822	782	*	189	145	2
Potri.002Chr02	18353041	18353822	782	*	189	145	2
Potri.002Chr02	18353041	18353822	782	*	189	145	2
Potri.002Chr02	18353041	18353822	782	*	189	145	2
Potri.002Chr02	18353041	18353822	782	*	189	145	2
Potri.002Chr02	18353041	18353822	782	*	189	145	2
Potri.002Chr02	18353041	18353822	782	*	189	145	2
Potri.002Chr02	18353041	18353822	782	*	189	145	2
Potri.002Chr02	18353041	18353822	782	*	189	145	2
Potri.002Chr02	18353041	18353822	782	*	189	145	2
Potri.002Chr02	18357968	18358646	679	*	206	436	2
Potri.002Chr02	18357968	18358646	679	*	206	436	2
Potri.002Chr02	18357968	18358646	679	*	206	436	2
Potri.002Chr02	18357968	18358646	679	*	206	436	2
Potri.002Chr02	18357968	18358646	679	*	206	436	2
Potri.002Chr02	18357968	18358646	679	*	206	436	2
Potri.002Chr02	18357968	18358646	679	*	206	436	2
Potri.002Chr02	18357968	18358646	679	*	206	436	2
Potri.002Chr02	18357968	18358646	679	*	206	436	2
Potri.002Chr02	18357968	18358646	679	*	206	436	2
Potri.002Chr02	18351111	18351350	240	*	202	134	2
Potri.002Chr02	18351111	18351350	240	*	202	134	2
Potri.002Chr02	18351111	18351350	240	*	202	134	2
Potri.002Chr02	18351111	18351350	240	*	202	134	2
Potri.002Chr02	18351111	18351350	240	*	202	134	2
Potri.002Chr02	18351111	18351350	240	*	202	134	2
Potri.002Chr02	18351111	18351350	240	*	202	134	2
Potri.002Chr02	18351111	18351350	240	*	202	134	2
Potri.002Chr02	18351111	18351350	240	*	202	134	2
Potri.002Chr02	18351111	18351350	240	*	202	134	2
Potri.002Chr02	18356805	18357546	742	*	263	239	2
Potri.002Chr02	18356805	18357546	742	*	263	239	2
Potri.002Chr02	18356805	18357546	742	*	263	239	2
Potri.002Chr02	18356805	18357546	742	*	263	239	2
Potri.002Chr02	18356805	18357546	742	*	263	239	2
Potri.002Chr02	18356805	18357546	742	*	263	239	2

Potri.002	Chr02	18356805	18357546	742	*	263	239	2
Potri.002	Chr02	18356805	18357546	742	*	263	239	2
Potri.002	Chr02	18356805	18357546	742	*	263	239	2
Potri.002	Chr02	18356805	18357546	742	*	263	239	2
Potri.002	Chr02	18348957	18349990	1034	*	208	862	2
Potri.002	Chr02	18348957	18349990	1034	*	208	862	2
Potri.002	Chr02	18348957	18349990	1034	*	208	862	2
Potri.002	Chr02	18348957	18349990	1034	*	208	862	2
Potri.002	Chr02	18348957	18349990	1034	*	208	862	2
Potri.002	Chr02	18348957	18349990	1034	*	208	862	2
Potri.002	Chr02	18348957	18349990	1034	*	208	862	2
Potri.002	Chr02	18348957	18349990	1034	*	208	862	2
Potri.002	Chr02	18348957	18349990	1034	*	208	862	2
Potri.002	Chr02	18348957	18349990	1034	*	208	862	2
Potri.002	Chr02	18354165	18354631	467	*	213	160	2
Potri.002	Chr02	18354165	18354631	467	*	213	160	2
Potri.002	Chr02	18354165	18354631	467	*	213	160	2
Potri.002	Chr02	18354165	18354631	467	*	213	160	2
Potri.002	Chr02	18354165	18354631	467	*	213	160	2
Potri.002	Chr02	18354165	18354631	467	*	213	160	2
Potri.002	Chr02	18354165	18354631	467	*	213	160	2
Potri.002	Chr02	18354165	18354631	467	*	213	160	2
Potri.002	Chr02	18354165	18354631	467	*	213	160	2
Potri.002	Chr02	18354165	18354631	467	*	213	160	2
Potri.002	Chr02	18363354	18364058	705	*	222	538	2
Potri.002	Chr02	18365231	18368232	3002	*	179	2578	2
Potri.002	Chr02	18365231	18368232	3002	*	179	2578	2
Potri.002	Chr02	18368445	18368681	237	*	267	97	2
Potri.002	Chr02	18368445	18368681	237	*	267	97	2
Potri.002	Chr02	18378206	18379838	1633	*	251	666	2
Potri.002	Chr02	18378206	18379838	1633	*	251	666	2
Potri.002	Chr02	18377661	18378000	340	*	212	247	2
Potri.002	Chr02	18377661	18378000	340	*	212	247	2
Potri.002	Chr02	18386656	18388425	1770	*	276	187	2
Potri.002	Chr02	18386656	18388425	1770	*	276	187	2
Potri.002	Chr02	18386656	18388425	1770	*	276	187	2
Potri.002	Chr02	18388713	18389284	572	*	184	235	2
Potri.002	Chr02	18388713	18389284	572	*	184	235	2
Potri.002	Chr02	19550541	19550809	269	*	178	145	4
Potri.002	Chr02	20470878	20471281	404	*	181	100	3
Potri.002	Chr02	20569255	20569478	224	*	324	116	4
Potri.002	Chr02	20569255	20569478	224	*	324	116	4
Potri.002	Chr02	21172016	21172253	238	*	304	128	5
Potri.002	Chr02	21546356	21546637	282	*	188	139	5
Potri.002	Chr02	21819525	21819731	207	*	176	97	4
Potri.002	Chr02	21925698	21925951	254	*	277	103	5
Potri.002	Chr02	22768066	22768274	209	*	259	131	3
Potri.002	Chr02	23764535	23764747	213	*	169	112	5
Potri.002	Chr02	23764535	23764747	213	*	169	112	5
Potri.002	Chr02	23906127	23906472	346	*	271	148	4
Potri.002	Chr02	23906127	23906472	346	*	271	148	4
Potri.002	Chr02	23906127	23906472	346	*	271	148	4
Potri.002	Chr02	23906127	23906472	346	*	271	148	4
Potri.002	Chr02	24751796	24752101	306	*	190	141	3

Potri.002	Chr02	25138468	25138735	268	*	185	145	4
Potri.002	Chr02	25138468	25138735	268	*	185	145	4
Potri.003	Chr03	63265	63586	322	*	220	191	4
Potri.003	Chr03	63265	63586	322	*	220	191	4
Potri.003	Chr03	61971	62230	260	*	266	150	6
Potri.003	Chr03	61971	62230	260	*	266	150	6
Potri.003	Chr03	656114	656315	202	*	231	93	3
Potri.003	Chr03	2551852	2552448	597	*	213	150	3
Potri.003	Chr03	2551852	2552448	597	*	213	150	3
Potri.003	Chr03	3022246	3022458	213	*	208	125	3
Potri.003	Chr03	3375111	3375353	243	*	177	125	4
Potri.003	Chr03	3376699	3377001	303	*	269	158	4
Potri.003	Chr03	4755672	4755880	209	*	240	121	4
Potri.003	Chr03	4755672	4755880	209	*	240	121	4
Potri.003	Chr03	6423038	6423278	241	*	192	112	4
Potri.003	Chr03	9386882	9387094	213	*	543	83	3
Potri.003	Chr03	9386882	9387094	213	*	543	83	3
Potri.003	Chr03	9386882	9387094	213	*	543	83	3
Potri.003	Chr03	9386042	9386341	300	*	842	168	3
Potri.003	Chr03	9386042	9386341	300	*	842	168	3
Potri.003	Chr03	9386042	9386341	300	*	842	168	3
Potri.003	Chr03	9387423	9387964	542	*	647	404	3
Potri.003	Chr03	9387423	9387964	542	*	647	404	3
Potri.003	Chr03	9387423	9387964	542	*	647	404	3
Potri.003	Chr03	9388515	9388716	202	*	297	119	3
Potri.003	Chr03	9388515	9388716	202	*	297	119	3
Potri.003	Chr03	9389169	9389542	374	*	418	205	2
Potri.003	Chr03	9389169	9389542	374	*	418	205	2
Potri.003	Chr03	9567962	9568296	335	*	420	171	2
Potri.003	Chr03	9568491	9568707	217	*	218	57	2
Potri.003	Chr03	9569214	9569530	317	*	555	149	3
Potri.003	Chr03	10182079	10182321	243	*	510	137	3
Potri.003	Chr03	10535089	10535357	269	*	168	121	3
Potri.003	Chr03	12735666	12735888	223	*	215	109	3
Potri.003	Chr03	12735666	12735888	223	*	215	109	3
Potri.003	Chr03	13043932	13044184	253	*	175	99	3
Potri.003	Chr03	13043932	13044184	253	*	175	99	3
Potri.003	Chr03	13055116	13055438	323	*	237	136	4
Potri.003	Chr03	13104122	13104423	302	*	185	110	3
Potri.003	Chr03	13158324	13158581	258	*	171	145	3
Potri.003	Chr03	13183531	13183972	442	*	183	167	3
Potri.003	Chr03	13697484	13697776	293	*	232	176	3
Potri.003	Chr03	14060435	14060677	243	*	215	153	5
Potri.003	Chr03	15333864	15334244	381	*	173	182	3
Potri.003	Chr03	15380497	15381719	1223	*	228	1102	3
Potri.003	Chr03	15553791	15554016	226	*	202	98	5
Potri.003	Chr03	15715390	15715627	238	*	224	110	5
Potri.003	Chr03	15837723	15837925	203	*	319	104	2
Potri.003	Chr03	16382870	16383080	211	*	170	84	4
Potri.003	Chr03	16382870	16383080	211	*	170	84	4
Potri.003	Chr03	16625715	16625946	232	*	168	116	3
Potri.003	Chr03	16699772	16700025	254	*	381	139	3
Potri.003	Chr03	17015196	17015423	228	*	193	132	4
Potri.003	Chr03	17595004	17595214	211	*	193	129	5

Potri.003	Chr03	17595004	17595214	211	*	193	129	5
Potri.003	Chr03	17956708	17956951	244	*	242	141	4
Potri.003	Chr03	17956708	17956951	244	*	242	141	4
Potri.003	Chr03	18363124	18363365	242	*	195	119	5
Potri.003	Chr03	18772456	18772727	272	*	290	110	5
Potri.003	Chr03	19533971	19534444	474	*	212	176	2
Potri.003	Chr03	20194417	20194929	513	*	227	401	3
Potri.003	Chr03	20194417	20194929	513	*	227	401	3
Potri.003	Chr03	20297536	20297754	219	*	539	92	4
Potri.003	Chr03	20297536	20297754	219	*	539	92	4
Potri.003	Chr03	20350203	20350446	244	*	216	141	4
Potri.003	Chr03	20350203	20350446	244	*	216	141	4
Potri.003	Chr03	20447696	20448330	635	*	285	241	3
Potri.003	Chr03	20657367	20657609	243	*	168	133	4
Potri.003	Chr03	20657367	20657609	243	*	168	133	4
Potri.003	Chr03	20657367	20657609	243	*	168	133	4
Potri.003	Chr03	21512332	21512557	226	*	214	110	4
Potri.003	Chr03	21527106	21527351	246	*	171	131	3
Potri.004	Chr04	811231	811469	239	*	225	101	4
Potri.004	Chr04	1297190	1297593	404	*	210	174	2
Potri.004	Chr04	2803076	2803297	222	*	170	125	4
Potri.004	Chr04	2803076	2803297	222	*	170	125	4
Potri.004	Chr04	3420787	3420993	207	*	229	104	5
Potri.004	Chr04	3801955	3802294	340	*	181	122	4
Potri.004	Chr04	3869416	3869634	219	*	276	54	3
Potri.004	Chr04	4190011	4190218	208	*	247	103	4
Potri.004	Chr04	5234097	5234310	214	*	188	94	5
Potri.004	Chr04	6269750	6269989	240	*	286	120	5
Potri.004	Chr04	6778391	6778660	270	*	189	148	3
Potri.004	Chr04	8208761	8209273	513	*	180	125	3
Potri.004	Chr04	8369143	8369385	243	*	187	132	4
Potri.004	Chr04	8369143	8369385	243	*	187	132	4
Potri.004	Chr04	8367999	8368237	239	*	168	99	4
Potri.004	Chr04	8367999	8368237	239	*	168	99	4
Potri.004	Chr04	9495749	9495958	210	*	296	97	4
Potri.004	Chr04	9503758	9504004	247	*	330	100	2
Potri.004	Chr04	10315252	10315464	213	*	371	91	3
Potri.004	Chr04	11934926	11935188	263	*	178	109	4
Potri.004	Chr04	12472419	12472824	406	*	365	164	2
Potri.004	Chr04	12472419	12472824	406	*	365	164	2
Potri.004	Chr04	12615952	12616166	215	*	203	110	4
Potri.004	Chr04	13200702	13201094	393	*	656	234	3
Potri.004	Chr04	14359023	14359340	318	*	375	145	2
Potri.004	Chr04	14359023	14359340	318	*	375	145	2
Potri.004	Chr04	14359023	14359340	318	*	375	145	2
Potri.004	Chr04	14359023	14359340	318	*	375	145	2
Potri.004	Chr04	14359023	14359340	318	*	375	145	2
Potri.004	Chr04	14359023	14359340	318	*	375	145	2
Potri.004	Chr04	14458094	14458306	213	*	405	86	3
Potri.004	Chr04	14899532	14899829	298	*	308	141	5
Potri.004	Chr04	16138759	16138967	209	*	346	80	3
Potri.004	Chr04	16138759	16138967	209	*	346	80	3
Potri.004	Chr04	16761882	16762123	242	*	246	144	4
Potri.004	Chr04	16761882	16762123	242	*	246	144	4

Potri.004Chr04	17750789	17751016	228	*	222	103	5
Potri.004Chr04	18158915	18159208	294	*	183	135	4
Potri.004Chr04	18526921	18527341	421	*	189	146	3
Potri.004Chr04	18516299	18516521	223	*	193	99	4
Potri.004Chr04	18677754	18678269	516	*	282	87	3
Potri.004Chr04	18676692	18677475	784	*	281	345	3
Potri.004Chr04	19046660	19047077	418	*	268	142	3
Potri.004Chr04	19650423	19650642	220	*	264	138	3
Potri.004Chr04	19650423	19650642	220	*	264	138	3
Potri.004Chr04	19650423	19650642	220	*	264	138	3
Potri.004Chr04	20206515	20206852	338	*	308	192	3
Potri.004Chr04	20356490	20356837	348	*	288	156	5
Potri.004Chr04	20857561	20857830	270	*	195	143	3
Potri.004Chr04	20887728	20888016	289	*	576	145	3
Potri.004Chr04	21399320	21399645	326	*	206	173	3
Potri.004Chr04	22883573	22883870	298	*	355	146	4
Potri.004Chr04	24170720	24170957	238	*	442	122	3
Potri.005Chr05	350362	350582	221	*	170	118	4
Potri.005Chr05	1855786	1856194	409	*	319	101	3
Potri.005Chr05	1855786	1856194	409	*	319	101	3
Potri.005Chr05	1855786	1856194	409	*	319	101	3
Potri.005Chr05	1879402	1880096	695	*	367	545	3
Potri.005Chr05	4183983	4184419	437	*	218	88	2
Potri.005Chr05	4183060	4183540	481	*	176	138	2
Potri.005Chr05	4535382	4535603	222	*	175	101	4
Potri.005Chr05	5598685	5598923	239	*	262	99	4
Potri.005Chr05	5688719	5688992	274	*	187	137	4
Potri.005Chr05	6352092	6352350	259	*	226	122	4
Potri.005Chr05	6476890	6477153	264	*	252	134	4
Potri.005Chr05	6900151	6900459	309	*	644	164	3
Potri.005Chr05	7165603	7165814	212	*	181	76	3
Potri.005Chr05	7378513	7378782	270	*	268	117	5
Potri.005Chr05	8404898	8405386	489	*	210	408	3
Potri.005Chr05	11607255	11607540	286	*	225	141	4
Potri.005Chr05	11709073	11709388	316	*	429	188	4
Potri.005Chr05	12279601	12279828	228	*	209	123	5
Potri.005Chr05	12743932	12744150	219	*	397	117	3
Potri.005Chr05	12743932	12744150	219	*	397	117	3
Potri.005Chr05	12752045	12752250	206	*	168	94	4
Potri.005Chr05	13493411	13493638	228	*	587	124	3
Potri.005Chr05	13504710	13504941	232	*	309	119	3
Potri.005Chr05	13504710	13504941	232	*	309	119	3
Potri.005Chr05	13504710	13504941	232	*	309	119	3
Potri.005Chr05	13504710	13504941	232	*	309	119	3
Potri.005Chr05	13510901	13511111	211	*	245	75	3
Potri.005Chr05	13510901	13511111	211	*	245	75	3
Potri.005Chr05	13510901	13511111	211	*	245	75	3
Potri.005Chr05	13510901	13511111	211	*	245	75	3
Potri.005Chr05	13510147	13510359	213	*	232	135	3
Potri.005Chr05	13510147	13510359	213	*	232	135	3
Potri.005Chr05	13510147	13510359	213	*	232	135	3
Potri.005Chr05	13510147	13510359	213	*	232	135	3
Potri.005Chr05	13547370	13547622	253	*	204	134	4
Potri.005Chr05	13547370	13547622	253	*	204	134	4

Potri.005	Chr05	14524539	14524743	205	*	306	112	3
Potri.005	Chr05	14814275	14814542	268	*	937	134	4
Potri.005	Chr05	14825925	14826134	210	*	408	68	3
Potri.005	Chr05	14825925	14826134	210	*	408	68	3
Potri.005	Chr05	16498370	16498667	298	*	431	146	2
Potri.005	Chr05	17718205	17718413	209	*	361	101	3
Potri.005	Chr05	17718205	17718413	209	*	361	101	3
Potri.005	Chr05	18577697	18578005	309	*	176	141	3
Potri.005	Chr05	19147111	19147415	305	*	404	147	5
Potri.005	Chr05	19331911	19332204	294	*	191	113	3
Potri.005	Chr05	19626375	19626622	248	*	327	81	2
Potri.005	Chr05	20544892	20545222	331	*	417	171	2
Potri.005	Chr05	21437309	21437533	225	*	171	99	3
Potri.005	Chr05	21883762	21883976	215	*	282	102	2
Potri.005	Chr05	21982630	21982932	303	*	244	136	3
Potri.005	Chr05	22394783	22395368	586	*	689	415	5
Potri.005	Chr05	22394783	22395368	586	*	689	415	5
Potri.005	Chr05	22985994	22986460	467	*	368	104	3
Potri.005	Chr05	23031405	23031721	317	*	198	158	3
Potri.005	Chr05	23576023	23576261	239	*	259	139	5
Potri.005	Chr05	23601924	23602134	211	*	175	116	4
Potri.005	Chr05	23601924	23602134	211	*	175	116	4
Potri.005	Chr05	23664862	23665310	449	*	224	155	3
Potri.005	Chr05	24083689	24083920	232	*	197	79	4
Potri.005	Chr05	24083689	24083920	232	*	197	79	4
Potri.005	Chr05	24205987	24206222	236	*	188	110	4
Potri.005	Chr05	24205987	24206222	236	*	188	110	4
Potri.005	Chr05	24285228	24285611	384	*	174	231	3
Potri.005	Chr05	25230823	25231369	547	*	224	378	3
Potri.005	Chr05	25230823	25231369	547	*	224	378	3
Potri.006	Chr06	584267	584492	226	*	209	98	3
Potri.006	Chr06	2704686	2704897	212	*	336	97	3
Potri.006	Chr06	3652600	3652801	202	*	178	111	3
Potri.006	Chr06	3652600	3652801	202	*	178	111	3
Potri.006	Chr06	3656156	3656406	251	*	171	118	2
Potri.006	Chr06	3674160	3674588	429	*	173	289	3
Potri.006	Chr06	4149558	4150011	454	*	176	135	3
Potri.006	Chr06	4149558	4150011	454	*	176	135	3
Potri.006	Chr06	4611631	4611904	274	*	181	95	2
Potri.006	Chr06	4611631	4611904	274	*	181	95	2
Potri.006	Chr06	4611631	4611904	274	*	181	95	2
Potri.006	Chr06	4993382	4993598	217	*	168	125	4
Potri.006	Chr06	5403030	5403262	233	*	196	139	4
Potri.006	Chr06	5456081	5456315	235	*	235	130	4
Potri.006	Chr06	6509763	6509977	215	*	175	112	5
Potri.006	Chr06	6522769	6523039	271	*	259	121	4
Potri.006	Chr06	6538315	6538566	252	*	232	88	4
Potri.006	Chr06	6538315	6538566	252	*	232	88	4
Potri.006	Chr06	7753987	7754199	213	*	169	129	5
Potri.006	Chr06	7753987	7754199	213	*	169	129	5
Potri.006	Chr06	8705806	8706086	281	*	241	129	4
Potri.006	Chr06	8705806	8706086	281	*	241	129	4
Potri.006	Chr06	8705806	8706086	281	*	241	129	4
Potri.006	Chr06	9339130	9339388	259	*	193	149	3

Potri.006	Chr06	9443032	9443257	226	*	169	120	5
Potri.006	Chr06	9457540	9457780	241	*	186	104	4
Potri.006	Chr06	10470671	10471079	409	*	203	195	3
Potri.006	Chr06	11142181	11142400	220	*	395	115	3
Potri.006	Chr06	12140513	12140752	240	*	288	153	6
Potri.006	Chr06	12897772	12898010	239	*	170	96	4
Potri.006	Chr06	12897772	12898010	239	*	170	96	4
Potri.006	Chr06	12897772	12898010	239	*	170	96	4
Potri.006	Chr06	12957848	12958058	211	*	309	116	3
Potri.006	Chr06	15214270	15214485	216	*	219	94	3
Potri.006	Chr06	15278442	15278658	217	*	513	138	5
Potri.006	Chr06	16338470	16338869	400	*	226	147	2
Potri.006	Chr06	16559802	16560015	214	*	175	117	4
Potri.006	Chr06	17649872	17650085	214	*	265	108	3
Potri.006	Chr06	18632759	18632964	206	*	402	103	3
Potri.006	Chr06	19020137	19020395	259	*	393	140	7
Potri.006	Chr06	19020137	19020395	259	*	393	140	7
Potri.006	Chr06	19020137	19020395	259	*	393	140	7
Potri.006	Chr06	19392821	19393162	342	*	237	153	5
Potri.006	Chr06	20042067	20042292	226	*	210	109	4
Potri.006	Chr06	20042067	20042292	226	*	210	109	4
Potri.006	Chr06	20917537	20917825	289	*	192	152	4
Potri.006	Chr06	20917537	20917825	289	*	192	152	4
Potri.006	Chr06	21209585	21210084	500	*	539	136	3
Potri.006	Chr06	21740868	21741446	579	*	354	171	2
Potri.006	Chr06	22052388	22052680	293	*	254	139	4
Potri.006	Chr06	22052388	22052680	293	*	254	139	4
Potri.006	Chr06	22052388	22052680	293	*	254	139	4
Potri.006	Chr06	22659554	22660043	490	*	305	336	3
Potri.006	Chr06	23738928	23739283	356	*	272	220	4
Potri.006	Chr06	24408878	24409099	222	*	188	115	5
Potri.006	Chr06	24678458	24678699	242	*	180	121	5
Potri.006	Chr06	24678458	24678699	242	*	180	121	5
Potri.006	Chr06	24678458	24678699	242	*	180	121	5
Potri.006	Chr06	26742203	26742449	247	*	197	112	4
Potri.007	Chr07	1007548	1007805	258	*	175	109	4
Potri.007	Chr07	1445906	1446148	243	*	210	135	5
Potri.007	Chr07	1445906	1446148	243	*	210	135	5
Potri.007	Chr07	3252335	3252665	331	*	545	163	3
Potri.007	Chr07	3578358	3578564	207	*	187	66	4
Potri.007	Chr07	3779481	3779747	267	*	168	103	3
Potri.007	Chr07	4771133	4771454	322	*	260	135	2
Potri.007	Chr07	4771133	4771454	322	*	260	135	2
Potri.007	Chr07	5102747	5102963	217	*	195	110	5
Potri.007	Chr07	5675940	5676165	226	*	183	119	4
Potri.007	Chr07	5974247	5974468	222	*	240	105	2
Potri.007	Chr07	7247424	7247633	210	*	295	140	3
Potri.007	Chr07	7247424	7247633	210	*	295	140	3
Potri.007	Chr07	7244866	7245083	218	*	266	116	4
Potri.007	Chr07	7244866	7245083	218	*	266	116	4
Potri.007	Chr07	7978966	7979178	213	*	267	97	3
Potri.007	Chr07	8147070	8147271	202	*	172	96	3
Potri.007	Chr07	8208911	8209304	394	*	349	285	4
Potri.007	Chr07	8773896	8774162	267	*	294	114	6

Potri.007	Chr07	8773896	8774162	267	*	294	114	6
Potri.007	Chr07	10547920	10548225	306	*	215	111	4
Potri.007	Chr07	11193598	11194041	444	*	168	157	3
Potri.007	Chr07	11690352	11691097	746	*	208	605	4
Potri.007	Chr07	12684523	12684810	288	*	174	142	3
Potri.007	Chr07	13002359	13002595	237	*	225	96	4
Potri.007	Chr07	13311166	13311725	560	*	254	144	3
Potri.007	Chr07	14210620	14210867	248	*	309	134	3
Potri.007	Chr07	14911290	14911517	228	*	175	115	4
Potri.007	Chr07	15318580	15318783	204	*	221	100	2
Potri.007	Chr07	15318082	15318344	263	*	274	149	3
Potri.007	Chr07	15358736	15359012	277	*	201	170	3
Potri.008	Chr08	380068	380344	277	*	383	148	2
Potri.008	Chr08	1517054	1517272	219	*	206	128	5
Potri.008	Chr08	1778725	1779103	379	*	207	211	3
Potri.008	Chr08	1778725	1779103	379	*	207	211	3
Potri.008	Chr08	1991147	1991369	223	*	174	126	3
Potri.008	Chr08	2815018	2815236	219	*	393	101	3
Potri.008	Chr08	3567336	3567589	254	*	169	115	4
Potri.008	Chr08	3616057	3616266	210	*	230	92	3
Potri.008	Chr08	3616057	3616266	210	*	230	92	3
Potri.008	Chr08	3695582	3695794	213	*	377	111	3
Potri.008	Chr08	3781586	3781857	272	*	284	108	4
Potri.008	Chr08	3781586	3781857	272	*	284	108	4
Potri.008	Chr08	4476890	4477540	651	*	196	377	3
Potri.008	Chr08	4516661	4516885	225	*	201	121	4
Potri.008	Chr08	5214475	5214697	223	*	195	131	5
Potri.008	Chr08	6070852	6071099	248	*	187	139	4
Potri.008	Chr08	6070852	6071099	248	*	187	139	4
Potri.008	Chr08	6163801	6164285	485	*	210	374	5
Potri.008	Chr08	6163801	6164285	485	*	210	374	5
Potri.008	Chr08	6625940	6626158	219	*	170	90	4
Potri.008	Chr08	6625940	6626158	219	*	170	90	4
Potri.008	Chr08	7843464	7843712	249	*	228	154	4
Potri.008	Chr08	8012196	8012437	242	*	175	133	4
Potri.008	Chr08	8012196	8012437	242	*	175	133	4
Potri.008	Chr08	8024917	8025172	256	*	229	140	4
Potri.008	Chr08	8631360	8631653	294	*	236	164	4
Potri.008	Chr08	8631360	8631653	294	*	236	164	4
Potri.008	Chr08	9867129	9867368	240	*	192	135	4
Potri.008	Chr08	9867129	9867368	240	*	192	135	4
Potri.008	Chr08	11092667	11092917	251	*	263	127	3
Potri.008	Chr08	11910275	11910530	256	*	203	121	4
Potri.008	Chr08	12093355	12093667	313	*	330	169	5
Potri.008	Chr08	12093355	12093667	313	*	330	169	5
Potri.008	Chr08	12401111	12401412	302	*	200	154	3
Potri.008	Chr08	13021089	13021310	222	*	190	107	4
Potri.008	Chr08	14569377	14569892	516	*	192	137	3
Potri.008	Chr08	15127483	15127814	332	*	294	169	4
Potri.008	Chr08	15732436	15732641	206	*	176	90	3
Potri.008	Chr08	15732436	15732641	206	*	176	90	3
Potri.008	Chr08	17238863	17239184	322	*	308	151	5
Potri.008	Chr08	17238863	17239184	322	*	308	151	5
Potri.008	Chr08	17235041	17235259	219	*	306	121	3

Potri.008	Chr08	17235041	17235259	219	*	306	121	3
Potri.008	Chr08	17483540	17484020	481	*	263	126	3
Potri.008	Chr08	18779671	18780309	639	*	469	452	2
Potri.008	Chr08	18779671	18780309	639	*	469	452	2
Potri.009	Chr09	188074	188275	202	*	288	138	3
Potri.009	Chr09	250842	251093	252	*	447	103	2
Potri.009	Chr09	415796	416018	223	*	169	121	5
Potri.009	Chr09	414081	414302	222	*	554	119	3
Potri.009	Chr09	987828	988123	296	*	616	172	3
Potri.009	Chr09	987828	988123	296	*	616	172	3
Potri.009	Chr09	987828	988123	296	*	616	172	3
Potri.009	Chr09	987828	988123	296	*	616	172	3
Potri.009	Chr09	989369	989621	253	*	397	152	3
Potri.009	Chr09	989369	989621	253	*	397	152	3
Potri.009	Chr09	989369	989621	253	*	397	152	3
Potri.009	Chr09	989369	989621	253	*	397	152	3
Potri.009	Chr09	991184	991712	529	*	393	351	2
Potri.009	Chr09	1385409	1385620	212	*	299	82	3
Potri.009	Chr09	1385409	1385620	212	*	299	82	3
Potri.009	Chr09	1385409	1385620	212	*	299	82	3
Potri.009	Chr09	2730027	2730244	218	*	523	129	3
Potri.009	Chr09	2730027	2730244	218	*	523	129	3
Potri.009	Chr09	2730027	2730244	218	*	523	129	3
Potri.009	Chr09	2730027	2730244	218	*	523	129	3
Potri.009	Chr09	2730720	2730929	210	*	330	140	3
Potri.009	Chr09	2730720	2730929	210	*	330	140	3
Potri.009	Chr09	2730720	2730929	210	*	330	140	3
Potri.009	Chr09	2730720	2730929	210	*	330	140	3
Potri.009	Chr09	4111819	4112061	243	*	254	130	4
Potri.009	Chr09	5384670	5384973	304	*	170	160	4
Potri.009	Chr09	5482353	5482584	232	*	170	139	4
Potri.009	Chr09	5494478	5494708	231	*	181	81	4
Potri.009	Chr09	5853196	5853452	257	*	180	137	3
Potri.009	Chr09	6591488	6591822	335	*	180	144	3
Potri.009	Chr09	6860119	6860380	262	*	247	118	4
Potri.009	Chr09	7143408	7143679	272	*	555	159	3
Potri.009	Chr09	7519930	7520201	272	*	177	167	4
Potri.009	Chr09	8620824	8621313	490	*	332	136	2
Potri.009	Chr09	10213863	10214125	263	*	192	94	4
Potri.009	Chr09	10239971	10240444	474	*	190	117	3
Potri.009	Chr09	10435910	10436209	300	*	230	153	5
Potri.009	Chr09	10435910	10436209	300	*	230	153	5
Potri.009	Chr09	11366307	11367012	706	*	262	578	4
Potri.009	Chr09	11403385	11403734	350	*	314	158	4
Potri.009	Chr09	11793301	11793562	262	*	187	135	4
Potri.009	Chr09	11793301	11793562	262	*	187	135	4
Potri.009	Chr09	11983524	11983734	211	*	212	76	3
Potri.009	Chr09	12026918	12027409	492	*	205	313	3
Potri.009	Chr09	12164492	12164899	408	*	170	197	3
Potri.009	Chr09	12948280	12948482	203	*	183	105	3
Potri.010	Chr10	678554	678782	229	*	175	126	3
Potri.010	Chr10	2849717	2849988	272	*	195	133	5
Potri.010	Chr10	4213200	4213410	211	*	720	85	5
Potri.010	Chr10	4424864	4425127	264	*	237	152	5

Potri.010	Chr10	4752260	4752479	220	*	372	100	3
Potri.010	Chr10	4752260	4752479	220	*	372	100	3
Potri.010	Chr10	4695536	4695839	304	*	237	155	4
Potri.010	Chr10	4695536	4695839	304	*	237	155	4
Potri.010	Chr10	4670264	4670561	298	*	341	127	4
Potri.010	Chr10	4670264	4670561	298	*	341	127	4
Potri.010	Chr10	5229777	5230032	256	*	1035	127	3
Potri.010	Chr10	5616028	5616276	249	*	379	75	5
Potri.010	Chr10	5747999	5748440	442	*	223	191	2
Potri.010	Chr10	7325234	7325860	627	*	182	332	3
Potri.010	Chr10	8333077	8333733	657	*	505	193	2
Potri.010	Chr10	8333077	8333733	657	*	505	193	2
Potri.010	Chr10	8334683	8334895	213	*	282	105	3
Potri.010	Chr10	8334683	8334895	213	*	282	105	3
Potri.010	Chr10	10002568	10002868	301	*	196	169	3
Potri.010	Chr10	10140443	10140767	325	*	221	182	3
Potri.010	Chr10	11301952	11302389	438	*	207	333	2
Potri.010	Chr10	11758523	11758757	235	*	170	133	4
Potri.010	Chr10	11849113	11849384	272	*	208	95	4
Potri.010	Chr10	12506727	12507011	285	*	299	132	4
Potri.010	Chr10	12506727	12507011	285	*	299	132	4
Potri.010	Chr10	13109552	13109776	225	*	178	117	4
Potri.010	Chr10	13134263	13134479	217	*	192	126	3
Potri.010	Chr10	13134263	13134479	217	*	192	126	3
Potri.010	Chr10	13192922	13193151	230	*	215	138	5
Potri.010	Chr10	13648842	13649046	205	*	171	99	4
Potri.010	Chr10	13736434	13736715	282	*	201	144	5
Potri.010	Chr10	14800777	14801185	409	*	181	196	4
Potri.010	Chr10	15939303	15939546	244	*	215	152	5
Potri.010	Chr10	16024808	16025131	324	*	225	167	4
Potri.010	Chr10	17008348	17008593	246	*	258	118	4
Potri.010	Chr10	17100301	17100667	367	*	172	177	3
Potri.010	Chr10	18069815	18070209	395	*	335	142	5
Potri.010	Chr10	18450658	18450875	218	*	169	97	3
Potri.010	Chr10	18934795	18935190	396	*	214	169	4
Potri.010	Chr10	19155725	19156085	361	*	312	207	3
Potri.010	Chr10	19411340	19411678	339	*	263	143	4
Potri.010	Chr10	19765326	19765557	232	*	410	121	3
Potri.010	Chr10	20360992	20361237	246	*	187	129	4
Potri.010	Chr10	20360992	20361237	246	*	187	129	4
Potri.010	Chr10	20360992	20361237	246	*	187	129	4
Potri.010	Chr10	20746208	20746443	236	*	206	91	4
Potri.010	Chr10	20746208	20746443	236	*	206	91	4
Potri.010	Chr10	20849709	20850172	464	*	171	135	3
Potri.010	Chr10	20955244	20955477	234	*	208	127	4
Potri.010	Chr10	20955244	20955477	234	*	208	127	4
Potri.011	Chr11	280384	280607	224	*	180	119	5
Potri.011	Chr11	1293925	1294164	240	*	207	131	4
Potri.011	Chr11	6675904	6676283	380	*	226	266	3
Potri.011	Chr11	7193887	7194291	405	*	729	279	3
Potri.011	Chr11	7204585	7204800	216	*	389	100	4
Potri.011	Chr11	7203916	7204136	221	*	293	117	3
Potri.011	Chr11	7206727	7206931	205	*	506	137	4
Potri.011	Chr11	7216295	7216796	502	*	486	166	2

Potri.011	Chr11	7216984	7217188	205	*	488	93	3
Potri.011	Chr11	7232108	7232312	205	*	215	125	3
Potri.011	Chr11	7232108	7232312	205	*	215	125	3
Potri.011	Chr11	8004416	8004657	242	*	223	86	5
Potri.011	Chr11	8281481	8281709	229	*	422	106	3
Potri.011	Chr11	8750210	8750433	224	*	206	98	5
Potri.011	Chr11	11651491	11651692	202	*	181	92	4
Potri.011	Chr11	11646597	11646832	236	*	208	118	3
Potri.011	Chr11	12064035	12064249	215	*	303	104	3
Potri.011	Chr11	12868168	12868389	222	*	177	151	4
Potri.011	Chr11	13861283	13861491	209	*	380	128	3
Potri.011	Chr11	14619743	14619978	236	*	188	113	3
Potri.011	Chr11	15719434	15720275	842	*	272	715	5
Potri.011	Chr11	16055882	16056093	212	*	411	107	3
Potri.011	Chr11	16376771	16376989	219	*	229	97	4
Potri.011	Chr11	16787551	16787780	230	*	197	100	5
Potri.011	Chr11	17248568	17248796	229	*	175	108	5
Potri.011	Chr11	18050016	18050691	676	*	277	498	3
Potri.012	Chr12	279345	279683	339	*	187	166	3
Potri.012	Chr12	878444	878674	231	*	232	121	3
Potri.012	Chr12	1700840	1701206	367	*	210	229	3
Potri.012	Chr12	1787499	1787713	215	*	168	124	3
Potri.012	Chr12	1875727	1875929	203	*	196	82	3
Potri.012	Chr12	1875727	1875929	203	*	196	82	3
Potri.012	Chr12	2051145	2051446	302	*	192	191	3
Potri.012	Chr12	3633292	3633510	219	*	175	91	5
Potri.012	Chr12	3767955	3768175	221	*	195	115	5
Potri.012	Chr12	3785404	3785650	247	*	285	105	4
Potri.012	Chr12	4989235	4989446	212	*	183	75	4
Potri.012	Chr12	5405245	5405472	228	*	177	126	4
Potri.012	Chr12	6891658	6891862	205	*	185	132	3
Potri.012	Chr12	6891658	6891862	205	*	185	132	3
Potri.012	Chr12	7487675	7487882	208	*	238	97	4
Potri.012	Chr12	8481537	8481822	286	*	258	160	4
Potri.012	Chr12	8481537	8481822	286	*	258	160	4
Potri.012	Chr12	8481537	8481822	286	*	258	160	4
Potri.012	Chr12	9825741	9826156	416	*	173	270	3
Potri.012	Chr12	9832325	9832546	222	*	191	132	4
Potri.012	Chr12	9850644	9850876	233	*	209	95	5
Potri.012	Chr12	10421124	10421343	220	*	264	92	5
Potri.012	Chr12	10421124	10421343	220	*	264	92	5
Potri.012	Chr12	10777583	10777798	216	*	331	124	3
Potri.012	Chr12	11144365	11144651	287	*	353	137	6
Potri.012	Chr12	11349177	11349416	240	*	238	159	4
Potri.012	Chr12	11684858	11685336	479	*	191	144	3
Potri.012	Chr12	12014961	12015164	204	*	183	91	3
Potri.012	Chr12	12312788	12313203	416	*	333	204	3
Potri.012	Chr12	12359956	12360182	227	*	187	70	4
Potri.012	Chr12	12359956	12360182	227	*	187	70	4
Potri.012	Chr12	12374717	12374919	203	*	200	105	3
Potri.012	Chr12	12374717	12374919	203	*	200	105	3
Potri.012	Chr12	12555547	12555862	316	*	209	172	3
Potri.012	Chr12	12872671	12872880	210	*	303	116	3
Potri.012	Chr12	12897468	12897762	295	*	209	112	3

Potri.012Chr12	14120146	14120661	516	*	226	200	3
Potri.012Chr12	14198834	14199046	213	*	169	99	3
Potri.012Chr12	14736852	14737208	357	*	175	185	3
Potri.012Chr12	14780382	14780601	220	*	193	95	4
Potri.012Chr12	14964775	14965018	244	*	169	126	3
Potri.012Chr12	14973069	14973402	334	*	195	154	3
Potri.012Chr12	15168580	15168920	341	*	347	180	3
Potri.012Chr12	15239700	15239936	237	*	192	128	4
Potri.012Chr12	15380375	15380653	279	*	230	143	5
Potri.012Chr12	15582553	15582973	421	*	172	170	3
Potri.012Chr12	15706156	15706422	267	*	186	77	3
Potri.013Chr13	635381	635924	544	*	210	163	3
Potri.013Chr13	792708	793014	307	*	182	180	4
Potri.013Chr13	1139200	1139429	230	*	270	102	5
Potri.013Chr13	1676299	1676684	386	*	360	148	2
Potri.013Chr13	1738462	1738744	283	*	208	112	3
Potri.013Chr13	3360468	3360706	239	*	219	111	3
Potri.013Chr13	3621400	3621688	289	*	277	144	4
Potri.013Chr13	3779731	3780132	402	*	187	296	3
Potri.013Chr13	3992374	3992719	346	*	281	193	4
Potri.013Chr13	5296954	5297207	254	*	642	123	3
Potri.013Chr13	5973873	5974118	246	*	212	129	3
Potri.013Chr13	6402281	6402538	258	*	402	130	2
Potri.013Chr13	6402281	6402538	258	*	402	130	2
Potri.013Chr13	6403078	6403523	446	*	250	89	2
Potri.013Chr13	6403078	6403523	446	*	250	89	2
Potri.013Chr13	6403918	6404394	477	*	678	148	3
Potri.013Chr13	6408437	6409840	1404	*	306	589	2
Potri.013Chr13	6410049	6410744	696	*	304	511	2
Potri.013Chr13	6414272	6414582	311	*	434	187	2
Potri.013Chr13	6539352	6539662	311	*	181	183	3
Potri.013Chr13	8398302	8398505	204	*	172	81	3
Potri.013Chr13	8397107	8397424	318	*	226	138	3
Potri.013Chr13	8676632	8676913	282	*	181	160	3
Potri.013Chr13	9592001	9592225	225	*	337	124	2
Potri.013Chr13	9592001	9592225	225	*	337	124	2
Potri.013Chr13	9857166	9857556	391	*	434	199	2
Potri.013Chr13	10736166	10736404	239	*	489	121	4
Potri.013Chr13	10736166	10736404	239	*	489	121	4
Potri.013Chr13	10738325	10738798	474	*	225	91	4
Potri.013Chr13	10738325	10738798	474	*	225	91	4
Potri.013Chr13	11150564	11150783	220	*	188	119	5
Potri.013Chr13	11190269	11190503	235	*	193	139	5
Potri.013Chr13	12676259	12676531	273	*	197	139	3
Potri.013Chr13	12942050	12942787	738	*	173	637	3
Potri.013Chr13	13253798	13254033	236	*	201	103	3
Potri.013Chr13	13244463	13244738	276	*	175	125	3
Potri.013Chr13	13614224	13614445	222	*	246	87	3
Potri.013Chr13	13967624	13967834	211	*	516	102	3
Potri.013Chr13	14522867	14523113	247	*	382	120	3
Potri.013Chr13	14666392	14667221	830	*	271	209	2
Potri.013Chr13	14666392	14667221	830	*	271	209	2
Potri.013Chr13	14684602	14685046	445	*	218	352	3
Potri.013Chr13	14754849	14755531	683	*	434	510	2

Potri.013Chr13	14757953	14758362	410	*	737	232	3
Potri.013Chr13	14757953	14758362	410	*	737	232	3
Potri.013Chr13	14765509	14765848	340	*	603	177	3
Potri.013Chr13	14765509	14765848	340	*	603	177	3
Potri.013Chr13	14765509	14765848	340	*	603	177	3
Potri.013Chr13	14768300	14768743	444	*	374	292	2
Potri.013Chr13	14768300	14768743	444	*	374	292	2
Potri.013Chr13	14768300	14768743	444	*	374	292	2
Potri.013Chr13	14767526	14767773	248	*	915	134	3
Potri.013Chr13	14767526	14767773	248	*	915	134	3
Potri.013Chr13	14767526	14767773	248	*	915	134	3
Potri.013Chr13	14770079	14770661	583	*	433	380	2
Potri.013Chr13	14770079	14770661	583	*	433	380	2
Potri.013Chr13	14773543	14773797	255	*	263	99	2
Potri.013Chr13	14773543	14773797	255	*	263	99	2
Potri.013Chr13	14772548	14772884	337	*	508	174	2
Potri.013Chr13	14772548	14772884	337	*	508	174	2
Potri.013Chr13	14775557	14775947	391	*	430	226	2
Potri.013Chr13	14779113	14779325	213	*	597	83	4
Potri.013Chr13	14785259	14785561	303	*	1228	140	4
Potri.013Chr13	14785259	14785561	303	*	1228	140	4
Potri.013Chr13	14788608	14789177	570	*	277	249	2
Potri.013Chr13	14790376	14790677	302	*	422	170	2
Potri.013Chr13	14790376	14790677	302	*	422	170	2
Potri.013Chr13	14789540	14790168	629	*	285	244	2
Potri.013Chr13	14789540	14790168	629	*	285	244	2
Potri.013Chr13	14790886	14791178	293	*	607	127	3
Potri.013Chr13	14790886	14791178	293	*	607	127	3
Potri.013Chr13	14792096	14792313	218	*	280	114	2
Potri.013Chr13	14793673	14795142	1470	*	318	598	2
Potri.013Chr13	14814110	14814340	231	*	459	125	3
Potri.013Chr13	14836649	14836865	217	*	624	131	3
Potri.013Chr13	14836649	14836865	217	*	624	131	3
Potri.013Chr13	14839740	14839970	231	*	337	103	3
Potri.013Chr13	14839740	14839970	231	*	337	103	3
Potri.013Chr13	14838921	14839387	467	*	615	243	3
Potri.013Chr13	14838921	14839387	467	*	615	243	3
Potri.013Chr13	14842253	14842764	512	*	441	316	2
Potri.013Chr13	14842253	14842764	512	*	441	316	2
Potri.013Chr13	14842253	14842764	512	*	441	316	2
Potri.013Chr13	14843072	14843558	487	*	388	139	2
Potri.013Chr13	14843072	14843558	487	*	388	139	2
Potri.013Chr13	14843072	14843558	487	*	388	139	2
Potri.013Chr13	14844892	14847116	2225	*	297	734	2
Potri.013Chr13	14844892	14847116	2225	*	297	734	2
Potri.013Chr13	14844892	14847116	2225	*	297	734	2
Potri.013Chr13	14849115	14850094	980	*	302	384	2
Potri.013Chr13	14849115	14850094	980	*	302	384	2
Potri.013Chr13	14851957	14852325	369	*	546	160	3
Potri.013Chr13	14853535	14853922	388	*	470	245	2
Potri.013Chr13	14853535	14853922	388	*	470	245	2
Potri.013Chr13	14867107	14869268	2162	*	284	237	2
Potri.013Chr13	14867107	14869268	2162	*	284	237	2
Potri.013Chr13	14866330	14866592	263	*	544	113	3

Potri.013	Chr13	14866330	14866592	263	*	544	113	3
Potri.013	Chr13	14879676	14880782	1107	*	451	180	2
Potri.013	Chr13	14879676	14880782	1107	*	451	180	2
Potri.013	Chr13	14883095	14883314	220	*	857	74	4
Potri.013	Chr13	14883095	14883314	220	*	857	74	4
Potri.013	Chr13	14896132	14896381	250	*	747	128	3
Potri.013	Chr13	14904725	14905092	368	*	370	131	3
Potri.013	Chr13	14904725	14905092	368	*	370	131	3
Potri.013	Chr13	14904725	14905092	368	*	370	131	3
Potri.013	Chr13	14907355	14907572	218	*	392	39	3
Potri.013	Chr13	14907355	14907572	218	*	392	39	3
Potri.013	Chr13	14907355	14907572	218	*	392	39	3
Potri.013	Chr13	14987548	14987800	253	*	203	135	4
Potri.013	Chr13	14987548	14987800	253	*	203	135	4
Potri.013	Chr13	15836524	15836740	217	*	374	100	3
Potri.013	Chr13	16069810	16070054	245	*	239	132	4
Potri.013	Chr13	16164331	16164605	275	*	281	148	6
Potri.014	Chr14	735918	736142	225	*	181	133	4
Potri.014	Chr14	1492151	1492456	306	*	357	141	6
Potri.014	Chr14	1492151	1492456	306	*	357	141	6
Potri.014	Chr14	1637553	1637791	239	*	191	123	3
Potri.014	Chr14	1637553	1637791	239	*	191	123	3
Potri.014	Chr14	2172135	2172369	235	*	186	124	4
Potri.014	Chr14	2172135	2172369	235	*	186	124	4
Potri.014	Chr14	3331410	3331703	294	*	193	146	4
Potri.014	Chr14	4240183	4240396	214	*	261	107	3
Potri.014	Chr14	4256216	4256475	260	*	286	100	3
Potri.014	Chr14	5356907	5357219	313	*	192	139	4
Potri.014	Chr14	6232305	6232575	271	*	177	117	3
Potri.014	Chr14	6386497	6386736	240	*	287	104	4
Potri.014	Chr14	6431184	6431637	454	*	357	121	3
Potri.014	Chr14	6975156	6975365	210	*	377	92	3
Potri.014	Chr14	6983024	6983371	348	*	194	154	4
Potri.014	Chr14	7291885	7292103	219	*	178	86	4
Potri.014	Chr14	8147135	8147397	263	*	183	128	3
Potri.014	Chr14	9201094	9201411	318	*	195	137	4
Potri.014	Chr14	9345135	9345353	219	*	308	123	3
Potri.014	Chr14	9345135	9345353	219	*	308	123	3
Potri.014	Chr14	10405941	10406300	360	*	428	201	5
Potri.014	Chr14	10405941	10406300	360	*	428	201	5
Potri.014	Chr14	11077199	11077427	229	*	407	130	3
Potri.014	Chr14	11077199	11077427	229	*	407	130	3
Potri.014	Chr14	12397126	12397396	271	*	269	130	5
Potri.014	Chr14	12397126	12397396	271	*	269	130	5
Potri.014	Chr14	12601509	12601777	269	*	227	149	4
Potri.014	Chr14	12946298	12946536	239	*	244	109	5
Potri.014	Chr14	12946298	12946536	239	*	244	109	5
Potri.014	Chr14	14775846	14776143	298	*	175	152	4
Potri.014	Chr14	15179903	15180132	230	*	192	128	5
Potri.014	Chr14	15933493	15933727	235	*	560	130	3
Potri.014	Chr14	16521024	16521270	247	*	383	102	2
Potri.014	Chr14	16978898	16980182	1285	*	179	976	2
Potri.014	Chr14	16978898	16980182	1285	*	179	976	2
Potri.014	Chr14	16978898	16980182	1285	*	179	976	2

Potri.014Chr14	16978898	16980182	1285	*	179	976	2
Potri.014Chr14	16978898	16980182	1285	*	179	976	2
Potri.014Chr14	17191025	17191266	242	*	204	116	4
Potri.014Chr14	17215397	17215628	232	*	405	113	3
Potri.014Chr14	18135696	18135898	203	*	250	106	3
Potri.014Chr14	18333017	18333238	222	*	357	119	4
Potri.014Chr14	18419621	18419980	360	*	175	226	3
Potri.015Chr15	461873	462108	236	*	181	95	4
Potri.015Chr15	1854564	1854801	238	*	272	88	4
Potri.015Chr15	1899690	1899953	264	*	179	150	3
Potri.015Chr15	2345890	2346330	441	*	186	155	3
Potri.015Chr15	3500748	3501045	298	*	192	163	4
Potri.015Chr15	4009026	4009277	252	*	233	123	4
Potri.015Chr15	4098981	4099336	356	*	229	190	3
Potri.015Chr15	4558037	4558245	209	*	311	90	2
Potri.015Chr15	4948623	4948906	284	*	206	158	5
Potri.015Chr15	4948623	4948906	284	*	206	158	5
Potri.015Chr15	4948623	4948906	284	*	206	158	5
Potri.015Chr15	4944476	4944707	232	*	191	128	5
Potri.015Chr15	4944476	4944707	232	*	191	128	5
Potri.015Chr15	4944476	4944707	232	*	191	128	5
Potri.015Chr15	4967890	4968263	374	*	652	171	6
Potri.015Chr15	4967890	4968263	374	*	652	171	6
Potri.015Chr15	4967890	4968263	374	*	652	171	6
Potri.015Chr15	6289629	6289839	211	*	267	104	3
Potri.015Chr15	6289629	6289839	211	*	267	104	3
Potri.015Chr15	6289629	6289839	211	*	267	104	3
Potri.015Chr15	6132555	6132816	262	*	223	126	5
Potri.015Chr15	6132555	6132816	262	*	223	126	5
Potri.015Chr15	6132555	6132816	262	*	223	126	5
Potri.015Chr15	6324550	6325657	1108	*	178	469	2
Potri.015Chr15	6324550	6325657	1108	*	178	469	2
Potri.015Chr15	6322481	6322828	348	*	509	161	3
Potri.015Chr15	6322481	6322828	348	*	509	161	3
Potri.015Chr15	6331008	6331542	535	*	199	364	2
Potri.015Chr15	6331008	6331542	535	*	199	364	2
Potri.015Chr15	6331008	6331542	535	*	199	364	2
Potri.015Chr15	6331008	6331542	535	*	199	364	2
Potri.015Chr15	6331008	6331542	535	*	199	364	2
Potri.015Chr15	6328535	6328930	396	*	209	173	2
Potri.015Chr15	6328535	6328930	396	*	209	173	2
Potri.015Chr15	6328535	6328930	396	*	209	173	2
Potri.015Chr15	6328535	6328930	396	*	209	173	2
Potri.015Chr15	6328535	6328930	396	*	209	173	2
Potri.015Chr15	6327259	6327672	414	*	218	270	2
Potri.015Chr15	6327259	6327672	414	*	218	270	2
Potri.015Chr15	6327259	6327672	414	*	218	270	2
Potri.015Chr15	6327259	6327672	414	*	218	270	2
Potri.015Chr15	6327259	6327672	414	*	218	270	2
Potri.015Chr15	6329820	6330191	372	*	227	171	2
Potri.015Chr15	6329820	6330191	372	*	227	171	2
Potri.015Chr15	6329820	6330191	372	*	227	171	2
Potri.015Chr15	6329820	6330191	372	*	227	171	2
Potri.015Chr15	6329820	6330191	372	*	227	171	2

Potri.015	Chr15	6325864	6326114	251	*	418	116	2
Potri.015	Chr15	6325864	6326114	251	*	418	116	2
Potri.015	Chr15	6325864	6326114	251	*	418	116	2
Potri.015	Chr15	6325864	6326114	251	*	418	116	2
Potri.015	Chr15	6325864	6326114	251	*	418	116	2
Potri.015	Chr15	6326514	6326745	232	*	628	123	3
Potri.015	Chr15	6326514	6326745	232	*	628	123	3
Potri.015	Chr15	6326514	6326745	232	*	628	123	3
Potri.015	Chr15	6326514	6326745	232	*	628	123	3
Potri.015	Chr15	6326514	6326745	232	*	628	123	3
Potri.015	Chr15	6409201	6409415	215	*	288	93	3
Potri.015	Chr15	6409201	6409415	215	*	288	93	3
Potri.015	Chr15	6588669	6589028	360	*	185	190	3
Potri.015	Chr15	6682178	6682397	220	*	172	123	3
Potri.015	Chr15	6969549	6969757	209	*	226	82	4
Potri.015	Chr15	7444315	7444539	225	*	168	113	5
Potri.015	Chr15	8388319	8388556	238	*	201	128	4
Potri.015	Chr15	8388319	8388556	238	*	201	128	4
Potri.015	Chr15	8887117	8887348	232	*	310	135	4
Potri.015	Chr15	10235085	10235307	223	*	503	123	3
Potri.015	Chr15	11321131	11321356	226	*	173	146	4
Potri.015	Chr15	12313711	12313968	258	*	210	141	5
Potri.015	Chr15	13653651	13653894	244	*	215	116	3
Potri.015	Chr15	13712456	13712705	250	*	399	129	5
Potri.015	Chr15	14300750	14301043	294	*	258	148	4
Potri.015	Chr15	14918229	14918486	258	*	192	139	3
Potri.015	Chr15	15241519	15241885	367	*	179	192	3
Potri.016	Chr16	625084	625538	455	*	254	335	5
Potri.016	Chr16	627588	627815	228	*	233	127	3
Potri.016	Chr16	905345	905600	256	*	237	96	3
Potri.016	Chr16	1867470	1867723	254	*	214	155	4
Potri.016	Chr16	3542430	3542665	236	*	196	93	4
Potri.016	Chr16	3796009	3796626	618	*	176	310	3
Potri.016	Chr16	4119864	4120176	313	*	183	170	3
Potri.016	Chr16	4267058	4267301	244	*	241	117	3
Potri.016	Chr16	4948225	4948497	273	*	269	124	5
Potri.016	Chr16	6098891	6099288	398	*	392	172	4
Potri.016	Chr16	6579784	6580058	275	*	210	145	3
Potri.016	Chr16	6678744	6679083	340	*	179	200	3
Potri.016	Chr16	7275824	7276179	356	*	320	165	3
Potri.016	Chr16	7689821	7690032	212	*	327	88	3
Potri.016	Chr16	7689821	7690032	212	*	327	88	3
Potri.016	Chr16	7689821	7690032	212	*	327	88	3
Potri.016	Chr16	8130685	8130911	227	*	214	114	3
Potri.016	Chr16	8218262	8218496	235	*	458	132	3
Potri.016	Chr16	11095519	11095738	220	*	246	109	3
Potri.016	Chr16	12349383	12350004	622	*	483	175	4
Potri.016	Chr16	12541018	12541219	202	*	215	85	3
Potri.016	Chr16	13771339	13771632	294	*	192	148	4
Potri.016	Chr16	13992626	13992871	246	*	170	104	4
Potri.016	Chr16	13992626	13992871	246	*	170	104	4
Potri.017	Chr17	804357	804607	251	*	203	114	4
Potri.017	Chr17	2039379	2039849	471	*	198	156	3
Potri.017	Chr17	2261594	2261979	386	*	188	242	3

Potri.017	Chr17	2355644	2355852	209	*	204	84	3
Potri.017	Chr17	2665049	2665258	210	*	183	78	3
Potri.017	Chr17	3582131	3582363	233	*	244	130	5
Potri.017	Chr17	3582131	3582363	233	*	244	130	5
Potri.017	Chr17	5654502	5654924	423	*	175	255	4
Potri.017	Chr17	5868625	5868846	222	*	460	112	3
Potri.017	Chr17	5940914	5941147	234	*	582	118	3
Potri.017	Chr17	5925983	5926211	229	*	181	114	4
Potri.017	Chr17	6939831	6940100	270	*	209	99	3
Potri.017	Chr17	7300015	7300245	231	*	874	128	5
Potri.017	Chr17	7358823	7359070	248	*	541	121	3
Potri.017	Chr17	8293044	8293296	253	*	237	138	4
Potri.017	Chr17	8769072	8769288	217	*	343	123	3
Potri.017	Chr17	8769072	8769288	217	*	343	123	3
Potri.017	Chr17	8769072	8769288	217	*	343	123	3
Potri.017	Chr17	9597791	9598003	213	*	171	105	3
Potri.017	Chr17	9597791	9598003	213	*	171	105	3
Potri.017	Chr17	9597791	9598003	213	*	171	105	3
Potri.017	Chr17	9634484	9634763	280	*	179	201	4
Potri.017	Chr17	10055068	10055295	228	*	550	94	3
Potri.017	Chr17	10938472	10938690	219	*	185	90	3
Potri.017	Chr17	10937777	10937996	220	*	216	129	4
Potri.017	Chr17	11508006	11508328	323	*	198	156	3
Potri.017	Chr17	13004792	13005061	270	*	208	157	3
Potri.017	Chr17	13146131	13146415	285	*	248	134	4
Potri.017	Chr17	13533660	13533872	213	*	303	92	3
Potri.017	Chr17	14201299	14201507	209	*	185	121	4
Potri.017	Chr17	14836792	14837023	232	*	491	95	3
Potri.017	Chr17	14836792	14837023	232	*	491	95	3
Potri.017	Chr17	14837449	14837653	205	*	314	92	3
Potri.017	Chr17	15101487	15102270	784	*	212	225	2
Potri.017	Chr17	15518775	15519123	349	*	173	154	3
Potri.018	Chr18	1278807	1279016	210	*	179	107	3
Potri.018	Chr18	2322662	2322924	263	*	175	90	3
Potri.018	Chr18	2343894	2344167	274	*	205	156	4
Potri.018	Chr18	2437335	2437686	352	*	317	176	2
Potri.018	Chr18	2684266	2684518	253	*	181	158	4
Potri.018	Chr18	3014239	3014597	359	*	175	220	4
Potri.018	Chr18	3014239	3014597	359	*	175	220	4
Potri.018	Chr18	3107667	3107892	226	*	530	127	3
Potri.018	Chr18	3229837	3230082	246	*	352	121	6
Potri.018	Chr18	4061342	4061794	453	*	221	183	2
Potri.018	Chr18	4112354	4112573	220	*	225	116	4
Potri.018	Chr18	4112354	4112573	220	*	225	116	4
Potri.018	Chr18	4112354	4112573	220	*	225	116	4
Potri.018	Chr18	4112354	4112573	220	*	225	116	4
Potri.018	Chr18	4690898	4691105	208	*	239	139	3
Potri.018	Chr18	5031688	5031913	226	*	349	95	3
Potri.018	Chr18	5242377	5242612	236	*	898	120	4
Potri.018	Chr18	6400348	6400583	236	*	177	146	4
Potri.018	Chr18	6400348	6400583	236	*	177	146	4
Potri.018	Chr18	6400348	6400583	236	*	177	146	4
Potri.018	Chr18	6400348	6400583	236	*	177	146	4
Potri.018	Chr18	6617839	6618084	246	*	704	135	6

Potri.018	Chr18	6617839	6618084	246	*	704	135	6
Potri.018	Chr18	6617839	6618084	246	*	704	135	6
Potri.018	Chr18	6608429	6608789	361	*	435	137	3
Potri.018	Chr18	6608429	6608789	361	*	435	137	3
Potri.018	Chr18	6608429	6608789	361	*	435	137	3
Potri.018	Chr18	7356032	7356235	204	*	184	97	2
Potri.018	Chr18	7356032	7356235	204	*	184	97	2
Potri.018	Chr18	7768175	7768466	292	*	189	120	2
Potri.018	Chr18	7768175	7768466	292	*	189	120	2
Potri.018	Chr18	8130695	8130952	258	*	181	137	4
Potri.018	Chr18	11640501	11640729	229	*	269	130	4
Potri.018	Chr18	11706478	11706743	266	*	573	138	6
Potri.018	Chr18	12559350	12559659	310	*	220	158	4
Potri.018	Chr18	12559350	12559659	310	*	220	158	4
Potri.018	Chr18	12559350	12559659	310	*	220	158	4
Potri.018	Chr18	12559350	12559659	310	*	220	158	4
Potri.018	Chr18	12640604	12640883	280	*	180	121	5
Potri.018	Chr18	12642901	12643260	360	*	180	170	3
Potri.018	Chr18	12797411	12797679	269	*	223	124	4
Potri.018	Chr18	13033689	13033899	211	*	188	114	5
Potri.018	Chr18	15517355	15517558	204	*	188	122	5
Potri.018	Chr18	15591553	15591759	207	*	216	140	3
Potri.019	Chr19	286217	286607	391	*	214	269	3
Potri.019	Chr19	457307	457518	212	*	214	92	4
Potri.019	Chr19	1431883	1432455	573	*	194	430	4
Potri.019	Chr19	1431883	1432455	573	*	194	430	4
Potri.019	Chr19	1998582	1998824	243	*	266	133	5
Potri.019	Chr19	2143248	2143463	216	*	253	65	3
Potri.019	Chr19	2175572	2175811	240	*	179	95	4
Potri.019	Chr19	2310559	2310857	299	*	168	113	3
Potri.019	Chr19	2751653	2751916	264	*	320	120	3
Potri.019	Chr19	3188226	3188439	214	*	228	59	3
Potri.019	Chr19	3188226	3188439	214	*	228	59	3
Potri.019	Chr19	3193969	3194199	231	*	399	165	3
Potri.019	Chr19	3193969	3194199	231	*	399	165	3
Potri.019	Chr19	3192907	3193128	222	*	642	109	4
Potri.019	Chr19	3192907	3193128	222	*	642	109	4
Potri.019	Chr19	3194890	3195647	758	*	292	389	2
Potri.019	Chr19	3202901	3203470	570	*	923	129	3
Potri.019	Chr19	3202901	3203470	570	*	923	129	3
Potri.019	Chr19	3831881	3832177	297	*	219	136	3
Potri.019	Chr19	4765413	4765637	225	*	197	151	4
Potri.019	Chr19	4765413	4765637	225	*	197	151	4
Potri.019	Chr19	4765413	4765637	225	*	197	151	4
Potri.019	Chr19	4775656	4775884	229	*	406	101	3
Potri.019	Chr19	5320875	5321121	247	*	808	139	3
Potri.019	Chr19	5545993	5546563	571	*	291	434	2
Potri.019	Chr19	6691063	6691619	557	*	404	92	3
Potri.019	Chr19	7535840	7536112	273	*	346	149	6
Potri.019	Chr19	7834493	7834749	257	*	509	120	3
Potri.019	Chr19	7834493	7834749	257	*	509	120	3
Potri.019	Chr19	7834038	7834258	221	*	211	106	2
Potri.019	Chr19	7834038	7834258	221	*	211	106	2
Potri.019	Chr19	9986010	9986234	225	*	272	65	5

Potri.019	Chr19	9986010	9986234	225	*	272	65	5
Potri.019	Chr19	9986010	9986234	225	*	272	65	5
Potri.019	Chr19	9986010	9986234	225	*	272	65	5
Potri.019	Chr19	9986720	9987027	308	*	309	151	2
Potri.019	Chr19	9986720	9987027	308	*	309	151	2
Potri.019	Chr19	9986720	9987027	308	*	309	151	2
Potri.019	Chr19	9986720	9987027	308	*	309	151	2
Potri.019	Chr19	10011824	10012061	238	*	183	162	4
Potri.019	Chr19	11359705	11359914	210	*	173	107	4
Potri.019	Chr19	11359705	11359914	210	*	173	107	4
Potri.019	Chr19	11801277	11801588	312	*	281	145	5
Potri.019	Chr19	11801277	11801588	312	*	281	145	5
Potri.019	Chr19	11801277	11801588	312	*	281	145	5
Potri.019	Chr19	12331612	12331842	231	*	271	124	4
Potri.019	Chr19	12958554	12958796	243	*	210	127	5
Potri.019	Chr19	13062550	13062795	246	*	304	108	5
Potri.019	Chr19	13062550	13062795	246	*	304	108	5
Potri.019	Chr19	13316679	13316924	246	*	203	87	4
Potri.019	Chr19	15371466	15371738	273	*	195	142	3
Potri.T00	scaffold_	235955	236178	224	*	580	121	3
Potri.T00	scaffold_	235955	236178	224	*	580	121	3
Potri.T00	scaffold_	437154	437696	543	*	446	410	3
Potri.T00	scaffold_	437154	437696	543	*	446	410	3
Potri.T00	scaffold_	437154	437696	543	*	446	410	3
Potri.T00	scaffold_	437154	437696	543	*	446	410	3
Potri.T00	scaffold_	437154	437696	543	*	446	410	3
Potri.T00	scaffold_	435713	435925	213	*	431	74	3
Potri.T00	scaffold_	435713	435925	213	*	431	74	3
Potri.T00	scaffold_	435713	435925	213	*	431	74	3
Potri.T00	scaffold_	435713	435925	213	*	431	74	3
Potri.T00	scaffold_	435713	435925	213	*	431	74	3
Potri.T00	scaffold_	438879	439424	546	*	313	141	2
Potri.T00	scaffold_	746250	746665	416	*	271	218	2
Potri.T01	scaffold_	101172	101701	530	*	272	430	3
Potri.T01	scaffold_	101172	101701	530	*	272	430	3
Potri.T01	scaffold_	396446	396714	269	*	224	149	3
Potri.T01	scaffold_	466595	466834	240	*	374	141	3
Potri.T01	scaffold_	192813	193031	219	*	398	96	4
Potri.T02	scaffold_	188221	188729	509	*	306	154	4
Potri.T02	scaffold_	188221	188729	509	*	306	154	4
Potri.T02	scaffold_	226718	227001	284	*	575	149	3
Potri.T02	scaffold_	226718	227001	284	*	575	149	3
Potri.T02	scaffold_	127749	127967	219	*	198	85	4
Potri.T03	scaffold_	229679	229892	214	*	317	154	4
Potri.T03	scaffold_	265464	265680	217	*	171	71	3
Potri.T03	scaffold_	246208	246636	429	*	189	337	2
Potri.T03	scaffold_	246208	246636	429	*	189	337	2
Potri.T03	scaffold_	246208	246636	429	*	189	337	2
Potri.T03	scaffold_	247062	247562	501	*	383	116	3
Potri.T03	scaffold_	247062	247562	501	*	383	116	3
Potri.T03	scaffold_	247062	247562	501	*	383	116	3
Potri.T03	scaffold_	247891	248093	203	*	241	88	3
Potri.T03	scaffold_	247891	248093	203	*	241	88	3
Potri.T03	scaffold_	247891	248093	203	*	241	88	3

Potri.T03scaffold_	263597	263824	228	*	360	108	4
Potri.T03scaffold_	272244	272483	240	*	473	112	3
Potri.T04scaffold_	109281	109492	212	*	313	120	3
Potri.T04scaffold_	109281	109492	212	*	313	120	3
Potri.T04scaffold_	109281	109492	212	*	313	120	3
Potri.T04scaffold_	109281	109492	212	*	313	120	3
Potri.T04scaffold_	78841	79062	222	*	280	96	4
Potri.T04scaffold_	78841	79062	222	*	280	96	4
Potri.T04scaffold_	78841	79062	222	*	280	96	4
Potri.T04scaffold_	78841	79062	222	*	280	96	4
Potri.T04scaffold_	153530	153744	215	*	247	121	3
Potri.T04scaffold_	51381	51608	228	*	401	106	3
Potri.T04scaffold_	196942	197161	220	*	175	136	5
Potri.T05scaffold_	187580	187803	224	*	312	103	4
Potri.T05scaffold_	183397	183607	211	*	293	113	3
Potri.T06scaffold_	112672	112987	316	*	176	168	3
Potri.T06scaffold_	112672	112987	316	*	176	168	3
Potri.T06scaffold_	112672	112987	316	*	176	168	3
Potri.T07scaffold_	11224	11477	254	*	215	137	3
Potri.T07scaffold_	10005	10212	208	*	203	118	3
Potri.T07scaffold_	71376	71636	261	*	467	143	2
Potri.T07scaffold_	124652	124857	206	*	186	131	3
Potri.T08scaffold_	16883	17093	211	*	185	97	3
Potri.T09scaffold_	2089	2308	220	*	176	134	4
Potri.T09scaffold_	2089	2308	220	*	176	134	4
Potri.T09scaffold_	16648	16862	215	*	172	127	4
Potri.T09scaffold_	58552	58835	284	*	378	165	3
Potri.T10scaffold_	15041	15346	306	*	187	173	2
Potri.T10scaffold_	15041	15346	306	*	187	173	2
Potri.T10scaffold_	15545	17317	1773	*	198	311	2
Potri.T10scaffold_	15545	17317	1773	*	198	311	2
Potri.T10scaffold_	23429	23762	334	*	338	177	2
Potri.T10scaffold_	61178	61730	553	*	756	130	3
Potri.T10scaffold_	54053	54260	208	*	281	102	3
Potri.T10scaffold_	8098	8322	225	*	427	122	3
Potri.T11scaffold_	37809	38018	210	*	221	101	3
Potri.T11scaffold_	70116	70351	236	*	410	100	5
Potri.T11scaffold_	21341	21569	229	*	341	109	4
Potri.T11scaffold_	21341	21569	229	*	341	109	4
Potri.T11scaffold_	48324	48548	225	*	193	126	4
Potri.T15scaffold_	3858	4117	260	*	252	143	3
Potri.T16scaffold_	12154	12726	573	*	221	267	2
Potri.T17scaffold_	1678	1928	251	*	208	104	4
Potri.T17scaffold_	1678	1928	251	*	208	104	4
Potri.T17scaffold_	1992	2197	206	*	250	66	3

)AP-seq comparison.

thick.end	thick.wid	itemRgb	peak.x	start_pos	end_posit	feature_s	insideFea	distancet
21	17	NA	LBD21.31R	931891	937283	+	upstream	-2746
26	23	NA	LBD21.31R	3608830	3616405	+	downstrea	11185
26	23	NA	LBD21.31R	3608830	3616405	+	downstrea	11185
26	23	NA	LBD21.31R	3608830	3616405	+	downstrea	11185
17	14	NA	LBD21.31R	3655019	3662744	+	inside	2877
44	42	NA	LBD21.31R	4424461	4425107	-	inside	540
17	15	NA	LBD21.31R	4817225	4820458	+	inside	214
57	54	NA	LBD21.31R	5081114	5083400	-	upstream	-234
17	14	NA	LBD21.31R	5163758	5164215	+	downstrea	487
21	17	NA	LBD21.31R	5525312	5526193	+	upstream	-6925
19	17	NA	LBD21.31R	5594633	5596573	+	upstream	-289
17	14	NA	LBD21.31R	5712602	5713239	-	overlapSt	183
17	14	NA	LBD21.31R	5712602	5713239	-	overlapSt	183
24	22	NA	LBD21.31R	6537780	6548390	+	inside	650
24	22	NA	LBD21.31R	6537780	6548390	+	inside	650
24	20	NA	LBD21.31R	7116809	7118715	+	inside	1357
24	20	NA	LBD21.31R	7116809	7118715	+	inside	1357
19	16	NA	LBD21.31R	7201940	7204932	+	inside	993
19	16	NA	LBD21.31R	7201940	7204932	+	inside	993
19	16	NA	LBD21.31R	7201940	7204932	+	inside	993
19	16	NA	LBD21.31R	9208104	9209012	-	upstream	-2680
31	27	NA	LBD21.31R	9497158	9500671	+	inside	1850
17	15	NA	LBD21.31R	9856787	9866426	+	upstream	-607
22	20	NA	LBD21.31R	9962114	9963181	+	downstrea	3357
36	35	NA	LBD21.31R	11492791	11497064	+	upstream	-1174
20	19	NA	LBD21.31R	11542659	11544254	-	upstream	-19426
18	15	NA	LBD21.31R	12721833	12723526	+	upstream	-1537
20	17	NA	LBD21.31R	13556117	13556365	+	downstrea	902
34	30	NA	LBD21.31R	15234652	15237125	-	inside	2391
34	30	NA	LBD21.31R	15234652	15237125	-	inside	2391
17	14	NA	LBD21.31R	15639966	15640319	-	downstrea	2587
19	17	NA	LBD21.31R	16178623	16183438	-	inside	1638
16	14	NA	LBD21.31R	17034511	17041769	+	inside	2781
41	40	NA	LBD21.31R	17650350	17656084	-	downstrea	6780
27	25	NA	LBD21.31R	18767933	18772523	+	downstrea	19821
21	17	NA	LBD21.31R	18808260	18809389	-	upstream	-29226
16	12	NA	LBD21.31R	19160680	19162246	-	upstream	-4316
29	27	NA	LBD21.31R	19852151	19852980	+	overlapSt	-35
24	22	NA	LBD21.31R	20658471	20661501	+	upstream	-770
23	19	NA	LBD21.31R	20856453	20862070	-	upstream	-1541
34	31	NA	LBD21.31R	20943752	20958263	-	inside	8570
22	20	NA	LBD21.31R	21096101	21098276	-	downstrea	3354
26	22	NA	LBD21.31R	21731709	21738117	+	inside	3919
26	22	NA	LBD21.31R	21731709	21738117	+	inside	3919
23	20	NA	LBD21.31R	22363394	22367792	-	inside	681
23	20	NA	LBD21.31R	22842447	22843911	-	overlapEn	1465
16	14	NA	LBD21.31R	23047696	23054875	-	inside	1409
24	20	NA	LBD21.31R	24556970	24562120	-	downstrea	7432
35	32	NA	LBD21.31R	25022582	25027843	+	inside	151
53	51	NA	LBD21.31R	26610644	26613598	-	inside	669
34	30	NA	LBD21.31R	27813645	27816590	-	upstream	-5579

17	14	NA	LBD21.31R	27813645	27816590	-	upstream	-4632
44	42	NA	LBD21.31R	28195252	28200532	-	upstream	-2392
17	13	NA	LBD21.31R	28195252	28200532	-	upstream	-3913
19	16	NA	LBD21.31R	28656046	28658388	-	upstream	-601
17	13	NA	LBD21.31R	28983611	28985938	-	inside	1156
29	28	NA	LBD21.31R	29390781	29391222	-	inside	405
22	18	NA	LBD21.31R	29748443	29750426	-	downstrea	7544
20	18	NA	LBD21.31R	31452614	31455086	+	upstream	-3823
34	32	NA	LBD21.31R	33457530	33457793	-	overlapSt	201
18	16	NA	LBD21.31R	33568408	33569446	-	upstream	-1135
17	15	NA	LBD21.31R	33974233	33976599	-	inside	494
20	17	NA	LBD21.31R	34316929	34319074	+	upstream	-392
20	17	NA	LBD21.31R	34316929	34319074	+	upstream	-392
18	16	NA	LBD21.31R	36660993	36687274	+	inside	7059
18	16	NA	LBD21.31R	36703035	36719635	-	inside	370
17	15	NA	LBD21.31R	36703035	36719635	-	inside	2948
17	15	NA	LBD21.31R	36770585	36775252	-	inside	1264
30	25	NA	LBD21.31R	38900791	38901054	-	downstrea	783
32	30	NA	LBD21.31R	40126837	40127766	-	downstrea	4693
17	13	NA	LBD21.31R	40937912	40940508	-	upstream	-3171
52	50	NA	LBD21.31R	41014240	41017948	-	inside	1999
17	14	NA	LBD21.31R	41340927	41349252	-	upstream	-3256
41	39	NA	LBD21.31R	41624735	41626633	+	downstrea	8901
23	19	NA	LBD21.31R	41808763	41810069	-	downstrea	4576
23	19	NA	LBD21.31R	41808763	41810069	-	downstrea	4576
23	19	NA	LBD21.31R	41808763	41810069	-	downstrea	4576
21	18	NA	LBD21.31R	42015598	42016722	-	upstream	-4637
27	24	NA	LBD21.31R	43033549	43041023	+	inside	3284
18	17	NA	LBD21.31R	43726632	43728235	-	inside	770
18	17	NA	LBD21.31R	43726632	43728235	-	inside	770
18	17	NA	LBD21.31R	43726632	43728235	-	inside	770
36	35	NA	LBD21.31R	43726632	43728235	-	inside	1555
36	35	NA	LBD21.31R	43726632	43728235	-	inside	1555
36	35	NA	LBD21.31R	43726632	43728235	-	inside	1555
46	44	NA	LBD21.31R	45750195	45758236	-	downstrea	39277
46	44	NA	LBD21.31R	45750195	45758236	-	downstrea	39277
21	19	NA	LBD21.31R	45800670	45801056	-	upstream	-10941
24	22	NA	LBD21.31R	45800670	45801056	-	upstream	-3720
29	26	NA	LBD21.31R	47420222	47423184	-	upstream	-4110
29	26	NA	LBD21.31R	47420222	47423184	-	upstream	-4110
29	26	NA	LBD21.31R	47420222	47423184	-	upstream	-4110
20	17	NA	LBD21.31R	48294396	48295061	-	downstrea	3697
30	26	NA	LBD21.31R	48744474	48747110	+	downstrea	4386
23	21	NA	LBD21.31R	48820335	48827240	+	inside	1361
17	14	NA	LBD21.31R	49433260	49437196	+	downstrea	7399
24	22	NA	LBD21.31R	49658499	49662020	+	upstream	-1248
19	17	NA	LBD21.31R	240097	242866	-	inside	469
25	23	NA	LBD21.31R	1269779	1270145	-	downstrea	6128
20	18	NA	LBD21.31R	1269779	1270145	-	upstream	-234
21	19	NA	LBD21.31R	1762114	1764183	+	inside	118
18	15	NA	LBD21.31R	1813265	1814888	-	upstream	-6948
19	16	NA	LBD21.31R	1813265	1814888	-	upstream	-9787
34	32	NA	LBD21.31R	1824453	1838295	-	inside	10252
17	15	NA	LBD21.31R	2557989	2565187	-	inside	820

33	31	NA	LBD21.31R	2560092	2560412	+	inside	86
26	23	NA	LBD21.31R	2804643	2807203	+	upstream	-2193
67	63	NA	LBD21.31R	2804643	2807203	+	inside	233
26	23	NA	LBD21.31R	3253001	3261425	-	inside	1555
41	38	NA	LBD21.31R	3483467	3485264	+	inside	746
41	38	NA	LBD21.31R	3483467	3485264	+	inside	746
18	16	NA	LBD21.31R	3665608	3675093	+	inside	3124
18	16	NA	LBD21.31R	3665608	3675093	+	inside	3124
38	37	NA	LBD21.31R	3748149	3751459	-	overlapSt	265
20	17	NA	LBD21.31R	3782590	3787280	+	overlapSt	-45
28	26	NA	LBD21.31R	3848631	3851551	+	inside	271
21	18	NA	LBD21.31R	4988728	4995830	-	upstream	-2007
46	44	NA	LBD21.31R	5521471	5523733	-	upstream	-671
25	22	NA	LBD21.31R	5971484	5974511	-	inside	857
35	33	NA	LBD21.31R	6134192	6137820	-	upstream	-2246
17	15	NA	LBD21.31R	6758985	6761062	+	inside	1376
17	15	NA	LBD21.31R	6758985	6761062	+	inside	1376
17	15	NA	LBD21.31R	6758985	6761062	+	inside	1376
17	14	NA	LBD21.31R	6809734	6813364	+	upstream	-4631
24	21	NA	LBD21.31R	6817567	6820006	+	upstream	-3314
24	21	NA	LBD21.31R	6817567	6820006	+	upstream	-3314
26	23	NA	LBD21.31R	6997442	6999188	-	upstream	-3418
19	16	NA	LBD21.31R	7140938	7145141	+	downstrea	4378
19	16	NA	LBD21.31R	7829783	7831956	+	downstrea	9181
19	16	NA	LBD21.31R	7829783	7831956	+	downstrea	9181
26	23	NA	LBD21.31R	8024624	8029932	+	inside	246
20	18	NA	LBD21.31R	8322992	8326606	+	upstream	-733
20	18	NA	LBD21.31R	8322992	8326606	+	upstream	-733
16	14	NA	LBD21.31R	9176812	9180006	+	inside	181
16	14	NA	LBD21.31R	9176812	9180006	+	inside	181
19	18	NA	LBD21.31R	10233594	10242380	+	inside	4327
16	14	NA	LBD21.31R	10423223	10424588	-	upstream	-1019
19	16	NA	LBD21.31R	10483728	10484993	+	downstrea	1321
17	14	NA	LBD21.31R	11792508	11794803	+	upstream	-3116
22	20	NA	LBD21.31R	12456399	12457606	+	downstrea	1909
18	15	NA	LBD21.31R	13296225	13298788	+	downstrea	6469
21	18	NA	LBD21.31R	13618757	13622764	+	inside	3594
26	22	NA	LBD21.31R	14004276	14007348	+	upstream	-1025
46	44	NA	LBD21.31R	14263537	14263850	+	inside	31
72	70	NA	LBD21.31R	15622905	15627562	+	upstream	-4371
31	27	NA	LBD21.31R	15787688	15793426	+	upstream	-1389
19	17	NA	LBD21.31R	16766346	16767636	-	overlapEn	1395
22	19	NA	LBD21.31R	16834480	16838766	+	inside	2357
27	25	NA	LBD21.31R	17124896	17127337	+	inside	651
39	37	NA	LBD21.31R	17656193	17656629	+	upstream	-4989
39	37	NA	LBD21.31R	17656193	17656629	+	upstream	-4989
39	37	NA	LBD21.31R	17656193	17656629	+	upstream	-4989
39	37	NA	LBD21.31R	17656193	17656629	+	upstream	-4989
63	61	NA	LBD21.31R	17656193	17656629	+	downstrea	1261
63	61	NA	LBD21.31R	17656193	17656629	+	downstrea	1261
63	61	NA	LBD21.31R	17656193	17656629	+	downstrea	1261
63	61	NA	LBD21.31R	17656193	17656629	+	downstrea	1261
73	71	NA	LBD21.31R	17656193	17656629	+	upstream	-3287
73	71	NA	LBD21.31R	17656193	17656629	+	upstream	-3287

73	71	NA	LBD21.31R	17656193	17656629	+	upstream	-3287
73	71	NA	LBD21.31R	17656193	17656629	+	upstream	-3287
19	17	NA	LBD21.31R	17656193	17656629	+	upstream	-4185
19	17	NA	LBD21.31R	17656193	17656629	+	upstream	-4185
19	17	NA	LBD21.31R	17656193	17656629	+	upstream	-4185
19	17	NA	LBD21.31R	17656193	17656629	+	upstream	-4185
32	30	NA	LBD21.31R	17802211	17802676	+	downstrea	11558
30	28	NA	LBD21.31R	18302824	18303082	+	inside	11
25	24	NA	LBD21.31R	18358100	18358803	-	downstrea	2637
25	24	NA	LBD21.31R	18358100	18358803	-	downstrea	2637
25	24	NA	LBD21.31R	18358100	18358803	-	downstrea	2637
25	24	NA	LBD21.31R	18358100	18358803	-	downstrea	2637
25	24	NA	LBD21.31R	18358100	18358803	-	downstrea	2637
25	24	NA	LBD21.31R	18358100	18358803	-	downstrea	2637
25	24	NA	LBD21.31R	18358100	18358803	-	downstrea	2637
25	24	NA	LBD21.31R	18358100	18358803	-	downstrea	2637
25	24	NA	LBD21.31R	18358100	18358803	-	downstrea	2637
25	24	NA	LBD21.31R	18358100	18358803	-	downstrea	2637
18	17	NA	LBD21.31R	18358100	18358803	-	downstrea	5762
18	17	NA	LBD21.31R	18358100	18358803	-	downstrea	5762
18	17	NA	LBD21.31R	18358100	18358803	-	downstrea	5762
18	17	NA	LBD21.31R	18358100	18358803	-	downstrea	5762
18	17	NA	LBD21.31R	18358100	18358803	-	downstrea	5762
18	17	NA	LBD21.31R	18358100	18358803	-	downstrea	5762
18	17	NA	LBD21.31R	18358100	18358803	-	downstrea	5762
18	17	NA	LBD21.31R	18358100	18358803	-	downstrea	5762
18	17	NA	LBD21.31R	18358100	18358803	-	downstrea	5762
18	17	NA	LBD21.31R	18358100	18358803	-	downstrea	5762
20	19	NA	LBD21.31R	18358100	18358803	-	overlapEn	835
20	19	NA	LBD21.31R	18358100	18358803	-	overlapEn	835
20	19	NA	LBD21.31R	18358100	18358803	-	overlapEn	835
20	19	NA	LBD21.31R	18358100	18358803	-	overlapEn	835
20	19	NA	LBD21.31R	18358100	18358803	-	overlapEn	835
20	19	NA	LBD21.31R	18358100	18358803	-	overlapEn	835
20	19	NA	LBD21.31R	18358100	18358803	-	overlapEn	835
20	19	NA	LBD21.31R	18358100	18358803	-	overlapEn	835
20	19	NA	LBD21.31R	18358100	18358803	-	overlapEn	835
20	19	NA	LBD21.31R	18358100	18358803	-	overlapEn	835
20	19	NA	LBD21.31R	18358100	18358803	-	downstrea	7692
20	19	NA	LBD21.31R	18358100	18358803	-	downstrea	7692
20	19	NA	LBD21.31R	18358100	18358803	-	downstrea	7692
20	19	NA	LBD21.31R	18358100	18358803	-	downstrea	7692
20	19	NA	LBD21.31R	18358100	18358803	-	downstrea	7692
20	19	NA	LBD21.31R	18358100	18358803	-	downstrea	7692
20	19	NA	LBD21.31R	18358100	18358803	-	downstrea	7692
20	19	NA	LBD21.31R	18358100	18358803	-	downstrea	7692
20	19	NA	LBD21.31R	18358100	18358803	-	downstrea	7692
20	19	NA	LBD21.31R	18358100	18358803	-	downstrea	7692
26	25	NA	LBD21.31R	18358100	18358803	-	downstrea	1998
26	25	NA	LBD21.31R	18358100	18358803	-	downstrea	1998
26	25	NA	LBD21.31R	18358100	18358803	-	downstrea	1998
26	25	NA	LBD21.31R	18358100	18358803	-	downstrea	1998
26	25	NA	LBD21.31R	18358100	18358803	-	downstrea	1998
26	25	NA	LBD21.31R	18358100	18358803	-	downstrea	1998

26	25	NA	LBD21.31R	18358100	18358803	-	downstrea	1998
26	25	NA	LBD21.31R	18358100	18358803	-	downstrea	1998
26	25	NA	LBD21.31R	18358100	18358803	-	downstrea	1998
26	25	NA	LBD21.31R	18358100	18358803	-	downstrea	1998
20	19	NA	LBD21.31R	18358100	18358803	-	downstrea	9846
20	19	NA	LBD21.31R	18358100	18358803	-	downstrea	9846
20	19	NA	LBD21.31R	18358100	18358803	-	downstrea	9846
20	19	NA	LBD21.31R	18358100	18358803	-	downstrea	9846
20	19	NA	LBD21.31R	18358100	18358803	-	downstrea	9846
20	19	NA	LBD21.31R	18358100	18358803	-	downstrea	9846
20	19	NA	LBD21.31R	18358100	18358803	-	downstrea	9846
20	19	NA	LBD21.31R	18358100	18358803	-	downstrea	9846
20	19	NA	LBD21.31R	18358100	18358803	-	downstrea	9846
20	19	NA	LBD21.31R	18358100	18358803	-	downstrea	9846
21	20	NA	LBD21.31R	18358100	18358803	-	downstrea	4638
21	20	NA	LBD21.31R	18358100	18358803	-	downstrea	4638
21	20	NA	LBD21.31R	18358100	18358803	-	downstrea	4638
21	20	NA	LBD21.31R	18358100	18358803	-	downstrea	4638
21	20	NA	LBD21.31R	18358100	18358803	-	downstrea	4638
21	20	NA	LBD21.31R	18358100	18358803	-	downstrea	4638
21	20	NA	LBD21.31R	18358100	18358803	-	downstrea	4638
21	20	NA	LBD21.31R	18358100	18358803	-	downstrea	4638
21	20	NA	LBD21.31R	18358100	18358803	-	downstrea	4638
21	20	NA	LBD21.31R	18358100	18358803	-	downstrea	4638
22	21	NA	LBD21.31R	18361982	18362639	+	downstrea	1372
17	16	NA	LBD21.31R	18367804	18368815	+	overlapSt	-2573
17	16	NA	LBD21.31R	18367804	18368815	+	overlapSt	-2573
26	25	NA	LBD21.31R	18367804	18368815	+	inside	641
26	25	NA	LBD21.31R	18367804	18368815	+	inside	641
25	24	NA	LBD21.31R	18377658	18379352	-	overlapSt	1146
25	24	NA	LBD21.31R	18377658	18379352	-	overlapSt	1146
21	20	NA	LBD21.31R	18377658	18379352	-	inside	1691
21	20	NA	LBD21.31R	18377658	18379352	-	inside	1691
27	26	NA	LBD21.31R	18387444	18388134	+	includeFe	-788
27	26	NA	LBD21.31R	18387444	18388134	+	includeFe	-788
27	26	NA	LBD21.31R	18387444	18388134	+	includeFe	-788
18	17	NA	LBD21.31R	18388647	18390665	+	inside	66
18	17	NA	LBD21.31R	18388647	18390665	+	inside	66
17	14	NA	LBD21.31R	19561742	19564303	+	upstream	-11201
18	16	NA	LBD21.31R	20471383	20474810	+	upstream	-505
32	29	NA	LBD21.31R	20561725	20563528	+	downstrea	7530
32	29	NA	LBD21.31R	20561725	20563528	+	downstrea	7530
30	26	NA	LBD21.31R	21167013	21172583	-	inside	567
18	14	NA	LBD21.31R	21548048	21548636	-	downstrea	2280
17	14	NA	LBD21.31R	21819526	21822784	-	overlapEn	3259
27	23	NA	LBD21.31R	21932768	21941584	+	upstream	-7070
25	23	NA	LBD21.31R	22760610	22768273	-	overlapSt	207
16	12	NA	LBD21.31R	23765393	23767140	+	upstream	-858
16	12	NA	LBD21.31R	23765393	23767140	+	upstream	-858
27	24	NA	LBD21.31R	23903060	23906186	-	overlapSt	59
27	24	NA	LBD21.31R	23903060	23906186	-	overlapSt	59
27	24	NA	LBD21.31R	23903060	23906186	-	overlapSt	59
27	24	NA	LBD21.31R	23903060	23906186	-	overlapSt	59
19	17	NA	LBD21.31R	24750711	24753375	+	inside	1085

18	15	NA	LBD21.31R	25138768	25142143	+	upstream	-300
18	15	NA	LBD21.31R	25138768	25142143	+	upstream	-300
22	19	NA	LBD21.31R	69145	71538	+	upstream	-5880
22	19	NA	LBD21.31R	69145	71538	+	upstream	-5880
26	21	NA	LBD21.31R	69145	71538	+	upstream	-7174
26	21	NA	LBD21.31R	69145	71538	+	upstream	-7174
23	21	NA	LBD21.31R	655424	657531	+	inside	690
21	19	NA	LBD21.31R	2550013	2551810	-	upstream	-42
21	19	NA	LBD21.31R	2550013	2551810	-	upstream	-42
20	18	NA	LBD21.31R	3022134	3022642	-	inside	396
17	14	NA	LBD21.31R	3374216	3375340	+	overlapEn	895
26	23	NA	LBD21.31R	3374216	3375340	+	downstrea	2483
24	21	NA	LBD21.31R	4749885	4750656	-	upstream	-5016
24	21	NA	LBD21.31R	4749885	4750656	-	upstream	-5016
19	16	NA	LBD21.31R	6415789	6419435	+	downstrea	7249
54	52	NA	LBD21.31R	9386830	9388800	+	inside	52
54	52	NA	LBD21.31R	9386830	9388800	+	inside	52
54	52	NA	LBD21.31R	9386830	9388800	+	inside	52
84	82	NA	LBD21.31R	9386830	9388800	+	upstream	-788
84	82	NA	LBD21.31R	9386830	9388800	+	upstream	-788
84	82	NA	LBD21.31R	9386830	9388800	+	upstream	-788
64	62	NA	LBD21.31R	9386830	9388800	+	inside	593
64	62	NA	LBD21.31R	9386830	9388800	+	inside	593
64	62	NA	LBD21.31R	9386830	9388800	+	inside	593
29	27	NA	LBD21.31R	9389396	9390123	+	upstream	-881
29	27	NA	LBD21.31R	9389396	9390123	+	upstream	-881
41	40	NA	LBD21.31R	9389396	9390123	+	overlapSt	-227
41	40	NA	LBD21.31R	9389396	9390123	+	overlapSt	-227
42	41	NA	LBD21.31R	9567815	9569026	-	inside	1064
21	20	NA	LBD21.31R	9567815	9569026	-	inside	535
55	53	NA	LBD21.31R	9567815	9569026	-	upstream	-188
51	49	NA	LBD21.31R	10183128	10183823	-	downstrea	1744
16	14	NA	LBD21.31R	10534366	10538515	+	inside	723
21	19	NA	LBD21.31R	12729229	12730819	+	downstrea	6437
21	19	NA	LBD21.31R	12729229	12730819	+	downstrea	6437
17	15	NA	LBD21.31R	13040521	13046522	-	inside	2590
17	15	NA	LBD21.31R	13040521	13046522	-	inside	2590
23	20	NA	LBD21.31R	13049039	13055620	-	inside	504
18	16	NA	LBD21.31R	13105319	13107221	+	upstream	-1197
17	15	NA	LBD21.31R	13156727	13175786	+	inside	1597
18	16	NA	LBD21.31R	13183164	13184572	+	inside	367
23	21	NA	LBD21.31R	13699658	13700594	+	upstream	-2174
21	17	NA	LBD21.31R	14047746	14056301	-	upstream	-4134
17	15	NA	LBD21.31R	15324744	15330797	-	upstream	-3067
22	20	NA	LBD21.31R	15379900	15381837	-	inside	1340
20	16	NA	LBD21.31R	15551696	15552231	+	downstrea	2095
22	18	NA	LBD21.31R	15709563	15713084	+	downstrea	5827
31	30	NA	LBD21.31R	15835933	15838778	+	inside	1790
17	14	NA	LBD21.31R	16372582	16375217	-	upstream	-7653
17	14	NA	LBD21.31R	16372582	16375217	-	upstream	-7653
16	14	NA	LBD21.31R	16625709	16626938	-	inside	1223
38	36	NA	LBD21.31R	16701064	16705723	+	upstream	-1292
19	16	NA	LBD21.31R	17002532	17008723	+	downstrea	12664
19	15	NA	LBD21.31R	17596241	17597637	+	upstream	-1237

19	15	NA	LBD21.31R	17596241	17597637	+	upstream	-1237
24	21	NA	LBD21.31R	17950578	17950986	+	downstrea	6130
24	21	NA	LBD21.31R	17950578	17950986	+	downstrea	6130
19	15	NA	LBD21.31R	18368222	18372666	+	upstream	-5098
29	25	NA	LBD21.31R	18770564	18771906	-	upstream	-550
21	20	NA	LBD21.31R	19531740	19535025	-	inside	1054
22	20	NA	LBD21.31R	20186834	20195032	+	inside	7583
22	20	NA	LBD21.31R	20186834	20195032	+	inside	7583
53	50	NA	LBD21.31R	20294523	20302361	-	inside	4825
53	50	NA	LBD21.31R	20294523	20302361	-	inside	4825
21	18	NA	LBD21.31R	20353339	20357188	+	upstream	-3136
21	18	NA	LBD21.31R	20353339	20357188	+	upstream	-3136
28	26	NA	LBD21.31R	20447558	20452100	+	inside	138
16	13	NA	LBD21.31R	20665302	20666936	+	upstream	-7935
16	13	NA	LBD21.31R	20665302	20666936	+	upstream	-7935
16	13	NA	LBD21.31R	20665302	20666936	+	upstream	-7935
21	18	NA	LBD21.31R	21514119	21516242	-	downstrea	3910
17	15	NA	LBD21.31R	21526754	21527456	+	inside	352
22	19	NA	LBD21.31R	808810	809677	+	downstrea	2421
21	20	NA	LBD21.31R	1296320	1297652	+	inside	870
17	14	NA	LBD21.31R	2795406	2799775	-	upstream	-3301
17	14	NA	LBD21.31R	2795406	2799775	-	upstream	-3301
22	18	NA	LBD21.31R	3417387	3417957	-	upstream	-2830
18	15	NA	LBD21.31R	3793576	3794363	-	upstream	-7592
27	25	NA	LBD21.31R	3868748	3870670	-	inside	1254
24	21	NA	LBD21.31R	4187175	4187502	+	downstrea	2836
18	14	NA	LBD21.31R	5238500	5250295	+	upstream	-4403
28	24	NA	LBD21.31R	6269473	6273663	+	inside	277
18	16	NA	LBD21.31R	6778244	6781216	+	inside	147
18	16	NA	LBD21.31R	8206433	8208989	+	overlapEn	2328
18	15	NA	LBD21.31R	8367867	8368944	+	downstrea	1276
18	15	NA	LBD21.31R	8367867	8368944	+	downstrea	1276
16	13	NA	LBD21.31R	8367867	8368944	+	inside	132
16	13	NA	LBD21.31R	8367867	8368944	+	inside	132
29	26	NA	LBD21.31R	9511345	9512253	+	upstream	-15596
33	32	NA	LBD21.31R	9511345	9512253	+	upstream	-7587
37	35	NA	LBD21.31R	10314112	10321954	+	inside	1140
17	14	NA	LBD21.31R	11929544	11932387	-	upstream	-2539
36	35	NA	LBD21.31R	12466740	12468284	-	upstream	-4135
36	35	NA	LBD21.31R	12466740	12468284	-	upstream	-4135
20	17	NA	LBD21.31R	12590162	12594879	+	downstrea	25790
65	63	NA	LBD21.31R	13124991	13125335	-	upstream	-75367
37	36	NA	LBD21.31R	14349106	14352191	+	downstrea	9917
37	36	NA	LBD21.31R	14349106	14352191	+	downstrea	9917
37	36	NA	LBD21.31R	14349106	14352191	+	downstrea	9917
37	36	NA	LBD21.31R	14349106	14352191	+	downstrea	9917
37	36	NA	LBD21.31R	14349106	14352191	+	downstrea	9917
37	36	NA	LBD21.31R	14349106	14352191	+	downstrea	9917
40	38	NA	LBD21.31R	14414010	14416706	+	downstrea	44084
30	26	NA	LBD21.31R	14903140	14906264	+	upstream	-3608
34	32	NA	LBD21.31R	16134853	16136863	+	downstrea	3906
34	32	NA	LBD21.31R	16134853	16136863	+	downstrea	3906
24	21	NA	LBD21.31R	16762048	16763104	-	overlapEn	1222
24	21	NA	LBD21.31R	16762048	16763104	-	overlapEn	1222

22	18	NA	LBD21.31R	17748145	17751189	+	inside	2644
18	15	NA	LBD21.31R	18167700	18173549	+	upstream	-8785
18	16	NA	LBD21.31R	18519395	18520295	+	downstrea	7526
19	16	NA	LBD21.31R	18519395	18520295	+	upstream	-3096
28	26	NA	LBD21.31R	18672434	18673602	-	upstream	-4152
28	26	NA	LBD21.31R	18672434	18673602	-	upstream	-3090
26	24	NA	LBD21.31R	19044352	19047726	+	inside	2308
26	24	NA	LBD21.31R	19645993	19651241	-	inside	818
26	24	NA	LBD21.31R	19645993	19651241	-	inside	818
26	24	NA	LBD21.31R	19645993	19651241	-	inside	818
30	28	NA	LBD21.31R	20203128	20207004	-	inside	489
28	24	NA	LBD21.31R	20353847	20355967	+	downstrea	2643
19	17	NA	LBD21.31R	20855645	20857045	+	downstrea	1916
57	55	NA	LBD21.31R	20885873	20888083	+	inside	1855
20	18	NA	LBD21.31R	21396958	21401302	+	inside	2362
35	32	NA	LBD21.31R	22881087	22884413	-	inside	840
44	42	NA	LBD21.31R	24164253	24172484	-	inside	1764
17	14	NA	LBD21.31R	347790	350058	+	downstrea	2572
31	29	NA	LBD21.31R	1854298	1856108	-	overlapSt	322
31	29	NA	LBD21.31R	1854298	1856108	-	overlapSt	322
31	29	NA	LBD21.31R	1854298	1856108	-	overlapSt	322
36	34	NA	LBD21.31R	1876628	1880313	-	inside	911
21	20	NA	LBD21.31R	4181343	4182149	+	downstrea	2640
17	16	NA	LBD21.31R	4181343	4182149	+	downstrea	1717
17	14	NA	LBD21.31R	4534405	4536336	+	inside	977
26	23	NA	LBD21.31R	5591213	5595844	-	upstream	-2841
18	15	NA	LBD21.31R	5704544	5706720	-	downstrea	18001
22	19	NA	LBD21.31R	6349752	6352599	+	inside	2340
25	22	NA	LBD21.31R	6474126	6478918	-	inside	2028
64	62	NA	LBD21.31R	6898522	6901736	+	inside	1629
18	16	NA	LBD21.31R	7170333	7173494	+	upstream	-4730
26	22	NA	LBD21.31R	7383725	7385275	+	upstream	-5212
21	19	NA	LBD21.31R	8401879	8410117	+	inside	3019
22	19	NA	LBD21.31R	11613891	11617243	+	upstream	-6636
42	39	NA	LBD21.31R	11705721	11709426	-	inside	353
20	16	NA	LBD21.31R	12269399	12271223	+	downstrea	10202
39	37	NA	LBD21.31R	12725031	12730565	+	downstrea	18901
39	37	NA	LBD21.31R	12725031	12730565	+	downstrea	18901
16	13	NA	LBD21.31R	12770351	12773239	+	upstream	-18306
58	56	NA	LBD21.31R	13493293	13493694	+	inside	118
30	28	NA	LBD21.31R	13495859	13496047	-	upstream	-8663
30	28	NA	LBD21.31R	13495859	13496047	-	upstream	-8663
30	28	NA	LBD21.31R	13495859	13496047	-	upstream	-8663
30	28	NA	LBD21.31R	13495859	13496047	-	upstream	-8663
24	22	NA	LBD21.31R	13495859	13496047	-	upstream	-14854
24	22	NA	LBD21.31R	13495859	13496047	-	upstream	-14854
24	22	NA	LBD21.31R	13495859	13496047	-	upstream	-14854
24	22	NA	LBD21.31R	13495859	13496047	-	upstream	-14854
23	21	NA	LBD21.31R	13495859	13496047	-	upstream	-14100
23	21	NA	LBD21.31R	13495859	13496047	-	upstream	-14100
23	21	NA	LBD21.31R	13495859	13496047	-	upstream	-14100
23	21	NA	LBD21.31R	13495859	13496047	-	upstream	-14100
20	17	NA	LBD21.31R	13548653	13549048	+	upstream	-1283
20	17	NA	LBD21.31R	13548653	13549048	+	upstream	-1283

30	28	NA	LBD21.31R	14532529	14533505	+	upstream	-7990
93	90	NA	LBD21.31R	14813787	14814410	-	overlapSt	135
40	38	NA	LBD21.31R	14824857	14826149	-	inside	224
40	38	NA	LBD21.31R	14824857	14826149	-	inside	224
43	42	NA	LBD21.31R	16507797	16510707	+	upstream	-9427
36	34	NA	LBD21.31R	17714154	17715725	-	upstream	-2480
36	34	NA	LBD21.31R	17714154	17715725	-	upstream	-2480
17	15	NA	LBD21.31R	18573573	18578651	-	inside	954
40	36	NA	LBD21.31R	19147416	19150201	+	upstream	-305
19	17	NA	LBD21.31R	19329500	19334579	+	inside	2411
32	31	NA	LBD21.31R	19611383	19622976	-	upstream	-3399
41	40	NA	LBD21.31R	20549534	20550472	-	downstrea	5580
17	15	NA	LBD21.31R	21433863	21438717	-	inside	1408
28	27	NA	LBD21.31R	21881226	21882935	+	downstrea	2536
24	22	NA	LBD21.31R	21981629	21983177	-	inside	547
68	64	NA	LBD21.31R	22394468	22396557	-	inside	1774
68	64	NA	LBD21.31R	22394468	22396557	-	inside	1774
36	34	NA	LBD21.31R	22985956	22986419	-	overlapSt	425
19	17	NA	LBD21.31R	23030899	23036682	+	inside	506
25	21	NA	LBD21.31R	23570628	23572383	+	downstrea	5395
17	14	NA	LBD21.31R	23600307	23604582	-	inside	2658
17	14	NA	LBD21.31R	23600307	23604582	-	inside	2658
22	20	NA	LBD21.31R	23664913	23671039	+	overlapSt	-51
19	16	NA	LBD21.31R	24079656	24082417	-	upstream	-1272
19	16	NA	LBD21.31R	24079656	24082417	-	upstream	-1272
18	15	NA	LBD21.31R	24202969	24205332	-	upstream	-655
18	15	NA	LBD21.31R	24202969	24205332	-	upstream	-655
17	15	NA	LBD21.31R	24283167	24285817	-	inside	589
22	20	NA	LBD21.31R	25225915	25231843	-	inside	1020
22	20	NA	LBD21.31R	25225915	25231843	-	inside	1020
20	18	NA	LBD21.31R	587389	588339	+	upstream	-3122
33	31	NA	LBD21.31R	2689811	2705764	-	inside	1078
17	15	NA	LBD21.31R	3652919	3653326	-	downstrea	726
17	15	NA	LBD21.31R	3652919	3653326	-	downstrea	726
17	16	NA	LBD21.31R	3655493	3666168	+	inside	663
17	15	NA	LBD21.31R	3671522	3674425	-	overlapSt	265
17	15	NA	LBD21.31R	4148448	4152549	-	inside	2991
17	15	NA	LBD21.31R	4148448	4152549	-	inside	2991
18	17	NA	LBD21.31R	4611204	4615026	-	inside	3395
18	17	NA	LBD21.31R	4611204	4615026	-	inside	3395
18	17	NA	LBD21.31R	4611204	4615026	-	inside	3395
16	13	NA	LBD21.31R	4996504	5006232	+	upstream	-3122
19	16	NA	LBD21.31R	5396646	5401181	-	upstream	-1849
23	20	NA	LBD21.31R	5451266	5458349	+	inside	4815
17	13	NA	LBD21.31R	6510362	6512593	+	upstream	-599
25	22	NA	LBD21.31R	6517063	6518158	-	upstream	-4611
23	20	NA	LBD21.31R	6538210	6541462	-	inside	3147
23	20	NA	LBD21.31R	6538210	6541462	-	inside	3147
16	12	NA	LBD21.31R	7746324	7748908	-	upstream	-5079
16	12	NA	LBD21.31R	7746324	7748908	-	upstream	-5079
24	21	NA	LBD21.31R	8701663	8703532	-	upstream	-2274
24	21	NA	LBD21.31R	8701663	8703532	-	upstream	-2274
24	21	NA	LBD21.31R	8701663	8703532	-	upstream	-2274
19	17	NA	LBD21.31R	9336398	9340989	-	inside	1859

16	12	NA	LBD21.31R	9445700	9447868	+	upstream	-2668
18	15	NA	LBD21.31R	9461415	9496093	+	upstream	-3875
20	18	NA	LBD21.31R	10470188	10470993	+	overlapEn	483
39	37	NA	LBD21.31R	11151518	11151907	+	upstream	-9337
28	23	NA	LBD21.31R	12135840	12136248	+	downstrea	4673
17	14	NA	LBD21.31R	12897437	12901197	-	inside	3425
17	14	NA	LBD21.31R	12897437	12901197	-	inside	3425
17	14	NA	LBD21.31R	12897437	12901197	-	inside	3425
30	28	NA	LBD21.31R	12953016	12957023	-	upstream	-825
21	19	NA	LBD21.31R	15215286	15217572	-	downstrea	3302
51	47	NA	LBD21.31R	15248067	15250270	-	upstream	-28172
22	21	NA	LBD21.31R	16330173	16330536	+	downstrea	8297
17	14	NA	LBD21.31R	16554131	16565217	+	inside	5671
26	24	NA	LBD21.31R	17626093	17627487	+	downstrea	23779
40	38	NA	LBD21.31R	18633690	18636357	-	downstrea	3598
39	33	NA	LBD21.31R	19005679	19006954	-	upstream	-13183
39	33	NA	LBD21.31R	19005679	19006954	-	upstream	-13183
39	33	NA	LBD21.31R	19005679	19006954	-	upstream	-13183
23	19	NA	LBD21.31R	19392135	19393336	-	inside	515
21	18	NA	LBD21.31R	20063876	20065794	+	upstream	-21809
21	18	NA	LBD21.31R	20063876	20065794	+	upstream	-21809
19	16	NA	LBD21.31R	20907598	20912145	-	upstream	-5392
19	16	NA	LBD21.31R	20907598	20912145	-	upstream	-5392
53	51	NA	LBD21.31R	21209507	21209889	+	overlapEn	78
35	34	NA	LBD21.31R	21744980	21746664	-	downstrea	5796
25	22	NA	LBD21.31R	22064648	22069887	+	upstream	-12260
25	22	NA	LBD21.31R	22064648	22069887	+	upstream	-12260
25	22	NA	LBD21.31R	22064648	22069887	+	upstream	-12260
30	28	NA	LBD21.31R	22659114	22660766	+	inside	440
27	24	NA	LBD21.31R	23751412	23754666	+	upstream	-12484
18	14	NA	LBD21.31R	24412663	24417659	+	upstream	-3785
18	14	NA	LBD21.31R	24667224	24674234	-	upstream	-4224
18	14	NA	LBD21.31R	24667224	24674234	-	upstream	-4224
18	14	NA	LBD21.31R	24667224	24674234	-	upstream	-4224
19	16	NA	LBD21.31R	26745119	26745769	-	downstrea	3566
17	14	NA	LBD21.31R	1002705	1005406	-	upstream	-2142
21	17	NA	LBD21.31R	1442351	1442648	-	upstream	-3258
21	17	NA	LBD21.31R	1442351	1442648	-	upstream	-3258
54	52	NA	LBD21.31R	3252284	3254824	-	inside	2489
18	15	NA	LBD21.31R	3566099	3570373	-	upstream	-7985
16	14	NA	LBD21.31R	3779080	3781182	-	inside	1701
26	25	NA	LBD21.31R	4759259	4760930	-	upstream	-10203
26	25	NA	LBD21.31R	4759259	4760930	-	upstream	-10203
19	15	NA	LBD21.31R	5114576	5118175	+	upstream	-11829
18	15	NA	LBD21.31R	5661027	5662744	+	downstrea	14913
24	23	NA	LBD21.31R	5978584	5981095	+	upstream	-4337
29	27	NA	LBD21.31R	7250907	7251317	+	upstream	-3483
29	27	NA	LBD21.31R	7250907	7251317	+	upstream	-3483
26	23	NA	LBD21.31R	7250907	7251317	+	upstream	-6041
26	23	NA	LBD21.31R	7250907	7251317	+	upstream	-6041
26	24	NA	LBD21.31R	7974628	7975279	+	downstrea	4338
17	15	NA	LBD21.31R	8144143	8149905	+	inside	2927
34	31	NA	LBD21.31R	8205831	8210012	-	inside	1101
29	24	NA	LBD21.31R	8776939	8780271	+	upstream	-3043

29	24	NA	LBD21.31R	8776939	8780271	+	upstream	-3043
21	18	NA	LBD21.31R	10542508	10549296	-	inside	1376
16	14	NA	LBD21.31R	11193574	11194068	-	inside	470
20	17	NA	LBD21.31R	11686122	11688658	+	downstrea	4230
17	15	NA	LBD21.31R	12678172	12681640	-	upstream	-2883
22	19	NA	LBD21.31R	13002911	13007361	+	upstream	-552
25	23	NA	LBD21.31R	13309004	13313082	-	inside	1916
30	28	NA	LBD21.31R	14208454	14211053	-	inside	433
17	14	NA	LBD21.31R	14911216	14914519	+	inside	74
22	21	NA	LBD21.31R	15314118	15322630	+	inside	4462
27	25	NA	LBD21.31R	15314118	15322630	+	inside	3964
20	18	NA	LBD21.31R	15358332	15361898	-	inside	3162
38	37	NA	LBD21.31R	379940	380409	+	inside	128
20	16	NA	LBD21.31R	1518098	1518765	-	downstrea	1711
20	18	NA	LBD21.31R	1782197	1783753	-	downstrea	5028
20	18	NA	LBD21.31R	1782197	1783753	-	downstrea	5028
17	15	NA	LBD21.31R	1985358	1989952	+	downstrea	5789
39	37	NA	LBD21.31R	2811985	2812920	-	upstream	-2098
16	13	NA	LBD21.31R	3564658	3566968	+	downstrea	2678
23	21	NA	LBD21.31R	3612661	3614813	-	upstream	-1244
23	21	NA	LBD21.31R	3612661	3614813	-	upstream	-1244
37	35	NA	LBD21.31R	3695827	3697846	-	downstrea	2264
28	25	NA	LBD21.31R	3776560	3779286	-	upstream	-2300
28	25	NA	LBD21.31R	3776560	3779286	-	upstream	-2300
19	17	NA	LBD21.31R	4476846	4478817	+	inside	44
20	17	NA	LBD21.31R	4513635	4514673	+	downstrea	3026
19	15	NA	LBD21.31R	5212150	5216225	-	inside	1750
18	15	NA	LBD21.31R	6064176	6065835	-	upstream	-5017
18	15	NA	LBD21.31R	6064176	6065835	-	upstream	-5017
21	17	NA	LBD21.31R	6162096	6169028	+	inside	1705
21	17	NA	LBD21.31R	6162096	6169028	+	inside	1705
17	14	NA	LBD21.31R	6626146	6628702	-	overlapEn	2762
17	14	NA	LBD21.31R	6626146	6628702	-	overlapEn	2762
22	19	NA	LBD21.31R	7843368	7843839	-	inside	375
17	14	NA	LBD21.31R	8011656	8012130	+	downstrea	540
17	14	NA	LBD21.31R	8011656	8012130	+	downstrea	540
22	19	NA	LBD21.31R	8017574	8018741	-	upstream	-6176
23	20	NA	LBD21.31R	8631059	8632295	-	inside	935
23	20	NA	LBD21.31R	8631059	8632295	-	inside	935
19	16	NA	LBD21.31R	9860569	9862692	+	downstrea	6560
19	16	NA	LBD21.31R	9860569	9862692	+	downstrea	6560
26	24	NA	LBD21.31R	11091933	11092256	+	downstrea	734
20	17	NA	LBD21.31R	11911824	11930638	+	upstream	-1549
33	29	NA	LBD21.31R	12097990	12102875	+	upstream	-4635
33	29	NA	LBD21.31R	12097990	12102875	+	upstream	-4635
20	18	NA	LBD21.31R	12401027	12405504	+	inside	84
19	16	NA	LBD21.31R	13019684	13023254	-	inside	2165
19	17	NA	LBD21.31R	14567016	14571279	+	inside	2361
29	26	NA	LBD21.31R	15126261	15127815	-	inside	332
17	15	NA	LBD21.31R	15742390	15744149	-	downstrea	11713
17	15	NA	LBD21.31R	15742390	15744149	-	downstrea	11713
30	26	NA	LBD21.31R	17239823	17240740	+	upstream	-960
30	26	NA	LBD21.31R	17239823	17240740	+	upstream	-960
30	28	NA	LBD21.31R	17239823	17240740	+	upstream	-4782

30	28	NA	LBD21.31R	17239823	17240740	+	upstream	-4782
26	24	NA	LBD21.31R	17478458	17484271	-	inside	731
46	45	NA	LBD21.31R	18790426	18792329	+	upstream	-10755
46	45	NA	LBD21.31R	18790426	18792329	+	upstream	-10755
28	26	NA	LBD21.31R	190781	191530	+	upstream	-2707
44	43	NA	LBD21.31R	238870	240371	+	downstrea	11972
16	12	NA	LBD21.31R	428906	430507	-	downstrea	14711
55	53	NA	LBD21.31R	428906	430507	-	downstrea	16426
61	59	NA	LBD21.31R	989221	989641	+	upstream	-1393
61	59	NA	LBD21.31R	989221	989641	+	upstream	-1393
61	59	NA	LBD21.31R	989221	989641	+	upstream	-1393
61	59	NA	LBD21.31R	989221	989641	+	upstream	-1393
39	37	NA	LBD21.31R	989221	989641	+	inside	148
39	37	NA	LBD21.31R	989221	989641	+	inside	148
39	37	NA	LBD21.31R	989221	989641	+	inside	148
39	37	NA	LBD21.31R	989221	989641	+	inside	148
39	38	NA	LBD21.31R	991605	991793	-	overlapEn	609
29	27	NA	LBD21.31R	1392167	1394565	+	upstream	-6758
29	27	NA	LBD21.31R	1392167	1394565	+	upstream	-6758
29	27	NA	LBD21.31R	1392167	1394565	+	upstream	-6758
52	50	NA	LBD21.31R	2730090	2730230	-	includeFe	203
52	50	NA	LBD21.31R	2730090	2730230	-	includeFe	203
52	50	NA	LBD21.31R	2730090	2730230	-	includeFe	203
52	50	NA	LBD21.31R	2730090	2730230	-	includeFe	203
33	31	NA	LBD21.31R	2730090	2730230	-	upstream	-490
33	31	NA	LBD21.31R	2730090	2730230	-	upstream	-490
33	31	NA	LBD21.31R	2730090	2730230	-	upstream	-490
33	31	NA	LBD21.31R	2730090	2730230	-	upstream	-490
25	22	NA	LBD21.31R	4112696	4116361	-	downstrea	4542
17	14	NA	LBD21.31R	5382460	5384224	+	downstrea	2210
17	14	NA	LBD21.31R	5478033	5483711	-	inside	1358
18	15	NA	LBD21.31R	5502089	5508765	+	upstream	-7611
18	16	NA	LBD21.31R	5853166	5857730	+	inside	30
18	16	NA	LBD21.31R	6590436	6593008	-	inside	1520
24	21	NA	LBD21.31R	6851601	6861463	-	inside	1344
55	53	NA	LBD21.31R	7142744	7143769	-	inside	361
17	14	NA	LBD21.31R	7516983	7520363	-	inside	433
33	32	NA	LBD21.31R	8624944	8627440	-	downstrea	6616
19	16	NA	LBD21.31R	10203623	10212959	-	upstream	-904
19	17	NA	LBD21.31R	10238649	10239156	-	upstream	-815
23	19	NA	LBD21.31R	10435173	10436054	+	overlapEn	737
23	19	NA	LBD21.31R	10435173	10436054	+	overlapEn	737
26	23	NA	LBD21.31R	11362951	11367004	-	overlapSt	697
31	28	NA	LBD21.31R	11407300	11410604	+	upstream	-3915
18	15	NA	LBD21.31R	11783112	11788425	-	upstream	-4876
18	15	NA	LBD21.31R	11783112	11788425	-	upstream	-4876
21	19	NA	LBD21.31R	11981991	11983282	-	upstream	-242
20	18	NA	LBD21.31R	12025721	12030665	+	inside	1197
17	15	NA	LBD21.31R	12157574	12165568	-	inside	1076
18	16	NA	LBD21.31R	12943202	12947095	+	downstrea	5078
17	15	NA	LBD21.31R	678213	681875	-	inside	3321
19	15	NA	LBD21.31R	2837752	2840631	-	upstream	-9086
72	68	NA	LBD21.31R	4155808	4161440	+	downstrea	57392
23	19	NA	LBD21.31R	4421608	4427087	-	inside	2223

37	35	NA	LBD21.31R	4663851	4675020	-	upstream	-77240
37	35	NA	LBD21.31R	4663851	4675020	-	upstream	-77240
23	20	NA	LBD21.31R	4663851	4675020	-	upstream	-20516
23	20	NA	LBD21.31R	4663851	4675020	-	upstream	-20516
34	31	NA	LBD21.31R	4663851	4675020	-	inside	4756
34	31	NA	LBD21.31R	4663851	4675020	-	inside	4756
103	101	NA	LBD21.31R	5229730	5230211	+	inside	47
37	33	NA	LBD21.31R	5669271	5671886	+	upstream	-53243
22	21	NA	LBD21.31R	5747967	5748499	+	inside	32
18	16	NA	LBD21.31R	7324413	7331352	+	inside	821
50	49	NA	LBD21.31R	8333019	8333763	+	inside	58
50	49	NA	LBD21.31R	8333019	8333763	+	inside	58
28	26	NA	LBD21.31R	8336268	8341257	+	upstream	-1585
28	26	NA	LBD21.31R	8336268	8341257	+	upstream	-1585
19	17	NA	LBD21.31R	10001785	10016269	+	inside	783
22	20	NA	LBD21.31R	10139477	10143131	-	inside	2688
20	19	NA	LBD21.31R	11303792	11304397	-	downstrea	2445
17	14	NA	LBD21.31R	11747167	11755290	+	downstrea	11356
20	17	NA	LBD21.31R	11844016	11848320	-	upstream	-793
29	26	NA	LBD21.31R	12503051	12507304	+	inside	3676
29	26	NA	LBD21.31R	12503051	12507304	+	inside	3676
17	14	NA	LBD21.31R	13116732	13120397	+	upstream	-7180
19	17	NA	LBD21.31R	13132830	13137057	+	inside	1433
19	17	NA	LBD21.31R	13132830	13137057	+	inside	1433
21	17	NA	LBD21.31R	13194526	13197475	+	upstream	-1604
17	14	NA	LBD21.31R	13640755	13644147	-	upstream	-4695
20	16	NA	LBD21.31R	13733411	13735324	+	downstrea	3023
18	15	NA	LBD21.31R	14802073	14803969	-	downstrea	3192
21	17	NA	LBD21.31R	15938397	15939837	+	inside	906
22	19	NA	LBD21.31R	16019876	16024647	-	upstream	-161
25	22	NA	LBD21.31R	17009869	17012319	+	upstream	-1521
17	15	NA	LBD21.31R	17098655	17100780	-	inside	479
33	29	NA	LBD21.31R	18066606	18069486	+	downstrea	3209
16	14	NA	LBD21.31R	18454527	18457379	+	upstream	-3869
21	18	NA	LBD21.31R	18925431	18927898	-	upstream	-6897
31	29	NA	LBD21.31R	19155230	19162196	+	inside	495
26	23	NA	LBD21.31R	19400650	19406402	-	upstream	-4938
41	39	NA	LBD21.31R	19761629	19769106	+	inside	3697
18	15	NA	LBD21.31R	20362434	20365631	+	upstream	-1442
18	15	NA	LBD21.31R	20362434	20365631	+	upstream	-1442
18	15	NA	LBD21.31R	20362434	20365631	+	upstream	-1442
20	17	NA	LBD21.31R	20747463	20750870	-	downstrea	4662
20	17	NA	LBD21.31R	20747463	20750870	-	downstrea	4662
17	15	NA	LBD21.31R	20849855	20856785	+	overlapSt	-146
20	17	NA	LBD21.31R	20955472	20956297	+	overlapSt	-228
20	17	NA	LBD21.31R	20955472	20956297	+	overlapSt	-228
18	14	NA	LBD21.31R	278846	281529	-	inside	1145
20	17	NA	LBD21.31R	1288198	1294255	-	inside	330
22	20	NA	LBD21.31R	6674943	6675394	+	downstrea	961
72	70	NA	LBD21.31R	7193480	7194553	-	inside	666
38	35	NA	LBD21.31R	7203770	7204270	-	upstream	-315
29	27	NA	LBD21.31R	7203770	7204270	-	inside	354
50	47	NA	LBD21.31R	7206842	7207063	-	overlapEn	336
48	47	NA	LBD21.31R	7215822	7217613	+	inside	473

48	46	NA	LBD21.31R	7215822	7217613	+	inside	1162
21	19	NA	LBD21.31R	7230312	7232353	-	inside	245
21	19	NA	LBD21.31R	7230312	7232353	-	inside	245
22	18	NA	LBD21.31R	8006037	8008475	-	downstrea	4059
42	40	NA	LBD21.31R	8288217	8297303	+	upstream	-6736
20	16	NA	LBD21.31R	8738465	8749355	-	upstream	-855
18	15	NA	LBD21.31R	11644313	11656304	-	inside	4813
20	18	NA	LBD21.31R	11644313	11656304	-	inside	9707
30	28	NA	LBD21.31R	12063003	12064255	+	inside	1032
17	14	NA	LBD21.31R	12867984	12868432	-	inside	264
38	36	NA	LBD21.31R	13861100	13861201	-	upstream	-82
18	16	NA	LBD21.31R	14618174	14623032	+	inside	1569
27	23	NA	LBD21.31R	15719348	15719855	+	overlapEn	86
41	39	NA	LBD21.31R	16057023	16060615	+	upstream	-1141
22	19	NA	LBD21.31R	16378944	16380645	-	downstrea	3874
19	15	NA	LBD21.31R	16785690	16786493	+	downstrea	1861
17	13	NA	LBD21.31R	17258225	17264952	+	upstream	-9657
27	25	NA	LBD21.31R	18049998	18051851	+	inside	18
18	16	NA	LBD21.31R	274497	275360	+	downstrea	4848
23	21	NA	LBD21.31R	878985	880332	+	upstream	-541
21	19	NA	LBD21.31R	1698979	1701858	-	inside	1018
16	14	NA	LBD21.31R	1790897	1791558	-	downstrea	4059
19	17	NA	LBD21.31R	1875251	1878254	-	inside	2527
19	17	NA	LBD21.31R	1875251	1878254	-	inside	2527
19	17	NA	LBD21.31R	2051060	2056456	+	inside	85
17	13	NA	LBD21.31R	3629109	3634234	+	inside	4183
19	15	NA	LBD21.31R	3756392	3763955	-	upstream	-4000
28	25	NA	LBD21.31R	3780252	3780497	-	upstream	-4907
18	15	NA	LBD21.31R	4986633	4992453	-	inside	3218
17	14	NA	LBD21.31R	5408743	5411011	+	upstream	-3498
18	16	NA	LBD21.31R	6940150	6960376	+	upstream	-48492
18	16	NA	LBD21.31R	6940150	6960376	+	upstream	-48492
23	20	NA	LBD21.31R	7540873	7541856	-	downstrea	54181
25	22	NA	LBD21.31R	8466107	8472367	+	downstrea	15430
25	22	NA	LBD21.31R	8466107	8472367	+	downstrea	15430
25	22	NA	LBD21.31R	8466107	8472367	+	downstrea	15430
17	15	NA	LBD21.31R	9823081	9826808	+	inside	2660
19	16	NA	LBD21.31R	9826733	9830338	-	upstream	-1987
20	16	NA	LBD21.31R	9855190	9858922	+	upstream	-4546
26	22	NA	LBD21.31R	10411438	10416356	-	upstream	-4768
26	22	NA	LBD21.31R	10411438	10416356	-	upstream	-4768
33	31	NA	LBD21.31R	10796766	10799160	+	upstream	-19183
35	30	NA	LBD21.31R	11137722	11139660	+	downstrea	6643
23	20	NA	LBD21.31R	11356226	11359696	+	upstream	-7049
19	17	NA	LBD21.31R	11682812	11684817	+	downstrea	2046
18	16	NA	LBD21.31R	12015845	12017020	+	upstream	-884
33	31	NA	LBD21.31R	12311205	12313557	-	inside	769
18	15	NA	LBD21.31R	12355907	12357365	-	upstream	-2591
18	15	NA	LBD21.31R	12355907	12357365	-	upstream	-2591
20	18	NA	LBD21.31R	12371433	12376015	-	inside	1298
20	18	NA	LBD21.31R	12371433	12376015	-	inside	1298
20	18	NA	LBD21.31R	12553770	12557943	+	inside	1777
30	28	NA	LBD21.31R	12868554	12869907	-	upstream	-2764
20	18	NA	LBD21.31R	12894761	12896793	-	upstream	-675

22	20	NA	LBD21.31R	14117032	14119973	+	downstrea	3114
16	14	NA	LBD21.31R	14196754	14206880	+	inside	2080
17	15	NA	LBD21.31R	14735670	14736753	+	downstrea	1182
19	16	NA	LBD21.31R	14777432	14782106	-	inside	1724
16	14	NA	LBD21.31R	14961839	14964665	+	downstrea	2936
19	17	NA	LBD21.31R	14970440	14975257	-	inside	2188
34	32	NA	LBD21.31R	15170317	15170755	+	upstream	-1737
19	16	NA	LBD21.31R	15237741	15239875	-	overlapSt	175
23	19	NA	LBD21.31R	15373698	15379797	-	upstream	-578
17	15	NA	LBD21.31R	15582384	15584094	-	inside	1541
18	16	NA	LBD21.31R	15704881	15708099	-	inside	1943
21	19	NA	LBD21.31R	634239	637149	+	inside	1142
18	15	NA	LBD21.31R	773339	783010	-	upstream	-9698
27	23	NA	LBD21.31R	1135684	1136628	-	upstream	-2572
36	35	NA	LBD21.31R	1673482	1677412	+	inside	2817
20	18	NA	LBD21.31R	1741029	1744938	+	upstream	-2567
21	19	NA	LBD21.31R	3353107	3353361	+	downstrea	7361
27	24	NA	LBD21.31R	3621041	3622906	-	inside	1506
18	16	NA	LBD21.31R	3771491	3782070	+	inside	8240
28	25	NA	LBD21.31R	3991611	3996963	+	inside	763
64	62	NA	LBD21.31R	5311388	5311926	-	downstrea	14972
21	19	NA	LBD21.31R	5972786	5973658	+	downstrea	1087
40	39	NA	LBD21.31R	6402922	6404074	+	upstream	-641
40	39	NA	LBD21.31R	6402922	6404074	+	upstream	-641
25	24	NA	LBD21.31R	6402922	6404074	+	inside	156
25	24	NA	LBD21.31R	6402922	6404074	+	inside	156
67	65	NA	LBD21.31R	6404076	6408104	+	overlapSt	-158
30	29	NA	LBD21.31R	6408512	6409298	+	includeFe	-75
30	29	NA	LBD21.31R	6410411	6410767	+	overlapSt	-362
43	42	NA	LBD21.31R	6413755	6414887	+	inside	517
18	16	NA	LBD21.31R	6539055	6540229	-	inside	877
17	15	NA	LBD21.31R	8382096	8385123	-	upstream	-13179
22	20	NA	LBD21.31R	8382096	8385123	-	upstream	-11984
18	16	NA	LBD21.31R	8652583	8677213	+	inside	24049
33	32	NA	LBD21.31R	9585458	9591300	-	upstream	-701
33	32	NA	LBD21.31R	9585458	9591300	-	upstream	-701
43	42	NA	LBD21.31R	9857154	9857592	-	inside	426
48	45	NA	LBD21.31R	10736488	10737257	-	downstrea	1091
48	45	NA	LBD21.31R	10736488	10737257	-	downstrea	1091
22	19	NA	LBD21.31R	10736488	10737257	-	upstream	-1068
22	19	NA	LBD21.31R	10736488	10737257	-	upstream	-1068
18	14	NA	LBD21.31R	11147296	11150772	+	overlapEn	3268
19	15	NA	LBD21.31R	11160987	11170428	-	upstream	-19841
19	17	NA	LBD21.31R	12673696	12675672	-	upstream	-587
17	15	NA	LBD21.31R	12941227	12944199	+	inside	823
20	18	NA	LBD21.31R	13248572	13251015	+	downstrea	5226
17	15	NA	LBD21.31R	13248572	13251015	+	upstream	-4109
24	22	NA	LBD21.31R	13611002	13611721	-	upstream	-2503
51	49	NA	LBD21.31R	13971000	13976252	+	upstream	-3376
38	36	NA	LBD21.31R	14518609	14522174	-	upstream	-693
27	26	NA	LBD21.31R	14666884	14667419	-	overlapEn	1027
27	26	NA	LBD21.31R	14666884	14667419	-	overlapEn	1027
21	19	NA	LBD21.31R	14683663	14685457	-	inside	855
43	42	NA	LBD21.31R	14754882	14755349	-	includeFe	500

73	71	NA	LBD21.31R	14757219	14757726	+	downstrea	734
73	71	NA	LBD21.31R	14757219	14757726	+	downstrea	734
60	58	NA	LBD21.31R	14765441	14765638	-	overlapSt	129
60	58	NA	LBD21.31R	14765441	14765638	-	overlapSt	129
60	58	NA	LBD21.31R	14765441	14765638	-	overlapSt	129
37	36	NA	LBD21.31R	14765441	14765638	-	upstream	-2662
37	36	NA	LBD21.31R	14765441	14765638	-	upstream	-2662
37	36	NA	LBD21.31R	14765441	14765638	-	upstream	-2662
91	89	NA	LBD21.31R	14765441	14765638	-	upstream	-1888
91	89	NA	LBD21.31R	14765441	14765638	-	upstream	-1888
91	89	NA	LBD21.31R	14765441	14765638	-	upstream	-1888
43	42	NA	LBD21.31R	14771293	14771695	-	downstrea	1616
43	42	NA	LBD21.31R	14771293	14771695	-	downstrea	1616
26	25	NA	LBD21.31R	14772455	14773204	+	downstrea	1088
26	25	NA	LBD21.31R	14772455	14773204	+	downstrea	1088
50	49	NA	LBD21.31R	14772455	14773204	+	inside	93
50	49	NA	LBD21.31R	14772455	14773204	+	inside	93
43	42	NA	LBD21.31R	14775554	14776118	-	inside	561
59	56	NA	LBD21.31R	14779861	14780816	-	downstrea	1703
122	119	NA	LBD21.31R	14783992	14784405	-	upstream	-854
122	119	NA	LBD21.31R	14783992	14784405	-	upstream	-854
27	26	NA	LBD21.31R	14788312	14789160	+	overlapEn	296
42	41	NA	LBD21.31R	14790129	14790383	-	overlapSt	7
42	41	NA	LBD21.31R	14790129	14790383	-	overlapSt	7
28	27	NA	LBD21.31R	14790129	14790383	-	overlapEn	843
28	27	NA	LBD21.31R	14790129	14790383	-	overlapEn	843
60	58	NA	LBD21.31R	14790129	14790383	-	upstream	-503
60	58	NA	LBD21.31R	14790129	14790383	-	upstream	-503
28	27	NA	LBD21.31R	14791829	14792637	+	inside	267
31	30	NA	LBD21.31R	14794072	14795129	+	includeFe	-399
45	43	NA	LBD21.31R	14814563	14814898	+	upstream	-453
62	60	NA	LBD21.31R	14836710	14837115	-	overlapEn	466
62	60	NA	LBD21.31R	14836710	14837115	-	overlapEn	466
33	31	NA	LBD21.31R	14837889	14838632	+	downstrea	1851
33	31	NA	LBD21.31R	14837889	14838632	+	downstrea	1851
61	59	NA	LBD21.31R	14837889	14838632	+	downstrea	1032
61	59	NA	LBD21.31R	14837889	14838632	+	downstrea	1032
44	43	NA	LBD21.31R	14844509	14845329	-	downstrea	3076
44	43	NA	LBD21.31R	14844509	14845329	-	downstrea	3076
44	43	NA	LBD21.31R	14844509	14845329	-	downstrea	3076
38	37	NA	LBD21.31R	14844509	14845329	-	downstrea	2257
38	37	NA	LBD21.31R	14844509	14845329	-	downstrea	2257
38	37	NA	LBD21.31R	14844509	14845329	-	downstrea	2257
29	28	NA	LBD21.31R	14844509	14845329	-	overlapSt	437
29	28	NA	LBD21.31R	14844509	14845329	-	overlapSt	437
29	28	NA	LBD21.31R	14844509	14845329	-	overlapSt	437
30	29	NA	LBD21.31R	14849394	14849797	+	includeFe	-279
30	29	NA	LBD21.31R	14849394	14849797	+	includeFe	-279
54	52	NA	LBD21.31R	14851611	14852408	+	inside	346
47	46	NA	LBD21.31R	14853384	14854012	+	inside	151
47	46	NA	LBD21.31R	14853384	14854012	+	inside	151
28	27	NA	LBD21.31R	14867350	14867454	+	includeFe	-243
28	27	NA	LBD21.31R	14867350	14867454	+	includeFe	-243
54	52	NA	LBD21.31R	14867350	14867454	+	upstream	-1020

54	52	NA	LBD21.31R	14867350	14867454	+	upstream	-1020
45	44	NA	LBD21.31R	14880122	14880328	-	includeFe	652
45	44	NA	LBD21.31R	14880122	14880328	-	includeFe	652
85	82	NA	LBD21.31R	14882770	14884113	+	inside	325
85	82	NA	LBD21.31R	14882770	14884113	+	inside	325
74	72	NA	LBD21.31R	14894928	14896038	+	downstrea	1204
37	35	NA	LBD21.31R	14901898	14907618	+	inside	2827
37	35	NA	LBD21.31R	14901898	14907618	+	inside	2827
37	35	NA	LBD21.31R	14901898	14907618	+	inside	2827
39	37	NA	LBD21.31R	14901898	14907618	+	inside	5457
39	37	NA	LBD21.31R	14901898	14907618	+	inside	5457
39	37	NA	LBD21.31R	14901898	14907618	+	inside	5457
20	17	NA	LBD21.31R	14996475	15001272	+	upstream	-8927
20	17	NA	LBD21.31R	14996475	15001272	+	upstream	-8927
37	35	NA	LBD21.31R	15832819	15835201	+	downstrea	3705
23	20	NA	LBD21.31R	16066967	16067796	+	downstrea	2843
28	23	NA	LBD21.31R	16162975	16169041	+	inside	1356
18	15	NA	LBD21.31R	731206	735001	-	upstream	-917
35	30	NA	LBD21.31R	1489482	1493387	-	inside	1236
35	30	NA	LBD21.31R	1489482	1493387	-	inside	1236
19	17	NA	LBD21.31R	1629064	1635605	-	upstream	-1948
19	17	NA	LBD21.31R	1629064	1635605	-	upstream	-1948
18	15	NA	LBD21.31R	2169470	2171490	+	downstrea	2665
18	15	NA	LBD21.31R	2169470	2171490	+	downstrea	2665
19	16	NA	LBD21.31R	3318499	3326082	-	upstream	-5328
26	24	NA	LBD21.31R	4238803	4238982	+	downstrea	1380
28	26	NA	LBD21.31R	4255330	4258195	+	inside	886
19	16	NA	LBD21.31R	5351230	5353789	-	upstream	-3118
17	15	NA	LBD21.31R	6226331	6230401	-	upstream	-1904
28	25	NA	LBD21.31R	6385919	6393427	+	inside	578
35	33	NA	LBD21.31R	6429060	6430243	-	upstream	-941
37	35	NA	LBD21.31R	6968454	6973051	-	upstream	-2105
19	16	NA	LBD21.31R	6980125	6985518	-	inside	2494
17	14	NA	LBD21.31R	7287631	7287810	-	upstream	-4075
18	16	NA	LBD21.31R	8140603	8148322	-	inside	1187
19	16	NA	LBD21.31R	9199617	9200053	-	upstream	-1041
30	28	NA	LBD21.31R	9349076	9356575	+	upstream	-3941
30	28	NA	LBD21.31R	9349076	9356575	+	upstream	-3941
42	38	NA	LBD21.31R	10403873	10405807	-	upstream	-134
42	38	NA	LBD21.31R	10403873	10405807	-	upstream	-134
40	38	NA	LBD21.31R	11073156	11075291	-	upstream	-1908
40	38	NA	LBD21.31R	11073156	11075291	-	upstream	-1908
26	22	NA	LBD21.31R	12397621	12399479	+	upstream	-495
26	22	NA	LBD21.31R	12397621	12399479	+	upstream	-495
22	19	NA	LBD21.31R	12601305	12605522	-	inside	4013
24	20	NA	LBD21.31R	12925765	12927225	+	downstrea	20533
24	20	NA	LBD21.31R	12925765	12927225	+	downstrea	20533
17	14	NA	LBD21.31R	14743200	14748976	-	upstream	-26870
19	15	NA	LBD21.31R	15176521	15179747	-	upstream	-156
56	54	NA	LBD21.31R	15925039	15932369	-	upstream	-1124
38	37	NA	LBD21.31R	16505398	16505793	-	upstream	-15231
17	16	NA	LBD21.31R	16984035	16984962	-	downstrea	6064
17	16	NA	LBD21.31R	16984035	16984962	-	downstrea	6064
17	16	NA	LBD21.31R	16984035	16984962	-	downstrea	6064

17	16	NA	LBD21.31R	16984035	16984962	-	downstrea	6064
17	16	NA	LBD21.31R	16984035	16984962	-	downstrea	6064
20	17	NA	LBD21.31R	17226201	17227664	+	upstream	-35176
40	38	NA	LBD21.31R	17226201	17227664	+	upstream	-10804
25	23	NA	LBD21.31R	18139525	18146950	-	downstrea	11254
35	32	NA	LBD21.31R	18318753	18326192	-	upstream	-6825
17	15	NA	LBD21.31R	18419460	18428607	+	inside	161
18	15	NA	LBD21.31R	458876	461241	+	downstrea	2997
27	24	NA	LBD21.31R	1858280	1862197	+	upstream	-3716
17	15	NA	LBD21.31R	1899112	1904272	+	inside	578
18	16	NA	LBD21.31R	2345768	2353767	+	inside	122
19	16	NA	LBD21.31R	3510981	3513035	-	downstrea	12287
23	20	NA	LBD21.31R	4011094	4014183	+	upstream	-2068
22	20	NA	LBD21.31R	4095980	4102909	+	inside	3001
31	30	NA	LBD21.31R	4554427	4560533	-	inside	2496
20	16	NA	LBD21.31R	4970325	4977849	+	upstream	-21702
20	16	NA	LBD21.31R	4970325	4977849	+	upstream	-21702
20	16	NA	LBD21.31R	4970325	4977849	+	upstream	-21702
19	15	NA	LBD21.31R	4970325	4977849	+	upstream	-25849
19	15	NA	LBD21.31R	4970325	4977849	+	upstream	-25849
19	15	NA	LBD21.31R	4970325	4977849	+	upstream	-25849
65	60	NA	LBD21.31R	4970325	4977849	+	upstream	-2435
65	60	NA	LBD21.31R	4970325	4977849	+	upstream	-2435
65	60	NA	LBD21.31R	4970325	4977849	+	upstream	-2435
26	24	NA	LBD21.31R	6288546	6289859	-	inside	230
26	24	NA	LBD21.31R	6288546	6289859	-	inside	230
26	24	NA	LBD21.31R	6288546	6289859	-	inside	230
22	18	NA	LBD21.31R	6288546	6289859	-	downstrea	157304
22	18	NA	LBD21.31R	6288546	6289859	-	downstrea	157304
22	18	NA	LBD21.31R	6288546	6289859	-	downstrea	157304
17	16	NA	LBD21.31R	6322447	6322818	+	downstrea	2103
17	16	NA	LBD21.31R	6322447	6322818	+	downstrea	2103
50	48	NA	LBD21.31R	6322447	6322818	+	overlapEn	34
50	48	NA	LBD21.31R	6322447	6322818	+	overlapEn	34
19	18	NA	LBD21.31R	6328465	6329003	+	downstrea	2543
19	18	NA	LBD21.31R	6328465	6329003	+	downstrea	2543
19	18	NA	LBD21.31R	6328465	6329003	+	downstrea	2543
19	18	NA	LBD21.31R	6328465	6329003	+	downstrea	2543
19	18	NA	LBD21.31R	6328465	6329003	+	downstrea	2543
20	19	NA	LBD21.31R	6328465	6329003	+	inside	70
20	19	NA	LBD21.31R	6328465	6329003	+	inside	70
20	19	NA	LBD21.31R	6328465	6329003	+	inside	70
20	19	NA	LBD21.31R	6328465	6329003	+	inside	70
20	19	NA	LBD21.31R	6328465	6329003	+	inside	70
21	20	NA	LBD21.31R	6328465	6329003	+	upstream	-1206
21	20	NA	LBD21.31R	6328465	6329003	+	upstream	-1206
21	20	NA	LBD21.31R	6328465	6329003	+	upstream	-1206
21	20	NA	LBD21.31R	6328465	6329003	+	upstream	-1206
21	20	NA	LBD21.31R	6328465	6329003	+	upstream	-1206
22	21	NA	LBD21.31R	6328465	6329003	+	downstrea	1355
22	21	NA	LBD21.31R	6328465	6329003	+	downstrea	1355
22	21	NA	LBD21.31R	6328465	6329003	+	downstrea	1355
22	21	NA	LBD21.31R	6328465	6329003	+	downstrea	1355
22	21	NA	LBD21.31R	6328465	6329003	+	downstrea	1355

41	40	NA	LBD21.31R	6328465	6329003	+	upstream	-2601
41	40	NA	LBD21.31R	6328465	6329003	+	upstream	-2601
41	40	NA	LBD21.31R	6328465	6329003	+	upstream	-2601
41	40	NA	LBD21.31R	6328465	6329003	+	upstream	-2601
41	40	NA	LBD21.31R	6328465	6329003	+	upstream	-2601
62	60	NA	LBD21.31R	6328465	6329003	+	upstream	-1951
62	60	NA	LBD21.31R	6328465	6329003	+	upstream	-1951
62	60	NA	LBD21.31R	6328465	6329003	+	upstream	-1951
62	60	NA	LBD21.31R	6328465	6329003	+	upstream	-1951
62	60	NA	LBD21.31R	6328465	6329003	+	upstream	-1951
28	26	NA	LBD21.31R	6406439	6408531	+	downstrea	2762
28	26	NA	LBD21.31R	6406439	6408531	+	downstrea	2762
18	16	NA	LBD21.31R	6581611	6581823	-	upstream	-6846
17	15	NA	LBD21.31R	6631345	6632413	-	upstream	-49765
22	19	NA	LBD21.31R	6956049	6957260	-	upstream	-12289
16	12	NA	LBD21.31R	7437985	7438842	-	upstream	-5473
20	17	NA	LBD21.31R	8381611	8388412	-	overlapSt	93
20	17	NA	LBD21.31R	8381611	8388412	-	overlapSt	93
31	28	NA	LBD21.31R	8886714	8888635	-	inside	1518
50	48	NA	LBD21.31R	10235060	10235369	+	inside	25
17	14	NA	LBD21.31R	11320982	11321489	-	inside	358
21	17	NA	LBD21.31R	12317986	12322365	+	upstream	-4275
21	19	NA	LBD21.31R	13653206	13657755	+	inside	445
39	35	NA	LBD21.31R	13712792	13715704	+	upstream	-336
25	22	NA	LBD21.31R	14297144	14299677	+	downstrea	3606
19	17	NA	LBD21.31R	14911353	14916935	-	upstream	-1294
17	15	NA	LBD21.31R	15243480	15246532	+	upstream	-1961
25	21	NA	LBD21.31R	626427	626889	-	downstrea	1805
23	21	NA	LBD21.31R	626427	626889	-	upstream	-699
23	21	NA	LBD21.31R	904073	905624	+	inside	1272
21	18	NA	LBD21.31R	1868366	1869367	-	downstrea	1897
19	16	NA	LBD21.31R	3541745	3542526	+	overlapEn	685
17	15	NA	LBD21.31R	3795851	3796694	-	inside	685
18	16	NA	LBD21.31R	4115461	4120192	-	inside	328
24	22	NA	LBD21.31R	4266555	4267758	-	inside	700
26	22	NA	LBD21.31R	4953185	4960792	+	upstream	-4960
39	36	NA	LBD21.31R	6097845	6099951	+	inside	1046
21	19	NA	LBD21.31R	6579485	6580823	+	inside	299
17	15	NA	LBD21.31R	6677891	6681323	+	inside	853
32	30	NA	LBD21.31R	7275771	7277504	-	inside	1680
32	30	NA	LBD21.31R	7710855	7711361	+	upstream	-21034
32	30	NA	LBD21.31R	7710855	7711361	+	upstream	-21034
32	30	NA	LBD21.31R	7710855	7711361	+	upstream	-21034
21	19	NA	LBD21.31R	8101978	8102394	+	downstrea	28707
45	43	NA	LBD21.31R	8218191	8219153	+	inside	71
24	22	NA	LBD21.31R	11100066	11100463	-	downstrea	4944
48	45	NA	LBD21.31R	12343379	12350042	-	inside	659
21	19	NA	LBD21.31R	12539743	12543487	+	inside	1275
19	16	NA	LBD21.31R	13767533	13768493	-	upstream	-2846
17	14	NA	LBD21.31R	13989680	13990909	-	upstream	-1717
17	14	NA	LBD21.31R	13989680	13990909	-	upstream	-1717
20	17	NA	LBD21.31R	804971	809233	+	upstream	-614
19	17	NA	LBD21.31R	2038429	2045055	+	inside	950
18	16	NA	LBD21.31R	2256891	2262332	+	inside	4703

20	18	NA	LBD21.31R	2353070	2353586	+	downstrea	2574
18	16	NA	LBD21.31R	2668624	2669811	+	upstream	-3575
24	20	NA	LBD21.31R	3583593	3583901	-	downstrea	1770
24	20	NA	LBD21.31R	3583593	3583901	-	downstrea	1770
17	14	NA	LBD21.31R	5642080	5644665	-	upstream	-9837
46	44	NA	LBD21.31R	5860500	5863375	-	upstream	-5250
58	56	NA	LBD21.31R	5940893	5941428	+	inside	21
18	15	NA	LBD21.31R	5940893	5941428	+	upstream	-14910
20	18	NA	LBD21.31R	6937683	6944376	+	inside	2148
87	83	NA	LBD21.31R	7261738	7264096	+	downstrea	38277
54	52	NA	LBD21.31R	7364780	7365135	-	downstrea	6312
23	20	NA	LBD21.31R	8296712	8297045	+	upstream	-3668
34	32	NA	LBD21.31R	8764503	8766101	-	upstream	-2971
34	32	NA	LBD21.31R	8764503	8766101	-	upstream	-2971
34	32	NA	LBD21.31R	8764503	8766101	-	upstream	-2971
17	15	NA	LBD21.31R	9599828	9601523	-	downstrea	3732
17	15	NA	LBD21.31R	9599828	9601523	-	downstrea	3732
17	15	NA	LBD21.31R	9599828	9601523	-	downstrea	3732
17	14	NA	LBD21.31R	9635769	9638227	+	upstream	-1285
55	53	NA	LBD21.31R	10065285	10065639	+	upstream	-10217
18	16	NA	LBD21.31R	10947583	10953970	+	upstream	-9111
21	18	NA	LBD21.31R	10947583	10953970	+	upstream	-9806
19	17	NA	LBD21.31R	11500621	11508966	-	inside	960
20	18	NA	LBD21.31R	13012869	13015325	+	upstream	-8077
24	21	NA	LBD21.31R	13150818	13156037	+	upstream	-4687
30	28	NA	LBD21.31R	13528321	13532208	-	upstream	-1452
18	15	NA	LBD21.31R	14201130	14201727	+	inside	169
49	47	NA	LBD21.31R	14835641	14836690	-	upstream	-102
49	47	NA	LBD21.31R	14835641	14836690	-	upstream	-102
31	29	NA	LBD21.31R	14838177	14839672	+	upstream	-728
21	20	NA	LBD21.31R	15112186	15115017	-	downstrea	13530
17	15	NA	LBD21.31R	15517239	15520067	+	inside	1536
17	15	NA	LBD21.31R	1274099	1276741	-	upstream	-2066
17	15	NA	LBD21.31R	2319954	2322915	-	overlapSt	253
20	17	NA	LBD21.31R	2345287	2346896	-	downstrea	3002
31	30	NA	LBD21.31R	2436803	2437920	+	inside	532
18	15	NA	LBD21.31R	2680940	2682556	-	upstream	-1710
17	14	NA	LBD21.31R	3014831	3018941	+	upstream	-592
17	14	NA	LBD21.31R	3014831	3018941	+	upstream	-592
53	51	NA	LBD21.31R	3119582	3122945	+	upstream	-11915
35	30	NA	LBD21.31R	3234742	3238131	+	upstream	-4905
22	21	NA	LBD21.31R	4061102	4061935	-	inside	593
22	19	NA	LBD21.31R	4122359	4129934	+	upstream	-10005
22	19	NA	LBD21.31R	4122359	4129934	+	upstream	-10005
22	19	NA	LBD21.31R	4122359	4129934	+	upstream	-10005
22	19	NA	LBD21.31R	4122359	4129934	+	upstream	-10005
23	21	NA	LBD21.31R	4676602	4678662	-	upstream	-12236
34	32	NA	LBD21.31R	5006621	5007839	-	upstream	-23849
89	86	NA	LBD21.31R	5252831	5256854	+	upstream	-10454
17	14	NA	LBD21.31R	6365527	6368967	-	upstream	-31381
17	14	NA	LBD21.31R	6365527	6368967	-	upstream	-31381
17	14	NA	LBD21.31R	6365527	6368967	-	upstream	-31381
17	14	NA	LBD21.31R	6365527	6368967	-	upstream	-31381
70	65	NA	LBD21.31R	6532988	6537493	+	downstrea	84851

70	65	NA	LBD21.31R	6532988	6537493	+	downstrea	84851
70	65	NA	LBD21.31R	6532988	6537493	+	downstrea	84851
43	41	NA	LBD21.31R	6532988	6537493	+	downstrea	75441
43	41	NA	LBD21.31R	6532988	6537493	+	downstrea	75441
43	41	NA	LBD21.31R	6532988	6537493	+	downstrea	75441
18	17	NA	LBD21.31R	7374464	7374985	+	upstream	-18432
18	17	NA	LBD21.31R	7374464	7374985	+	upstream	-18432
18	17	NA	LBD21.31R	7766617	7770793	+	inside	1558
18	17	NA	LBD21.31R	7766617	7770793	+	inside	1558
18	15	NA	LBD21.31R	8133687	8142269	-	downstrea	11574
26	23	NA	LBD21.31R	11640875	11642874	-	downstrea	2373
57	52	NA	LBD21.31R	11697912	11699851	-	upstream	-6627
22	19	NA	LBD21.31R	12568154	12573071	+	upstream	-8804
22	19	NA	LBD21.31R	12568154	12573071	+	upstream	-8804
22	19	NA	LBD21.31R	12568154	12573071	+	upstream	-8804
22	19	NA	LBD21.31R	12568154	12573071	+	upstream	-8804
18	14	NA	LBD21.31R	12643275	12644045	-	downstrea	3441
18	16	NA	LBD21.31R	12643275	12644045	-	downstrea	1144
22	19	NA	LBD21.31R	12799233	12802179	-	downstrea	4768
18	14	NA	LBD21.31R	13029141	13030024	-	upstream	-3665
18	14	NA	LBD21.31R	15515402	15516727	+	downstrea	1953
21	19	NA	LBD21.31R	15590333	15591232	-	upstream	-321
21	19	NA	LBD21.31R	286427	288019	-	overlapEn	1802
21	18	NA	LBD21.31R	457316	459522	+	overlapSt	-9
19	16	NA	LBD21.31R	1432709	1436609	+	upstream	-826
19	16	NA	LBD21.31R	1432709	1436609	+	upstream	-826
26	22	NA	LBD21.31R	1992431	1994565	-	upstream	-4017
25	23	NA	LBD21.31R	2145453	2153015	+	upstream	-2205
17	14	NA	LBD21.31R	2174154	2175874	+	inside	1418
16	14	NA	LBD21.31R	2309317	2313931	+	inside	1242
32	30	NA	LBD21.31R	2751574	2757351	+	inside	79
22	20	NA	LBD21.31R	3188159	3189958	+	inside	67
22	20	NA	LBD21.31R	3188159	3189958	+	inside	67
39	37	NA	LBD21.31R	3192473	3193492	+	downstrea	1496
39	37	NA	LBD21.31R	3192473	3193492	+	downstrea	1496
64	61	NA	LBD21.31R	3192473	3193492	+	inside	434
64	61	NA	LBD21.31R	3192473	3193492	+	inside	434
29	28	NA	LBD21.31R	3194810	3195886	-	inside	996
92	90	NA	LBD21.31R	3201672	3202760	-	upstream	-141
92	90	NA	LBD21.31R	3201672	3202760	-	upstream	-141
21	19	NA	LBD21.31R	3830946	3835094	+	inside	935
19	16	NA	LBD21.31R	4758852	4759878	+	downstrea	6561
19	16	NA	LBD21.31R	4758852	4759878	+	downstrea	6561
19	16	NA	LBD21.31R	4758852	4759878	+	downstrea	6561
40	38	NA	LBD21.31R	4777056	4777541	-	downstrea	1885
80	78	NA	LBD21.31R	5320857	5321204	+	inside	18
29	28	NA	LBD21.31R	5539256	5540996	-	upstream	-4997
40	38	NA	LBD21.31R	6688729	6691334	-	overlapSt	271
34	29	NA	LBD21.31R	7540268	7542125	+	upstream	-4428
50	48	NA	LBD21.31R	7839613	7849712	-	downstrea	15219
50	48	NA	LBD21.31R	7839613	7849712	-	downstrea	15219
21	20	NA	LBD21.31R	7839613	7849712	-	downstrea	15674
21	20	NA	LBD21.31R	7839613	7849712	-	downstrea	15674
27	23	NA	LBD21.31R	9984160	9985937	-	upstream	-73

27	23	NA	LBD21.31R	9984160	9985937	-	upstream	-73
27	23	NA	LBD21.31R	9984160	9985937	-	upstream	-73
27	23	NA	LBD21.31R	9984160	9985937	-	upstream	-73
30	29	NA	LBD21.31R	9984160	9985937	-	upstream	-783
30	29	NA	LBD21.31R	9984160	9985937	-	upstream	-783
30	29	NA	LBD21.31R	9984160	9985937	-	upstream	-783
30	29	NA	LBD21.31R	9984160	9985937	-	upstream	-783
18	15	NA	LBD21.31R	10011999	10016353	+	overlapSt	-175
17	14	NA	LBD21.31R	11353130	11356983	-	upstream	-2722
17	14	NA	LBD21.31R	11353130	11356983	-	upstream	-2722
28	24	NA	LBD21.31R	11796617	11800728	-	upstream	-549
28	24	NA	LBD21.31R	11796617	11800728	-	upstream	-549
28	24	NA	LBD21.31R	11796617	11800728	-	upstream	-549
27	24	NA	LBD21.31R	12330740	12331554	-	upstream	-58
21	17	NA	LBD21.31R	12958757	12961224	+	overlapSt	-203
30	26	NA	LBD21.31R	13059593	13062592	+	overlapEn	2957
30	26	NA	LBD21.31R	13059593	13062592	+	overlapEn	2957
20	17	NA	LBD21.31R	13306929	13311828	-	upstream	-4851
19	17	NA	LBD21.31R	15371119	15377454	+	inside	347
58	56	NA	LBD21.31R	193697	197138	-	upstream	-38817
58	56	NA	LBD21.31R	193697	197138	-	upstream	-38817
44	42	NA	LBD21.31R	435395	436960	-	upstream	-194
44	42	NA	LBD21.31R	435395	436960	-	upstream	-194
44	42	NA	LBD21.31R	435395	436960	-	upstream	-194
44	42	NA	LBD21.31R	435395	436960	-	upstream	-194
44	42	NA	LBD21.31R	435395	436960	-	upstream	-194
43	41	NA	LBD21.31R	435395	436960	-	inside	1247
43	41	NA	LBD21.31R	435395	436960	-	inside	1247
43	41	NA	LBD21.31R	435395	436960	-	inside	1247
43	41	NA	LBD21.31R	435395	436960	-	inside	1247
43	41	NA	LBD21.31R	435395	436960	-	inside	1247
31	30	NA	LBD21.31R	438693	439050	+	overlapEn	186
27	26	NA	LBD21.31R	746690	748017	-	downstrea	1767
27	25	NA	LBD21.31R	100631	101775	+	inside	541
27	25	NA	LBD21.31R	100631	101775	+	inside	541
22	20	NA	LBD21.31R	386255	388907	-	upstream	-7539
37	35	NA	LBD21.31R	474713	477251	-	downstrea	10656
39	36	NA	LBD21.31R	185949	186314	-	upstream	-6499
30	27	NA	LBD21.31R	230695	231069	-	downstrea	42848
30	27	NA	LBD21.31R	230695	231069	-	downstrea	42848
57	55	NA	LBD21.31R	230695	231069	-	downstrea	4351
57	55	NA	LBD21.31R	230695	231069	-	downstrea	4351
19	16	NA	LBD21.31R	125722	129365	+	inside	2027
31	28	NA	LBD21.31R	286887	287644	-	downstrea	57965
17	15	NA	LBD21.31R	286887	287644	-	downstrea	22180
18	17	NA	LBD21.31R	243811	244128	+	downstrea	2397
18	17	NA	LBD21.31R	243811	244128	+	downstrea	2397
18	17	NA	LBD21.31R	243811	244128	+	downstrea	2397
38	36	NA	LBD21.31R	243811	244128	+	downstrea	3251
38	36	NA	LBD21.31R	243811	244128	+	downstrea	3251
38	36	NA	LBD21.31R	243811	244128	+	downstrea	3251
24	22	NA	LBD21.31R	243811	244128	+	downstrea	4080
24	22	NA	LBD21.31R	243811	244128	+	downstrea	4080
24	22	NA	LBD21.31R	243811	244128	+	downstrea	4080

36	33	NA	LBD21.31R	261478	261795	+	downstrea	2119
47	45	NA	LBD21.31R	279248	279568	+	upstream	-7004
31	29	NA	LBD21.31R	96849	104538	+	downstrea	12432
31	29	NA	LBD21.31R	96849	104538	+	downstrea	12432
31	29	NA	LBD21.31R	96849	104538	+	downstrea	12432
31	29	NA	LBD21.31R	96849	104538	+	downstrea	12432
28	25	NA	LBD21.31R	96849	104538	+	upstream	-18008
28	25	NA	LBD21.31R	96849	104538	+	upstream	-18008
28	25	NA	LBD21.31R	96849	104538	+	upstream	-18008
28	25	NA	LBD21.31R	96849	104538	+	upstream	-18008
24	22	NA	LBD21.31R	150794	155589	-	inside	2059
40	38	NA	LBD21.31R	54850	60572	-	downstrea	9191
17	13	NA	LBD21.31R	194889	197500	-	inside	558
31	28	NA	LBD21.31R	183283	183870	+	downstrea	4297
29	27	NA	LBD21.31R	194973	196597	-	downstrea	13200
17	15	NA	LBD21.31R	113891	116786	+	upstream	-1219
17	15	NA	LBD21.31R	113891	116786	+	upstream	-1219
17	15	NA	LBD21.31R	113891	116786	+	upstream	-1219
21	19	NA	LBD21.31R	7821	17171	-	inside	5947
20	18	NA	LBD21.31R	7821	17171	-	inside	7166
46	45	NA	LBD21.31R	70232	71679	-	inside	303
18	16	NA	LBD21.31R	118786	126050	-	inside	1398
18	16	NA	LBD21.31R	18047	18426	+	upstream	-1164
17	14	NA	LBD21.31R	3275	4020	+	upstream	-1186
17	14	NA	LBD21.31R	3275	4020	+	upstream	-1186
17	14	NA	LBD21.31R	28036	33071	+	upstream	-11388
37	35	NA	LBD21.31R	62331	65349	+	upstream	-3779
18	17	NA	LBD21.31R	6537	8055	-	upstream	-6986
18	17	NA	LBD21.31R	6537	8055	-	upstream	-6986
19	18	NA	LBD21.31R	6537	8055	-	upstream	-7490
19	18	NA	LBD21.31R	6537	8055	-	upstream	-7490
33	32	NA	LBD21.31R	28859	31174	+	upstream	-5430
75	73	NA	LBD21.31R	61152	61778	+	inside	26
28	26	NA	LBD21.31R	46265	46630	+	downstrea	7788
42	40	NA	LBD21.31R	8978	13165	+	upstream	-880
22	20	NA	LBD21.31R	28580	30088	+	downstrea	9229
41	37	NA	LBD21.31R	70936	73058	-	downstrea	2942
34	31	NA	LBD21.31R	16345	19466	-	upstream	-1875
34	31	NA	LBD21.31R	16345	19466	-	upstream	-1875
19	16	NA	LBD21.31R	51460	53460	+	upstream	-3136
25	23	NA	LBD21.31R	15266	15851	+	upstream	-11408
22	21	NA	LBD21.31R	12142	13396	-	inside	1242
20	17	NA	LBD21.31R	4548	5317	-	downstrea	3639
20	17	NA	LBD21.31R	4548	5317	-	downstrea	3639
25	23	NA	LBD21.31R	327	706	-	upstream	-1286

shortestD	fromOverl	seqnames.	start.y	end.y	width.y	strand.y	peak.y	start_pos
2493	NearestLo	Chr01	932382	933100	719	+	423	931891
3610	NearestLo	Chr01	3623118	3623362	245	+	608	3608879
3610	NearestLo	Chr01	3628172	3628378	207	+	855	3608879
3610	NearestLo	Chr01	3620022	3620254	233	+	298	3608879
2877	NearestLo	Chr01	3641718	3642047	330	+	242	3655178
20	NearestLo	Chr01	4424546	4425097	552	+	51	4424461
214	NearestLo	Chr01	4817641	4817898	258	+	499	4817345
234	NearestLo	Chr01	5083634	5083913	280	+	317	5081114
30	NearestLo	Chr01	5164265	5164485	221	+	545	5163758
6630	NearestLo	Chr01	5524665	5524934	270	+	483	5525312
14	NearestLo	Chr01	5594338	5594595	258	+	456	5594300
75	NearestLo	Chr01	5711592	5711807	216	+	672	5712602
75	NearestLo	Chr01	5710948	5711167	220	+	181	5712602
650	NearestLo	Chr01	6538416	6538667	252	+	290	6537993
650	NearestLo	Chr01	6541710	6542782	1073	+	694	6537993
287	NearestLo	Chr01	7118107	7118453	347	+	9	7116848
287	NearestLo	Chr01	7117259	7117924	666	+	714	7116848
993	NearestLo	Chr01	7201934	7202679	746	+	218	7201940
993	NearestLo	Chr01	7202924	7203215	292	+	556	7201940
993	NearestLo	Chr01	7204199	7204513	315	+	285	7201940
2680	NearestLo	Chr01	9208067	9208368	302	+	434	9208104
1372	NearestLo	Chr01	9498024	9498686	663	+	123	9497091
314	NearestLo	Chr01	9858132	9858463	332	+	44	9857742
2290	NearestLo	Chr01	9965463	9965680	218	+	250	9962114
936	NearestLo	Chr01	11491589	11491873	285	+	52	11493369
19426	NearestLo	Chr01	11563671	11564502	832	+	367	11542659
1332	NearestLo	Chr01	12722889	12723395	507	+	405	12722218
654	NearestLo	Chr01	13562385	13562608	224	+	549	13556117
82	NearestLo	Chr01	15221222	15221616	395	+	750	15234652
82	NearestLo	Chr01	15234710	15234949	240	+	261	15234652
1987	NearestLo	Chr01	15637740	15638040	301	+	751	15639966
1333	NearestLo	Chr01	16183016	16183553	538	+	135	16179793
2781	NearestLo	Chr01	17041342	17041719	378	+	605	17034637
472	NearestLo	Chr01	17649298	17649900	603	+	24	17650319
15231	NearestLo	Chr01	18772112	18772436	325	+	356	18767919
29226	NearestLo	Chr01	18838608	18838830	223	+	474	18808260
4316	NearestLo	Chr01	19166498	19166760	263	+	742	19160680
35	NearestLo	Chr01	19852102	19852335	234	+	67	19852151
560	NearestLo	Chr01	20657523	20658097	575	+	161	20658471
1541	NearestLo	Chr01	20856646	20856858	213	+	569	20856525
5941	NearestLo	Chr01	20949663	20949904	242	+	366	20943768
936	NearestLo	Chr01	21097534	21097907	374	+	171	21096741
2272	NearestLo	Chr01	21734912	21735133	222	+	894	21731865
2272	NearestLo	Chr01	21738719	21739150	432	+	782	21731865
443	NearestLo	Chr01	22365401	22365687	287	+	960	22363237
1	NearestLo	Chr01	22842429	22842660	232	+	462	22842430
662	NearestLo	Chr01	23047875	23048101	227	+	480	23047697
1970	NearestLo	Chr01	24554743	24555000	258	+	588	24557303
151	NearestLo	Chr01	25022765	25023605	841	+	2	25021006
440	NearestLo	Chr01	26612919	26613167	249	+	34	26610971
5579	NearestLo	Chr01	27822217	27822455	239	+	862	27813645

4632	NearestLoChr01	27822217	27822455	239	+	862	27813645
2392	NearestLoChr01	28202895	28203148	254	+	11	28195252
3913	NearestLoChr01	28202895	28203148	254	+	11	28195252
601	NearestLoChr01	28659036	28659252	217	+	666	28656491
937	NearestLoChr01	28984788	28985001	214	+	854	28983611
36	NearestLoChr01	29390804	29391197	394	+	95	29390781
5345	NearestLoChr01	29745057	29745337	281	+	603	29748511
3463	NearestLoChr01	31447555	31447793	239	+	663	31452744
12	NearestLoChr01	33457590	33457806	217	+	203	33457530
1135	NearestLoChr01	33568599	33568886	288	+	490	33568341
246	NearestLoChr01	33976103	33976365	263	+	498	33974233
112	NearestLoChr01	34318385	34318712	328	+	353	34316929
112	NearestLoChr01	34316631	34316877	247	+	518	34316929
7059	NearestLoChr01	36686557	36686915	359	+	609	36660993
44	NearestLoChr01	36719112	36719614	503	+	45	36703023
2672	NearestLoChr01	36719112	36719614	503	+	45	36703023
972	NearestLoChr01	36774562	36774954	393	+	360	36770547
290	NearestLoChr01	38900288	38900494	207	+	872	38900791
3555	NearestLoChr01	40123072	40123284	213	+	237	40126837
3171	NearestLoChr01	40938389	40938666	278	+	655	40937912
1704	NearestLoChr01	41015927	41016246	320	+	26	41014239
3256	NearestLoChr01	41352504	41352705	202	+	783	41340927
7003	NearestLoChr01	41633635	41633865	231	+	125	41624735
2933	NearestLoChr01	41809038	41809891	854	+	14	41808763
2933	NearestLoChr01	41814930	41815289	360	+	117	41808763
2933	NearestLoChr01	41805451	41805852	402	+	151	41808763
4637	NearestLoChr01	42021621	42021829	209	+	740	42015598
3284	NearestLoChr01	43039185	43039452	268	+	431	43033745
317	NearestLoChr01	43726664	43727041	378	+	39	43726632
317	NearestLoChr01	43727361	43727931	571	+	241	43726632
317	NearestLoChr01	43729989	43730208	220	+	168	43726632
48	NearestLoChr01	43726664	43727041	378	+	39	43726632
48	NearestLoChr01	43727361	43727931	571	+	241	43726632
48	NearestLoChr01	43729989	43730208	220	+	168	43726632
31017	NearestLoChr01	45718963	45719168	206	+	292	45750532
31017	NearestLoChr01	45731444	45731649	206	+	238	45750532
10941	NearestLoChr01	45804771	45804975	205	+	391	45800670
3720	NearestLoChr01	45804771	45804975	205	+	391	45800670
4110	NearestLoChr01	47428469	47428961	493	+	803	47420222
4110	NearestLoChr01	47422165	47422964	800	+	679	47420222
4110	NearestLoChr01	47436597	47436939	343	+	3	47420222
2791	NearestLoChr01	48290385	48290860	476	+	491	48294396
1750	NearestLoChr01	48747733	48748097	365	+	863	48744474
1361	NearestLoChr01	48820207	48820500	294	+	669	48820373
3463	NearestLoChr01	49433969	49434323	355	+	733	49433744
996	NearestLoChr01	49657257	49657507	251	+	706	49658499
55	NearestLoChr02	242208	242892	685	+	1079	240044
5501	NearestLoChr02	1263996	1264275	280	+	1216	1269779
234	NearestLoChr02	1263996	1264275	280	+	1216	1269779
118	NearestLoChr02	1762071	1762642	572	+	1050	1760719
6948	NearestLoChr02	1824639	1824907	269	+	1226	1813265
9787	NearestLoChr02	1824639	1824907	269	+	1226	1813265
3590	NearestLoChr02	1828023	1828251	229	+	1088	1831033
555	NearestLoChr02	2564326	2564748	423	+	1124	2557986

27	NearestLo	Chr02	2560154	2560385	232	+	1078	2560092
1941	NearestLo	Chr02	2804834	2805345	512	+	1019	2804643
233	NearestLo	Chr02	2804834	2805345	512	+	1019	2804643
1284	NearestLo	Chr02	3266552	3266849	298	+	1511	3253001
574	NearestLo	Chr02	3483779	3484864	1086	+	1020	3483467
574	NearestLo	Chr02	3483063	3483321	259	+	1270	3483467
3124	NearestLo	Chr02	3668999	3669882	884	+	1319	3665608
3124	NearestLo	Chr02	3665049	3665349	301	+	1223	3665608
50	NearestLo	Chr02	3751152	3751535	384	+	1044	3748149
45	NearestLo	Chr02	3782476	3782885	410	+	1035	3782649
271	NearestLo	Chr02	3848739	3849698	960	+	1027	3848631
2007	NearestLo	Chr02	4994342	4994824	483	+	1117	4989306
671	NearestLo	Chr02	5524389	5524655	267	+	1046	5521471
490	NearestLo	Chr02	5973132	5973334	203	+	1413	5971700
2246	NearestLo	Chr02	6140044	6140297	254	+	1066	6134192
431	NearestLo	Chr02	6760357	6760655	299	+	1160	6758985
431	NearestLo	Chr02	6755450	6755721	272	+	1107	6758985
431	NearestLo	Chr02	6757232	6757511	280	+	1532	6758985
4408	NearestLo	Chr02	6811237	6811502	266	+	1560	6809739
3083	NearestLo	Chr02	6816832	6817114	283	+	1254	6817567
3083	NearestLo	Chr02	6817662	6817914	253	+	1417	6817567
3418	NearestLo	Chr02	7002628	7002916	289	+	1351	6997442
175	NearestLo	Chr02	7141027	7141507	481	+	1040	7140938
7008	NearestLo	Chr02	7840144	7840442	299	+	1147	7829783
7008	NearestLo	Chr02	7840877	7841152	276	+	1334	7829783
246	NearestLo	Chr02	8024799	8025148	350	+	1021	8024667
530	NearestLo	Chr02	8319015	8319222	208	+	1580	8322992
530	NearestLo	Chr02	8323657	8324384	728	+	1213	8322992
181	NearestLo	Chr02	9177128	9177579	452	+	1345	9176812
181	NearestLo	Chr02	9169823	9170081	259	+	1227	9176812
4182	NearestLo	Chr02	10237846	10238244	399	+	1096	10233626
1019	NearestLo	Chr02	10425581	10425893	313	+	1218	10423223
56	NearestLo	Chr02	10485109	10485433	325	+	1332	10483728
2910	NearestLo	Chr02	11793702	11794306	605	+	1457	11792441
702	NearestLo	Chr02	12458296	12458518	223	+	1157	12456399
3906	NearestLo	Chr02	13295121	13295877	757	+	1335	13296746
119	NearestLo	Chr02	13618950	13619349	400	+	1342	13618930
739	NearestLo	Chr02	14003263	14003493	231	+	1458	14004400
31	NearestLo	Chr02	14263551	14263826	276	+	1055	14263537
4081	NearestLo	Chr02	15618532	15618841	310	+	1026	15622968
1093	NearestLo	Chr02	15797941	15798199	259	+	1619	15787688
105	NearestLo	Chr02	16767147	16767539	393	+	1148	16766346
1705	NearestLo	Chr02	16836819	16837102	284	+	1195	16834566
651	NearestLo	Chr02	17125535	17125752	218	+	1290	17124896
4780	NearestLo	Chr02	17657486	17657737	252	+	1120	17656193
4780	NearestLo	Chr02	17652902	17653160	259	+	1031	17656193
4780	NearestLo	Chr02	17648928	17649163	236	+	1100	17656193
4780	NearestLo	Chr02	17654370	17654587	218	+	1208	17656193
825	NearestLo	Chr02	17657486	17657737	252	+	1120	17656193
825	NearestLo	Chr02	17652902	17653160	259	+	1031	17656193
825	NearestLo	Chr02	17648928	17649163	236	+	1100	17656193
825	NearestLo	Chr02	17654370	17654587	218	+	1208	17656193
3027	NearestLo	Chr02	17657486	17657737	252	+	1120	17656193
3027	NearestLo	Chr02	17652902	17653160	259	+	1031	17656193

3027	NearestLoChr02	17648928	17649163	236	+	1100	17656193
3027	NearestLoChr02	17654370	17654587	218	+	1208	17656193
3980	NearestLoChr02	17657486	17657737	252	+	1120	17656193
3980	NearestLoChr02	17652902	17653160	259	+	1031	17656193
3980	NearestLoChr02	17648928	17649163	236	+	1100	17656193
3980	NearestLoChr02	17654370	17654587	218	+	1208	17656193
11093	NearestLoChr02	17813769	17813987	219	+	1167	17802211
11	NearestLoChr02	18302829	18303058	230	+	1121	18302824
1638	NearestLoChr02	18355444	18356469	1026	+	1052	18358100
1638	NearestLoChr02	18351044	18351388	345	+	1039	18358100
1638	NearestLoChr02	18356795	18357601	807	+	1070	18358100
1638	NearestLoChr02	18358916	18359235	320	+	1156	18358100
1638	NearestLoChr02	18354892	18355272	381	+	1247	18358100
1638	NearestLoChr02	18357950	18358670	721	+	1187	18358100
1638	NearestLoChr02	18353027	18353833	807	+	1089	18358100
1638	NearestLoChr02	18354058	18354647	590	+	1067	18358100
1638	NearestLoChr02	18351538	18352808	1271	+	1151	18358100
1638	NearestLoChr02	18348928	18349979	1052	+	1099	18358100
4278	NearestLoChr02	18355444	18356469	1026	+	1052	18358100
4278	NearestLoChr02	18351044	18351388	345	+	1039	18358100
4278	NearestLoChr02	18356795	18357601	807	+	1070	18358100
4278	NearestLoChr02	18358916	18359235	320	+	1156	18358100
4278	NearestLoChr02	18354892	18355272	381	+	1247	18358100
4278	NearestLoChr02	18357950	18358670	721	+	1187	18358100
4278	NearestLoChr02	18353027	18353833	807	+	1089	18358100
4278	NearestLoChr02	18354058	18354647	590	+	1067	18358100
4278	NearestLoChr02	18351538	18352808	1271	+	1151	18358100
4278	NearestLoChr02	18348928	18349979	1052	+	1099	18358100
132	NearestLoChr02	18355444	18356469	1026	+	1052	18358100
132	NearestLoChr02	18351044	18351388	345	+	1039	18358100
132	NearestLoChr02	18356795	18357601	807	+	1070	18358100
132	NearestLoChr02	18358916	18359235	320	+	1156	18358100
132	NearestLoChr02	18354892	18355272	381	+	1247	18358100
132	NearestLoChr02	18357950	18358670	721	+	1187	18358100
132	NearestLoChr02	18353027	18353833	807	+	1089	18358100
132	NearestLoChr02	18354058	18354647	590	+	1067	18358100
132	NearestLoChr02	18351538	18352808	1271	+	1151	18358100
132	NearestLoChr02	18348928	18349979	1052	+	1099	18358100
6750	NearestLoChr02	18355444	18356469	1026	+	1052	18358100
6750	NearestLoChr02	18351044	18351388	345	+	1039	18358100
6750	NearestLoChr02	18356795	18357601	807	+	1070	18358100
6750	NearestLoChr02	18358916	18359235	320	+	1156	18358100
6750	NearestLoChr02	18354892	18355272	381	+	1247	18358100
6750	NearestLoChr02	18357950	18358670	721	+	1187	18358100
6750	NearestLoChr02	18353027	18353833	807	+	1089	18358100
6750	NearestLoChr02	18354058	18354647	590	+	1067	18358100
6750	NearestLoChr02	18351538	18352808	1271	+	1151	18358100
6750	NearestLoChr02	18348928	18349979	1052	+	1099	18358100
554	NearestLoChr02	18355444	18356469	1026	+	1052	18358100
554	NearestLoChr02	18351044	18351388	345	+	1039	18358100
554	NearestLoChr02	18356795	18357601	807	+	1070	18358100
554	NearestLoChr02	18358916	18359235	320	+	1156	18358100
554	NearestLoChr02	18354892	18355272	381	+	1247	18358100
554	NearestLoChr02	18357950	18358670	721	+	1187	18358100

554	NearestLo	Chr02	18353027	18353833	807	+	1089	18358100
554	NearestLo	Chr02	18354058	18354647	590	+	1067	18358100
554	NearestLo	Chr02	18351538	18352808	1271	+	1151	18358100
554	NearestLo	Chr02	18348928	18349979	1052	+	1099	18358100
8110	NearestLo	Chr02	18355444	18356469	1026	+	1052	18358100
8110	NearestLo	Chr02	18351044	18351388	345	+	1039	18358100
8110	NearestLo	Chr02	18356795	18357601	807	+	1070	18358100
8110	NearestLo	Chr02	18358916	18359235	320	+	1156	18358100
8110	NearestLo	Chr02	18354892	18355272	381	+	1247	18358100
8110	NearestLo	Chr02	18357950	18358670	721	+	1187	18358100
8110	NearestLo	Chr02	18353027	18353833	807	+	1089	18358100
8110	NearestLo	Chr02	18354058	18354647	590	+	1067	18358100
8110	NearestLo	Chr02	18351538	18352808	1271	+	1151	18358100
8110	NearestLo	Chr02	18348928	18349979	1052	+	1099	18358100
3469	NearestLo	Chr02	18355444	18356469	1026	+	1052	18358100
3469	NearestLo	Chr02	18351044	18351388	345	+	1039	18358100
3469	NearestLo	Chr02	18356795	18357601	807	+	1070	18358100
3469	NearestLo	Chr02	18358916	18359235	320	+	1156	18358100
3469	NearestLo	Chr02	18354892	18355272	381	+	1247	18358100
3469	NearestLo	Chr02	18357950	18358670	721	+	1187	18358100
3469	NearestLo	Chr02	18353027	18353833	807	+	1089	18358100
3469	NearestLo	Chr02	18354058	18354647	590	+	1067	18358100
3469	NearestLo	Chr02	18351538	18352808	1271	+	1151	18358100
3469	NearestLo	Chr02	18348928	18349979	1052	+	1099	18358100
715	NearestLo	Chr02	18361708	18364064	2357	+	1102	18361982
428	NearestLo	Chr02	18365205	18368268	3064	+	1077	18367804
428	NearestLo	Chr02	18368431	18368689	259	+	1197	18367804
134	NearestLo	Chr02	18365205	18368268	3064	+	1077	18367804
134	NearestLo	Chr02	18368431	18368689	259	+	1197	18367804
486	NearestLo	Chr02	18377627	18381039	3413	+	1093	18377658
486	NearestLo	Chr02	18382101	18383266	1166	+	1090	18377658
3	NearestLo	Chr02	18377627	18381039	3413	+	1093	18377658
3	NearestLo	Chr02	18382101	18383266	1166	+	1090	18377658
291	NearestLo	Chr02	18385275	18386331	1057	+	1080	18387444
291	NearestLo	Chr02	18386632	18388410	1779	+	1069	18387444
291	NearestLo	Chr02	18384360	18385054	695	+	1188	18387444
66	NearestLo	Chr02	18388683	18389323	641	+	1135	18388647
66	NearestLo	Chr02	18389494	18389743	250	+	1180	18388647
10933	NearestLo	Chr02	19550571	19550789	219	+	1730	19561742
102	NearestLo	Chr02	20474380	20474597	218	+	1559	20471388
5727	NearestLo	Chr02	20569249	20569480	232	+	1115	20561725
5727	NearestLo	Chr02	20575419	20575621	203	+	1543	20561725
330	NearestLo	Chr02	21175537	21175984	448	+	1462	21167613
1411	NearestLo	Chr02	21546341	21546642	302	+	1640	21548048
1	NearestLo	Chr02	21820213	21820933	721	+	1174	21819882
6817	NearestLo	Chr02	21925256	21925489	234	+	1526	21932798
1	NearestLo	Chr02	22768032	22768300	269	+	1137	22760605
646	NearestLo	Chr02	23765393	23765639	247	+	1565	23765393
646	NearestLo	Chr02	23766134	23766416	283	+	1591	23765393
59	NearestLo	Chr02	23906096	23906524	429	+	1017	23903060
59	NearestLo	Chr02	23898092	23898409	318	+	1398	23903060
59	NearestLo	Chr02	23902234	23902461	228	+	1670	23903060
59	NearestLo	Chr02	23916162	23916363	202	+	1685	23903060
1085	NearestLo	Chr02	24751777	24752108	332	+	1416	24750711

33	NearestLo	Chr02	25139994	25140322	329	+	1159	25138768
33	NearestLo	Chr02	25141444	25141808	365	+	1659	25138768
5559	NearestLo	Chr03	63366	63586	221	+	2277	70322
5559	NearestLo	Chr03	61960	62217	258	+	1929	70322
6915	NearestLo	Chr03	63366	63586	221	+	2277	70322
6915	NearestLo	Chr03	61960	62217	258	+	1929	70322
690	NearestLo	Chr03	656107	656313	207	+	1971	655216
42	NearestLo	Chr03	2551836	2552237	402	+	1810	2550013
42	NearestLo	Chr03	2550883	2551215	333	+	1906	2550013
112	NearestLo	Chr03	3022241	3022443	203	+	2169	3022134
13	NearestLo	Chr03	3376749	3376995	247	+	2198	3374216
1359	NearestLo	Chr03	3376749	3376995	247	+	2198	3374216
5016	NearestLo	Chr03	4750342	4750798	457	+	2190	4749885
5016	NearestLo	Chr03	4755675	4755888	214	+	1895	4749885
3603	NearestLo	Chr03	6446737	6446943	207	+	2302	6415677
52	NearestLo	Chr03	9386689	9387104	416	+	1762	9386830
52	NearestLo	Chr03	9386035	9386357	323	+	1747	9386830
52	NearestLo	Chr03	9387415	9387995	581	+	1758	9386830
489	NearestLo	Chr03	9386689	9387104	416	+	1762	9386830
489	NearestLo	Chr03	9386035	9386357	323	+	1747	9386830
489	NearestLo	Chr03	9387415	9387995	581	+	1758	9386830
593	NearestLo	Chr03	9386689	9387104	416	+	1762	9386830
593	NearestLo	Chr03	9386035	9386357	323	+	1747	9386830
593	NearestLo	Chr03	9387415	9387995	581	+	1758	9386830
680	NearestLo	Chr03	9389145	9389560	416	+	1779	9389396
680	NearestLo	Chr03	9388507	9388737	231	+	1785	9389396
146	NearestLo	Chr03	9389145	9389560	416	+	1779	9389396
146	NearestLo	Chr03	9388507	9388737	231	+	1785	9389396
147	NearestLo	Chr03	9567952	9568765	814	+	1792	9567815
319	NearestLo	Chr03	9567952	9568765	814	+	1792	9567815
188	NearestLo	Chr03	9567952	9568765	814	+	1792	9567815
807	NearestLo	Chr03	10182068	10182377	310	+	1773	10183128
723	NearestLo	Chr03	10536346	10536550	205	+	2239	10534418
4847	NearestLo	Chr03	12730302	12730611	310	+	1994	12729225
4847	NearestLo	Chr03	12729315	12729763	449	+	2039	12729225
2338	NearestLo	Chr03	13046255	13046503	249	+	2085	13041081
2338	NearestLo	Chr03	13050558	13050821	264	+	2207	13041081
182	NearestLo	Chr03	13055166	13055395	230	+	1976	13049026
896	NearestLo	Chr03	13103233	13103563	331	+	1960	13105462
1597	NearestLo	Chr03	13156669	13156917	249	+	1992	13156603
367	NearestLo	Chr03	13183197	13183981	785	+	1744	13183164
1882	NearestLo	Chr03	13697429	13697781	353	+	1753	13699658
4134	NearestLo	Chr03	14060441	14060698	258	+	1905	14047734
3067	NearestLo	Chr03	15330206	15330637	432	+	1924	15325012
118	NearestLo	Chr03	15380503	15381788	1286	+	1748	15379900
1560	NearestLo	Chr03	15553774	15554026	253	+	1813	15551696
2306	NearestLo	Chr03	15708796	15709082	287	+	1867	15709696
853	NearestLo	Chr03	15837711	15837925	215	+	1828	15836051
7653	NearestLo	Chr03	16373534	16373837	304	+	2055	16372582
7653	NearestLo	Chr03	16374816	16375066	251	+	1932	16372582
6	NearestLo	Chr03	16625718	16625969	252	+	2092	16625655
1039	NearestLo	Chr03	16699812	16700045	234	+	1823	16701322
6473	NearestLo	Chr03	17009754	17010243	490	+	2299	17002606
1027	NearestLo	Chr03	17594844	17595282	439	+	1928	17596241

1027	NearestLo	Chr03	17596471	17596730	260	+	2091	17596241
5722	NearestLo	Chr03	17954376	17954598	223	+	2183	17950578
5722	NearestLo	Chr03	17956701	17956968	268	+	1880	17950578
4857	NearestLo	Chr03	18363163	18363377	215	+	2300	18368222
550	NearestLo	Chr03	18772419	18772641	223	+	2089	18770564
581	NearestLo	Chr03	19533934	19534457	524	+	1769	19531706
103	NearestLo	Chr03	20191594	20191833	240	+	2033	20187285
103	NearestLo	Chr03	20194416	20194873	458	+	2304	20187285
3013	NearestLo	Chr03	20293299	20293500	202	+	2275	20294387
3013	NearestLo	Chr03	20297536	20297743	208	+	1829	20294387
2893	NearestLo	Chr03	20350200	20350420	221	+	2217	20353339
2893	NearestLo	Chr03	20353179	20353712	534	+	2199	20353339
138	NearestLo	Chr03	20447594	20448234	641	+	1756	20447584
7693	NearestLo	Chr03	20661193	20661425	233	+	1972	20665302
7693	NearestLo	Chr03	20665290	20665988	699	+	1990	20665302
7693	NearestLo	Chr03	20666230	20666769	540	+	1830	20665302
1562	NearestLo	Chr03	21512367	21512597	231	+	1968	21514119
105	NearestLo	Chr03	21526764	21527319	556	+	1903	21526643
1554	NearestLo	Chr04	811217	811498	282	+	2609	808810
59	NearestLo	Chr04	1297150	1297608	459	+	2476	1296320
3301	NearestLo	Chr04	2805131	2805420	290	+	2734	2795361
3301	NearestLo	Chr04	2803066	2803311	246	+	2566	2795361
2830	NearestLo	Chr04	3420780	3421004	225	+	2431	3417387
7592	NearestLo	Chr04	3792167	3792468	302	+	2598	3793576
668	NearestLo	Chr04	3869410	3869623	214	+	2389	3868748
2509	NearestLo	Chr04	4187105	4187340	236	+	2665	4187175
4190	NearestLo	Chr04	5246525	5246832	308	+	2766	5238758
277	NearestLo	Chr04	6269728	6269974	247	+	2485	6269490
147	NearestLo	Chr04	6778390	6778728	339	+	2371	6778244
228	NearestLo	Chr04	8206524	8206891	368	+	2728	8206433
199	NearestLo	Chr04	8369163	8369385	223	+	2680	8367867
199	NearestLo	Chr04	8367991	8368788	798	+	2386	8367867
132	NearestLo	Chr04	8369163	8369385	223	+	2680	8367867
132	NearestLo	Chr04	8367991	8368788	798	+	2386	8367867
15387	NearestLo	Chr04	9503383	9504015	633	+	2363	9511345
7341	NearestLo	Chr04	9503383	9504015	633	+	2363	9511345
1140	NearestLo	Chr04	10315231	10315486	256	+	2347	10314112
2539	NearestLo	Chr04	11931886	11932194	309	+	2445	11929544
4135	NearestLo	Chr04	12421490	12422088	599	+	2449	12466740
4135	NearestLo	Chr04	12472409	12472862	454	+	2356	12466740
21073	NearestLo	Chr04	12669400	12669633	234	+	2625	12590366
75367	NearestLo	Chr04	13200687	13201112	426	+	2329	13124991
6832	NearestLo	Chr04	14359017	14359433	417	+	2377	14349119
6832	NearestLo	Chr04	14342520	14342721	202	+	2727	14349119
6832	NearestLo	Chr04	14340423	14340667	245	+	2475	14349119
6832	NearestLo	Chr04	14367586	14367832	247	+	2610	14349119
6832	NearestLo	Chr04	14363542	14363757	216	+	2439	14349119
6832	NearestLo	Chr04	14358086	14358387	302	+	2496	14349119
41388	NearestLo	Chr04	14458085	14458313	229	+	2361	14414010
3311	NearestLo	Chr04	14909202	14909516	315	+	2731	14903197
1896	NearestLo	Chr04	16138721	16138963	243	+	2396	16134853
1896	NearestLo	Chr04	16148251	16148461	211	+	2532	16134853
75	NearestLo	Chr04	16761854	16762152	299	+	2405	16762048
75	NearestLo	Chr04	16762853	16763508	656	+	2646	16762048

173	NearestLo	Chr04	17750780	17750991	212	+	2593	17748285
8492	NearestLo	Chr04	18158896	18159128	233	+	2545	18167081
6626	NearestLo	Chr04	18519405	18519887	483	+	2426	18519395
2874	NearestLo	Chr04	18519405	18519887	483	+	2426	18519395
4152	NearestLo	Chr04	18677749	18678375	627	+	2408	18672434
3090	NearestLo	Chr04	18677749	18678375	627	+	2408	18672434
649	NearestLo	Chr04	19046633	19047047	415	+	2322	19044352
599	NearestLo	Chr04	19654537	19654780	244	+	2626	19646094
599	NearestLo	Chr04	19650431	19650635	205	+	2631	19646094
599	NearestLo	Chr04	19643502	19643833	332	+	2553	19646094
152	NearestLo	Chr04	20206262	20206920	659	+	2320	20203128
523	NearestLo	Chr04	20356481	20356750	270	+	2410	20354217
516	NearestLo	Chr04	20859141	20859422	282	+	2564	20855645
67	NearestLo	Chr04	20887712	20888041	330	+	2334	20885873
1657	NearestLo	Chr04	21400354	21400726	373	+	2655	21396989
543	NearestLo	Chr04	22883525	22884071	547	+	2324	22881130
1527	NearestLo	Chr04	24170682	24171278	597	+	2341	24164589
304	NearestLo	Chr05	350371	350605	235	+	3206	347790
86	NearestLo	Chr05	1855190	1855469	280	+	2828	1854298
86	NearestLo	Chr05	1854191	1854433	243	+	2864	1854298
86	NearestLo	Chr05	1855778	1856252	475	+	2974	1854298
217	NearestLo	Chr05	1879751	1880217	467	+	2783	1876628
1834	NearestLo	Chr05	4183994	4184430	437	+	3069	4181343
911	NearestLo	Chr05	4183994	4184430	437	+	3069	4181343
733	NearestLo	Chr05	4534341	4534587	247	+	3125	4534405
2841	NearestLo	Chr05	5598678	5598907	230	+	3089	5591213
15552	NearestLo	Chr05	5707538	5707816	279	+	2867	5704544
249	NearestLo	Chr05	6349840	6350345	506	+	2879	6349758
1765	NearestLo	Chr05	6478499	6478800	302	+	3276	6473959
1277	NearestLo	Chr05	6900130	6900465	336	+	2792	6898522
4519	NearestLo	Chr05	7165558	7165799	242	+	3135	7170333
4943	NearestLo	Chr05	7393713	7393961	249	+	3096	7383725
3019	NearestLo	Chr05	8402073	8402842	770	+	3136	8401879
6351	NearestLo	Chr05	11618888	11619107	220	+	3127	11614109
38	NearestLo	Chr05	11709051	11709431	381	+	2777	11705721
8378	NearestLo	Chr05	12269447	12270175	729	+	3063	12269399
13367	NearestLo	Chr05	12743922	12744163	242	+	2817	12725044
13367	NearestLo	Chr05	12729522	12729739	218	+	3260	12725044
18101	NearestLo	Chr05	12771935	12772425	491	+	2794	12771010
56	NearestLo	Chr05	13493406	13493640	235	+	2810	13493293
8663	NearestLo	Chr05	13507265	13507466	202	+	3240	13493828
8663	NearestLo	Chr05	13510887	13511110	224	+	2927	13493828
8663	NearestLo	Chr05	13510154	13510359	206	+	3182	13493828
8663	NearestLo	Chr05	13504514	13504939	426	+	2954	13493828
14854	NearestLo	Chr05	13507265	13507466	202	+	3240	13493828
14854	NearestLo	Chr05	13510887	13511110	224	+	2927	13493828
14854	NearestLo	Chr05	13510154	13510359	206	+	3182	13493828
14854	NearestLo	Chr05	13504514	13504939	426	+	2954	13493828
14100	NearestLo	Chr05	13507265	13507466	202	+	3240	13493828
14100	NearestLo	Chr05	13510887	13511110	224	+	2927	13493828
14100	NearestLo	Chr05	13510154	13510359	206	+	3182	13493828
14100	NearestLo	Chr05	13504514	13504939	426	+	2954	13493828
1031	NearestLo	Chr05	13547004	13547557	554	+	3257	13548653
1031	NearestLo	Chr05	13549114	13549343	230	+	3262	13548653

7786	NearestLoChr05	14524542	14524743	202	+	3033	14532529
132	NearestLoChr05	14814265	14814555	291	+	2786	14813787
15	NearestLoChr05	14916520	14916728	209	+	3029	14824857
15	NearestLoChr05	14825929	14826133	205	+	2911	14824857
9130	NearestLoChr05	16498362	16498690	329	+	2805	16508873
2480	NearestLoChr05	17723981	17724206	226	+	3205	17714154
2480	NearestLoChr05	17718189	17718418	230	+	2821	17714154
646	NearestLoChr05	18577550	18578487	938	+	2796	18573573
1	NearestLoChr05	19147023	19147438	416	+	2788	19147421
2375	NearestLoChr05	19329831	19330127	297	+	3143	19329742
3399	NearestLoChr05	19626288	19626634	347	+	2802	19612098
4312	NearestLoChr05	20544888	20545238	351	+	2822	20549534
1184	NearestLoChr05	21437904	21438458	555	+	2837	21433980
827	NearestLoChr05	21883716	21883991	276	+	2813	21881226
245	NearestLoChr05	21982056	21982987	932	+	2780	21981629
315	NearestLoChr05	22394592	22395722	1131	+	2779	22394468
315	NearestLoChr05	22396237	22396492	256	+	2991	22394468
38	NearestLoChr05	22985989	22986462	474	+	2842	22985956
506	NearestLoChr05	23031314	23031805	492	+	2776	23030899
3640	NearestLoChr05	23575980	23576639	660	+	2897	23570628
1617	NearestLoChr05	23612422	23612652	231	+	3050	23600307
1617	NearestLoChr05	23602874	23603123	250	+	3112	23600307
51	NearestLoChr05	23664824	23665204	381	+	2836	23664968
1272	NearestLoChr05	24086408	24086690	283	+	3042	24079692
1272	NearestLoChr05	24083622	24083942	321	+	2873	24079692
655	NearestLoChr05	24202532	24202964	433	+	3233	24202979
655	NearestLoChr05	24205985	24206264	280	+	2900	24202979
206	NearestLoChr05	24285249	24285614	366	+	3011	24283167
474	NearestLoChr05	25231111	25231484	374	+	2815	25224831
474	NearestLoChr05	25235564	25235806	243	+	3265	25224831
2897	NearestLoChr06	584244	584458	215	+	3837	587389
867	NearestLoChr06	2704663	2704895	233	+	3431	2689574
118	NearestLoChr06	3653785	3654016	232	+	3689	3652919
118	NearestLoChr06	3652119	3652415	297	+	3485	3652919
663	NearestLoChr06	3655635	3655853	219	+	3545	3655561
163	NearestLoChr06	3673011	3673404	394	+	3382	3672303
1110	NearestLoChr06	4148608	4149172	565	+	3950	4148440
1110	NearestLoChr06	4152245	4152606	362	+	3528	4148440
427	NearestLoChr06	4614546	4614888	343	+	3970	4611139
427	NearestLoChr06	4611581	4611895	315	+	3423	4611139
427	NearestLoChr06	4611221	4611423	203	+	3741	4611139
2906	NearestLoChr06	4993411	4993640	230	+	3767	4996545
1849	NearestLoChr06	5400773	5400978	206	+	3836	5396914
2034	NearestLoChr06	5454022	5454604	583	+	3541	5453655
385	NearestLoChr06	6509794	6509996	203	+	4013	6510362
4611	NearestLoChr06	6522707	6523053	347	+	3367	6517063
105	NearestLoChr06	6550277	6550505	229	+	3639	6538210
105	NearestLoChr06	6543144	6543671	528	+	3841	6538210
5079	NearestLoChr06	7753995	7754213	219	+	3790	7746324
5079	NearestLoChr06	7748208	7748668	461	+	3972	7746324
2274	NearestLoChr06	8705787	8706060	274	+	3694	8701663
2274	NearestLoChr06	8702421	8703085	665	+	3776	8701663
2274	NearestLoChr06	8701804	8702040	237	+	3858	8701663
1601	NearestLoChr06	9338474	9338823	350	+	3943	9336398

2443	NearestLo	Chr06	9441753	9442017	265	+	3686	9445700
3635	NearestLo	Chr06	9461522	9461804	283	+	3460	9461477
86	NearestLo	Chr06	10468466	10468869	404	+	3455	10470188
9118	NearestLo	Chr06	11142177	11142427	251	+	3397	11151518
4265	NearestLo	Chr06	12135935	12136298	364	+	3661	12135840
335	NearestLo	Chr06	12899536	12899809	274	+	3838	12897663
335	NearestLo	Chr06	12873224	12873479	256	+	4009	12897663
335	NearestLo	Chr06	12897756	12898012	257	+	3526	12897663
825	NearestLo	Chr06	12957834	12958073	240	+	3478	12953296
801	NearestLo	Chr06	15216299	15216741	443	+	3699	15215286
28172	NearestLo	Chr06	15278450	15278661	212	+	3470	15248067
7934	NearestLo	Chr06	16338447	16338898	452	+	3430	16330173
5202	NearestLo	Chr06	16559820	16560218	399	+	3899	16554908
22385	NearestLo	Chr06	17649863	17650082	220	+	3536	17626307
726	NearestLo	Chr06	18632736	18632968	233	+	3395	18634640
13183	NearestLo	Chr06	19018887	19019090	204	+	3896	19005679
13183	NearestLo	Chr06	19020960	19021163	204	+	3980	19005679
13183	NearestLo	Chr06	19020120	19020421	302	+	3357	19005679
174	NearestLo	Chr06	19392868	19393162	295	+	3897	19392135
21584	NearestLo	Chr06	20042083	20042330	248	+	3702	20063874
21584	NearestLo	Chr06	20065027	20065385	359	+	3518	20063874
5392	NearestLo	Chr06	20911621	20912121	501	+	3552	20907598
5392	NearestLo	Chr06	20917499	20917821	323	+	3771	20907598
78	NearestLo	Chr06	21209579	21210104	526	+	3363	21209507
3534	NearestLo	Chr06	21740866	21741160	295	+	3652	21744980
11968	NearestLo	Chr06	22052393	22052615	223	+	3797	22064648
11968	NearestLo	Chr06	22053482	22053793	312	+	3938	22064648
11968	NearestLo	Chr06	22064469	22065172	704	+	3712	22064648
440	NearestLo	Chr06	22659175	22660194	1020	+	3368	22659114
12129	NearestLo	Chr06	23738887	23739256	370	+	3479	23751412
3564	NearestLo	Chr06	24408868	24409127	260	+	3789	24412663
4224	NearestLo	Chr06	24671942	24672329	388	+	3505	24667224
4224	NearestLo	Chr06	24680390	24680646	257	+	3643	24667224
4224	NearestLo	Chr06	24679741	24679994	254	+	3991	24667224
2670	NearestLo	Chr06	26743818	26744040	223	+	3669	26745119
2142	NearestLo	Chr07	1006397	1006681	285	+	4160	1002705
3258	NearestLo	Chr07	1441636	1442114	479	+	4123	1442351
3258	NearestLo	Chr07	1445931	1446154	224	+	4308	1442351
51	NearestLo	Chr07	3252317	3252672	356	+	4070	3252283
7985	NearestLo	Chr07	3578358	3578572	215	+	4239	3565933
401	NearestLo	Chr07	3779340	3779826	487	+	4043	3778290
10203	NearestLo	Chr07	4771090	4771576	487	+	4060	4759259
10203	NearestLo	Chr07	4760747	4761012	266	+	4332	4759259
11613	NearestLo	Chr07	5102743	5102973	231	+	4154	5114576
13196	NearestLo	Chr07	5675905	5676149	245	+	4161	5661027
4116	NearestLo	Chr07	5978374	5979018	645	+	4090	5978584
3274	NearestLo	Chr07	7247418	7247634	217	+	4104	7250907
3274	NearestLo	Chr07	7244873	7245089	217	+	4146	7250907
5824	NearestLo	Chr07	7247418	7247634	217	+	4104	7250907
5824	NearestLo	Chr07	7244873	7245089	217	+	4146	7250907
3687	NearestLo	Chr07	7978968	7979174	207	+	4147	7974628
2634	NearestLo	Chr07	8132118	8132588	471	+	4205	8144143
708	NearestLo	Chr07	8208994	8209534	541	+	4044	8206533
2777	NearestLo	Chr07	8780661	8780875	215	+	4316	8776939

2777	NearestLo	Chr07	8773936	8774150	215	+	4352	8776939
1071	NearestLo	Chr07	10548555	10548811	257	+	4183	10542508
24	NearestLo	Chr07	11193624	11194038	415	+	4144	11193574
1694	NearestLo	Chr07	11690608	11691111	504	+	4110	11686122
2883	NearestLo	Chr07	12681054	12681631	578	+	4047	12677944
316	NearestLo	Chr07	13002314	13003401	1088	+	4069	13002911
1357	NearestLo	Chr07	13312533	13312937	405	+	4086	13309003
186	NearestLo	Chr07	14210622	14210839	218	+	4143	14208713
74	NearestLo	Chr07	14911349	14911588	240	+	4219	14911273
3847	NearestLo	Chr07	15318105	15318350	246	+	4197	15314118
3964	NearestLo	Chr07	15318105	15318350	246	+	4197	15314118
404	NearestLo	Chr07	15362504	15362983	480	+	4300	15358332
65	NearestLo	Chr08	380053	380355	303	+	4441	379940
826	NearestLo	Chr08	1517013	1517307	295	+	4403	1518098
3094	NearestLo	Chr08	1778725	1779095	371	+	4442	1782197
3094	NearestLo	Chr08	1786018	1786233	216	+	4841	1782197
1195	NearestLo	Chr08	1991117	1991351	235	+	4799	1985311
2098	NearestLo	Chr08	2814992	2815235	244	+	4448	2811985
368	NearestLo	Chr08	3567344	3567575	232	+	4887	3564751
1244	NearestLo	Chr08	3614354	3614673	320	+	4763	3612661
1244	NearestLo	Chr08	3616048	3616278	231	+	4455	3612661
33	NearestLo	Chr08	3695591	3695798	208	+	4536	3695827
2300	NearestLo	Chr08	3780020	3780402	383	+	4390	3776560
2300	NearestLo	Chr08	3781559	3781865	307	+	4399	3776560
44	NearestLo	Chr08	4477035	4477500	466	+	4413	4476846
1988	NearestLo	Chr08	4508162	4508392	231	+	4688	4513635
1528	NearestLo	Chr08	5214458	5214724	267	+	4595	5212511
5017	NearestLo	Chr08	6064381	6064677	297	+	4741	6064176
5017	NearestLo	Chr08	6065456	6065748	293	+	4534	6064176
1705	NearestLo	Chr08	6163029	6163379	351	+	4422	6162187
1705	NearestLo	Chr08	6162300	6162535	236	+	4672	6162187
12	NearestLo	Chr08	6627619	6627837	219	+	4826	6626146
12	NearestLo	Chr08	6628242	6628492	251	+	4923	6626146
96	NearestLo	Chr08	7844950	7845183	234	+	4700	7843368
66	NearestLo	Chr08	8011358	8011579	222	+	4610	8011656
66	NearestLo	Chr08	8012191	8012424	234	+	4704	8011656
6176	NearestLo	Chr08	8025385	8025603	219	+	4675	8017574
301	NearestLo	Chr08	8631363	8631704	342	+	4462	8631059
301	NearestLo	Chr08	8633376	8633604	229	+	4800	8631059
4437	NearestLo	Chr08	9866344	9866563	220	+	4833	9860607
4437	NearestLo	Chr08	9854600	9854894	295	+	4625	9860607
411	NearestLo	Chr08	11091539	11091784	246	+	4894	11091933
1294	NearestLo	Chr08	11910232	11910568	337	+	4393	11911838
4323	NearestLo	Chr08	12097658	12097908	251	+	4565	12097990
4323	NearestLo	Chr08	12093340	12093728	389	+	4445	12097990
84	NearestLo	Chr08	12401081	12401459	379	+	4394	12401027
1405	NearestLo	Chr08	13021053	13021338	286	+	4570	13019788
1387	NearestLo	Chr08	14569413	14569944	532	+	4634	14568575
1	NearestLo	Chr08	15126916	15127841	926	+	4391	15126261
9749	NearestLo	Chr08	15743233	15744060	828	+	4400	15742390
9749	NearestLo	Chr08	15732430	15732636	207	+	4731	15742390
639	NearestLo	Chr08	17235052	17235466	415	+	4820	17239823
639	NearestLo	Chr08	17238861	17239150	290	+	4792	17239823
4564	NearestLo	Chr08	17235052	17235466	415	+	4820	17239823

4564	NearestLo	Chr08	17238861	17239150	290	+	4792	17239823
251	NearestLo	Chr08	17483505	17484104	600	+	4408	17478246
10117	NearestLo	Chr08	18791610	18791833	224	+	4868	18790427
10117	NearestLo	Chr08	18779653	18780331	679	+	4417	18790427
2506	NearestLo	Chr09	188070	188281	212	+	5034	190781
10471	NearestLo	Chr09	250769	251112	344	+	4958	238870
12888	NearestLo	Chr09	414077	414299	223	+	4980	428906
14604	NearestLo	Chr09	414077	414299	223	+	4980	428906
1098	NearestLo	Chr09	988444	989002	559	+	5096	989221
1098	NearestLo	Chr09	987785	988134	350	+	4954	989221
1098	NearestLo	Chr09	989363	989660	298	+	4959	989221
1098	NearestLo	Chr09	990379	990611	233	+	4974	989221
20	NearestLo	Chr09	988444	989002	559	+	5096	989221
20	NearestLo	Chr09	987785	988134	350	+	4954	989221
20	NearestLo	Chr09	989363	989660	298	+	4959	989221
20	NearestLo	Chr09	990379	990611	233	+	4974	989221
81	NearestLo	Chr09	991153	991726	574	+	4973	991605
6547	NearestLo	Chr09	1366456	1366744	289	+	5315	1392220
6547	NearestLo	Chr09	1385394	1385634	241	+	4981	1392220
6547	NearestLo	Chr09	1393175	1393688	514	+	4995	1392220
14	NearestLo	Chr09	2728635	2728871	237	+	4951	2730090
14	NearestLo	Chr09	2727855	2728268	414	+	4952	2730090
14	NearestLo	Chr09	2727327	2727658	332	+	4948	2730090
14	NearestLo	Chr09	2730020	2730245	226	+	4967	2730090
490	NearestLo	Chr09	2728635	2728871	237	+	4951	2730090
490	NearestLo	Chr09	2727855	2728268	414	+	4952	2730090
490	NearestLo	Chr09	2727327	2727658	332	+	4948	2730090
490	NearestLo	Chr09	2730020	2730245	226	+	4967	2730090
635	NearestLo	Chr09	4118380	4118763	384	+	5029	4112691
446	NearestLo	Chr09	5380858	5381154	297	+	5016	5382460
1127	NearestLo	Chr09	5483028	5483563	536	+	4942	5478033
7381	NearestLo	Chr09	5498972	5499272	301	+	5051	5502775
30	NearestLo	Chr09	5853142	5853469	328	+	4975	5853151
1052	NearestLo	Chr09	6591556	6591931	376	+	5219	6590436
1083	NearestLo	Chr09	6860720	6861258	539	+	4936	6852192
90	NearestLo	Chr09	7143400	7143964	565	+	4947	7142744
162	NearestLo	Chr09	7525776	7525992	217	+	5126	7517263
3631	NearestLo	Chr09	8620807	8621341	535	+	4992	8625064
904	NearestLo	Chr09	10213825	10214058	234	+	5155	10202790
815	NearestLo	Chr09	10239956	10240183	228	+	5204	10238649
144	NearestLo	Chr09	10435139	10435390	252	+	5215	10435173
144	NearestLo	Chr09	10435866	10436236	371	+	5168	10435173
8	NearestLo	Chr09	11366516	11367072	557	+	4933	11362432
3566	NearestLo	Chr09	11403434	11403787	354	+	4955	11407300
4876	NearestLo	Chr09	11791593	11791808	216	+	5311	11782925
4876	NearestLo	Chr09	11786491	11786702	212	+	5240	11782925
242	NearestLo	Chr09	11983517	11983741	225	+	5071	11981991
1197	NearestLo	Chr09	12026936	12027416	481	+	4946	12025721
669	NearestLo	Chr09	12165018	12165472	455	+	4985	12157613
1185	NearestLo	Chr09	12948267	12948492	226	+	5030	12943204
341	NearestLo	Chr10	680609	680868	260	+	5512	680335
9086	NearestLo	Chr10	2849714	2849963	250	+	5682	2837752
51760	NearestLo	Chr10	4213197	4213412	216	+	5399	4155805
1960	NearestLo	Chr10	4426923	4427231	309	+	5507	4421703

77240	NearestLo	Chr10	4752261	4752476	216	+	5527	4663851
77240	NearestLo	Chr10	4670343	4670558	216	+	5940	4663851
20516	NearestLo	Chr10	4752261	4752476	216	+	5527	4663851
20516	NearestLo	Chr10	4670343	4670558	216	+	5940	4663851
4459	NearestLo	Chr10	4752261	4752476	216	+	5527	4663851
4459	NearestLo	Chr10	4670343	4670558	216	+	5940	4663851
47	NearestLo	Chr10	5229760	5230053	294	+	5373	5229730
52995	NearestLo	Chr10	5616023	5616277	255	+	5526	5669271
32	NearestLo	Chr10	5747989	5748460	472	+	5468	5747967
821	NearestLo	Chr10	7325223	7325792	570	+	5380	7324408
30	NearestLo	Chr10	8333068	8333736	669	+	5398	8333019
30	NearestLo	Chr10	8334196	8334467	272	+	5730	8333019
1373	NearestLo	Chr10	8335371	8335588	218	+	5450	8336247
1373	NearestLo	Chr10	8334675	8334899	225	+	5490	8336247
783	NearestLo	Chr10	10002448	10002941	494	+	5633	10002082
966	NearestLo	Chr10	10141967	10143198	1232	+	5513	10139477
1403	NearestLo	Chr10	11301599	11302404	806	+	5432	11303163
3233	NearestLo	Chr10	11758511	11758742	232	+	5811	11747167
793	NearestLo	Chr10	11849048	11849307	260	+	5661	11844006
293	NearestLo	Chr10	12506674	12507025	352	+	5402	12503053
293	NearestLo	Chr10	12503399	12503783	385	+	5382	12503053
6956	NearestLo	Chr10	13107615	13107847	233	+	5567	13117045
1433	NearestLo	Chr10	13134070	13134518	449	+	5651	13132809
1433	NearestLo	Chr10	13133016	13133738	723	+	5495	13132809
1375	NearestLo	Chr10	13192868	13193207	340	+	5377	13194842
4695	NearestLo	Chr10	13643936	13644152	217	+	5703	13640755
1110	NearestLo	Chr10	13736443	13736714	272	+	5848	13733411
888	NearestLo	Chr10	14800898	14801229	332	+	5591	14802073
291	NearestLo	Chr10	15939341	15939543	203	+	5912	15938397
161	NearestLo	Chr10	16024795	16025134	340	+	5559	16019864
1276	NearestLo	Chr10	17008270	17008650	381	+	5411	17010039
113	NearestLo	Chr10	17100307	17100657	351	+	5465	17098655
329	NearestLo	Chr10	18068981	18069365	385	+	5392	18066606
3652	NearestLo	Chr10	18454600	18455022	423	+	5458	18454527
6897	NearestLo	Chr10	18934830	18935271	442	+	5372	18925453
495	NearestLo	Chr10	19155651	19156167	517	+	5375	19155276
4938	NearestLo	Chr10	19411276	19411714	439	+	5443	19401066
3549	NearestLo	Chr10	19765285	19765586	302	+	5379	19761629
1197	NearestLo	Chr10	20360962	20361281	320	+	5564	20362434
1197	NearestLo	Chr10	20365302	20365545	244	+	5776	20362434
1197	NearestLo	Chr10	20363439	20363722	284	+	5508	20362434
1020	NearestLo	Chr10	20743986	20744410	425	+	5404	20747463
1020	NearestLo	Chr10	20755176	20755520	345	+	5429	20747463
146	NearestLo	Chr10	20849184	20849488	305	+	5405	20849527
5	NearestLo	Chr10	20955655	20955879	225	+	5583	20955472
5	NearestLo	Chr10	20960113	20960325	213	+	5889	20955472
922	NearestLo	Chr11	279812	280607	796	+	6176	278846
91	NearestLo	Chr11	1293669	1294232	564	+	5993	1288323
510	NearestLo	Chr11	6675903	6676285	383	+	6098	6674943
262	NearestLo	Chr11	7193808	7194679	872	+	5994	7193480
315	NearestLo	Chr11	7203912	7204142	231	+	6040	7203770
134	NearestLo	Chr11	7203912	7204142	231	+	6040	7203770
89	NearestLo	Chr11	7206726	7206942	217	+	6015	7206842
473	NearestLo	Chr11	7216266	7217206	941	+	6005	7215822

425	NearestLo	Chr11	7216266	7217206	941	+	6005	7215822
41	NearestLo	Chr11	7232098	7232313	216	+	6052	7230312
41	NearestLo	Chr11	7231581	7231791	211	+	6161	7230312
1380	NearestLo	Chr11	8004403	8004663	261	+	6127	8006034
6508	NearestLo	Chr11	8281439	8281745	307	+	5992	8288217
855	NearestLo	Chr11	8750202	8750427	226	+	6169	8738401
4612	NearestLo	Chr11	11651088	11651305	218	+	6199	11644134
2284	NearestLo	Chr11	11651088	11651305	218	+	6199	11644134
6	NearestLo	Chr11	12064023	12064251	229	+	6022	12063003
43	NearestLo	Chr11	12868147	12868388	242	+	6041	12867984
82	NearestLo	Chr11	13861278	13861488	211	+	6044	13861100
1569	NearestLo	Chr11	14618344	14618747	404	+	6014	14618169
86	NearestLo	Chr11	15719391	15720251	861	+	6129	15719348
930	NearestLo	Chr11	16055877	16056090	214	+	6030	16056930
1955	NearestLo	Chr11	16378024	16378379	356	+	6113	16378944
1058	NearestLo	Chr11	16785839	16786356	518	+	5999	16785690
9429	NearestLo	Chr11	17249423	17249657	235	+	6130	17259631
18	NearestLo	Chr11	18050046	18050784	739	+	6004	18049998
3985	NearestLo	Chr12	279479	279700	222	+	6439	274497
311	NearestLo	Chr12	878456	878668	213	+	6441	878985
652	NearestLo	Chr12	1700438	1701262	825	+	6237	1698979
3184	NearestLo	Chr12	1791509	1791811	303	+	6322	1790897
476	NearestLo	Chr12	1874034	1874274	241	+	6577	1875416
476	NearestLo	Chr12	1875754	1875955	202	+	6582	1875416
85	NearestLo	Chr12	2051080	2051799	720	+	6231	2051060
724	NearestLo	Chr12	3633241	3633502	262	+	6490	3629169
4000	NearestLo	Chr12	3758909	3759381	473	+	6592	3756506
4907	NearestLo	Chr12	3785396	3785607	212	+	6502	3780252
2602	NearestLo	Chr12	4991491	4992211	721	+	6522	4986584
3271	NearestLo	Chr12	5408755	5409131	377	+	6580	5408743
48288	NearestLo	Chr12	6951538	6951761	224	+	6586	6940150
48288	NearestLo	Chr12	6959856	6960364	509	+	6514	6940150
52991	NearestLo	Chr12	7487675	7487881	207	+	6420	7540873
9170	NearestLo	Chr12	8466149	8466461	313	+	6456	8466099
9170	NearestLo	Chr12	8467552	8467851	300	+	6273	8466099
9170	NearestLo	Chr12	8442781	8442989	209	+	6543	8466099
652	NearestLo	Chr12	9824632	9825157	526	+	6544	9823077
1987	NearestLo	Chr12	9830055	9830259	205	+	6467	9825259
4314	NearestLo	Chr12	9855293	9855723	431	+	6403	9855190
4768	NearestLo	Chr12	10427676	10428056	381	+	6324	10411438
4768	NearestLo	Chr12	10421084	10421371	288	+	6230	10411438
18968	NearestLo	Chr12	10777572	10777807	236	+	6258	10796766
4705	NearestLo	Chr12	11144407	11144644	238	+	6457	11137722
6810	NearestLo	Chr12	11347358	11347818	461	+	6581	11356226
41	NearestLo	Chr12	11684413	11684618	206	+	6389	11682812
681	NearestLo	Chr12	12014926	12015133	208	+	6475	12011800
354	NearestLo	Chr12	12312732	12313551	820	+	6235	12311205
2591	NearestLo	Chr12	12359926	12360189	264	+	6294	12355907
2591	NearestLo	Chr12	12357220	12357619	400	+	6417	12355907
1096	NearestLo	Chr12	12374693	12374934	242	+	6373	12371993
1096	NearestLo	Chr12	12374020	12374270	251	+	6562	12371993
1777	NearestLo	Chr12	12553783	12554251	469	+	6545	12553770
2764	NearestLo	Chr12	12872659	12872890	232	+	6268	12868554
675	NearestLo	Chr12	12893441	12893645	205	+	6599	12894761

173	NearestLo	Chr12	14120122	14120522	401	+	6546	14117032
2080	NearestLo	Chr12	14198830	14199040	211	+	6433	14196754
99	NearestLo	Chr12	14735756	14736070	315	+	6282	14735670
1505	NearestLo	Chr12	14781193	14781640	448	+	6600	14777432
110	NearestLo	Chr12	14964827	14965093	267	+	6563	14961839
1855	NearestLo	Chr12	14975290	14975491	202	+	6556	14970437
1397	NearestLo	Chr12	15168589	15168920	332	+	6287	15170317
61	NearestLo	Chr12	15239726	15239963	238	+	6429	15237670
578	NearestLo	Chr12	15380371	15380619	249	+	6512	15374256
169	NearestLo	Chr12	15582573	15583050	478	+	6290	15582384
1275	NearestLo	Chr12	15705385	15705676	292	+	6387	15704924
1142	NearestLo	Chr13	634016	634247	232	+	6791	634239
9698	NearestLo	Chr13	786204	786556	353	+	6684	773213
2572	NearestLo	Chr13	1139145	1139476	332	+	6627	1135684
728	NearestLo	Chr13	1676293	1676720	428	+	6683	1673651
2285	NearestLo	Chr13	1738456	1738780	325	+	6716	1741029
7107	NearestLo	Chr13	3360477	3360709	233	+	6770	3353107
359	NearestLo	Chr13	3621413	3621966	554	+	6720	3621326
1938	NearestLo	Chr13	3767369	3767571	203	+	6939	3771465
763	NearestLo	Chr13	3991763	3991998	236	+	6891	3991611
14181	NearestLo	Chr13	5296924	5297235	312	+	6613	5311388
215	NearestLo	Chr13	5973920	5974132	213	+	6843	5972786
384	NearestLo	Chr13	6403043	6403545	503	+	6690	6402922
384	NearestLo	Chr13	6402204	6402570	367	+	6628	6402922
156	NearestLo	Chr13	6403043	6403545	503	+	6690	6402922
156	NearestLo	Chr13	6402204	6402570	367	+	6628	6402922
158	NearestLo	Chr13	6403887	6404421	535	+	6624	6404076
75	NearestLo	Chr13	6408424	6409853	1430	+	6695	6408512
23	NearestLo	Chr13	6410026	6410750	725	+	6713	6410411
305	NearestLo	Chr13	6414261	6415217	957	+	6659	6413755
297	NearestLo	Chr13	6538032	6538356	325	+	6729	6539055
13179	NearestLo	Chr13	8397124	8397388	265	+	6819	8382096
11984	NearestLo	Chr13	8397124	8397388	265	+	6819	8382096
300	NearestLo	Chr13	8652674	8653305	632	+	6772	8651425
701	NearestLo	Chr13	9592800	9593201	402	+	6873	9585579
701	NearestLo	Chr13	9591988	9592282	295	+	6687	9585579
12	NearestLo	Chr13	9857156	9857573	418	+	6650	9857154
84	NearestLo	Chr13	10736167	10736405	239	+	6704	10736488
84	NearestLo	Chr13	10738340	10738778	439	+	6849	10736488
1068	NearestLo	Chr13	10736167	10736405	239	+	6704	10736488
1068	NearestLo	Chr13	10738340	10738778	439	+	6849	10736488
11	NearestLo	Chr13	11150566	11150786	221	+	6947	11147296
19841	NearestLo	Chr13	11194217	11194524	308	+	6809	11168807
587	NearestLo	Chr13	12677267	12677536	270	+	6850	12673726
823	NearestLo	Chr13	12941705	12942875	1171	+	6625	12941227
2783	NearestLo	Chr13	13250917	13251424	508	+	6805	13248572
3834	NearestLo	Chr13	13250917	13251424	508	+	6805	13248572
2503	NearestLo	Chr13	13614224	13614430	207	+	6818	13611002
3166	NearestLo	Chr13	13967575	13968054	480	+	6642	13970999
693	NearestLo	Chr13	14521349	14521564	216	+	6842	14518669
198	NearestLo	Chr13	14664160	14664880	721	+	6679	14666884
198	NearestLo	Chr13	14665032	14667253	2222	+	6678	14666884
411	NearestLo	Chr13	14684602	14685051	450	+	6765	14684272
33	NearestLo	Chr13	14754829	14755572	744	+	6662	14754882

227	NearestLo	Chr13	14757910	14758370	461	+	6621	14757219
227	NearestLo	Chr13	14756168	14757623	1456	+	6703	14757219
68	NearestLo	Chr13	14768265	14768761	497	+	6681	14765441
68	NearestLo	Chr13	14767518	14767774	257	+	6616	14765441
68	NearestLo	Chr13	14765484	14765870	387	+	6611	14765441
2662	NearestLo	Chr13	14768265	14768761	497	+	6681	14765441
2662	NearestLo	Chr13	14767518	14767774	257	+	6616	14765441
2662	NearestLo	Chr13	14765484	14765870	387	+	6611	14765441
1888	NearestLo	Chr13	14768265	14768761	497	+	6681	14765441
1888	NearestLo	Chr13	14767518	14767774	257	+	6616	14765441
1888	NearestLo	Chr13	14765484	14765870	387	+	6611	14765441
632	NearestLo	Chr13	14770004	14770929	926	+	6666	14771293
632	NearestLo	Chr13	14768936	14769157	222	+	6618	14771293
339	NearestLo	Chr13	14773469	14773840	372	+	6689	14772455
339	NearestLo	Chr13	14772522	14772898	377	+	6646	14772455
93	NearestLo	Chr13	14773469	14773840	372	+	6689	14772455
93	NearestLo	Chr13	14772522	14772898	377	+	6646	14772455
3	NearestLo	Chr13	14775325	14775953	629	+	6665	14775554
536	NearestLo	Chr13	14779114	14779329	216	+	6674	14779861
854	NearestLo	Chr13	14785244	14785580	337	+	6605	14783992
854	NearestLo	Chr13	14783257	14783609	353	+	6761	14783992
17	NearestLo	Chr13	14788549	14789195	647	+	6653	14788312
7	NearestLo	Chr13	14789521	14790192	672	+	6693	14790129
7	NearestLo	Chr13	14790342	14791189	848	+	6649	14790129
39	NearestLo	Chr13	14789521	14790192	672	+	6693	14790129
39	NearestLo	Chr13	14790342	14791189	848	+	6649	14790129
503	NearestLo	Chr13	14789521	14790192	672	+	6693	14790129
503	NearestLo	Chr13	14790342	14791189	848	+	6649	14790129
267	NearestLo	Chr13	14792080	14792323	244	+	6698	14791829
13	NearestLo	Chr13	14793647	14795268	1622	+	6699	14794072
223	NearestLo	Chr13	14814095	14814363	269	+	6632	14814563
61	NearestLo	Chr13	14836645	14837091	447	+	6680	14836710
61	NearestLo	Chr13	14836029	14836276	248	+	6612	14836710
1108	NearestLo	Chr13	14838890	14839423	534	+	6630	14837889
1108	NearestLo	Chr13	14839724	14839981	258	+	6669	14837889
289	NearestLo	Chr13	14838890	14839423	534	+	6630	14837889
289	NearestLo	Chr13	14839724	14839981	258	+	6669	14837889
1745	NearestLo	Chr13	14842219	14842823	605	+	6654	14844509
1745	NearestLo	Chr13	14844884	14847136	2253	+	6705	14844509
1745	NearestLo	Chr13	14843052	14843578	527	+	6658	14844509
951	NearestLo	Chr13	14842219	14842823	605	+	6654	14844509
951	NearestLo	Chr13	14844884	14847136	2253	+	6705	14844509
951	NearestLo	Chr13	14843052	14843578	527	+	6658	14844509
383	NearestLo	Chr13	14842219	14842823	605	+	6654	14844509
383	NearestLo	Chr13	14844884	14847136	2253	+	6705	14844509
383	NearestLo	Chr13	14843052	14843578	527	+	6658	14844509
279	NearestLo	Chr13	14848683	14850110	1428	+	6708	14849394
279	NearestLo	Chr13	14847318	14847713	396	+	6668	14849394
83	NearestLo	Chr13	14851924	14852343	420	+	6641	14851611
90	NearestLo	Chr13	14853508	14853942	435	+	6643	14853384
90	NearestLo	Chr13	14852589	14853090	502	+	6655	14853384
243	NearestLo	Chr13	14865853	14866582	730	+	6640	14865774
243	NearestLo	Chr13	14865326	14865614	289	+	6671	14865774
758	NearestLo	Chr13	14865853	14866582	730	+	6640	14865774

758	NearestLoChr13	14865326	14865614	289	+	6671	14865774
446	NearestLoChr13	14879643	14880840	1198	+	6717	14880122
446	NearestLoChr13	14878684	14879065	382	+	6606	14880122
325	NearestLoChr13	14883080	14883318	239	+	6608	14882770
325	NearestLoChr13	14881738	14882536	799	+	6607	14882770
94	NearestLoChr13	14896103	14896420	318	+	6609	14894928
2526	NearestLoChr13	14907349	14907573	225	+	6706	14901898
2526	NearestLoChr13	14904720	14905094	375	+	6675	14901898
2526	NearestLoChr13	14903992	14904198	207	+	6711	14901898
46	NearestLoChr13	14907349	14907573	225	+	6706	14901898
46	NearestLoChr13	14904720	14905094	375	+	6675	14901898
46	NearestLoChr13	14903992	14904198	207	+	6711	14901898
8675	NearestLoChr13	14998220	14998787	568	+	6910	14996475
8675	NearestLoChr13	14987536	14987876	341	+	6660	14996475
1323	NearestLoChr13	15836504	15836764	261	+	6626	15832819
2014	NearestLoChr13	16069795	16070026	232	+	6865	16066967
1356	NearestLoChr13	16164361	16164595	235	+	6780	16162962
917	NearestLoChr14	735877	736192	316	+	6995	731206
931	NearestLoChr14	1492171	1492391	221	+	7258	1489589
931	NearestLoChr14	1489676	1489894	219	+	7254	1489589
1948	NearestLoChr14	1634982	1635401	420	+	7029	1629064
1948	NearestLoChr14	1633845	1634678	834	+	7303	1629064
645	NearestLoChr14	2167576	2168099	524	+	7151	2169818
645	NearestLoChr14	2170311	2170975	665	+	7242	2169818
5328	NearestLoChr14	3325551	3325769	219	+	7143	3318496
1201	NearestLoChr14	4240173	4240444	272	+	7073	4238803
886	NearestLoChr14	4256208	4256478	271	+	7003	4255382
3118	NearestLoChr14	5345748	5345956	209	+	7280	5351464
1904	NearestLoChr14	6232261	6232579	319	+	7111	6224064
578	NearestLoChr14	6386455	6386785	331	+	6957	6386405
941	NearestLoChr14	6431162	6431656	495	+	6962	6429060
2105	NearestLoChr14	6975151	6975360	210	+	7059	6968470
2147	NearestLoChr14	6983051	6983347	297	+	7271	6980125
4075	NearestLoChr14	7291807	7292124	318	+	7067	7287631
925	NearestLoChr14	8147095	8148194	1100	+	6992	8140542
1041	NearestLoChr14	9202168	9202461	294	+	7114	9199617
3723	NearestLoChr14	9343542	9343856	315	+	7273	9349076
3723	NearestLoChr14	9345133	9345354	222	+	7053	9349076
134	NearestLoChr14	10405919	10406336	418	+	6960	10403873
134	NearestLoChr14	10404298	10405198	901	+	7095	10403873
1908	NearestLoChr14	11077190	11077433	244	+	6986	11073134
1908	NearestLoChr14	11075004	11075233	230	+	7447	11073134
225	NearestLoChr14	12397172	12397382	211	+	7410	12397146
225	NearestLoChr14	12396041	12396284	244	+	7359	12397146
204	NearestLoChr14	12608007	12608228	222	+	7466	12601436
19073	NearestLoChr14	12934555	12934820	266	+	7377	12925765
19073	NearestLoChr14	12938335	12938561	227	+	7185	12925765
26870	NearestLoChr14	14747901	14748285	385	+	7289	14743200
156	NearestLoChr14	15178282	15178792	511	+	7007	15176521
1124	NearestLoChr14	15933503	15933706	204	+	7107	15925039
15231	NearestLoChr14	16520637	16521285	649	+	6990	16505398
3853	NearestLoChr14	16984125	16984785	661	+	7063	16984035
3853	NearestLoChr14	16978864	16980206	1343	+	7071	16984035
3853	NearestLoChr14	16983479	16983946	468	+	7037	16984035

3853	NearestLoChr14	16985023	16985250	228	+	6974	16984035
3853	NearestLoChr14	16981925	16983010	1086	+	7089	16984035
34935	NearestLoChr14	17215382	17215655	274	+	6975	17226201
10573	NearestLoChr14	17215382	17215655	274	+	6975	17226201
3627	NearestLoChr14	18135695	18135909	215	+	7060	18139660
6825	NearestLoChr14	18333000	18333227	228	+	7030	18320205
161	NearestLoChr14	18419622	18419998	377	+	6979	18419623
632	NearestLoChr15	459173	459402	230	+	7607	458876
3479	NearestLoChr15	1860722	1861002	281	+	7624	1860897
578	NearestLoChr15	1899654	1900049	396	+	7476	1899112
122	NearestLoChr15	2343000	2343248	249	+	7674	2345768
9936	NearestLoChr15	3512641	3513095	455	+	7517	3510981
1817	NearestLoChr15	4009029	4009232	204	+	7755	4011222
3001	NearestLoChr15	4095939	4096479	541	+	7617	4096035
2288	NearestLoChr15	4557981	4558256	276	+	7498	4554466
21419	NearestLoChr15	4956927	4957174	248	+	7533	4970293
21419	NearestLoChr15	4967856	4968252	397	+	7470	4970293
21419	NearestLoChr15	4970123	4970375	253	+	7618	4970293
25618	NearestLoChr15	4956927	4957174	248	+	7533	4970293
25618	NearestLoChr15	4967856	4968252	397	+	7470	4970293
25618	NearestLoChr15	4970123	4970375	253	+	7618	4970293
2062	NearestLoChr15	4956927	4957174	248	+	7533	4970293
2062	NearestLoChr15	4967856	4968252	397	+	7470	4970293
2062	NearestLoChr15	4970123	4970375	253	+	7618	4970293
20	NearestLoChr15	6265642	6265961	320	+	7484	6288546
20	NearestLoChr15	6289627	6289847	221	+	7529	6288546
20	NearestLoChr15	6132558	6132795	238	+	7765	6288546
155730	NearestLoChr15	6265642	6265961	320	+	7484	6288546
155730	NearestLoChr15	6289627	6289847	221	+	7529	6288546
155730	NearestLoChr15	6132558	6132795	238	+	7765	6288546
1732	NearestLoChr15	6323292	6326138	2847	+	7527	6322416
1732	NearestLoChr15	6322474	6322907	434	+	7508	6322416
10	NearestLoChr15	6323292	6326138	2847	+	7527	6322416
10	NearestLoChr15	6322474	6322907	434	+	7508	6322416
2005	NearestLoChr15	6327215	6328289	1075	+	7519	6328465
2005	NearestLoChr15	6329774	6330239	466	+	7513	6328465
2005	NearestLoChr15	6328486	6328952	467	+	7495	6328465
2005	NearestLoChr15	6326497	6326761	265	+	7482	6328465
2005	NearestLoChr15	6330976	6331567	592	+	7542	6328465
70	NearestLoChr15	6327215	6328289	1075	+	7519	6328465
70	NearestLoChr15	6329774	6330239	466	+	7513	6328465
70	NearestLoChr15	6328486	6328952	467	+	7495	6328465
70	NearestLoChr15	6326497	6326761	265	+	7482	6328465
70	NearestLoChr15	6330976	6331567	592	+	7542	6328465
793	NearestLoChr15	6327215	6328289	1075	+	7519	6328465
793	NearestLoChr15	6329774	6330239	466	+	7513	6328465
793	NearestLoChr15	6328486	6328952	467	+	7495	6328465
793	NearestLoChr15	6326497	6326761	265	+	7482	6328465
793	NearestLoChr15	6330976	6331567	592	+	7542	6328465
817	NearestLoChr15	6327215	6328289	1075	+	7519	6328465
817	NearestLoChr15	6329774	6330239	466	+	7513	6328465
817	NearestLoChr15	6328486	6328952	467	+	7495	6328465
817	NearestLoChr15	6326497	6326761	265	+	7482	6328465
817	NearestLoChr15	6330976	6331567	592	+	7542	6328465

2351	NearestLo	Chr15	6327215	6328289	1075	+	7519	6328465
2351	NearestLo	Chr15	6329774	6330239	466	+	7513	6328465
2351	NearestLo	Chr15	6328486	6328952	467	+	7495	6328465
2351	NearestLo	Chr15	6326497	6326761	265	+	7482	6328465
2351	NearestLo	Chr15	6330976	6331567	592	+	7542	6328465
1720	NearestLo	Chr15	6327215	6328289	1075	+	7519	6328465
1720	NearestLo	Chr15	6329774	6330239	466	+	7513	6328465
1720	NearestLo	Chr15	6328486	6328952	467	+	7495	6328465
1720	NearestLo	Chr15	6326497	6326761	265	+	7482	6328465
1720	NearestLo	Chr15	6330976	6331567	592	+	7542	6328465
670	NearestLo	Chr15	6397874	6398620	747	+	7480	6406266
670	NearestLo	Chr15	6409207	6409409	203	+	7610	6406266
6846	NearestLo	Chr15	6571571	6571777	207	+	7582	6581611
49765	NearestLo	Chr15	6682165	6682373	209	+	7655	6631345
12289	NearestLo	Chr15	6969554	6969763	210	+	7627	6956049
5473	NearestLo	Chr15	7432183	7432451	269	+	7626	7437985
93	NearestLo	Chr15	8388202	8388601	400	+	7474	8381611
93	NearestLo	Chr15	8382777	8383011	235	+	7736	8381611
403	NearestLo	Chr15	8887139	8887341	203	+	7654	8886714
25	NearestLo	Chr15	10235078	10235326	249	+	7478	10235060
133	NearestLo	Chr15	11321185	11321390	206	+	7776	11320982
4018	NearestLo	Chr15	12313753	12313975	223	+	7777	12317939
445	NearestLo	Chr15	13655966	13656171	206	+	7697	13653311
87	NearestLo	Chr15	13712445	13712702	258	+	7497	13711820
1073	NearestLo	Chr15	14299934	14300500	567	+	7758	14297509
1294	NearestLo	Chr15	14918880	14919430	551	+	7766	14911344
1595	NearestLo	Chr15	15244260	15245048	789	+	7580	15243937
889	NearestLo	Chr16	625070	625529	460	+	7933	626427
699	NearestLo	Chr16	625070	625529	460	+	7933	626427
24	NearestLo	Chr16	905292	905573	282	+	7963	904073
643	NearestLo	Chr16	1867944	1868178	235	+	7987	1868366
96	NearestLo	Chr16	3541724	3541981	258	+	8030	3541745
68	NearestLo	Chr16	3796036	3796700	665	+	7938	3795851
16	NearestLo	Chr16	4119797	4120202	406	+	7816	4115461
457	NearestLo	Chr16	4266993	4267576	584	+	7786	4266555
4688	NearestLo	Chr16	4953396	4953647	252	+	7920	4953360
663	NearestLo	Chr16	6098376	6099948	1573	+	7781	6098378
299	NearestLo	Chr16	6579582	6580084	503	+	7800	6579485
853	NearestLo	Chr16	6678146	6678394	249	+	8003	6677907
53	NearestLo	Chr16	7275804	7276181	378	+	7820	7275771
20823	NearestLo	Chr16	7698673	7698879	207	+	8012	7710855
20823	NearestLo	Chr16	7678877	7679089	213	+	7922	7710855
20823	NearestLo	Chr16	7689824	7690031	208	+	7837	7710855
28291	NearestLo	Chr16	8130684	8130926	243	+	7891	8101978
71	NearestLo	Chr16	8218259	8218520	262	+	7792	8218191
4328	NearestLo	Chr16	11095530	11095732	203	+	8046	11100066
38	NearestLo	Chr16	12349269	12349991	723	+	7783	12343353
1275	NearestLo	Chr16	12540778	12541244	467	+	7828	12539743
2846	NearestLo	Chr16	13771808	13772067	260	+	7853	13767533
1717	NearestLo	Chr16	13992544	13992934	391	+	7815	13989680
1717	NearestLo	Chr16	13990094	13990609	516	+	7862	13989680
364	NearestLo	Chr17	804370	804586	217	+	8268	804971
950	NearestLo	Chr17	2037610	2037845	236	+	8305	2038429
353	NearestLo	Chr17	2262135	2262366	232	+	8310	2256891

2058	NearestLo	Chr17	2355649	2355864	216	+	8170	2353070
3366	NearestLo	Chr17	2665036	2665277	242	+	8096	2668624
1230	NearestLo	Chr17	3582145	3582410	266	+	8151	3583593
1230	NearestLo	Chr17	3583158	3583428	271	+	8222	3583593
9837	NearestLo	Chr17	5643454	5643877	424	+	8114	5642080
5250	NearestLo	Chr17	5868610	5868855	246	+	8079	5861150
21	NearestLo	Chr17	5940901	5941155	255	+	8074	5940893
14682	NearestLo	Chr17	5940901	5941155	255	+	8074	5940893
2148	NearestLo	Chr17	6919219	6919439	221	+	8218	6937683
35919	NearestLo	Chr17	7300012	7300249	238	+	8073	7261738
5710	NearestLo	Chr17	7358820	7359092	273	+	8080	7364780
3416	NearestLo	Chr17	8293448	8293690	243	+	8330	8296712
2971	NearestLo	Chr17	8764943	8765145	203	+	8271	8764503
2971	NearestLo	Chr17	8765327	8765538	212	+	8322	8764503
2971	NearestLo	Chr17	8769063	8769307	245	+	8085	8764503
1825	NearestLo	Chr17	9600422	9600775	354	+	8285	9599828
1825	NearestLo	Chr17	9597773	9597985	213	+	8299	9599828
1825	NearestLo	Chr17	9601289	9601726	438	+	8256	9599828
1006	NearestLo	Chr17	9633735	9633997	263	+	8217	9635852
9990	NearestLo	Chr17	10054731	10055304	574	+	8078	10065285
8893	NearestLo	Chr17	10947566	10947833	268	+	8277	10947583
9587	NearestLo	Chr17	10947566	10947833	268	+	8277	10947583
638	NearestLo	Chr17	11507969	11508283	315	+	8184	11500571
7808	NearestLo	Chr17	13004774	13005123	350	+	8135	13012818
4403	NearestLo	Chr17	13146173	13146445	273	+	8239	13149653
1452	NearestLo	Chr17	13533627	13533877	251	+	8087	13528321
169	NearestLo	Chr17	14200327	14200626	300	+	8153	14201130
102	NearestLo	Chr17	14836774	14837064	291	+	8076	14835641
102	NearestLo	Chr17	14835947	14836159	213	+	8317	14835641
524	NearestLo	Chr17	14837438	14837660	223	+	8088	14838177
9916	NearestLo	Chr17	15101473	15102293	821	+	8101	15112186
944	NearestLo	Chr17	15518492	15519060	569	+	8236	15517208
2066	NearestLo	Chr18	1278817	1279082	266	+	8594	1274099
9	NearestLo	Chr18	2321314	2322109	796	+	8375	2319968
1120	NearestLo	Chr18	2343963	2344172	210	+	8626	2345287
234	NearestLo	Chr18	2437330	2437741	412	+	8353	2436803
1710	NearestLo	Chr18	2682007	2682487	481	+	8646	2680940
234	NearestLo	Chr18	3016567	3016795	229	+	8600	3015343
234	NearestLo	Chr18	3014948	3015277	330	+	8664	3015343
11690	NearestLo	Chr18	3107667	3107900	234	+	8355	3119563
4660	NearestLo	Chr18	3237122	3237403	282	+	8460	3234729
141	NearestLo	Chr18	4061294	4061847	554	+	8337	4061102
9786	NearestLo	Chr18	4128450	4128732	283	+	8428	4122412
9786	NearestLo	Chr18	4112361	4112579	219	+	8507	4122412
9786	NearestLo	Chr18	4111222	4111651	430	+	8512	4122412
9786	NearestLo	Chr18	4114811	4115015	205	+	8535	4122412
12236	NearestLo	Chr18	4690890	4691102	213	+	8407	4676605
23849	NearestLo	Chr18	5031706	5031911	206	+	8454	5006621
10219	NearestLo	Chr18	5242373	5242622	250	+	8334	5252819
31381	NearestLo	Chr18	6365437	6365879	443	+	8573	6365455
31381	NearestLo	Chr18	6367447	6367839	393	+	8480	6365455
31381	NearestLo	Chr18	6400329	6400617	289	+	8661	6365455
31381	NearestLo	Chr18	6368511	6369232	722	+	8432	6365455
80346	NearestLo	Chr18	6536831	6537131	301	+	8425	6532966

80346	NearestLo	Chr18	6608419	6608777	359	+	8379	6532966
80346	NearestLo	Chr18	6581305	6581645	341	+	8509	6532966
70936	NearestLo	Chr18	6536831	6537131	301	+	8425	6532966
70936	NearestLo	Chr18	6608419	6608777	359	+	8379	6532966
70936	NearestLo	Chr18	6581305	6581645	341	+	8509	6532966
18229	NearestLo	Chr18	7369611	7369817	207	+	8552	7374464
18229	NearestLo	Chr18	7356005	7356256	252	+	8336	7374464
1558	NearestLo	Chr18	7766584	7766809	226	+	8550	7766617
1558	NearestLo	Chr18	7766036	7766397	362	+	8358	7766617
2735	NearestLo	Chr18	8130640	8131182	543	+	8335	8133687
146	NearestLo	Chr18	11647403	11647637	235	+	8658	11640585
6627	NearestLo	Chr18	11706496	11706721	226	+	8398	11697912
8495	NearestLo	Chr18	12568759	12569138	380	+	8639	12568154
8495	NearestLo	Chr18	12569614	12570012	399	+	8402	12568154
8495	NearestLo	Chr18	12570248	12570481	234	+	8565	12568154
8495	NearestLo	Chr18	12559343	12559680	338	+	8412	12568154
2392	NearestLo	Chr18	12640579	12640840	262	+	8423	12643275
15	NearestLo	Chr18	12640579	12640840	262	+	8423	12643275
1554	NearestLo	Chr18	12797394	12797673	280	+	8372	12799233
3665	NearestLo	Chr18	13033696	13033943	248	+	8466	13029187
628	NearestLo	Chr18	15517353	15517554	202	+	8544	15513707
321	NearestLo	Chr18	15591517	15591801	285	+	8362	15590333
180	NearestLo	Chr19	286311	286640	330	+	8824	286427
9	NearestLo	Chr19	456126	456328	203	+	8808	457316
254	NearestLo	Chr19	1430719	1431143	425	+	8748	1432709
254	NearestLo	Chr19	1435583	1435904	322	+	8862	1432709
4017	NearestLo	Chr19	1998577	1998817	241	+	8775	1992958
1990	NearestLo	Chr19	2143251	2143463	213	+	8745	2145435
63	NearestLo	Chr19	2183364	2183592	229	+	8791	2174154
1242	NearestLo	Chr19	2311667	2312148	482	+	8859	2309395
79	NearestLo	Chr19	2751701	2751923	223	+	8746	2751574
67	NearestLo	Chr19	3188215	3188439	225	+	8704	3188159
67	NearestLo	Chr19	3188869	3189162	294	+	8688	3188159
477	NearestLo	Chr19	3193963	3194213	251	+	8693	3192473
477	NearestLo	Chr19	3192904	3193133	230	+	8682	3192473
364	NearestLo	Chr19	3193963	3194213	251	+	8693	3192473
364	NearestLo	Chr19	3192904	3193133	230	+	8682	3192473
80	NearestLo	Chr19	3194878	3195700	823	+	8698	3194810
141	NearestLo	Chr19	3202448	3202663	216	+	8700	3201672
141	NearestLo	Chr19	3202876	3203481	606	+	8672	3201672
935	NearestLo	Chr19	3831884	3832105	222	+	8847	3830946
5535	NearestLo	Chr19	4767545	4767819	275	+	8809	4758852
5535	NearestLo	Chr19	4759110	4759497	388	+	8726	4758852
5535	NearestLo	Chr19	4766995	4767211	217	+	8738	4758852
1172	NearestLo	Chr19	4775654	4775876	223	+	8717	4777056
18	NearestLo	Chr19	5320873	5321130	258	+	8675	5320857
4997	NearestLo	Chr19	5545986	5546567	582	+	8695	5539256
271	NearestLo	Chr19	6691062	6691627	566	+	8696	6688729
4156	NearestLo	Chr19	7541173	7541449	277	+	8819	7540234
4864	NearestLo	Chr19	7834028	7834276	249	+	8727	7845538
4864	NearestLo	Chr19	7834489	7834717	229	+	8714	7845538
5355	NearestLo	Chr19	7834028	7834276	249	+	8727	7845538
5355	NearestLo	Chr19	7834489	7834717	229	+	8714	7845538
73	NearestLo	Chr19	9986015	9986230	216	+	8828	9984160

73	NearestLo	Chr19	9984068	9984273	206	+	8844	9984160
73	NearestLo	Chr19	9982550	9982763	214	+	8864	9984160
73	NearestLo	Chr19	9986709	9987039	331	+	8686	9984160
783	NearestLo	Chr19	9986015	9986230	216	+	8828	9984160
783	NearestLo	Chr19	9984068	9984273	206	+	8844	9984160
783	NearestLo	Chr19	9982550	9982763	214	+	8864	9984160
783	NearestLo	Chr19	9986709	9987039	331	+	8686	9984160
62	NearestLo	Chr19	10013668	10014085	418	+	8732	10011999
2722	NearestLo	Chr19	11350405	11350749	345	+	8687	11346583
2722	NearestLo	Chr19	11356320	11356658	339	+	8842	11346583
549	NearestLo	Chr19	11796994	11797294	301	+	8812	11796718
549	NearestLo	Chr19	11799071	11799369	299	+	8713	11796718
549	NearestLo	Chr19	11799552	11800325	774	+	8723	11796718
58	NearestLo	Chr19	12331629	12331832	204	+	8836	12331168
39	NearestLo	Chr19	12958539	12958800	262	+	8800	12958978
42	NearestLo	Chr19	13062545	13062824	280	+	8751	13059593
42	NearestLo	Chr19	13060284	13060878	595	+	8804	13059593
4851	NearestLo	Chr19	13312606	13312949	344	+	8712	13306929
347	NearestLo	Chr19	15371154	15372227	1074	+	8671	15371086
38817	NearestLo	scaffold_	235949	236176	228	+	8918	193697
38817	NearestLo	scaffold_	196750	197081	332	+	8921	193697
194	NearestLo	scaffold_	339107	339338	232	+	8940	435395
194	NearestLo	scaffold_	337856	338120	265	+	8934	435395
194	NearestLo	scaffold_	436758	436984	227	+	8937	435395
194	NearestLo	scaffold_	339886	340251	366	+	8933	435395
194	NearestLo	scaffold_	435704	435935	232	+	8935	435395
318	NearestLo	scaffold_	339107	339338	232	+	8940	435395
318	NearestLo	scaffold_	337856	338120	265	+	8934	435395
318	NearestLo	scaffold_	436758	436984	227	+	8937	435395
318	NearestLo	scaffold_	339886	340251	366	+	8933	435395
318	NearestLo	scaffold_	435704	435935	232	+	8935	435395
171	NearestLo	scaffold_	438728	439429	702	+	8938	438693
25	NearestLo	scaffold_	746242	746679	438	+	8946	746690
74	NearestLo	scaffold_	101979	102181	203	+	8956	100631
74	NearestLo	scaffold_	101214	101725	512	+	8952	100631
7539	NearestLo	scaffold_	388187	388391	205	+	8958	386255
7879	NearestLo	scaffold_	466620	466842	223	+	8963	474713
6499	NearestLo	scaffold_	192813	193041	229	+	8966	185949
41966	NearestLo	scaffold_	188264	188689	426	+	8968	230695
41966	NearestLo	scaffold_	226701	227030	330	+	8967	230695
3694	NearestLo	scaffold_	188264	188689	426	+	8968	230695
3694	NearestLo	scaffold_	226701	227030	330	+	8967	230695
1398	NearestLo	scaffold_	127718	127951	234	+	8973	125839
56995	NearestLo	scaffold_	229672	229893	222	+	8989	286887
21207	NearestLo	scaffold_	229672	229893	222	+	8989	286887
2080	NearestLo	scaffold_	247049	247558	510	+	8998	243811
2080	NearestLo	scaffold_	246196	246642	447	+	8999	243811
2080	NearestLo	scaffold_	247874	248100	227	+	9000	243811
2934	NearestLo	scaffold_	247049	247558	510	+	8998	243811
2934	NearestLo	scaffold_	246196	246642	447	+	8999	243811
2934	NearestLo	scaffold_	247874	248100	227	+	9000	243811
3763	NearestLo	scaffold_	247049	247558	510	+	8998	243811
3763	NearestLo	scaffold_	246196	246642	447	+	8999	243811
3763	NearestLo	scaffold_	247874	248100	227	+	9000	243811

1802	NearestLoscaffold_	263605	263806	202	+	9001	261478
6765	NearestLoscaffold_	272202	272504	303	+	8997	279248
4743	NearestLoscaffold_	109291	109503	213	+	9004	97055
4743	NearestLoscaffold_	78861	79064	204	+	9007	97055
4743	NearestLoscaffold_	114355	114563	209	+	9006	97055
4743	NearestLoscaffold_	102611	102865	255	+	9003	97055
17787	NearestLoscaffold_	109291	109503	213	+	9004	97055
17787	NearestLoscaffold_	78861	79064	204	+	9007	97055
17787	NearestLoscaffold_	114355	114563	209	+	9006	97055
17787	NearestLoscaffold_	102611	102865	255	+	9003	97055
1845	NearestLoscaffold_	153531	153745	215	+	9005	150933
3242	NearestLoscaffold_	51355	51629	275	+	9013	54839
339	NearestLoscaffold_	193669	193914	246	+	9014	194888
3710	NearestLoscaffold_	181288	181516	229	+	9025	183283
11366	NearestLoscaffold_	183395	183608	214	+	9031	194973
904	NearestLoscaffold_	106098	106404	307	+	9045	109338
904	NearestLoscaffold_	109790	110079	290	+	9044	109338
904	NearestLoscaffold_	112717	113001	285	+	9049	109338
3403	NearestLoscaffold_	16657	16936	280	+	9065	7345
2184	NearestLoscaffold_	16657	16936	280	+	9065	7345
43	NearestLoscaffold_	70794	71734	941	+	9067	70232
1193	NearestLoscaffold_	124676	124904	229	+	9068	118815
954	NearestLoscaffold_	16891	17153	263	+	8880	18047
967	NearestLoscaffold_	3334	3877	544	+	8893	3275
967	NearestLoscaffold_	2054	2323	270	+	8891	3275
11174	NearestLoscaffold_	16641	16877	237	+	8896	28046
3496	NearestLoscaffold_	58572	58843	272	+	8899	62462
6986	NearestLoscaffold_	15516	17355	1840	+	8905	6537
6986	NearestLoscaffold_	14989	15360	372	+	8903	6537
7490	NearestLoscaffold_	15516	17355	1840	+	8905	6537
7490	NearestLoscaffold_	14989	15360	372	+	8903	6537
5097	NearestLoscaffold_	23389	23780	392	+	8904	29049
26	NearestLoscaffold_	61170	61740	571	+	8906	61152
7423	NearestLoscaffold_	54048	54273	226	+	8907	46265
656	NearestLoscaffold_	8088	8337	250	+	8908	8978
7721	NearestLoscaffold_	37805	38022	218	+	8911	28580
585	NearestLoscaffold_	70137	70363	227	+	8914	70936
1875	NearestLoscaffold_	23959	24168	210	+	8916	16739
1875	NearestLoscaffold_	30567	30781	215	+	8917	16739
2912	NearestLoscaffold_	48305	48554	250	+	8915	51459
11149	NearestLoscaffold_	13056	13268	213	+	9042	15266
12	NearestLoscaffold_	12141	12748	608	+	9077	12142
2620	NearestLoscaffold_	1638	1915	278	+	8902	4548
2620	NearestLoscaffold_	3268	3872	605	+	8901	4548
1286	NearestLoscaffold_	1990	2198	209	+	8930	327

end_posit	feature_s	insideFea	distancet	shortestD	fromOverl	Pfam	Panther	KOG
937283	+	inside	850	491	NearestLc	PF00439,P	PTHR37888,PTHR3788	
3616418	+	downstrea	14361	6700	NearestLc	PF14510,P	PTHR19241,PTHR1924	
3616418	+	downstrea	19396	11754	NearestLc	PF14510,P	PTHR19241,PTHR1924	
3616418	+	downstrea	11259	3604	NearestLc	PF14510,P	PTHR19241,PTHR1924	
3662745	+	upstream	-13296	13131	NearestLc	PF14510,P	PTHR19241,PTHR1924	
4425107	-	inside	285	10	NearestLocation			
4820010	+	inside	425	296	NearestLocation		PTHR31568	
5083400	-	upstream	-374	234	NearestLc	PF13855	PTHR27004,PTHR2700	
5164215	+	downstrea	617	50	NearestLc	PF01535	PTHR24015	
5526193	+	upstream	-512	378	NearestLc	PF00847	PTHR31677,PTHR3167	
5596585	+	inside	166	38	NearestLc	PF14259		
5713239	-	downstrea	1539	795	NearestLocation			
5713239	-	downstrea	2181	1435	NearestLocation			
6548358	+	inside	549	423	NearestLc	PF07714,P	PTHR27006	KOG1187
6548358	+	inside	4253	3717	NearestLc	PF07714,P	PTHR27006	KOG1187
7118040	+	downstrea	1432	67	NearestLc	PF03168	PTHR31852,PTHR3185	
7118040	+	inside	744	116	NearestLc	PF03168	PTHR31852,PTHR3185	
7204932	+	overlapSt	366	6	NearestLc	PF13947,P	PTHR14155,PTHR1415	
7204932	+	inside	1130	984	NearestLc	PF13947,P	PTHR14155,PTHR1415	
7204932	+	inside	2416	419	NearestLc	PF13947,P	PTHR14155,PTHR1415	
9209012	-	overlapEn	794	37	NearestLc	PF03479	PTHR31100,PTHR3110	
9500697	+	inside	1264	933	NearestLc	PF02727,P	PTHR10638,PTHR1063	
9866447	+	inside	556	390	NearestLc	PF01237	PTHR10972	KOG2209
9963181	+	downstrea	3458	2282	NearestLc	PF15054	PTHR33528,PTHR3352	
11494939	+	upstream	-1638	1496	NearestLc	PF05678	PTHR33783,PTHR3378	
11544254	-	upstream	-19832	19417	NearestLc	PF03479	PTHR31100,PTHR3110	
12723517	+	inside	924	122	NearestLc	PF00847	PTHR31677,PTHR3167	
13556516	+	downstrea	6379	5869	NearestLocation			
15237125	-	downstrea	15706	13036	NearestLc	PF01643	PTHR31727,PTHR3172	
15237125	-	inside	2295	58	NearestLc	PF01643	PTHR31727,PTHR3172	
15640319	-	downstrea	2429	1926	NearestLocation		PTHR33168,PTHR3316	
16183416	-	overlapSt	132	137	NearestLc	PF03638	PTHR12446,PTHR1244	
17041802	+	inside	6893	83	NearestLc	PF03985	PTHR23188	KOG2478
17656082	-	downstrea	6483	419	NearestLc	PF13371,P	PTHR31350,PTHR3135	
18772544	+	inside	4355	108	NearestLc	PF01975	PTHR30457,PTHR3045	
18809389	-	upstream	-29330	29219	NearestLocation		PTHR33176	
19162246	-	upstream	-4383	4252	NearestLocation		PTHR34072	
19852980	+	overlapSt	67	49	NearestLc	PF00587	PTHR11778,PTHR1177	
20661501	+	upstream	-661	374	NearestLocation		PTHR31827,PTHR3182	
20861958	-	inside	5206	121	NearestLc	PF03168	PTHR31234,PTHR3123	
20958253	-	inside	8469	5895	NearestLc	PF12333	PTHR16056	KOG2149
21098755	-	inside	1035	793	NearestLc	PF00314	PTHR31048,PTHR3104	
21738310	+	inside	3157	3047	NearestLc	PF09011	PTHR31675,PTHR3167	
21738310	+	downstrea	7069	409	NearestLc	PF09011	PTHR31675,PTHR3167	
22367602	-	inside	2058	1915	NearestLc	PF08263,P	PTHR27001	KOG1187
22843912	-	overlapEn	1368	1	NearestLc	PF00314	PTHR31048,PTHR3104	
23054718	-	inside	6730	178	NearestLc	PF03643	PTHR12233,PTHR1223	
24559309	-	downstrea	4437	2303	NearestLc	PF06203	PTHR31319,PTHR3131	
25027873	+	inside	2179	1759	NearestLc	PF00582,P	PTHR27003	KOG1187
26613185	-	inside	142	18	NearestLocation		PTHR33068	
27816590	-	upstream	-5746	5627	NearestLc	PF00067	PTHR24286	KOG0157

27816590	-	upstream	-5746	5627	NearestLc	PF00067	PTHR24286	KOG0157
28200532	-	upstream	-2490	2363	NearestLocation		PTHR31485,PTHR3148	
28200532	-	upstream	-2490	2363	NearestLocation		PTHR31485,PTHR3148	
28658092	-	upstream	-1052	944	NearestLc	PF00249	PTHR31314	
28985938	-	inside	1044	937	NearestLc	PF03388,P	PTHR27007	KOG1187
29391222	-	inside	222	23	NearestLocation			
29750367	-	downstrea	5170	3174	NearestLocation		PTHR33347,PTHR3334	
31455199	+	upstream	-5070	4951	NearestLc	PF01092	PTHR11502	KOG1646
33457793	-	overlapSt	95	13	NearestLc	PF00124	PTHR33149,PTHR3314	
33569287	-	inside	545	258	NearestLc	PF02298	PTHR33021,PTHR3302	
33976599	-	inside	365	234	NearestLc	PF06155	PTHR35303	
34319080	+	inside	1619	368	NearestLc	PF00544	PTHR31683,PTHR3168	
34319080	+	upstream	-175	52	NearestLc	PF00544	PTHR31683,PTHR3168	
36687274	+	inside	25743	359	NearestLocation		PTHR24365	KOG1644
36719647	-	inside	284	33	NearestLc	PF04146	PTHR12357	KOG1902
36719647	-	inside	284	33	NearestLc	PF04146	PTHR12357	KOG1902
36775147	-	inside	389	193	NearestLc	PF14703,P	PTHR13018	KOG1134
38901054	-	downstrea	663	297	NearestLocation		PTHR23024,PTHR2302	
40127766	-	downstrea	4588	3553	NearestLocation		PTHR27000,PTHR2700	
40940508	-	inside	1980	477	NearestLc	PF08240,P	PTHR11695	KOG1198
41017929	-	inside	1843	1683	NearestLc	PF01503,P	PTHR21256,PTHR2125	
41349252	-	upstream	-3352	3252	NearestLc	PF00436	PTHR10302,PTHR1030	
41626633	+	downstrea	9015	7002	NearestLc	PF02458	PTHR31625,PTHR3162	
41810069	-	inside	605	178	NearestLc	PF00847	PTHR31677,PTHR3167	
41810069	-	upstream	-5041	4861	NearestLc	PF00847	PTHR31677,PTHR3167	
41810069	-	downstrea	4417	2911	NearestLc	PF00847	PTHR31677,PTHR3167	
42016722	-	upstream	-5003	4899	NearestLc	PF05678	PTHR33402,PTHR3340	
43041031	+	inside	5573	1579	NearestLc	PF00027,P	PTHR10217,PTHR1021	
43728235	-	inside	1383	32	NearestLocation			
43728235	-	inside	589	304	NearestLocation			
43728235	-	upstream	-1863	1754	NearestLocation			
43728235	-	inside	1383	32	NearestLocation			
43728235	-	inside	589	304	NearestLocation			
43728235	-	upstream	-1863	1754	NearestLocation			
45755261	-	downstrea	36195	31364	NearestLc	PF00931	PTHR23155	KOG4658
45755261	-	downstrea	23715	18883	NearestLc	PF00931	PTHR23155	KOG4658
45801056	-	upstream	-3817	3715	NearestLocation			
45801056	-	upstream	-3817	3715	NearestLocation			
47423184	-	upstream	-5531	5285	NearestLocation		PTHR12112,PTHR1211	
47423184	-	inside	620	220	NearestLocation		PTHR12112,PTHR1211	
47423184	-	upstream	-13584	13413	NearestLocation		PTHR12112,PTHR1211	
48295061	-	downstrea	4439	3536	NearestLc	PF14009	PTHR33148,PTHR3314	
48747110	+	downstrea	3441	623	NearestLc	PF02362	PTHR31140,PTHR3114	
48827279	+	overlapSt	-19	127	NearestLc	PF04096	PTHR23198	KOG0845
49436406	+	inside	402	225	NearestLc	PF03106	PTHR32096,PTHR3209	
49662020	+	upstream	-1117	992	NearestLc	PF08031,P	PTHR32448,PTHR3244	
242875	-	overlapSt	325	17	NearestLc	PF00368	PTHR10572,PTHR1057	
1270182	-	downstrea	6046	5504	NearestLocation			
1270182	-	downstrea	6046	5504	NearestLocation			
1764861	+	inside	1637	1352	NearestLc	PF00137	PTHR10263	KOG0232
1814888	-	upstream	-9885	9751	NearestLc	PF00643	PTHR31832,PTHR3183	
1814888	-	upstream	-9885	9751	NearestLc	PF00643	PTHR31832,PTHR3183	
1838158	-	downstrea	10021	2782	NearestLc	PF00498,P	PTHR13233,PTHR1323	
2565072	-	inside	535	324	NearestLc	PF04109	PTHR13038,PTHR1303	

2560412	+	inside	178	27	NearestLc	PF00223		
2807203	+	inside	447	191	NearestLc	PF03208	PTHR19317	KOG3142
2807203	+	inside	447	191	NearestLc	PF03208	PTHR19317	KOG3142
3261425	-	upstream	-5275	5127	NearestLc	PF07765	PTHR32258	KOG0978
3485264	+	inside	855	312	NearestLc	PF01734	PTHR32241	KOG0513
3485264	+	upstream	-275	146	NearestLc	PF01734	PTHR32241	KOG0513
3675093	+	inside	3832	3391	NearestLc	PF02309, P	PTHR31384, PTHR3138	
3675093	+	upstream	-409	259	NearestLc	PF02309, P	PTHR31384, PTHR3138	
3751459	-	overlapSt	115	76	NearestLocation			
3786916	+	overlapSt	31	173	NearestLc	PF03798	PTHR13439	KOG4561
3851551	+	inside	587	108	NearestLc	PF00903	PTHR11959	KOG0638
4995734	-	inside	1151	910	NearestLc	PF03547	PTHR31752, PTHR3175	
5523733	-	upstream	-789	656	NearestLc	PF01490	PTHR22950	KOG1303
5974534	-	inside	1301	1200	NearestLc	PF00892	PTHR31218, PTHR3121	
6137820	-	upstream	-2350	2224	NearestLc	PF01490	PTHR22950	KOG1303
6761062	+	inside	1521	407	NearestLc	PF00847	PTHR31657, PTHR3165	
6761062	+	upstream	-3399	3264	NearestLc	PF00847	PTHR31657, PTHR3165	
6761062	+	upstream	-1613	1474	NearestLc	PF00847	PTHR31657, PTHR3165	
6811537	+	inside	1631	35	NearestLocation		PTHR31215, PTHR3121	
6820006	+	upstream	-594	453	NearestLocation		PTHR33448, PTHR3344	
6820006	+	inside	221	95	NearestLocation		PTHR33448, PTHR3344	
6999188	-	upstream	-3584	3440	NearestLc	PF00230	PTHR19139	KOG0223
7145141	+	inside	329	89	NearestLocation		PTHR24006	
7831956	+	downstrea	10510	8188	NearestLc	PF01554	PTHR11206	KOG1347
7831956	+	downstrea	11231	8921	NearestLc	PF01554	PTHR11206	KOG1347
8029976	+	inside	307	132	NearestLc	PF14531, P	PTHR24343	KOG0583
8326606	+	upstream	-3874	3770	NearestLc	PF08263, P	PTHR27000	KOG1187
8326606	+	inside	1028	665	NearestLc	PF08263, P	PTHR27000	KOG1187
9180006	+	inside	542	316	NearestLc	PF07991, P	PTHR10996, PTHR1099	
9180006	+	upstream	-6860	6731	NearestLc	PF07991, P	PTHR10996, PTHR1099	
10242056	+	inside	4419	3812	NearestLc	PF02893, P	PTHR23319	
10424588	-	upstream	-1149	993	NearestLc	PF04570	PTHR33059, PTHR3305	
10484993	+	downstrea	1543	116	NearestLc	PF00847	PTHR31985, PTHR3198	
11794866	+	inside	1563	560	NearestLc	PF00005, P	PTHR19241, PTHR1924	
12457606	+	downstrea	2008	690	NearestLc	PF03168	PTHR31459	
13298822	+	upstream	-1247	869	NearestLc	PF02701	PTHR31992, PTHR3199	
13622730	+	inside	220	20	NearestLc	PF00786, P	PTHR23177, PTHR2317	
14007334	+	upstream	-1022	907	NearestLc	PF00010	PTHR11969, PTHR1196	
14263850	+	inside	151	14	NearestLocation			
15627324	+	upstream	-4282	4127	NearestLc	PF00574	PTHR10381	KOG0840
15793426	+	downstrea	10382	4515	NearestLc	PF14310, P	PTHR30620, PTHR3062	
16767636	-	inside	293	97	NearestLocation		PTHR36775, PTHR3677	
16838633	+	inside	2394	1531	NearestLc	PF13920	PTHR23041, PTHR2304	
17127337	+	inside	748	639	NearestLocation		PTHR35928	
17656629	+	downstrea	1419	857	NearestLocation			
17656629	+	upstream	-3162	3033	NearestLocation			
17656629	+	upstream	-7147	7030	NearestLocation			
17656629	+	upstream	-1715	1606	NearestLocation			
17656629	+	downstrea	1419	857	NearestLocation			
17656629	+	upstream	-3162	3033	NearestLocation			
17656629	+	upstream	-7147	7030	NearestLocation			
17656629	+	upstream	-1715	1606	NearestLocation			
17656629	+	downstrea	1419	857	NearestLocation			
17656629	+	upstream	-3162	3033	NearestLocation			

17656629	+	upstream	-7147	7030	NearestLocation			
17656629	+	upstream	-1715	1606	NearestLocation			
17656629	+	downstrea	1419	857	NearestLocation			
17656629	+	upstream	-3162	3033	NearestLocation			
17656629	+	upstream	-7147	7030	NearestLocation			
17656629	+	upstream	-1715	1606	NearestLocation			
17802676	+	downstrea	11667	11093	NearestLc	PF00462	PTHR10168	KOG1752
18303082	+	inside	120	5	NearestLc	PF00306	PTHR15184,PTHR1518	
18358803	-	downstrea	2847	1631	NearestLc	PF00361	PTHR22773,PTHR2277	
18358803	-	downstrea	7587	6712	NearestLc	PF00361	PTHR22773,PTHR2277	
18358803	-	downstrea	1605	499	NearestLc	PF00361	PTHR22773,PTHR2277	
18358803	-	upstream	-273	113	NearestLc	PF00361	PTHR22773,PTHR2277	
18358803	-	downstrea	3721	2828	NearestLc	PF00361	PTHR22773,PTHR2277	
18358803	-	overlapEn	493	133	NearestLc	PF00361	PTHR22773,PTHR2277	
18358803	-	downstrea	5373	4267	NearestLc	PF00361	PTHR22773,PTHR2277	
18358803	-	downstrea	4451	3453	NearestLc	PF00361	PTHR22773,PTHR2277	
18358803	-	downstrea	6630	5292	NearestLc	PF00361	PTHR22773,PTHR2277	
18358803	-	downstrea	9349	8121	NearestLc	PF00361	PTHR22773,PTHR2277	
18358803	-	downstrea	2847	1631	NearestLc	PF00361	PTHR22773,PTHR2277	
18358803	-	downstrea	7587	6712	NearestLc	PF00361	PTHR22773,PTHR2277	
18358803	-	downstrea	1605	499	NearestLc	PF00361	PTHR22773,PTHR2277	
18358803	-	upstream	-273	113	NearestLc	PF00361	PTHR22773,PTHR2277	
18358803	-	downstrea	3721	2828	NearestLc	PF00361	PTHR22773,PTHR2277	
18358803	-	overlapEn	493	133	NearestLc	PF00361	PTHR22773,PTHR2277	
18358803	-	downstrea	5373	4267	NearestLc	PF00361	PTHR22773,PTHR2277	
18358803	-	downstrea	4451	3453	NearestLc	PF00361	PTHR22773,PTHR2277	
18358803	-	downstrea	6630	5292	NearestLc	PF00361	PTHR22773,PTHR2277	
18358803	-	downstrea	9349	8121	NearestLc	PF00361	PTHR22773,PTHR2277	
18358803	-	downstrea	2847	1631	NearestLc	PF00361	PTHR22773,PTHR2277	
18358803	-	downstrea	7587	6712	NearestLc	PF00361	PTHR22773,PTHR2277	
18358803	-	downstrea	1605	499	NearestLc	PF00361	PTHR22773,PTHR2277	
18358803	-	upstream	-273	113	NearestLc	PF00361	PTHR22773,PTHR2277	
18358803	-	downstrea	3721	2828	NearestLc	PF00361	PTHR22773,PTHR2277	
18358803	-	overlapEn	493	133	NearestLc	PF00361	PTHR22773,PTHR2277	
18358803	-	downstrea	5373	4267	NearestLc	PF00361	PTHR22773,PTHR2277	
18358803	-	downstrea	4451	3453	NearestLc	PF00361	PTHR22773,PTHR2277	
18358803	-	downstrea	6630	5292	NearestLc	PF00361	PTHR22773,PTHR2277	
18358803	-	downstrea	9349	8121	NearestLc	PF00361	PTHR22773,PTHR2277	
18358803	-	downstrea	2847	1631	NearestLc	PF00361	PTHR22773,PTHR2277	
18358803	-	downstrea	7587	6712	NearestLc	PF00361	PTHR22773,PTHR2277	
18358803	-	downstrea	1605	499	NearestLc	PF00361	PTHR22773,PTHR2277	
18358803	-	upstream	-273	113	NearestLc	PF00361	PTHR22773,PTHR2277	
18358803	-	downstrea	3721	2828	NearestLc	PF00361	PTHR22773,PTHR2277	
18358803	-	overlapEn	493	133	NearestLc	PF00361	PTHR22773,PTHR2277	
18358803	-	downstrea	5373	4267	NearestLc	PF00361	PTHR22773,PTHR2277	
18358803	-	downstrea	4451	3453	NearestLc	PF00361	PTHR22773,PTHR2277	
18358803	-	downstrea	6630	5292	NearestLc	PF00361	PTHR22773,PTHR2277	
18358803	-	downstrea	9349	8121	NearestLc	PF00361	PTHR22773,PTHR2277	
18358803	-	downstrea	2847	1631	NearestLc	PF00361	PTHR22773,PTHR2277	
18358803	-	downstrea	7587	6712	NearestLc	PF00361	PTHR22773,PTHR2277	
18358803	-	downstrea	1605	499	NearestLc	PF00361	PTHR22773,PTHR2277	
18358803	-	upstream	-273	113	NearestLc	PF00361	PTHR22773,PTHR2277	
18358803	-	downstrea	3721	2828	NearestLc	PF00361	PTHR22773,PTHR2277	
18358803	-	overlapEn	493	133	NearestLc	PF00361	PTHR22773,PTHR2277	

18358803	-	downstrea	5373	4267	NearestLc	PF00361	PTHR22773,PTHR2277	
18358803	-	downstrea	4451	3453	NearestLc	PF00361	PTHR22773,PTHR2277	
18358803	-	downstrea	6630	5292	NearestLc	PF00361	PTHR22773,PTHR2277	
18358803	-	downstrea	9349	8121	NearestLc	PF00361	PTHR22773,PTHR2277	
18358803	-	downstrea	2847	1631	NearestLc	PF00361	PTHR22773,PTHR2277	
18358803	-	downstrea	7587	6712	NearestLc	PF00361	PTHR22773,PTHR2277	
18358803	-	downstrea	1605	499	NearestLc	PF00361	PTHR22773,PTHR2277	
18358803	-	upstream	-273	113	NearestLc	PF00361	PTHR22773,PTHR2277	
18358803	-	downstrea	3721	2828	NearestLc	PF00361	PTHR22773,PTHR2277	
18358803	-	overlapEn	493	133	NearestLc	PF00361	PTHR22773,PTHR2277	
18358803	-	downstrea	5373	4267	NearestLc	PF00361	PTHR22773,PTHR2277	
18358803	-	downstrea	4451	3453	NearestLc	PF00361	PTHR22773,PTHR2277	
18358803	-	downstrea	6630	5292	NearestLc	PF00361	PTHR22773,PTHR2277	
18358803	-	downstrea	9349	8121	NearestLc	PF00361	PTHR22773,PTHR2277	
18358803	-	downstrea	2847	1631	NearestLc	PF00361	PTHR22773,PTHR2277	
18358803	-	downstrea	7587	6712	NearestLc	PF00361	PTHR22773,PTHR2277	
18358803	-	downstrea	1605	499	NearestLc	PF00361	PTHR22773,PTHR2277	
18358803	-	upstream	-273	113	NearestLc	PF00361	PTHR22773,PTHR2277	
18358803	-	downstrea	3721	2828	NearestLc	PF00361	PTHR22773,PTHR2277	
18358803	-	overlapEn	493	133	NearestLc	PF00361	PTHR22773,PTHR2277	
18358803	-	downstrea	5373	4267	NearestLc	PF00361	PTHR22773,PTHR2277	
18358803	-	downstrea	4451	3453	NearestLc	PF00361	PTHR22773,PTHR2277	
18358803	-	downstrea	6630	5292	NearestLc	PF00361	PTHR22773,PTHR2277	
18358803	-	downstrea	9349	8121	NearestLc	PF00361	PTHR22773,PTHR2277	
18362639	+	includeFe	904	274	NearestLocation			
18368815	+	overlapSt	-1068	464	NearestLocation			
18368815	+	inside	756	126	NearestLocation			
18368815	+	overlapSt	-1068	464	NearestLocation			
18368815	+	inside	756	126	NearestLocation			
18379352	-	includeFe	19	31	NearestLocation			
18379352	-	upstream	-3332	2749	NearestLocation			
18379352	-	includeFe	19	31	NearestLocation			
18379352	-	upstream	-3332	2749	NearestLocation			
18388134	+	upstream	-1641	1113	NearestLocation			
18388134	+	includeFe	77	276	NearestLocation			
18388134	+	upstream	-2737	2390	NearestLocation			
18390665	+	inside	356	36	NearestLocation			
18390665	+	inside	971	847	NearestLocation			
19564303	+	upstream	-11062	10953	NearestLocation			
20474845	+	inside	3100	248	NearestLc	PF00657	PTHR22835,PTHR2283	
20563528	+	downstrea	7639	5721	NearestLc	PF11955	PTHR31476,PTHR3147	
20563528	+	downstrea	13795	11891	NearestLc	PF11955	PTHR31476,PTHR3147	
21172572	-	upstream	-3188	2965	NearestLc	PF00642	PTHR12506	KOG1677
21548636	-	downstrea	2144	1406	NearestLocation		PTHR36705,PTHR3670	
21822589	-	inside	2016	331	NearestLc	PF03106	PTHR31429,PTHR3142	
21941453	+	upstream	-7426	7309	NearestLc	PF00570,P	PTHR12124,PTHR1212	
22768189	-	overlapSt	23	111	NearestLc	PF00248	PTHR11732,PTHR1173	
23767140	+	inside	123	0	NearestLocation		PTHR36760,PTHR3676	
23767140	+	inside	882	724	NearestLocation		PTHR36760,PTHR3676	
23906186	-	overlapSt	-124	90	NearestLc	PF00010	PTHR12565,PTHR1256	
23906186	-	downstrea	7936	4651	NearestLc	PF00010	PTHR12565,PTHR1256	
23906186	-	downstrea	3838	599	NearestLc	PF00010	PTHR12565,PTHR1256	
23906186	-	upstream	-10076	9976	NearestLc	PF00010	PTHR12565,PTHR1256	
24753375	+	inside	1231	1066	NearestLc	PF00854	PTHR11654,PTHR1165	

25142143	+	inside	1390	1226	NearestLc	PF12171	PTHR10593,PTHR1059
25142143	+	inside	2858	335	NearestLc	PF12171	PTHR10593,PTHR1059
71164	+	upstream	-6846	6736	NearestLc	PF04770	PTHR31948,PTHR3194
71164	+	upstream	-8234	8105	NearestLc	PF04770	PTHR31948,PTHR3194
71164	+	upstream	-6846	6736	NearestLc	PF04770	PTHR31948,PTHR3194
71164	+	upstream	-8234	8105	NearestLc	PF04770	PTHR31948,PTHR3194
657606	+	inside	994	891	NearestLc	PF00067	PTHR24298,PTHR2429
2551810	-	upstream	-226	26	NearestLc	PF04043,P	PTHR31707,PTHR3170
2551810	-	inside	761	595	NearestLc	PF04043,P	PTHR31707,PTHR3170
3022642	-	inside	300	107	NearestLocation		
3375340	+	downstrea	2656	1409	NearestLocation		PTHR33137
3375340	+	downstrea	2656	1409	NearestLocation		PTHR33137
4750656	-	overlapSt	86	142	NearestLocation		
4750656	-	upstream	-5126	5019	NearestLocation		
6418583	+	downstrea	31163	28154	NearestLc	PF02365	PTHR31989,PTHR3198
9388800	+	overlapSt	66	141	NearestLc	PF13424	PTHR26312,PTHR2631
9388800	+	upstream	-634	473	NearestLc	PF13424	PTHR26312,PTHR2631
9388800	+	inside	875	585	NearestLc	PF13424	PTHR26312,PTHR2631
9388800	+	overlapSt	66	141	NearestLc	PF13424	PTHR26312,PTHR2631
9388800	+	upstream	-634	473	NearestLc	PF13424	PTHR26312,PTHR2631
9388800	+	inside	875	585	NearestLc	PF13424	PTHR26312,PTHR2631
9388800	+	overlapSt	66	141	NearestLc	PF13424	PTHR26312,PTHR2631
9388800	+	upstream	-634	473	NearestLc	PF13424	PTHR26312,PTHR2631
9388800	+	inside	875	585	NearestLc	PF13424	PTHR26312,PTHR2631
9390123	+	overlapSt	-44	164	NearestLc	PF00223	PTHR30128,PTHR3012
9390123	+	upstream	-774	659	NearestLc	PF00223	PTHR30128,PTHR3012
9390123	+	overlapSt	-44	164	NearestLc	PF00223	PTHR30128,PTHR3012
9390123	+	upstream	-774	659	NearestLc	PF00223	PTHR30128,PTHR3012
9569026	-	inside	668	137	NearestLc	PF00471	PTHR15238,PTHR1523
9569026	-	inside	668	137	NearestLc	PF00471	PTHR15238,PTHR1523
9569026	-	inside	668	137	NearestLc	PF00471	PTHR15238,PTHR1523
10183823	-	downstrea	1601	751	NearestLc	PF02362	
10537457	+	inside	2030	907	NearestLc	PF01095	PTHR31321,PTHR3132
12730874	+	inside	1231	263	NearestLc	PF01764	PTHR31828 KOG4569
12730874	+	inside	314	90	NearestLc	PF01764	PTHR31828 KOG4569
13046482	-	overlapSt	103	21	NearestLc	PF13893,P	PTHR13069 KOG4176
13046482	-	upstream	-4208	4076	NearestLc	PF13893,P	PTHR13069 KOG4176
13055621	-	inside	341	226	NearestLc	PF03759	PTHR33101,PTHR3310
13107208	+	upstream	-2064	1899	NearestLc	PF06232	PTHR31718,PTHR3171
13176445	+	inside	190	66	NearestLc	PF02463	PTHR19306,PTHR1930
13184572	+	inside	425	33	NearestLc	PF00694	PTHR11670
13700594	+	upstream	-2053	1877	NearestLc	PF02519	PTHR31374,PTHR3137
14056231	-	upstream	-4339	4210	NearestLc	PF00514,P	PTHR24115 KOG0240
15330661	-	inside	239	24	NearestLc	PF02705	PTHR30540,PTHR3054
15381837	-	inside	691	49	NearestLc	PF01370	PTHR32487,PTHR3248
15552231	+	downstrea	2204	1543	NearestLc	PF03953	PTHR11588,PTHR1158
15713106	+	upstream	-757	614	NearestLocation		PTHR34200,PTHR3420
15838530	+	inside	1767	605	NearestLc	PF00657	PTHR22835,PTHR2283
16375217	-	inside	1531	952	NearestLc	PF03195	PTHR31301,PTHR3130
16375217	-	inside	276	151	NearestLc	PF03195	PTHR31301,PTHR3130
16626805	-	inside	961	63	NearestLocation		
16706575	+	upstream	-1394	1277	NearestLocation		
17008343	+	downstrea	7392	1411	NearestLc	PF02163	PTHR13325 KOG2921
17597637	+	upstream	-1178	959	NearestLc	PF02893	PTHR31969,PTHR3196

17597637	+	inside	359	230	NearestLc	PF02893	PTHR31969,PTHR3196	
17950986	+	downstrea	3909	3390	NearestLocation		PTHR36030,PTHR3603	
17950986	+	downstrea	6256	5715	NearestLocation		PTHR36030,PTHR3603	
18372666	+	upstream	-4952	4845	NearestLc	PF00560,P	PTHR27000	KOG1187
18771906	-	upstream	-624	513	NearestLocation			
19535016	-	inside	820	559	NearestLc	PF01849	PTHR21713	KOG2239
20195013	+	inside	4429	3180	NearestLc	PF13191,P	PTHR23155	KOG4658
20195013	+	inside	7359	140	NearestLc	PF13191,P	PTHR23155	KOG4658
20302160	-	downstrea	8760	887	NearestLocation		PTHR33334,PTHR3333	
20302160	-	inside	4520	3149	NearestLocation		PTHR33334,PTHR3333	
20357188	+	upstream	-3029	2919	NearestLc	PF07839	PTHR33923,PTHR3392	
20357188	+	overlapSt	107	160	NearestLc	PF07839	PTHR33923,PTHR3392	
20451939	+	inside	330	10	NearestLc	PF12854,P	PTHR24015,PTHR2401	
20666936	+	upstream	-3993	3877	NearestLc	PF07714,P	PTHR24351	KOG0610
20666936	+	overlapSt	337	12	NearestLc	PF07714,P	PTHR24351	KOG0610
20666936	+	inside	1198	167	NearestLc	PF07714,P	PTHR24351	KOG0610
21516113	-	downstrea	3631	1522	NearestLc	PF00355,P	PTHR21266,PTHR2126	
21527534	+	inside	399	121	NearestLocation			
809677	+	downstrea	2548	1540	NearestLc	PF07714	PTHR27002,PTHR2700	
1297652	+	inside	1059	44	NearestLocation			
2799622	-	upstream	-5654	5509	NearestLocation		PTHR33728,PTHR3372	
2799622	-	upstream	-3566	3444	NearestLocation		PTHR33728,PTHR3372	
3417957	-	upstream	-2935	2823	NearestLc	PF05498	PTHR33136,PTHR3313	
3794363	-	downstrea	2045	1108	NearestLc	PF05078	PTHR31621,PTHR3162	
3870670	-	inside	1154	662	NearestLc	PF02330	PTHR10826,PTHR1082	
4187502	+	overlapSt	47	70	NearestLocation		PTHR33869,PTHR3386	
5250131	+	inside	7920	3299	NearestLc	PF07714,P	PTHR27006	KOG1187
6273742	+	inside	361	238	NearestLc	PF00282	PTHR11999,PTHR1199	
6781216	+	inside	315	146	NearestLocation		PTHR35508,PTHR3550	
8208989	+	inside	275	91	NearestLc	PF00564	PTHR31066,PTHR3106	
8368944	+	downstrea	1407	219	NearestLocation		PTHR34542,PTHR3454	
8368944	+	inside	523	124	NearestLocation		PTHR34542,PTHR3454	
8368944	+	downstrea	1407	219	NearestLocation		PTHR34542,PTHR3454	
8368944	+	inside	523	124	NearestLocation		PTHR34542,PTHR3454	
9512253	+	upstream	-7646	7330	NearestLocation		PTHR34191,PTHR3419	
9512253	+	upstream	-7646	7330	NearestLocation		PTHR34191,PTHR3419	
10321954	+	inside	1246	1119	NearestLc	PF13519,P	PTHR12695,PTHR1269	
11932387	-	inside	347	193	NearestLocation		PTHR36391,PTHR3639	
12468284	-	downstrea	46495	44652	NearestLc	PF00249	PTHR10641	KOG0048
12468284	-	upstream	-4352	4125	NearestLc	PF00249	PTHR10641	KOG0048
12594595	+	downstrea	79150	74805	NearestLc	PF07891	PTHR10759,PTHR1075	
13125335	-	upstream	-75565	75352	NearestLocation		PTHR33078,PTHR3307	
14352155	+	downstrea	10106	6862	NearestLc	PF01263	PTHR10091	KOG1604
14352155	+	upstream	-6499	6398	NearestLc	PF01263	PTHR10091	KOG1604
14352155	+	upstream	-8574	8452	NearestLc	PF01263	PTHR10091	KOG1604
14352155	+	downstrea	18590	15431	NearestLc	PF01263	PTHR10091	KOG1604
14352155	+	downstrea	14531	11387	NearestLc	PF01263	PTHR10091	KOG1604
14352155	+	downstrea	9117	5931	NearestLc	PF01263	PTHR10091	KOG1604
14416706	+	downstrea	44189	41379	NearestLc	PF02780,P	PTHR11624,PTHR1162	
14906274	+	downstrea	6162	2928	NearestLc	PF01920	PTHR21431	KOG3478
16136863	+	downstrea	3989	1858	NearestLc	PF01453,P	PTHR27002,PTHR2700	
16136863	+	downstrea	13503	11388	NearestLc	PF01453,P	PTHR27002,PTHR2700	
16763104	-	overlapEn	1101	104	NearestLc	PF00403	PTHR22814	KOG1603
16763104	-	overlapSt	-76	251	NearestLc	PF00403	PTHR22814	KOG1603

17751087	+	inside	2601	96	NearestLc	PF12695	PTHR10992	KOG1454
18176691	+	upstream	-8069	7953	NearestLc	PF00494	PTHR11626,	PTHR1162
18520295	+	inside	251	10	NearestLc	PF02519	PTHR31374,	PTHR3137
18520295	+	inside	251	10	NearestLc	PF02519	PTHR31374,	PTHR3137
18673602	-	upstream	-4460	4147	NearestLc	PF00230	PTHR19139	KOG0223
18673602	-	upstream	-4460	4147	NearestLc	PF00230	PTHR19139	KOG0223
19047726	+	inside	2488	679	NearestLc	PF01425	PTHR11895	KOG1211
19652964	-	upstream	-1694	1573	NearestLc	PF07714	PTHR23257	KOG0192
19652964	-	inside	2431	2329	NearestLc	PF07714	PTHR23257	KOG0192
19652964	-	downstrea	9296	2261	NearestLc	PF07714	PTHR23257	KOG0192
20207004	-	inside	413	84	NearestLc	PF07714, P	PTHR24351,	PTHR2435
20355530	+	downstrea	2399	951	NearestLocation			
20857045	+	downstrea	3637	2096	NearestLc	PF00069	PTHR24361	KOG0198
20888083	+	inside	2003	42	NearestLocation		PTHR33269,	PTHR3326
21401342	+	inside	3551	616	NearestLc	PF02167	PTHR10266,	PTHR1026
22884394	-	inside	596	323	NearestLc	PF01106	PTHR31942	KOG2358
24169908	-	upstream	-1072	774	NearestLc	PF10996, P	PTHR11203	KOG1138
350058	+	downstrea	2698	313	NearestLc	PF02781	PTHR23429,	PTHR2342
1856108	-	inside	778	639	NearestLc	PF00657	PTHR22835,	PTHR2283
1856108	-	overlapEn	1796	107	NearestLc	PF00657	PTHR22835,	PTHR2283
1856108	-	overlapSt	93	144	NearestLc	PF00657	PTHR22835,	PTHR2283
1880313	-	inside	329	96	NearestLc	PF01553	PTHR12497	KOG2847
4182149	+	downstrea	2869	1845	NearestLc	PF00704	PTHR11177,	PTHR1117
4182149	+	downstrea	2869	1845	NearestLc	PF00704	PTHR11177,	PTHR1117
4536336	+	overlapSt	59	64	NearestLc	PF10251	PTHR16318	KOG3402
5595844	-	upstream	-2948	2834	NearestLc	PF13414	PTHR22904,	PTHR2290
5706720	-	upstream	-957	818	NearestLc	PF00847	PTHR31657,	PTHR3165
6352430	+	inside	334	82	NearestLc	PF01521	PTHR10072	KOG1120
6478912	-	inside	262	112	NearestLc	PF03151	PTHR11132	KOG1441
6901736	+	inside	1776	1271	NearestLc	PF00574	PTHR10381	KOG0840
7173494	+	upstream	-4655	4534	NearestLc	PF01397, P	PTHR31225,	PTHR3122
7385275	+	downstrea	10112	8438	NearestLc	PF06203	PTHR31717,	PTHR3171
8410117	+	inside	579	194	NearestLc	PF05383	PTHR22792,	PTHR2279
11617068	+	downstrea	4889	1820	NearestLocation		PTHR13593,	PTHR1359
11709426	-	overlapSt	185	5	NearestLocation		PTHR13593,	PTHR1359
12271223	+	inside	412	48	NearestLc	PF00067	PTHR24298,	PTHR2429
12730556	+	downstrea	18998	13366	NearestLc	PF07714	PTHR24054,	PTHR2405
12730556	+	inside	4586	817	NearestLc	PF07714	PTHR24054,	PTHR2405
12773153	+	inside	1170	728	NearestLc	PF00583	PTHR23091,	PTHR2309
13493694	+	inside	230	54	NearestLc	PF01039	PTHR22855,	PTHR2285
13496246	-	upstream	-11120	11019	NearestLocation		PTHR34724,	PTHR3472
13496246	-	upstream	-14752	14641	NearestLocation		PTHR34724,	PTHR3472
13496246	-	upstream	-14010	13908	NearestLocation		PTHR34724,	PTHR3472
13496246	-	upstream	-8480	8268	NearestLocation		PTHR34724,	PTHR3472
13496246	-	upstream	-11120	11019	NearestLocation		PTHR34724,	PTHR3472
13496246	-	upstream	-14752	14641	NearestLocation		PTHR34724,	PTHR3472
13496246	-	upstream	-14010	13908	NearestLocation		PTHR34724,	PTHR3472
13496246	-	upstream	-8480	8268	NearestLocation		PTHR34724,	PTHR3472
13496246	-	upstream	-11120	11019	NearestLocation		PTHR34724,	PTHR3472
13496246	-	upstream	-14752	14641	NearestLocation		PTHR34724,	PTHR3472
13496246	-	upstream	-14010	13908	NearestLocation		PTHR34724,	PTHR3472
13496246	-	upstream	-8480	8268	NearestLocation		PTHR34724,	PTHR3472
13549048	+	upstream	-1373	1096	NearestLocation			
13549048	+	downstrea	575	66	NearestLocation			

14533505	+	upstream	-7887	7786	NearestLocation			
14815682	-	inside	1272	478	NearestLc	PF00181	PTHR13691,PTHR1369	
14826149	-	upstream	-90475	90371	NearestLc	PF00137	PTHR10031	
14826149	-	inside	118	16	NearestLc	PF00137	PTHR10031	
16510622	+	upstream	-10347	10183	NearestLc	PF02183,P	PTHR24326	KOG0483
17715725	-	upstream	-8369	8256	NearestLc	PF04570	PTHR33059,PTHR3305	
17715725	-	upstream	-2579	2464	NearestLc	PF04570	PTHR33059,PTHR3305	
18578651	-	inside	633	164	NearestLc	PF02362,P	PTHR31384,PTHR3138	
19150115	+	overlapSt	-191	17	NearestLc	PF02493	PTHR23084,PTHR2308	
19334608	+	inside	237	89	NearestLc	PF13414	PTHR22904	KOG0553
19621347	-	upstream	-5114	4941	NearestLc	PF00168,P	PTHR23315,PTHR2331	
20550472	-	downstrea	5409	4296	NearestLc	PF04720	PTHR31579,PTHR3157	
21438717	-	inside	536	259	NearestLc	PF00156	PTHR22573	KOG3367
21882935	+	downstrea	2628	781	NearestLc	PF05678		
21983177	-	inside	655	190	NearestLocation		PTHR11959	KOG0638
22396557	-	inside	1400	124	NearestLc	PF01734	PTHR32241	KOG0513
22396557	-	inside	193	65	NearestLc	PF01734	PTHR32241	KOG0513
22986419	-	overlapSt	193	33	NearestLocation		PTHR22773,PTHR2277	
23036682	+	inside	661	415	NearestLc	PF00010	PTHR16223,PTHR1622	
23572383	+	downstrea	5682	3597	NearestLc	PF04852	PTHR31165,PTHR3116	
23604582	-	upstream	-7955	7840	NearestLc	PF02365	PTHR31744,PTHR3174	
23604582	-	inside	1584	1459	NearestLc	PF02365	PTHR31744,PTHR3174	
23671043	+	overlapSt	46	144	NearestLc	PF00433,P	PTHR24356,PTHR2435	
24082503	-	upstream	-4046	3905	NearestLc	PF01419	PTHR23244,PTHR2324	
24082503	-	upstream	-1279	1119	NearestLc	PF01419	PTHR23244,PTHR2324	
24205400	-	downstrea	2652	15	NearestLc	PF05142	PTHR31604,PTHR3160	
24205400	-	upstream	-724	585	NearestLc	PF05142	PTHR31604,PTHR3160	
24285817	-	inside	385	203	NearestLc	PF00137	PTHR10263	KOG0232
25231607	-	inside	309	123	NearestLc	PF13414,P	PTHR27001	KOG1187
25231607	-	upstream	-4078	3957	NearestLc	PF13414,P	PTHR27001	KOG1187
588339	+	upstream	-3038	2931	NearestLc	PF14547	PTHR31731,PTHR3173	
2705921	-	inside	1142	1026	NearestLc	PF01493,P	PTHR11938,PTHR1193	
3653326	-	upstream	-574	459	NearestLc	PF07065	PTHR15323,PTHR1532	
3653326	-	downstrea	1059	504	NearestLc	PF07065	PTHR15323,PTHR1532	
3666125	+	inside	183	74	NearestLc	PF05193,P	PTHR11851,PTHR1185	
3674265	-	inside	1057	708	NearestLocation		PTHR34799	
4152565	-	inside	3675	168	NearestLc	PF10183	PTHR31339	
4152565	-	overlapSt	139	41	NearestLc	PF10183	PTHR31339	
4615026	-	inside	309	138	NearestLc	PF12796,P	PTHR24177,PTHR2417	
4615026	-	inside	3288	442	NearestLc	PF12796,P	PTHR24177,PTHR2417	
4615026	-	inside	3704	82	NearestLc	PF12796,P	PTHR24177,PTHR2417	
5006017	+	upstream	-3019	2905	NearestLc	PF00400	PTHR22847	KOG0266
5400972	-	overlapSt	96	6	NearestLc	PF13855	PTHR23155	KOG0617
5458225	+	inside	658	367	NearestLc	PF02096	PTHR12428	KOG1239
6512593	+	upstream	-467	366	NearestLocation			
6518158	-	upstream	-4722	4549	NearestLc	PF13921	PTHR10641	KOG0048
6541462	-	upstream	-8929	8815	NearestLc	PF03330,P	PTHR31867,PTHR3186	
6541462	-	upstream	-1946	1682	NearestLc	PF03330,P	PTHR31867,PTHR3186	
7748908	-	upstream	-5196	5087	NearestLc	PF03171,P	PTHR10209	KOG0143
7748908	-	inside	470	240	NearestLc	PF03171,P	PTHR10209	KOG0143
8703532	-	upstream	-2392	2255	NearestLc	PF11891	PTHR31620,PTHR3162	
8703532	-	inside	779	447	NearestLc	PF11891	PTHR31620,PTHR3162	
8703532	-	inside	1610	141	NearestLc	PF11891	PTHR31620,PTHR3162	
9341032	-	inside	2384	2076	NearestLc	PF00585,P	PTHR10314	KOG1250

9447868	+	upstream	-3815	3683	NearestLc	PF04640	PTHR31065,PTHR3106	
9479638	+	inside	186	45	NearestLc	PF11894	PTHR31344,PTHR3134	
10470993	+	upstream	-1520	1319	NearestLc	PF07714	PTHR27003,PTHR2700	
11151907	+	upstream	-9216	9091	NearestLocation			
12136248	+	overlapEn	276	50	NearestLocation			
12899780	-	overlapSt	108	29	NearestLc	PF00010	PTHR12565,PTHR1256	
12899780	-	downstrea	26428	24184	NearestLc	PF00010	PTHR12565,PTHR1256	
12899780	-	inside	1896	93	NearestLc	PF00010	PTHR12565,PTHR1256	
12956974	-	upstream	-980	860	NearestLc	PF13847	PTHR11006	
15217572	-	inside	1052	831	NearestLc	PF09118,P	PTHR32208,PTHR3220	
15250270	-	upstream	-28286	28180	NearestLocation		PTHR33388,PTHR3338	
16330536	+	downstrea	8499	7911	NearestLocation		PTHR31934,PTHR3193	
16565141	+	inside	5111	4912	NearestLc	PF05183	PTHR23079	KOG0988
17626867	+	downstrea	23665	22996	NearestLocation			
18636292	-	downstrea	3440	1672	NearestLc	PF02469	PTHR32382,PTHR3238	
19006954	-	upstream	-12034	11933	NearestLocation		PTHR33124,PTHR3312	
19006954	-	upstream	-14108	14006	NearestLocation		PTHR33124,PTHR3312	
19006954	-	upstream	-13316	13166	NearestLocation		PTHR33124,PTHR3312	
19393395	-	inside	380	233	NearestLocation			
20066055	+	upstream	-21668	21544	NearestLc	PF14531,P	PTHR24347	KOG0583
20066055	+	inside	1332	670	NearestLc	PF14531,P	PTHR24347	KOG0583
20912145	-	inside	274	24	NearestLc	PF03145	PTHR10315	KOG3002
20912145	-	upstream	-5515	5354	NearestLc	PF03145	PTHR10315	KOG3002
21209889	+	overlapEn	335	72	NearestLocation			
21746664	-	downstrea	5651	3820	NearestLc	PF04564	PTHR22849,PTHR2284	
22069887	+	upstream	-12144	12033	NearestLc	PF03641	PTHR31223,PTHR3122	
22069887	+	upstream	-11010	10855	NearestLc	PF03641	PTHR31223,PTHR3122	
22069887	+	overlapSt	172	179	NearestLc	PF03641	PTHR31223,PTHR3122	
22660766	+	inside	570	61	NearestLocation		PTHR34120,PTHR3412	
23754666	+	upstream	-12340	12156	NearestLc	PF04851,P	PTHR24031,PTHR2403	
24417659	+	upstream	-3665	3536	NearestLc	PF04545,P	PTHR30603,PTHR3060	
24674234	-	inside	2098	1905	NearestLc	PF00046,P	PTHR24326	KOG0483
24674234	-	upstream	-6284	6156	NearestLc	PF00046,P	PTHR24326	KOG0483
24674234	-	upstream	-5634	5507	NearestLc	PF00046,P	PTHR24326	KOG0483
26745769	-	downstrea	1840	1079	NearestLc	PF05577	PTHR11010,PTHR1101	
1005406	-	upstream	-1133	991	NearestLc	PF01625	PTHR10173	KOG1635
1442648	-	downstrea	773	237	NearestLocation			
1442648	-	upstream	-3394	3283	NearestLocation			
3254895	-	inside	2401	34	NearestLc	PF01734	PTHR32176	KOG0513
3570355	-	upstream	-8110	8003	NearestLc	PF00685	PTHR32175,PTHR3217	
3781782	-	inside	2199	1050	NearestLc	PF00447	PTHR10015,PTHR1001	
4760930	-	upstream	-10403	10160	NearestLc	PF00067	PTHR24298	KOG0156
4760930	-	overlapSt	50	82	NearestLc	PF00067	PTHR24298	KOG0156
5118175	+	upstream	-11718	11603	NearestLc	PF00481	PTHR13832	KOG0700
5662744	+	downstrea	15000	13161	NearestLocation			
5981095	+	overlapSt	112	210	NearestLc	PF13414	PTHR36326,PTHR3632	
7251317	+	upstream	-3381	3273	NearestLocation			
7251317	+	upstream	-5926	5818	NearestLocation			
7251317	+	upstream	-3381	3273	NearestLocation			
7251317	+	upstream	-5926	5818	NearestLocation			
7975279	+	downstrea	4443	3689	NearestLocation			
8149905	+	upstream	-11790	11555	NearestLc	PF03094	PTHR31942,PTHR3194	
8210013	-	inside	749	479	NearestLc	PF13855	PTHR23155	KOG0617
8780271	+	downstrea	3829	390	NearestLc	PF00249	PTHR10641	KOG0048

8780271	+	upstream	-2896	2789	NearestLc	PF00249	PTHR10641	KOG0048
10549296	-	inside	613	485	NearestLc	PF02446	PTHR32438,PTHR3243	
11194068	-	inside	237	30	NearestLc	PF13639	PTHR14155,PTHR1415	
11688658	+	downstrea	4738	1950	NearestLocation		PTHR33924,PTHR3392	
12681607	-	overlapSt	265	24	NearestLc	PF13450	PTHR15944	
13007361	+	overlapSt	-53	490	NearestLc	PF12708	PTHR31339,PTHR3133	
13313045	-	inside	310	108	NearestLc	PF00856,P	PTHR10615,PTHR1061	
14212062	-	inside	1332	1223	NearestLc	PF00069	PTHR27009	KOG1187
14914390	+	inside	195	76	NearestLc	PF00249	PTHR31312,PTHR3131	
15322624	+	inside	4110	3987	NearestLc	PF13676,P	PTHR11017,PTHR1101	
15322624	+	inside	4110	3987	NearestLc	PF13676,P	PTHR11017,PTHR1101	
15361898	-	upstream	-846	606	NearestLc	PF00107,P	PTHR11695,PTHR1169	
380409	+	inside	264	54	NearestLocation			
1518765	-	downstrea	1605	791	NearestLocation			
1783753	-	downstrea	4843	3102	NearestLc	PF07795	PTHR33431,PTHR3343	
1783753	-	upstream	-2373	2265	NearestLc	PF07795	PTHR33431,PTHR3343	
1989518	+	downstrea	5923	1599	NearestLc	PF00025	PTHR24073	KOG0092
2812920	-	upstream	-2194	2072	NearestLocation		PTHR14154,PTHR1415	
3566978	+	downstrea	2709	366	NearestLc	PF00481	PTHR13832	KOG0698
3614813	-	inside	299	140	NearestLc	PF00076,P	PTHR10352	KOG0122
3614813	-	upstream	-1350	1235	NearestLc	PF00076,P	PTHR10352	KOG0122
3697846	-	downstrea	2152	29	NearestLc	PF00025	PTHR24073	KOG0087
3779286	-	upstream	-925	734	NearestLc	PF04788	PTHR31300,PTHR3130	
3779286	-	upstream	-2426	2273	NearestLc	PF04788	PTHR31300,PTHR3130	
4478817	+	inside	422	189	NearestLc	PF12222	PTHR31104,PTHR3110	
4514673	+	upstream	-5358	5243	NearestLc	PF03195	PTHR31301,PTHR3130	
5216145	-	inside	1554	1421	NearestLc	PF00478	PTHR11911,PTHR1191	
6065835	-	inside	1306	205	NearestLc	PF05019	PTHR12922	KOG3244
6065835	-	inside	233	87	NearestLc	PF05019	PTHR12922	KOG3244
6169119	+	inside	1017	842	NearestLc	PF01486,P	PTHR11945	KOG0014
6169119	+	inside	231	113	NearestLc	PF01486,P	PTHR11945	KOG0014
6628702	-	inside	974	865	NearestLc	PF00179	PTHR24067	KOG0419,K
6628702	-	inside	335	210	NearestLc	PF00179	PTHR24067	KOG0419,K
7843839	-	upstream	-1227	1111	NearestLocation		PTHR37184	
8012130	+	upstream	-188	77	NearestLocation			
8012130	+	downstrea	652	61	NearestLocation			
8018741	-	upstream	-6753	6644	NearestLc	PF13912		
8632295	-	inside	761	304	NearestLc	PF00407	PTHR31338,PTHR3133	
8632295	-	upstream	-1195	1081	NearestLc	PF00407	PTHR31338,PTHR3133	
9862692	+	downstrea	5847	3652	NearestLc	PF14009	PTHR33413,PTHR3341	
9862692	+	upstream	-5860	5713	NearestLc	PF14009	PTHR33413,PTHR3341	
11092256	+	upstream	-271	149	NearestLocation		PTHR33592,PTHR3359	
11930495	+	upstream	-1438	1270	NearestLc	PF02736,P	PTHR13140,PTHR1314	
12102875	+	upstream	-207	82	NearestLc	PF04564	PTHR22849,PTHR2284	
12102875	+	upstream	-4456	4262	NearestLc	PF04564	PTHR22849,PTHR2284	
12405504	+	inside	243	54	NearestLc	PF02096	PTHR12428	KOG1239
13022935	-	inside	1739	1265	NearestLc	PF00295	PTHR31375,PTHR3137	
14571002	+	inside	1103	838	NearestLc	PF00230	PTHR19139	KOG0223
15127815	-	overlapSt	437	26	NearestLocation		PTHR34451,PTHR3445	
15744149	-	inside	503	89	NearestLc	PF05637	PTHR31311	KOG4748
15744149	-	downstrea	11616	9754	NearestLc	PF05637	PTHR31311	KOG4748
17240740	+	upstream	-4564	4357	NearestLc	PF00462	PTHR10168	KOG1752
17240740	+	upstream	-817	673	NearestLc	PF00462	PTHR10168	KOG1752
17240740	+	upstream	-4564	4357	NearestLc	PF00462	PTHR10168	KOG1752

17240740	+	upstream	-817	673	NearestLc	PF00462	PTHR10168	KOG1752
17484307	-	inside	503	203	NearestLc	PF00642	PTHR12506	KOG1677
18792449	+	inside	1295	616	NearestLc	PF12348	PTHR21567	KOG2933
18792449	+	upstream	-10435	10096	NearestLc	PF12348	PTHR21567	KOG2933
191530	+	upstream	-2605	2500	NearestLocation			
240371	+	downstrea	12070	10398	NearestLocation		PTHR33233,PTHR3323	
430507	-	downstrea	16319	14607	NearestLc	PF14111	PTHR31286,PTHR3128	
430507	-	downstrea	16319	14607	NearestLc	PF14111	PTHR31286,PTHR3128	
989641	+	upstream	-498	219	NearestLocation			
989641	+	upstream	-1261	1087	NearestLocation			
989641	+	overlapEn	291	19	NearestLocation			
989641	+	downstrea	1274	738	NearestLocation			
989641	+	upstream	-498	219	NearestLocation			
989641	+	upstream	-1261	1087	NearestLocation			
989641	+	overlapEn	291	19	NearestLocation			
989641	+	downstrea	1274	738	NearestLocation			
991793	-	overlapEn	353	67	NearestLc	PF13631	PTHR19271,PTHR1927	
1394665	+	upstream	-25620	25476	NearestLc	PF00403	PTHR22814	KOG1603
1394665	+	upstream	-6706	6586	NearestLc	PF00403	PTHR22814	KOG1603
1394665	+	inside	1212	955	NearestLc	PF00403	PTHR22814	KOG1603
2730230	-	downstrea	1477	1219	NearestLc	PF01737	PTHR34971	
2730230	-	downstrea	2168	1822	NearestLc	PF01737	PTHR34971	
2730230	-	downstrea	2738	2432	NearestLc	PF01737	PTHR34971	
2730230	-	includeFe	98	15	NearestLc	PF01737	PTHR34971	
2730230	-	downstrea	1477	1219	NearestLc	PF01737	PTHR34971	
2730230	-	downstrea	2168	1822	NearestLc	PF01737	PTHR34971	
2730230	-	downstrea	2738	2432	NearestLc	PF01737	PTHR34971	
2730230	-	includeFe	98	15	NearestLc	PF01737	PTHR34971	
4116374	-	upstream	-2198	2006	NearestLc	PF00481	PTHR13832	KOG0700
5384224	+	upstream	-1454	1306	NearestLc	PF04227		
5483711	-	inside	415	148	NearestLc	PF00349,P	PTHR19443,PTHR1944	
5508800	+	upstream	-3653	3503	NearestLc	PF03435	PTHR12286,PTHR1228	
5857717	+	overlapSt	155	9	NearestLc	PF12697	PTHR10992	KOG1454
6593008	-	inside	1264	1077	NearestLc	PF00067	PTHR24298,PTHR2429	
6861355	-	inside	366	97	NearestLc	PF08627	PTHR31326,PTHR3132	
7143769	-	overlapSt	87	195	NearestLocation			
7520372	-	upstream	-5512	5404	NearestLc	PF00117	PTHR11922	KOG3179
8626665	-	downstrea	5591	3723	NearestLc	PF00201	PTHR11926	KOG1192
10212912	-	upstream	-1030	913	NearestLc	PF13920,P	PTHR24115,PTHR2411	
10239156	-	upstream	-914	800	NearestLocation		PTHR11206,PTHR1120	
10436054	+	overlapSt	91	34	NearestLc	PF02519	PTHR31374,PTHR3137	
10436054	+	overlapEn	878	182	NearestLc	PF02519	PTHR31374,PTHR3137	
11367122	-	inside	328	50	NearestLc	PF10250	PTHR31818,PTHR3181	
11410604	+	upstream	-3690	3513	NearestLocation		PTHR10593,PTHR1059	
11788467	-	upstream	-3233	3126	NearestLc	PF13632	PTHR32044,PTHR3204	
11788467	-	inside	1871	1765	NearestLc	PF13632	PTHR32044,PTHR3204	
11983282	-	upstream	-347	235	NearestLc	PF00011	PTHR11527,PTHR1152	
12030673	+	inside	1455	1215	NearestLc	PF01915,P	PTHR30620,PTHR3062	
12165578	-	inside	333	106	NearestLc	PF03454,P	PTHR10192	KOG2371
12947133	+	downstrea	5176	1134	NearestLc	PF00850	PTHR10625,PTHR1062	
681849	-	inside	1111	274	NearestLocation		PTHR31346,PTHR3134	
2840631	-	upstream	-9207	9083	NearestLc	PF00226,P	PTHR24078,PTHR2407	
4161547	+	downstrea	57499	51650	NearestLc	PF00917,P	PTHR24411	KOG1987
4423356	-	upstream	-3721	3567	NearestLocation			

4675182	-	upstream	-77186	77079	NearestLc	PF00069	PTHR22983,PTHR2298
4675182	-	inside	4732	4624	NearestLc	PF00069	PTHR22983,PTHR2298
4675182	-	upstream	-77186	77079	NearestLc	PF00069	PTHR22983,PTHR2298
4675182	-	inside	4732	4624	NearestLc	PF00069	PTHR22983,PTHR2298
4675182	-	upstream	-77186	77079	NearestLc	PF00069	PTHR22983,PTHR2298
4675182	-	inside	4732	4624	NearestLc	PF00069	PTHR22983,PTHR2298
5230211	+	inside	176	30	NearestLc	PF00421	PTHR33180,PTHR3318
5671886	+	upstream	-53121	52994	NearestLocation		PTHR33976,PTHR3397
5748499	+	inside	257	22	NearestLocation		
7331368	+	inside	1100	815	NearestLc	PF01985	PTHR31846,PTHR3184
8333763	+	inside	383	27	NearestLocation		PTHR33163,PTHR3316
8333763	+	downstrea	1313	433	NearestLocation		PTHR33163,PTHR3316
8340580	+	upstream	-767	659	NearestLc	PF13920	PTHR15315 KOG4172
8340580	+	upstream	-1460	1348	NearestLc	PF13920	PTHR15315 KOG4172
10016283	+	inside	612	366	NearestLc	PF00022	PTHR11937 KOG0797
10143131	-	overlapSt	549	67	NearestLc	PF00498	PTHR23308,PTHR2330
11304654	-	downstrea	2652	759	NearestLc	PF02531	PTHR31982,PTHR3198
11755290	+	downstrea	11459	3221	NearestLc	PF01693,P	PTHR33033,PTHR3303
11848430	-	upstream	-748	618	NearestLc	PF00892	PTHR31218,PTHR3121
12507193	+	inside	3797	168	NearestLc	PF08282,P	PTHR10788,PTHR1078
12507193	+	inside	538	346	NearestLc	PF08282,P	PTHR10788,PTHR1078
13120417	+	upstream	-9314	9198	NearestLc	PF02183,P	PTHR24326 KOG0483
13137175	+	inside	1485	1261	NearestLc	PF03547	PTHR31752,PTHR3175
13137175	+	inside	568	207	NearestLc	PF03547	PTHR31752,PTHR3175
13197170	+	upstream	-1804	1635	NearestLc	PF02749,P	PTHR32179
13644147	-	overlapSt	103	5	NearestLc	PF01569	PTHR10165 KOG3030
13735324	+	downstrea	3167	1119	NearestLc	PF00117	PTHR11922,PTHR1192
14803969	-	downstrea	2905	844	NearestLocation		PTHR10997,PTHR1099
15939837	+	inside	1045	294	NearestLc	PF07876	PTHR33178
16024613	-	upstream	-351	182	NearestLc	PF04146	PTHR12357 KOG1901
17012320	+	upstream	-1579	1389	NearestLc	PF03330,P	PTHR31867
17100780	-	inside	298	123	NearestLc	PF14432,P	PTHR24015,PTHR2401
18069486	+	inside	2567	121	NearestLc	PF08263,P	PTHR27008 KOG1187
18457379	+	inside	284	73	NearestLc	PF13540,P	PTHR27003 KOG1187
18927914	-	upstream	-7136	6916	NearestLocation		PTHR33356,PTHR3335
19162306	+	inside	633	375	NearestLc	PF03141	PTHR10108,PTHR1010
19406380	-	upstream	-5115	4896	NearestLocation		PTHR21717,PTHR2171
19769106	+	inside	3807	3520	NearestLc	PF01842	PTHR31096,PTHR3109
20365631	+	upstream	-1312	1153	NearestLc	PF00076	PTHR24012 KOG0149
20365631	+	inside	2990	86	NearestLc	PF00076	PTHR24012 KOG0149
20365631	+	inside	1146	1005	NearestLc	PF00076	PTHR24012 KOG0149
20750870	-	downstrea	6672	3053	NearestLc	PF00320	PTHR10071,PTHR1007
20750870	-	upstream	-4478	4306	NearestLc	PF00320	PTHR10071,PTHR1007
20856785	+	upstream	-191	39	NearestLc	PF02375,P	PTHR10694,PTHR1069
20956297	+	inside	295	183	NearestLocation		PTHR36619,PTHR3661
20956297	+	downstrea	4747	3816	NearestLocation		PTHR36619,PTHR3661
281529	-	inside	1319	922	NearestLc	PF14531,P	PTHR27002 KOG1187
1294248	-	inside	298	16	NearestLc	PF00365	PTHR13697,PTHR1369
6675394	+	downstrea	1151	509	NearestLocation		
7194553	-	overlapSt	309	126	NearestLc	PF05758	PTHR33163,PTHR3316
7204270	-	inside	243	128	NearestLc	PF00032	PTHR19271,PTHR1927
7204270	-	inside	243	128	NearestLc	PF00032	PTHR19271,PTHR1927
7207063	-	overlapEn	229	100	NearestLc	PF00737	PTHR34469
7217613	+	inside	914	407	NearestLocation		PTHR33078,PTHR3307

7217613	+	inside	914	407	NearestLocation		PTHR33078,PTHR3307	
7232353	-	inside	147	40	NearestLc	PF00361	PTHR22773	KOG4668
7232353	-	inside	667	562	NearestLc	PF00361	PTHR22773	KOG4668
8008521	-	downstrea	3988	1371	NearestLc	PF03188	PTHR23130,PTHR2313	
8297303	+	upstream	-6625	6472	NearestLc	PF00013	PTHR10288	KOG2190
8749299	-	upstream	-1015	903	NearestLc	PF10557,P	PTHR11932,PTHR1193	
11654381	-	inside	3185	3076	NearestLc	PF12333	PTHR16056	KOG2149
11654381	-	inside	3185	3076	NearestLc	PF12333	PTHR16056	KOG2149
12064255	+	inside	1134	4	NearestLocation			
12868517	-	inside	249	129	NearestLocation		PTHR27004,PTHR2700	
13861201	-	upstream	-182	77	NearestLc	PF01405	PTHR36411,PTHR3641	
14623025	+	inside	377	175	NearestLocation		PTHR34689,PTHR3468	
15719855	+	overlapEn	473	43	NearestLocation			
16064549	+	upstream	-946	840	NearestLc	PF03092	PTHR31585,PTHR3158	
16380645	-	downstrea	2443	565	NearestLc	PF13920	PTHR10044	KOG1100
16786493	+	inside	408	137	NearestLocation			
17265372	+	upstream	-10091	9974	NearestLc	PF02833	PTHR12112	KOG4129
18051851	+	inside	417	48	NearestLc	PF08031,P	PTHR32448	
275360	+	downstrea	5093	4119	NearestLc	PF00069	PTHR27009,PTHR2700	
880332	+	upstream	-423	317	NearestLc	PF16321	PTHR33231,PTHR3323	
1701858	-	inside	1008	596	NearestLc	PF00856,P	PTHR10615,PTHR1061	
1791558	-	overlapSt	-102	49	NearestLc	PF00249	PTHR10641,PTHR1064	
1878215	-	downstrea	4061	1142	NearestLc	PF08263,P	PTHR27004	KOG0472
1878215	-	inside	2361	338	NearestLc	PF08263,P	PTHR27004	KOG0472
2056456	+	inside	380	20	NearestLc	PF00069	PTHR24349	KOG0032
3631119	+	downstrea	4203	2122	NearestLc	PF06217	PTHR31421,PTHR3142	
3763917	-	inside	4772	2403	NearestLc	PF12695	PTHR11614	KOG1455
3780497	-	upstream	-5005	4899	NearestLc	PF07911	PTHR33108,PTHR3310	
4992404	-	inside	553	193	NearestLocation		PTHR24014	KOG1971
5411011	+	inside	200	12	NearestLc	PF00564	PTHR31066,PTHR3106	
6960376	+	inside	11500	8615	NearestLc	PF00319,P	PTHR11945	KOG0014
6960376	+	inside	19960	12	NearestLc	PF00319,P	PTHR11945	KOG0014
7541856	-	downstrea	54078	52992	NearestLc	PF01585	PTHR13288,PTHR1328	
8472222	+	inside	206	50	NearestLc	PF00400,P	PTHR19932,PTHR1993	
8472222	+	inside	1603	1453	NearestLc	PF00400,P	PTHR19932,PTHR1993	
8472222	+	upstream	-23214	23110	NearestLc	PF00400,P	PTHR19932,PTHR1993	
9826283	+	inside	1817	1126	NearestLc	PF00010	PTHR12565,PTHR1256	
9830346	-	inside	189	87	NearestLc	PF01812	PTHR23407	KOG3093
9858922	+	inside	318	103	NearestLc	PF00149	PTHR11668,PTHR1166	
10416356	-	upstream	-11510	11320	NearestLc	PF02358,P	PTHR10788,PTHR1078	
10416356	-	upstream	-4872	4728	NearestLc	PF02358,P	PTHR10788,PTHR1078	
10799160	+	upstream	-19076	18959	NearestLc	PF02701	PTHR31992,PTHR3199	
11139660	+	downstrea	6804	4747	NearestLc	PF00249	PTHR10641	KOG0048
11359696	+	upstream	-8638	8408	NearestLc	PF14571,P	PTHR31875,PTHR3187	
11684817	+	inside	1704	199	NearestLc	PF00067	PTHR24298,PTHR2429	
12017020	+	inside	3230	1887	NearestLc	PF01399,P	PTHR14145,PTHR1414	
12313557	-	inside	415	6	NearestLc	PF03081	PTHR12542,PTHR1254	
12357365	-	upstream	-2693	2561	NearestLc	PF08238,P	PTHR12298,PTHR1229	
12357365	-	overlapSt	-55	145	NearestLc	PF08238,P	PTHR12298,PTHR1229	
12375940	-	inside	1126	1006	NearestLc	PF00383	PTHR11086	KOG3127
12375940	-	inside	1795	1670	NearestLc	PF00383	PTHR11086	KOG3127
12557943	+	inside	247	13	NearestLocation		PTHR13061	KOG3121
12869907	-	upstream	-2867	2752	NearestLc	PF01471,P	PTHR10201,PTHR1020	
12896793	-	downstrea	3250	1116	NearestLc	PF00010	PTHR12565,PTHR1256	

14119973	+	downstrea	3290	149	NearestLocation			
14206880	+	inside	2181	2076	NearestLc	PF00227	PTHR11599,PTHR1159	
14736753	+	inside	243	86	NearestLc	PF09360	PTHR13680	
14782106	-	inside	690	466	NearestLc	PF13906,P	PTHR11785	KOG1286
14964665	+	downstrea	3121	162	NearestLc	PF14383,P	PTHR21726,PTHR2172	
14975357	-	overlapSt	-33	67	NearestLc	PF01486,P	PTHR11945	KOG0014
15170755	+	upstream	-1563	1397	NearestLocation		PTHR11552,PTHR1155	
15239896	-	overlapSt	52	67	NearestLc	PF01192	PTHR10773	KOG3405
15378451	-	upstream	-2044	1920	NearestLc	PF00046,P	PTHR24326,PTHR2432	
15584094	-	inside	1282	189	NearestLc	PF01158	PTHR10114	KOG3452
15707972	-	inside	2442	461	NearestLc	PF01554	PTHR11206	KOG1347
637149	+	overlapSt	-107	8	NearestLc	PF08477	PTHR11711	KOG0077
782948	-	upstream	-3432	3256	NearestLc	PF00400	PTHR22847	KOG0266
1136628	-	upstream	-2682	2517	NearestLc	PF05498	PTHR33136,PTHR3313	
1676643	+	overlapEn	2855	77	NearestLc	PF07714	PTHR27001	KOG1187
1744938	+	upstream	-2411	2249	NearestLc	PF00657	PTHR22835,PTHR2283	
3353361	+	downstrea	7486	7116	NearestLocation			
3622848	-	inside	1158	87	NearestLc	PF00909	PTHR11730,PTHR1173	
3781878	+	upstream	-3995	3894	NearestLc	PF13424,P	PTHR23083,PTHR2308	
3996963	+	inside	269	152	NearestLc	PF01150	PTHR11782	KOG1385
5311926	-	downstrea	14846	14153	NearestLocation			
5973658	+	downstrea	1240	262	NearestLc	PF05970	PTHR10492	
6404074	+	inside	372	121	NearestLc	PF00346	PTHR11993	KOG2870
6404074	+	upstream	-535	352	NearestLc	PF00346	PTHR11993	KOG2870
6404074	+	inside	372	121	NearestLc	PF00346	PTHR11993	KOG2870
6404074	+	upstream	-535	352	NearestLc	PF00346	PTHR11993	KOG2870
6408104	+	overlapSt	78	189	NearestLc	PF00146	PTHR11432,PTHR1143	
6409298	+	includeFe	626	88	NearestLc	PF00146	PTHR11432,PTHR1143	
6410767	+	overlapSt	-23	17	NearestLc	PF00499	PTHR33269,PTHR3326	
6414887	+	overlapEn	984	330	NearestLocation		PTHR33163,PTHR3316	
6540229	-	downstrea	2035	699	NearestLc	PF04081	PTHR14303,PTHR1430	
8385123	-	upstream	-12133	12001	NearestLc	PF00571	PTHR13780	KOG1764
8385123	-	upstream	-12133	12001	NearestLc	PF00571	PTHR13780	KOG1764
8656708	+	inside	1565	1249	NearestLc	PF00067	PTHR24291	KOG0157
9589819	-	upstream	-3181	2981	NearestLc	PF02149,P	PTHR24343	KOG0583
9589819	-	upstream	-2316	2169	NearestLc	PF02149,P	PTHR24343	KOG0583
9857592	-	inside	228	2	NearestLocation			
10737257	-	downstrea	971	83	NearestLocation			
10737257	-	upstream	-1302	1083	NearestLocation			
10737257	-	downstrea	971	83	NearestLocation			
10737257	-	upstream	-1302	1083	NearestLocation			
11150772	+	overlapEn	3380	14	NearestLc	PF04720	PTHR31579,PTHR3157	
11169792	-	upstream	-24578	24425	NearestLc	PF12680	PTHR33698,PTHR3369	
12675596	-	upstream	-1806	1671	NearestLc	PF02704	PTHR23201,PTHR2320	
12944199	+	inside	1063	478	NearestLc	PF00954,P	PTHR27002	KOG1187
13251015	+	overlapEn	2598	98	NearestLc	PF03634	PTHR31072,PTHR3107	
13251015	+	overlapEn	2598	98	NearestLc	PF03634	PTHR31072,PTHR3107	
13611721	-	upstream	-2606	2503	NearestLc	PF08100,P	PTHR11746,PTHR1174	
13977052	+	upstream	-3185	2945	NearestLocation		PTHR34057,PTHR3405	
14522073	-	inside	617	509	NearestLc	PF12796	PTHR24177	KOG4412
14667419	-	downstrea	2899	2004	NearestLocation		PTHR19271,PTHR1927	
14667419	-	overlapEn	1277	166	NearestLocation		PTHR19271,PTHR1927	
14684971	-	overlapSt	145	80	NearestLc	PF00033	PTHR19271	KOG4663
14755349	-	includeFe	149	53	NearestLc	PF00177	PTHR11205	KOG3291

14757726	+	downstrea	921	184	NearestLocation		PTHR33078
14757726	+	overlapSt	-323	103	NearestLocation		PTHR33078
14765638	-	upstream	-2875	2627	NearestLocation		
14765638	-	upstream	-2008	1880	NearestLocation		
14765638	-	overlapSt	-39	43	NearestLocation		
14765638	-	upstream	-2875	2627	NearestLocation		
14765638	-	upstream	-2008	1880	NearestLocation		
14765638	-	overlapSt	-39	43	NearestLocation		
14765638	-	upstream	-2875	2627	NearestLocation		
14765638	-	upstream	-2008	1880	NearestLocation		
14765638	-	overlapSt	-39	43	NearestLocation		
14771695	-	downstrea	1229	364	NearestLocation		PTHR22773,PTHR2277
14771695	-	downstrea	2649	2136	NearestLocation		PTHR22773,PTHR2277
14773204	+	downstrea	1199	265	NearestLc	PF01578	PTHR30071,PTHR3007
14773204	+	inside	255	67	NearestLc	PF01578	PTHR30071,PTHR3007
14773204	+	downstrea	1199	265	NearestLc	PF01578	PTHR30071,PTHR3007
14773204	+	inside	255	67	NearestLc	PF01578	PTHR30071,PTHR3007
14776118	-	overlapEn	479	165	NearestLocation		
14780816	-	downstrea	1594	532	NearestLocation		PTHR11993,PTHR1199
14784405	-	upstream	-1007	839	NearestLc	PF05758	PTHR33163,PTHR3316
14784405	-	downstrea	972	383	NearestLc	PF05758	PTHR33163,PTHR3316
14789160	+	overlapEn	560	35	NearestLocation		
14790383	-	overlapEn	527	63	NearestLocation		
14790383	-	overlapSt	-383	41	NearestLocation		
14790383	-	overlapEn	527	63	NearestLocation		
14790383	-	overlapSt	-383	41	NearestLocation		
14790383	-	overlapEn	527	63	NearestLocation		
14790383	-	overlapSt	-383	41	NearestLocation		
14792637	+	inside	373	251	NearestLocation		
14795129	+	includeFe	386	139	NearestLocation		PTHR38146,PTHR3814
14814898	+	upstream	-334	200	NearestLc	PF00238	PTHR11761
14837115	-	overlapEn	247	24	NearestLocation		PTHR22855,PTHR2285
14837115	-	downstrea	963	434	NearestLocation		PTHR22855,PTHR2285
14838632	+	downstrea	1267	258	NearestLocation		
14838632	+	downstrea	1963	1092	NearestLocation		
14838632	+	downstrea	1267	258	NearestLocation		
14838632	+	downstrea	1963	1092	NearestLocation		
14845329	-	downstrea	2808	1686	NearestLocation		
14845329	-	overlapSt	-681	375	NearestLocation		
14845329	-	downstrea	2014	931	NearestLocation		
14845329	-	downstrea	2808	1686	NearestLocation		
14845329	-	overlapSt	-681	375	NearestLocation		
14845329	-	downstrea	2014	931	NearestLocation		
14845329	-	downstrea	2808	1686	NearestLocation		
14845329	-	overlapSt	-681	375	NearestLocation		
14845329	-	downstrea	2014	931	NearestLocation		
14849797	+	includeFe	2	313	NearestLc	PF01479,P	PTHR11831,PTHR1183
14849797	+	upstream	-1878	1681	NearestLc	PF01479,P	PTHR11831,PTHR1183
14852408	+	inside	523	65	NearestLocation		PTHR26312,PTHR2631
14854012	+	inside	341	70	NearestLc	PF00223	PTHR30128,PTHR3012
14854012	+	upstream	-544	294	NearestLc	PF00223	PTHR30128,PTHR3012
14869322	+	inside	444	79	NearestLc	PF05151	PTHR35774
14869322	+	upstream	-304	160	NearestLc	PF05151	PTHR35774
14869322	+	inside	444	79	NearestLc	PF05151	PTHR35774

14869322	+	upstream	-304	160	NearestLc	PF05151	PTHR35774	
14880328	-	includeFe	86	479	NearestLocation			
14880328	-	downstrea	1454	1057	NearestLocation			
14884113	+	inside	429	310	NearestLc	PF00137	PTHR10031	
14884113	+	upstream	-633	234	NearestLc	PF00137	PTHR10031	
14896038	+	downstrea	1334	65	NearestLc	PF00124	PTHR33149, PTHR3314	
14907618	+	inside	5563	45	NearestLc	PF00004, P	PTHR33078, PTHR3307	
14907618	+	inside	3009	2524	NearestLc	PF00004, P	PTHR33078, PTHR3307	
14907618	+	inside	2197	2094	NearestLc	PF00004, P	PTHR33078, PTHR3307	
14907618	+	inside	5563	45	NearestLc	PF00004, P	PTHR33078, PTHR3307	
14907618	+	inside	3009	2524	NearestLc	PF00004, P	PTHR33078, PTHR3307	
14907618	+	inside	2197	2094	NearestLc	PF00004, P	PTHR33078, PTHR3307	
15001272	+	inside	2029	1745	NearestLc	PF00481	PTHR13832	KOG0698
15001272	+	upstream	-8769	8599	NearestLc	PF00481	PTHR13832	KOG0698
15835201	+	downstrea	3815	1303	NearestLc	PF14531, P	PTHR24347	KOG0583
16067796	+	downstrea	2943	1999	NearestLocation		PTHR34366, PTHR3436	
16169115	+	inside	1516	1399	NearestLc	PF13855, P	PTHR27003	KOG1187
735001	-	upstream	-1033	876	NearestLc	PF06404	PTHR33285, PTHR3328	
1494323	-	inside	2042	1932	NearestLc	PF00860	PTHR11119, PTHR1111	
1494323	-	inside	4538	87	NearestLc	PF00860	PTHR11119, PTHR1111	
1635605	-	inside	413	204	NearestLc	PF02845	PTHR21494	KOG4501
1635605	-	inside	1343	927	NearestLc	PF02845	PTHR21494	KOG4501
2171379	+	upstream	-1980	1719	NearestLc	PF00046	PTHR24326, PTHR2432	
2171379	+	inside	825	404	NearestLc	PF00046	PTHR24326, PTHR2432	
3326077	-	inside	417	308	NearestLc	PF07974, P	PTHR10942, PTHR1094	
4238982	+	downstrea	1505	1191	NearestLc	PF03140	PTHR31170	
4258542	+	inside	961	826	NearestLc	PF03140	PTHR31549, PTHR3154	
5353654	-	downstrea	7802	5508	NearestLc	PF13912	PTHR26374, PTHR2637	
6230401	-	upstream	-2019	1860	NearestLc	PF00752, P	PTHR11081	KOG2519
6393683	+	inside	215	50	NearestLc	PF00390, P	PTHR23406, PTHR2340	
6430243	-	upstream	-1166	919	NearestLc	PF00763, P	PTHR10025	KOG0089
6972722	-	upstream	-2534	2429	NearestLc	PF00201	PTHR11926, PTHR1192	
6985518	-	inside	2319	2171	NearestLc	PF00069	PTHR24055, PTHR2405	
7287810	-	upstream	-4156	3997	NearestLc	PF13639		
8148330	-	inside	686	136	NearestLc	PF06293	PTHR24349	KOG0032
9200053	-	upstream	-2261	2115	NearestLocation		PTHR33124, PTHR3312	
9356575	+	upstream	-5377	5220	NearestLc	PF14551, P	PTHR11630, PTHR1163	
9356575	+	upstream	-3832	3722	NearestLc	PF14551, P	PTHR11630, PTHR1163	
10405807	-	upstream	-321	112	NearestLc	PF08263, P	PTHR27000, PTHR2700	
10405807	-	inside	1059	425	NearestLc	PF08263, P	PTHR27000, PTHR2700	
11075291	-	upstream	-2021	1899	NearestLc	PF08541, P	PTHR11877, PTHR1187	
11075291	-	inside	173	58	NearestLc	PF08541, P	PTHR11877, PTHR1187	
12398985	+	inside	131	26	NearestLc	PF13920	PTHR12183	KOG4172
12398985	+	upstream	-984	862	NearestLc	PF13920	PTHR12183	KOG4172
12605527	-	upstream	-2591	2480	NearestLc	PF00657	PTHR22835, PTHR2283	
12927225	+	downstrea	8923	7330	NearestLc	PF00646	PTHR32212, PTHR3221	
12927225	+	downstrea	12683	11110	NearestLc	PF00646	PTHR32212, PTHR3221	
14748976	-	inside	883	691	NearestLc	PF13414, P	PTHR27001	KOG1187
15179747	-	inside	1210	955	NearestLc	PF12171	PTHR10593	KOG2462
15932369	-	upstream	-1235	1134	NearestLc	PF00332	PTHR32227, PTHR3222	
16505793	-	upstream	-15168	14844	NearestLc	PF00697	PTHR22854, PTHR2285	
16984962	-	inside	507	90	NearestLc	PF12899	PTHR31916	
16984962	-	downstrea	5427	3829	NearestLc	PF12899	PTHR31916	
16984962	-	downstrea	1250	89	NearestLc	PF12899	PTHR31916	

16984962	-	upstream	-174	61	NearestLc	PF12899	PTHR31916	
16984962	-	downstrea	2494	1025	NearestLc	PF12899	PTHR31916	
17227664	+	upstream	-10683	10546	NearestLc	PF00730	PTHR15074	
17227664	+	upstream	-10683	10546	NearestLc	PF00730	PTHR15074	
18148766	-	downstrea	12964	3751	NearestLc	PF00400	PTHR22874	KOG0266
18326154	-	upstream	-6960	6846	NearestLc	PF00560	PTHR24106	KOG4308
18428448	+	overlapSt	187	1	NearestLc	PF02450	PTHR11440	KOG2369
461241	+	inside	412	297	NearestLc	PF01182	PTHR11054	KOG3147
1863368	+	overlapSt	-35	105	NearestLc	PF00704	PTHR11177,PTHR1117	
1904272	+	inside	740	542	NearestLc	PF00270,P	PTHR18934,PTHR1893	
2353767	+	upstream	-2644	2520	NearestLc	PF16487,P	PTHR22891,PTHR2289	
3513035	-	overlapSt	167	60	NearestLc	PF03953,P	PTHR11588,PTHR1158	
4014031	+	upstream	-2092	1990	NearestLc	PF04577	PTHR20961	KOG4698
4102910	+	overlapSt	174	96	NearestLocation		PTHR24014	KOG1971
4560593	-	inside	2475	2337	NearestLc	PF08969,P	PTHR12947	KOG2880
4977552	+	upstream	-13243	13119	NearestLc	PF00010	PTHR12565,PTHR1256	
4977552	+	upstream	-2239	2041	NearestLc	PF00010	PTHR12565,PTHR1256	
4977552	+	overlapSt	-44	82	NearestLc	PF00010	PTHR12565,PTHR1256	
4977552	+	upstream	-13243	13119	NearestLc	PF00010	PTHR12565,PTHR1256	
4977552	+	upstream	-2239	2041	NearestLc	PF00010	PTHR12565,PTHR1256	
4977552	+	overlapSt	-44	82	NearestLc	PF00010	PTHR12565,PTHR1256	
4977552	+	upstream	-13243	13119	NearestLc	PF00010	PTHR12565,PTHR1256	
4977552	+	upstream	-2239	2041	NearestLc	PF00010	PTHR12565,PTHR1256	
4977552	+	overlapSt	-44	82	NearestLc	PF00010	PTHR12565,PTHR1256	
6289859	-	downstrea	24057	22585	NearestLocation		PTHR11410,PTHR1141	
6289859	-	inside	122	12	NearestLocation		PTHR11410,PTHR1141	
6289859	-	downstrea	157183	155751	NearestLocation		PTHR11410,PTHR1141	
6289859	-	downstrea	24057	22585	NearestLocation		PTHR11410,PTHR1141	
6289859	-	inside	122	12	NearestLocation		PTHR11410,PTHR1141	
6289859	-	downstrea	157183	155751	NearestLocation		PTHR11410,PTHR1141	
6322830	+	downstrea	2299	462	NearestLocation		PTHR22773,PTHR2277	
6322830	+	overlapEn	274	58	NearestLocation		PTHR22773,PTHR2277	
6322830	+	downstrea	2299	462	NearestLocation		PTHR22773,PTHR2277	
6322830	+	overlapEn	274	58	NearestLocation		PTHR22773,PTHR2277	
6329003	+	upstream	-713	176	NearestLocation			
6329003	+	downstrea	1541	771	NearestLocation			
6329003	+	inside	254	21	NearestLocation			
6329003	+	upstream	-1836	1704	NearestLocation			
6329003	+	downstrea	2807	1973	NearestLocation			
6329003	+	upstream	-713	176	NearestLocation			
6329003	+	downstrea	1541	771	NearestLocation			
6329003	+	inside	254	21	NearestLocation			
6329003	+	upstream	-1836	1704	NearestLocation			
6329003	+	downstrea	2807	1973	NearestLocation			
6329003	+	upstream	-713	176	NearestLocation			
6329003	+	downstrea	1541	771	NearestLocation			
6329003	+	inside	254	21	NearestLocation			
6329003	+	upstream	-1836	1704	NearestLocation			
6329003	+	downstrea	2807	1973	NearestLocation			
6329003	+	upstream	-713	176	NearestLocation			
6329003	+	downstrea	1541	771	NearestLocation			
6329003	+	inside	254	21	NearestLocation			
6329003	+	upstream	-1836	1704	NearestLocation			
6329003	+	downstrea	2807	1973	NearestLocation			

6329003	+	upstream	-713	176	NearestLocation			
6329003	+	downstrea	1541	771	NearestLocation			
6329003	+	inside	254	21	NearestLocation			
6329003	+	upstream	-1836	1704	NearestLocation			
6329003	+	downstrea	2807	1973	NearestLocation			
6329003	+	upstream	-713	176	NearestLocation			
6329003	+	downstrea	1541	771	NearestLocation			
6329003	+	inside	254	21	NearestLocation			
6329003	+	upstream	-1836	1704	NearestLocation			
6329003	+	downstrea	2807	1973	NearestLocation			
6408513	+	upstream	-8019	7646	NearestLc	PF00026	PTHR13683	KOG1339
6408513	+	downstrea	3042	694	NearestLc	PF00026	PTHR13683	KOG1339
6581823	-	downstrea	10149	9834	NearestLc	PF08263	PTHR27006,PTHR2700	
6632413	-	upstream	-49856	49752	NearestLc	PF00160	PTHR11071	KOG0880
6957260	-	upstream	-12398	12294	NearestLc	PF14392,P	PTHR31286,PTHR3128	
7438842	-	downstrea	6525	5534	NearestLocation			
8388412	-	overlapSt	10	189	NearestLc	PF07714	PTHR24056,PTHR2405	
8388412	-	inside	5518	1166	NearestLc	PF07714	PTHR24056,PTHR2405	
8888635	-	inside	1395	425	NearestLc	PF00544,P	PTHR31683,PTHR3168	
10235369	+	inside	142	18	NearestLocation			
11321489	-	inside	201	99	NearestLocation		PTHR38398,PTHR3839	
12322478	+	upstream	-4075	3964	NearestLc	PF00010	PTHR31945,PTHR3194	
13657757	+	inside	2757	1586	NearestLc	PF00118	PTHR11353,PTHR1135	
13716224	+	inside	754	625	NearestLc	PF00118	PTHR11353	KOG0356
14299705	+	downstrea	2708	229	NearestLc	PF01477	PTHR31718,PTHR3171	
14916885	-	upstream	-2270	1995	NearestLc	PF00752,P	PTHR11081	KOG2519
15246439	+	inside	717	323	NearestLocation		PTHR34367,PTHR3436	
626889	-	downstrea	1589	898	NearestLocation			
626889	-	downstrea	1589	898	NearestLocation			
905624	+	inside	1359	51	NearestLc	PF00201	PTHR11926,PTHR1192	
1869367	-	downstrea	1306	188	NearestLc	PF08711,P	PTHR11477	KOG1105
3542526	+	overlapSt	107	21	NearestLocation			
3796694	-	overlapSt	326	6	NearestLc	PF04759	PTHR31696,PTHR3169	
4120192	-	overlapSt	192	10	NearestLc	PF03145	PTHR10315	KOG3002
4267758	-	inside	474	182	NearestLc	PF03018	PTHR21495,PTHR2149	
4960872	+	inside	162	36	NearestLc	PF00270,P	PTHR18934,PTHR1893	
6099917	+	includeFe	784	2	NearestLocation		PTHR34120,PTHR3412	
6580823	+	inside	348	97	NearestLc	PF10018	PTHR13208,PTHR1320	
6681346	+	inside	363	239	NearestLc	PF00808	PTHR11064	KOG0869
7277504	-	inside	1512	33	NearestLc	PF00223	PTHR30128,PTHR3012	
7711361	+	upstream	-12079	11976	NearestLocation			
7711361	+	upstream	-31872	31766	NearestLocation			
7711361	+	upstream	-20927	20824	NearestLocation			
8102394	+	downstrea	28827	28290	NearestLc	PF05678	PTHR33624,PTHR3362	
8219153	+	inside	199	68	NearestLc	PF01333,P	PTHR33288,PTHR3328	
11100463	-	downstrea	4832	4334	NearestLocation		PTHR23056,PTHR2305	
12350099	-	inside	469	108	NearestLc	PF02383	PTHR11200,PTHR1120	
12543487	+	inside	1268	1035	NearestLc	PF08263,P	PTHR27004	KOG0472
13768493	-	upstream	-3445	3315	NearestLocation			
13990909	-	upstream	-1830	1635	NearestLocation			
13990909	-	inside	557	300	NearestLocation			
809233	+	upstream	-493	385	NearestLc	PF13947,P	PTHR27009	KOG1187
2045055	+	upstream	-701	584	NearestLc	PF12579	PTHR14000	
2262332	+	overlapEn	5359	34	NearestLc	PF03140	PTHR31170	

2353586	+	downstrea	2686	2063	NearestLocation			
2669811	+	upstream	-3468	3347	NearestLc	PF02365	PTHR31719,PTHR3171	
3583948	-	downstrea	1670	1183	NearestLc	PF00320	PTHR10071,PTHR1007	
3583948	-	downstrea	655	165	NearestLc	PF00320	PTHR10071,PTHR1007	
5644665	-	inside	999	788	NearestLc	PF02362	PTHR31140,PTHR3114	
5863374	-	upstream	-5358	5236	NearestLc	PF01202	PTHR21087,PTHR2108	
5941428	+	inside	135	8	NearestLocation		PTHR19376,PTHR1937	
5941428	+	inside	135	8	NearestLocation		PTHR19376,PTHR1937	
6944376	+	upstream	-18354	18244	NearestLc	PF09273	PTHR13271	KOG1337
7264096	+	downstrea	38392	35916	NearestLocation			
7365135	-	downstrea	6179	5688	NearestLocation			
8297045	+	upstream	-3143	3022	NearestLocation			
8766101	-	inside	1057	440	NearestLc	PF03171	PTHR10869	KOG1591
8766101	-	inside	669	563	NearestLc	PF03171	PTHR10869	KOG1591
8766101	-	upstream	-3084	2962	NearestLc	PF03171	PTHR10869	KOG1591
9601523	-	inside	925	594	NearestLc	PF00462	PTHR10168	KOG2824
9601523	-	downstrea	3644	1843	NearestLc	PF00462	PTHR10168	KOG2824
9601523	-	overlapSt	15	203	NearestLc	PF00462	PTHR10168	KOG2824
9638227	+	upstream	-1986	1855	NearestLc	PF00046,P	PTHR24326	KOG0483
10065639	+	upstream	-10267	9981	NearestLocation			
10953970	+	overlapSt	117	17	NearestLc	PF05193,P	PTHR11851,PTHR1185	
10953970	+	overlapSt	117	17	NearestLc	PF05193,P	PTHR11851,PTHR1185	
11508931	-	inside	805	648	NearestLc	PF00005,P	PTHR24221,PTHR2422	
13014518	+	upstream	-7870	7695	NearestLc	PF12338,P	PTHR31262,PTHR3126	
13156337	+	upstream	-3344	3208	NearestLc	PF07690	PTHR23500	KOG0569
13532208	-	upstream	-1544	1419	NearestLc	PF03547	PTHR31419	KOG2722
14201727	+	upstream	-654	504	NearestLc	PF00931	PTHR23155,PTHR2315	
14836690	-	upstream	-229	84	NearestLc	PF00574	PTHR10381,PTHR1038	
14836690	-	inside	637	306	NearestLc	PF00574	PTHR10381,PTHR1038	
14839672	+	upstream	-628	517	NearestLc	PF00421	PTHR33180,PTHR3318	
15115017	-	downstrea	13134	9893	NearestLc	PF00931	PTHR23155	KOG4658
15520019	+	inside	1568	959	NearestLc	PF04716	PTHR12653	KOG3365
1276741	-	upstream	-2209	2076	NearestLc	PF14299	PTHR32278,PTHR3227	
2322849	-	inside	1137	740	NearestLc	PF00170	PTHR13301,PTHR1330	
2346896	-	downstrea	2828	1115	NearestLc	PF00641	PTHR23111	KOG4198
2437920	+	inside	733	179	NearestLc	PF01479	PTHR11831,PTHR1183	
2682556	-	inside	309	69	NearestLc	PF14226,P	PTHR10209	KOG0143
3018845	+	inside	1338	1224	NearestLc	PF00743	PTHR23023	KOG1399
3018845	+	upstream	-231	66	NearestLc	PF00743	PTHR23023	KOG1399
3122947	+	upstream	-11779	11663	NearestLc	PF01925	PTHR14255,PTHR1425	
3238309	+	inside	2533	906	NearestLc	PF00179	PTHR24067	KOG0417
4061935	-	inside	365	88	NearestLocation			
4129941	+	inside	6179	1209	NearestLc	PF00046,P	PTHR24326	KOG0483
4129941	+	upstream	-9942	9833	NearestLc	PF00046,P	PTHR24326	KOG0483
4129941	+	upstream	-10976	10761	NearestLc	PF00046,P	PTHR24326	KOG0483
4129941	+	upstream	-7499	7397	NearestLc	PF00046,P	PTHR24326	KOG0483
4678709	-	upstream	-12287	12181	NearestLc	PF07650,P	PTHR11760	KOG3181
5007839	-	upstream	-23969	23867	NearestLc	PF00249	PTHR10641	KOG0048
5256910	+	upstream	-10321	10197	NearestLc	PF14531	PTHR24361	KOG0581
6368928	-	overlapEn	3270	18	NearestLc	PF01650	PTHR12000,PTHR1200	
6368928	-	inside	1285	1089	NearestLc	PF01650	PTHR12000,PTHR1200	
6368928	-	upstream	-31545	31401	NearestLc	PF01650	PTHR12000,PTHR1200	
6368928	-	overlapSt	56	304	NearestLc	PF01650	PTHR12000,PTHR1200	
6537885	+	inside	4015	754	NearestLc	PF00481	PTHR13832	KOG0698

6537885	+	downstrea	75632	70534	NearestLc	PF00481	PTHR13832	KOG0698
6537885	+	downstrea	48509	43420	NearestLc	PF00481	PTHR13832	KOG0698
6537885	+	inside	4015	754	NearestLc	PF00481	PTHR13832	KOG0698
6537885	+	downstrea	75632	70534	NearestLc	PF00481	PTHR13832	KOG0698
6537885	+	downstrea	48509	43420	NearestLc	PF00481	PTHR13832	KOG0698
7374985	+	upstream	-4750	4647	NearestLocation			
7374985	+	upstream	-18334	18208	NearestLocation			
7770793	+	overlapSt	79	33	NearestLc	PF02140,P	PTHR23421,PTHR2342	
7770793	+	upstream	-401	220	NearestLc	PF02140,P	PTHR23421,PTHR2342	
8142269	-	downstrea	11358	2505	NearestLc	PF01388	PTHR22970,PTHR2297	
11642935	-	upstream	-4585	4468	NearestLc	PF00657	PTHR22835,PTHR2283	
11699851	-	upstream	-6757	6645	NearestLc	PF00657	PTHR22835,PTHR2283	
12573071	+	inside	794	605	NearestLc	PF02861	PTHR11638,PTHR1163	
12573071	+	inside	1659	1460	NearestLc	PF02861	PTHR11638,PTHR1163	
12573071	+	inside	2210	2094	NearestLc	PF02861	PTHR11638,PTHR1163	
12573071	+	upstream	-8642	8474	NearestLc	PF02861	PTHR11638,PTHR1163	
12644045	-	downstrea	3335	2435	NearestLc	PF13639	PTHR14155,PTHR1415	
12644045	-	downstrea	3335	2435	NearestLc	PF13639	PTHR14155,PTHR1415	
12802179	-	downstrea	4645	1560	NearestLc	PF02297	PTHR11387,PTHR1138	
13029911	-	upstream	-3909	3785	NearestLocation		PTHR38365,PTHR3836	
15516682	+	downstrea	3747	671	NearestLocation		PTHR23155,PTHR2315	
15591232	-	upstream	-427	285	NearestLocation			
288019	-	overlapEn	1543	116	NearestLc	PF08787	PTHR33681,PTHR3368	
459522	+	upstream	-1089	988	NearestLc	PF00190	PTHR31189,PTHR3118	
1436609	+	upstream	-1778	1566	NearestLc	PF04576	PTHR31448,PTHR3144	
1436609	+	inside	3035	705	NearestLc	PF04576	PTHR31448,PTHR3144	
1994306	-	upstream	-4391	4271	NearestLocation			
2152955	+	upstream	-2078	1972	NearestLc	PF13921,P	PTHR10641,PTHR1064	
2175874	+	downstrea	9324	7490	NearestLc	PF04852	PTHR31165,PTHR3116	
2313422	+	inside	2513	1274	NearestLc	PF03107,P	PTHR32410,PTHR3241	
2757351	+	inside	238	127	NearestLocation			
3189958	+	inside	168	56	NearestLocation		PTHR34995,PTHR3499	
3189958	+	inside	857	710	NearestLocation		PTHR34995,PTHR3499	
3193492	+	downstrea	1615	471	NearestLc	PF04983,P	PTHR19376	
3193492	+	inside	545	359	NearestLc	PF04983,P	PTHR19376	
3193492	+	downstrea	1615	471	NearestLc	PF04983,P	PTHR19376	
3193492	+	inside	545	359	NearestLc	PF04983,P	PTHR19376	
3195886	-	inside	597	68	NearestLc	PF00223	PTHR30128,PTHR3012	
3202760	-	inside	204	97	NearestLc	PF00137	PTHR10031,PTHR1003	
3202760	-	upstream	-418	116	NearestLc	PF00137	PTHR10031,PTHR1003	
3835094	+	inside	1048	938	NearestLc	PF00628,P	PTHR10333	KOG1973
4759878	+	downstrea	8830	7667	NearestLc	PF06094	PTHR12510	KOG4450
4759878	+	inside	452	258	NearestLc	PF06094	PTHR12510	KOG4450
4759878	+	downstrea	8251	7117	NearestLc	PF06094	PTHR12510	KOG4450
4777541	-	downstrea	1776	1180	NearestLocation			
5321204	+	inside	145	16	NearestLc	PF01010	PTHR22773,PTHR2277	
5540996	-	upstream	-5280	4990	NearestLc	PF00249	PTHR10641	KOG0048
6691334	-	overlapSt	-10	272	NearestLc	PF00421	PTHR33180,PTHR3318	
7542120	+	inside	1077	671	NearestLc	PF01048	PTHR21234,PTHR2123	
7849391	-	downstrea	15239	11262	NearestLc	PF07725,P	PTHR11017	KOG4658
7849391	-	downstrea	14788	10821	NearestLc	PF07725,P	PTHR11017	KOG4658
7849391	-	downstrea	15239	11262	NearestLc	PF07725,P	PTHR11017	KOG4658
7849391	-	downstrea	14788	10821	NearestLc	PF07725,P	PTHR11017	KOG4658
9985937	-	upstream	-185	78	NearestLc	PF00295	PTHR31375,PTHR3137	

9985937	-	overlapEn	1767	92	NearestLc	PF00295	PTHR31375,PTHR3137	
9985937	-	downstrea	3281	1397	NearestLc	PF00295	PTHR31375,PTHR3137	
9985937	-	upstream	-937	772	NearestLc	PF00295	PTHR31375,PTHR3137	
9985937	-	upstream	-185	78	NearestLc	PF00295	PTHR31375,PTHR3137	
9985937	-	overlapEn	1767	92	NearestLc	PF00295	PTHR31375,PTHR3137	
9985937	-	downstrea	3281	1397	NearestLc	PF00295	PTHR31375,PTHR3137	
9985937	-	upstream	-937	772	NearestLc	PF00295	PTHR31375,PTHR3137	
10016353	+	inside	1877	1669	NearestLc	PF13334,P	PTHR11214	KOG2288
11356825	-	inside	6248	3822	NearestLc	PF07690	PTHR11654,PTHR1165	
11356825	-	inside	336	167	NearestLc	PF07690	PTHR11654,PTHR1165	
11800559	-	inside	3415	276	NearestLc	PF13347	PTHR19432,PTHR1943	
11800559	-	inside	1339	1190	NearestLc	PF13347	PTHR19432,PTHR1943	
11800559	-	inside	621	234	NearestLc	PF13347	PTHR19432,PTHR1943	
12331554	-	upstream	-176	75	NearestLocation		PTHR12499,PTHR1249	
12960683	+	upstream	-308	178	NearestLc	PF02365	PTHR31989,PTHR3198	
13062592	+	overlapEn	3091	47	NearestLc	PF13962,P	PTHR24177	KOG4412
13062592	+	inside	988	691	NearestLc	PF13962,P	PTHR24177	KOG4412
13311828	-	upstream	-950	778	NearestLc	PF01342	PTHR34053,PTHR3405	
15377510	+	inside	604	68	NearestLc	PF16940	PTHR34935	
197138	-	upstream	-38924	38811	NearestLc	PF01397,P	PTHR31225,PTHR3122	
197138	-	inside	222	57	NearestLc	PF01397,P	PTHR31225,PTHR3122	
436960	-	downstrea	97738	96057	NearestLc	PF00306,P	PTHR15184	KOG1350
436960	-	downstrea	98972	97275	NearestLc	PF00306,P	PTHR15184	KOG1350
436960	-	overlapSt	89	24	NearestLc	PF00306,P	PTHR15184	KOG1350
436960	-	downstrea	96892	95144	NearestLc	PF00306,P	PTHR15184	KOG1350
436960	-	inside	1140	309	NearestLc	PF00306,P	PTHR15184	KOG1350
436960	-	downstrea	97738	96057	NearestLc	PF00306,P	PTHR15184	KOG1350
436960	-	downstrea	98972	97275	NearestLc	PF00306,P	PTHR15184	KOG1350
436960	-	overlapSt	89	24	NearestLc	PF00306,P	PTHR15184	KOG1350
436960	-	downstrea	96892	95144	NearestLc	PF00306,P	PTHR15184	KOG1350
436960	-	inside	1140	309	NearestLc	PF00306,P	PTHR15184	KOG1350
439050	+	overlapEn	385	35	NearestLocation			
748017	-	downstrea	1557	11	NearestLc	PF00223	PTHR30128,PTHR3012	
101775	+	downstrea	1449	204	NearestLocation			
101775	+	inside	839	50	NearestLocation			
388907	-	inside	618	516	NearestLc	PF13855,P	PTHR23155	KOG4658
477251	-	downstrea	10520	7871	NearestLc	PF04398	PTHR31676,PTHR3167	
186314	-	upstream	-6613	6499	NearestLc	PF05617	PTHR31951,PTHR3195	
231069	-	downstrea	42593	42006	NearestLc	PF00421	PTHR33180,PTHR3318	
231069	-	downstrea	4203	3665	NearestLc	PF00421	PTHR33180,PTHR3318	
231069	-	downstrea	42593	42006	NearestLc	PF00421	PTHR33180,PTHR3318	
231069	-	downstrea	4203	3665	NearestLc	PF00421	PTHR33180,PTHR3318	
129285	+	inside	1995	1334	NearestLc	PF08276,P	PTHR27002	KOG1187
287644	-	downstrea	57862	56994	NearestLc	PF14392,P	PTHR31286,PTHR3128	
287644	-	downstrea	57862	56994	NearestLc	PF14392,P	PTHR31286,PTHR3128	
244128	+	downstrea	3493	2921	NearestLc	PF02362		
244128	+	downstrea	2608	2068	NearestLc	PF02362		
244128	+	downstrea	4176	3746	NearestLc	PF02362		
244128	+	downstrea	3493	2921	NearestLc	PF02362		
244128	+	downstrea	2608	2068	NearestLc	PF02362		
244128	+	downstrea	4176	3746	NearestLc	PF02362		
244128	+	downstrea	3493	2921	NearestLc	PF02362		
244128	+	downstrea	2608	2068	NearestLc	PF02362		
244128	+	downstrea	4176	3746	NearestLc	PF02362		

261795	+	downstrea	2228	1810	NearestLocation			
279568	+	upstream	-6895	6744	NearestLocation			
104569	+	downstrea	12342	4722	NearestLc	PF16987	PTHR33137	
104569	+	upstream	-18093	17991	NearestLc	PF16987	PTHR33137	
104569	+	downstrea	17404	9786	NearestLc	PF16987	PTHR33137	
104569	+	inside	5683	1704	NearestLc	PF16987	PTHR33137	
104569	+	downstrea	12342	4722	NearestLc	PF16987	PTHR33137	
104569	+	upstream	-18093	17991	NearestLc	PF16987	PTHR33137	
104569	+	downstrea	17404	9786	NearestLc	PF16987	PTHR33137	
104569	+	inside	5683	1704	NearestLc	PF16987	PTHR33137	
155580	-	inside	1942	1835	NearestLc	PF00560,P	PTHR27004	KOG0472
58095	-	downstrea	6603	3210	NearestLc	PF00931	PTHR23155,PTHR2315	
197414	-	downstrea	3622	974	NearestLc	PF00141	PTHR31235,PTHR3123	
183870	+	upstream	-1881	1767	NearestLocation			
196597	-	downstrea	13095	11365	NearestLc	PF13202,P	PTHR23063	
116547	+	upstream	-3087	2934	NearestLc	PF00931	PTHR23155	KOG4658
116547	+	inside	596	452	NearestLc	PF00931	PTHR23155	KOG4658
116547	+	inside	3521	3379	NearestLc	PF00931	PTHR23155	KOG4658
17456	-	inside	660	520	NearestLc	PF14700,P	PTHR10102,PTHR1010	
17456	-	inside	660	520	NearestLc	PF14700,P	PTHR10102,PTHR1010	
71679	-	overlapSt	415	55	NearestLc	PF00361,P	PTHR22773,PTHR2277	
124641	-	upstream	-149	35	NearestLc	PF13676,P	PTHR11017	KOG0472
18426	+	upstream	-1025	894	NearestLocation		PTHR34361	
4020	+	inside	331	59	NearestLc	PF00188	PTHR10334,PTHR1033	
4020	+	upstream	-1087	952	NearestLc	PF00188	PTHR10334,PTHR1033	
33003	+	upstream	-11287	11169	NearestLc	PF08263,P	PTHR27004	KOG0472
65358	+	upstream	-3754	3619	NearestLc	PF03140	PTHR31170	
8055	-	upstream	-8381	7461	NearestLc	PF01373	PTHR31352,PTHR3135	
8055	-	upstream	-7119	6934	NearestLc	PF01373	PTHR31352,PTHR3135	
8055	-	upstream	-8381	7461	NearestLc	PF01373	PTHR31352,PTHR3135	
8055	-	upstream	-7119	6934	NearestLc	PF01373	PTHR31352,PTHR3135	
29736	+	upstream	-5465	5269	NearestLocation		PTHR36036	
61778	+	inside	303	18	NearestLc	PF05758	PTHR33163,PTHR3316	
46630	+	downstrea	7895	7418	NearestLocation		PTHR10903,PTHR1090	
13165	+	upstream	-766	641	NearestLc	PF11543,P	PTHR12710	KOG2834
30088	+	downstrea	9334	7717	NearestLc	PF02458	PTHR31623,PTHR3162	
73058	-	downstrea	2808	573	NearestLc	PF13193,P	PTHR24095,PTHR2409	
19366	-	upstream	-4698	4593	NearestLc	PF08263,P	PTHR27004	KOG0472
19366	-	upstream	-11308	11201	NearestLc	PF08263,P	PTHR27004	KOG0472
53329	+	upstream	-3029	2905	NearestLc	PF00581	PTHR12231	KOG1530
15851	+	upstream	-2104	1998	NearestLocation		PTHR34124	
13396	-	overlapEn	952	1	NearestLc	PF14111	PTHR33233,PTHR3323	
5317	-	downstrea	3541	2633	NearestLocation			
5317	-	downstrea	1747	676	NearestLocation			
706	-	upstream	-1388	1284	NearestLocation		PTHR12565,PTHR1256	

ec	KO	GO	Best.hit.arabi.sym		arabi.defline
38:SF2		GO:000551	AT2G44430.1		DNA-binding bromodomain-containing p
3.6.3.17		GO:001688	AT1G15520	ABCG40,AT	pleiotropic drug resistance 12
3.6.3.17		GO:001688	AT1G15520	ABCG40,AT	pleiotropic drug resistance 12
3.6.3.17		GO:001688	AT1G15520	ABCG40,AT	pleiotropic drug resistance 12
3.6.3.17		GO:001688	AT1G15520	ABCG40,AT	pleiotropic drug resistance 12
			AT2G33520.1		
2.7.11.1		GO:000551	AT1G74180	AtRLP14,R	receptor like protein 14
			AT2G34400.1		Pentatricopeptide repeat (PPR-like)
7:SF12	K09286	GO:000635	AT5G13910	LEP	Integrase-type DNA-binding superfami
			AT5G32450.1		RNA binding (RRM/RBD/RNP motifs) fam
2.7.11.1		GO:000646	AT1G56140.1		Leucine-rich repeat transmembrane pr
2.7.11.1		GO:000646	AT1G56140.1		Leucine-rich repeat transmembrane pr
52:SF22			AT1G64450.1		Glycine-rich protein family
52:SF22			AT1G64450.1		Glycine-rich protein family
55:SF157		GO:003024	AT5G53110.1		RING/U-box superfamily protein
55:SF157		GO:003024	AT5G53110.1		RING/U-box superfamily protein
55:SF157		GO:003024	AT5G53110.1		RING/U-box superfamily protein
00:SF15			AT4G22810.1		Predicted AT-hook DNA-binding family
1.4.3.21	K00276	GO:005511	AT4G12290.1		Copper amine oxidase family protein
			AT4G22540	ORP2A	OSBP(oxysterol binding protein)-rela
28:SF3			AT2G35736.1		
33:SF2			AT2G35230	IKU1	VQ motif-containing protein
00:SF14			AT4G17800.1		Predicted AT-hook DNA-binding family
7:SF8	K09286	GO:000635	AT5G47230	ATERF-5,A	ethylene responsive element binding
3.1.2.14		GO:001679	AT1G08510	FATB	fatty acyl-ACP thioesterases B
3.1.2.14		GO:001679	AT1G08510	FATB	fatty acyl-ACP thioesterases B
58:SF6			AT2G25735.1		
46:SF14			AT4G29000.1		Tesmin/TSO1-like CXC domain-containi
	K15174	GO:001659	AT1G79730	ELF7	hydroxyproline-rich glycoprotein fam
50:SF4			AT4G19160.2		
3.1.3.5	K03787	GO:001678	AT1G72880.2		Survival protein SurE-like phosphata
6.1.1.11		GO:000641	AT5G27470.1		seryl-tRNA synthetase / serine--tRNA
27:SF1			AT5G64550.1		loricrin-related
34:SF8			AT2G01080.1		Late embryogenesis abundant (LEA) hy
	K14827		AT5G06350.1		ARM repeat superfamily protein
48:SF34			AT1G77700.1		Pathogenesis-related thaumatin super
75:SF2		GO:000727	AT1G08465	YAB2	Plant-specific transcription factor
75:SF2		GO:000727	AT1G08465	YAB2	Plant-specific transcription factor
2.7.11.1		GO:000646	AT3G23750.1		Leucine-rich repeat protein kinase f
48:SF32			AT1G75800.1		Pathogenesis-related thaumatin super
33:SF6	K18466		AT5G53530	VPS26A	vacuolar protein sorting 26A
9:SF1		GO:000551	AT5G59990.1		CCT motif family protein
2.7.11.1		GO:000695	AT2G45910.1		U-box domain-containing protein kina
		GO:005511	AT1G12740	CYP87A2	cytochrome P450, family 87, subfamil

		GO:005511	AT1G12740	CYP87A2	cytochrome P450, family 87, subfamil
35:SF4			AT5G13500.3		
35:SF4			AT5G13500.3		
			AT2G38300.1		myb-like HTH transcriptional regulat
2.7.11.1		GO:001602	AT5G10530.1		Concanavalin A-like lectin protein k
47:SF5			AT4G33800.1		
	K02991	GO:000641	AT5G10360	EMB3010, R	Ribosomal protein S6e
1.10.3.9	K02706	GO:004515	ATCG00270.1		photosystem II reaction center prote
21:SF50		GO:000905	AT3G27200.1		Cupredoxin superfamily protein
			AT3G27340.1		
4.2.2.2	K01728		AT3G27400.1		Pectin lyase-like superfamily protei
4.2.2.2	K01728		AT3G27400.1		Pectin lyase-like superfamily protei
			AT2G34680	AIR9	Outer arm dynein light chain 1 prote
	K14404		AT1G30460	ATCPSF30,	cleavage and polyadenylation specifi
	K14404		AT1G30460	ATCPSF30,	cleavage and polyadenylation specifi
		GO:001602	AT1G30360	ERD4	Early-responsive to dehydration stre
3.1.1.1			AT1G47480.1		alpha/beta-Hydrolases superfamily pr
2.7.11.1			AT1G25320.1		Leucine-rich repeat protein kinase f
1.6.5.5		GO:005511	AT4G13010.1		Oxidoreductase, zinc-binding dehydro
3.6.1.31,	K11755	GO:000463	AT1G31860	AT-IE,HIS	histidine biosynthesis bifunctional
02:SF8		GO:000369	AT4G20010	OSB2,PTAC	plastid transcriptionally active 9
2.3.1.115		GO:001674	AT5G39050.1		HXXXD-type acyl-transferase family p
77:SF7	K09286	GO:000635	AT3G15210	ATERF-4,A	ethylene responsive element binding
77:SF7	K09286	GO:000635	AT3G15210	ATERF-4,A	ethylene responsive element binding
77:SF7	K09286	GO:000635	AT3G15210	ATERF-4,A	ethylene responsive element binding
02:SF6			AT1G28280.2		VQ motif-containing protein
.7:SF476		GO:005508	AT5G54250	ATCNGC4,C	cyclic nucleotide-gated cation chann
		GO:004353	AT4G27190.1		NB-ARC domain-containing disease res
		GO:004353	AT4G27190.1		NB-ARC domain-containing disease res
3.6.1.1	K01514				
3.6.1.1	K01514				
3.6.1.1	K01514				
48:SF10			AT1G64700.1		
40:SF4		GO:000367	AT2G46870	NGA1	AP2/B3-like transcriptional factor f
	K14297	GO:000681	AT1G10390.2		Nucleoporin autopeptidase
06:SF18		GO:004356	AT1G30650	AR411,ATW	WRKY DNA-binding protein 14
1.3.3.8		GO:005511	AT1G30700.1		FAD-binding Berberine family protein
1.1.1.34	K00021	GO:005511	AT1G76490	HMG1,HMGR	hydroxy methylglutaryl CoA reductase
	K02155	GO:003317	AT1G19910	ATVHA-C2,	ATPase, F0/V0 complex, subunit C pro
32:SF6		GO:000827	AT1G75540	STH2	salt tolerance homolog2
32:SF6		GO:000827	AT1G75540	STH2	salt tolerance homolog2
33:SF0		GO:000551	AT3G54350	emb1967	Forkhead-associated (FHA) domain-con
38:SF10			AT2G31260	APG9,ATAP	autophagy 9 (APG9)

1.97.1.12	K02689	GO:001602	ATCG00350.1		Photosystem I, PsaA/PsaB protein
			AT1G55190	PRA1.F2,P	PRA1 (Prenylated rab acceptor) famil
			AT1G55190	PRA1.F2,P	PRA1 (Prenylated rab acceptor) famil
			AT1G03080.1		kinase interacting (KIP1-like) famil
		GO:000662	AT3G63200	PLA IIIB,	PATATIN-like protein 9
		GO:000662	AT3G63200	PLA IIIB,	PATATIN-like protein 9
34:SF33		GO:000635	AT5G37020	ARF8,ATAR	auxin response factor 8
34:SF33		GO:000635	AT5G37020	ARF8,ATAR	auxin response factor 8
		GO:001602	AT4G19645.2		TRAM, LAG1 and CLN8 (TLC) lipid-sens
1.13.11.2	K00457	GO:005511	AT1G06570	HPD,PDS1	phytoene desaturation 1
52:SF8		GO:005508	AT1G77110	PIN6	Auxin efflux carrier family protein
			AT1G77380	AAP3,ATAA	amino acid permease 3
.8:SF12		GO:001602	AT1G21890.1		nodulin MtN21 /EamA-like transporter
	K13946		AT1G77690	LAX3	like AUX1 3
57:SF9	K09286	GO:000635	AT1G78080	RAP2.4	related to AP2 4
57:SF9	K09286	GO:000635	AT1G78080	RAP2.4	related to AP2 4
57:SF9	K09286	GO:000635	AT1G78080	RAP2.4	related to AP2 4
.5:SF6			AT1G78100.1		F-box family protein
18:SF3			AT1G78110.1		
18:SF3			AT1G78110.1		
	K09874	GO:001602	AT5G37820	NIP4;2,NL	NOD26-like intrinsic protein 4;2
			AT4G08980	FBW2	F-BOX WITH WD-40 2
	K03327	GO:005508	AT1G58340	ZF14	MATE efflux family protein
	K03327	GO:005508	AT1G58340	ZF14	MATE efflux family protein
2.7.11.1		GO:000716	AT4G24400	ATCIPK8,C	CBL-interacting protein kinase 8
2.7.11.1		GO:000551	AT5G49660.1		Leucine-rich repeat transmembrane pr
2.7.11.1		GO:000551	AT5G49660.1		Leucine-rich repeat transmembrane pr
1.1.1.95	K00058	GO:001659	AT4G34200	EDA9	D-3-phosphoglycerate dehydrogenase
1.1.1.95	K00058	GO:001659	AT4G34200	EDA9	D-3-phosphoglycerate dehydrogenase
			AT1G02120	VAD1	GRAM domain family protein
59:SF32			AT2G44670.1		Protein of unknown function (DUF581)
35:SF11		GO:000635	AT2G44940.1		Integrase-type DNA-binding superfami
11:SF258		GO:001688	AT2G37360.1		ABC-2 type transporter family protei
			AT2G46140.1		Late embryogenesis abundant protein
92:SF25		GO:000635	AT3G61850	DAG1	Dof-type zinc finger DNA-binding fam
77:SF24		GO:000716	AT2G46710.1		Rho GTPase activating protein with P
59:SF29		GO:004698	AT2G46810.1		basic helix-loop-helix (bHLH) DNA-bi
3.4.21.92	K01358		AT1G02560	CLPP5,NCL	nuclear encoded CLP protease 5
3.2.1.37		GO:000597	AT1G02640	ATBXL2,BX	beta-xylosidase 2
75:SF1			AT4G02425.1		
11:SF64	K16275		AT1G02860	BAH1,NLA	SPX (SYG1/Pho81/XPR1) domain-contain
	K02982		ATMG00090.1		structural constituent of ribosome;p

1.8.1.9	K03676	GO:004545	AT3G62930.1	Thioredoxin superfamily protein
3.6.3.14		GO:003317	ATCG00480.1	ATP synthase subunit beta
1.6.5.3	K03879		ATMG01320.1	NADH dehydrogenase 2B
1.6.5.3	K03879		ATMG01320.1	NADH dehydrogenase 2B
1.6.5.3	K03879		ATMG01320.1	NADH dehydrogenase 2B
1.6.5.3	K03879		ATMG01320.1	NADH dehydrogenase 2B
1.6.5.3	K03879		ATMG01320.1	NADH dehydrogenase 2B
1.6.5.3	K03879		ATMG01320.1	NADH dehydrogenase 2B
1.6.5.3	K03879		ATMG01320.1	NADH dehydrogenase 2B
1.6.5.3	K03879		ATMG01320.1	NADH dehydrogenase 2B
1.6.5.3	K03879		ATMG01320.1	NADH dehydrogenase 2B
1.6.5.3	K03879		ATMG01320.1	NADH dehydrogenase 2B
1.6.5.3	K03879		ATMG01320.1	NADH dehydrogenase 2B
1.6.5.3	K03879		ATMG01320.1	NADH dehydrogenase 2B
1.6.5.3	K03879		ATMG01320.1	NADH dehydrogenase 2B
1.6.5.3	K03879		ATMG01320.1	NADH dehydrogenase 2B
1.6.5.3	K03879		ATMG01320.1	NADH dehydrogenase 2B
1.6.5.3	K03879		ATMG01320.1	NADH dehydrogenase 2B
1.6.5.3	K03879		ATMG01320.1	NADH dehydrogenase 2B
1.6.5.3	K03879		ATMG01320.1	NADH dehydrogenase 2B
1.6.5.3	K03879		ATMG01320.1	NADH dehydrogenase 2B
1.6.5.3	K03879		ATMG01320.1	NADH dehydrogenase 2B
1.6.5.3	K03879		ATMG01320.1	NADH dehydrogenase 2B
1.6.5.3	K03879		ATMG01320.1	NADH dehydrogenase 2B
1.6.5.3	K03879		ATMG01320.1	NADH dehydrogenase 2B
1.6.5.3	K03879		ATMG01320.1	NADH dehydrogenase 2B
1.6.5.3	K03879		ATMG01320.1	NADH dehydrogenase 2B
1.6.5.3	K03879		ATMG01320.1	NADH dehydrogenase 2B
1.6.5.3	K03879		ATMG01320.1	NADH dehydrogenase 2B
1.6.5.3	K03879		ATMG01320.1	NADH dehydrogenase 2B
1.6.5.3	K03879		ATMG01320.1	NADH dehydrogenase 2B
1.6.5.3	K03879		ATMG01320.1	NADH dehydrogenase 2B
1.6.5.3	K03879		ATMG01320.1	NADH dehydrogenase 2B
1.6.5.3	K03879		ATMG01320.1	NADH dehydrogenase 2B
1.6.5.3	K03879		ATMG01320.1	NADH dehydrogenase 2B
1.6.5.3	K03879		ATMG01320.1	NADH dehydrogenase 2B
1.6.5.3	K03879		ATMG01320.1	NADH dehydrogenase 2B
1.6.5.3	K03879		ATMG01320.1	NADH dehydrogenase 2B
1.6.5.3	K03879		ATMG01320.1	NADH dehydrogenase 2B
1.6.5.3	K03879		ATMG01320.1	NADH dehydrogenase 2B
1.6.5.3	K03879		ATMG01320.1	NADH dehydrogenase 2B
1.6.5.3	K03879		ATMG01320.1	NADH dehydrogenase 2B
1.6.5.3	K03879		ATMG01320.1	NADH dehydrogenase 2B
1.6.5.3	K03879		ATMG01320.1	NADH dehydrogenase 2B
1.6.5.3	K03879		ATMG01320.1	NADH dehydrogenase 2B
1.6.5.3	K03879		ATMG01320.1	NADH dehydrogenase 2B
1.6.5.3	K03879		ATMG01320.1	NADH dehydrogenase 2B
1.6.5.3	K03879		ATMG01320.1	NADH dehydrogenase 2B

1.6.5.3	K03879		ATMG01320.1		NADH dehydrogenase 2B
1.6.5.3	K03879		ATMG01320.1		NADH dehydrogenase 2B
1.6.5.3	K03879		ATMG01320.1		NADH dehydrogenase 2B
1.6.5.3	K03879		ATMG01320.1		NADH dehydrogenase 2B
1.6.5.3	K03879		ATMG01320.1		NADH dehydrogenase 2B
1.6.5.3	K03879		ATMG01320.1		NADH dehydrogenase 2B
1.6.5.3	K03879		ATMG01320.1		NADH dehydrogenase 2B
1.6.5.3	K03879		ATMG01320.1		NADH dehydrogenase 2B
1.6.5.3	K03879		ATMG01320.1		NADH dehydrogenase 2B
1.6.5.3	K03879		ATMG01320.1		NADH dehydrogenase 2B
1.6.5.3	K03879		ATMG01320.1		NADH dehydrogenase 2B
1.6.5.3	K03879		ATMG01320.1		NADH dehydrogenase 2B
1.6.5.3	K03879		ATMG01320.1		NADH dehydrogenase 2B
1.6.5.3	K03879		ATMG01320.1		NADH dehydrogenase 2B
1.6.5.3	K03879		ATMG01320.1		NADH dehydrogenase 2B
1.6.5.3	K03879		ATMG01320.1		NADH dehydrogenase 2B
1.6.5.3	K03879		ATMG01320.1		NADH dehydrogenase 2B
1.6.5.3	K03879		ATMG01320.1		NADH dehydrogenase 2B
1.6.5.3	K03879		ATMG01320.1		NADH dehydrogenase 2B
1.6.5.3	K03879		ATMG01320.1		NADH dehydrogenase 2B
1.6.5.3	K03879		ATMG01320.1		NADH dehydrogenase 2B
1.6.5.3	K03879		ATMG01320.1		NADH dehydrogenase 2B
1.6.5.3	K03879		ATMG01320.1		NADH dehydrogenase 2B
1.6.5.3	K03879		ATMG01320.1		NADH dehydrogenase 2B
			AT2G42620	MAX2,ORE9	RNI-like superfamily protein
3.1.1.3		GO:001678	AT2G42990.1		GDSL-like Lipase/Acylhydrolase super
76:SF3			AT3G63090.1		Ubiquitin carboxyl-terminal hydrolas
76:SF3			AT3G63090.1		Ubiquitin carboxyl-terminal hydrolas
		GO:004687	AT1G04990.2		Zinc finger C-x8-C-x5-C-x3-H type fa
)5:SF2			AT1G05065	CLE20	CLAVATA3/ESR-RELATED 20
29:SF10		GO:004356	AT1G62300	ATWRKY6,W	WRKY family transcription factor
3.1.13.5	K12951	GO:000562	AT2G32415.1		Polynucleotidyl transferase, ribonuc
1.1.1.317			AT1G60690.1		NAD(P)-linked oxidoreductase superfa
50:SF1			AT3G25130.1		
50:SF1			AT3G25130.1		
55:SF99		GO:004698	AT3G07340.1		basic helix-loop-helix (bHLH) DNA-bi
55:SF99		GO:004698	AT3G07340.1		basic helix-loop-helix (bHLH) DNA-bi
55:SF99		GO:004698	AT3G07340.1		basic helix-loop-helix (bHLH) DNA-bi
55:SF99		GO:004698	AT3G07340.1		basic helix-loop-helix (bHLH) DNA-bi
54:SF132	K14638	GO:001602	AT5G46050	ATPTR3,PT	peptide transporter 3

)3:SF32			AT3G50700	AtIDD2,ID	indeterminate(ID)-domain 2
)3:SF32			AT3G50700	AtIDD2,ID	indeterminate(ID)-domain 2
l8:SF19			AT1G14440	AtHB31,HB	homeobox protein 31
l8:SF19			AT1G14440	AtHB31,HB	homeobox protein 31
l8:SF19			AT1G14440	AtHB31,HB	homeobox protein 31
l8:SF19			AT1G14440	AtHB31,HB	homeobox protein 31
1.14.13.89		GO:005511	AT4G37370	CYP81D8	cytochrome P450, family 81, subfamil
3.1.1.11	K01051	GO:000485	AT4G33230.1		Plant invertase/pectin methylesteras
3.1.1.11	K01051	GO:000485	AT4G33230.1		Plant invertase/pectin methylesteras
			AT1G15780.1		
			AT1G15780.1		
39:SF11		GO:000635	AT2G02450	anac034,A	NAC domain containing protein 35
.2:SF81			ATCG00360.1		Tetratricopeptide repeat (TPR)-like
.2:SF81			ATCG00360.1		Tetratricopeptide repeat (TPR)-like
.2:SF81			ATCG00360.1		Tetratricopeptide repeat (TPR)-like
.2:SF81			ATCG00360.1		Tetratricopeptide repeat (TPR)-like
.2:SF81			ATCG00360.1		Tetratricopeptide repeat (TPR)-like
.2:SF81			ATCG00360.1		Tetratricopeptide repeat (TPR)-like
.2:SF81			ATCG00360.1		Tetratricopeptide repeat (TPR)-like
.2:SF81			ATCG00360.1		Tetratricopeptide repeat (TPR)-like
.2:SF81			ATCG00360.1		Tetratricopeptide repeat (TPR)-like
1.97.1.12		GO:001602	ATCG00350.1		Photosystem I, PsaA/PsaB protein
1.97.1.12		GO:001602	ATCG00350.1		Photosystem I, PsaA/PsaB protein
1.97.1.12		GO:001602	ATCG00350.1		Photosystem I, PsaA/PsaB protein
1.97.1.12		GO:001602	ATCG00350.1		Photosystem I, PsaA/PsaB protein
38:SF3	K02913	GO:000641	ATCG00640.1		ribosomal protein L33
38:SF3	K02913	GO:000641	ATCG00640.1		ribosomal protein L33
38:SF3	K02913	GO:000641	ATCG00640.1		ribosomal protein L33
		GO:000367	AT1G68840	EDF2,RAP2	related to ABI3/VP1 2
3.1.1.11		GO:004254	AT5G47500.1		Pectin lyase-like superfamily protei
3.1.1.32		GO:000662	AT2G42690.1		alpha/beta-Hydrolases superfamily pr
3.1.1.32		GO:000662	AT2G42690.1		alpha/beta-Hydrolases superfamily pr
2.1.1.229	K10770		AT1G31600.3		RNA-binding (RRM/RBD/RNP motifs) fam
2.1.1.229	K10770		AT1G31600.3		RNA-binding (RRM/RBD/RNP motifs) fam
)1:SF2		GO:000508	AT1G31650	ATROPGEF1	RHO guanyl-nucleotide exchange facto
.8:SF9			AT4G39730.1		Lipase/lipooxygenase, PLAT/LH2 famil
)6:SF6			AT5G61460	ATRAD18,M	P-loop containing nucleoside triphos
4.2.1.33	K01704	GO:000815	AT2G43090.1		Aconitase/3-isopropylmalate dehydrat
'4:SF20	K14488	GO:000973	AT4G22620.1		SAUR-like auxin-responsive protein f
3.6.4.4		GO:000551	AT1G12430	ARK3,PAK	armadillo repeat kinesin 3
l0:SF21	K03549	GO:007180	AT4G23640	ATKT3,KUP	Potassium transporter family protein
1.3.1.3		GO:005066	AT4G24220	AWI31,VEP	NAD(P)-binding Rossmann-fold superfa
38:SF48	K07374	GO:000701	AT5G19770	TUA3	tubulin alpha-3
)0:SF2			AT1G64385.1		
3.1.1.3		GO:001678	AT4G10950.1		SGNH hydrolase-type esterase superfa
)1:SF15			AT1G31320	LBD4	LOB domain-containing protein 4
)1:SF15			AT1G31320	LBD4	LOB domain-containing protein 4
3.4.24.85	K07765	GO:000650	AT4G20310.2		Peptidase M50 family protein
59:SF3			AT5G13200.1		GRAM domain family protein

59:SF3			AT5G13200.1		GRAM domain family protein
30:SF1					
30:SF1					
2.7.11.1		GO:000551	AT2G33170.1		Leucine-rich repeat receptor-like pr
			AT5G10970.1		C2H2 and C2HC zinc fingers superfami
	K03626		AT3G12390.1		Nascent polypeptide-associated compl
		GO:000716	AT3G14470.1		NB-ARC domain-containing disease res
		GO:000716	AT3G14470.1		NB-ARC domain-containing disease res
34:SF1			AT3G54500.1		
34:SF1			AT3G54500.1		
2.7.11.17		GO:000551	AT2G38800.1		Plant calmodulin-binding protein-rel
2.7.11.17		GO:000551	AT2G38800.1		Plant calmodulin-binding protein-rel
.5:SF384			AT4G20740.1		Pentatricopeptide repeat (PPR-like)
2.7.11.1		GO:000646	AT3G20830.1		AGC (cAMP-dependent, cGMP-dependent
2.7.11.1		GO:000646	AT3G20830.1		AGC (cAMP-dependent, cGMP-dependent
2.7.11.1		GO:000646	AT3G20830.1		AGC (cAMP-dependent, cGMP-dependent
1.14.12.20		GO:005511	AT4G25650	ACD1-LIKE	ACD1-like
2.7.11.1		GO:000646	AT4G23180	CRK10,RLK	cysteine-rich RLK (RECEPTOR-like pro
28:SF1			AT1G11120.2		
28:SF1			AT1G11120.2		
36:SF7			AT1G28270	RALFL4	ralf-like 4
21:SF0			AT4G18425.1		Protein of unknown function (DUF679)
26:SF12		GO:000575	AT5G02050.1		Mitochondrial glycoprotein family pr
59:SF1			AT2G31081	CLE4	CLAVATA3/ESR-RELATED 4
2.7.11.1		GO:000646	AT1G29750	RKF1	receptor-like kinase in flowers 1
4.1.1.15	K01580	GO:003017	AT5G17330	GAD,GAD1	glutamate decarboxylase
08:SF1			AT5G16550.1		
56:SF3		GO:000551	AT5G16220.1		Octicosapeptide/Phox/Bem1p family pr
12:SF1			AT5G16200.1		50S ribosomal protein-related
12:SF1			AT5G16200.1		50S ribosomal protein-related
12:SF1			AT5G16200.1		50S ribosomal protein-related
12:SF1			AT5G16200.1		50S ribosomal protein-related
)1:SF2			AT5G38760.1		Late embryogenesis abundant protein
)1:SF2			AT5G38760.1		Late embryogenesis abundant protein
)5:SF2	K03142	GO:000827	AT1G05055	ATGTF2H2,	general transcription factor II H2
)1:SF1			AT5G08060.1		
	K09422		AT5G62470	ATMYB96,M	myb domain protein 96
	K09422		AT5G62470	ATMYB96,M	myb domain protein 96
59:SF1		GO:000641	AT5G39785.1		Protein of unknown function (DUF1666
78:SF4			ATCG01280.1		Chloroplast Ycf2;ATPase, AAA type, c
5.1.3.3	K01785	GO:001685	AT3G47800.1		Galactose mutarotase-like superfamil
5.1.3.3	K01785	GO:001685	AT3G47800.1		Galactose mutarotase-like superfamil
5.1.3.3	K01785	GO:001685	AT3G47800.1		Galactose mutarotase-like superfamil
5.1.3.3	K01785	GO:001685	AT3G47800.1		Galactose mutarotase-like superfamil
5.1.3.3	K01785	GO:001685	AT3G47800.1		Galactose mutarotase-like superfamil
5.1.3.3	K01785	GO:001685	AT3G47800.1		Galactose mutarotase-like superfamil
1.2.4.1	K00162	GO:000815	AT2G34590.1		Transketolase family protein
	K04798	GO:005108	AT1G29990	PFD6	prefoldin 6
2.7.11.1		GO:000646	AT5G35370.1		S-locus lectin protein kinase family
2.7.11.1		GO:000646	AT5G35370.1		S-locus lectin protein kinase family
		GO:004687	AT4G23882.1		Heavy metal transport/detoxification
		GO:004687	AT4G23882.1		Heavy metal transport/detoxification

3.1.1.23			AT2G18360.1		alpha/beta-Hydrolases superfamily pr
2.5.1.21	K00801	GO:001674	AT4G34640	ERG9,SQS1	squalene synthase 1
'4:SF40	K14488	GO:000973	AT5G10990.1		SAUR-like auxin-responsive protein f
'4:SF40	K14488	GO:000973	AT5G10990.1		SAUR-like auxin-responsive protein f
	K09872	GO:001602	AT3G61430	ATPIP1,PI	plasma membrane intrinsic protein 1A
	K09872	GO:001602	AT3G61430	ATPIP1,PI	plasma membrane intrinsic protein 1A
	K01426	GO:001688	AT4G34880.1		Amidase family protein
2.7.12.1		GO:000646	AT4G38470.1		ACT-like protein tyrosine kinase fam
2.7.12.1		GO:000646	AT4G38470.1		ACT-like protein tyrosine kinase fam
2.7.12.1		GO:000646	AT4G38470.1		ACT-like protein tyrosine kinase fam
2.7.11.1		GO:000646	AT3G44610.1		Protein kinase superfamily protein
2.7.11.1	K08835	GO:000646	AT4G14480.1		Protein kinase superfamily protein
i9:SF3			ATCG01080.1		NADH:ubiquinone/plastoquinone oxidor
i6:SF3	K00413	GO:002003	AT5G40810.1		Cytochrome C1 family
		GO:005153	AT5G49940	ATCNFU2,N	NIFU-like protein 2
	K13146	GO:003203	AT3G07530.1		
1.1.1.49	K00036	GO:005511	AT1G09420	G6PD4	glucose-6-phosphate dehydrogenase 4
2.3.1.98		GO:001678	AT5G03980.1		SGNH hydrolase-type esterase superfa
2.3.1.98		GO:001678	AT5G03980.1		SGNH hydrolase-type esterase superfa
2.3.1.98		GO:001678	AT5G03980.1		SGNH hydrolase-type esterase superfa
2.3.1.23	K13511	GO:001674	AT3G05510.1		Phospholipid/glycerol acyltransferas
3.2.1.14		GO:0005975,GO:0004553			
3.2.1.14		GO:0005975,GO:0004553			
	K06170		AT5G09310.1		
)4:SF379			AT5G10200.1		ARM-repeat/Tetratricopeptide repeat
i7:SF9	K09286	GO:000635	AT1G78080	RAP2.4	related to AP2 4
	K13628		AT1G10500	ATCPISCA,	chloroplast-localized ISCA-like prot
			AT4G39390	ATNST-KT1	nucleotide sugar transporter-KT 1
3.4.21.92	K01358		AT5G23140	CLPP2,NCL	nuclear-encoded CLP protease P7
4.2.3.22,	K15803	GO:001682	AT5G23960	ATTPS21,T	terpene synthase 21
.7:SF9		GO:000551	AT3G12890	ASML2	activator of spomin::LUC2
)2:SF59	K18757		AT4G35890.1		winged-helix DNA-binding transcripti
)3:SF52			AT4G36945.1		PLC-like phosphodiesterases superfam
)3:SF31			AT5G67130.1		PLC-like phosphodiesterases superfam
1.14.13.89		GO:005511	AT4G37370	CYP81D8	cytochrome P450, family 81, subfamil
2.7.11.1	K03097	GO:000646	AT2G23070.1		Protein kinase superfamily protein
2.7.11.1	K03097	GO:000646	AT2G23070.1		Protein kinase superfamily protein
2.3.1.48,2.3.1.88		GO:000808	AT2G23060.1		Acyl-CoA N-acyltransferases (NAT) su
6.4.1.2	K01963		ATCG00500.1		acetyl-CoA carboxylase carboxyl tran
!4:SF2					
!4:SF2					
!4:SF2					
!4:SF2					
!4:SF2					
!4:SF2					
!4:SF2					
!4:SF2					
!4:SF2					
!4:SF2					
!4:SF2					
!4:SF2					

01:SF21	K02886	GO:000641	ATCG00830.1		ribosomal protein L2
3.6.3.14	K02110	GO:003317	ATCG00140.1		ATP synthase subunit C family protei
3.6.3.14	K02110	GO:003317	ATCG00140.1		ATP synthase subunit C family protei
	K09338	GO:004356	AT4G40060	ATHB-16,A	homeobox protein 16
59:SF9			AT1G22160.1		Protein of unknown function (DUF581)
59:SF9			AT1G22160.1		Protein of unknown function (DUF581)
34:SF19		GO:000367	AT1G77850	ARF17	auxin response factor 17
2.7.1.68			AT1G77660.1		Histone H3 K4-specific methyltransfe
	K16365		AT4G08320.1		Tetratricopeptide repeat (TPR)-like
15:SF135		GO:000551	AT1G77460.2		Armadillo/beta-catenin-like repeat ;
79:SF14			AT1G77160.1		Protein of unknown function (DUF506)
2.4.2.8	K00760	GO:000911	AT1G71750	HGPT	Hypoxanthine-guanine phosphoribosylt
1.13.11.2	K00457	GO:005511	AT1G06570	HPD,PDS1	phytoene desaturation 1
		GO:000662	AT3G63200	PLA IIIB,	PATATIN-like protein 9
		GO:000662	AT3G63200	PLA IIIB,	PATATIN-like protein 9
1.6.5.3			ATMG00580.1		NADH dehydrogenase subunit 4
23:SF15		GO:004698	AT4G02590	UNE12	basic helix-loop-helix (bHLH) DNA-bi
55:SF12			AT2G31160	LSH3	Protein of unknown function (DUF640)
14:SF4		GO:000635	AT4G28530	anac074,N	NAC domain containing protein 74
14:SF4		GO:000635	AT4G28530	anac074,N	NAC domain containing protein 74
2.7.11.1	K08790	GO:000646	AT2G20470.1		AGC (cAMP-dependent, cGMP-dependent
14:SF249		GO:008002	AT1G19715.3		Mannose-binding lectin superfamily p
14:SF249		GO:008002	AT1G19715.3		Mannose-binding lectin superfamily p
04:SF6			AT1G75520	SRS5	SHI-related sequence 5
04:SF6			AT1G75520	SRS5	SHI-related sequence 5
	K02155	GO:003317	AT1G19910	ATVHA-C2,	ATPase, F0/V0 complex, subunit C pro
2.7.11.1,	K14500	GO:000646	AT4G35230	BSK1	BR-signaling kinase 1
2.7.11.1,	K14500	GO:000646	AT4G35230	BSK1	BR-signaling kinase 1
31:SF16			AT3G22142.1		Bifunctional inhibitor/lipid-transfe
1.4.7.1	K00284	GO:005511	AT5G04140	FD-GOGAT,	glutamate synthase 1
23:SF6		GO:000704	AT4G05440	EDA35	temperature sensing protein-related
23:SF6		GO:000704	AT4G05440	EDA35	temperature sensing protein-related
3.4.24.56	K01408		AT2G41790.1		Insulinase (Peptidase family M16) fa
			AT3G57440.1		
1.6.5.3			AT3G57785.1		
1.6.5.3			AT3G57785.1		
77:SF42			AT3G18670.1		Ankyrin repeat family protein
77:SF42			AT3G18670.1		Ankyrin repeat family protein
77:SF42			AT3G18670.1		Ankyrin repeat family protein
		GO:000551	AT3G15880	TPR4,WSIP	WUS-interacting protein 2
		GO:000551	AT2G19330	PIRL6	plant intracellular ras group-relate
	K03217	GO:005120	AT5G62050	ATOXA1,OX	homolog of yeast oxidase assembly 1
	K09422	GO:001033	AT2G37630	AS1,ATMYB	myb-like HTH transcriptional regulat
57:SF9			AT2G37640	ATEXP3,AT	Barwin-like endoglucanases superfami
57:SF9			AT2G37640	ATEXP3,AT	Barwin-like endoglucanases superfami
1.14.11.19		GO:005511	AT5G05600.1		2-oxoglutarate (2OG) and Fe(II)-depe
1.14.11.19		GO:005511	AT5G05600.1		2-oxoglutarate (2OG) and Fe(II)-depe
20:SF5			AT3G08640.1		Protein of unknown function (DUF3411
20:SF5			AT3G08640.1		Protein of unknown function (DUF3411
20:SF5			AT3G08640.1		Protein of unknown function (DUF3411
4.3.1.19	K01754		AT3G10050	OMR1	L-O-methylthreonine resistant 1

55:SF4			AT1G32700.1		PLATZ transcription factor family pr
14:SF0	K14310	GO:000564	AT5G51200.1		Protein of unknown function (DUF3414
2.7.11.1		GO:000646	AT3G09780	ATCRR1,CC	CRINKLY4 related 1
55:SF157		GO:004698	AT5G56960.1		basic helix-loop-helix (bHLH) DNA-bi
55:SF157		GO:004698	AT5G56960.1		basic helix-loop-helix (bHLH) DNA-bi
55:SF157		GO:004698	AT5G56960.1		basic helix-loop-helix (bHLH) DNA-bi
2.1.1.125	K11434	GO:000816	AT4G29510	ATPRMT11,	arginine methyltransferase 11
1.1.3.9			AT5G19580.1		glyoxal oxidase-related protein
38:SF4			AT2G20080.1		
34:SF3					
2.7.7.48	K11699	GO:000396	AT2G19930.1		RNA-dependent RNA polymerase family
			AT5G57520	ATZFP2,ZF	zinc finger protein 2
32:SF0			AT3G46550	SOS5	Fasciclin-like arabinogalactan famil
24:SF5			AT4G30410.2		sequence-specific DNA binding transc
24:SF5			AT4G30410.2		sequence-specific DNA binding transc
24:SF5			AT4G30410.2		sequence-specific DNA binding transc
			AT2G23940.1		Protein of unknown function (DUF788)
2.7.11.1		GO:000716	AT4G30960	ATCIPK6,C	SOS3-interacting protein 3
2.7.11.1		GO:000716	AT4G30960	ATCIPK6,C	SOS3-interacting protein 3
6.3.2.19	K04506	GO:000727	AT3G58040	SINAT2	seven in absentia of Arabidopsis 2
6.3.2.19	K04506	GO:000727	AT3G58040	SINAT2	seven in absentia of Arabidopsis 2
6.3.2.19		GO:001656	AT3G52450	PUB22	plant U-box 22
23:SF20	K06966		AT5G06300.1		Putative lysine decarboxylase family
23:SF20	K06966		AT5G06300.1		Putative lysine decarboxylase family
23:SF20	K06966		AT5G06300.1		Putative lysine decarboxylase family
2.7.11.17			AT3G52710.1		
3.6.4.13	K03257	GO:001678	AT3G13920	EIF4A1,RH	eukaryotic translation initiation fa
03:SF13	K03093	GO:001698	AT3G53920	SIG3,SIGC	RNApolymerase sigma-subunit C
	K09338	GO:000367	AT4G32880	ATHB-8,AT	homeobox gene 8
	K09338	GO:000367	AT4G32880	ATHB-8,AT	homeobox gene 8
	K09338	GO:000367	AT4G32880	ATHB-8,AT	homeobox gene 8
3.4.16.2		GO:000823	AT5G22860.2		Serine carboxypeptidase S28 family p
1.8.4.11	K07304	GO:005511	AT2G18030.1		Peptide methionine sulfoxide reducta
3.1.1.26		GO:000662	AT2G26560	PLA IIA,P	phospholipase A 2A
75:SF6		GO:000814	AT3G50620.1		P-loop containing nucleoside triphos
15:SF166	K09419	GO:004356	AT4G36990	AT-HSFB1,	heat shock factor 4
1.14.13.89		GO:005511	AT4G37370	CYP81D8	cytochrome P450, family 81, subfamil
1.14.13.89		GO:005511	AT4G37370	CYP81D8	cytochrome P450, family 81, subfamil
3.1.3.16		GO:000382	AT4G33920.1		Protein phosphatase 2C family protei
26:SF1			AT3G51280.1		Tetratricopeptide repeat (TPR)-like
42:SF18	K08472	GO:001602	AT2G17480	ATMLO8,ML	Seven transmembrane MLO family prote
		GO:000551	AT4G35470	PIRL4	plant intracellular ras group-relate
	K09422		AT1G79180	ATMYB63,M	myb domain protein 63

	K09422		AT1G79180	ATMYB63,M	myb domain protein 63
2.4.1.25	K00705	GO:000597	AT5G64860	DPE1	disproportionating enzyme
55:SF175	K16282	GO:000827	AT1G72310	ATL3	RING/U-box superfamily protein
24:SF2			AT5G65120.1		
1.8.3.5,1	K05906	GO:000173	AT5G63910	FCLY	farnesylcysteine lyase
39:SF4			AT4G33440.1		Pectin lyase-like superfamily protei
2.1.1.43		GO:000551	AT5G09790	ATXR5,SDG	ARABIDOPSIS TRITHORAX-RELATED PROTEI
3.1.4.46		GO:000646	AT1G66980	SNC4	suppressor of npr1-1 constitutive 4
.2:SF1			AT2G20570	ATGLK1,GL	GBF\'s pro-rich region-interacting f
.7:SF172		GO:000716	AT5G36930.2		Disease resistance protein (TIR-NBS-
.7:SF172		GO:000716	AT5G36930.2		Disease resistance protein (TIR-NBS-
1.3.1.74	K07119	GO:005511	AT5G16970	AER,AT-AE	alkenal reductase
31:SF1			AT5G22930.1		Protein of unknown function (DUF1635
31:SF1			AT5G22930.1		Protein of unknown function (DUF1635
	K07889	GO:000552	AT3G54840	ARA-6,ARA	Ras-related small GTP-binding family
54:SF15			AT3G12345.1		
3.1.3.16	K14497	GO:000382	AT3G11410	AHG3,ATPP	protein phosphatase 2CA
	K03248	GO:000367	AT3G11400	ATEIF3G1,	eukaryotic translation initiation fa
	K03248	GO:000367	AT3G11400	ATEIF3G1,	eukaryotic translation initiation fa
	K07904	GO:000552	AT1G07410	ATRAB-A2B	RAB GTPase homolog A2B
00:SF2			AT5G05840.1		Protein of unknown function (DUF620)
00:SF2			AT5G05840.1		Protein of unknown function (DUF620)
3.5.1.52			AT5G05480.1		Peptide-N4-(N-acetyl-beta-glucosamin
01:SF4			AT2G30130	ASL5,LBD1	Lateral organ boundaries (LOB) domai
1.1.1.205		GO:005511	AT1G16350.1		Aldolase-type TIM barrel family prot
	K18586	GO:000674	AT2G03690.1		coenzyme Q biosynthesis Coq4 family
	K18586	GO:000674	AT2G03690.1		coenzyme Q biosynthesis Coq4 family
	K09264	GO:000635	AT2G03710	AGL3,SEP4	K-box region and MADS-box transcript
	K09264	GO:000635	AT2G03710	AGL3,SEP4	K-box region and MADS-box transcript
6.3.2.19	K10581		AT3G15355	PFU1,UBC2	ubiquitin-conjugating enzyme 25
6.3.2.19	K10581		AT3G15355	PFU1,UBC2	ubiquitin-conjugating enzyme 25
			AT3G25905	CLE27	CLAVATA3/ESR-RELATED 27
			AT1G68360.1		C2H2 and C2HC zinc fingers superfami
38:SF15		GO:000960	AT1G14930.1		Polyketide cyclase/dehydrase and lip
38:SF15		GO:000960	AT1G14930.1		Polyketide cyclase/dehydrase and lip
.3:SF4			AT1G60010.1		
.3:SF4			AT1G60010.1		
02:SF2					
3.6.4.1	K10357	GO:001645	AT5G43900	ATMYA2,MY	myosin 2
6.3.2.19		GO:001656	AT1G24330.1		ARM repeat superfamily protein
6.3.2.19		GO:001656	AT1G24330.1		ARM repeat superfamily protein
	K03217	GO:005120	AT1G24490	ALB4,ARTE	OxaA/YidC-like membrane insertion pr
3.2.1.15		GO:000597	AT3G26610.1		Pectin lyase-like superfamily protei
	K09874	GO:001602	AT3G06100	NIP7;1,NL	NOD26-like intrinsic protein 7;1
51:SF2			AT1G09520.1		
2.4.2.39	K08238	GO:001675	AT4G02500	ATXT2,XT2	UDP-xylosyltransferase 2
2.4.2.39	K08238	GO:001675	AT4G02500	ATXT2,XT2	UDP-xylosyltransferase 2
1.8.1.9	K03676	GO:004545	AT5G18600.1		Thioredoxin superfamily protein
1.8.1.9	K03676	GO:004545	AT5G18600.1		Thioredoxin superfamily protein
1.8.1.9	K03676	GO:004545	AT5G18600.1		Thioredoxin superfamily protein

1.8.1.9	K03676	GO:004545	AT5G18600.1		Thioredoxin superfamily protein
		GO:004687	AT5G18550.1		Zinc finger C-x8-C-x5-C-x3-H type fa
			AT4G15830.1		ARM repeat superfamily protein
			AT4G15830.1		ARM repeat superfamily protein
33:SF1					
36:SF2			AT2G01050.1		zinc ion binding;nucleic acid bindin
36:SF2			AT2G01050.1		zinc ion binding;nucleic acid bindin
71:SF8	K02635	GO:001649	ATCG00720.1		photosynthetic electron transfer B
		GO:004687	AT5G60800.2		Heavy metal transport/detoxification
		GO:004687	AT5G60800.2		Heavy metal transport/detoxification
		GO:004687	AT5G60800.2		Heavy metal transport/detoxification
	K02724	GO:004254	ATCG00300.1		YCF9
	K02724	GO:004254	ATCG00300.1		YCF9
	K02724	GO:004254	ATCG00300.1		YCF9
	K02724	GO:004254	ATCG00300.1		YCF9
	K02724	GO:004254	ATCG00300.1		YCF9
	K02724	GO:004254	ATCG00300.1		YCF9
	K02724	GO:004254	ATCG00300.1		YCF9
	K02724	GO:004254	ATCG00300.1		YCF9
3.1.3.16		GO:000382	AT1G07630	PLL5	pol-like 5
4.2.1.70	K16329	GO:001679	AT1G50510.1		indigoidine synthase A family protei
2.7.1.1	K00844	GO:001677	AT1G50460	ATHKL1,HK	hexokinase-like 1
1.5.1.43		GO:005511	AT1G50450.1		Saccharopine dehydrogenase
			AT5G13800	CRN1,PPH	pheophytinase
1.14.99.43		GO:005511	AT5G42580	CYP705A12	cytochrome P450, family 705, subfami
26:SF3			AT5G19380	CLT1	CRT (chloroquine-resistance transpor
			AT2G23970.1		Class I glutamine amidotransferase-l
	K13692	GO:001675	AT2G23260	UGT84B1	UDP-glucosyl transferase 84B1
3.6.4.4	K11498	GO:000801	AT4G39050.1		Kinesin motor family protein
06:SF116			AT2G21340.1		MATE efflux family protein
74:SF40	K14488	GO:000973	AT1G75590.1		SAUR-like auxin-responsive protein f
74:SF40	K14488	GO:000973	AT1G75590.1		SAUR-like auxin-responsive protein f
18:SF0			AT1G20550.1		O-fucosyltransferase family protein
93:SF27			AT1G08290	WIP3	WIP domain protein 3
2.4.1.32	K13680		AT5G22740	ATCSLA02,	cellulose synthase-like A02
2.4.1.32	K13680		AT5G22740	ATCSLA02,	cellulose synthase-like A02
27:SF156			AT2G27140.1		HSP20-like chaperones superfamily pr
3.2.1.58	K01188	GO:000597	AT5G20950.2		Glycosyl hydrolase family protein
2.10.1.1,	K15376	GO:003232	AT5G20990	B73,CHL6,	molybdopterin biosynthesis CNX1 prot
3.5.1.98	K06067		AT4G38130	ATHD1,ATH	histone deacetylase 1
16:SF1			AT3G06790.2		plastid developmental protein DAG, p
78:SF224			AT5G27240.1		DNAJ heat shock N-terminal domain-co
	K10523	GO:000551	AT3G06190	ATBPM2,BP	BTB-POZ and MATH domain 2

2.7.11.1	K17545	GO:000646	AT5G18700	EMB3013,R	Protein kinase family protein with A
2.7.11.1	K17545	GO:000646	AT5G18700	EMB3013,R	Protein kinase family protein with A
2.7.11.1	K17545	GO:000646	AT5G18700	EMB3013,R	Protein kinase family protein with A
2.7.11.1	K17545	GO:000646	AT5G18700	EMB3013,R	Protein kinase family protein with A
2.7.11.1	K17545	GO:000646	AT5G18700	EMB3013,R	Protein kinase family protein with A
2.7.11.1	K17545	GO:000646	AT5G18700	EMB3013,R	Protein kinase family protein with A
30:SF4	K02705	GO:001968	ATCG00280.1		photosystem II reaction center prote
76:SF1			AT3G06035.1		Glycoprotein membrane precursor GPI-
16:SF5		GO:000372	AT1G23400	ATCAF2,CA	RNA-binding CRS1 / YhbY (CRM) domain
53:SF3			ATCG01130.1		Ycf1 protein
53:SF3			ATCG01130.1		Ycf1 protein
			AT1G13195.1		RING/U-box superfamily protein
			AT1G13195.1		RING/U-box superfamily protein
		GO:003101	AT5G43500	ARP9,ATAR	actin-related protein 9
08:SF34		GO:000551	AT3G02400.1		SMAD/FHA domain-containing protein
32:SF4	K02692	GO:001597	AT1G03130	PSAD-2	photosystem I subunit D-2
3.1.26.4			AT1G24090.1		RNase H family protein
18:SF61		GO:001602	AT1G70260.1		nodulin MtN21 /EamA-like transporter
2.4.1.15,	K16055	GO:000599	AT1G68020	ATTPS6,TP	UDP-Glycosyltransferase / trehalose-
2.4.1.15,	K16055	GO:000599	AT1G68020	ATTPS6,TP	UDP-Glycosyltransferase / trehalose-
	K09338	GO:004356	AT2G01430	ATHB-17,A	homeobox-leucine zipper protein 17
52:SF12	K13947	GO:005508	AT1G70940	ATPIN3,PI	Auxin efflux carrier family protein
52:SF12	K13947	GO:005508	AT1G70940	ATPIN3,PI	Auxin efflux carrier family protein
2.4.2.19		GO:001676	AT2G01350	QPT	quinolinate phoshoribosyltransferase
3.1.3.4			AT3G02600	ATLPP3,LP	lipid phosphate phosphatase 3
3.5.1.94			AT5G38200.1		Class I glutamine amidotransferase-l
97:SF9			AT1G26170.1		ARM repeat superfamily protein
			AT3G17210	ATHS1,HS1	heat stable protein 1
			AT1G48110	ECT7	evolutionarily conserved C-terminal
			AT1G69530	AT-EXP1,A	expansin A1
15:SF44			AT2G02980	OTP85	Pentatricopeptide repeat (PPR) super
2.7.11.1		GO:000551	AT3G51740	IMK2	inflorescence meristem receptor-like
2.7.11.1		GO:000646	AT3G55950	ATCRR3,CC	CRINKLY4 related 3
56:SF4			AT2G39870.1		
2.1.1.157		GO:000816	AT2G39750.1		S-adenosyl-L-methionine-dependent me
17:SF9			AT3G12560	ATTBP2,TR	TRF-like 9
2.7.7.59		GO:001659	AT2G39570.1		ACT domain-containing protein
		GO:000367	AT3G54770.1		RNA-binding (RRM/RBD/RNP motifs) fam
		GO:000367	AT3G54770.1		RNA-binding (RRM/RBD/RNP motifs) fam
		GO:000367	AT3G54770.1		RNA-binding (RRM/RBD/RNP motifs) fam
71:SF194		GO:004356	AT3G60530	GATA4	GATA transcription factor 4
71:SF194		GO:004356	AT3G60530	GATA4	GATA transcription factor 4
1.14.11.27			AT5G04240	ELF6	Zinc finger (C2H2 type) family prote
19:SF2					
19:SF2					
2.7.11.1	K04733		AT5G38280	PR5K	PR5-like receptor kinase
2.7.1.90	K00895	GO:000609	AT1G12000.1		Phosphofructokinase family protein
53:SF3			ATCG01130.1		Ycf1 protein
71:SF2	K02637	GO:001649	ATCG00730.1		photosynthetic electron transfer D
71:SF2	K02637	GO:001649	ATCG00730.1		photosynthetic electron transfer D
	K02709	GO:005082	ATCG00710.1		photosystem II reaction center prote
78:SF4			ATCG01280.1		Chloroplast Ycf2;ATPase, AAA type, c

'8:SF4			ATCG01280.1		Chloroplast Ycf2;ATPase, AAA type, c
	K02992,K05573		ATCG00890.1		NADH-Ubiquinone/plastoquinone (compl
	K02992,K05573		ATCG00890.1		NADH-Ubiquinone/plastoquinone (compl
30:SF88		GO:001602	AT4G18260.1		Cytochrome b561/ferric reductase tra
	K13162	GO:000372	AT5G46190.1		RNA-binding KH domain-containing pro
32:SF27	K10609	GO:003162	AT5G46210	ATCUL4,CU	cullin4
	K14827		AT5G06350.1		ARM repeat superfamily protein
	K14827		AT5G06350.1		ARM repeat superfamily protein
)4:SF1			AT3G05650	AtRLP32,R	receptor like protein 32
.1:SF2	K02718	GO:001602	ATCG00690.1		photosystem II reaction center prote
39:SF1			AT5G53800.1		
35:SF7		GO:001602	AT5G54860.1		Major facilitator superfamily protei
6.3.2.19	K19042		AT3G12920.1		SBP (S-ribonuclease binding protein)
			AT1G33055.1		
3.6.1.1	K01514	GO:0016462,GO:0005737			
1.3.3.8		GO:005511	AT4G20820.1		FAD-binding Berberine family protein
3.1.4.46		GO:000646	AT1G66920.2		Protein kinase superfamily protein
31:SF1			AT5G24490.1		30S ribosomal protein, putative
2.1.1.43		GO:000551	AT5G24330	ATXR6,SDG	ARABIDOPSIS TRITHORAX-RELATED PROTEI
11:SF563			AT5G52260	AtMYB19,M	myb domain protein 19
		GO:000551	AT1G71400	AtRLP12,R	receptor like protein 12
		GO:000551	AT1G71400	AtRLP12,R	receptor like protein 12
2.7.11.1	K13412	GO:000646	AT5G24430.1		Calcium-dependent protein kinase (CD
21:SF0			AT2G01930	ATBPC1,BB	basic pentacysteine1
3.1.1.23			AT1G73480.1		alpha/beta-Hydrolases superfamily pr
)8:SF7			AT1G79770.1		Protein of unknown function (DUF1677
1.14.11.4			AT3G18210.1		2-oxoglutarate (2OG) and Fe(II)-depe
56:SF12		GO:000551	AT2G01190.1		Octicosapeptide/Phox/Bem1p family pr
	K09264	GO:004698	AT5G60910	AGL8,FUL	AGAMOUS-like 8
	K09264	GO:004698	AT5G60910	AGL8,FUL	AGAMOUS-like 8
38:SF8	K12840	GO:000367	AT1G30480	DRT111	D111/G-patch domain-containing prote
32:SF10	K11274	GO:000551	AT3G42660.1		transducin family protein / WD-40 re
32:SF10	K11274	GO:000551	AT3G42660.1		transducin family protein / WD-40 re
32:SF10	K11274	GO:000551	AT3G42660.1		transducin family protein / WD-40 re
55:SF107		GO:004698	AT5G62610.1		basic helix-loop-helix (bHLH) DNA-bi
6.3.3.2	K01934		AT5G13050	5-FCL	5-formyltetrahydrofolate cycloligase
3.1.3.16	K04382	GO:001678	AT1G10430	PP2A-2	protein phosphatase 2A-2
2.4.1.15,	K16055	GO:000599	AT1G23870	ATTPS9,TP	trehalose-phosphatase/synthase 9
2.4.1.15,	K16055	GO:000599	AT1G23870	ATTPS9,TP	trehalose-phosphatase/synthase 9
)2:SF14		GO:000635	AT5G62940	DOF5.6,HC	Dof-type zinc finger DNA-binding fam
	K09422		AT5G26660	ATMYB86,M	myb domain protein 86
'5:SF3			AT1G56280	ATDI19,DI	drought-induced 19
1.14.13.121		GO:005511	AT3G26330	CYP71B37	cytochrome P450, family 71, subfamil
15:SF1		GO:000551	AT4G24820.2		26S proteasome, regulatory subunit R
12:SF19	K07195	GO:000688	AT5G50380	ATEXO70F1	exocyst subunit exo70 family protein
)8:SF7			AT1G67340.1		HCP-like superfamily protein with MY
)8:SF7			AT1G67340.1		HCP-like superfamily protein with MY
3.5.4.12	K01493	GO:000827	AT3G48540.1		Cytidine/deoxycytidylate deaminase f
3.5.4.12	K01493	GO:000827	AT3G48540.1		Cytidine/deoxycytidylate deaminase f
1.6.5.3			AT3G48680	GAMMA CAL	gamma carbonic anhydrase-like 2
3.4.24.23		GO:003101	AT1G24140.1		Matrixin family protein
55:SF90		GO:004698	AT5G50915.1		basic helix-loop-helix (bHLH) DNA-bi

3.4.25.1		GO:005160	AT1G16470	PAB1	proteasome subunit PAB1
		GO:005153	AT5G51720.1		2 iron, 2 sulfur cluster binding
	K03294	GO:001602	AT4G21120	AAT1,CAT1	amino acid transporter 1
26:SF51			AT5G62170.1		
		GO:000635	AT5G62165	AGL42	AGAMOUS-like 42
52:SF141			AT5G51930.1		Glucose-methanol-choline (GMC) oxido
2.7.7.6	K03014	GO:000635	AT5G51940	NRPB6A,NR	RNA polymerase Rpb6
26:SF288	K09338	GO:000367	AT4G00730	AHDP,ANL2	Homeobox-leucine zipper family prote
	K02920	GO:000641	AT3G53740.3		Ribosomal protein L36e family protei
	K03327	GO:005508	AT4G25640	ATDTX35,D	detoxifying efflux carrier 35
	K07953	GO:000726	AT4G02080	ASAR1,ATS	secretion-associated RAS super famil
		GO:000551	AT5G27030	TPR3	TOPLESS-related 3
36:SF5			AT1G02900	ATRALF1,R	rapid alkalinization factor 1
2.7.11.1		GO:000646	AT1G54820.1		Protein kinase superfamily protein
3.2.1.51		GO:001678	AT1G54790.3		GDSL-like Lipase/Acylhydrolase super
30:SF39		GO:001602	AT1G64780	AMT1;2,AT	ammonium transporter 1;2
2.4.1.255	K09667		AT3G04240	SEC	Tetratricopeptide repeat (TPR)-like
3.6.1.5	K14641	GO:001678	AT5G18280	APY2,ATAP	apyrase 2
			AT2G44900	ARABIDILL	ARABIDILLO-1
3.6.4.12		GO:0006281,GO:0003678,GO:0000723			
1.6.99.5	K05579	GO:005511	ATCG01110.1		NAD(P)H dehydrogenase subunit H
1.6.99.5	K05579	GO:005511	ATCG01110.1		NAD(P)H dehydrogenase subunit H
1.6.99.5	K05579	GO:005511	ATCG01110.1		NAD(P)H dehydrogenase subunit H
1.6.99.5	K05579	GO:005511	ATCG01110.1		NAD(P)H dehydrogenase subunit H
32:SF7	K05572	GO:005511	ATCG01100.1		NADH dehydrogenase family protein
32:SF7		GO:005511	ATCG01100.1		NADH dehydrogenase family protein
59:SF3	K05578	GO:005511	ATCG01080.1		NADH:ubiquinone/plastoquinone oxidor
53:SF3			ATCG01130.1		Ycf1 protein
03:SF0	K03505	GO:000626	AT1G09815	POLD4	polymerase delta 4
			AT1G65320.1		Cystathionine beta-synthase (CBS) fa
			AT1G65320.1		Cystathionine beta-synthase (CBS) fa
	K15747	GO:005511	AT1G31800	CYP97A3,L	cytochrome P450, family 97, subfamil
2.7.11.1	K07198	GO:000646	AT3G01090	AKIN10,KI	SNF1 kinase homolog 10
2.7.11.1	K07198	GO:000646	AT3G01090	AKIN10,KI	SNF1 kinase homolog 10
79:SF17			AT4G32480.1		Protein of unknown function (DUF506)
08:SF3			AT1G71480.1		Nuclear transport factor 2 (NTF2) fa
01:SF12			AT2G18420.1		Gibberellin-regulated family protein
2.7.11.1		GO:004854	AT1G34300.1		lectin protein kinase family protein
72:SF13			AT3G15030	MEE35,TCP	TCP family transcription factor 4
72:SF13			AT3G15030	MEE35,TCP	TCP family transcription factor 4
16:SF99		GO:004698	AT4G35150.1		O-methyltransferase family protein
57:SF4			AT3G59670.1		
			AT1G03670.1		ankyrin repeat family protein
71:SF2			ATCG00730.1		photosynthetic electron transfer D
71:SF2			ATCG00730.1		photosynthetic electron transfer D
1.10.9.1		GO:002290	ATCG00720.1		photosynthetic electron transfer B
	K02992	GO:000641	ATCG01240.1		ribosomal protein S7

2.7.4.1			ATCG01010.1	NADH-Ubiquinone oxidoreductase (comp
2.7.4.1			ATCG01010.1	NADH-Ubiquinone oxidoreductase (comp
'1:SF8		GO:002003	ATCG01040.1	Cytochrome C assembly protein
'1:SF8		GO:002003	ATCG01040.1	Cytochrome C assembly protein
'1:SF8		GO:002003	ATCG01040.1	Cytochrome C assembly protein
'1:SF8		GO:002003	ATCG01040.1	Cytochrome C assembly protein
1.6.99.5			ATCG01110.1	NAD(P)H dehydrogenase subunit H
3:SF3			ATCG01130.1	Ycf1 protein
3:SF3			ATCG01130.1	Ycf1 protein
16:SF2				
		GO:000641	ATCG00780.1	ribosomal protein L14
6.4.1.2			ATCG00500.1	acetyl-CoA carboxylase carboxyl tran
6.4.1.2			ATCG00500.1	acetyl-CoA carboxylase carboxyl tran
1:SF9	K02986	GO:000372	ATCG00380.1	chloroplast ribosomal protein S4
1:SF9	K02986	GO:000372	ATCG00380.1	chloroplast ribosomal protein S4
2:SF81			ATCG00360.1	Tetratricopeptide repeat (TPR)-like
1.97.1.12	K02689	GO:001602	ATCG00350.1	Photosystem I, PsaA/PsaB protein
1.97.1.12	K02689	GO:001602	ATCG00350.1	Photosystem I, PsaA/PsaB protein
	K02714	GO:001968	ATCG00220.1	photosystem II reaction center prote
	K02714	GO:001968	ATCG00220.1	photosystem II reaction center prote
	K02714	GO:001968	ATCG00220.1	photosystem II reaction center prote

	K02714	GO:001968	ATCG00220.1	photosystem II reaction center prote
		GO:003317	ATCG00140.1	ATP synthase subunit C family protei
		GO:003317	ATCG00140.1	ATP synthase subunit C family protei
1.10.3.9	K02703	GO:004515	ATCG00020.1	photosystem II reaction center prote
78:SF4		GO:000552	ATCG00860.1	Chloroplast Ycf2;ATPase, AAA type, c
78:SF4		GO:000552	ATCG00860.1	Chloroplast Ycf2;ATPase, AAA type, c
78:SF4		GO:000552	ATCG00860.1	Chloroplast Ycf2;ATPase, AAA type, c
78:SF4		GO:000552	ATCG00860.1	Chloroplast Ycf2;ATPase, AAA type, c
78:SF4		GO:000552	ATCG00860.1	Chloroplast Ycf2;ATPase, AAA type, c
78:SF4		GO:000552	ATCG00860.1	Chloroplast Ycf2;ATPase, AAA type, c
3.1.3.16		GO:000382	AT4G03415.2	Protein phosphatase 2C family protei
3.1.3.16		GO:000382	AT4G03415.2	Protein phosphatase 2C family protei
2.7.11.1	K12761	GO:000716	AT2G30360CIPK11,PK	SOS3-interacting protein 4
56:SF6			AT5G49350.1	Glycine-rich protein family
2.7.11.1		GO:000551	AT1G06840.1	Leucine-rich repeat protein kinase f
35:SF3		GO:000828	AT3G49780ATPSK3 (F	phytosulfokine 4 precursor
.9:SF52	K14611	GO:005508	AT1G65550.1	Xanthine/uracil permease family prot
.9:SF52	K14611	GO:005508	AT1G65550.1	Xanthine/uracil permease family prot
	K18667	GO:000551	AT1G27752.1	Ubiquitin system component Cue prote
	K18667	GO:000551	AT1G27752.1	Ubiquitin system component Cue prote
26:SF263		GO:000367	AT1G46480WOX4	WUSCHEL related homeobox 4
26:SF263		GO:000367	AT1G46480WOX4	WUSCHEL related homeobox 4
3.4.24.36	K01404	GO:001602	AT5G42620.2	metalloendopeptidases;zinc ion bindi
			AT4G31980.1	
49:SF2			AT3G60470.1	Plant protein of unknown function (D
74:SF175			AT2G45120.1	C2H2-like zinc finger protein
3.1.16.1	K15338	GO:000628	AT1G01880.1	5\'-3\' exonuclease family protein
1.1.1.39	K00028	GO:005511	AT4G00570NAD-ME2	NAD-dependent malic enzyme 2
3.5.4.9,1.5.1.5		GO:005511	AT4G00620.1	Amino acid dehydrogenase family prot
2.4.1.273		GO:001675	AT2G22590.1	UDP-Glycosyltransferase superfamily
2.7.11.24	K04371	GO:000646	AT4G01370ATMPK4,MP	MAP kinase 4
		GO:000827	AT1G71980.1	Protease-associated (PA) RING/U-box
2.7.11.1	K13412	GO:001677	AT2G46700ATCRK3,CR	CDPK-related kinase 3
24:SF13			AT2G47270.1	sequence-specific DNA binding transc
3.6.4.12	K02210	GO:000626	AT4G02060MCM7,PRL	Minichromosome maintenance (MCM2/3/5
3.6.4.12	K02210	GO:000626	AT4G02060MCM7,PRL	Minichromosome maintenance (MCM2/3/5
2.7.11.1		GO:000551	AT1G03440.1	Leucine-rich repeat (LRR) family pro
2.7.11.1		GO:000551	AT1G03440.1	Leucine-rich repeat (LRR) family pro
2.3.1.74	K00660	GO:001674	AT5G13930ATCHS,CHS	Chalcone and stilbene synthase famil
2.3.1.74	K00660	GO:001674	AT5G13930ATCHS,CHS	Chalcone and stilbene synthase famil
			AT4G22250.1	RING/U-box superfamily protein
			AT4G22250.1	RING/U-box superfamily protein
3.1.1.3		GO:001678	AT2G42990.1	GDSL-like Lipase/Acylhydrolase super
.2:SF126		GO:000551	AT1G16930.1	F-box/RNI-like/FBD-like domains-cont
.2:SF126		GO:000551	AT1G16930.1	F-box/RNI-like/FBD-like domains-cont
2.7.11.1,	K14500	GO:000646	AT5G46570BSK2	BR-signaling kinase 2
			AT5G66730.1	C2H2-like zinc finger protein
3.2.1.39		GO:000597	AT2G16230.1	O-Glycosyl hydrolases family 17 prot
5.3.1.24		GO:000656	AT5G05590PAI2	phosphoribosylanthranilate isomerase
3.2.1.26		GO:003392	AT1G35580CINV1	cytosolic invertase 1
3.2.1.26		GO:003392	AT1G35580CINV1	cytosolic invertase 1
3.2.1.26		GO:003392	AT1G35580CINV1	cytosolic invertase 1

3.2.1.26		GO:003392	AT1G35580	CINV1	cytosolic invertase 1
3.2.1.26		GO:003392	AT1G35580	CINV1	cytosolic invertase 1
	K10801	GO:000628	AT3G07930.3		DNA glycosylase superfamily protein
	K10801	GO:000628	AT3G07930.3		DNA glycosylase superfamily protein
	K17985	GO:000551	AT1G04140.1		Transducin family protein / WD-40 re
		GO:000551	AT2G20210.1		RNI-like superfamily protein
2.3.1.158	K00679	GO:000837	AT1G04010	ATPSAT1,P	phospholipid sterol acyl transferase
3.1.1.31	K01057	GO:000597	AT5G24400	EMB2024,P	NagB/RpiA/CoA transferase-like super
3.2.1.14	K01183	GO:000597	AT5G24090	ATCHIA,CH	chitinase A
3.6.4.13	K12818	GO:000552	AT3G26560.1		ATP-dependent RNA helicase, putative
)1:SF34	K11593	GO:000551	AT1G48410	AGO1	Stabilizer of iron transporter SufD
2.4.2.1	K07375	GO:000392	AT5G12250	TUB6	beta-6 tubulin
2.4.1.255	K18207	GO:001675	AT3G18170.1		Glycosyltransferase family 61 protei
1.14.11.4			AT3G18210.2		2-oxoglutarate (2OG) and Fe(II)-depe
3.4.25.1	K11866	GO:000551	AT1G48790	AMSH1	associated molecule with the SH3 dom
55:SF153		GO:004698	AT5G08130	BIM1	basic helix-loop-helix (bHLH) DNA-bi
55:SF153		GO:004698	AT5G08130	BIM1	basic helix-loop-helix (bHLH) DNA-bi
55:SF153		GO:004698	AT5G08130	BIM1	basic helix-loop-helix (bHLH) DNA-bi
55:SF153		GO:004698	AT5G08130	BIM1	basic helix-loop-helix (bHLH) DNA-bi
55:SF153		GO:004698	AT5G08130	BIM1	basic helix-loop-helix (bHLH) DNA-bi
55:SF153		GO:004698	AT5G08130	BIM1	basic helix-loop-helix (bHLH) DNA-bi
55:SF153		GO:004698	AT5G08130	BIM1	basic helix-loop-helix (bHLH) DNA-bi
55:SF153		GO:004698	AT5G08130	BIM1	basic helix-loop-helix (bHLH) DNA-bi
55:SF153		GO:004698	AT5G08130	BIM1	basic helix-loop-helix (bHLH) DNA-bi
.0:SF0		GO:001598	ATMG00410.1		ATPase subunit 6-1
.0:SF0		GO:001598	ATMG00410.1		ATPase subunit 6-1
.0:SF0		GO:001598	ATMG00410.1		ATPase subunit 6-1
.0:SF0		GO:001598	ATMG00410.1		ATPase subunit 6-1
.0:SF0		GO:001598	ATMG00410.1		ATPase subunit 6-1
.0:SF0		GO:001598	ATMG00410.1		ATPase subunit 6-1
1.6.5.3	K03883		ATMG00513.1		NADH dehydrogenase 5A
1.6.5.3	K03883		ATMG00513.1		NADH dehydrogenase 5A
1.6.5.3	K03883		ATMG00513.1		NADH dehydrogenase 5A
1.6.5.3	K03883		ATMG00513.1		NADH dehydrogenase 5A

3.4.23.12		GO:000650	AT3G18490.1		Eukaryotic aspartyl protease family
3.4.23.12		GO:000650	AT3G18490.1		Eukaryotic aspartyl protease family
)6:SF43			AT4G22730.1		Leucine-rich repeat protein kinase f
5.2.1.8		GO:000645	AT1G74070.1		Cyclophilin-like peptidyl-prolyl cis
36:SF2			AT2G01050.1		zinc ion binding;nucleic acid bindin
2.7.11.22,2.7.11.2		GO:000646	AT1G18670	IBS1	Protein kinase superfamily protein
2.7.11.22,2.7.11.2		GO:000646	AT1G18670	IBS1	Protein kinase superfamily protein
4.2.2.2	K01728	GO:003057	AT5G15110.1		Pectate lyase family protein
)8:SF1			AT5G50335.1		
15:SF2		GO:004698	AT3G26744	ATICE1,IC	basic helix-loop-helix (bHLH) DNA-bi
53:SF99		GO:000552	AT5G18820	EMB3007	TCP-1/cpn60 chaperonin family protei
		GO:000552	AT5G18820	EMB3007	TCP-1/cpn60 chaperonin family protei
.8:SF8		GO:000551	AT5G62200.1		Embryo-specific protein 3, (ATS3)
3.1.16.1	K15338	GO:000628	AT3G48900.2		single-stranded DNA endonuclease fam
57:SF1			AT5G37010.1		
26:SF283		GO:001675	AT3G21760	HYR1	UDP-Glycosyltransferase superfamily
	K03145	GO:000635	AT2G42730.1		F-box family protein
			AT5G13650.2		elongation factor family protein
)6:SF14			AT2G41660	MIZ1	Protein of unknown function, DUF617
6.3.2.19	K04506	GO:000727	AT3G58040	SINAT2	seven in absentia of Arabidopsis 2
)5:SF71			AT1G58170.1		Disease resistance-responsive (dirig
3.6.4.13	K14442	GO:000552	AT2G35920.1		RNA helicase family protein
2.7.11.17			AT3G52710.1		
)8:SF2		GO:001659	AT5G02850.1		hydroxyproline-rich glycoprotein fam
			AT2G37060	NF-YB8	nuclear factor Y, subunit B8
1.97.1.12	K02690,K0	GO:001602	ATCG00350.1		Photosystem I, PsaA/PsaB protein
24:SF3			AT3G56710	SIB1	sigma factor binding protein 1
38:SF3	K02634	GO:003136	ATCG00540.1		photosynthetic electron transfer A
56:SF45			AT5G24270	ATSOS3,CB	Calcium-binding EF-hand family prote
3.1.3.66		GO:004257	AT3G51830	ATG5,SAC8	SAC domain-containing protein 8
		GO:000551	AT1G45616	AtRLP6,RL	receptor like protein 6
3.1.4.46		GO:003024	AT5G38260.1		Protein kinase superfamily protein
			AT3G07565.1		Protein of unknown function (DUF3755
			AT3G60470.1		Plant protein of unknown function (D

.9:SF3		GO:000635	AT4G27410	ANAC072,R	NAC (No Apical Meristem) domain tran
'1:SF186		GO:004356	AT3G21175	GATA24,TI	ZIM-like 1
'1:SF186		GO:004356	AT3G21175	GATA24,TI	ZIM-like 1
10:SF10		GO:000367	AT3G26790	FUS3	AP2/B3-like transcriptional factor f
37:SF4	K00891		AT3G26900	ATSKL1,SK	shikimate kinase like 1
2.7.7.6			ATCG00170.1		DNA-directed RNA polymerase family p
2.7.7.6			ATCG00170.1		DNA-directed RNA polymerase family p
2.1.1.43			AT5G14260.2		Rubisco methyltransferase family pro
1.14.11.2	K00472	GO:005511	AT3G28480.1		Oxoglutarate/iron-dependent oxygenas
1.14.11.2	K00472	GO:005511	AT3G28480.1		Oxoglutarate/iron-dependent oxygenas
1.14.11.2	K00472	GO:005511	AT3G28480.1		Oxoglutarate/iron-dependent oxygenas
	K17479	GO:004545	AT5G39865.1		Glutaredoxin family protein
	K17479	GO:004545	AT5G39865.1		Glutaredoxin family protein
	K17479	GO:004545	AT5G39865.1		Glutaredoxin family protein
	K09338	GO:000367	AT5G15150	ATHB-3,AT	homeobox 3
1.10.2.2	K17732		AT3G02090	MPPBETA	Insulinase (Peptidase family M16) pr
1.10.2.2	K17732		AT3G02090	MPPBETA	Insulinase (Peptidase family M16) pr
3.6.3.43		GO:001688	AT5G39040	ALS1,ATTA	transporter associated with antigen
4.1.1.39	K01602		AT1G67090	RBCS1A	ribulose bisphosphate carboxylase sm
		GO:005508	AT5G16150	GLT1,PGLC	plastidic GLC translocator
		GO:005508	AT1G71090.1		Auxin efflux carrier family protein
55:SF443		GO:004353	AT3G14460.1		LRR and NB-ARC domains-containing di
3.4.21.92			ATCG00670.1		plastid-encoded CLP P
3.4.21.92			ATCG00670.1		plastid-encoded CLP P
30:SF8		GO:001968	ATCG00680.1		photosystem II reaction center prote
		GO:004353	AT3G14470.1		NB-ARC domain-containing disease res
1.6.5.3	K03949	GO:002290	AT5G52840.1		NADH-ubiquinone oxidoreductase-relat
'8:SF6			AT2G02230	AtPP2-B1,	phloem protein 2-B1
)1:SF29	K16241	GO:004356	AT5G11260	HY5,TED 5	Basic-leucine zipper (bZIP) transcri
		GO:000827	AT3G15680.1		Ran BP2/NZF zinc finger-like superfa
31:SF12	K02986	GO:000372	ATMG00290.1		mitochondrial ribosomal protein S4
1.14.11.1	K04124	GO:005511	AT1G15550	ATGA3OX1,	gibberellin 3-oxidase 1
1.14.13.1	K11816	GO:005511	AT5G25620	YUC6	Flavin-binding monooxygenase family
1.14.13.1	K11816	GO:005511	AT5G25620	YUC6	Flavin-binding monooxygenase family
55:SF1		GO:001602	AT2G25737.1		Sulfite exporter TauE/SafE family pr
6.3.2.19	K10689		AT5G25760	PEX4,UBC2	peroxin4
	K09338	GO:000367	AT4G32880	ATHB-8,AT	homeobox gene 8
	K09338	GO:000367	AT4G32880	ATHB-8,AT	homeobox gene 8
	K09338	GO:000367	AT4G32880	ATHB-8,AT	homeobox gene 8
	K09338	GO:000367	AT4G32880	ATHB-8,AT	homeobox gene 8
	K02985	GO:000372	AT5G35530.1		Ribosomal protein S3 family protein
	K09422		AT2G16720	ATMYB7,AT	myb domain protein 7
2.7.12.2	K04368		AT4G29810	ATMKK2,MK	MAP kinase kinase 2
3.4.22.34	K01369	GO:000823	AT4G32940	GAMMA-VPE	gamma vacuolar processing enzyme
3.4.22.34	K01369	GO:000823	AT4G32940	GAMMA-VPE	gamma vacuolar processing enzyme
3.4.22.34	K01369	GO:000823	AT4G32940	GAMMA-VPE	gamma vacuolar processing enzyme
3.4.22.34	K01369	GO:000823	AT4G32940	GAMMA-VPE	gamma vacuolar processing enzyme
3.1.3.16	K14497	GO:000382	AT1G72770	HAB1	homology to ABI1

3.1.3.16	K14497	GO:000382	AT1G72770	HAB1	homology to ABI1
3.1.3.16	K14497	GO:000382	AT1G72770	HAB1	homology to ABI1
3.1.3.16	K14497	GO:000382	AT1G72770	HAB1	homology to ABI1
3.1.3.16	K14497	GO:000382	AT1G72770	HAB1	homology to ABI1
3.1.3.16	K14497	GO:000382	AT1G72770	HAB1	homology to ABI1
3.2.1.23		GO:003024	AT5G20710	BGAL7	beta-galactosidase 7
3.2.1.23		GO:003024	AT5G20710	BGAL7	beta-galactosidase 7
70:SF21		GO:000367	AT3G43240.1		ARID/BRIGHT DNA-binding domain-conta
3.1.1.3		GO:001678	AT1G29660.1		GDSL-like Lipase/Acylhydrolase super
3.1.1.3		GO:001678	AT1G29670.1		GDSL-like Lipase/Acylhydrolase super
38:SF94		GO:001953	AT5G57710.1		Double Clp-N motif-containing P-loop
38:SF94		GO:001953	AT5G57710.1		Double Clp-N motif-containing P-loop
38:SF94		GO:001953	AT5G57710.1		Double Clp-N motif-containing P-loop
38:SF94		GO:001953	AT5G57710.1		Double Clp-N motif-containing P-loop
55:SF138		GO:000827	AT2G18670.1		RING/U-box superfamily protein
55:SF138		GO:000827	AT2G18670.1		RING/U-box superfamily protein
1.9.3.1	K02267	GO:000573	AT1G22450	ATCOX6B2,	cytochrome C oxidase 6B
55:SF1			AT3G26390.1		
55:SF497			AT4G27220.1		NB-ARC domain-containing disease res
31:SF4					
39:SF1		GO:004573	AT5G44120	ATCRA1,CR	RmlC-like cupins superfamily protein
48:SF10			AT1G08800.1		Protein of unknown function, DUF593
48:SF10			AT1G08800.1		Protein of unknown function, DUF593
41:SF559	K12860		AT1G09770	ATCDC5,AT	cell division cycle 5
55:SF16			AT5G28490	LSH1	Protein of unknown function (DUF640)
0:SF152		GO:005511	AT2G21840.1		Cysteine/Histidine-rich C1 domain fa
2.7.7.6	K03046		ATCG00170.1		DNA-directed RNA polymerase family p
2.7.7.6	K03046		ATCG00170.1		DNA-directed RNA polymerase family p
2.7.7.6	K03046	GO:000635	ATCG00180.1		DNA-directed RNA polymerase family p
2.7.7.6	K03046	GO:000635	ATCG00180.1		DNA-directed RNA polymerase family p
2.7.7.6	K03046	GO:000635	ATCG00180.1		DNA-directed RNA polymerase family p
2.7.7.6	K03046	GO:000635	ATCG00180.1		DNA-directed RNA polymerase family p
1.97.1.12	K02690,K0	GO:001602	ATCG00350.1		Photosystem I, PsaA/PsaB protein
3.6.3.14	K02110	GO:003317	ATCG00140.1		ATP synthase subunit C family protei
3.6.3.14	K02110	GO:003317	ATCG00140.1		ATP synthase subunit C family protei
	K11346	GO:000551	AT1G54390	ING2	PHD finger protein-related
2.3.2.4			AT3G02910.1		AIG2-like (avirulence induced gene)
2.3.2.4			AT3G02910.1		AIG2-like (avirulence induced gene)
2.3.2.4			AT3G02910.1		AIG2-like (avirulence induced gene)
2.7.4.1			ATCG01010.1		NADH-Ubiquinone oxidoreductase (comp
	K09422		AT1G08810	AtMYB60,M	myb domain protein 60
30:SF4		GO:001968	ATCG00280.1		photosystem II reaction center prote
3.2.2.16		GO:000911	AT4G24340.1		Phosphorylase superfamily protein
		GO:000716	AT5G36930.1		Disease resistance protein (TIR-NBS-
		GO:000716	AT5G36930.1		Disease resistance protein (TIR-NBS-
		GO:000716	AT5G36930.1		Disease resistance protein (TIR-NBS-
		GO:000716	AT5G36930.1		Disease resistance protein (TIR-NBS-
3.2.1.15		GO:000597	AT3G07820.1		Pectin lyase-like superfamily protei

3.2.1.15		GO:000597	AT3G07820.1	Pectin lyase-like superfamily protei
3.2.1.15		GO:000597	AT3G07820.1	Pectin lyase-like superfamily protei
3.2.1.15		GO:000597	AT3G07820.1	Pectin lyase-like superfamily protei
3.2.1.15		GO:000597	AT3G07820.1	Pectin lyase-like superfamily protei
3.2.1.15		GO:000597	AT3G07820.1	Pectin lyase-like superfamily protei
3.2.1.15		GO:000597	AT3G07820.1	Pectin lyase-like superfamily protei
3.2.1.15		GO:000597	AT3G07820.1	Pectin lyase-like superfamily protei
2.4.1.134		GO:001602	AT1G33430.1	Galactosyltransferase family protein
54:SF116	K14638	GO:005508	AT1G22540.1	Major facilitator superfamily protei
54:SF116	K14638	GO:005508	AT1G22540.1	Major facilitator superfamily protei
32:SF38	K15378		AT1G22710ATSUC2,SU	sucrose-proton symporter 2
32:SF38	K15378		AT1G22710ATSUC2,SU	sucrose-proton symporter 2
32:SF38	K15378		AT1G22710ATSUC2,SU	sucrose-proton symporter 2
09:SF3			AT1G28510.1	Optic atrophy 3 protein (OPA3)
39:SF21		GO:000635	AT5G24590ANAC091,T	TCV-interacting protein
			AT4G03500.1	Ankyrin repeat family protein
			AT4G03500.1	Ankyrin repeat family protein
53:SF1		GO:000367	AT4G28190ULT,ULT1	Developmental regulator, ULTRAPETALA
			AT1G06950ATTIC110,	translocon at the inner envelope mem
4.2.3.111		GO:001682	AT3G25810.1	Terpenoid cyclases/Protein prenyltra
4.2.3.111		GO:001682	AT3G25810.1	Terpenoid cyclases/Protein prenyltra
3.6.3.14		GO:003317	ATCG00480.1	ATP synthase subunit beta
3.6.3.14		GO:003317	ATCG00480.1	ATP synthase subunit beta
3.6.3.14		GO:003317	ATCG00480.1	ATP synthase subunit beta
3.6.3.14		GO:003317	ATCG00480.1	ATP synthase subunit beta
3.6.3.14		GO:003317	ATCG00480.1	ATP synthase subunit beta
3.6.3.14		GO:003317	ATCG00480.1	ATP synthase subunit beta
3.6.3.14		GO:003317	ATCG00480.1	ATP synthase subunit beta
3.6.3.14		GO:003317	ATCG00480.1	ATP synthase subunit beta
3.6.3.14		GO:003317	ATCG00480.1	ATP synthase subunit beta
3.6.3.14		GO:003317	ATCG00480.1	ATP synthase subunit beta
1.97.1.12		GO:001602	ATCG00350.1	Photosystem I, PsaA/PsaB protein
	K13459	GO:000551	AT4G10780.1	LRR and NB-ARC domains-containing di
76:SF3			AT5G19590.1	Protein of unknown function, DUF538
51:SF22			AT5G34885.1	Protein of unknown function (DUF784)
30:SF3	K02705	GO:001968	ATCG00280.1	photosystem II reaction center prote
30:SF3	K02705	GO:001968	ATCG00280.1	photosystem II reaction center prote
30:SF3	K02705	GO:001968	ATCG00280.1	photosystem II reaction center prote
30:SF3	K02705	GO:001968	ATCG00280.1	photosystem II reaction center prote
2.7.11.1		GO:004854	AT4G27290.1	S-locus lectin protein kinase family
36:SF2			AT2G01050.1	zinc ion binding;nucleic acid bindin
36:SF2			AT2G01050.1	zinc ion binding;nucleic acid bindin
		GO:0003677		
		GO:0003677		
		GO:0003677		
		GO:0003677		
		GO:0003677		
		GO:0003677		
		GO:0003677		
		GO:0003677		
		GO:0003677		

			AT1G15780.1		
			AT1G15780.1		
			AT1G15780.1		
			AT1G15780.1		
			AT1G15780.1		
			AT1G15780.1		
			AT1G15780.1		
			AT1G15780.1		
2.7.11.1		GO:000551	AT1G07390	AtRLP1,RL	receptor like protein 1
55:SF497		GO:004353	AT4G27220.1		NB-ARC domain-containing disease res
1.11.1.7	K00430	GO:005511	AT4G33420.1		Peroxidase superfamily protein
2.3.1.67,2.3.1.23,		GO:000550	AT4G13440.1		Calcium-binding EF-hand family prote
		GO:004353	AT3G14470.1		NB-ARC domain-containing disease res
		GO:004353	AT3G14470.1		NB-ARC domain-containing disease res
		GO:004353	AT3G14470.1		NB-ARC domain-containing disease res
2.7.7.6	K10908	GO:000635	AT5G15700.1		DNA/RNA polymerases superfamily prot
2.7.7.6	K10908	GO:000635	AT5G15700.1		DNA/RNA polymerases superfamily prot
2.7.4.1	K05577		ATCG01010.1		NADH-Ubiquinone oxidoreductase (comp
		GO:000716	AT4G12010.1		Disease resistance protein (TIR-NBS-
			AT3G49490.1		
34:SF204			AT4G30320.1		CAP (Cysteine-rich secretory protein
34:SF204			AT4G30320.1		CAP (Cysteine-rich secretory protein
2.7.11.1		GO:000551	AT1G74190	AtRLP15,R	receptor like protein 15
			AT3G60470.1		Plant protein of unknown function (D
3.2.1.2		GO:001616	AT4G15210	AT-BETA-A	beta-amylase 5
3.2.1.2		GO:001616	AT4G15210	AT-BETA-A	beta-amylase 5
3.2.1.2		GO:001616	AT4G15210	AT-BETA-A	beta-amylase 5
3.2.1.2		GO:001616	AT4G15210	AT-BETA-A	beta-amylase 5
			AT3G22070.1		proline-rich family protein
53:SF3			ATCG01130.1		Ycf1 protein
)3:SF47			AT2G16640	ATTOC132,	multimeric translocon complex in the
	K14015		AT3G63000	NPL41	NPL4-like protein 1
2.3.1.160		GO:001674	AT1G24430.1		HXXXD-type acyl-transferase family p
)5:SF189		GO:000815	AT1G65880	BZO1	benzoyloxyglucosinolate 1
		GO:000551	AT3G05660	AtRLP33,R	receptor like protein 33
		GO:000551	AT3G05660	AtRLP33,R	receptor like protein 33
			AT4G27700.1		Rhodanese/Cell cycle control phospha
			AT3G02640.1		
33:SF1					
55:SF85			AT1G69010	BIM2	BES1-interacting Myc-like protein 2

protein

superfamily protein
ly protein
nily protein

rotein kinase
rotein kinase

protein

ated protein 2A

protein
factor 5

ng protein
nily protein

ase/nucleotidase

ligase

droxyproline-rich glycoprotein family

family protein
YABBY family protein
YABBY family protein
amily protein
family protein

ase family protein

y A, polypeptide 2

.y A, polypeptide 2

:or family protein
<inase family protein

ein D

.n
.n
ein
.city factor 30
.city factor 30
ess protein (ERD4)
rotein
family protein
ogenase family protein
protein (HISIE)

protein
factor 4
factor 4
factor 4

nel 4

sistance protein
sistance protein

family protein

n
e 1

otein

ntaining protein

.y protein
.y protein
.y protein

sing domain containing protein

: family protein

:otein kinase family protein
:otein kinase family protein

.ly protein
.n

nily protein
'AK-box/P21-Rho-binding domain
.nding superfamily protein

ning protein
protein binding

:family protein
se family protein
se family protein
amily protein

clease H fold protein with HRDC domain
amily protein

.nding superfamily protein
.nding superfamily protein
.nding superfamily protein
.nding superfamily protein

.y D, polypeptide 8
se inhibitor superfamily
se inhibitor superfamily

superfamily protein
superfamily protein
superfamily protein
superfamily protein
superfamily protein
superfamily protein
superfamily protein
superfamily protein
superfamily protein

.n
rotein
rotein
nily protein
nily protein
or 14
.y protein
sphate hydrolases superfamily protein
case protein
family

n
amily protein

amily protein

:otein kinase family protein
.ly protein
.ex (NAC), alpha subunit family protein
;istance protein
;istance protein

.ated
.ated
superfamily protein
and protein kinase C) kinase family protein
and protein kinase C) kinase family protein
and protein kinase C) kinase family protein

)tein kinase) 10

:otein

:otein

(LEA) family protein
(LEA) family protein

;)
:ore
.y protein
.y protein
.y protein
.y protein
.y protein
.y protein

' protein
' protein
ı superfamily protein
ı superfamily protein

:otein

:amily
:amily
\
\

nily protein
nily protein
nily protein

:eductase, chain 6

amily protein
amily protein
amily protein
se family protein

(TPR)-like protein

:ein

.on factor family protein
nily protein
nily protein
.y D, polypeptide 8

aperfamily protein
nsferase subunit beta

.n
.n

erase SET7/9 family protein
superfamily protein
C2 calcium/lipid-binding domain (CaLB) protein

ransferase

nding superfamily protein

and protein kinase C) kinase family protein
protein
protein

otein

r protein/seed storage 2S albumin superfamily protein

amily protein

d LRR 6
(OXA1)

or family protein
ly protein
ly protein
ndent oxygenase superfamily protein
ndent oxygenase superfamily protein
.)
.)
.)

:otein
l)

.nding family protein
.nding family protein
.nding family protein

protein

.y protein
:ription factors
:ription factors
:ription factors

r protein
r protein
r protein

ictor 4A1

protein
ise family protein

sphate hydrolases superfamily protein

.y D, polypeptide 8
.y D, polypeptide 8
.n

superfamily protein

:in
:d LRR 4

.n
[N 5

[actor 1
-LRR class) family
-LRR class) family

5)
5)
r protein

actor 3G1
actor 3G1

nyl)asparagine amidase A protein
.n family protein
tein
protein / ubiquinone biosynthesis Coq4 family protein
protein / ubiquinone biosynthesis Coq4 family protein
tion factor family protein
tion factor family protein

.ly protein
pid transport superfamily protein
pid transport superfamily protein

rotein
.n

ımily protein

ıg
ıg

ı superfamily protein
ı superfamily protein
ı superfamily protein

.n

.ly A, polypeptide 12
:ter)-like transporter 1

.ike superfamily protein

:amily
:amily

:otein

:ein / molybdenum cofactor biosynthesis enzyme CNX1 (CNX1)

ɔutative
ɔntaining protein

ARM repeat domain
ARM repeat domain
ARM repeat domain
ARM repeat domain
ARM repeat domain
ARM repeat domain
ein C
-anchored

n-containing protein

family protein
-phosphatase family protein
-phosphatase family protein

e

like superfamily protein

region 7

family protein
e kinase 2

ethyltransferases superfamily protein

nily protein
nily protein
nily protein

ein / transcription factor jumonji (jmj) family protein

ein H
core

:ore
.ex I) protein
.ex I) protein
ansmembrane protein family
otein

ein T

.n
 family protein

n

N 6

)PK) family protein

rotein
7)
endent oxygenase superfamily protein
rotein

ein
epeat family protein
epeat family protein
epeat family protein
nding superfamily protein
e

nily protein

.y B, polypeptide 37
Rpn7;Proteasome component (PCI) domain
n F1
ZND-type zinc finger
ZND-type zinc finger
family protein
family protein

nding superfamily protein

›reductase family protein

›in / lipid-binding START domain-containing protein
.n

.y 2

rfamily protein

superfamily protein

reductase, chain 6

amily protein
amily protein
.y A, polypeptide 3

amily protein
1
1

lex I), chain 5 protein
lex I), chain 5 protein

sferase subunit beta
sferase subunit beta

superfamily protein

ein M
ein M
ein M

ein M

n
n
ein A
ore
ore
ore
ore
ore
ore
n
n

amily protein

ein
ein
in
in

ng

UF247)

ein
protein

zinc finger family protein

ription factors;transcription regulators
) family protein
) family protein
tein
tein
y protein
y protein

family protein
aining protein
aining protein

ein
2

epeat family protein

e 1
rfamily protein

e
/ Polynucleotidyl transferase

in
endent oxygenase superfamily protein
main of STAM 1
inding superfamily protein
inding superfamily protein
inding superfamily protein
inding superfamily protein
inding superfamily protein
inding superfamily protein
inding superfamily protein
inding superfamily protein
inding superfamily protein

protein
protein
family protein
s-trans isomerase family protein
ng

nding superfamily protein
n
n

nily protein

protein

gent-like protein) family protein

nily protein

ein

5)
DUF247)

ıscriptional regulator superfamily protein

:amily protein

›rotein
›rotein
›tein

se
se
se

:otein
:otein
processing protein 2
nall chain 1A

.sease resistance protein

:in B
sistance protein
:ed

.ption factor family protein
ımily protein

protein
protein
:otein

ıining protein
:family protein
:family protein
› nucleoside triphosphate hydrolases superfamily protein
› nucleoside triphosphate hydrolases superfamily protein
› nucleoside triphosphate hydrolases superfamily protein
› nucleoside triphosphate hydrolases superfamily protein

;istance protein

ı

ımily protein

›rotein
›rotein
›rotein
›rotein
›rotein
›rotein

.n
.n

family protein
family protein
family protein

›lex I), chain 5 protein

›in C

-LRR class) family
-LRR class) family
-LRR class) family
-LRR class) family
.n

.n
.n
.n
.n
.n
.n
.n
ı
.n
.n

\
ıbrane of chloroplasts 110
ınsferases superfamily protein
ınsferases superfamily protein

.sease resistance protein

ein C
ein C
ein C
ein C
' protein
ıg
ıg

sistance protein

ein
sistance protein
sistance protein
sistance protein
tein
tein
plex I), chain 5 protein
-LRR class) family

ns, Antigen 5, and Pathogenesis-related 1 protein) superfamily protein
ns, Antigen 5, and Pathogenesis-related 1 protein) superfamily protein

DUF247)

e outer envelope membrane 132

protein

atase superfamily protein
