# Supplementary material for: Transcription factor PagLBD21 functions as a repressor of secondary xylem development in Populus
Source: For Res (Fayettev). 2022 Dec 21;2:19. doi: 10.48130/FR-2022-0019 (PMC11524276; doi:10.48130/FR-2022-0019)
Supplement: Supplementary file 1 — Supplementary data to this article can be found online. [file FR-2022-0019-S1.zip › 10.48130_FR-2022-0019-Suppl-TableS7.pdf]

**Supplemental Table S7.** The overlapped DEGs in PagLBD21 RNA-seq & PagLBD3 RNA-se

| genes | logFC.x | logCPM.x | PValue.x | FDR.x | OX.WT.LBD | LLJGY.A | LLJGY.B | LLJGY.C |
|---|---|---|---|---|---|---|---|---|
| Potri.001 | -3.13512 | 3.626244 | 3.92E-14 | 4.01E-11 | -1 | 214 | 399 | 228 |
| Potri.001 | -0.84518 | 1.410782 | 0.001171 | 0.039407 | -1 | 47 | 38 | 40 |
| Potri.001 | 1.525735 | 0.918472 | 0.000125 | 0.007107 | 1 | 12 | 7 | 16 |
| Potri.001 | 0.55523 | 7.331455 | 0.000676 | 0.026114 | 1 | 1306 | 1965 | 1772 |
| Potri.001 | -0.75869 | 2.329501 | 0.00016 | 0.008516 | -1 | 79 | 78 | 81 |
| Potri.001 | 1.647527 | 2.300543 | 6.86E-13 | 5.86E-10 | 1 | 37 | 26 | 28 |
| Potri.001 | 0.51275 | 6.153491 | 5.85E-06 | 0.000622 | 1 | 784 | 724 | 779 |
| Potri.001 | 0.654147 | 3.362225 | 0.000204 | 0.010422 | 1 | 92 | 121 | 94 |
| Potri.001 | 0.5732 | 5.439324 | 3.48E-05 | 0.002637 | 1 | 501 | 483 | 376 |
| Potri.001 | 0.47671 | 7.254934 | 0.000656 | 0.025513 | 1 | 1883 | 1392 | 1728 |
| Potri.001 | 0.719771 | 3.519537 | 2.98E-05 | 0.002349 | 1 | 135 | 91 | 110 |
| Potri.001 | -0.58717 | 4.850383 | 0.000316 | 0.014595 | -1 | 394 | 509 | 434 |
| Potri.001 | -0.57131 | 4.041254 | 0.000643 | 0.025104 | -1 | 303 | 192 | 269 |
| Potri.001 | -0.57569 | 4.831037 | 0.000181 | 0.009396 | -1 | 523 | 371 | 434 |
| Potri.001 | -0.95512 | 3.063317 | 1.17E-06 | 0.000167 | -1 | 127 | 130 | 163 |
| Potri.001 | -0.97827 | 3.169961 | 5.89E-06 | 0.000624 | -1 | 141 | 175 | 139 |
| Potri.001 | 0.995222 | 5.430854 | 5.81E-09 | 1.91E-06 | 1 | 393 | 356 | 374 |
| Potri.001 | 0.691354 | 4.032509 | 6.81E-07 | 0.000107 | 1 | 165 | 160 | 160 |
| Potri.001 | -0.79479 | 3.241011 | 1.26E-05 | 0.001156 | -1 | 156 | 152 | 151 |
| Potri.001 | -0.46957 | 4.776263 | 0.000134 | 0.007472 | -1 | 444 | 403 | 389 |
| Potri.001 | -0.54611 | 5.968187 | 0.000527 | 0.021384 | -1 | 852 | 1112 | 913 |
| Potri.001 | 1.005888 | 2.519795 | 5.26E-05 | 0.003688 | 1 | 45 | 62 | 38 |
| Potri.001 | -0.79283 | 4.083285 | 4.34E-08 | 1.05E-05 | -1 | 286 | 256 | 288 |
| Potri.001 | -0.63718 | 5.290515 | 1.10E-05 | 0.001027 | -1 | 769 | 591 | 502 |
| Potri.001 | 0.476094 | 4.644343 | 0.000258 | 0.012517 | 1 | 310 | 246 | 259 |
| Potri.001 | 0.814917 | 4.48763 | 0.000113 | 0.006532 | 1 | 280 | 156 | 201 |
| Potri.001 | -0.49661 | 4.130852 | 0.0004 | 0.017437 | -1 | 302 | 233 | 260 |
| Potri.001 | 0.755798 | 6.44516 | 1.65E-07 | 3.27E-05 | 1 | 1084 | 629 | 837 |
| Potri.001 | 0.566291 | 5.865582 | 4.06E-06 | 0.000461 | 1 | 727 | 520 | 594 |
| Potri.001 | -0.54976 | 4.871831 | 2.29E-05 | 0.00192 | -1 | 466 | 438 | 446 |
| Potri.001 | 0.707213 | 6.579802 | 5.96E-06 | 0.000629 | 1 | 764 | 1096 | 952 |
| Potri.001 | -0.56195 | 4.096389 | 4.57E-05 | 0.003314 | -1 | 294 | 233 | 263 |
| Potri.001 | -1.46901 | 1.099163 | 1.24E-06 | 0.000173 | -1 | 43 | 30 | 40 |
| Potri.001 | 0.816923 | 6.304792 | 3.27E-09 | 1.19E-06 | 1 | 721 | 841 | 664 |
| Potri.001 | 0.985569 | 3.380787 | 7.27E-08 | 1.62E-05 | 1 | 96 | 82 | 92 |
| Potri.001 | 0.382987 | 5.760247 | 0.001538 | 0.047254 | 1 | 667 | 618 | 550 |
| Potri.001 | 0.522728 | 7.039502 | 3.34E-06 | 0.000395 | 1 | 1406 | 1348 | 1455 |
| Potri.001 | 0.432314 | 7.627102 | 0.001123 | 0.038114 | 1 | 2284 | 1828 | 2464 |
| Potri.001 | 0.597357 | 5.799112 | 5.57E-07 | 9.02E-05 | 1 | 594 | 589 | 543 |
| Potri.001 | -1.99106 | 5.031189 | 9.95E-20 | 3.29E-16 | -1 | 622 | 787 | 607 |
| Potri.001 | -2.46939 | -0.65892 | 0.000344 | 0.015469 | -1 | 7 | 16 | 8 |
| Potri.001 | -0.57302 | 5.75023 | 5.28E-05 | 0.003691 | -1 | 959 | 770 | 785 |
| Potri.001 | -1.24878 | 5.238923 | 4.14E-07 | 7.00E-05 | -1 | 559 | 749 | 742 |
| Potri.001 | 0.388254 | 6.603889 | 0.001236 | 0.040569 | 1 | 1163 | 1162 | 961 |
| Potri.001 | 1.401929 | 4.1197 | 7.88E-15 | 9.96E-12 | 1 | 147 | 102 | 123 |
| Potri.001 | 0.820627 | 4.471962 | 3.76E-07 | 6.55E-05 | 1 | 270 | 165 | 193 |
| Potri.001 | 0.582783 | 3.504078 | 0.00148 | 0.046018 | 1 | 124 | 96 | 131 |
| Potri.001 | -0.83854 | 4.049635 | 7.56E-09 | 2.34E-06 | -1 | 307 | 239 | 276 |
| Potri.001 | 0.452777 | 3.779103 | 0.001651 | 0.049927 | 1 | 152 | 151 | 145 |
| Potri.001 | -0.50504 | 5.018607 | 7.54E-05 | 0.004884 | -1 | 593 | 440 | 451 |
| Potri.001 | -0.51955 | 5.873937 | 0.000172 | 0.009027 | -1 | 805 | 1019 | 852 |

| | | | | | | | | |
|---|---|---|---|---|---|---|---|---|
| Potri.001 | -0.85923 | 4.969299 | 2.74E-10 | 1.32E-07 | -1 | 507 | 493 | 565 |
| Potri.001 | -0.97416 | 6.029428 | 7.76E-07 | 0.000118 | -1 | 902 | 1297 | 1144 |
| Potri.001 | -0.46319 | 4.520176 | 0.000333 | 0.015167 | -1 | 376 | 334 | 322 |
| Potri.001 | -0.53298 | 3.08634 | 0.001325 | 0.042728 | -1 | 128 | 125 | 131 |
| Potri.001 | 0.507613 | 5.569049 | 2.06E-05 | 0.001756 | 1 | 542 | 501 | 485 |
| Potri.001 | -0.44593 | 4.017667 | 0.001053 | 0.036534 | -1 | 252 | 227 | 243 |
| Potri.001 | -0.84924 | 6.274699 | 4.63E-08 | 1.10E-05 | -1 | 1354 | 1225 | 1301 |
| Potri.001 | 0.614754 | 3.237927 | 0.000362 | 0.016153 | 1 | 117 | 92 | 79 |
| Potri.001 | 0.646784 | 4.457837 | 0.000211 | 0.010722 | 1 | 188 | 275 | 198 |
| Potri.001 | -1.4308 | 0.414602 | 0.001476 | 0.045971 | -1 | 12 | 24 | 30 |
| Potri.001 | -0.87856 | 3.029737 | 0.000284 | 0.013415 | -1 | 125 | 135 | 143 |
| Potri.001 | -0.61114 | 3.032776 | 0.001311 | 0.04237 | -1 | 120 | 147 | 110 |
| Potri.001 | -0.48413 | 5.782281 | 2.44E-05 | 0.002014 | -1 | 871 | 846 | 781 |
| Potri.001 | 0.732351 | 2.73283 | 0.000315 | 0.014589 | 1 | 64 | 63 | 64 |
| Potri.001 | 0.557111 | 6.938344 | 3.27E-06 | 0.000388 | 1 | 1224 | 1321 | 1316 |
| Potri.001 | -1.53774 | 2.348601 | 2.71E-05 | 0.002179 | -1 | 70 | 119 | 94 |
| Potri.001 | -2.41269 | -0.49038 | 0.000116 | 0.006644 | -1 | 15 | 14 | 7 |
| Potri.001 | 0.735158 | 5.876774 | 3.99E-07 | 6.86E-05 | 1 | 514 | 675 | 521 |
| Potri.001 | -1.26445 | 1.164938 | 0.001602 | 0.048792 | -1 | 38 | 50 | 26 |
| Potri.001 | -0.61748 | 2.506131 | 0.00156 | 0.047688 | -1 | 82 | 89 | 89 |
| Potri.001 | -1.09081 | 3.592691 | 6.86E-10 | 2.89E-07 | -1 | 193 | 205 | 231 |
| Potri.001 | -0.9754 | 2.800004 | 7.23E-08 | 1.62E-05 | -1 | 124 | 116 | 111 |
| Potri.001 | 0.731313 | 4.44416 | 2.06E-08 | 5.72E-06 | 1 | 222 | 196 | 218 |
| Potri.001 | -0.49736 | 6.742809 | 2.64E-06 | 0.000326 | -1 | 1740 | 1538 | 1615 |
| Potri.001 | -0.52087 | 5.051128 | 0.000967 | 0.03447 | -1 | 538 | 519 | 461 |
| Potri.001 | 1.028422 | 6.339934 | 6.91E-06 | 0.000705 | 1 | 515 | 961 | 579 |
| Potri.001 | -1.81756 | 0.371985 | 0.000291 | 0.013712 | -1 | 13 | 35 | 20 |
| Potri.001 | -0.50759 | 3.888072 | 0.000761 | 0.028803 | -1 | 258 | 185 | 230 |
| Potri.001 | -0.37551 | 6.817241 | 0.001028 | 0.035839 | -1 | 1936 | 1515 | 1536 |
| Potri.001 | 1.341658 | 1.801856 | 0.000131 | 0.007363 | 1 | 31 | 22 | 21 |
| Potri.001 | 0.447768 | 5.041034 | 0.000209 | 0.010648 | 1 | 370 | 364 | 349 |
| Potri.002 | -0.60142 | 6.238102 | 3.00E-07 | 5.42E-05 | -1 | 1294 | 1146 | 1110 |
| Potri.002 | 0.629985 | 6.569268 | 1.87E-05 | 0.001611 | 1 | 1035 | 901 | 975 |
| Potri.002 | -1.28948 | 1.616772 | 1.60E-06 | 0.000212 | -1 | 55 | 52 | 53 |
| Potri.002 | -0.71892 | 2.876799 | 0.000156 | 0.008383 | -1 | 114 | 100 | 134 |
| Potri.002 | 0.599504 | 5.379166 | 3.80E-07 | 6.59E-05 | 1 | 424 | 424 | 439 |
| Potri.002 | -0.60368 | 4.136589 | 9.60E-05 | 0.005819 | -1 | 245 | 277 | 295 |
| Potri.002 | -0.62827 | 3.077539 | 0.000387 | 0.017029 | -1 | 153 | 116 | 124 |
| Potri.002 | -0.58163 | 6.121173 | 4.54E-06 | 0.000503 | -1 | 1307 | 895 | 1067 |
| Potri.002 | -0.81062 | 2.74085 | 0.000251 | 0.012245 | -1 | 90 | 127 | 105 |
| Potri.002 | -1.00118 | 5.622178 | 5.33E-10 | 2.26E-07 | -1 | 885 | 844 | 825 |
| Potri.002 | -0.60124 | 3.169002 | 0.001041 | 0.036244 | -1 | 175 | 127 | 115 |
| Potri.002 | 0.623793 | 4.087572 | 0.000268 | 0.01288 | 1 | 219 | 144 | 159 |
| Potri.002 | 0.437132 | 5.682819 | 0.000267 | 0.012871 | 1 | 577 | 564 | 558 |
| Potri.002 | -1.18194 | 2.393768 | 3.07E-06 | 0.000368 | -1 | 86 | 113 | 75 |
| Potri.002 | -0.54215 | 4.643873 | 1.77E-05 | 0.00154 | -1 | 411 | 378 | 361 |
| Potri.002 | -0.76727 | 6.419735 | 8.39E-06 | 0.000837 | -1 | 1234 | 1330 | 1620 |
| Potri.002 | 0.864 | 7.432394 | 8.69E-06 | 0.000862 | 1 | 1153 | 1972 | 1602 |
| Potri.002 | -0.95283 | 2.371896 | 1.29E-05 | 0.001178 | -1 | 103 | 73 | 82 |
| Potri.002 | 0.717939 | 3.963313 | 5.60E-07 | 9.03E-05 | 1 | 171 | 137 | 150 |
| Potri.002 | -1.3295 | 1.169068 | 2.52E-05 | 0.002045 | -1 | 46 | 43 | 27 |
| Potri.002 | 0.854925 | 2.880068 | 2.23E-05 | 0.001874 | 1 | 68 | 62 | 71 |
| Potri.002 | 0.471667 | 7.373167 | 6.66E-06 | 0.000687 | 1 | 1858 | 1700 | 1863 |
| Potri.002 | -1.51245 | 1.131792 | 3.38E-05 | 0.002585 | -1 | 27 | 48 | 41 |

| | | | | | | | | |
|---|---|---|---|---|---|---|---|---|
| Potri.002 | -1.03671 | 3.210023 | 6.62E-09 | 2.11E-06 | -1 | 166 | 144 | 166 |
| Potri.002 | 0.768995 | 6.275182 | 1.91E-10 | 9.31E-08 | 1 | 756 | 748 | 728 |
| Potri.002 | -0.68999 | 3.891161 | 1.50E-06 | 0.000201 | -1 | 265 | 215 | 228 |
| Potri.002 | 0.766393 | 6.42309 | 3.40E-09 | 1.22E-06 | 1 | 824 | 780 | 872 |
| Potri.002 | -0.36205 | 6.787399 | 0.001481 | 0.046018 | -1 | 1792 | 1583 | 1479 |
| Potri.002 | -0.94476 | 4.443899 | 7.01E-09 | 2.20E-06 | -1 | 338 | 409 | 357 |
| Potri.002 | -0.46859 | 6.088222 | 0.000107 | 0.006322 | -1 | 1079 | 1062 | 935 |
| Potri.003 | -1.24835 | 1.23633 | 2.87E-05 | 0.002274 | -1 | 46 | 43 | 31 |
| Potri.003 | -0.51647 | 4.126087 | 0.00057 | 0.022728 | -1 | 318 | 232 | 248 |
| Potri.003 | 0.854616 | 2.960937 | 0.000352 | 0.01575 | 1 | 65 | 80 | 67 |
| Potri.003 | 0.717787 | 2.553515 | 0.000549 | 0.022035 | 1 | 70 | 43 | 57 |
| Potri.003 | 0.676997 | 4.285084 | 3.69E-06 | 0.000426 | 1 | 188 | 212 | 181 |
| Potri.003 | 1.682148 | 2.579024 | 2.09E-11 | 1.20E-08 | 1 | 38 | 34 | 37 |
| Potri.003 | 0.788922 | 7.346334 | 5.46E-05 | 0.003794 | 1 | 1152 | 1962 | 1491 |
| Potri.003 | 1.116424 | 5.097493 | 1.04E-12 | 8.44E-10 | 1 | 248 | 287 | 303 |
| Potri.003 | 0.699018 | 6.800049 | 5.08E-06 | 0.000555 | 1 | 1015 | 1254 | 1030 |
| Potri.003 | 0.711347 | 4.369904 | 4.09E-05 | 0.003012 | 1 | 251 | 167 | 194 |
| Potri.003 | -0.63878 | 3.297433 | 0.001102 | 0.03764 | -1 | 163 | 169 | 127 |
| Potri.003 | -0.63823 | 4.632977 | 9.31E-05 | 0.005702 | -1 | 398 | 400 | 372 |
| Potri.003 | 0.552013 | 6.367706 | 1.50E-06 | 0.000201 | 1 | 877 | 910 | 821 |
| Potri.003 | -0.7836 | 3.562829 | 6.47E-05 | 0.004373 | -1 | 191 | 194 | 189 |
| Potri.003 | 0.505143 | 4.961525 | 0.000116 | 0.006644 | 1 | 339 | 343 | 319 |
| Potri.003 | 0.813329 | 6.112858 | 8.90E-08 | 1.92E-05 | 1 | 783 | 542 | 643 |
| Potri.003 | 0.602275 | 5.094114 | 1.19E-05 | 0.001104 | 1 | 374 | 361 | 321 |
| Potri.003 | -0.72407 | 4.783236 | 2.24E-08 | 6.13E-06 | -1 | 451 | 459 | 419 |
| Potri.003 | -0.40514 | 5.436004 | 0.000397 | 0.017311 | -1 | 709 | 583 | 631 |
| Potri.003 | 1.065518 | 1.87097 | 3.78E-06 | 0.000433 | 1 | 32 | 25 | 32 |
| Potri.003 | 0.424437 | 5.331467 | 0.001283 | 0.041608 | 1 | 506 | 426 | 410 |
| Potri.003 | -1.98732 | -0.4646 | 0.000332 | 0.015167 | -1 | 14 | 11 | 10 |
| Potri.003 | 0.42403 | 8.086047 | 0.001489 | 0.04619 | 1 | 2984 | 3011 | 3049 |
| Potri.003 | 0.760154 | 9.0103 | 2.54E-09 | 9.65E-07 | 1 | 4383 | 5110 | 5379 |
| Potri.003 | -0.61805 | 4.076674 | 0.000222 | 0.011131 | -1 | 346 | 224 | 225 |
| Potri.003 | 0.441681 | 6.066027 | 0.000314 | 0.014582 | 1 | 683 | 764 | 760 |
| Potri.003 | -0.46742 | 5.655082 | 0.000195 | 0.009992 | -1 | 821 | 657 | 802 |
| Potri.003 | 5.488767 | -1.43455 | 0.000135 | 0.007472 | 1 | 0 | 0 | 0 |
| Potri.003 | 0.551493 | 3.322974 | 0.000661 | 0.025659 | 1 | 111 | 96 | 106 |
| Potri.003 | -0.65944 | 2.968201 | 8.70E-05 | 0.005401 | -1 | 129 | 112 | 125 |
| Potri.003 | -0.50984 | 5.842655 | 0.000114 | 0.006569 | -1 | 811 | 872 | 933 |
| Potri.003 | 0.624865 | 4.902444 | 0.000191 | 0.009847 | 1 | 310 | 338 | 266 |
| Potri.003 | 0.482309 | 5.807377 | 0.000102 | 0.006109 | 1 | 739 | 516 | 576 |
| Potri.003 | 0.727536 | 4.582709 | 0.000147 | 0.008053 | 1 | 324 | 156 | 229 |
| Potri.003 | -0.43239 | 5.061458 | 0.000583 | 0.023147 | -1 | 502 | 482 | 505 |
| Potri.003 | -2.85443 | -0.01313 | 0.000139 | 0.0077 | -1 | 7 | 30 | 19 |
| Potri.003 | 0.585213 | 5.299015 | 8.41E-05 | 0.005283 | 1 | 383 | 487 | 351 |
| Potri.003 | -0.52846 | 3.591735 | 0.001144 | 0.038692 | -1 | 190 | 178 | 180 |
| Potri.003 | 0.566342 | 5.053538 | 0.000156 | 0.008383 | 1 | 343 | 307 | 391 |
| Potri.003 | -0.54563 | 3.727711 | 0.001295 | 0.04196 | -1 | 202 | 217 | 186 |
| Potri.003 | 0.427972 | 4.376439 | 0.001603 | 0.048792 | 1 | 233 | 223 | 231 |
| Potri.003 | -0.74221 | 2.484828 | 0.000474 | 0.019913 | -1 | 95 | 97 | 73 |
| Potri.003 | -1.45942 | 0.321644 | 0.000784 | 0.029428 | -1 | 25 | 21 | 16 |
| Potri.003 | 0.648803 | 7.585939 | 1.39E-07 | 2.86E-05 | 1 | 1824 | 2157 | 1836 |
| Potri.004 | 0.647534 | 6.195357 | 2.68E-06 | 0.000327 | 1 | 750 | 852 | 620 |
| Potri.004 | -1.14755 | 3.958255 | 2.80E-06 | 0.00034 | -1 | 409 | 236 | 190 |
| Potri.004 | 0.804029 | 5.124847 | 5.24E-10 | 2.25E-07 | 1 | 364 | 318 | 309 |

| | | | | | | | | |
|---|---|---|---|---|---|---|---|---|
| Potri.004 | 0.587352 | 9.066446 | 2.38E-07 | 4.43E-05 | 1 | 5890 | 5176 | 5678 |
| Potri.004 | -0.57524 | 6.201165 | 1.26E-06 | 0.000176 | -1 | 1257 | 966 | 1216 |
| Potri.004 | -1.04601 | 4.13805 | 3.65E-06 | 0.000422 | -1 | 443 | 190 | 294 |
| Potri.004 | -0.66726 | 2.997528 | 0.000919 | 0.033221 | -1 | 140 | 109 | 126 |
| Potri.004 | 1.555424 | 1.517432 | 1.55E-07 | 3.14E-05 | 1 | 19 | 19 | 16 |
| Potri.004 | -0.8293 | 5.811825 | 6.80E-06 | 0.000697 | -1 | 750 | 1092 | 933 |
| Potri.004 | -1.87603 | 1.029771 | 5.53E-07 | 9.00E-05 | -1 | 33 | 51 | 30 |
| Potri.004 | -0.45979 | 8.518899 | 2.33E-05 | 0.001938 | -1 | 5792 | 5156 | 5642 |
| Potri.004 | 1.358355 | 2.936321 | 4.91E-11 | 2.67E-08 | 1 | 68 | 40 | 58 |
| Potri.004 | 0.982763 | 3.650102 | 3.75E-07 | 6.54E-05 | 1 | 119 | 103 | 105 |
| Potri.004 | -1.80445 | -0.11552 | 0.000541 | 0.021822 | -1 | 17 | 14 | 15 |
| Potri.004 | 0.689231 | 6.698208 | 8.14E-07 | 0.000123 | 1 | 897 | 1057 | 1131 |
| Potri.004 | 0.421877 | 6.73898 | 0.000302 | 0.01411 | 1 | 1230 | 1121 | 1211 |
| Potri.004 | -1.54461 | 3.528254 | 3.29E-12 | 2.21E-09 | -1 | 194 | 239 | 223 |
| Potri.004 | -0.69537 | 2.62804 | 0.000309 | 0.01439 | -1 | 118 | 85 | 88 |
| Potri.004 | -1.02315 | 3.408474 | 1.35E-08 | 3.96E-06 | -1 | 218 | 142 | 188 |
| Potri.004 | -0.67172 | 3.285445 | 0.000245 | 0.011977 | -1 | 152 | 135 | 172 |
| Potri.004 | 1.068642 | 3.34319 | 3.50E-07 | 6.21E-05 | 1 | 74 | 80 | 98 |
| Potri.004 | -0.6862 | 2.399933 | 0.001537 | 0.047254 | -1 | 72 | 95 | 78 |
| Potri.004 | 1.885981 | 4.768346 | 1.54E-28 | 1.65E-24 | 1 | 142 | 142 | 167 |
| Potri.004 | -1.08339 | 1.585227 | 6.70E-05 | 0.004491 | -1 | 56 | 52 | 42 |
| Potri.004 | -1.94835 | 1.735985 | 2.73E-05 | 0.002187 | -1 | 34 | 107 | 52 |
| Potri.004 | -1.11312 | 2.045407 | 2.49E-05 | 0.002039 | -1 | 98 | 62 | 52 |
| Potri.004 | 0.591571 | 5.04312 | 8.71E-06 | 0.000862 | 1 | 394 | 303 | 330 |
| Potri.004 | 1.233569 | 1.397149 | 1.83E-05 | 0.001581 | 1 | 19 | 20 | 19 |
| Potri.004 | -0.42303 | 4.986564 | 0.000685 | 0.026391 | -1 | 543 | 439 | 433 |
| Potri.004 | 0.504944 | 8.736081 | 8.86E-07 | 0.000132 | 1 | 4843 | 4387 | 4542 |
| Potri.004 | 0.99788 | 4.709529 | 5.35E-05 | 0.003722 | 1 | 296 | 172 | 216 |
| Potri.004 | -0.77593 | 2.345836 | 0.000429 | 0.018455 | -1 | 103 | 67 | 73 |
| Potri.004 | -0.36646 | 7.261809 | 0.001098 | 0.037536 | -1 | 2193 | 2285 | 2250 |
| Potri.004 | -1.24339 | 4.841806 | 1.60E-08 | 4.57E-06 | -1 | 421 | 628 | 502 |
| Potri.004 | 0.372054 | 7.140819 | 0.000456 | 0.019428 | 1 | 1650 | 1566 | 1583 |
| Potri.004 | 0.542924 | 4.659701 | 2.81E-05 | 0.002237 | 1 | 283 | 258 | 259 |
| Potri.004 | -0.63226 | 4.003632 | 1.93E-05 | 0.001652 | -1 | 252 | 252 | 248 |
| Potri.004 | -0.96946 | 3.339124 | 9.85E-06 | 0.000946 | -1 | 127 | 170 | 213 |
| Potri.004 | -0.39941 | 6.407688 | 0.000469 | 0.019826 | -1 | 1353 | 1121 | 1296 |
| Potri.004 | -2.07942 | 3.413293 | 2.82E-22 | 1.52E-18 | -1 | 213 | 241 | 203 |
| Potri.005 | 0.739379 | 5.528423 | 4.85E-10 | 2.10E-07 | 1 | 454 | 450 | 442 |
| Potri.005 | 0.488963 | 5.057097 | 0.000497 | 0.020517 | 1 | 400 | 339 | 341 |
| Potri.005 | -2.64211 | -1.03449 | 0.000835 | 0.0309 | -1 | 7 | 10 | 5 |
| Potri.005 | -0.60486 | 2.679938 | 0.001491 | 0.046207 | -1 | 103 | 83 | 108 |
| Potri.005 | -2.58858 | -0.05375 | 6.03E-06 | 0.000635 | -1 | 11 | 24 | 18 |
| Potri.005 | 0.494577 | 7.126896 | 6.61E-06 | 0.000684 | 1 | 1632 | 1479 | 1422 |
| Potri.005 | 0.8859 | 2.579631 | 6.39E-06 | 0.000668 | 1 | 47 | 54 | 59 |
| Potri.005 | -0.53521 | 6.367033 | 3.70E-06 | 0.000426 | -1 | 1241 | 1299 | 1258 |
| Potri.005 | -1.55181 | 2.523132 | 4.62E-08 | 1.10E-05 | -1 | 92 | 122 | 108 |
| Potri.005 | -0.86009 | 4.118291 | 0.000547 | 0.022008 | -1 | 224 | 365 | 269 |
| Potri.005 | -1.37263 | 0.384274 | 0.000688 | 0.026483 | -1 | 20 | 26 | 18 |
| Potri.005 | -0.44271 | 4.27048 | 0.001195 | 0.039872 | -1 | 312 | 250 | 300 |
| Potri.005 | -0.39338 | 5.782048 | 0.001197 | 0.039872 | -1 | 952 | 748 | 742 |
| Potri.005 | 0.95249 | 2.781292 | 2.76E-07 | 4.99E-05 | 1 | 69 | 47 | 64 |
| Potri.005 | -0.42442 | 7.614746 | 0.000176 | 0.009226 | -1 | 3248 | 2702 | 2836 |
| Potri.005 | -0.7328 | 3.362914 | 0.001002 | 0.035217 | -1 | 178 | 123 | 193 |
| Potri.005 | -0.49454 | 7.551986 | 4.72E-06 | 0.00052 | -1 | 2884 | 2786 | 2887 |

| | | | | | | | | |
|---|---|---|---|---|---|---|---|---|
| Potri.005 | -1.0059 | 1.18914 | 0.00094 | 0.033687 | -1 | 37 | 29 | 44 |
| Potri.005 | -1.45525 | 3.45393 | 3.80E-08 | 9.42E-06 | -1 | 170 | 234 | 208 |
| Potri.005 | -0.55581 | 4.206249 | 0.000386 | 0.017029 | -1 | 308 | 260 | 283 |
| Potri.005 | 0.794099 | 5.16942 | 3.57E-09 | 1.27E-06 | 1 | 394 | 325 | 309 |
| Potri.005 | 0.901551 | 5.212703 | 2.27E-08 | 6.16E-06 | 1 | 376 | 373 | 259 |
| Potri.005 | -1.60457 | 2.129072 | 2.78E-08 | 7.36E-06 | -1 | 108 | 71 | 68 |
| Potri.005 | 0.361505 | 7.251505 | 0.001236 | 0.040569 | 1 | 1926 | 1649 | 1641 |
| Potri.005 | -1.30999 | 1.384681 | 4.56E-05 | 0.003312 | -1 | 37 | 56 | 42 |
| Potri.005 | -0.46274 | 6.261908 | 0.001156 | 0.039048 | -1 | 1124 | 1067 | 1269 |
| Potri.005 | 0.539118 | 5.122583 | 3.11E-05 | 0.00244 | 1 | 446 | 308 | 357 |
| Potri.005 | 0.606765 | 5.42366 | 5.81E-07 | 9.24E-05 | 1 | 476 | 403 | 448 |
| Potri.005 | 0.562517 | 4.934894 | 4.78E-05 | 0.003438 | 1 | 306 | 349 | 303 |
| Potri.005 | -0.94076 | 1.870048 | 0.000408 | 0.017723 | -1 | 47 | 63 | 68 |
| Potri.005 | -0.81174 | 2.05911 | 0.000321 | 0.014773 | -1 | 76 | 61 | 62 |
| Potri.005 | 0.538155 | 4.233637 | 0.000838 | 0.03095 | 1 | 178 | 201 | 214 |
| Potri.005 | -0.48778 | 4.206288 | 0.001188 | 0.039784 | -1 | 311 | 224 | 301 |
| Potri.005 | -0.72692 | 3.806899 | 0.000199 | 0.010165 | -1 | 214 | 227 | 230 |
| Potri.005 | 0.415951 | 6.205676 | 0.000485 | 0.02021 | 1 | 780 | 819 | 860 |
| Potri.005 | -0.64177 | 5.302218 | 5.68E-05 | 0.003931 | -1 | 771 | 471 | 640 |
| Potri.005 | 0.411214 | 6.376942 | 0.00026 | 0.012614 | 1 | 1077 | 870 | 845 |
| Potri.005 | 0.585568 | 8.338319 | 5.71E-09 | 1.89E-06 | 1 | 3585 | 3192 | 3338 |
| Potri.005 | -0.75999 | 3.214344 | 4.01E-05 | 0.002969 | -1 | 158 | 141 | 148 |
| Potri.005 | -0.58965 | 7.501352 | 0.001395 | 0.044345 | -1 | 4118 | 1912 | 2587 |
| Potri.005 | 0.392352 | 7.730171 | 0.000236 | 0.011654 | 1 | 2525 | 2312 | 2334 |
| Potri.005 | -1.55039 | 1.260528 | 4.03E-07 | 6.90E-05 | -1 | 44 | 48 | 37 |
| Potri.006 | -0.51823 | 4.102917 | 0.001444 | 0.045275 | -1 | 326 | 197 | 264 |
| Potri.006 | -1.12007 | 3.374216 | 3.89E-07 | 6.71E-05 | -1 | 233 | 117 | 198 |
| Potri.006 | 0.48723 | 4.303926 | 0.001404 | 0.044478 | 1 | 228 | 187 | 224 |
| Potri.006 | -1.33382 | 0.308012 | 0.001074 | 0.037077 | -1 | 26 | 20 | 14 |
| Potri.006 | 0.939677 | 3.274996 | 1.31E-06 | 0.000181 | 1 | 77 | 103 | 75 |
| Potri.006 | -1.22662 | 2.908646 | 5.01E-08 | 1.17E-05 | -1 | 160 | 94 | 148 |
| Potri.006 | -0.53989 | 3.958022 | 0.001327 | 0.042746 | -1 | 267 | 249 | 196 |
| Potri.006 | 0.409493 | 8.166672 | 0.000486 | 0.020243 | 1 | 3199 | 2888 | 3542 |
| Potri.006 | -0.78506 | 5.045792 | 4.71E-06 | 0.00052 | -1 | 711 | 418 | 508 |
| Potri.006 | 1.083573 | 7.480539 | 6.95E-13 | 5.86E-10 | 1 | 1782 | 1226 | 1487 |
| Potri.006 | -0.60873 | 3.688349 | 0.000459 | 0.019503 | -1 | 189 | 215 | 194 |
| Potri.006 | -0.8525 | 2.942446 | 1.93E-05 | 0.001652 | -1 | 111 | 127 | 138 |
| Potri.006 | 0.380875 | 6.956897 | 0.001003 | 0.035217 | 1 | 1423 | 1256 | 1531 |
| Potri.006 | 1.007919 | 1.670261 | 0.000233 | 0.011539 | 1 | 27 | 22 | 30 |
| Potri.006 | 0.398711 | 8.035484 | 0.001092 | 0.037453 | 1 | 3401 | 2368 | 3108 |
| Potri.006 | -0.40875 | 6.091339 | 0.000277 | 0.013169 | -1 | 1099 | 977 | 958 |
| Potri.006 | -2.06377 | 3.719631 | 2.63E-07 | 4.79E-05 | -1 | 167 | 400 | 239 |
| Potri.006 | -0.80242 | 2.78556 | 1.26E-05 | 0.00116 | -1 | 125 | 93 | 116 |
| Potri.006 | -0.50468 | 4.733937 | 0.000134 | 0.007472 | -1 | 430 | 370 | 412 |
| Potri.006 | -1.12559 | 4.1793 | 2.02E-06 | 0.000256 | -1 | 235 | 379 | 337 |
| Potri.006 | 1.605001 | 1.794669 | 1.02E-06 | 0.000149 | 1 | 15 | 33 | 16 |
| Potri.006 | 1.208376 | 6.277807 | 3.97E-17 | 7.54E-14 | 1 | 694 | 473 | 669 |
| Potri.006 | 0.497026 | 5.916648 | 0.000128 | 0.007229 | 1 | 604 | 717 | 624 |
| Potri.006 | -4.00124 | -1.07632 | 3.10E-05 | 0.002436 | -1 | 12 | 6 | 5 |
| Potri.006 | 0.497241 | 5.719551 | 2.85E-05 | 0.002266 | 1 | 587 | 575 | 540 |
| Potri.006 | 0.71698 | 3.098252 | 6.83E-05 | 0.004551 | 1 | 82 | 78 | 89 |
| Potri.006 | 0.500677 | 5.221102 | 2.58E-05 | 0.002087 | 1 | 432 | 365 | 407 |
| Potri.006 | 0.350799 | 8.837468 | 0.000775 | 0.029208 | 1 | 5253 | 5191 | 5236 |
| Potri.006 | 1.904007 | -0.38259 | 0.000178 | 0.00931 | 1 | 5 | 3 | 2 |

| | | | | | | | | |
|---|---|---|---|---|---|---|---|---|
| Potri.006 | 0.377252 | 7.827916 | 0.000469 | 0.019828 | 1 | 2925 | 2270 | 2548 |
| Potri.006 | -0.87064 | 3.504264 | 2.47E-06 | 0.000306 | -1 | 204 | 176 | 184 |
| Potri.006 | -0.39348 | 7.403529 | 0.000294 | 0.013817 | -1 | 2488 | 2384 | 2620 |
| Potri.006 | -0.69881 | 2.541187 | 0.00162 | 0.049164 | -1 | 99 | 93 | 81 |
| Potri.006 | -0.70617 | 5.461544 | 7.40E-05 | 0.004815 | -1 | 727 | 637 | 761 |
| Potri.006 | 0.668007 | 6.379124 | 1.63E-05 | 0.001437 | 1 | 857 | 983 | 663 |
| Potri.006 | 0.641397 | 5.397664 | 2.47E-06 | 0.000306 | 1 | 391 | 444 | 443 |
| Potri.006 | 0.911964 | 2.512193 | 0.000142 | 0.00784 | 1 | 51 | 37 | 63 |
| Potri.006 | -1.11863 | 1.472404 | 0.001506 | 0.04657 | -1 | 26 | 57 | 55 |
| Potri.006 | -0.38945 | 8.085736 | 0.000235 | 0.011615 | -1 | 4050 | 4065 | 3896 |
| Potri.006 | 0.437533 | 6.090905 | 0.000729 | 0.027736 | 1 | 925 | 638 | 708 |
| Potri.006 | 0.680397 | 2.872806 | 0.001522 | 0.046894 | 1 | 64 | 91 | 60 |
| Potri.006 | -0.56201 | 3.801044 | 0.000274 | 0.013072 | -1 | 256 | 206 | 181 |
| Potri.006 | -0.43383 | 5.592608 | 0.000152 | 0.008207 | -1 | 756 | 715 | 687 |
| Potri.006 | -0.67645 | 6.036515 | 9.22E-09 | 2.77E-06 | -1 | 1165 | 1040 | 945 |
| Potri.006 | -3.67759 | 0.815196 | 1.14E-13 | 1.11E-10 | -1 | 44 | 47 | 23 |
| Potri.006 | -3.87797 | 3.737086 | 7.49E-37 | 3.22E-32 | -1 | 489 | 244 | 234 |
| Potri.006 | 0.680814 | 7.571514 | 8.79E-09 | 2.70E-06 | 1 | 1955 | 1659 | 2093 |
| Potri.006 | -0.92868 | 2.748867 | 2.22E-05 | 0.001871 | -1 | 103 | 113 | 118 |
| Potri.006 | -2.33619 | 1.504841 | 2.51E-05 | 0.002045 | -1 | 67 | 68 | 38 |
| Potri.006 | 0.759921 | 4.926295 | 1.55E-06 | 0.000206 | 1 | 259 | 342 | 273 |
| Potri.007 | -0.4826 | 6.227142 | 0.000159 | 0.008484 | -1 | 1033 | 1145 | 1211 |
| Potri.007 | -1.79363 | 3.332394 | 1.58E-14 | 1.79E-11 | -1 | 192 | 220 | 184 |
| Potri.007 | -0.47456 | 4.889154 | 0.000606 | 0.023849 | -1 | 486 | 451 | 402 |
| Potri.007 | 0.547159 | 7.837176 | 2.36E-07 | 4.41E-05 | 1 | 2701 | 2127 | 2447 |
| Potri.007 | -1.37689 | 1.109266 | 9.03E-06 | 0.000888 | -1 | 45 | 31 | 36 |
| Potri.007 | -0.66512 | 4.439421 | 0.00057 | 0.022728 | -1 | 351 | 386 | 292 |
| Potri.007 | -0.4995 | 8.251177 | 6.98E-05 | 0.004642 | -1 | 4527 | 4769 | 4601 |
| Potri.007 | -0.9743 | 4.291315 | 3.99E-05 | 0.002968 | -1 | 270 | 405 | 321 |
| Potri.007 | 0.667056 | 2.626518 | 0.000905 | 0.032817 | 1 | 75 | 48 | 60 |
| Potri.007 | -0.5271 | 4.118495 | 0.000104 | 0.006177 | -1 | 280 | 262 | 251 |
| Potri.007 | -0.97186 | 6.028766 | 1.34E-05 | 0.001208 | -1 | 845 | 1342 | 1147 |
| Potri.007 | -2.20053 | 0.575166 | 4.87E-07 | 8.02E-05 | -1 | 18 | 32 | 34 |
| Potri.007 | 0.825414 | 2.160488 | 0.000365 | 0.01624 | 1 | 38 | 35 | 49 |
| Potri.007 | -0.52221 | 4.910651 | 3.29E-05 | 0.002546 | -1 | 474 | 418 | 485 |
| Potri.007 | -0.56334 | 5.786903 | 0.000153 | 0.008254 | -1 | 801 | 1009 | 742 |
| Potri.007 | -1.42187 | 3.865865 | 8.54E-11 | 4.46E-08 | -1 | 239 | 322 | 252 |
| Potri.007 | -0.50417 | 4.266085 | 0.001179 | 0.039588 | -1 | 327 | 277 | 271 |
| Potri.007 | -1.02564 | 2.880539 | 1.45E-08 | 4.20E-06 | -1 | 132 | 120 | 124 |
| Potri.007 | -1.21678 | 3.316892 | 1.04E-08 | 3.12E-06 | -1 | 158 | 210 | 163 |
| Potri.007 | -0.53861 | 9.579096 | 9.64E-07 | 0.000142 | -1 | 11803 | 11890 | 11630 |
| Potri.007 | -0.90591 | 2.168169 | 0.0009 | 0.032764 | -1 | 83 | 80 | 57 |
| Potri.007 | -0.40551 | 6.00418 | 0.000825 | 0.030602 | -1 | 944 | 1025 | 874 |
| Potri.007 | 2.563311 | -0.51199 | 8.78E-05 | 0.005435 | 1 | 2 | 2 | 2 |
| Potri.007 | 0.376592 | 6.647199 | 0.000795 | 0.029742 | 1 | 1318 | 1040 | 1057 |
| Potri.007 | -1.17343 | 4.510188 | 1.79E-08 | 5.05E-06 | -1 | 326 | 468 | 419 |
| Potri.007 | -0.59394 | 3.006476 | 0.000501 | 0.020621 | -1 | 125 | 127 | 117 |
| Potri.007 | -0.43665 | 5.151958 | 0.000274 | 0.013082 | -1 | 576 | 511 | 504 |
| Potri.007 | -0.63465 | 3.967555 | 6.46E-06 | 0.000673 | -1 | 248 | 250 | 236 |
| Potri.008 | -0.6071 | 6.705963 | 3.01E-08 | 7.84E-06 | -1 | 1827 | 1606 | 1491 |
| Potri.008 | -0.44044 | 7.807973 | 7.32E-05 | 0.004783 | -1 | 3740 | 2945 | 3414 |
| Potri.008 | 1.321274 | 1.819899 | 5.29E-05 | 0.003691 | 1 | 32 | 14 | 30 |
| Potri.008 | -1.67363 | 2.282093 | 1.42E-11 | 8.22E-09 | -1 | 86 | 92 | 99 |
| Potri.008 | 0.353074 | 7.892937 | 0.001375 | 0.043948 | 1 | 2667 | 2820 | 2643 |

| | | | | | | | | |
|---|---|---|---|---|---|---|---|---|
| Potri.008 | -0.57658 | 3.140712 | 0.001028 | 0.035839 | -1 | 140 | 141 | 123 |
| Potri.008 | -1.16406 | 3.736152 | 1.53E-07 | 3.11E-05 | -1 | 191 | 288 | 225 |
| Potri.008 | 0.330655 | 8.480291 | 0.001294 | 0.041958 | 1 | 4608 | 3792 | 3985 |
| Potri.008 | 0.610595 | 4.406739 | 6.16E-06 | 0.000647 | 1 | 218 | 229 | 204 |
| Potri.008 | -0.78517 | 2.915856 | 7.13E-05 | 0.004712 | -1 | 141 | 106 | 118 |
| Potri.008 | 0.468141 | 6.239946 | 0.000119 | 0.006804 | 1 | 845 | 840 | 786 |
| Potri.008 | -0.65809 | 8.914153 | 3.48E-05 | 0.002637 | -1 | 8203 | 7057 | 7824 |
| Potri.008 | 0.585206 | 7.249599 | 2.46E-07 | 4.56E-05 | 1 | 1680 | 1632 | 1439 |
| Potri.008 | -0.4338 | 7.062808 | 0.000226 | 0.011225 | -1 | 1946 | 2155 | 1875 |
| Potri.008 | -0.5279 | 6.416613 | 2.08E-05 | 0.001765 | -1 | 1319 | 1430 | 1175 |
| Potri.008 | -0.63917 | 6.461415 | 8.04E-08 | 1.76E-05 | -1 | 1310 | 1386 | 1474 |
| Potri.008 | -0.53798 | 6.877833 | 2.00E-06 | 0.000255 | -1 | 1796 | 1744 | 1884 |
| Potri.008 | 0.707553 | 4.755209 | 6.88E-06 | 0.000704 | 1 | 283 | 208 | 308 |
| Potri.008 | -1.73518 | 0.398686 | 0.000229 | 0.011386 | -1 | 25 | 26 | 18 |
| Potri.008 | -0.63263 | 5.733476 | 1.80E-07 | 3.50E-05 | -1 | 868 | 845 | 803 |
| Potri.008 | 0.856411 | 4.515398 | 9.19E-09 | 2.77E-06 | 1 | 197 | 206 | 228 |
| Potri.008 | -0.65897 | 2.992311 | 0.000424 | 0.018302 | -1 | 115 | 139 | 117 |
| Potri.008 | -3.10096 | 1.456521 | 5.25E-12 | 3.27E-09 | -1 | 87 | 64 | 28 |
| Potri.008 | 0.53808 | 3.95444 | 0.000337 | 0.015298 | 1 | 183 | 130 | 178 |
| Potri.008 | -1.42306 | 2.767864 | 0.000112 | 0.006512 | -1 | 70 | 180 | 122 |
| Potri.008 | 0.830599 | 3.05757 | 2.02E-06 | 0.000256 | 1 | 90 | 73 | 68 |
| Potri.008 | 0.776973 | 10.20572 | 4.96E-08 | 1.16E-05 | 1 | 13053 | 11655 | 9380 |
| Potri.008 | 0.521554 | 5.574727 | 0.000157 | 0.008405 | 1 | 573 | 405 | 552 |
| Potri.008 | 0.466466 | 5.250619 | 0.00041 | 0.017803 | 1 | 387 | 453 | 400 |
| Potri.008 | -0.44543 | 6.491986 | 0.000104 | 0.006159 | -1 | 1521 | 1256 | 1280 |
| Potri.008 | 0.586247 | 6.374797 | 0.000472 | 0.019883 | 1 | 867 | 683 | 1039 |
| Potri.008 | -0.51994 | 4.86078 | 0.001204 | 0.039955 | -1 | 556 | 435 | 345 |
| Potri.008 | -1.29373 | 0.757237 | 0.000915 | 0.033175 | -1 | 20 | 36 | 28 |
| Potri.008 | 0.563573 | 6.869962 | 2.12E-05 | 0.001794 | 1 | 1141 | 1351 | 1176 |
| Potri.008 | -0.9154 | 4.667412 | 7.54E-10 | 3.11E-07 | -1 | 427 | 435 | 423 |
| Potri.008 | -0.64399 | 4.477002 | 0.000238 | 0.011732 | -1 | 434 | 241 | 384 |
| Potri.008 | -0.92918 | 1.548146 | 0.001262 | 0.041153 | -1 | 48 | 35 | 58 |
| Potri.008 | 0.710965 | 5.00379 | 3.15E-08 | 8.00E-06 | 1 | 307 | 330 | 308 |
| Potri.008 | -0.63895 | 3.954555 | 4.67E-05 | 0.003371 | -1 | 247 | 253 | 228 |
| Potri.008 | 0.576597 | 3.757658 | 0.000325 | 0.014903 | 1 | 162 | 138 | 121 |
| Potri.008 | -0.48234 | 4.425752 | 0.00032 | 0.014757 | -1 | 371 | 295 | 307 |
| Potri.008 | 0.796616 | 6.741656 | 6.88E-08 | 1.55E-05 | 1 | 1010 | 1120 | 914 |
| Potri.008 | -0.69611 | 5.981349 | 4.88E-06 | 0.000534 | -1 | 1285 | 779 | 1002 |
| Potri.008 | 0.576804 | 3.881871 | 0.000605 | 0.023827 | 1 | 162 | 162 | 134 |
| Potri.008 | 0.590781 | 2.930927 | 0.000864 | 0.031761 | 1 | 72 | 80 | 81 |
| Potri.009 | 0.496155 | 5.508836 | 8.83E-05 | 0.00545 | 1 | 465 | 523 | 479 |
| Potri.009 | -0.59484 | 6.413811 | 4.77E-07 | 7.89E-05 | -1 | 1451 | 1258 | 1295 |
| Potri.009 | -0.74952 | 2.184992 | 0.000923 | 0.033266 | -1 | 72 | 74 | 68 |
| Potri.009 | -0.49926 | 4.815857 | 6.18E-05 | 0.004211 | -1 | 426 | 410 | 443 |
| Potri.009 | -0.56398 | 7.197263 | 1.15E-06 | 0.000165 | -1 | 2148 | 2334 | 2325 |
| Potri.009 | 0.709677 | 4.219195 | 9.52E-05 | 0.005802 | 1 | 177 | 149 | 222 |
| Potri.009 | -2.23261 | 2.770109 | 2.49E-15 | 3.35E-12 | -1 | 131 | 169 | 124 |
| Potri.009 | 0.578482 | 3.893613 | 6.69E-05 | 0.004491 | 1 | 179 | 132 | 152 |
| Potri.009 | -0.39685 | 5.447107 | 0.000997 | 0.035168 | -1 | 722 | 573 | 639 |
| Potri.009 | -1.114 | 6.062991 | 6.59E-08 | 1.51E-05 | -1 | 949 | 1380 | 1202 |
| Potri.009 | 0.632914 | 5.404899 | 2.19E-06 | 0.000277 | 1 | 482 | 390 | 425 |
| Potri.009 | -2.21168 | 0.353832 | 2.65E-06 | 0.000326 | -1 | 23 | 17 | 31 |
| Potri.009 | -3.61904 | 1.269807 | 4.07E-13 | 3.72E-10 | -1 | 28 | 72 | 59 |
| Potri.009 | -1.07793 | 2.978093 | 1.72E-05 | 0.001501 | -1 | 108 | 170 | 127 |

| | | | | | | | | |
|---|---|---|---|---|---|---|---|---|
| Potri.009 | -0.37531 | 6.400678 | 0.001517 | 0.046821 | -1 | 1481 | 1114 | 1142 |
| Potri.009 | -0.99866 | 4.873567 | 4.33E-09 | 1.47E-06 | -1 | 473 | 499 | 539 |
| Potri.009 | -0.60024 | 6.418584 | 9.11E-06 | 0.000892 | -1 | 1352 | 1448 | 1210 |
| Potri.009 | 0.540768 | 6.421683 | 2.46E-06 | 0.000306 | 1 | 998 | 811 | 921 |
| Potri.009 | 1.303156 | 4.166692 | 1.88E-16 | 3.23E-13 | 1 | 135 | 130 | 137 |
| Potri.009 | 0.885876 | 3.92364 | 6.04E-05 | 0.004138 | 1 | 108 | 162 | 140 |
| Potri.009 | -0.56834 | 5.525684 | 4.11E-05 | 0.003026 | -1 | 683 | 761 | 690 |
| Potri.009 | 0.512772 | 5.209402 | 0.000272 | 0.013032 | 1 | 404 | 446 | 335 |
| Potri.009 | -0.66464 | 3.301246 | 0.000124 | 0.007071 | -1 | 155 | 161 | 147 |
| Potri.009 | 1.589317 | 4.583169 | 1.62E-13 | 1.54E-10 | 1 | 183 | 170 | 114 |
| Potri.009 | 0.335679 | 6.649222 | 0.001393 | 0.044325 | 1 | 1223 | 1094 | 1147 |
| Potri.009 | -0.70447 | 3.262482 | 0.000196 | 0.010059 | -1 | 192 | 133 | 133 |
| Potri.009 | 0.428868 | 7.699385 | 9.61E-05 | 0.005819 | 1 | 2388 | 2304 | 2221 |
| Potri.009 | 0.731245 | 6.099768 | 2.23E-09 | 8.55E-07 | 1 | 770 | 580 | 668 |
| Potri.009 | -0.62437 | 6.029115 | 1.55E-08 | 4.47E-06 | -1 | 1078 | 1024 | 983 |
| Potri.009 | -0.7956 | 2.217801 | 0.000284 | 0.013415 | -1 | 80 | 70 | 72 |
| Potri.009 | 0.426847 | 7.105002 | 0.000452 | 0.019326 | 1 | 1729 | 1340 | 1529 |
| Potri.009 | -2.47474 | 3.388679 | 3.68E-17 | 7.53E-14 | -1 | 195 | 295 | 184 |
| Potri.009 | -0.7391 | 2.855223 | 0.000525 | 0.021343 | -1 | 96 | 135 | 112 |
| Potri.009 | 0.762583 | 3.477223 | 2.36E-06 | 0.000295 | 1 | 112 | 107 | 100 |
| Potri.009 | 0.532368 | 4.381339 | 7.94E-05 | 0.005072 | 1 | 236 | 203 | 223 |
| Potri.009 | -0.74618 | 3.219596 | 0.001096 | 0.037523 | -1 | 193 | 110 | 147 |
| Potri.010 | -0.49043 | 5.414903 | 5.30E-05 | 0.003693 | -1 | 763 | 565 | 619 |
| Potri.010 | 0.444224 | 5.628315 | 0.000135 | 0.007472 | 1 | 613 | 537 | 486 |
| Potri.010 | 0.368176 | 6.211808 | 0.001392 | 0.044325 | 1 | 812 | 856 | 849 |
| Potri.010 | -0.77616 | 2.012043 | 0.001211 | 0.040141 | -1 | 81 | 59 | 51 |
| Potri.010 | 1.020813 | 1.209087 | 0.0012 | 0.039925 | 1 | 24 | 12 | 20 |
| Potri.010 | -0.59619 | 4.666745 | 8.67E-06 | 0.000862 | -1 | 434 | 380 | 373 |
| Potri.010 | 0.771331 | 3.946148 | 4.57E-07 | 7.58E-05 | 1 | 162 | 135 | 145 |
| Potri.010 | -3.05849 | 1.666163 | 1.53E-20 | 6.55E-17 | -1 | 69 | 81 | 57 |
| Potri.010 | -0.52803 | 6.416446 | 3.42E-05 | 0.002606 | -1 | 1402 | 1371 | 1159 |
| Potri.010 | 0.984428 | 2.733993 | 5.31E-06 | 0.000574 | 1 | 76 | 44 | 52 |
| Potri.010 | 0.816288 | 2.443342 | 0.001096 | 0.037523 | 1 | 65 | 28 | 58 |
| Potri.010 | -0.66743 | 2.821807 | 0.000327 | 0.014967 | -1 | 105 | 102 | 123 |
| Potri.010 | -0.90361 | 1.610713 | 0.00141 | 0.044624 | -1 | 59 | 46 | 42 |
| Potri.010 | -0.58436 | 3.404225 | 0.000434 | 0.018645 | -1 | 157 | 161 | 169 |
| Potri.010 | -0.80785 | 3.451392 | 4.80E-06 | 0.000527 | -1 | 188 | 157 | 190 |
| Potri.010 | 0.492235 | 4.724016 | 0.001328 | 0.042746 | 1 | 241 | 304 | 304 |
| Potri.010 | -0.6309 | 3.704769 | 0.000127 | 0.00719 | -1 | 213 | 212 | 185 |
| Potri.010 | -1.74895 | 0.262042 | 2.47E-05 | 0.002025 | -1 | 22 | 20 | 20 |
| Potri.010 | -1.39334 | 0.095728 | 0.001351 | 0.043345 | -1 | 15 | 21 | 15 |
| Potri.010 | -1.21689 | 4.890178 | 5.22E-06 | 0.000567 | -1 | 363 | 654 | 573 |
| Potri.010 | 0.417902 | 6.592317 | 0.000222 | 0.011136 | 1 | 1043 | 1087 | 1085 |
| Potri.010 | -0.59125 | 5.402578 | 0.000663 | 0.025736 | -1 | 587 | 826 | 552 |
| Potri.010 | 2.173831 | 1.657517 | 1.75E-12 | 1.34E-09 | 1 | 18 | 13 | 12 |
| Potri.010 | 0.417242 | 5.986752 | 0.00034 | 0.015371 | 1 | 713 | 737 | 664 |
| Potri.010 | -0.40841 | 5.408198 | 0.000931 | 0.033465 | -1 | 639 | 635 | 608 |
| Potri.010 | -4.64696 | -0.63094 | 1.41E-07 | 2.88E-05 | -1 | 13 | 17 | 6 |
| Potri.010 | 0.708616 | 4.89776 | 3.12E-06 | 0.000371 | 1 | 362 | 264 | 259 |
| Potri.010 | 0.599384 | 3.015242 | 0.000268 | 0.012871 | 1 | 86 | 76 | 85 |
| Potri.010 | -0.84061 | 4.070334 | 6.48E-07 | 0.000102 | -1 | 314 | 219 | 302 |
| Potri.010 | -0.70568 | 6.412507 | 7.30E-05 | 0.004783 | -1 | 1009 | 1386 | 1683 |
| Potri.010 | -0.54821 | 6.453177 | 5.66E-06 | 0.000608 | -1 | 1271 | 1409 | 1362 |
| Potri.010 | 0.655169 | 4.425667 | 1.62E-06 | 0.000213 | 1 | 243 | 181 | 226 |

| | | | | | | | | |
|---|---|---|---|---|---|---|---|---|
| Potri.010 | -0.57923 | 5.471732 | 2.01E-06 | 0.000256 | -1 | 816 | 611 | 650 |
| Potri.010 | -0.40018 | 5.627239 | 0.001069 | 0.036992 | -1 | 859 | 640 | 699 |
| Potri.010 | -0.66664 | 8.025793 | 0.000152 | 0.008207 | -1 | 3827 | 4425 | 4174 |
| Potri.010 | -1.31428 | 4.374748 | 1.47E-09 | 5.81E-07 | -1 | 497 | 232 | 425 |
| Potri.010 | -1.18662 | 4.602913 | 2.32E-07 | 4.36E-05 | -1 | 311 | 493 | 491 |
| Potri.010 | -0.61436 | 5.306756 | 5.79E-06 | 0.000617 | -1 | 650 | 623 | 588 |
| Potri.010 | -1.25987 | 1.895245 | 1.16E-05 | 0.001082 | -1 | 48 | 69 | 77 |
| Potri.010 | -0.99277 | 3.842518 | 5.85E-08 | 1.34E-05 | -1 | 212 | 267 | 253 |
| Potri.010 | -0.48642 | 5.948115 | 0.001087 | 0.03734 | -1 | 795 | 1027 | 966 |
| Potri.010 | -0.84388 | 2.806642 | 4.26E-06 | 0.000477 | -1 | 124 | 113 | 105 |
| Potri.010 | -0.38866 | 6.235802 | 0.000849 | 0.031301 | -1 | 1326 | 985 | 1035 |
| Potri.010 | -1.73882 | 0.324406 | 7.02E-05 | 0.004661 | -1 | 19 | 16 | 30 |
| Potri.010 | -0.41868 | 4.543823 | 0.00123 | 0.040439 | -1 | 349 | 324 | 361 |
| Potri.011 | 0.612897 | 4.215865 | 0.000324 | 0.014879 | 1 | 159 | 204 | 204 |
| Potri.011 | -0.92493 | 2.100934 | 0.000181 | 0.009403 | -1 | 63 | 60 | 87 |
| Potri.011 | -0.87764 | 3.08206 | 9.92E-06 | 0.000947 | -1 | 177 | 120 | 124 |
| Potri.011 | -0.51258 | 4.120704 | 0.000566 | 0.022628 | -1 | 255 | 270 | 264 |
| Potri.011 | -0.9941 | 2.614251 | 3.21E-05 | 0.002498 | -1 | 141 | 72 | 98 |
| Potri.011 | -1.04649 | 1.101579 | 0.000368 | 0.016369 | -1 | 38 | 34 | 32 |
| Potri.011 | 0.566079 | 6.106496 | 4.26E-06 | 0.000477 | 1 | 844 | 632 | 699 |
| Potri.011 | -0.74288 | 3.111409 | 0.000147 | 0.008053 | -1 | 176 | 107 | 133 |
| Potri.011 | 0.842869 | 3.023052 | 4.88E-05 | 0.003482 | 1 | 66 | 76 | 81 |
| Potri.011 | 0.948361 | 3.224493 | 2.35E-06 | 0.000295 | 1 | 74 | 103 | 68 |
| Potri.011 | 0.553241 | 4.925446 | 0.000904 | 0.032814 | 1 | 293 | 259 | 404 |
| Potri.011 | 1.057643 | 1.163375 | 0.000252 | 0.012264 | 1 | 15 | 22 | 16 |
| Potri.011 | -1.1034 | 2.607957 | 1.43E-05 | 0.00128 | -1 | 77 | 118 | 118 |
| Potri.011 | 0.765219 | 2.509178 | 0.001441 | 0.045255 | 1 | 64 | 64 | 33 |
| Potri.011 | -0.65298 | 4.073855 | 0.00018 | 0.009396 | -1 | 359 | 210 | 233 |
| Potri.011 | -0.49131 | 5.873835 | 1.16E-05 | 0.001082 | -1 | 964 | 873 | 834 |
| Potri.011 | -3.76003 | 0.873483 | 7.02E-12 | 4.31E-09 | -1 | 24 | 60 | 35 |
| Potri.011 | 0.603194 | 4.416322 | 3.03E-06 | 0.000363 | 1 | 239 | 211 | 209 |
| Potri.011 | 0.544633 | 4.000386 | 0.000254 | 0.012336 | 1 | 157 | 178 | 168 |
| Potri.012 | -0.3932 | 10.06187 | 8.16E-05 | 0.00521 | -1 | 16456 | 15243 | 15695 |
| Potri.012 | -0.88689 | 3.038988 | 4.51E-06 | 0.000501 | -1 | 123 | 144 | 139 |
| Potri.012 | -0.64652 | 6.521623 | 0.000458 | 0.019503 | -1 | 2082 | 973 | 1374 |
| Potri.012 | -0.49605 | 4.786454 | 8.30E-05 | 0.005247 | -1 | 420 | 403 | 429 |
| Potri.012 | 1.274614 | 1.020006 | 0.000108 | 0.006326 | 1 | 15 | 19 | 9 |
| Potri.012 | -1.84566 | 2.482419 | 1.87E-09 | 7.23E-07 | -1 | 99 | 133 | 96 |
| Potri.012 | -1.08738 | 6.406832 | 4.89E-05 | 0.003483 | -1 | 988 | 1909 | 1539 |
| Potri.012 | 0.621414 | 3.416599 | 8.43E-05 | 0.005288 | 1 | 117 | 92 | 116 |
| Potri.012 | -0.75041 | 7.573216 | 2.96E-09 | 1.09E-06 | -1 | 3864 | 2522 | 3001 |
| Potri.012 | 0.353265 | 7.081593 | 0.001038 | 0.036167 | 1 | 1568 | 1521 | 1548 |
| Potri.012 | 0.574282 | 5.438353 | 7.31E-05 | 0.004783 | 1 | 446 | 494 | 414 |
| Potri.012 | 0.604393 | 5.414095 | 2.32E-05 | 0.001936 | 1 | 400 | 490 | 422 |
| Potri.012 | -0.46393 | 5.302108 | 0.001627 | 0.049307 | -1 | 754 | 496 | 541 |
| Potri.012 | -1.00703 | 2.186514 | 0.000483 | 0.020161 | -1 | 56 | 67 | 104 |
| Potri.012 | 0.50745 | 6.528601 | 1.74E-05 | 0.001518 | 1 | 1055 | 923 | 999 |
| Potri.012 | 0.632683 | 6.334907 | 5.62E-07 | 9.03E-05 | 1 | 786 | 884 | 791 |
| Potri.012 | -0.79176 | 2.982573 | 5.49E-06 | 0.000591 | -1 | 144 | 120 | 119 |
| Potri.012 | 1.052703 | 1.470458 | 0.001125 | 0.038141 | 1 | 25 | 18 | 24 |
| Potri.012 | 0.510321 | 6.134953 | 3.50E-05 | 0.002643 | 1 | 714 | 759 | 781 |
| Potri.012 | 1.829029 | 0.831006 | 1.28E-07 | 2.64E-05 | 1 | 8 | 11 | 9 |
| Potri.012 | -0.60049 | 7.625639 | 3.97E-08 | 9.74E-06 | -1 | 3178 | 2980 | 3125 |
| Potri.012 | -1.14766 | 5.867865 | 5.54E-16 | 7.93E-13 | -1 | 1132 | 1044 | 958 |

| | | | | | | | | |
|---|---|---|---|---|---|---|---|---|
| Potri.013 | -0.5108 | 3.804479 | 0.00117 | 0.039407 | -1 | 260 | 196 | 180 |
| Potri.013 | 0.474356 | 6.529862 | 0.000833 | 0.03086 | 1 | 955 | 1067 | 985 |
| Potri.013 | -0.8561 | 4.916857 | 6.48E-05 | 0.004379 | -1 | 570 | 526 | 417 |
| Potri.013 | 0.463193 | 4.669728 | 0.000592 | 0.023433 | 1 | 334 | 257 | 244 |
| Potri.013 | 1.606807 | 0.119639 | 0.000226 | 0.011225 | 1 | 5 | 7 | 6 |
| Potri.013 | -0.45573 | 7.946559 | 8.80E-05 | 0.00544 | -1 | 4244 | 3099 | 3838 |
| Potri.013 | -0.42816 | 5.967918 | 0.000182 | 0.009439 | -1 | 1052 | 882 | 870 |
| Potri.013 | 0.396749 | 6.288164 | 0.000347 | 0.015566 | 1 | 911 | 885 | 834 |
| Potri.013 | -0.74385 | 4.10534 | 1.32E-05 | 0.001196 | -1 | 363 | 257 | 218 |
| Potri.013 | 0.595917 | 3.251936 | 0.000925 | 0.03329 | 1 | 100 | 96 | 96 |
| Potri.013 | 0.53875 | 3.737291 | 0.000538 | 0.021747 | 1 | 139 | 145 | 136 |
| Potri.013 | 0.453424 | 7.431134 | 7.69E-05 | 0.004947 | 1 | 2138 | 1788 | 1775 |
| Potri.013 | -0.84192 | 5.68138 | 2.82E-10 | 1.35E-07 | -1 | 989 | 824 | 758 |
| Potri.013 | 0.531919 | 4.453812 | 0.001578 | 0.048201 | 1 | 285 | 166 | 249 |
| Potri.013 | -1.19111 | 0.950472 | 0.000478 | 0.02004 | -1 | 42 | 21 | 33 |
| Potri.013 | 0.407173 | 5.808473 | 0.000956 | 0.034155 | 1 | 602 | 640 | 631 |
| Potri.013 | 0.798832 | 5.157561 | 8.37E-06 | 0.000836 | 1 | 299 | 391 | 319 |
| Potri.013 | -0.97462 | 4.996132 | 9.27E-06 | 0.000903 | -1 | 592 | 565 | 486 |
| Potri.013 | -0.69383 | 6.827092 | 0.000165 | 0.00871 | -1 | 1780 | 1738 | 1944 |
| Potri.013 | -0.60348 | 5.364709 | 3.47E-05 | 0.002637 | -1 | 560 | 730 | 631 |
| Potri.013 | 1.17666 | 2.483799 | 5.41E-09 | 1.80E-06 | 1 | 47 | 49 | 35 |
| Potri.013 | -1.11286 | 1.819942 | 0.000417 | 0.018015 | -1 | 60 | 60 | 59 |
| Potri.013 | 1.429892 | 3.453524 | 3.40E-08 | 8.54E-06 | 1 | 55 | 115 | 57 |
| Potri.013 | -0.46587 | 6.037852 | 0.001117 | 0.037942 | -1 | 1039 | 1012 | 917 |
| Potri.013 | -0.3821 | 7.79998 | 0.000816 | 0.030315 | -1 | 3628 | 3013 | 3224 |
| Potri.013 | 0.534304 | 5.201606 | 1.69E-05 | 0.001485 | 1 | 437 | 348 | 388 |
| Potri.013 | -1.02506 | 2.14368 | 1.89E-06 | 0.000244 | -1 | 79 | 76 | 67 |
| Potri.013 | -1.5755 | 0.146643 | 0.000322 | 0.014773 | -1 | 21 | 17 | 17 |
| Potri.013 | -0.69388 | 3.145737 | 4.65E-05 | 0.003371 | -1 | 155 | 136 | 128 |
| Potri.014 | 0.716847 | 2.594026 | 0.000968 | 0.034471 | 1 | 70 | 46 | 59 |
| Potri.014 | 0.669176 | 4.550684 | 1.38E-05 | 0.001247 | 1 | 266 | 217 | 222 |
| Potri.014 | 0.781881 | 6.229861 | 0.000157 | 0.008405 | 1 | 510 | 893 | 725 |
| Potri.014 | 1.224587 | 3.60906 | 3.58E-07 | 6.33E-05 | 1 | 58 | 102 | 120 |
| Potri.014 | 0.709614 | 3.013388 | 4.07E-05 | 0.003004 | 1 | 74 | 84 | 77 |
| Potri.014 | -0.76166 | 3.881969 | 0.000397 | 0.017311 | -1 | 325 | 160 | 237 |
| Potri.014 | 0.786005 | 2.384515 | 0.00015 | 0.008156 | 1 | 52 | 37 | 57 |
| Potri.014 | 0.537689 | 5.836482 | 0.000241 | 0.011806 | 1 | 629 | 521 | 668 |
| Potri.014 | 0.431059 | 5.093975 | 0.000923 | 0.033266 | 1 | 443 | 346 | 347 |
| Potri.014 | -0.61413 | 4.93841 | 0.000472 | 0.019883 | -1 | 552 | 421 | 472 |
| Potri.014 | -1.11171 | 3.82359 | 1.76E-07 | 3.43E-05 | -1 | 219 | 275 | 248 |
| Potri.014 | -0.4238 | 6.60115 | 0.000113 | 0.006556 | -1 | 1451 | 1454 | 1426 |
| Potri.014 | -1.44014 | 2.08857 | 3.76E-09 | 1.32E-06 | -1 | 78 | 65 | 89 |
| Potri.014 | -2.04707 | -0.17839 | 0.000108 | 0.006338 | -1 | 12 | 22 | 11 |
| Potri.014 | -0.48258 | 4.585692 | 0.000581 | 0.02309 | -1 | 360 | 354 | 370 |
| Potri.014 | -0.47088 | 4.714936 | 0.000222 | 0.011131 | -1 | 422 | 358 | 405 |
| Potri.014 | -0.97808 | 4.548095 | 1.74E-10 | 8.70E-08 | -1 | 378 | 417 | 403 |
| Potri.014 | -0.61616 | 3.453228 | 0.001166 | 0.039374 | -1 | 147 | 187 | 173 |
| Potri.014 | -1.48352 | 2.996196 | 8.04E-08 | 1.76E-05 | -1 | 107 | 183 | 154 |
| Potri.014 | -1.31084 | 2.194533 | 1.10E-05 | 0.00103 | -1 | 63 | 108 | 72 |
| Potri.014 | -1.21168 | 1.508555 | 5.98E-05 | 0.004113 | -1 | 37 | 48 | 60 |
| Potri.014 | 0.98539 | 2.285671 | 2.94E-05 | 0.002319 | 1 | 40 | 49 | 35 |
| Potri.014 | 1.215104 | 4.779126 | 4.30E-09 | 1.47E-06 | 1 | 155 | 202 | 281 |
| Potri.014 | 0.825607 | 3.090588 | 1.20E-06 | 0.000171 | 1 | 73 | 79 | 84 |
| Potri.014 | -0.67851 | 6.054964 | 1.33E-06 | 0.000182 | -1 | 1374 | 913 | 925 |

| | | | | | | | | |
|---|---|---|---|---|---|---|---|---|
| Potri.014 | 1.805943 | 4.036965 | 2.19E-16 | 3.62E-13 | 1 | 84 | 108 | 90 |
| Potri.014 | -1.34138 | 5.469652 | 2.29E-13 | 2.14E-10 | -1 | 1109 | 565 | 821 |
| Potri.014 | -0.84672 | 2.541504 | 5.15E-05 | 0.003629 | -1 | 111 | 77 | 96 |
| Potri.014 | 0.454242 | 4.609343 | 0.001311 | 0.04237 | 1 | 242 | 294 | 261 |
| Potri.014 | 0.715266 | 2.353442 | 0.00055 | 0.022035 | 1 | 51 | 50 | 46 |
| Potri.014 | -1.20073 | 1.532929 | 1.08E-05 | 0.001015 | -1 | 54 | 41 | 53 |
| Potri.014 | 0.560526 | 5.004431 | 1.48E-05 | 0.001317 | 1 | 343 | 310 | 356 |
| Potri.014 | 0.572474 | 4.379669 | 9.93E-05 | 0.005981 | 1 | 268 | 195 | 190 |
| Potri.014 | -0.48756 | 4.974276 | 0.000556 | 0.022247 | -1 | 538 | 438 | 453 |
| Potri.014 | -1.09296 | 3.211246 | 3.08E-08 | 7.87E-06 | -1 | 187 | 117 | 180 |
| Potri.014 | 0.499522 | 6.38417 | 0.00022 | 0.011063 | 1 | 892 | 1020 | 780 |
| Potri.014 | 0.339452 | 7.34508 | 0.001546 | 0.047352 | 1 | 1921 | 1897 | 1780 |
| Potri.014 | -1.05836 | 3.301683 | 1.79E-06 | 0.000233 | -1 | 148 | 186 | 174 |
| Potri.014 | -1.11794 | 1.287278 | 0.000287 | 0.013516 | -1 | 35 | 40 | 46 |
| Potri.015 | 0.785063 | 2.410629 | 0.00122 | 0.040267 | 1 | 54 | 44 | 51 |
| Potri.015 | -0.91693 | 2.963801 | 1.21E-05 | 0.001115 | -1 | 110 | 156 | 121 |
| Potri.015 | -1.93473 | 2.126491 | 2.45E-12 | 1.81E-09 | -1 | 72 | 106 | 79 |
| Potri.015 | -1.31197 | 1.961807 | 8.35E-05 | 0.005268 | -1 | 42 | 89 | 74 |
| Potri.015 | -0.4636 | 4.287848 | 0.000757 | 0.028703 | -1 | 319 | 260 | 299 |
| Potri.015 | -1.42592 | 1.101525 | 3.63E-06 | 0.000422 | -1 | 35 | 43 | 34 |
| Potri.015 | -2.93066 | -0.85263 | 5.19E-05 | 0.003652 | -1 | 10 | 9 | 8 |
| Potri.015 | -0.44237 | 5.570131 | 0.000769 | 0.029031 | -1 | 832 | 647 | 659 |
| Potri.015 | -1.26801 | 4.170743 | 2.60E-09 | 9.79E-07 | -1 | 280 | 419 | 277 |
| Potri.015 | 0.397551 | 5.119405 | 0.001244 | 0.040732 | 1 | 437 | 367 | 366 |
| Potri.015 | -0.46265 | 4.480355 | 0.000784 | 0.029428 | -1 | 324 | 311 | 366 |
| Potri.015 | -0.65846 | 3.695195 | 0.000111 | 0.006489 | -1 | 177 | 217 | 214 |
| Potri.015 | 0.464953 | 4.5529 | 0.000633 | 0.024818 | 1 | 287 | 221 | 260 |
| Potri.016 | -0.72581 | 3.269722 | 0.001049 | 0.036462 | -1 | 128 | 190 | 140 |
| Potri.016 | -0.75911 | 4.413281 | 7.63E-08 | 1.69E-05 | -1 | 379 | 339 | 320 |
| Potri.016 | 0.999797 | 3.444303 | 1.12E-07 | 2.34E-05 | 1 | 90 | 93 | 97 |
| Potri.016 | -0.6171 | 6.944742 | 2.49E-06 | 0.000308 | -1 | 1936 | 1943 | 1928 |
| Potri.016 | -1.04324 | 3.040725 | 2.81E-05 | 0.002237 | -1 | 123 | 164 | 134 |
| Potri.016 | -0.84362 | 2.400486 | 0.001269 | 0.041212 | -1 | 71 | 85 | 99 |
| Potri.016 | 0.545251 | 5.539262 | 8.19E-05 | 0.00521 | 1 | 564 | 443 | 471 |
| Potri.016 | 0.419679 | 6.878467 | 0.000917 | 0.033207 | 1 | 1185 | 1406 | 1318 |
| Potri.016 | -0.38953 | 5.025612 | 0.001432 | 0.045063 | -1 | 541 | 430 | 468 |
| Potri.016 | -0.69125 | 6.017547 | 3.20E-09 | 1.18E-06 | -1 | 1009 | 1037 | 1063 |
| Potri.016 | -0.71134 | 5.909298 | 1.22E-06 | 0.000172 | -1 | 1021 | 981 | 903 |
| Potri.016 | -0.76664 | 7.76737 | 7.59E-05 | 0.004905 | -1 | 4343 | 3191 | 3237 |
| Potri.016 | -0.73962 | 2.807925 | 0.000709 | 0.027143 | -1 | 99 | 137 | 96 |
| Potri.016 | -0.53141 | 5.084979 | 5.24E-05 | 0.003678 | -1 | 510 | 507 | 539 |
| Potri.016 | -0.41361 | 7.785919 | 0.000262 | 0.01267 | -1 | 3699 | 2840 | 3333 |
| Potri.016 | -0.45462 | 5.986484 | 0.001517 | 0.046821 | -1 | 884 | 1060 | 899 |
| Potri.016 | -0.50465 | 5.0246 | 8.70E-05 | 0.005401 | -1 | 511 | 514 | 457 |
| Potri.016 | 0.388414 | 5.886369 | 0.000731 | 0.027803 | 1 | 691 | 627 | 679 |
| Potri.017 | -0.34837 | 8.335004 | 0.00161 | 0.048925 | -1 | 4628 | 4818 | 4643 |
| Potri.017 | -1.32692 | 1.336388 | 0.001427 | 0.044997 | -1 | 57 | 28 | 47 |
| Potri.017 | 1.025984 | 1.838813 | 0.000217 | 0.010961 | 1 | 19 | 37 | 32 |
| Potri.017 | 0.616913 | 4.967373 | 7.89E-06 | 0.000799 | 1 | 290 | 342 | 325 |
| Potri.017 | 1.18499 | 3.018621 | 1.04E-05 | 0.000984 | 1 | 82 | 37 | 73 |
| Potri.017 | -1.77243 | 1.961314 | 2.27E-06 | 0.000286 | -1 | 47 | 103 | 72 |
| Potri.017 | -1.91247 | 0.306669 | 3.14E-05 | 0.002451 | -1 | 29 | 13 | 24 |
| Potri.017 | -0.88944 | 4.024208 | 2.65E-09 | 9.89E-07 | -1 | 267 | 291 | 256 |
| Potri.017 | -0.65637 | 4.548705 | 8.08E-07 | 0.000123 | -1 | 437 | 324 | 353 |

| | | | | | | | | |
|---|---|---|---|---|---|---|---|---|
| Potri.017 | 0.55476 | 5.181967 | 8.04E-06 | 0.000809 | 1 | 364 | 381 | 397 |
| Potri.017 | 1.170851 | 2.481422 | 2.48E-08 | 6.69E-06 | 1 | 40 | 36 | 55 |
| Potri.017 | 0.680839 | 3.950798 | 0.001181 | 0.039608 | 1 | 198 | 123 | 142 |
| Potri.017 | -0.94864 | 3.286415 | 3.52E-05 | 0.002659 | -1 | 185 | 110 | 199 |
| Potri.017 | 0.631297 | 3.020163 | 0.001215 | 0.040201 | 1 | 94 | 71 | 80 |
| Potri.017 | -1.45618 | 1.31499 | 7.39E-07 | 0.000115 | -1 | 41 | 50 | 41 |
| Potri.017 | 0.804246 | 7.618499 | 8.10E-13 | 6.69E-10 | 1 | 2000 | 1630 | 1966 |
| Potri.017 | -0.67689 | 5.511981 | 9.21E-07 | 0.000137 | -1 | 861 | 650 | 682 |
| Potri.017 | -0.91837 | 1.99254 | 6.33E-05 | 0.004298 | -1 | 63 | 67 | 64 |
| Potri.018 | 0.531021 | 3.866443 | 0.00014 | 0.007747 | 1 | 162 | 145 | 155 |
| Potri.018 | -2.22333 | 2.191267 | 1.36E-17 | 3.25E-14 | -1 | 84 | 93 | 103 |
| Potri.018 | -1.66886 | 0.130396 | 0.000123 | 0.006997 | -1 | 15 | 19 | 21 |
| Potri.018 | -4.34427 | 1.948238 | 6.85E-14 | 6.84E-11 | -1 | 126 | 27 | 120 |
| Potri.018 | -0.66843 | 4.561609 | 7.81E-05 | 0.004998 | -1 | 315 | 441 | 360 |
| Potri.018 | 0.358558 | 6.213251 | 0.001132 | 0.038344 | 1 | 881 | 782 | 873 |
| Potri.018 | 0.835255 | 6.688023 | 0.000434 | 0.018645 | 1 | 615 | 1320 | 912 |
| Potri.018 | -0.42874 | 6.156778 | 0.000206 | 0.01047 | -1 | 1069 | 1060 | 1057 |
| Potri.018 | -1.05274 | 2.751579 | 1.69E-05 | 0.001485 | -1 | 106 | 134 | 104 |
| Potri.018 | 0.642177 | 4.363848 | 5.08E-05 | 0.003597 | 1 | 203 | 220 | 200 |
| Potri.018 | -0.62701 | 3.209869 | 0.001386 | 0.044218 | -1 | 116 | 153 | 159 |
| Potri.018 | 1.299021 | 3.988258 | 1.81E-08 | 5.07E-06 | 1 | 118 | 109 | 128 |
| Potri.018 | 0.479511 | 7.142851 | 2.46E-05 | 0.002024 | 1 | 1569 | 1445 | 1591 |
| Potri.018 | -0.32683 | 7.833673 | 0.001445 | 0.045275 | -1 | 3410 | 3185 | 3310 |
| Potri.018 | 0.632036 | 2.687348 | 0.000712 | 0.02722 | 1 | 64 | 65 | 64 |
| Potri.018 | 0.545604 | 3.632107 | 0.000404 | 0.017592 | 1 | 158 | 118 | 115 |
| Potri.018 | -1.91472 | 0.450021 | 9.99E-06 | 0.000952 | -1 | 36 | 15 | 23 |
| Potri.018 | -0.50297 | 4.535215 | 0.000121 | 0.006938 | -1 | 410 | 331 | 316 |
| Potri.018 | -0.77088 | 2.652302 | 0.000475 | 0.019922 | -1 | 96 | 123 | 81 |
| Potri.018 | -0.57368 | 4.755013 | 0.000122 | 0.006981 | -1 | 534 | 332 | 396 |
| Potri.018 | 0.532966 | 4.201472 | 0.001241 | 0.040668 | 1 | 182 | 231 | 168 |
| Potri.019 | -0.37564 | 6.969283 | 0.001018 | 0.035607 | -1 | 1766 | 1857 | 1882 |
| Potri.019 | 1.506338 | 4.324928 | 2.82E-23 | 1.73E-19 | 1 | 159 | 124 | 124 |
| Potri.019 | 0.967293 | 2.176073 | 0.001203 | 0.03995 | 1 | 29 | 44 | 42 |
| Potri.019 | -2.0381 | 2.316408 | 2.81E-08 | 7.39E-06 | -1 | 72 | 142 | 84 |
| Potri.019 | -2.38374 | 0.180286 | 4.19E-07 | 7.06E-05 | -1 | 21 | 21 | 21 |
| Potri.019 | -1.27682 | 1.254077 | 4.52E-05 | 0.003296 | -1 | 36 | 36 | 50 |
| Potri.019 | 0.545945 | 5.258999 | 0.001201 | 0.03994 | 1 | 475 | 343 | 399 |
| Potri.019 | -0.63427 | 8.842316 | 5.45E-08 | 1.26E-05 | -1 | 8676 | 6484 | 6748 |
| Potri.019 | -0.78849 | 3.422752 | 1.57E-05 | 0.001398 | -1 | 216 | 137 | 171 |
| Potri.019 | -0.42078 | 7.496723 | 0.000999 | 0.0352 | -1 | 3267 | 2199 | 2652 |
| Potri.019 | -0.68445 | 8.091765 | 2.18E-07 | 4.12E-05 | -1 | 5460 | 3398 | 4369 |
| Potri.019 | -1.63289 | 0.441463 | 4.22E-05 | 0.003097 | -1 | 18 | 24 | 28 |
| Potri.019 | -2.04492 | 3.680571 | 2.04E-08 | 5.69E-06 | -1 | 216 | 377 | 193 |
| Potri.019 | -1.51747 | 3.171228 | 4.42E-12 | 2.92E-09 | -1 | 144 | 194 | 169 |
| Potri.019 | -0.47819 | 5.243822 | 0.000543 | 0.021896 | -1 | 715 | 472 | 539 |
| Potri.019 | -0.56209 | 4.969769 | 2.88E-05 | 0.002276 | -1 | 466 | 510 | 471 |
| Potri.019 | -0.63282 | 4.850596 | 9.75E-06 | 0.000941 | -1 | 506 | 389 | 470 |
| Potri.019 | -1.36685 | 1.397053 | 3.46E-06 | 0.000405 | -1 | 45 | 56 | 37 |
| Potri.019 | -1.07574 | 4.440191 | 2.44E-12 | 1.81E-09 | -1 | 469 | 338 | 338 |
| Potri.019 | -0.91412 | 6.396962 | 3.66E-10 | 1.67E-07 | -1 | 1889 | 1124 | 1317 |
| Potri.T01 | 0.682868 | 5.49821 | 1.67E-05 | 0.00147 | 1 | 530 | 406 | 421 |
| Potri.T02 | 1.070686 | 2.31681 | 1.31E-06 | 0.000181 | 1 | 39 | 51 | 32 |
| Potri.T05 | -0.6433 | 6.326917 | 0.000102 | 0.006147 | -1 | 1306 | 1336 | 1168 |
| Potri.T05 | -0.57638 | 6.323684 | 4.46E-05 | 0.003255 | -1 | 1145 | 1393 | 1181 |

| | | | | | | | | |
|---|---|---|---|---|---|---|---|---|
| Potri.T07 | -0.86985 | 3.387269 | 2.74E-08 | 7.31E-06 | -1 | 198 | 155 | 167 |
| Potri.T08 | -0.39833 | 5.344357 | 0.000673 | 0.026003 | -1 | 610 | 604 | 581 |
| Potri.T09 | 0.590084 | 2.987081 | 0.000923 | 0.033266 | 1 | 86 | 85 | 72 |
| Potri.T09 | -3.52523 | 0.112361 | 4.62E-10 | 2.05E-07 | -1 | 15 | 29 | 21 |
| Potri.T10 | 1.031965 | 7.174474 | 1.38E-14 | 1.64E-11 | 1 | 1367 | 1346 | 994 |
| Potri.T11 | -0.86897 | 3.006554 | 0.000116 | 0.006644 | -1 | 106 | 157 | 131 |
| Potri.T11 | -2.76475 | 2.736297 | 2.65E-28 | 2.27E-24 | -1 | 151 | 159 | 128 |
| Potri.T12 | 1.119137 | 4.327276 | 1.99E-14 | 2.19E-11 | 1 | 199 | 133 | 162 |
| Potri.T13 | -0.98467 | 1.763424 | 5.13E-05 | 0.003621 | -1 | 56 | 51 | 60 |
| Potri.T17 | -5.00992 | -1.65814 | 0.001266 | 0.041193 | -1 | 2 | 7 | 3 |

:q comparison.

| LLJGY.M | LLJGY.N | LLJGY.O | F-R | logFC.y | logCPM.y | PValue.y | FDR.y | OX.WT.LBD |
|---|---|---|---|---|---|---|---|---|
| 39 | 42 | 8 | 0 | -1.32387 | 4.420061 | 0.001833 | 0.015165 | -1 |
| 18 | 25 | 22 | -2 | 1.125945 | 4.536711 | 6.93E-05 | 0.000873 | 1 |
| 18 | 31 | 47 | 0 | 1.631032 | 0.941618 | 1.21E-05 | 0.000183 | 1 |
| 2362 | 2219 | 2424 | 0 | 2.239473 | 3.747017 | 9.50E-19 | 7.89E-17 | 1 |
| 37 | 44 | 51 | 0 | -1.29402 | 2.694702 | 4.22E-08 | 9.99E-07 | -1 |
| 84 | 74 | 110 | 0 | 1.714864 | 0.354332 | 0.002829 | 0.022003 | 1 |
| 929 | 978 | 1160 | 0 | 1.810407 | 3.907885 | 1.50E-16 | 1.00E-14 | 1 |
| 141 | 151 | 163 | 2 | -0.64358 | 2.467035 | 0.002666 | 0.02092 | -1 |
| 674 | 602 | 613 | 2 | -0.64966 | 4.652471 | 3.51E-06 | 5.89E-05 | -1 |
| 1746 | 2058 | 2745 | 0 | 0.661495 | 5.961523 | 3.20E-09 | 8.97E-08 | 1 |
| 141 | 170 | 208 | 0 | 1.580581 | 2.480388 | 3.35E-12 | 1.38E-10 | 1 |
| 307 | 238 | 292 | 0 | -0.77636 | 6.131472 | 5.36E-11 | 1.92E-09 | -1 |
| 136 | 179 | 165 | 0 | -0.44095 | 4.623355 | 0.001527 | 0.012985 | -1 |
| 262 | 326 | 241 | -2 | 1.189151 | 3.107414 | 3.27E-06 | 5.53E-05 | 1 |
| 69 | 73 | 61 | 0 | -1.00234 | 5.270036 | 0.000224 | 0.002448 | -1 |
| 90 | 62 | 64 | 0 | -1.19399 | 3.541406 | 7.17E-06 | 0.000113 | -1 |
| 522 | 617 | 981 | 0 | 1.055392 | 3.884361 | 7.65E-11 | 2.67E-09 | 1 |
| 217 | 245 | 274 | 2 | -0.507 | 3.792504 | 0.00428 | 0.031244 | -1 |
| 92 | 87 | 68 | 0 | -1.1861 | 3.67093 | 3.67E-14 | 1.87E-12 | -1 |
| 249 | 288 | 300 | -2 | 0.411451 | 5.120698 | 0.000219 | 0.002401 | 1 |
| 688 | 587 | 574 | 0 | -0.49734 | 6.461127 | 2.91E-08 | 7.09E-07 | -1 |
| 68 | 111 | 96 | 2 | -0.62463 | 3.016319 | 0.000589 | 0.00568 | -1 |
| 159 | 143 | 146 | 0 | -0.84334 | 5.174593 | 9.74E-05 | 0.001177 | -1 |
| 343 | 413 | 359 | 0 | -0.53912 | 6.84938 | 1.64E-07 | 3.52E-06 | -1 |
| 361 | 349 | 348 | 0 | 0.659665 | 4.25239 | 3.84E-06 | 6.40E-05 | 1 |
| 299 | 262 | 489 | 0 | 3.550998 | -0.80613 | 0.00024 | 0.002599 | 1 |
| 156 | 189 | 182 | -2 | 0.448337 | 4.685058 | 0.002229 | 0.017928 | 1 |
| 1240 | 1298 | 1471 | 0 | 1.776111 | 4.892593 | 5.30E-16 | 3.38E-14 | 1 |
| 821 | 786 | 939 | 0 | 1.699455 | 4.338221 | 4.48E-13 | 2.05E-11 | 1 |
| 279 | 314 | 270 | -2 | 0.480807 | 6.092066 | 5.97E-05 | 0.000763 | 1 |
| 1432 | 1320 | 1589 | 2 | -2.04299 | 7.438249 | 1.11E-79 | 1.77E-76 | -1 |
| 156 | 159 | 186 | -2 | 0.530048 | 5.804562 | 0.00527 | 0.037089 | 1 |
| 10 | 13 | 15 | 0 | -1.69533 | 2.324229 | 4.85E-13 | 2.21E-11 | -1 |
| 1303 | 1179 | 1194 | 2 | -0.49416 | 4.671015 | 0.001521 | 0.012959 | -1 |
| 130 | 160 | 214 | 0 | 0.614026 | 4.104071 | 0.006424 | 0.043817 | 1 |
| 699 | 809 | 731 | 0 | 0.448992 | 4.666434 | 0.000106 | 0.001264 | 1 |
| 1662 | 1888 | 2142 | 0 | 1.157127 | 6.21347 | 1.11E-37 | 3.27E-35 | 1 |
| 2904 | 2788 | 2585 | 2 | -0.24995 | 6.955303 | 0.007337 | 0.048903 | -1 |
| 830 | 817 | 797 | 0 | 0.560728 | 4.662394 | 0.000105 | 0.001249 | 1 |
| 181 | 189 | 103 | 0 | -1.28807 | 5.391702 | 4.69E-08 | 1.10E-06 | -1 |
| 0 | 3 | 2 | 0 | -1.47633 | 1.256833 | 3.17E-05 | 0.000438 | -1 |
| 391 | 579 | 618 | 0 | -0.6767 | 7.485159 | 4.54E-09 | 1.25E-07 | -1 |
| 357 | 281 | 165 | 0 | -1.20599 | 6.521961 | 8.79E-05 | 0.001079 | -1 |
| 1343 | 1351 | 1331 | 0 | 0.333106 | 6.627611 | 9.39E-05 | 0.001141 | 1 |
| 223 | 317 | 385 | 0 | 2.115342 | 1.153443 | 9.25E-06 | 0.000142 | 1 |
| 369 | 335 | 325 | 0 | 1.386872 | 3.086039 | 7.28E-06 | 0.000114 | 1 |
| 133 | 153 | 209 | 2 | -1.0524 | 2.021223 | 2.75E-05 | 0.000384 | -1 |
| 125 | 158 | 147 | 0 | -0.80221 | 6.324685 | 7.83E-14 | 3.86E-12 | -1 |
| 182 | 198 | 195 | 0 | 0.956212 | 3.608688 | 9.50E-10 | 2.83E-08 | 1 |
| 306 | 328 | 342 | 0 | -0.43357 | 4.546511 | 0.001297 | 0.01128 | -1 |
| 573 | 564 | 622 | 0 | -1.11792 | 5.931228 | 8.58E-29 | 1.47E-26 | -1 |

| | | | | | | | | | |
|---|---|---|---|---|---|---|---|---|---|
| 225 | 289 | 298 | 0 | -0.489 | 9.084197 | 0.003982 | 0.029413 | | -1 |
| 603 | 587 | 405 | 0 | -0.70245 | 7.60916 | 0.003182 | 0.024349 | | -1 |
| 228 | 243 | 229 | -2 | 0.33888 | 5.975893 | 0.000755 | 0.007054 | | 1 |
| 74 | 87 | 88 | -2 | 0.960827 | 2.237318 | 5.50E-05 | 0.000711 | | 1 |
| 704 | 635 | 693 | 0 | 0.765876 | 4.479784 | 4.85E-09 | 1.32E-07 | | 1 |
| 156 | 159 | 182 | 0 | -0.45619 | 4.260514 | 0.001179 | 0.010397 | | -1 |
| 684 | 797 | 525 | -2 | 2.262505 | 7.984614 | ######## | ######## | | 1 |
| 140 | 127 | 145 | 2 | -0.75926 | 2.482031 | 0.000188 | 0.002103 | | -1 |
| 314 | 295 | 369 | 2 | -0.36228 | 4.363115 | 0.005361 | 0.037665 | | -1 |
| 6 | 7 | 10 | -2 | 3.486352 | 3.263784 | 9.05E-24 | 1.09E-21 | | 1 |
| 93 | 61 | 50 | 0 | -1.02151 | 4.21126 | 5.36E-08 | 1.24E-06 | | -1 |
| 74 | 74 | 84 | 0 | -0.8742 | 2.269699 | 0.001065 | 0.009545 | | -1 |
| 543 | 529 | 603 | 0 | -1.11891 | 6.181175 | 4.32E-29 | 7.64E-27 | | -1 |
| 73 | 111 | 115 | 0 | 1.393231 | 2.805717 | 2.20E-07 | 4.60E-06 | | 1 |
| 1686 | 1642 | 2024 | 2 | -0.33279 | 6.043345 | 0.001048 | 0.009402 | | -1 |
| 36 | 42 | 13 | 0 | -1.0762 | 4.088812 | 1.18E-05 | 0.000178 | | -1 |
| 5 | 0 | 1 | 0 | -1.43977 | 0.81349 | 0.000225 | 0.002453 | | -1 |
| 896 | 864 | 920 | 0 | 0.319296 | 5.083432 | 0.003015 | 0.023256 | | 1 |
| 18 | 20 | 6 | 0 | -0.97262 | 2.765482 | 0.003653 | 0.027346 | | -1 |
| 49 | 56 | 54 | 0 | -0.83369 | 2.616788 | 3.40E-05 | 0.000464 | | -1 |
| 87 | 107 | 83 | 0 | -1.46238 | 3.830512 | 1.33E-19 | 1.16E-17 | | -1 |
| 59 | 50 | 58 | 0 | -1.36397 | 3.370027 | 6.45E-14 | 3.22E-12 | | -1 |
| 312 | 346 | 331 | 0 | 1.332085 | 4.867344 | 8.96E-18 | 6.76E-16 | | 1 |
| 1051 | 1089 | 1104 | 0 | -0.26448 | 6.73447 | 0.002793 | 0.021765 | | -1 |
| 365 | 356 | 264 | 0 | -0.52039 | 4.132051 | 0.000353 | 0.003628 | | -1 |
| 1575 | 1149 | 1227 | 0 | 1.360651 | 4.365871 | 2.40E-08 | 5.94E-07 | | 1 |
| 6 | 8 | 4 | 0 | -1.05798 | 2.901889 | 0.001113 | 0.00989 | | -1 |
| 146 | 143 | 153 | 0 | -0.70261 | 6.881315 | 1.34E-07 | 2.91E-06 | | -1 |
| 1047 | 1215 | 1337 | -2 | 0.377636 | 7.561883 | 1.82E-05 | 0.000266 | | 1 |
| 28 | 63 | 87 | 0 | 0.683373 | 2.848627 | 0.000665 | 0.006302 | | 1 |
| 438 | 440 | 509 | 2 | -0.43464 | 5.072462 | 0.000113 | 0.00134 | | -1 |
| 745 | 748 | 691 | 0 | -0.65275 | 6.502589 | 7.99E-12 | 3.14E-10 | | -1 |
| 1174 | 1200 | 1878 | 0 | 0.73628 | 6.449071 | 1.24E-08 | 3.20E-07 | | 1 |
| 21 | 26 | 14 | 0 | -1.6578 | 4.114317 | 2.01E-14 | 1.06E-12 | | -1 |
| 67 | 64 | 67 | -2 | 1.12417 | 3.046789 | 4.90E-08 | 1.15E-06 | | 1 |
| 561 | 608 | 664 | 2 | -1.46595 | 5.231706 | 1.42E-32 | 3.18E-30 | | -1 |
| 173 | 163 | 169 | 0 | -0.63554 | 4.507403 | 1.83E-07 | 3.88E-06 | | -1 |
| 84 | 80 | 73 | -2 | 1.041456 | 3.392815 | 8.88E-07 | 1.67E-05 | | 1 |
| 656 | 632 | 750 | 0 | -0.90048 | 7.33163 | 7.93E-14 | 3.90E-12 | | -1 |
| 59 | 48 | 66 | 0 | -1.60495 | 3.881544 | 2.49E-13 | 1.16E-11 | | -1 |
| 447 | 436 | 304 | 0 | -0.61817 | 7.34613 | 0.003737 | 0.027885 | | -1 |
| 83 | 91 | 82 | 0 | -0.82318 | 3.72981 | 3.12E-07 | 6.36E-06 | | -1 |
| 282 | 239 | 225 | 0 | 0.550679 | 3.373288 | 0.001592 | 0.013439 | | 1 |
| 747 | 695 | 711 | 0 | 0.589064 | 4.988473 | 5.01E-06 | 8.13E-05 | | 1 |
| 41 | 42 | 30 | 0 | -0.6814 | 3.512781 | 0.004809 | 0.034452 | | -1 |
| 243 | 250 | 246 | 0 | -0.46202 | 5.812512 | 3.90E-05 | 0.000524 | | -1 |
| 661 | 978 | 672 | 0 | -0.266 | 7.018217 | 0.005624 | 0.039247 | | -1 |
| 2981 | 2309 | 2849 | 2 | -1.26978 | 8.102981 | 4.13E-18 | 3.23E-16 | | -1 |
| 44 | 46 | 34 | 0 | -0.71325 | 3.618393 | 5.18E-06 | 8.38E-05 | | -1 |
| 224 | 214 | 268 | 0 | 0.878073 | 2.576699 | 2.04E-05 | 0.000295 | | 1 |
| 13 | 14 | 16 | 0 | -1.72738 | 2.351605 | 9.65E-15 | 5.25E-13 | | -1 |
| 87 | 110 | 146 | 2 | -0.64162 | 3.555089 | 0.000104 | 0.001239 | | -1 |
| 2236 | 2322 | 2493 | 0 | 0.701603 | 6.596691 | 3.62E-07 | 7.30E-06 | | 1 |
| 17 | 12 | 9 | 0 | -1.29131 | 1.873933 | 0.001265 | 0.011046 | | -1 |

| | | | | | | | | |
|---|---|---|---|---|---|---|---|---|
| 65 | 87 | 65 | 0 | -0.97031 | 3.128493 | 2.88E-06 | 4.92E-05 | -1 |
| 1258 | 1142 | 1158 | 2 | -0.38481 | 4.435552 | 0.004349 | 0.031663 | -1 |
| 135 | 140 | 135 | 0 | -1.23251 | 4.267373 | 7.51E-18 | 5.71E-16 | -1 |
| 1392 | 1119 | 1438 | 2 | -0.72777 | 5.016178 | 3.32E-05 | 0.000455 | -1 |
| 1078 | 1283 | 1172 | 0 | -0.26007 | 6.989957 | 0.006958 | 0.046795 | -1 |
| 181 | 200 | 157 | 0 | -0.69287 | 2.925752 | 0.001266 | 0.011049 | -1 |
| 713 | 693 | 673 | 0 | -0.76814 | 6.46297 | 6.41E-18 | 4.93E-16 | -1 |
| 17 | 17 | 13 | 0 | -1.33256 | 2.86156 | 2.37E-07 | 4.92E-06 | -1 |
| 143 | 185 | 194 | -2 | 0.378125 | 4.221501 | 0.006947 | 0.046754 | 1 |
| 95 | 96 | 173 | 2 | -1.24777 | 4.059338 | 2.56E-11 | 9.46E-10 | -1 |
| 78 | 78 | 106 | 0 | 1.479994 | 2.042203 | 2.01E-05 | 0.00029 | 1 |
| 299 | 267 | 306 | 2 | -0.65832 | 3.82572 | 1.30E-05 | 0.000195 | -1 |
| 139 | 96 | 91 | 0 | 2.909771 | 2.866399 | 5.94E-17 | 4.12E-15 | 1 |
| 2540 | 2041 | 2976 | 2 | -1.70699 | 8.638716 | 1.69E-24 | 2.18E-22 | -1 |
| 475 | 526 | 720 | 2 | -1.23296 | 5.868262 | 1.27E-27 | 2.03E-25 | -1 |
| 1860 | 1672 | 1483 | 0 | 0.2855 | 5.834724 | 0.003794 | 0.028252 | 1 |
| 250 | 280 | 411 | 0 | 0.786784 | 3.937823 | 0.000605 | 0.005806 | 1 |
| 80 | 114 | 82 | 0 | -1.51835 | 2.728557 | 3.82E-14 | 1.95E-12 | -1 |
| 254 | 262 | 185 | 0 | -0.51101 | 4.811348 | 0.00012 | 0.001411 | -1 |
| 1156 | 1134 | 1302 | 2 | -0.2969 | 6.371479 | 0.002537 | 0.020044 | -1 |
| 112 | 123 | 76 | 0 | -0.98129 | 3.818476 | 3.77E-11 | 1.36E-09 | -1 |
| 374 | 448 | 516 | 2 | -0.62132 | 4.726511 | 2.62E-08 | 6.44E-07 | -1 |
| 801 | 1129 | 1318 | 0 | 0.761509 | 5.472101 | 5.60E-08 | 1.30E-06 | 1 |
| 465 | 574 | 461 | 2 | -0.4916 | 5.143505 | 0.000134 | 0.001558 | -1 |
| 255 | 243 | 256 | 0 | -0.59474 | 5.469743 | 2.53E-07 | 5.23E-06 | -1 |
| 423 | 459 | 477 | 0 | -0.5023 | 6.282206 | 9.42E-06 | 0.000144 | -1 |
| 51 | 61 | 63 | 2 | -1.59697 | 1.643718 | 3.35E-08 | 8.07E-07 | -1 |
| 490 | 512 | 690 | 0 | 0.677403 | 3.973411 | 2.41E-06 | 4.18E-05 | 1 |
| 3 | 0 | 5 | 0 | -1.71179 | 1.305226 | 2.73E-07 | 5.62E-06 | -1 |
| 4048 | 4005 | 3278 | 0 | 0.304197 | 6.050836 | 0.005527 | 0.038664 | 1 |
| 7467 | 7625 | 8676 | 2 | -0.77084 | 5.750535 | 2.04E-06 | 3.58E-05 | -1 |
| 160 | 167 | 154 | 0 | -0.56279 | 4.916756 | 0.000282 | 0.002983 | -1 |
| 889 | 958 | 973 | 2 | -0.87567 | 5.697523 | 5.68E-13 | 2.56E-11 | -1 |
| 534 | 506 | 499 | 0 | -0.70845 | 4.62852 | 1.93E-08 | 4.84E-07 | -1 |
| 5 | 2 | 9 | 0 | 8.351434 | 1.414641 | 2.96E-13 | 1.37E-11 | 1 |
| 147 | 146 | 136 | 2 | -0.51564 | 3.29682 | 0.004163 | 0.030526 | -1 |
| 69 | 71 | 77 | 0 | -0.49888 | 5.090164 | 4.55E-05 | 0.000601 | -1 |
| 609 | 535 | 580 | 0 | -0.81336 | 6.748292 | 3.65E-14 | 1.87E-12 | -1 |
| 441 | 516 | 360 | 2 | -0.46533 | 3.981172 | 0.000599 | 0.005757 | -1 |
| 738 | 767 | 884 | 0 | 2.283199 | 1.702605 | 6.54E-10 | 2.01E-08 | 1 |
| 398 | 331 | 356 | 0 | 2.506836 | 1.3042 | 1.91E-10 | 6.34E-09 | 1 |
| 325 | 375 | 334 | 0 | -0.55252 | 5.722264 | 0.000384 | 0.00392 | -1 |
| 5 | 2 | 0 | 0 | -1.03088 | 3.827687 | 0.00227 | 0.018211 | -1 |
| 571 | 578 | 573 | 2 | -0.28761 | 5.181156 | 0.006712 | 0.045442 | -1 |
| 102 | 141 | 113 | -2 | 1.223418 | 5.906738 | 6.46E-23 | 7.15E-21 | 1 |
| 500 | 394 | 553 | 0 | 2.28864 | 1.600582 | 4.25E-08 | 1.01E-06 | 1 |
| 103 | 123 | 165 | 0 | -0.84438 | 6.294651 | 9.92E-05 | 0.001194 | -1 |
| 279 | 260 | 329 | 2 | -0.42269 | 4.759598 | 9.19E-05 | 0.001122 | -1 |
| 54 | 49 | 45 | 0 | -0.8119 | 4.432876 | 3.66E-06 | 6.12E-05 | -1 |
| 4 | 3 | 14 | 0 | -2.56639 | 0.891996 | 1.31E-10 | 4.44E-09 | -1 |
| 2728 | 2890 | 2966 | 2 | -0.46431 | 8.736486 | 6.89E-08 | 1.57E-06 | -1 |
| 1103 | 1025 | 1139 | 2 | -0.62031 | 4.00479 | 3.45E-05 | 0.00047 | -1 |
| 112 | 144 | 91 | -2 | 1.106512 | 1.987807 | 0.001421 | 0.012221 | 1 |
| 443 | 581 | 601 | 0 | 1.169388 | 2.497525 | 1.70E-05 | 0.000249 | 1 |

| | | | | | | | | |
|---|---|---|---|---|---|---|---|---|
| 8002 | 8134 | 7351 | 0 | 0.286678 | 8.21192 | 0.000434 | 0.004354 | 1 |
| 685 | 728 | 745 | -2 | 1.474972 | 6.702549 | 2.47E-50 | 1.54E-47 | 1 |
| 113 | 136 | 168 | -2 | 1.974384 | 3.522898 | 7.23E-20 | 6.45E-18 | 1 |
| 72 | 90 | 58 | -2 | 0.621464 | 3.615683 | 5.89E-05 | 0.000754 | 1 |
| 47 | 36 | 67 | 0 | 1.530548 | 2.355615 | 1.76E-06 | 3.12E-05 | 1 |
| 572 | 471 | 423 | 0 | -0.97084 | 7.173382 | 0.000602 | 0.005785 | -1 |
| 7 | 14 | 8 | 0 | -0.97006 | 3.041521 | 0.006661 | 0.045171 | -1 |
| 3637 | 3424 | 4259 | 0 | -0.55371 | 9.697988 | 5.01E-08 | 1.17E-06 | -1 |
| 126 | 152 | 119 | 0 | 1.553675 | 1.784951 | 3.95E-07 | 7.92E-06 | 1 |
| 242 | 153 | 208 | 0 | 1.898731 | 0.589307 | 1.68E-05 | 0.000246 | 1 |
| 9 | 1 | 2 | 0 | -1.40342 | 2.91785 | 0.000774 | 0.007204 | -1 |
| 1585 | 1390 | 1713 | 2 | -1.48522 | 6.330142 | 2.65E-26 | 3.82E-24 | -1 |
| 1561 | 1465 | 1433 | 0 | 1.058672 | 6.564319 | 1.50E-15 | 8.92E-14 | 1 |
| 85 | 76 | 49 | 0 | -1.47378 | 5.463925 | 1.28E-10 | 4.34E-09 | -1 |
| 49 | 54 | 65 | -2 | 1.499877 | 2.748154 | 4.89E-08 | 1.14E-06 | 1 |
| 88 | 81 | 82 | -2 | 1.753874 | 5.413202 | 1.97E-43 | 8.12E-41 | 1 |
| 78 | 108 | 84 | -2 | 1.846666 | 4.65086 | 2.11E-35 | 5.62E-33 | 1 |
| 117 | 193 | 190 | 0 | 1.484325 | 2.897488 | 2.22E-11 | 8.27E-10 | 1 |
| 48 | 48 | 47 | 0 | -1.11652 | 4.348499 | 3.70E-06 | 6.18E-05 | -1 |
| 441 | 451 | 685 | 0 | 2.230507 | 0.241293 | 0.00613 | 0.042043 | 1 |
| 27 | 16 | 23 | 0 | -5.49297 | 1.915923 | 1.62E-39 | 5.24E-37 | -1 |
| 21 | 17 | 9 | 0 | -2.24232 | 3.57558 | 8.87E-08 | 1.99E-06 | -1 |
| 31 | 26 | 34 | -2 | 2.436102 | 3.574788 | 1.07E-31 | 2.24E-29 | 1 |
| 422 | 448 | 582 | 0 | 0.865923 | 4.304383 | 2.27E-06 | 3.95E-05 | 1 |
| 32 | 40 | 57 | 0 | 2.925491 | -0.66865 | 0.00387 | 0.028733 | 1 |
| 296 | 348 | 343 | 0 | -0.4287 | 5.419218 | 0.000308 | 0.003222 | -1 |
| 5979 | 6193 | 6116 | 2 | -0.43477 | 8.360712 | 4.00E-06 | 6.64E-05 | -1 |
| 296 | 317 | 676 | 0 | 1.121417 | 2.773171 | 0.003493 | 0.026313 | 1 |
| 45 | 47 | 40 | 0 | -1.13819 | 2.087132 | 4.56E-06 | 7.46E-05 | -1 |
| 1588 | 1672 | 1638 | 0 | -0.8349 | 6.633631 | 2.28E-14 | 1.19E-12 | -1 |
| 263 | 191 | 159 | 0 | -1.01653 | 6.78282 | 4.49E-05 | 0.000595 | -1 |
| 1755 | 1927 | 2155 | 0 | 0.389425 | 6.477804 | 0.000158 | 0.001802 | 1 |
| 369 | 327 | 396 | 2 | -0.63865 | 5.276342 | 5.20E-05 | 0.000676 | -1 |
| 161 | 150 | 143 | 0 | -1.4021 | 3.590554 | 8.88E-18 | 6.72E-16 | -1 |
| 76 | 83 | 87 | 0 | -1.13974 | 5.2305 | 2.57E-07 | 5.29E-06 | -1 |
| 873 | 924 | 874 | -2 | 0.900983 | 7.149946 | 2.73E-23 | 3.12E-21 | 1 |
| 64 | 42 | 39 | 0 | -1.64465 | 6.869811 | 1.75E-05 | 0.000256 | -1 |
| 709 | 659 | 739 | 0 | 2.619794 | 3.696887 | 3.85E-22 | 3.99E-20 | 1 |
| 403 | 424 | 599 | 2 | -0.29496 | 6.049602 | 0.002629 | 0.020683 | -1 |
| 1 | 2 | 0 | 0 | -1.88531 | 0.91779 | 1.04E-05 | 0.000159 | -1 |
| 60 | 54 | 67 | -2 | 0.579069 | 3.360519 | 0.000369 | 0.00378 | 1 |
| 4 | 3 | 1 | 0 | -1.63407 | 3.093457 | 1.36E-15 | 8.17E-14 | -1 |
| 2024 | 1847 | 2105 | 2 | -0.28806 | 6.626441 | 0.002299 | 0.018407 | -1 |
| 86 | 97 | 95 | 0 | 1.347237 | 0.476018 | 0.002534 | 0.020024 | 1 |
| 816 | 780 | 865 | 0 | -1.72987 | 6.778062 | 1.65E-46 | 7.88E-44 | -1 |
| 28 | 52 | 23 | 0 | -1.20344 | 4.413616 | 1.07E-05 | 0.000162 | -1 |
| 170 | 173 | 100 | 0 | -1.16189 | 5.998022 | 5.43E-05 | 0.000704 | -1 |
| 9 | 4 | 10 | 0 | -1.26061 | 3.553481 | 3.13E-05 | 0.000432 | -1 |
| 198 | 192 | 203 | 0 | -0.50026 | 4.28193 | 0.000319 | 0.00332 | -1 |
| 553 | 616 | 565 | -2 | 0.85864 | 5.896276 | 4.30E-12 | 1.74E-10 | 1 |
| 103 | 113 | 110 | 0 | 1.471416 | 1.474173 | 0.000281 | 0.002976 | 1 |
| 1982 | 2199 | 1928 | 0 | -1.22129 | 8.499375 | 4.43E-32 | 9.65E-30 | -1 |
| 116 | 80 | 80 | -2 | 0.468505 | 4.019791 | 0.000656 | 0.006223 | 1 |
| 1738 | 1822 | 2153 | 0 | -0.61399 | 8.207769 | 5.15E-12 | 2.07E-10 | -1 |

| | | | | | | | | | |
|---|---|---|---|---|---|---|---|---|---|
| 20 | 18 | 13 | -2 | 0.694039 | 1.991337 | 0.004679 | 0.033706 | | 1 |
| 94 | 71 | 43 | 0 | -1.34628 | 5.569655 | 7.69E-06 | 0.00012 | | -1 |
| 165 | 147 | 232 | 0 | -0.45093 | 5.398593 | 1.03E-05 | 0.000156 | | -1 |
| 528 | 480 | 663 | 0 | 1.76255 | 3.171968 | 1.94E-09 | 5.59E-08 | | 1 |
| 530 | 506 | 736 | 0 | 1.60212 | 3.293423 | 9.07E-08 | 2.03E-06 | | 1 |
| 26 | 34 | 15 | -2 | 0.867274 | 3.787918 | 1.36E-05 | 0.000203 | | 1 |
| 2130 | 2111 | 2011 | 0 | 1.04788 | 5.160181 | 1.68E-11 | 6.36E-10 | | 1 |
| 22 | 11 | 18 | 0 | -1.38048 | 2.509672 | 7.27E-05 | 0.000911 | | -1 |
| 863 | 793 | 688 | 0 | -0.88895 | 6.395159 | 3.86E-14 | 1.97E-12 | | -1 |
| 469 | 523 | 514 | 0 | 3.013392 | 4.778719 | 1.67E-65 | 1.94E-62 | | 1 |
| 651 | 576 | 663 | 0 | 0.886465 | 3.970053 | 9.55E-09 | 2.51E-07 | | 1 |
| 443 | 463 | 421 | 2 | -0.36459 | 6.050021 | 0.000423 | 0.004261 | | -1 |
| 29 | 34 | 24 | 0 | -0.79999 | 2.904058 | 0.004692 | 0.033777 | | -1 |
| 28 | 42 | 36 | -2 | 0.606052 | 3.658879 | 0.000336 | 0.003478 | | 1 |
| 227 | 254 | 333 | 2 | -0.87616 | 4.69507 | 3.52E-14 | 1.81E-12 | | -1 |
| 176 | 170 | 212 | 0 | -0.8781 | 3.634949 | 4.94E-08 | 1.15E-06 | | -1 |
| 161 | 112 | 105 | -2 | 0.52609 | 4.115414 | 0.000225 | 0.002459 | | 1 |
| 959 | 983 | 1147 | 2 | -0.40798 | 5.658246 | 0.002199 | 0.017722 | | -1 |
| 335 | 325 | 468 | -2 | 0.489364 | 6.31642 | 6.35E-06 | 0.000101 | | 1 |
| 1118 | 1108 | 1244 | 2 | -0.29325 | 5.480802 | 0.006151 | 0.04214 | | -1 |
| 4510 | 4786 | 4921 | 0 | 0.334185 | 8.319695 | 3.44E-05 | 0.00047 | | 1 |
| 97 | 80 | 69 | 0 | -0.85915 | 3.971015 | 1.63E-06 | 2.91E-05 | | -1 |
| 1741 | 1617 | 1935 | -2 | 2.100215 | 7.446902 | 3.23E-77 | 4.62E-74 | | 1 |
| 2921 | 3004 | 2877 | 2 | -0.35854 | 7.976971 | 2.07E-05 | 0.000298 | | -1 |
| 18 | 10 | 13 | -2 | 0.596112 | 3.616308 | 0.000386 | 0.003933 | | 1 |
| 163 | 165 | 184 | -2 | 2.003795 | 5.341294 | 4.52E-60 | 4.22E-57 | | 1 |
| 81 | 71 | 82 | -2 | 2.488841 | 5.488593 | 8.10E-48 | 4.35E-45 | | 1 |
| 308 | 275 | 252 | 0 | 0.520192 | 3.68476 | 0.003239 | 0.024726 | | 1 |
| 9 | 6 | 7 | -2 | 2.550998 | 0.754906 | 2.16E-07 | 4.53E-06 | | 1 |
| 162 | 155 | 142 | 2 | -0.9186 | 3.587501 | 5.82E-10 | 1.80E-08 | | -1 |
| 45 | 64 | 51 | 0 | -1.2384 | 5.295516 | 8.95E-15 | 4.90E-13 | | -1 |
| 164 | 125 | 169 | -2 | 0.649863 | 4.756052 | 3.59E-08 | 8.60E-07 | | 1 |
| 3936 | 4107 | 3935 | 0 | 0.296727 | 7.4916 | 0.000569 | 0.005515 | | 1 |
| 293 | 335 | 251 | -2 | 1.486231 | 4.788492 | 7.44E-28 | 1.21E-25 | | 1 |
| 2315 | 2815 | 3823 | 0 | 2.716613 | 5.336918 | 9.67E-47 | 4.72E-44 | | 1 |
| 136 | 124 | 107 | 0 | -0.41424 | 5.011508 | 8.32E-05 | 0.001026 | | -1 |
| 52 | 77 | 67 | 0 | -0.65275 | 7.678889 | 0.000327 | 0.003396 | | -1 |
| 1707 | 1664 | 1764 | 0 | 3.866396 | 1.812111 | 1.55E-15 | 9.18E-14 | | 1 |
| 58 | 36 | 55 | 0 | 0.844949 | 1.785162 | 0.001477 | 0.012636 | | 1 |
| 3606 | 3514 | 3793 | 0 | 0.705302 | 5.983999 | 2.99E-09 | 8.42E-08 | | 1 |
| 624 | 726 | 795 | 0 | -1.58536 | 6.679576 | 6.12E-40 | 2.12E-37 | | -1 |
| 88 | 67 | 25 | 0 | -1.11341 | 5.423299 | 0.000411 | 0.004157 | | -1 |
| 55 | 63 | 61 | 0 | -0.81467 | 3.818989 | 2.32E-07 | 4.84E-06 | | -1 |
| 283 | 255 | 260 | 0 | -0.56635 | 3.749714 | 0.002136 | 0.017293 | | -1 |
| 139 | 171 | 100 | 0 | -1.23518 | 6.497577 | 2.33E-05 | 0.000331 | | -1 |
| 63 | 45 | 77 | 2 | -2.80866 | 2.799947 | 4.11E-26 | 5.77E-24 | | -1 |
| 1216 | 1174 | 1586 | 0 | 0.829681 | 4.537182 | 0.000246 | 0.002655 | | 1 |
| 859 | 818 | 905 | 2 | -0.32829 | 6.05755 | 0.001766 | 0.014714 | | -1 |
| 0 | 0 | 1 | -2 | 2.179146 | -0.50287 | 0.002807 | 0.021865 | | 1 |
| 675 | 817 | 762 | 2 | -0.55544 | 6.069485 | 3.46E-05 | 0.000471 | | -1 |
| 113 | 148 | 123 | 0 | 0.846033 | 2.47351 | 8.47E-05 | 0.001041 | | 1 |
| 533 | 503 | 558 | 0 | 0.488991 | 4.195293 | 0.000834 | 0.007689 | | 1 |
| 6051 | 6007 | 6719 | 2 | -0.37007 | 8.292816 | 1.26E-05 | 0.000189 | | -1 |
| 13 | 10 | 13 | 0 | 3.339379 | 1.090663 | 1.01E-14 | 5.44E-13 | | 1 |

| | | | | | | | | | |
|---|---|---|---|---|---|---|---|---|---|
| 2856 | 3073 | 3487 | 0 | 0.416219 | 6.568715 | 5.86E-05 | 0.000752 | 1 |
| 110 | 103 | 74 | -2 | 0.711906 | 2.910237 | 9.59E-05 | 0.001161 | 1 |
| 1642 | 1762 | 1958 | 0 | -0.40927 | 7.45228 | 1.29E-05 | 0.000193 | -1 |
| 49 | 67 | 41 | 0 | -2.09372 | 4.151581 | 1.05E-31 | 2.22E-29 | -1 |
| 485 | 425 | 299 | -2 | 1.611688 | 6.630229 | 1.23E-31 | 2.57E-29 | 1 |
| 1348 | 1135 | 1241 | 2 | -0.343 | 6.173106 | 0.00075 | 0.007013 | -1 |
| 649 | 557 | 668 | 2 | -0.34553 | 5.520626 | 0.001515 | 0.012913 | -1 |
| 68 | 86 | 114 | 0 | 4.718835 | 3.209736 | 2.54E-35 | 6.72E-33 | 1 |
| 22 | 18 | 20 | 0 | -0.84785 | 3.758031 | 0.000241 | 0.002609 | -1 |
| 2746 | 2746 | 3122 | 0 | -0.51847 | 8.930601 | 3.49E-08 | 8.38E-07 | -1 |
| 869 | 891 | 1115 | 0 | 0.944129 | 5.247297 | 9.87E-05 | 0.001189 | 1 |
| 115 | 98 | 111 | 2 | -2.17505 | 4.004231 | 1.69E-20 | 1.58E-18 | -1 |
| 124 | 140 | 143 | 0 | -0.75519 | 5.769681 | 2.03E-08 | 5.07E-07 | -1 |
| 490 | 501 | 505 | 0 | -0.91467 | 7.122616 | 2.84E-18 | 2.28E-16 | -1 |
| 613 | 627 | 601 | 0 | -0.71477 | 7.205283 | 5.73E-14 | 2.88E-12 | -1 |
| 2 | 5 | 1 | 0 | -1.36737 | 3.027252 | 1.06E-09 | 3.13E-08 | -1 |
| 26 | 21 | 13 | 0 | -1.55891 | 6.699681 | 2.03E-10 | 6.71E-09 | -1 |
| 2534 | 2826 | 3239 | 0 | 1.539413 | 5.240028 | 5.88E-07 | 1.14E-05 | 1 |
| 60 | 63 | 41 | 0 | -0.87682 | 4.75551 | 2.92E-11 | 1.07E-09 | -1 |
| 20 | 11 | 0 | 0 | -1.179 | 5.1338 | 0.00229 | 0.018345 | -1 |
| 496 | 451 | 443 | 2 | -0.60916 | 4.289325 | 5.26E-05 | 0.000683 | -1 |
| 669 | 797 | 821 | -2 | 0.528785 | 6.620911 | 4.82E-07 | 9.50E-06 | 1 |
| 71 | 53 | 36 | 0 | -1.3707 | 5.784631 | 4.67E-06 | 7.62E-05 | -1 |
| 326 | 296 | 277 | -2 | 0.447803 | 5.159513 | 0.001045 | 0.009378 | 1 |
| 3103 | 3319 | 3522 | 0 | 0.702622 | 6.234356 | 1.09E-08 | 2.84E-07 | 1 |
| 16 | 14 | 10 | 0 | -1.03822 | 3.548878 | 4.55E-05 | 0.000601 | -1 |
| 242 | 209 | 153 | 0 | -0.56961 | 4.172996 | 3.57E-05 | 0.000483 | -1 |
| 3311 | 3030 | 2855 | 0 | -0.77873 | 8.779513 | 1.91E-15 | 1.12E-13 | -1 |
| 171 | 198 | 106 | 0 | -1.02638 | 5.34798 | 2.57E-07 | 5.30E-06 | -1 |
| 98 | 84 | 89 | 2 | -0.61563 | 3.216911 | 0.002087 | 0.016946 | -1 |
| 165 | 160 | 191 | 0 | -0.30501 | 5.846626 | 0.005852 | 0.040501 | -1 |
| 636 | 574 | 383 | 0 | -0.89485 | 6.690301 | 0.000452 | 0.004524 | -1 |
| 4 | 7 | 6 | 0 | -1.83125 | 0.963933 | 2.34E-07 | 4.88E-06 | -1 |
| 60 | 79 | 64 | 2 | -1.5634 | 1.246857 | 2.53E-06 | 4.37E-05 | -1 |
| 292 | 279 | 328 | 0 | -0.43374 | 5.11533 | 0.000189 | 0.002107 | -1 |
| 534 | 567 | 522 | 0 | -0.89585 | 5.564457 | 5.24E-19 | 4.44E-17 | -1 |
| 104 | 112 | 68 | 0 | -1.37918 | 6.618521 | 2.25E-05 | 0.00032 | -1 |
| 192 | 220 | 163 | -2 | 1.62561 | 3.250928 | 3.79E-14 | 1.93E-12 | 1 |
| 58 | 48 | 67 | 0 | -0.93447 | 5.292362 | 8.21E-05 | 0.001013 | -1 |
| 83 | 71 | 60 | 0 | -1.58357 | 6.095061 | 1.38E-07 | 2.99E-06 | -1 |
| 7718 | 7589 | 7471 | -2 | 0.660869 | 9.931051 | 2.90E-10 | 9.36E-09 | 1 |
| 23 | 52 | 35 | 0 | -1.0346 | 3.569103 | 6.08E-06 | 9.71E-05 | -1 |
| 638 | 649 | 731 | 0 | -2.30938 | 7.006421 | 5.67E-74 | 7.61E-71 | -1 |
| 15 | 5 | 15 | 0 | 5.417922 | -0.90468 | 0.000191 | 0.002126 | 1 |
| 1355 | 1394 | 1387 | 2 | -0.39667 | 5.441299 | 0.000198 | 0.002193 | -1 |
| 210 | 154 | 140 | 0 | -0.93903 | 4.519984 | 0.000524 | 0.005142 | -1 |
| 78 | 74 | 77 | 0 | -1.05881 | 4.396327 | 9.88E-16 | 6.02E-14 | -1 |
| 361 | 379 | 359 | 0 | -0.71618 | 5.051925 | 4.80E-11 | 1.72E-09 | -1 |
| 134 | 146 | 164 | -2 | 0.69615 | 4.31955 | 4.48E-06 | 7.33E-05 | 1 |
| 932 | 988 | 1109 | 0 | -1.5404 | 7.125068 | 1.16E-32 | 2.64E-30 | -1 |
| 2094 | 2227 | 2656 | 0 | -0.44055 | 9.198353 | 8.32E-05 | 0.001026 | -1 |
| 35 | 69 | 75 | 0 | 3.547631 | 2.244773 | 8.88E-24 | 1.07E-21 | 1 |
| 36 | 23 | 22 | 0 | -1.43374 | 5.308609 | 3.57E-06 | 5.99E-05 | -1 |
| 2963 | 3352 | 3453 | 0 | 0.3321 | 7.251417 | 0.000161 | 0.001835 | 1 |

| | | | | | | | | | |
|---|---|---|---|---|---|---|---|---|---|
| 87 | 71 | 96 | 0 | -0.91347 | 4.237351 | 6.27E-12 | 2.50E-10 | | -1 |
| 106 | 112 | 77 | 0 | -1.13081 | 5.027144 | 0.000535 | 0.005231 | | -1 |
| 4743 | 4834 | 4974 | 0 | 0.284244 | 7.871529 | 0.000723 | 0.006788 | | 1 |
| 307 | 313 | 312 | 0 | 0.772524 | 2.313103 | 0.001323 | 0.011475 | | 1 |
| 78 | 62 | 57 | -2 | 0.603415 | 4.951279 | 9.97E-05 | 0.001199 | | 1 |
| 1047 | 1153 | 999 | 2 | -0.59656 | 5.920687 | 6.93E-09 | 1.85E-07 | | -1 |
| 5302 | 4723 | 3546 | -2 | 0.392368 | 8.010486 | 0.005285 | 0.037172 | | 1 |
| 2262 | 2170 | 2237 | 2 | -0.35705 | 6.576223 | 0.000599 | 0.005757 | | -1 |
| 1320 | 1409 | 1429 | 0 | -0.63386 | 7.388578 | 3.22E-14 | 1.65E-12 | | -1 |
| 826 | 882 | 845 | 0 | -0.53713 | 7.222068 | 7.01E-07 | 1.34E-05 | | -1 |
| 773 | 852 | 895 | 0 | -0.45707 | 7.050275 | 2.18E-05 | 0.000312 | | -1 |
| 1047 | 1246 | 1217 | 0 | -0.40447 | 6.484785 | 1.24E-05 | 0.000186 | | -1 |
| 332 | 410 | 485 | 0 | 1.305213 | 2.76467 | 6.25E-05 | 0.000795 | | 1 |
| 9 | 10 | 0 | 0 | -1.0895 | 3.237796 | 0.000243 | 0.002627 | | -1 |
| 513 | 526 | 479 | -2 | 0.408012 | 6.003454 | 2.67E-05 | 0.000373 | | 1 |
| 372 | 370 | 328 | 2 | -0.57512 | 4.235159 | 0.000101 | 0.001218 | | -1 |
| 63 | 81 | 77 | 0 | -0.98927 | 3.350254 | 5.01E-09 | 1.36E-07 | | -1 |
| 7 | 7 | 5 | 0 | -3.47375 | 4.130768 | 1.72E-21 | 1.72E-19 | | -1 |
| 208 | 225 | 234 | 0 | 1.270047 | 1.484 | 2.14E-05 | 0.000307 | | 1 |
| 45 | 59 | 27 | 0 | -1.60491 | 3.688623 | 5.92E-08 | 1.36E-06 | | -1 |
| 109 | 141 | 135 | 0 | 0.833027 | 1.466773 | 0.003918 | 0.029017 | | 1 |
| 20509 | 16798 | 17000 | 0 | 4.086415 | 3.963698 | 3.59E-18 | 2.82E-16 | | 1 |
| 643 | 753 | 653 | 0 | 0.625073 | 5.329009 | 1.44E-05 | 0.000214 | | 1 |
| 495 | 514 | 606 | 0 | 0.32411 | 4.988307 | 0.002181 | 0.017604 | | 1 |
| 820 | 891 | 1084 | -2 | 0.616401 | 7.594507 | 8.25E-12 | 3.24E-10 | | 1 |
| 1385 | 1179 | 1054 | 2 | -0.6516 | 5.205553 | 8.02E-05 | 0.000993 | | -1 |
| 286 | 318 | 262 | 0 | -1.08029 | 5.692906 | 7.28E-18 | 5.56E-16 | | -1 |
| 14 | 10 | 8 | 0 | -1.80037 | 1.524696 | 7.09E-07 | 1.35E-05 | | -1 |
| 1673 | 1504 | 1933 | 2 | -0.53431 | 5.274052 | 3.24E-07 | 6.58E-06 | | -1 |
| 200 | 251 | 187 | 0 | -0.72062 | 6.725772 | 0.005263 | 0.037048 | | -1 |
| 211 | 204 | 215 | 0 | -1.20147 | 3.2152 | 1.12E-06 | 2.07E-05 | | -1 |
| 24 | 27 | 18 | 0 | -0.72743 | 4.865237 | 1.10E-05 | 0.000167 | | -1 |
| 489 | 455 | 508 | 0 | 0.852853 | 5.712707 | 2.93E-12 | 1.22E-10 | | 1 |
| 134 | 170 | 134 | 0 | -1.277 | 5.142723 | 6.20E-28 | 1.02E-25 | | -1 |
| 193 | 214 | 179 | 0 | 0.570215 | 3.499817 | 0.001328 | 0.011506 | | 1 |
| 218 | 220 | 212 | 0 | -0.61128 | 5.578583 | 0.000369 | 0.00378 | | -1 |
| 1857 | 1621 | 1460 | 2 | -0.54957 | 5.534574 | 7.13E-07 | 1.36E-05 | | -1 |
| 495 | 558 | 716 | 0 | -0.28588 | 7.58728 | 0.003117 | 0.02393 | | -1 |
| 175 | 198 | 271 | 0 | 0.600417 | 3.509828 | 0.000382 | 0.0039 | | 1 |
| 98 | 114 | 118 | 0 | 0.485416 | 3.910049 | 0.000346 | 0.003569 | | 1 |
| 575 | 654 | 721 | 0 | 0.407193 | 4.906693 | 0.000226 | 0.002463 | | 1 |
| 772 | 921 | 785 | 0 | -0.97926 | 5.628743 | 3.52E-18 | 2.76E-16 | | -1 |
| 39 | 48 | 32 | 0 | -1.16247 | 4.012986 | 4.32E-05 | 0.000574 | | -1 |
| 267 | 288 | 294 | 0 | -0.86234 | 5.817405 | 1.30E-08 | 3.35E-07 | | -1 |
| 1335 | 1421 | 1581 | 0 | -0.85449 | 8.039537 | 1.07E-23 | 1.27E-21 | | -1 |
| 289 | 221 | 332 | 0 | 0.896884 | 4.257521 | 0.002138 | 0.017308 | | 1 |
| 35 | 34 | 15 | 0 | -2.33765 | 5.04263 | 2.50E-14 | 1.30E-12 | | -1 |
| 215 | 207 | 224 | 0 | 1.326418 | 2.911487 | 3.26E-11 | 1.19E-09 | | 1 |
| 470 | 440 | 461 | -2 | 2.038009 | 5.105666 | 8.29E-43 | 3.33E-40 | | 1 |
| 566 | 591 | 372 | 0 | -1.08218 | 7.548877 | 6.13E-05 | 0.000782 | | -1 |
| 693 | 555 | 627 | 0 | 0.366228 | 4.475108 | 0.002639 | 0.020742 | | 1 |
| 8 | 5 | 1 | 0 | -1.71926 | 1.431348 | 2.08E-08 | 5.20E-07 | | -1 |
| 4 | 3 | 5 | 0 | -2.19172 | 6.890786 | 3.31E-07 | 6.72E-06 | | -1 |
| 70 | 63 | 47 | 0 | -1.00149 | 5.026286 | 0.000219 | 0.002401 | | -1 |

| | | | | | | | | |
|---|---|---|---|---|---|---|---|---|
| 791 | 914 | 989 | 0 | -0.36284 | 7.600782 | 9.51E-05 | 0.001153 | -1 |
| 264 | 258 | 184 | 0 | -1.2987 | 6.704461 | 1.45E-06 | 2.62E-05 | -1 |
| 882 | 840 | 751 | 0 | -0.52972 | 6.534888 | 8.45E-07 | 1.59E-05 | -1 |
| 1226 | 1128 | 1363 | 2 | -0.40754 | 4.442771 | 0.002641 | 0.020748 | -1 |
| 241 | 336 | 358 | 0 | 2.520881 | 2.482155 | 1.81E-14 | 9.60E-13 | 1 |
| 264 | 169 | 283 | 2 | -1.61643 | 4.628102 | 1.77E-15 | 1.03E-13 | -1 |
| 429 | 510 | 411 | -2 | 0.444756 | 5.65112 | 0.000215 | 0.002363 | 1 |
| 551 | 505 | 528 | 2 | -0.97312 | 4.449313 | 4.84E-12 | 1.95E-10 | -1 |
| 78 | 108 | 88 | 0 | -0.5484 | 5.520728 | 0.000227 | 0.002479 | -1 |
| 561 | 341 | 402 | 2 | -0.65237 | 3.183658 | 0.000379 | 0.003872 | -1 |
| 1284 | 1375 | 1438 | 2 | -2.13469 | 8.149945 | ######## | ######## | -1 |
| 94 | 91 | 76 | 0 | -0.97821 | 4.161214 | 2.55E-07 | 5.26E-06 | -1 |
| 2920 | 2967 | 2820 | 2 | -0.61488 | 8.07256 | 1.88E-13 | 8.93E-12 | -1 |
| 1088 | 977 | 1058 | 0 | 1.201608 | 4.487735 | 0.000135 | 0.001564 | 1 |
| 593 | 633 | 650 | 0 | -0.8408 | 7.465873 | 3.15E-11 | 1.15E-09 | -1 |
| 27 | 46 | 47 | 0 | -0.48489 | 3.321285 | 0.004579 | 0.033087 | -1 |
| 1690 | 1787 | 2325 | 0 | 0.860864 | 7.388256 | 6.55E-23 | 7.24E-21 | 1 |
| 53 | 37 | 23 | 0 | -0.98797 | 6.156287 | 0.001841 | 0.015217 | -1 |
| 59 | 58 | 77 | 0 | -1.10808 | 4.105037 | 2.15E-17 | 1.57E-15 | -1 |
| 148 | 158 | 203 | 0 | 1.053625 | 2.396295 | 0.00019 | 0.002117 | 1 |
| 280 | 326 | 290 | 2 | -0.55391 | 4.386561 | 6.08E-05 | 0.000776 | -1 |
| 88 | 98 | 62 | 0 | -0.53244 | 4.90034 | 0.001921 | 0.015804 | -1 |
| 403 | 421 | 471 | -2 | 0.810429 | 6.541205 | 8.32E-16 | 5.16E-14 | 1 |
| 669 | 674 | 740 | 0 | 0.325518 | 5.306662 | 0.002637 | 0.020727 | 1 |
| 952 | 1000 | 1104 | 0 | 0.585358 | 6.42614 | 9.52E-09 | 2.50E-07 | 1 |
| 30 | 39 | 35 | -2 | 0.859528 | 3.57419 | 9.43E-07 | 1.76E-05 | 1 |
| 26 | 34 | 47 | 0 | 0.800585 | 1.614339 | 0.006197 | 0.042417 | 1 |
| 264 | 230 | 239 | 0 | -0.42482 | 4.151635 | 0.001844 | 0.015233 | -1 |
| 237 | 199 | 271 | 0 | 1.890583 | 2.606553 | 6.81E-13 | 3.05E-11 | 1 |
| 6 | 12 | 5 | 0 | -1.16881 | 4.647522 | 0.000494 | 0.004886 | -1 |
| 833 | 928 | 788 | 0 | -0.51241 | 7.247566 | 1.55E-09 | 4.53E-08 | -1 |
| 85 | 102 | 132 | 0 | 1.053327 | 2.220349 | 0.005009 | 0.035595 | 1 |
| 82 | 73 | 93 | 0 | 0.865246 | 3.031834 | 1.97E-05 | 0.000284 | 1 |
| 60 | 65 | 70 | 0 | -0.72226 | 4.483059 | 3.11E-06 | 5.28E-05 | -1 |
| 33 | 17 | 23 | -2 | 0.738145 | 3.719466 | 0.003575 | 0.026817 | 1 |
| 87 | 116 | 102 | 0 | -0.47806 | 5.038873 | 0.007337 | 0.048903 | -1 |
| 70 | 111 | 106 | 0 | -0.95516 | 5.083104 | 5.24E-07 | 1.03E-05 | -1 |
| 364 | 339 | 424 | 2 | -0.56559 | 4.642269 | 2.87E-05 | 0.000399 | -1 |
| 138 | 116 | 114 | -2 | 0.944712 | 5.976512 | 1.11E-06 | 2.05E-05 | 1 |
| 8 | 5 | 4 | -2 | 0.873948 | 2.161642 | 0.000206 | 0.002277 | 1 |
| 6 | 7 | 5 | 0 | -1.19602 | 1.695672 | 0.001636 | 0.013765 | -1 |
| 273 | 228 | 140 | 0 | -1.1643 | 5.588783 | 9.02E-05 | 0.001104 | -1 |
| 1297 | 1298 | 1442 | 2 | -0.29649 | 6.928177 | 0.002027 | 0.016523 | -1 |
| 426 | 422 | 378 | 0 | -0.59987 | 6.023394 | 4.27E-09 | 1.18E-07 | -1 |
| 75 | 45 | 62 | 0 | 1.400616 | 0.43603 | 0.001991 | 0.016279 | 1 |
| 798 | 882 | 975 | 0 | 0.844049 | 5.142418 | 7.14E-08 | 1.62E-06 | 1 |
| 465 | 411 | 452 | 0 | -0.49307 | 5.619925 | 1.75E-05 | 0.000257 | -1 |
| 0 | 1 | 0 | 0 | -1.42647 | 3.504034 | 1.46E-06 | 2.64E-05 | -1 |
| 364 | 432 | 561 | 0 | 0.810032 | 3.609763 | 7.71E-05 | 0.000959 | 1 |
| 111 | 113 | 127 | 0 | 1.776475 | 2.307122 | 6.93E-13 | 3.10E-11 | 1 |
| 163 | 132 | 139 | -2 | 1.425818 | 3.731262 | 3.64E-06 | 6.09E-05 | 1 |
| 777 | 741 | 848 | 0 | -0.67266 | 6.256662 | 5.14E-13 | 2.34E-11 | -1 |
| 833 | 892 | 873 | 0 | -0.45151 | 6.819647 | 3.71E-07 | 7.46E-06 | -1 |
| 308 | 299 | 351 | 2 | -1.07542 | 5.043376 | 1.07E-15 | 6.51E-14 | -1 |

| | | | | | | | | | |
|---|---|---|---|---|---|---|---|---|---|
| 391 | 427 | 482 | 0 | -0.66684 | 5.824663 | 1.85E-08 | 4.65E-07 | | -1 |
| 526 | 491 | 536 | -2 | 0.590336 | 6.291878 | 6.21E-11 | 2.19E-09 | | 1 |
| 2851 | 2609 | 1839 | 0 | -1.00628 | 7.951045 | 1.55E-10 | 5.23E-09 | | -1 |
| 158 | 133 | 138 | -2 | 4.103005 | 3.658929 | 4.21E-50 | 2.55E-47 | | 1 |
| 191 | 211 | 133 | 0 | -1.05352 | 5.853339 | 0.000614 | 0.005878 | | -1 |
| 425 | 330 | 381 | 0 | -0.37998 | 5.51566 | 0.000573 | 0.005545 | | -1 |
| 27 | 29 | 20 | 0 | -1.08424 | 2.999888 | 1.12E-07 | 2.46E-06 | | -1 |
| 126 | 119 | 100 | 0 | -1.12989 | 5.839006 | 0.00044 | 0.00441 | | -1 |
| 630 | 657 | 585 | 0 | -1.37334 | 5.792628 | 1.39E-12 | 6.03E-11 | | -1 |
| 63 | 60 | 55 | 0 | -0.55499 | 2.983043 | 0.002719 | 0.021299 | | -1 |
| 747 | 790 | 849 | 0 | -0.37442 | 6.392776 | 0.001946 | 0.01599 | | -1 |
| 7 | 6 | 5 | 0 | -1.3081 | 1.805888 | 0.000839 | 0.007725 | | -1 |
| 217 | 254 | 255 | 0 | -0.42776 | 5.160012 | 7.31E-05 | 0.000915 | | -1 |
| 293 | 236 | 287 | 2 | -0.51447 | 4.465668 | 6.23E-05 | 0.000793 | | -1 |
| 30 | 31 | 43 | -2 | 1.757278 | 3.2988 | 1.79E-23 | 2.09E-21 | | 1 |
| 81 | 59 | 73 | -2 | 0.905922 | 4.275618 | 5.63E-10 | 1.75E-08 | | 1 |
| 181 | 176 | 161 | -2 | 0.403263 | 5.460026 | 0.003515 | 0.026435 | | 1 |
| 48 | 42 | 55 | -2 | 0.552671 | 3.779651 | 0.002415 | 0.01919 | | 1 |
| 16 | 12 | 19 | -2 | 1.701457 | 1.719685 | 3.81E-06 | 6.36E-05 | | 1 |
| 1022 | 1022 | 954 | 0 | 0.364214 | 5.113806 | 0.001447 | 0.012419 | | 1 |
| 62 | 84 | 86 | -2 | 0.507106 | 4.534133 | 0.000241 | 0.002601 | | 1 |
| 128 | 96 | 153 | 2 | -1.51862 | 3.988197 | 8.88E-14 | 4.35E-12 | | -1 |
| 153 | 132 | 160 | 2 | -0.99018 | 3.987961 | 4.08E-12 | 1.66E-10 | | -1 |
| 400 | 406 | 516 | 0 | 1.678095 | 1.092359 | 2.37E-06 | 4.11E-05 | | 1 |
| 34 | 32 | 38 | 2 | -0.83214 | 1.510595 | 0.006323 | 0.043179 | | -1 |
| 49 | 49 | 39 | 0 | -0.81322 | 3.49838 | 0.006589 | 0.044798 | | -1 |
| 76 | 80 | 101 | 2 | -1.25146 | 2.634129 | 9.55E-08 | 2.13E-06 | | -1 |
| 145 | 164 | 165 | -2 | 0.616771 | 4.665236 | 3.89E-06 | 6.48E-05 | | 1 |
| 574 | 603 | 601 | -2 | 0.408805 | 6.197088 | 1.03E-05 | 0.000157 | | 1 |
| 3 | 3 | 2 | 0 | -1.39726 | 3.947214 | 0.002321 | 0.01855 | | -1 |
| 284 | 330 | 324 | 2 | -0.71556 | 3.405057 | 0.001598 | 0.013471 | | -1 |
| 208 | 222 | 261 | 2 | -0.72312 | 4.681188 | 1.47E-06 | 2.65E-05 | | -1 |
| 10801 | 11404 | 11613 | 0 | -0.63606 | 10.41442 | 7.09E-15 | 3.92E-13 | | -1 |
| 65 | 80 | 61 | 0 | -0.91166 | 3.856837 | 7.17E-08 | 1.63E-06 | | -1 |
| 816 | 941 | 857 | -2 | 1.46914 | 6.349921 | 5.30E-14 | 2.68E-12 | | 1 |
| 272 | 281 | 279 | 0 | -0.78331 | 4.77378 | 2.29E-10 | 7.49E-09 | | -1 |
| 28 | 41 | 29 | 0 | 2.549981 | -0.0426 | 3.84E-05 | 0.000516 | | 1 |
| 33 | 38 | 14 | 0 | -1.23161 | 4.136806 | 0.000107 | 0.001277 | | -1 |
| 810 | 720 | 429 | 0 | -1.00365 | 6.637265 | 0.000143 | 0.001643 | | -1 |
| 156 | 149 | 163 | 0 | 0.817046 | 2.877949 | 0.000567 | 0.005498 | | 1 |
| 1659 | 1668 | 1870 | -2 | 0.291503 | 7.680952 | 0.004711 | 0.033898 | | 1 |
| 1832 | 1766 | 1958 | 2 | -0.25394 | 6.491808 | 0.005103 | 0.036122 | | -1 |
| 699 | 609 | 577 | 2 | -0.46714 | 4.551564 | 0.000237 | 0.002567 | | -1 |
| 651 | 632 | 589 | 0 | 0.645805 | 4.611771 | 2.57E-07 | 5.29E-06 | | 1 |
| 433 | 393 | 378 | -2 | 0.670954 | 4.326324 | 5.62E-07 | 1.09E-05 | | 1 |
| 38 | 38 | 30 | 0 | -0.79787 | 3.683617 | 0.002015 | 0.016445 | | -1 |
| 1145 | 1258 | 1578 | 0 | 0.830123 | 5.499319 | 8.31E-16 | 5.16E-14 | | 1 |
| 1185 | 1250 | 1145 | 0 | 0.395458 | 5.261567 | 0.001103 | 0.009824 | | 1 |
| 59 | 77 | 71 | 0 | -1.17442 | 4.910586 | 2.09E-23 | 2.44E-21 | | -1 |
| 56 | 26 | 48 | 0 | 1.663939 | 1.131948 | 1.95E-05 | 0.000282 | | 1 |
| 873 | 1007 | 1148 | 2 | -1.31859 | 5.557697 | 7.32E-29 | 1.27E-26 | | -1 |
| 37 | 28 | 29 | 0 | 2.396479 | 0.864001 | 1.38E-07 | 2.99E-06 | | 1 |
| 1857 | 2015 | 1860 | -2 | 1.73722 | 8.190759 | 3.49E-83 | 6.25E-80 | | 1 |
| 459 | 490 | 369 | 0 | -1.03495 | 8.383536 | 1.68E-12 | 7.24E-11 | | -1 |

| | | | | | | | | | |
|---|---|---|---|---|---|---|---|---|---|
| 123 | 144 | 150 | 0 | -0.99849 | 4.311676 | 2.28E-13 | 1.07E-11 | -1 |
| 1429 | 1330 | 1148 | 2 | -0.50286 | 4.209727 | 0.002845 | 0.02209 | -1 |
| 256 | 348 | 172 | 0 | -1.29379 | 4.837862 | 4.04E-20 | 3.66E-18 | -1 |
| 362 | 339 | 373 | 2 | -0.8203 | 4.620079 | 8.68E-11 | 3.00E-09 | -1 |
| 23 | 13 | 16 | 2 | -1.48392 | 2.47178 | 2.78E-07 | 5.69E-06 | -1 |
| 2531 | 2473 | 2599 | -2 | 0.828506 | 7.465772 | 1.90E-10 | 6.32E-09 | 1 |
| 577 | 689 | 685 | -2 | 1.043616 | 6.333535 | 5.35E-21 | 5.16E-19 | 1 |
| 971 | 1100 | 1182 | 2 | -0.31395 | 6.211882 | 0.002395 | 0.019066 | -1 |
| 158 | 153 | 154 | 0 | -1.03476 | 5.785532 | 1.28E-07 | 2.79E-06 | -1 |
| 111 | 130 | 175 | 0 | 0.702614 | 2.621059 | 0.000587 | 0.005666 | 1 |
| 192 | 167 | 214 | 0 | 0.605521 | 2.945966 | 0.002172 | 0.017536 | 1 |
| 2564 | 2257 | 2460 | 0 | 0.714257 | 6.127008 | 1.92E-06 | 3.38E-05 | 1 |
| 428 | 503 | 406 | 0 | -0.91415 | 6.826167 | 6.32E-13 | 2.84E-11 | -1 |
| 290 | 280 | 376 | 0 | 3.132592 | 3.229149 | 8.91E-14 | 4.36E-12 | 1 |
| 12 | 14 | 13 | 0 | -0.92517 | 1.467426 | 0.005454 | 0.038227 | -1 |
| 708 | 846 | 780 | 2 | -1.78063 | 6.253511 | 4.67E-20 | 4.19E-18 | -1 |
| 616 | 413 | 624 | 2 | -1.66709 | 6.02273 | 8.82E-26 | 1.21E-23 | -1 |
| 368 | 209 | 196 | -2 | 1.91773 | 3.59764 | 0.000421 | 0.004242 | 1 |
| 1277 | 1104 | 755 | 0 | -2.5007 | 7.367132 | 2.97E-42 | 1.16E-39 | -1 |
| 398 | 383 | 410 | 0 | -1.10112 | 5.943033 | 1.06E-31 | 2.24E-29 | -1 |
| 91 | 87 | 100 | 0 | 2.466347 | 2.093316 | 5.75E-16 | 3.65E-14 | 1 |
| 25 | 39 | 13 | 0 | -0.84687 | 2.443257 | 0.000326 | 0.003389 | -1 |
| 185 | 175 | 221 | 2 | -2.42568 | 2.795748 | 1.24E-10 | 4.21E-09 | -1 |
| 760 | 663 | 579 | 0 | -0.54493 | 5.735492 | 2.96E-08 | 7.19E-07 | -1 |
| 2041 | 2257 | 2814 | 0 | -0.67716 | 8.392927 | 1.21E-17 | 9.03E-16 | -1 |
| 541 | 533 | 511 | 0 | 0.728304 | 3.276518 | 7.11E-05 | 0.000892 | 1 |
| 35 | 32 | 35 | 0 | -1.1729 | 1.930014 | 2.56E-05 | 0.000361 | -1 |
| 9 | 3 | 5 | 0 | -1.32881 | 1.586433 | 9.66E-06 | 0.000148 | -1 |
| 87 | 74 | 81 | 0 | -0.80564 | 3.515213 | 2.30E-06 | 4.00E-05 | -1 |
| 95 | 69 | 105 | 0 | 0.790388 | 3.453544 | 1.98E-05 | 0.000286 | 1 |
| 376 | 371 | 296 | 0 | 1.931037 | 1.891905 | 3.93E-08 | 9.35E-07 | 1 |
| 1274 | 910 | 1282 | 2 | -1.63768 | 7.250816 | 2.49E-30 | 4.82E-28 | -1 |
| 166 | 220 | 237 | 2 | -1.51994 | 2.494672 | 9.22E-11 | 3.18E-09 | -1 |
| 115 | 121 | 125 | 2 | -1.11094 | 4.735312 | 1.03E-14 | 5.56E-13 | -1 |
| 152 | 119 | 122 | -2 | 0.785561 | 5.134466 | 1.94E-09 | 5.58E-08 | 1 |
| 74 | 79 | 83 | 0 | 1.571519 | 3.327491 | 1.03E-15 | 6.23E-14 | 1 |
| 803 | 667 | 1010 | 0 | 1.460351 | 2.944432 | 6.34E-12 | 2.52E-10 | 1 |
| 404 | 477 | 555 | 0 | 1.915153 | 5.464875 | 7.43E-45 | 3.29E-42 | 1 |
| 320 | 339 | 216 | -2 | 0.753315 | 1.969201 | 0.003048 | 0.023462 | 1 |
| 120 | 125 | 76 | 0 | -0.732 | 4.118365 | 2.81E-05 | 0.000392 | -1 |
| 925 | 1010 | 1100 | 0 | -0.80635 | 6.694805 | 3.94E-19 | 3.37E-17 | -1 |
| 20 | 33 | 27 | -2 | 0.695739 | 4.170588 | 1.37E-05 | 0.000204 | 1 |
| 2 | 4 | 4 | 0 | -0.99186 | 1.004836 | 0.005206 | 0.036744 | -1 |
| 194 | 251 | 286 | 0 | -1.1416 | 4.884204 | 6.18E-24 | 7.56E-22 | -1 |
| 232 | 278 | 292 | 0 | -0.87669 | 5.667742 | 1.27E-17 | 9.42E-16 | -1 |
| 188 | 215 | 167 | 0 | -0.84465 | 6.415275 | 0.004313 | 0.031434 | -1 |
| 103 | 84 | 125 | 0 | -1.19322 | 3.868097 | 1.32E-12 | 5.77E-11 | -1 |
| 61 | 52 | 36 | 0 | -1.26088 | 4.068317 | 1.49E-07 | 3.21E-06 | -1 |
| 32 | 37 | 23 | 0 | -1.0441 | 4.291615 | 0.000193 | 0.002152 | -1 |
| 14 | 20 | 25 | 0 | -0.76965 | 1.682185 | 0.002798 | 0.0218 | -1 |
| 58 | 78 | 96 | 0 | 0.602055 | 3.615022 | 0.001746 | 0.01457 | 1 |
| 369 | 467 | 573 | 0 | 1.747593 | 3.216089 | 1.94E-09 | 5.59E-08 | 1 |
| 127 | 126 | 140 | 0 | 1.072008 | 3.37305 | 1.48E-07 | 3.19E-06 | 1 |
| 595 | 663 | 606 | -2 | 2.62923 | 3.082694 | 6.14E-24 | 7.54E-22 | 1 |

| | | | | | | | | |
|---|---|---|---|---|---|---|---|---|
| 231 | 264 | 444 | 0 | 1.244187 | 2.187522 | 2.23E-05 | 0.000318 | 1 |
| 286 | 346 | 279 | -2 | 6.600154 | 1.5785 | 7.05E-22 | 7.21E-20 | 1 |
| 55 | 48 | 44 | -2 | 1.015599 | 4.302758 | 3.27E-12 | 1.35E-10 | 1 |
| 324 | 341 | 363 | 2 | -1.39238 | 5.617158 | 5.77E-16 | 3.66E-14 | -1 |
| 60 | 81 | 86 | 2 | -1.01806 | 1.899815 | 0.001149 | 0.010171 | -1 |
| 24 | 15 | 21 | 0 | -0.91079 | 2.488737 | 0.000125 | 0.001462 | -1 |
| 468 | 482 | 442 | 2 | -0.97312 | 4.109938 | 2.73E-13 | 1.26E-11 | -1 |
| 266 | 288 | 355 | 0 | 0.843652 | 3.041519 | 1.25E-06 | 2.28E-05 | 1 |
| 244 | 329 | 385 | -2 | 0.959339 | 6.236312 | 3.67E-24 | 4.56E-22 | 1 |
| 61 | 72 | 79 | -2 | 1.293612 | 0.712105 | 0.001646 | 0.013832 | 1 |
| 1221 | 1186 | 1161 | 0 | 0.331451 | 5.91074 | 0.000454 | 0.004541 | 1 |
| 2074 | 2130 | 2451 | 2 | -0.24261 | 7.048706 | 0.00436 | 0.031723 | -1 |
| 97 | 70 | 61 | 0 | -1.27255 | 4.881845 | 4.09E-06 | 6.77E-05 | -1 |
| 24 | 14 | 14 | 0 | -0.76563 | 4.025712 | 0.000537 | 0.005243 | -1 |
| 92 | 88 | 59 | 0 | 1.505961 | 1.087776 | 8.00E-05 | 0.000991 | 1 |
| 62 | 65 | 66 | 0 | -0.96237 | 3.285647 | 3.02E-09 | 8.49E-08 | -1 |
| 22 | 23 | 18 | 0 | -1.13151 | 3.195596 | 0.002434 | 0.019331 | -1 |
| 24 | 32 | 22 | 0 | -1.2233 | 3.036057 | 0.000597 | 0.005744 | -1 |
| 184 | 181 | 232 | 0 | -0.50215 | 5.275179 | 3.09E-06 | 5.24E-05 | -1 |
| 9 | 14 | 16 | -2 | 1.007773 | 1.912288 | 0.006992 | 0.046987 | 1 |
| 2 | 0 | 1 | 0 | -3.62531 | 0.018139 | 6.63E-08 | 1.52E-06 | -1 |
| 482 | 538 | 445 | 0 | -0.25737 | 6.195342 | 0.004896 | 0.034928 | -1 |
| 127 | 148 | 106 | 0 | -1.28172 | 5.514895 | 8.75E-07 | 1.65E-05 | -1 |
| 426 | 463 | 557 | 0 | 0.574386 | 4.895992 | 2.77E-06 | 4.74E-05 | 1 |
| 208 | 218 | 257 | 0 | -0.8131 | 3.834346 | 0.000934 | 0.008492 | -1 |
| 123 | 121 | 118 | 0 | -0.88812 | 4.435311 | 1.24E-06 | 2.27E-05 | -1 |
| 282 | 361 | 350 | 0 | 0.663555 | 3.59606 | 0.003855 | 0.028636 | 1 |
| 100 | 83 | 77 | 0 | -0.89724 | 3.669873 | 4.34E-10 | 1.37E-08 | -1 |
| 184 | 214 | 175 | 0 | -0.90161 | 5.533784 | 6.93E-10 | 2.12E-08 | -1 |
| 198 | 138 | 189 | 2 | -0.77958 | 3.119779 | 0.000295 | 0.003106 | -1 |
| 1244 | 1257 | 1036 | 0 | -0.45071 | 7.183405 | 9.21E-08 | 2.06E-06 | -1 |
| 70 | 79 | 42 | 0 | -0.75848 | 4.35111 | 0.000178 | 0.001998 | -1 |
| 58 | 34 | 41 | 0 | -0.94617 | 3.382514 | 6.78E-08 | 1.55E-06 | -1 |
| 549 | 632 | 847 | 0 | 1.158172 | 4.642511 | 1.02E-14 | 5.51E-13 | 1 |
| 1574 | 1545 | 1811 | 2 | -0.38497 | 6.227181 | 0.000387 | 0.003941 | -1 |
| 341 | 336 | 349 | -2 | 0.380001 | 4.996046 | 0.000503 | 0.004962 | 1 |
| 586 | 579 | 644 | 0 | -0.65406 | 6.704444 | 8.08E-15 | 4.45E-13 | -1 |
| 622 | 570 | 460 | -2 | 0.861746 | 6.040472 | 1.02E-13 | 4.98E-12 | 1 |
| 2348 | 2137 | 1347 | 0 | -0.7742 | 7.154438 | 1.84E-12 | 7.89E-11 | -1 |
| 59 | 67 | 61 | 0 | -0.90081 | 2.104415 | 0.000192 | 0.002138 | -1 |
| 359 | 320 | 329 | 0 | -0.84546 | 4.878141 | 2.18E-07 | 4.55E-06 | -1 |
| 2045 | 2257 | 2645 | 0 | -0.48922 | 8.455187 | 3.06E-09 | 8.62E-08 | -1 |
| 683 | 671 | 591 | -2 | 0.455257 | 5.106305 | 0.000423 | 0.004257 | 1 |
| 322 | 343 | 313 | -2 | 1.37913 | 6.450443 | 1.06E-37 | 3.15E-35 | 1 |
| 750 | 877 | 823 | 2 | -1.35493 | 7.186029 | 1.17E-39 | 3.86E-37 | -1 |
| 3478 | 3331 | 3576 | 0 | -0.43334 | 7.543374 | 2.13E-06 | 3.74E-05 | -1 |
| 27 | 14 | 7 | -2 | 0.731815 | 4.13333 | 0.000421 | 0.004242 | 1 |
| 58 | 60 | 51 | 0 | 1.97215 | 1.042576 | 7.86E-06 | 0.000123 | 1 |
| 405 | 457 | 523 | 2 | -0.77166 | 6.179062 | 9.14E-15 | 4.99E-13 | -1 |
| 156 | 94 | 156 | 0 | 1.277715 | 3.130288 | 8.97E-09 | 2.37E-07 | 1 |
| 24 | 25 | 12 | 0 | -1.20417 | 3.746182 | 0.000956 | 0.008672 | -1 |
| 7 | 7 | 2 | -2 | 1.156301 | 3.888915 | 4.91E-09 | 1.34E-07 | 1 |
| 131 | 127 | 155 | -2 | 1.295424 | 7.53268 | 1.97E-15 | 1.15E-13 | 1 |
| 206 | 225 | 229 | 0 | -0.71877 | 6.40116 | 2.85E-08 | 6.97E-07 | -1 |

| | | | | | | | | | |
|---|---|---|---|---|---|---|---|---|---|
| 506 | 501 | 570 | 0 | 0.568993 | 4.71761 | 3.29E-05 | 0.000452 | | 1 |
| 90 | 93 | 94 | 0 | 2.814229 | 0.142513 | 6.86E-06 | 0.000108 | | 1 |
| 159 | 221 | 318 | 0 | 0.882816 | 4.043685 | 2.27E-10 | 7.45E-09 | | 1 |
| 78 | 93 | 67 | -2 | 2.504566 | 3.624672 | 1.26E-43 | 5.27E-41 | | 1 |
| 93 | 110 | 154 | 0 | 0.804514 | 3.085861 | 0.006903 | 0.046513 | | 1 |
| 11 | 19 | 15 | 0 | -1.00601 | 2.608895 | 0.000491 | 0.004862 | | -1 |
| 3070 | 2950 | 3113 | 0 | 1.209711 | 4.960705 | 5.68E-11 | 2.02E-09 | | 1 |
| 421 | 478 | 377 | -2 | 0.664179 | 5.555447 | 1.07E-09 | 3.18E-08 | | 1 |
| 35 | 32 | 29 | 0 | -0.58081 | 3.43495 | 0.000284 | 0.003 | | -1 |
| 191 | 215 | 220 | 0 | 1.428511 | 2.607032 | 2.36E-09 | 6.73E-08 | | 1 |
| 24 | 13 | 19 | 0 | -1.40246 | 4.923683 | 1.91E-05 | 0.000278 | | -1 |
| 5 | 6 | 5 | 0 | -1.64293 | 3.324698 | 9.61E-14 | 4.69E-12 | | -1 |
| 5 | 5 | 2 | -2 | 4.788822 | -0.03649 | 0.000313 | 0.00327 | | 1 |
| 226 | 223 | 212 | 0 | -2.14059 | 7.233038 | 1.42E-17 | 1.05E-15 | | -1 |
| 972 | 979 | 1098 | 0 | 1.654677 | 4.449748 | 8.67E-35 | 2.20E-32 | | 1 |
| 1762 | 1309 | 1756 | 2 | -1.45919 | 7.868844 | 3.21E-20 | 2.94E-18 | | -1 |
| 654 | 725 | 849 | 0 | -0.9074 | 7.096061 | 1.18E-26 | 1.75E-24 | | -1 |
| 56 | 63 | 36 | 0 | -1.17692 | 3.938111 | 8.11E-09 | 2.16E-07 | | -1 |
| 295 | 248 | 373 | 2 | -1.45712 | 4.0787 | 1.39E-15 | 8.33E-14 | | -1 |
| 88 | 82 | 91 | 0 | -0.62537 | 3.053872 | 0.000565 | 0.00548 | | -1 |
| 170 | 258 | 403 | 0 | 0.974285 | 4.111265 | 4.22E-10 | 1.33E-08 | | 1 |
| 2075 | 1950 | 1981 | 2 | -0.49302 | 5.372862 | 5.52E-05 | 0.000713 | | -1 |
| 2260 | 2507 | 2647 | 0 | -0.47504 | 8.633002 | 5.57E-08 | 1.29E-06 | | -1 |
| 90 | 84 | 107 | 2 | -0.73192 | 2.404049 | 0.002123 | 0.017202 | | -1 |
| 176 | 172 | 185 | 2 | -0.55295 | 3.769134 | 0.004517 | 0.032702 | | -1 |
| 5 | 6 | 7 | 0 | -1.6448 | 1.91404 | 1.21E-07 | 2.66E-06 | | -1 |
| 222 | 231 | 244 | -2 | 0.47653 | 4.104534 | 0.000431 | 0.004332 | | 1 |
| 56 | 54 | 55 | 0 | -1.84694 | 6.131724 | 1.30E-66 | 1.55E-63 | | -1 |
| 247 | 252 | 291 | -2 | 1.095374 | 6.239375 | 7.09E-17 | 4.87E-15 | | 1 |
| 229 | 289 | 274 | 2 | -0.71936 | 3.177481 | 0.000169 | 0.00191 | | -1 |
| 1313 | 1282 | 1391 | 0 | -0.95788 | 7.337269 | 8.40E-24 | 1.02E-21 | | -1 |
| 365 | 308 | 409 | 0 | 1.453977 | 1.486294 | 0.000516 | 0.005069 | | 1 |
| 63 | 48 | 103 | 0 | 1.089785 | 0.959711 | 0.007111 | 0.047708 | | 1 |
| 25 | 31 | 12 | 0 | -1.42737 | 5.652082 | 5.82E-08 | 1.34E-06 | | -1 |
| 4 | 7 | 0 | 0 | -1.39563 | 2.738859 | 2.08E-05 | 0.000299 | | -1 |
| 20 | 11 | 16 | 0 | -1.02046 | 2.225724 | 0.002023 | 0.016497 | | -1 |
| 390 | 553 | 731 | 0 | 0.42037 | 6.176011 | 3.22E-05 | 0.000444 | | 1 |
| 3885 | 4772 | 4520 | -2 | 1.215546 | 9.50909 | 2.08E-36 | 5.77E-34 | | 1 |
| 96 | 100 | 86 | -2 | 1.241369 | 1.541218 | 0.000305 | 0.003201 | | 1 |
| 1605 | 1928 | 2137 | -2 | 0.300388 | 8.563322 | 0.000114 | 0.001346 | | 1 |
| 2398 | 2583 | 2679 | -2 | 0.974033 | 8.158792 | 2.25E-23 | 2.62E-21 | | 1 |
| 6 | 7 | 8 | 0 | -1.26191 | 1.057107 | 0.004903 | 0.034973 | | -1 |
| 84 | 67 | 26 | 0 | -1.92877 | 5.50365 | 2.23E-12 | 9.50E-11 | | -1 |
| 63 | 58 | 45 | 0 | -1.14738 | 4.954139 | 2.05E-06 | 3.60E-05 | | -1 |
| 375 | 350 | 430 | -2 | 1.261346 | 5.967904 | 2.15E-32 | 4.80E-30 | | 1 |
| 289 | 335 | 296 | -2 | 1.397243 | 6.763732 | 6.41E-26 | 8.95E-24 | | 1 |
| 217 | 278 | 332 | 0 | -0.37599 | 5.60847 | 0.000357 | 0.003667 | | -1 |
| 18 | 15 | 17 | 0 | -1.64504 | 4.056133 | 2.83E-09 | 8.02E-08 | | -1 |
| 170 | 182 | 153 | -2 | 1.500071 | 4.782176 | 1.20E-18 | 9.87E-17 | | 1 |
| 686 | 728 | 718 | -2 | 0.724615 | 8.37457 | 1.07E-09 | 3.18E-08 | | 1 |
| 594 | 547 | 907 | 0 | 0.687321 | 4.684328 | 0.003526 | 0.026503 | | 1 |
| 76 | 82 | 83 | 0 | 1.714674 | 1.893251 | 4.94E-08 | 1.15E-06 | | 1 |
| 879 | 805 | 585 | 0 | -0.66445 | 7.339803 | 5.60E-05 | 0.000723 | | -1 |
| 775 | 847 | 720 | 0 | -1.06272 | 6.484254 | 2.43E-22 | 2.56E-20 | | -1 |

| | | | | | | | | |
|---|---|---|---|---|---|---|---|---|
| 84 | 88 | 94 | -2 | 1.589608 | 2.816507 | 5.64E-10 | 1.75E-08 | 1 |
| 413 | 428 | 436 | -2 | 0.78633 | 2.476828 | 0.000161 | 0.001835 | 1 |
| 117 | 100 | 126 | 0 | 0.460904 | 3.35123 | 0.004559 | 0.032954 | 1 |
| 2 | 1 | 2 | 0 | -1.81905 | 2.942898 | 4.61E-06 | 7.53E-05 | -1 |
| 2136 | 2251 | 2735 | 2 | -0.33802 | 5.535345 | 0.003972 | 0.029354 | -1 |
| 77 | 57 | 69 | 0 | -1.12452 | 4.256548 | 7.92E-06 | 0.000123 | -1 |
| 19 | 29 | 12 | 0 | -0.92703 | 5.249842 | 0.000531 | 0.005198 | -1 |
| 308 | 314 | 382 | 0 | 1.977562 | 3.382459 | 4.13E-17 | 2.94E-15 | 1 |
| 21 | 31 | 27 | 0 | -0.71724 | 1.985954 | 0.003996 | 0.029505 | -1 |
| 0 | 0 | 0 | 0 | -2.07554 | -0.31786 | 0.002503 | 0.019826 | -1 |

| LLJYT03G | LLJYT03H | LLJYT03I | LLJYT03M | LLJYT03N | LLJYT03O | Best.hit.arabi.sym | arabi.def |
|---|---|---|---|---|---|---|---|
| 243 | 216 | 296 | 167 | 156 | 21 | AT3G60130 | BGLU16 | beta gluc |
| 117 | 155 | 87 | 317 | 439 | 123 | AT3G45140 | ATLOX2, LC | lipoxygen |
| 10 | 3 | 7 | 21 | 29 | 19 | AT3G45140 | ATLOX2, LC | lipoxygen |
| 47 | 29 | 37 | 125 | 247 | 217 | AT3G20820.1 | | Leucine-r |
| 58 | 91 | 76 | 37 | 44 | 20 | AT2G41560 | ACA4 | autoinhib |
| 8 | 3 | 1 | 14 | 9 | 20 | AT3G13650.1 | | Disease r |
| 44 | 72 | 47 | 155 | 220 | 244 | AT1G15960 | ATNRAMP6, | NRAMP met |
| 59 | 58 | 46 | 30 | 43 | 41 | AT1G80760 | NIP6, NIP6 | NOD26-lik |
| 307 | 242 | 215 | 164 | 200 | 169 | AT1G04770.1 | | Tetratric |
| 373 | 486 | 346 | 615 | 793 | 671 | AT4G13710.1 | | Pectin ly |
| 18 | 29 | 19 | 67 | 75 | 74 | AT5G60020 | ATLAC17, L | laccase 1 |
| 834 | 822 | 565 | 466 | 535 | 414 | AT1G27920 | MAP65-8 | microtubu |
| 234 | 250 | 221 | 209 | 233 | 131 | AT2G33550.1 | | Homeodoma |
| 49 | 30 | 46 | 77 | 157 | 86 | AT5G32450.1 | | RNA bindi |
| 456 | 403 | 420 | 307 | 321 | 91 | AT4G24140.1 | | alpha/bet |
| 137 | 115 | 147 | 90 | 74 | 30 | AT4G24130.1 | | Protein o |
| 76 | 82 | 76 | 142 | 234 | 161 | AT1G64390 | AtGH9C2, G | glycosyl |
| 121 | 150 | 131 | 89 | 99 | 119 | AT5G41460.1 | | Protein o |
| 127 | 170 | 142 | 68 | 78 | 65 | AT5G23750.1 | | Remorin f |
| 269 | 243 | 228 | 358 | 427 | 298 | AT1G63020 | NRPD1, NRP | nuclear R |
| 897 | 899 | 780 | 611 | 771 | 618 | AT1G62990 | IXR11, KNA | KNOTTED-1 |
| 71 | 89 | 80 | 60 | 56 | 54 | AT4G22890 | PGR5-LIKE | PGR5-LIKE |
| 484 | 369 | 304 | 285 | 306 | 126 | AT4G12350 | AtMYB42, M | myb domai |
| 1208 | 1297 | 922 | 783 | 1017 | 779 | AT1G62710 | BETA-VPE, | beta vacu |
| 145 | 123 | 96 | 200 | 228 | 200 | AT1G31920.1 | | Tetratric |
| 1 | 0 | 0 | 5 | 5 | 7 | | | |
| 192 | 152 | 191 | 257 | 339 | 214 | AT1G32540 | LOL1 | lsd one l |
| 98 | 138 | 96 | 253 | 501 | 484 | AT3G14310 | ATPME3, PM | pectin me |
| 53 | 97 | 83 | 177 | 355 | 300 | AT3G14310 | ATPME3, PM | pectin me |
| 449 | 522 | 447 | 756 | 905 | 527 | AT1G19110.1 | | inter-alp |
| 2331 | 2209 | 2413 | 559 | 702 | 594 | AT5G07990 | CYP75B1, D | Cytochrom |
| 446 | 327 | 362 | 567 | 487 | 708 | AT3G15990 | SULTR3;4 | sulfate t |
| 60 | 62 | 64 | 24 | 27 | 12 | AT3G16360 | AHP4 | HPT phosp |
| 255 | 294 | 195 | 175 | 184 | 211 | AT3G21360.1 | | 2-oxoglut |
| 152 | 97 | 86 | 150 | 181 | 223 | AT1G72970 | EDA17, HTH | Glucose-m |
| 185 | 188 | 158 | 267 | 280 | 245 | AT1G15760.1 | | Sterile a |
| 380 | 415 | 348 | 914 | 1034 | 841 | AT5G64570 | ATBXL4, XY | beta-D-xy |
| 1108 | 1118 | 1131 | 943 | 1222 | 943 | AT1G15820 | CP24, LHCB | light har |
| 189 | 182 | 136 | 247 | 274 | 290 | AT5G21105.1 | | Plant L-a |
| 385 | 515 | 579 | 217 | 354 | 117 | AT1G20030.2 | | Pathogene |
| 33 | 22 | 26 | 14 | 15 | 3 | AT3G60120 | BGLU27 | beta gluc |
| 1912 | 1941 | 1660 | 1395 | 1485 | 921 | AT2G44500.1 | | O-fucosyl |
| 1205 | 875 | 1101 | 622 | 772 | 180 | AT5G58860 | CYP86, CYP | cytochrom |
| 751 | 751 | 679 | 966 | 1155 | 894 | AT2G13360 | AGT, AGT1, | alanine:g |
| 9 | 3 | 6 | 13 | 37 | 37 | AT1G74670.1 | | Gibberell |
| 50 | 27 | 36 | 68 | 109 | 144 | AT4G36250 | ALDH3F1 | aldehyde |
| 38 | 49 | 44 | 16 | 27 | 26 | AT5G58600 | PMR5, TBL4 | Plant pro |
| 916 | 859 | 770 | 532 | 668 | 412 | AT1G07120.1 | | |
| 60 | 76 | 66 | 129 | 158 | 142 | AT2G14520.1 | | CBS domai |
| 230 | 222 | 214 | 165 | 243 | 138 | AT4G34160 | CYCD3, CYC | CYCLIN D3 |
| 692 | 697 | 694 | 322 | 397 | 334 | AT5G03170 | ATFLA11, F | FASCICLIN |

| | | | | | | | | |
|---|---|---|---|---|---|---|---|---|
| 6314 | 4302 | 5139 | 4629 | 3557 | 3992 | AT3G02040 | SRG3 | senescenc |
| 2238 | 1859 | 1926 | 1662 | 1885 | 624 | AT5G41040.1 | | HXXXD-typ |
| 475 | 497 | 413 | 557 | 785 | 583 | AT3G01990 | ACR6 | ACT domai |
| 20 | 23 | 32 | 54 | 62 | 45 | AT3G27027.1 | | Protein o |
| 126 | 148 | 133 | 218 | 289 | 251 | AT1G60550 | DHNS, ECHI | enoyl-CoA |
| 187 | 211 | 153 | 154 | 178 | 109 | AT5G40780 | LHT1 | lysine hi |
| 683 | 805 | 692 | 3868 | 4460 | 3171 | AT3G27400.1 | | Pectin ly |
| 61 | 58 | 51 | 31 | 44 | 35 | AT3G01660.1 | | S-adenosy |
| 185 | 197 | 191 | 175 | 170 | 143 | AT5G14460.1 | | Pseudouri |
| 10 | 17 | 10 | 182 | 213 | 72 | AT5G14650.1 | | Pectin ly |
| 196 | 215 | 205 | 122 | 154 | 62 | AT4G18170 | ATWRKY28, | WRKY DNA- |
| 48 | 44 | 58 | 32 | 23 | 34 | AT3G13130.1 | | |
| 877 | 886 | 725 | 421 | 502 | 336 | AT5G55970.1 | | RING/U-bo |
| 33 | 39 | 21 | 96 | 68 | 98 | AT3G58120 | ATBZIP61, | Basic-leu |
| 634 | 643 | 557 | 527 | 544 | 515 | AT1G53240 | mMDH1 | Lactate/m |
| 223 | 178 | 171 | 117 | 142 | 45 | AT3G62730.1 | | |
| 20 | 16 | 21 | 9 | 12 | 2 | AT1G68470.1 | | Exostosin |
| 244 | 267 | 237 | 355 | 364 | 302 | AT1G78630 | emb1473 | Ribosomal |
| 87 | 58 | 73 | 45 | 67 | 14 | AT1G53270.1 | | ABC-2 typ |
| 62 | 70 | 59 | 42 | 38 | 37 | AT5G53900.2 | | Serine/th |
| 163 | 181 | 175 | 67 | 94 | 47 | AT4G27460.1 | | Cystathio |
| 110 | 144 | 115 | 62 | 59 | 36 | AT5G53750.1 | | CBS domai |
| 143 | 145 | 122 | 332 | 363 | 422 | AT5G54250 | ATCNGC4, C | cyclic nu |
| 1022 | 1029 | 854 | 850 | 1044 | 761 | AT5G54690 | GAUT12, IR | galacturo |
| 166 | 185 | 161 | 143 | 158 | 92 | AT1G78380 | ATGSTU19, | glutathio |
| 113 | 87 | 85 | 285 | 182 | 314 | AT1G79040 | PSBR | photosyst |
| 84 | 80 | 82 | 55 | 65 | 13 | AT4G21390 | B120 | S-locus l |
| 1185 | 1107 | 1333 | 872 | 993 | 611 | AT2G36840.1 | | ACT-like |
| 1407 | 1514 | 1192 | 1935 | 2300 | 1630 | AT4G10550.1 | | Subtilase |
| 52 | 43 | 38 | 84 | 74 | 75 | AT2G34780 | EMB1611, M | maternal |
| 340 | 347 | 277 | 277 | 283 | 220 | AT1G55690.1 | | Sec14p-li |
| 976 | 924 | 866 | 618 | 787 | 535 | AT4G35550 | ATWOX13, H | WUSCHEL r |
| 576 | 561 | 496 | 970 | 916 | 1053 | AT1G76160 | sks5 | SKU5 simi |
| 203 | 192 | 257 | 94 | 92 | 44 | AT1G60420.1 | | DC1 domai |
| 39 | 50 | 36 | 113 | 118 | 69 | AT3G21570.1 | | |
| 482 | 490 | 408 | 198 | 171 | 174 | AT1G19940 | AtGH9B5, G | glycosyl |
| 245 | 224 | 218 | 162 | 189 | 135 | AT2G16400 | BLH7 | BEL1-like |
| 36 | 68 | 62 | 129 | 157 | 92 | AT2G20500.1 | | |
| 2047 | 1765 | 1441 | 1027 | 1273 | 803 | AT3G04810 | ATNEK2, NE | NIMA-rela |
| 195 | 215 | 144 | 71 | 98 | 33 | AT3G51895 | AST12, SUL | sulfate t |
| 1725 | 1472 | 1697 | 1357 | 1639 | 602 | AT3G22600.1 | | Bifunctio |
| 158 | 131 | 130 | 89 | 108 | 64 | AT2G27310.1 | | F-box fam |
| 73 | 61 | 70 | 112 | 135 | 83 | AT1G03600 | PSB27 | photosyst |
| 177 | 234 | 216 | 299 | 434 | 307 | AT1G43710 | emb1075 | Pyridoxal |
| 108 | 89 | 146 | 74 | 115 | 52 | AT1G34670 | AtMYB93, M | myb domai |
| 628 | 534 | 463 | 408 | 513 | 375 | AT1G72220.1 | | RING/U-bo |
| 1275 | 1263 | 1004 | 965 | 1261 | 999 | AT2G22540 | AGL22, SVP | K-box reg |
| 2893 | 2954 | 3788 | 1226 | 1699 | 1485 | AT4G24220 | AWI31, VEP | NAD(P)-bi |
| 133 | 132 | 112 | 87 | 106 | 60 | AT1G46768 | RAP2.1 | related t |
| 27 | 37 | 36 | 60 | 79 | 63 | AT1G47490 | ATRBP47C, | RNA-bindi |
| 68 | 67 | 56 | 22 | 25 | 16 | AT1G02040.1 | | C2H2-type |
| 136 | 127 | 91 | 85 | 92 | 71 | AT4G00230 | XSP1 | xylem ser |
| 603 | 669 | 566 | 823 | 1238 | 1196 | AT4G00570 | NAD-ME2 | NAD-depen |
| 49 | 20 | 54 | 24 | 21 | 11 | AT2G46130 | ATWRKY43, | WRKY DNA- |

| | | | | | | | | |
|---|---|---|---|---|---|---|---|---|
| 91 | 106 | 88 | 69 | 44 | 45 | AT2G46770 | ANAC043, E | NAC (No A |
| 193 | 247 | 170 | 172 | 204 | 136 | AT2G34430 | LHB1B1, LH | light-har |
| 228 | 265 | 182 | 93 | 116 | 104 | AT1G03010.1 | | Phototrop |
| 354 | 327 | 323 | 151 | 247 | 263 | AT1G12900 | GAPA-2 | glycerald |
| 1157 | 1207 | 1094 | 997 | 1343 | 853 | AT3G24770 | CLE41 | CLAVATA3/ |
| 83 | 75 | 71 | 56 | 72 | 29 | AT3G25400.1 | | |
| 959 | 959 | 858 | 557 | 671 | 557 | AT4G28640 | IAA11 | indole-3- |
| 101 | 78 | 76 | 48 | 47 | 17 | AT4G37370 | CYP81D8 | cytochrom |
| 148 | 138 | 114 | 160 | 234 | 177 | AT3G18430.1 | | Calcium-b |
| 159 | 190 | 232 | 87 | 90 | 91 | AT3G21250 | ATMRP6, MR | multidrug |
| 21 | 18 | 12 | 37 | 44 | 72 | AT1G72970 | EDA17, HTH | Glucose-m |
| 152 | 142 | 135 | 80 | 117 | 101 | AT1G45688.1 | | |
| 11 | 18 | 12 | 136 | 71 | 123 | AT1G72610 | ATGER1, GE | germin-li |
| 4653 | 4483 | 5980 | 1291 | 2047 | 1783 | AT5G07990 | CYP75B1, D | Cytochrom |
| 684 | 672 | 687 | 268 | 367 | 319 | AT5G07990 | CYP75B1, D | Cytochrom |
| 447 | 433 | 399 | 544 | 615 | 545 | AT1G32470.1 | | Single hy |
| 108 | 80 | 88 | 144 | 149 | 220 | AT4G25960 | PGP2 | P-glycopr |
| 71 | 90 | 80 | 33 | 34 | 25 | AT4G39730.1 | | Lipase/li |
| 280 | 301 | 241 | 223 | 265 | 148 | AT4G12350 | AtMYB42, M | myb domai |
| 782 | 760 | 732 | 717 | 726 | 585 | AT4G20360 | ATRAB8D, A | RAB GTPas |
| 174 | 148 | 142 | 83 | 101 | 74 | AT5G23750.1 | | Remorin f |
| 255 | 296 | 248 | 185 | 225 | 160 | AT5G23860 | TUB8 | tubulin b |
| 271 | 313 | 236 | 461 | 482 | 557 | AT5G12250 | TUB6 | beta-6 tu |
| 358 | 372 | 300 | 256 | 256 | 282 | AT3G61490.3 | | Pectin ly |
| 479 | 496 | 359 | 334 | 371 | 262 | AT1G63910 | AtMYB103, | myb domai |
| 819 | 803 | 659 | 616 | 719 | 440 | AT5G41800.1 | | Transmemb |
| 31 | 38 | 42 | 13 | 14 | 13 | AT4G23990 | ATCSLG3, C | cellulose |
| 97 | 117 | 83 | 179 | 179 | 160 | AT4G23990 | ATCSLG3, C | cellulose |
| 31 | 33 | 24 | 8 | 7 | 14 | AT3G12720 | ATMYB67, A | myb domai |
| 440 | 550 | 487 | 580 | 766 | 651 | AT1G09690.1 | | Translati |
| 563 | 702 | 441 | 299 | 356 | 419 | AT5G13870 | EXGT-A4, X | xylogluca |
| 272 | 309 | 313 | 245 | 282 | 145 | AT2G33550.1 | | Homeodoma |
| 611 | 547 | 517 | 316 | 330 | 347 | AT5G13120 | ATCYP20-2 | cyclophil |
| 255 | 299 | 212 | 168 | 208 | 138 | AT5G14090.1 | | |
| 0 | 0 | 0 | 9 | 66 | 54 | AT1G24260 | AGL9, SEP3 | K-box reg |
| 105 | 97 | 82 | 85 | 82 | 51 | AT5G43260.1 | | chaperone |
| 312 | 361 | 320 | 290 | 263 | 215 | AT2G23520.1 | | Pyridoxal |
| 1293 | 1221 | 921 | 676 | 841 | 624 | AT1G27920 | MAP65-8 | microtubu |
| 154 | 173 | 127 | 111 | 141 | 108 | AT1G04770.1 | | Tetratric |
| 12 | 3 | 10 | 36 | 43 | 55 | AT1G64660 | ATMGL, MGL | methionin |
| 5 | 2 | 9 | 24 | 42 | 36 | AT1G64660 | ATMGL, MGL | methionin |
| 647 | 491 | 428 | 399 | 510 | 275 | AT3G21240 | 4CL2, AT4C | 4-coumara |
| 165 | 158 | 147 | 97 | 141 | 24 | AT2G03220 | ATFT1, ATF | fucosyltr |
| 344 | 339 | 309 | 310 | 336 | 246 | AT5G07020.1 | | proline-r |
| 316 | 318 | 260 | 607 | 983 | 710 | AT5G12330 | LRP1 | Lateral r |
| 12 | 7 | 4 | 21 | 57 | 47 | AT3G20820.1 | | Leucine-r |
| 939 | 804 | 770 | 554 | 770 | 256 | AT3G57880.1 | | Calcium-d |
| 255 | 274 | 242 | 208 | 233 | 189 | AT2G05990 | ENR1, MOD1 | NAD(P)-bi |
| 251 | 241 | 193 | 167 | 178 | 86 | AT3G45240 | ATSNAK2, G | geminivir |
| 21 | 29 | 22 | 3 | 6 | 4 | AT5G17230 | PSY | PHYTOENE |
| 4390 | 4119 | 3840 | 3332 | 3585 | 2888 | AT3G13470.1 | | TCP-1/cpn |
| 166 | 173 | 143 | 123 | 106 | 112 | AT4G21990 | APR3, ATAP | APS reduc |
| 10 | 37 | 12 | 46 | 52 | 40 | AT1G70880.1 | | Polyketid |
| 30 | 31 | 22 | 67 | 53 | 81 | AT1G11545 | XTH8 | xylogluca |

| | | | | | | | | |
|---|---|---|---|---|---|---|---|---|
| 2232 | 2451 | 2001 | 2822 | 3280 | 2796 | AT4G05180 | PSBQ,PSBQ | photosyst |
| 455 | 493 | 424 | 1318 | 1753 | 1140 | AT4G05150.1 | | Octicosap |
| 38 | 42 | 32 | 136 | 237 | 118 | AT4G27450.1 | | Aluminium |
| 89 | 83 | 64 | 123 | 163 | 113 | AT1G28360 | ATERF12,E | ERF domai |
| 35 | 13 | 14 | 58 | 63 | 74 | | | |
| 1869 | 1435 | 1459 | 1121 | 1283 | 345 | AT1G28680.1 | | HXXXD-typ |
| 83 | 87 | 96 | 76 | 62 | 14 | AT1G06330.1 | | Heavy met |
| 9312 | 8762 | 6707 | 6092 | 7224 | 5192 | AT4G18780 | ATCESA8,C | cellulose |
| 18 | 8 | 14 | 38 | 43 | 48 | AT1G29670.1 | | GDSL-like |
| 7 | 4 | 2 | 16 | 18 | 20 | AT1G17860.1 | | Kunitz fa |
| 99 | 97 | 73 | 55 | 53 | 6 | AT3G16520 | UGT88A1 | UDP-gluco |
| 939 | 902 | 1105 | 313 | 463 | 386 | AT1G65880 | BZO1 | benzoylox |
| 547 | 555 | 430 | 886 | 1435 | 1175 | AT5G37600 | ATGLN1;1, | glutamine |
| 538 | 531 | 548 | 257 | 301 | 97 | AT5G37690.1 | | SGNH hydr |
| 22 | 39 | 23 | 114 | 77 | 67 | AT1G66400 | CML23 | calmoduli |
| 156 | 167 | 158 | 620 | 726 | 447 | AT2G01210.1 | | Leucine-r |
| 81 | 101 | 86 | 353 | 453 | 261 | AT3G18280.1 | | Bifunctio |
| 26 | 38 | 30 | 105 | 86 | 95 | AT5G16030.1 | | |
| 256 | 234 | 203 | 131 | 176 | 52 | AT5G15900 | TBL19 | TRICHOME |
| 4 | 1 | 3 | 2 | 15 | 25 | AT1G69530 | AT-EXP1,A | expansin |
| 50 | 69 | 61 | 0 | 2 | 2 | AT3G28917 | MIF2 | mini zinc |
| 230 | 133 | 126 | 43 | 68 | 7 | AT5G08640 | ATFLS1,FL | flavonol |
| 32 | 19 | 38 | 154 | 199 | 178 | AT5G42650 | AOS,CYP74 | allene ox |
| 113 | 136 | 97 | 187 | 222 | 271 | AT4G34530 | CIB1 | cryptochr |
| 2 | 0 | 0 | 1 | 7 | 11 | | | |
| 396 | 448 | 380 | 377 | 334 | 281 | AT1G75390 | AtbZIP44, | basic leu |
| 3484 | 3064 | 2885 | 2499 | 2769 | 2365 | AT4G38970 | FBA2 | fructose- |
| 60 | 28 | 16 | 54 | 81 | 108 | AT4G38770 | ATPRP4,PR | proline-r |
| 53 | 40 | 47 | 21 | 32 | 17 | AT2G24960.2 | | |
| 985 | 1180 | 1016 | 535 | 789 | 633 | AT2G16700 | ADF5,ATAD | actin dep |
| 1316 | 1214 | 1146 | 763 | 1007 | 287 | AT4G33790 | CER4,FAR3 | Jojoba ac |
| 588 | 673 | 658 | 929 | 965 | 854 | AT1G08200 | AXS2 | UDP-D-api |
| 330 | 371 | 467 | 235 | 360 | 240 | AT2G36830 | GAMMA-TIP | gamma ton |
| 138 | 160 | 136 | 70 | 68 | 42 | AT1G09610.1 | | Protein o |
| 459 | 385 | 438 | 177 | 361 | 124 | AT1G59740.1 | | Major fac |
| 777 | 956 | 743 | 1580 | 1976 | 1509 | AT4G19420.1 | | Pectinace |
| 1761 | 1348 | 1314 | 689 | 795 | 128 | AT4G19380.1 | | Long-chai |
| 36 | 30 | 22 | 128 | 212 | 247 | AT1G09240 | ATNAS3,NA | nicotiana |
| 661 | 592 | 565 | 525 | 626 | 477 | AT5G64740 | CESA6,E11 | cellulose |
| 25 | 24 | 18 | 5 | 15 | 0 | AT3G52970 | CYP76G1 | cytochrom |
| 64 | 66 | 70 | 108 | 132 | 89 | AT5G27550.1 | | P-loop co |
| 102 | 114 | 103 | 47 | 46 | 20 | AT1G08990 | PGSIP5 | plant gly |
| 951 | 881 | 874 | 782 | 970 | 691 | AT3G04880 | DRT102 | DNA-damag |
| 7 | 7 | 2 | 15 | 14 | 16 | AT5G48920 | TED7 | tracheary |
| 1452 | 1537 | 1239 | 541 | 507 | 348 | AT3G18660 | GUX1,PGSI | plant gly |
| 262 | 244 | 232 | 145 | 172 | 44 | AT1G34670 | AtMYB93,M | myb domai |
| 813 | 661 | 723 | 482 | 492 | 134 | AT5G23190 | CYP86B1 | cytochrom |
| 172 | 137 | 101 | 72 | 97 | 23 | AT1G65730 | YSL7 | YELLOW ST |
| 188 | 184 | 192 | 153 | 178 | 109 | AT1G56170 | ATHAP5B,H | nuclear f |
| 328 | 407 | 319 | 767 | 818 | 517 | AT4G35550 | ATWOX13,H | WUSCHEL r |
| 11 | 9 | 13 | 16 | 41 | 44 | AT2G16850 | PIP2;8,PI | plasma me |
| 4704 | 4338 | 3639 | 2129 | 2164 | 1657 | AT4G36220 | CYP84A1,F | ferulic a |
| 109 | 130 | 96 | 148 | 202 | 158 | AT3G50410 | OBP1 | OBF bindi |
| 3271 | 3114 | 2568 | 2097 | 2408 | 1896 | AT5G67210.1 | | Protein o |

| | | | | | | | |
|---|---|---|---|---|---|---|---|
| 20 | 30 | 21 | 45 | 46 | 35 | AT1G72200.1 | RING/U-bo |
| 576 | 507 | 609 | 315 | 356 | 85 | AT1G34670AtMYB93,M | myb domai |
| 425 | 406 | 382 | 317 | 385 | 274 | AT5G65170.1 | VQ motif- |
| 29 | 51 | 20 | 89 | 119 | 155 | AT1G22150SULTR1;3 | sulfate t |
| 39 | 48 | 31 | 71 | 143 | 173 | AT1G22150SULTR1;3 | sulfate t |
| 64 | 102 | 73 | 118 | 223 | 141 | AT4G08300.1 | nodulin M |
| 204 | 199 | 174 | 320 | 505 | 478 | AT1G77000ATSKP2;2, | RNI-like |
| 94 | 72 | 35 | 33 | 39 | 13 | AT1G17950ATMYB52,B | myb domai |
| 886 | 870 | 963 | 511 | 692 | 428 | AT1G71695.1 | Peroxidas |
| 55 | 50 | 43 | 330 | 543 | 442 | AT5G09970CYP78A7 | cytochrom |
| 80 | 93 | 96 | 184 | 181 | 178 | AT1G03600PSB27 | photosyst |
| 630 | 672 | 560 | 551 | 531 | 492 | AT1G06690.1 | NAD(P)-li |
| 85 | 63 | 83 | 65 | 61 | 22 | AT2G48130.1 | Bifunctio |
| 81 | 78 | 85 | 149 | 162 | 99 | AT4G28530anac074,N | NAC domai |
| 291 | 283 | 261 | 148 | 204 | 148 | AT1G27680APL2 | ADPGLC-PP |
| 133 | 140 | 125 | 78 | 107 | 54 | AT1G27620.1 | HXXXD-typ |
| 125 | 116 | 108 | 174 | 237 | 144 | AT1G19715.3 | Mannose-b |
| 491 | 493 | 451 | 392 | 357 | 421 | AT4G36360BGAL3 | beta-gala |
| 556 | 560 | 533 | 817 | 1088 | 660 | AT1G19850ARF5,IAA2 | Transcrip |
| 388 | 448 | 389 | 320 | 416 | 358 | AT1G47840HXK3 | hexokinas |
| 2352 | 2459 | 2245 | 2979 | 3747 | 3030 | AT1G76100PETE1 | plastocya |
| 170 | 167 | 164 | 98 | 85 | 116 | AT4G35750.1 | SEC14 cyt |
| 527 | 557 | 556 | 2329 | 3379 | 2103 | AT1G42430.1 | |
| 2495 | 2438 | 2142 | 1908 | 2233 | 1892 | AT1G42970GAPB | glycerald |
| 88 | 76 | 74 | 114 | 177 | 107 | AT1G21000.2 | PLATZ tra |
| 130 | 153 | 117 | 504 | 732 | 529 | AT4G16480ATINT4,IN | inositol |
| 90 | 138 | 107 | 553 | 985 | 555 | AT4G16480ATINT4,IN | inositol |
| 96 | 81 | 81 | 102 | 168 | 137 | AT4G16410.1 | |
| 4 | 5 | 1 | 24 | 33 | 10 | AT1G22400ATUGT85A1 | UDP-Glyco |
| 134 | 141 | 114 | 75 | 81 | 69 | AT5G04200AtMC9,MC9 | metacaspa |
| 535 | 485 | 362 | 244 | 260 | 141 | AT1G66120.1 | AMP-depen |
| 183 | 181 | 156 | 265 | 372 | 261 | AT3G08550ABI8,ELD1 | elongatio |
| 1266 | 1444 | 1319 | 1767 | 1996 | 1655 | AT3G57490.1 | Ribosomal |
| 111 | 137 | 110 | 342 | 480 | 287 | AT3G53720ATCHX20,C | cation/H+ |
| 76 | 91 | 96 | 561 | 567 | 739 | AT2G10940.1 | Bifunctio |
| 313 | 310 | 293 | 249 | 283 | 222 | AT2G43290MSS3 | Calcium-b |
| 2510 | 1775 | 1952 | 1701 | 1166 | 1416 | AT2G26660ATSPX2,SP | SPX domai |
| 3 | 4 | 3 | 29 | 59 | 75 | AT2G37460.1 | nodulin M |
| 17 | 25 | 15 | 38 | 37 | 37 | AT5G02540.1 | NAD(P)-bi |
| 381 | 417 | 397 | 589 | 808 | 735 | AT2G37640ATEXP3,AT | Barwin-li |
| 1459 | 1343 | 1054 | 516 | 501 | 386 | AT5G60020ATLAC17,L | laccase 1 |
| 498 | 468 | 492 | 310 | 379 | 78 | AT2G29150.1 | NAD(P)-bi |
| 172 | 150 | 124 | 90 | 118 | 71 | AT5G02090.1 | |
| 111 | 140 | 145 | 91 | 92 | 108 | AT2G38080ATLMCO4,I | Laccase/D |
| 1140 | 999 | 1020 | 581 | 769 | 178 | AT2G38110ATGPAT6,G | glycerol- |
| 116 | 79 | 106 | 12 | 18 | 17 | AT4G00430PIP1;4,PI | plasma me |
| 143 | 115 | 153 | 213 | 232 | 344 | AT3G51860ATCAX3,AT | cation ex |
| 668 | 672 | 514 | 537 | 569 | 502 | AT5G05580FAD8 | fatty aci |
| 4 | 0 | 0 | 6 | 5 | 10 | AT1G07080.1 | Thioredox |
| 720 | 777 | 509 | 409 | 564 | 510 | AT5G01930.1 | Glycosyl |
| 34 | 25 | 35 | 59 | 71 | 56 | AT1G14360ATUTR3,UT | UDP-galac |
| 109 | 138 | 128 | 170 | 207 | 198 | AT3G54210.1 | Ribosomal |
| 2967 | 3168 | 2708 | 2353 | 2697 | 2411 | AT2G36530ENO2,LOS2 | Enolase |
| 5 | 1 | 2 | 34 | 29 | 29 | AT5G03795.1 | Exostosin |

| | | | | | | | | |
|---|---|---|---|---|---|---|---|---|
| 641 | 729 | 656 | 941 | 1016 | 986 | AT2G36870 | XTH32 | xylogluca |
| 44 | 46 | 47 | 79 | 100 | 68 | AT2G37170 | PIP2;2,PI | plasma me |
| 1604 | 1874 | 1522 | 1410 | 1423 | 1265 | AT5G03170 | ATFLA11,F | FASCICLIN |
| 249 | 258 | 214 | 64 | 90 | 33 | AT2G39200 | ATMLO12,M | Seven tra |
| 479 | 386 | 349 | 1410 | 1716 | 986 | AT2G37130.1 | | Peroxidas |
| 744 | 681 | 589 | 533 | 653 | 551 | AT5G02790 | GSTL3 | Glutathio |
| 455 | 409 | 413 | 369 | 389 | 342 | AT3G26060 | ATPRX Q | Thioredox |
| 7 | 6 | 3 | 201 | 107 | 152 | AT5G20630 | ATGER3,GE | germin 3 |
| 166 | 130 | 133 | 103 | 119 | 44 | AT3G21240 | 4CL2,AT4C | 4-coumara |
| 5237 | 5191 | 3983 | 3705 | 4150 | 3147 | AT5G17420 | ATCESA7,C | Cellulose |
| 284 | 176 | 184 | 328 | 402 | 600 | AT2G26250 | FDH,KCS10 | 3-ketoacy |
| 165 | 199 | 288 | 52 | 55 | 52 | AT5G35550 | ATMYB123, | Duplicate |
| 557 | 564 | 584 | 374 | 494 | 257 | AT4G38620 | ATMYB4,MY | myb domai |
| 1497 | 1615 | 1439 | 980 | 977 | 693 | AT5G11890.1 | | |
| 1512 | 1517 | 1541 | 923 | 1238 | 911 | AT4G32870.1 | | Polyketid |
| 109 | 106 | 75 | 53 | 46 | 24 | AT5G25610 | ATRD22,RD | BURP doma |
| 1529 | 1211 | 1137 | 542 | 726 | 219 | AT5G25610 | ATRD22,RD | BURP doma |
| 206 | 131 | 140 | 221 | 642 | 656 | AT5G11420.1 | | Protein o |
| 275 | 345 | 254 | 165 | 227 | 132 | AT4G32280 | IAA29 | indole-3- |
| 464 | 393 | 355 | 251 | 316 | 44 | AT2G24430 | ANAC038,A | NAC domai |
| 229 | 200 | 158 | 127 | 184 | 113 | AT4G31290.1 | | ChaC-like |
| 590 | 735 | 681 | 1098 | 1138 | 932 | AT5G23400.1 | | Leucine-r |
| 736 | 577 | 663 | 354 | 415 | 99 | AT1G32910.1 | | HXXXD-typ |
| 261 | 245 | 242 | 331 | 516 | 288 | AT4G37790 | HAT22 | Homeobox- |
| 527 | 492 | 409 | 689 | 980 | 870 | AT4G37870 | PCK1,PEPC | phosphoen |
| 151 | 118 | 119 | 85 | 95 | 31 | AT5G65500.1 | | U-box dom |
| 186 | 192 | 157 | 130 | 169 | 98 | AT2G18060 | ANAC037,V | vascular |
| 5176 | 4723 | 3997 | 2987 | 3487 | 2431 | AT4G36220 | CYP84A1,F | ferulic a |
| 480 | 413 | 464 | 273 | 345 | 131 | AT2G18360.1 | | alpha/bet |
| 117 | 81 | 78 | 59 | 72 | 66 | AT3G50685.1 | | |
| 574 | 547 | 466 | 479 | 576 | 361 | AT2G23760 | BLH4,SAW2 | BEL1-like |
| 1211 | 985 | 1140 | 827 | 914 | 286 | AT2G23540.1 | | GDSL-like |
| 25 | 23 | 21 | 7 | 9 | 5 | AT5G66870 | ASL1,LBD3 | ASYMMETRI |
| 26 | 34 | 22 | 10 | 8 | 12 | AT2G26560 | PLA IIA,P | phospholi |
| 344 | 345 | 303 | 243 | 352 | 216 | AT5G67020.1 | | |
| 498 | 548 | 490 | 308 | 323 | 272 | AT5G67210.1 | | Protein o |
| 1271 | 1000 | 1252 | 675 | 702 | 162 | AT5G23190 | CYP86B1 | cytochrom |
| 40 | 46 | 26 | 112 | 177 | 94 | AT5G08350.1 | | GRAM doma |
| 488 | 418 | 376 | 307 | 333 | 111 | AT5G10280 | ATMYB64,A | myb domai |
| 976 | 870 | 720 | 424 | 428 | 109 | AT5G64440 | AtFAAH,FA | fatty aci |
| 6324 | 7052 | 5578 | 9266 | 12163 | 11185 | AT5G09220 | AAP2 | amino aci |
| 157 | 125 | 112 | 80 | 98 | 36 | AT4G33450 | ATMYB69,M | myb domai |
| 1969 | 1961 | 1446 | 452 | 408 | 321 | AT3G18660 | GUX1,PGSI | plant gly |
| 0 | 0 | 0 | 3 | 3 | 10 | AT5G48920 | TED7 | tracheary |
| 380 | 482 | 371 | 333 | 378 | 312 | AT1G58120.1 | | |
| 230 | 218 | 295 | 155 | 225 | 62 | AT5G09520.1 | | hydroxypr |
| 259 | 229 | 220 | 122 | 152 | 100 | AT4G33580 | ATBCA5,BC | beta carb |
| 363 | 347 | 317 | 208 | 260 | 217 | AT4G28500 | ANAC073,N | NAC domai |
| 137 | 142 | 97 | 233 | 272 | 165 | AT4G28380.1 | | Leucine-r |
| 1855 | 1926 | 1434 | 766 | 641 | 540 | AT5G03760 | ATCSLA09, | Nucleotid |
| 6352 | 5771 | 4811 | 4635 | 5658 | 3487 | AT2G37040 | ATPAL1,PA | PHE ammon |
| 4 | 4 | 9 | 93 | 66 | 62 | AT3G54770.1 | | RNA-bindi |
| 580 | 468 | 397 | 253 | 285 | 65 | AT3G55090.1 | | ABC-2 typ |
| 1188 | 1186 | 996 | 1516 | 1675 | 1439 | AT5G02500 | AT-HSC70- | heat shoc |

| | | | | | | | | |
|---|---|---|---|---|---|---|---|---|
| 211 | 219 | 183 | 129 | 137 | 91 | AT3G55420.1 | | |
| 433 | 338 | 341 | 229 | 292 | 57 | AT5G06090 | ATGPAT7,G | glycerol- |
| 1855 | 1822 | 1598 | 2262 | 2567 | 2190 | AT2G39730 | RCA | rubisco a |
| 28 | 41 | 18 | 49 | 68 | 46 | AT2G44920.2 | | Tetratric |
| 226 | 200 | 181 | 370 | 426 | 226 | AT5G02070.1 | | Protein k |
| 628 | 676 | 520 | 402 | 483 | 430 | AT2G39770 | CYT1,EMB1 | Glucose-1 |
| 1998 | 1655 | 1886 | 2307 | 3757 | 2083 | AT5G05960.1 | | Bifunctio |
| 993 | 864 | 815 | 758 | 806 | 715 | AT3G55800 | SBPASE | sedoheptu |
| 1704 | 1897 | 1503 | 1162 | 1359 | 1076 | AT3G55990 | ESK1,TBL2 | Plant pro |
| 1564 | 1506 | 1346 | 1212 | 1291 | 848 | AT2G40320 | TBL33 | TRICHOME |
| 1248 | 1442 | 1149 | 1102 | 1196 | 776 | AT2G40370 | LAC5 | laccase 5 |
| 836 | 884 | 824 | 642 | 789 | 674 | AT5G05390 | LAC12 | laccase 1 |
| 39 | 30 | 25 | 47 | 87 | 117 | AT5G44680.1 | | DNA glyco |
| 114 | 117 | 85 | 66 | 81 | 19 | AT3G12720 | ATMYB67,A | myb domai |
| 439 | 511 | 424 | 636 | 813 | 557 | AT3G28960.1 | | Transmemb |
| 200 | 205 | 155 | 133 | 132 | 143 | AT1G26820 | RNS3 | ribonucle |
| 100 | 133 | 102 | 64 | 62 | 58 | AT3G15580 | APG8H,ATG | Ubiquitin |
| 317 | 235 | 253 | 34 | 45 | 3 | | | |
| 12 | 16 | 9 | 30 | 34 | 34 | AT5G49360 | ATBXL1,BX | beta-xylo |
| 165 | 144 | 171 | 80 | 77 | 20 | AT5G49350.1 | | Glycine-r |
| 15 | 14 | 16 | 26 | 30 | 32 | AT1G23000.1 | | Heavy met |
| 10 | 5 | 26 | 122 | 286 | 367 | AT1G14930.1 | | Polyketid |
| 220 | 286 | 277 | 514 | 445 | 362 | AT1G14890.1 | | Plant inv |
| 230 | 240 | 228 | 304 | 359 | 295 | AT1G14810.1 | | semialdeh |
| 1311 | 1355 | 1146 | 2198 | 2236 | 1937 | AT1G14720 | ATXTH28,E | xylogluca |
| 447 | 297 | 374 | 224 | 281 | 275 | AT2G37870.1 | | Bifunctio |
| 520 | 581 | 646 | 286 | 372 | 256 | AT2G32150.1 | | Haloacid |
| 34 | 28 | 43 | 18 | 10 | 5 | AT3G22840 | ELIP,ELIP | Chlorophy |
| 374 | 406 | 360 | 277 | 306 | 277 | AT3G22890 | APS1 | ATP sulfu |
| 1182 | 1051 | 1047 | 855 | 1097 | 304 | AT2G26640 | KCS11 | 3-ketoacy |
| 101 | 90 | 126 | 40 | 81 | 34 | AT4G14465 | AHL20 | AT-hook m |
| 346 | 340 | 223 | 148 | 262 | 192 | AT5G07050.1 | | nodulin M |
| 344 | 277 | 304 | 648 | 632 | 549 | AT3G23510.1 | | Cycloprop |
| 434 | 448 | 369 | 206 | 214 | 146 | AT2G33385 | arpc2b | actin-rel |
| 71 | 77 | 74 | 136 | 110 | 113 | AT4G17740.1 | | Peptidase |
| 535 | 465 | 438 | 370 | 477 | 205 | AT3G21530.1 | | DNAse I-l |
| 454 | 462 | 455 | 308 | 444 | 284 | AT4G04830 | ATMSRB5,M | methionin |
| 1853 | 1882 | 1552 | 1551 | 1971 | 1261 | AT3G23590 | MED33A,RF | REF4-rela |
| 85 | 75 | 61 | 127 | 119 | 119 | AT5G19160 | TBL11 | TRICHOME |
| 103 | 102 | 102 | 152 | 181 | 139 | AT4G02510 | ATTOC159, | transloco |
| 206 | 246 | 188 | 287 | 371 | 273 | AT2G27920 | SCPL51 | serine ca |
| 523 | 612 | 507 | 308 | 381 | 229 | AT5G22930.1 | | Protein o |
| 193 | 164 | 194 | 113 | 131 | 34 | AT5G57620 | AtMYB36,M | myb domai |
| 675 | 628 | 516 | 409 | 465 | 234 | AT2G28250 | NCRK | Protein k |
| 3005 | 2957 | 2516 | 1716 | 1974 | 1455 | AT2G28315.1 | | Nucleotid |
| 161 | 80 | 87 | 126 | 268 | 274 | AT5G22430.1 | | Pollen Ol |
| 482 | 455 | 439 | 126 | 152 | 30 | AT2G18370.1 | | Bifunctio |
| 34 | 46 | 23 | 90 | 101 | 91 | AT3G46780 | PTAC16 | plastid t |
| 99 | 139 | 95 | 402 | 650 | 454 | AT5G59080.1 | | |
| 2415 | 1795 | 2111 | 1304 | 1630 | 459 | AT5G58860 | CYP86,CYP | cytochrom |
| 153 | 162 | 164 | 217 | 250 | 210 | AT3G63540.1 | | Mog1/PsbP |
| 34 | 38 | 25 | 11 | 13 | 8 | AT2G32560.1 | | F-box fam |
| 1876 | 1562 | 1431 | 558 | 576 | 77 | AT5G07990 | CYP75B1,D | Cytochrom |
| 406 | 294 | 376 | 258 | 265 | 83 | AT5G19410.1 | | ABC-2 typ |

| | | | | | | | | |
|---|---|---|---|---|---|---|---|---|
| 1989 | 1791 | 1671 | 1574 | 1707 | 1364 | AT5G12300.1 | | Calcium-d |
| 1327 | 1150 | 1216 | 710 | 763 | 221 | AT1G49960.1 | | Xanthine/ |
| 898 | 1102 | 750 | 651 | 840 | 595 | AT1G10200 | WLIM1 | GATA type |
| 195 | 209 | 210 | 144 | 189 | 174 | AT4G34215.1 | | Domain of |
| 11 | 15 | 13 | 58 | 77 | 108 | AT5G03260 | LAC11 | laccase 1 |
| 247 | 276 | 401 | 100 | 118 | 114 | AT1G19190.1 | | alpha/bet |
| 328 | 431 | 303 | 519 | 658 | 413 | AT2G15790 | CYP40, SQN | peptidyl- |
| 280 | 228 | 212 | 127 | 144 | 130 | AT3G19000.1 | | 2-oxoglut |
| 520 | 443 | 396 | 364 | 441 | 226 | AT3G18950.1 | | Transduci |
| 103 | 81 | 88 | 52 | 75 | 63 | AT4G39230.1 | | NmrA-like |
| 3836 | 4027 | 3702 | 1030 | 1034 | 820 | AT4G39210 | APL3 | Glucose-1 |
| 195 | 178 | 214 | 130 | 134 | 67 | AT2G16385.1 | | |
| 2896 | 2737 | 2503 | 1837 | 2236 | 1757 | AT4G38970 | FBA2 | fructose- |
| 153 | 93 | 90 | 152 | 261 | 414 | AT3G61430 | ATPIP1, PI | plasma me |
| 1970 | 1820 | 1864 | 1091 | 1581 | 852 | AT4G38560.2 | | Arabidops |
| 94 | 98 | 94 | 87 | 79 | 58 | AT1G20550.1 | | O-fucosyl |
| 960 | 1006 | 995 | 1917 | 2225 | 1764 | AT5G22740 | ATCSLA02, | cellulose |
| 857 | 702 | 796 | 579 | 627 | 142 | AT5G22810.1 | | GDSL-like |
| 195 | 209 | 181 | 89 | 124 | 85 | AT2G29130 | ATLAC2, LA | laccase 2 |
| 23 | 26 | 32 | 48 | 58 | 77 | AT3G52500.1 | | Eukaryoti |
| 216 | 232 | 171 | 137 | 163 | 159 | AT2G36250 | ATFTSZ2-1 | Tubulin/F |
| 300 | 320 | 259 | 232 | 313 | 132 | AT1G08320 | bZIP21, TG | bZIP tran |
| 531 | 619 | 535 | 1148 | 1216 | 879 | AT3G17510 | CIPK1, SnR | CBL-inter |
| 281 | 345 | 249 | 372 | 474 | 355 | AT5G48960.1 | | HAD-super |
| 530 | 619 | 563 | 1001 | 1002 | 808 | AT5G26670.1 | | Pectinace |
| 57 | 83 | 66 | 127 | 181 | 105 | AT4G21380 | ARK3, RK3 | receptor |
| 15 | 18 | 18 | 24 | 43 | 31 | | | |
| 172 | 169 | 163 | 139 | 172 | 103 | AT3G17210 | ATHS1, HS1 | heat stab |
| 18 | 20 | 23 | 61 | 91 | 96 | AT3G01680.1 | | |
| 322 | 250 | 287 | 180 | 214 | 41 | AT1G24430.1 | | HXXXD-typ |
| 1438 | 1656 | 1375 | 1081 | 1281 | 1063 | AT2G33385 | arpc2b | actin-rel |
| 39 | 18 | 15 | 43 | 43 | 74 | AT2G33420.1 | | Protein o |
| 45 | 51 | 44 | 95 | 83 | 99 | AT5G46050 | ATPTR3, PT | peptide t |
| 235 | 241 | 216 | 171 | 195 | 98 | AT5G43420.1 | | RING/U-bo |
| 73 | 104 | 64 | 162 | 211 | 75 | AT1G06620.1 | | 2-oxoglut |
| 341 | 381 | 235 | 220 | 371 | 172 | AT4G24010 | ATCSLG1, C | cellulose |
| 413 | 323 | 374 | 243 | 276 | 121 | AT1G04220 | KCS2 | 3-ketoacy |
| 265 | 231 | 243 | 164 | 193 | 189 | AT3G18420.1 | | Protein p |
| 300 | 462 | 312 | 850 | 1004 | 440 | AT1G14780.1 | | MAC/Perfo |
| 26 | 21 | 27 | 53 | 52 | 44 | AT1G24620.1 | | EF hand c |
| 34 | 42 | 30 | 10 | 35 | 7 | AT2G01340 | At17.1 | |
| 580 | 512 | 562 | 369 | 369 | 95 | AT1G68850.1 | | Peroxidas |
| 1208 | 1119 | 1023 | 1020 | 1054 | 906 | AT2G04030 | AtHsp90.5 | Chaperone |
| 718 | 684 | 557 | 437 | 532 | 444 | AT1G13635.2 | | DNA glyco |
| 6 | 4 | 5 | 11 | 16 | 17 | AT4G37580 | COP3, HLS1 | Acyl-CoA |
| 188 | 246 | 192 | 441 | 532 | 272 | AT1G48100.1 | | Pectin ly |
| 531 | 531 | 377 | 357 | 439 | 323 | AT1G79420.1 | | Protein o |
| 163 | 117 | 128 | 67 | 84 | 20 | AT3G12720 | ATMYB67, A | myb domai |
| 78 | 78 | 61 | 107 | 139 | 166 | AT5G44680.1 | | DNA glyco |
| 17 | 12 | 23 | 65 | 76 | 56 | AT1G30820.1 | | CTP synth |
| 47 | 88 | 40 | 108 | 297 | 122 | AT2G40390.1 | | |
| 831 | 823 | 695 | 506 | 647 | 466 | AT5G05390 | LAC12 | laccase 1 |
| 1122 | 1187 | 956 | 876 | 1003 | 738 | AT2G40320 | TBL33 | TRICHOME |
| 429 | 330 | 354 | 200 | 212 | 168 | AT5G05690 | CBB3, CPD, | Cytochrom |

| | | | | | | | | |
|---|---|---|---|---|---|---|---|---|
| 595 | 668 | 479 | 340 | 446 | 408 | AT2G38120 | AUX1,MAP1 | Transmemb |
| 520 | 539 | 497 | 788 | 1010 | 776 | AT5G05790.1 | | Duplicate |
| 2921 | 2334 | 2944 | 1558 | 1989 | 1029 | AT5G05960.1 | | Bifunctio |
| 11 | 11 | 11 | 141 | 279 | 212 | AT2G39855.2 | | |
| 755 | 545 | 637 | 440 | 501 | 119 | AT3G11430 | ATGPAT5,G | glycerol- |
| 417 | 471 | 402 | 391 | 417 | 281 | AT5G54980.1 | | Uncharact |
| 96 | 92 | 79 | 50 | 63 | 26 | AT2G37430.1 | | C2H2 and |
| 731 | 594 | 629 | 427 | 484 | 103 | AT2G39350.1 | | ABC-2 typ |
| 802 | 637 | 561 | 352 | 341 | 162 | AT5G03760 | ATCSLA09, | Nucleotid |
| 85 | 80 | 65 | 60 | 71 | 41 | AT3G54240.1 | | alpha/bet |
| 893 | 720 | 748 | 645 | 680 | 662 | AT3G11760.1 | | |
| 47 | 38 | 33 | 25 | 25 | 3 | AT4G31540 | ATEXO70G1 | exocyst s |
| 344 | 382 | 297 | 289 | 308 | 235 | AT1G16860.1 | | Ubiquitin |
| 203 | 216 | 225 | 153 | 204 | 140 | AT3G21690.1 | | MATE effl |
| 42 | 36 | 30 | 121 | 163 | 118 | AT5G38280 | PR5K | PR5-like |
| 100 | 109 | 121 | 201 | 290 | 193 | AT3G54070.1 | | Ankyrin r |
| 328 | 327 | 286 | 455 | 613 | 315 | AT3G54070.1 | | Ankyrin r |
| 73 | 86 | 112 | 134 | 179 | 127 | AT3G54070.1 | | Ankyrin r |
| 5 | 18 | 12 | 27 | 63 | 37 | AT3G54070.1 | | Ankyrin r |
| 285 | 248 | 218 | 326 | 409 | 325 | AT4G05180 | PSBQ,PSBQ | photosyst |
| 173 | 155 | 144 | 249 | 308 | 184 | AT1G61660.1 | | basic hel |
| 199 | 155 | 228 | 81 | 93 | 52 | AT5G43060.1 | | Granulin |
| 180 | 168 | 175 | 84 | 112 | 93 | AT1G09850 | XBCP3 | xylem bar |
| 8 | 5 | 9 | 26 | 27 | 25 | AT1G29670.1 | | GDSL-like |
| 35 | 28 | 22 | 14 | 18 | 20 | AT1G29050 | TBL38 | TRICHOME |
| 135 | 109 | 109 | 77 | 123 | 28 | AT1G53270.1 | | ABC-2 typ |
| 60 | 71 | 82 | 24 | 37 | 37 | AT1G78380 | ATGSTU19, | glutathio |
| 134 | 197 | 164 | 249 | 330 | 254 | AT5G06740.1 | | Concanava |
| 533 | 560 | 478 | 694 | 925 | 674 | AT2G46770 | ANAC043,E | NAC (No A |
| 255 | 173 | 128 | 125 | 98 | 13 | AT5G44440.1 | | FAD-bindi |
| 126 | 120 | 80 | 79 | 55 | 79 | AT1G30700.1 | | FAD-bindi |
| 284 | 303 | 211 | 148 | 183 | 193 | AT1G23460.1 | | Pectin ly |
| 14774 | 14743 | 12079 | 9705 | 11117 | 8490 | AT5G54160 | ATOMT1,OM | O-methylt |
| 151 | 170 | 148 | 104 | 114 | 57 | AT3G24770 | CLE41 | CLAVATA3/ |
| 294 | 300 | 468 | 829 | 1407 | 1049 | AT1G74100 | ATSOT16,A | sulfotran |
| 287 | 284 | 289 | 153 | 225 | 172 | | | |
| 2 | 2 | 1 | 5 | 15 | 14 | AT1G73680 | ALPHA DOX | alpha dio |
| 223 | 183 | 204 | 118 | 147 | 30 | AT3G18400 | anac058,N | NAC domai |
| 1229 | 923 | 1144 | 756 | 846 | 259 | AT1G74460.1 | | GDSL-like |
| 49 | 52 | 27 | 83 | 111 | 55 | AT1G74710 | ATICS1,ED | ADC synth |
| 1526 | 1604 | 1474 | 1871 | 2710 | 1669 | AT3G01470 | ATHB-1,AT | homeobox |
| 778 | 848 | 813 | 734 | 850 | 662 | AT5G62790 | DXR,PDE12 | 1-deoxy-D |
| 228 | 253 | 196 | 149 | 212 | 175 | AT5G61640 | ATMSRA1,P | peptideme |
| 167 | 167 | 137 | 267 | 274 | 262 | AT5G63310 | ATNDPK2,N | nucleosid |
| 119 | 140 | 122 | 209 | 282 | 178 | AT3G48660.1 | | Protein o |
| 155 | 129 | 118 | 100 | 122 | 37 | AT1G24140.1 | | Matrixin |
| 266 | 289 | 253 | 501 | 643 | 438 | AT5G13980.1 | | Glycosyl |
| 246 | 303 | 273 | 366 | 409 | 403 | AT4G25080 | CHLM | magnesium |
| 363 | 333 | 342 | 171 | 188 | 146 | AT5G07330.1 | | |
| 8 | 7 | 8 | 18 | 26 | 36 | AT2G35640.1 | | Homeodoma |
| 517 | 690 | 481 | 234 | 285 | 220 | AT3G54950 | PLA IIIA, | patatin-l |
| 4 | 4 | 4 | 22 | 37 | 13 | AT4G25250.1 | | Plant inv |
| 1032 | 1215 | 1115 | 3795 | 4729 | 3776 | AT5G62350.1 | | Plant inv |
| 3830 | 3889 | 3499 | 2245 | 2563 | 1280 | AT5G23750.2 | | Remorin f |

| | | | | | | | | |
|---|---|---|---|---|---|---|---|---|
| 216 | 242 | 201 | 138 | 129 | 94 | AT4G25810 | XTH23, XTR | xylogluca |
| 203 | 165 | 169 | 124 | 133 | 155 | AT1G56580 | SVB | Protein o |
| 364 | 338 | 312 | 154 | 202 | 102 | | | |
| 253 | 296 | 234 | 134 | 206 | 147 | AT3G62630.1 | | Protein o |
| 89 | 70 | 41 | 28 | 22 | 27 | AT5G06900 | CYP93D1 | cytochrom |
| 954 | 1031 | 1174 | 1686 | 2589 | 1940 | | | |
| 426 | 482 | 404 | 1032 | 963 | 941 | AT5G56750 | NDL1 | N-MYC dow |
| 689 | 687 | 670 | 641 | 691 | 480 | AT3G04790.1 | | Ribose 5- |
| 721 | 497 | 624 | 405 | 262 | 308 | AT3G04530 | ATPPCK2, P | phosphoen |
| 40 | 41 | 31 | 65 | 66 | 68 | AT3G04760.1 | | Pentatric |
| 41 | 51 | 55 | 76 | 105 | 66 | AT3G03900 | APK3 | adenosine |
| 437 | 520 | 366 | 583 | 911 | 865 | AT5G18170 | GDH1 | glutamate |
| 1241 | 1295 | 1168 | 737 | 952 | 496 | AT5G56340 | ATCRT1 | RING/U-bo |
| 20 | 23 | 4 | 99 | 117 | 218 | AT2G03090 | ATEXP15, A | expansin |
| 35 | 26 | 23 | 15 | 27 | 7 | AT3G03700.1 | | Plasma-me |
| 1008 | 825 | 1101 | 365 | 407 | 186 | AT5G07990 | CYP75B1, D | Cytochrom |
| 761 | 751 | 945 | 216 | 346 | 293 | AT5G17540.1 | | HXXXD-typ |
| 22 | 20 | 75 | 52 | 324 | 146 | AT4G24350.1 | | Phosphory |
| 2308 | 2199 | 2480 | 330 | 451 | 559 | AT4G24340.1 | | Phosphory |
| 702 | 735 | 660 | 358 | 418 | 298 | AT1G33800.1 | | Protein o |
| 15 | 7 | 8 | 74 | 60 | 49 | AT1G75240 | AtHB33, HB | homeobox |
| 61 | 58 | 50 | 33 | 52 | 19 | AT1G50420 | SCL-3, SCL | scarecrow |
| 59 | 95 | 134 | 8 | 25 | 26 | AT4G35160.1 | | O-methylt |
| 502 | 561 | 513 | 357 | 465 | 364 | AT4G35020 | ARAC3, ATR | RAC-like |
| 3447 | 3838 | 3076 | 2329 | 2666 | 2092 | AT3G59690 | IQD13 | IQ-domain |
| 59 | 62 | 56 | 89 | 113 | 118 | AT1G21500.1 | | |
| 30 | 51 | 45 | 24 | 21 | 16 | AT4G20970.1 | | basic hel |
| 35 | 34 | 32 | 21 | 17 | 6 | AT4G03270 | CYCD6;1 | Cyclin D6 |
| 141 | 115 | 103 | 75 | 94 | 57 | AT2G40470 | ASL11, LBD | LOB domai |
| 80 | 58 | 57 | 101 | 144 | 125 | AT2G22840 | AtGRF1, GR | growth-re |
| 11 | 11 | 13 | 27 | 71 | 51 | AT3G49940 | LBD38 | LOB domai |
| 1675 | 1789 | 2256 | 586 | 808 | 641 | AT4G24220 | AWI31, VEP | NAD(P)-bi |
| 70 | 74 | 60 | 29 | 18 | 30 | AT5G59845.1 | | Gibberell |
| 342 | 275 | 289 | 157 | 194 | 113 | AT2G38185.1 | | RING/U-bo |
| 192 | 207 | 237 | 404 | 413 | 383 | AT4G37260 | ATMYB73, M | myb domai |
| 50 | 38 | 34 | 133 | 127 | 135 | AT1G60060.1 | | Serine/th |
| 28 | 37 | 33 | 75 | 124 | 98 | AT5G67090.1 | | Subtilisi |
| 174 | 161 | 123 | 641 | 795 | 472 | AT5G67090.1 | | Subtilisi |
| 22 | 27 | 19 | 48 | 48 | 30 | AT5G42380 | CML37, CML | calmoduli |
| 143 | 199 | 195 | 138 | 120 | 96 | AT3G50610.1 | | |
| 1153 | 1153 | 989 | 660 | 749 | 649 | AT1G19300 | ATGATL1, G | Nucleotid |
| 112 | 112 | 113 | 152 | 262 | 189 | AT5G42610.1 | | Protein o |
| 16 | 20 | 24 | 15 | 12 | 6 | AT4G01070 | GT72B1, UG | UDP-Glyco |
| 328 | 348 | 337 | 155 | 204 | 146 | AT1G75130 | CYP721A1 | cytochrom |
| 582 | 565 | 497 | 327 | 394 | 264 | AT4G00750.1 | | S-adenosy |
| 1058 | 792 | 874 | 641 | 886 | 205 | AT2G37360.1 | | ABC-2 typ |
| 189 | 171 | 145 | 79 | 111 | 54 | AT1G01780.1 | | GATA type |
| 222 | 172 | 192 | 90 | 142 | 44 | AT2G46130 | ATWRKY43, | WRKY DNA- |
| 238 | 183 | 230 | 138 | 174 | 46 | AT3G61680.1 | | alpha/bet |
| 30 | 35 | 30 | 17 | 26 | 18 | AT1G09530 | PAP3, PIF3 | phytochro |
| 90 | 98 | 51 | 147 | 141 | 107 | AT4G01800 | AGY1, Atcp | Albino or |
| 48 | 26 | 29 | 98 | 111 | 164 | AT5G19730.1 | | Pectin ly |
| 70 | 48 | 44 | 140 | 117 | 114 | AT4G13030.1 | | P-loop co |
| 20 | 18 | 18 | 88 | 150 | 143 | AT5G64260 | EXL2 | EXORDIUM |

| | | | | | | | | |
|---|---|---|---|---|---|---|---|---|
| 31 | 14 | 18 | 40 | 70 | 55 | AT4G02290 | AtGH9B13, | glycosyl |
| 1 | 0 | 0 | 24 | 68 | 53 | AT3G62950.1 | | Thioredox |
| 105 | 121 | 95 | 251 | 289 | 175 | AT3G63010 | ATGID1B, G | alpha/bet |
| 537 | 506 | 718 | 200 | 328 | 221 | AT2G48020.1 | | Major fac |
| 41 | 27 | 50 | 18 | 32 | 15 | AT2G48070 | RPH1 | resistanc |
| 54 | 75 | 49 | 38 | 46 | 20 | AT5G45710 | AT-HSFA4C | winged-he |
| 200 | 187 | 181 | 104 | 127 | 87 | AT2G42610 | LSH10 | Protein o |
| 47 | 50 | 45 | 91 | 98 | 90 | AT5G14060 | CARAB-AK- | Aspartate |
| 445 | 465 | 366 | 907 | 990 | 813 | AT3G23690.1 | | basic hel |
| 5 | 10 | 5 | 13 | 19 | 22 | AT3G59010 | PME61 | pectin me |
| 463 | 461 | 402 | 596 | 663 | 565 | AT1G05190 | emb2394 | Ribosomal |
| 1177 | 1271 | 1134 | 1068 | 1208 | 1034 | AT2G04280.1 | | |
| 358 | 328 | 350 | 201 | 224 | 59 | AT1G29000.1 | | Heavy met |
| 167 | 182 | 159 | 143 | 133 | 55 | AT2G16230.1 | | O-Glycosy |
| 11 | 9 | 4 | 17 | 38 | 21 | AT3G28920 | AtHB34, HB | homeobox |
| 107 | 117 | 94 | 51 | 76 | 52 | AT1G17950 | ATMYB52, B | myb domai |
| 122 | 76 | 109 | 70 | 75 | 14 | AT3G18170.1 | | Glycosylt |
| 109 | 82 | 90 | 64 | 59 | 12 | AT3G18400 | anac058, N | NAC domai |
| 388 | 371 | 370 | 290 | 348 | 240 | AT5G61340.1 | | |
| 19 | 18 | 20 | 44 | 69 | 17 | AT1G60190.1 | | ARM repea |
| 11 | 18 | 9 | 3 | 0 | 0 | AT5G14740 | BETA CA2, | carbonic |
| 659 | 712 | 620 | 549 | 714 | 564 | AT1G17100.1 | | SOUL heme |
| 547 | 519 | 546 | 295 | 357 | 98 | AT1G08510 | FATB | fatty acy |
| 197 | 198 | 196 | 312 | 326 | 322 | AT5G45160.1 | | Root hair |
| 157 | 142 | 149 | 112 | 132 | 42 | AT5G51460 | ATTPPA | Haloacid |
| 218 | 262 | 219 | 165 | 174 | 79 | AT5G62350.1 | | Plant inv |
| 68 | 91 | 71 | 125 | 103 | 162 | AT3G03620.1 | | MATE effl |
| 137 | 149 | 124 | 71 | 93 | 77 | AT5G47635.1 | | Pollen Ol |
| 587 | 481 | 438 | 352 | 291 | 237 | AT4G17220 | ATMAP70-5 | microtubu |
| 116 | 88 | 66 | 46 | 69 | 57 | AT5G04200 | AtMC9, MC9 | metacaspa |
| 1404 | 1443 | 1342 | 1045 | 1276 | 1040 | AT5G05110.1 | | Cystatin/ |
| 227 | 201 | 207 | 153 | 193 | 74 | AT2G05990 | ENR1, MOD1 | NAD(P)-bi |
| 125 | 117 | 97 | 74 | 74 | 45 | AT2G41690 | AT-HSFB3, | heat shoc |
| 142 | 104 | 133 | 287 | 330 | 310 | AT3G52500.1 | | Eukaryoti |
| 680 | 678 | 750 | 539 | 705 | 537 | AT5G06290 | 2-Cys Prx | 2-cystein |
| 244 | 245 | 199 | 299 | 373 | 308 | AT3G09600.1 | | Homeodoma |
| 1057 | 1122 | 1006 | 718 | 856 | 648 | AT2G37090 | IRX9 | Nucleotid |
| 427 | 395 | 340 | 661 | 1005 | 667 | AT2G37750.1 | | |
| 1418 | 1429 | 1622 | 941 | 1132 | 816 | AT3G53980.1 | | Bifunctio |
| 39 | 47 | 48 | 23 | 37 | 19 | AT3G09085.1 | | Protein o |
| 335 | 319 | 287 | 223 | 240 | 117 | AT2G38060 | PHT4;2 | phosphate |
| 3551 | 3622 | 3082 | 2470 | 3246 | 2322 | AT2G38120 | AUX1, MAP1 | Transmemb |
| 268 | 269 | 186 | 326 | 390 | 362 | AT5G01360 | TBL3 | Plant pro |
| 358 | 423 | 424 | 1079 | 1254 | 1099 | AT5G01750.2 | | Protein o |
| 1872 | 1797 | 1568 | 691 | 784 | 754 | AT5G01930.1 | | Glycosyl |
| 1739 | 1924 | 1693 | 1534 | 1625 | 1193 | AT2G44160 | MTHFR2 | methylene |
| 123 | 100 | 99 | 191 | 290 | 119 | AT3G59850.1 | | Pectin ly |
| 4 | 3 | 11 | 29 | 20 | 29 | AT2G43560.1 | | FKBP-like |
| 839 | 752 | 690 | 455 | 565 | 446 | AT5G10610 | CYP81K1 | cytochrom |
| 55 | 38 | 30 | 83 | 137 | 108 | AT3G45140 | ATLOX2, LC | lipoxygen |
| 193 | 147 | 123 | 102 | 105 | 19 | AT1G58120.1 | | |
| 60 | 85 | 78 | 180 | 253 | 121 | AT1G70840 | MLP31 | MLP-like |
| 857 | 967 | 848 | 2582 | 3276 | 1470 | AT1G17840 | ABCG11, AT | white-bro |
| 1005 | 803 | 812 | 601 | 732 | 432 | AT2G32800 | AP4.3A | protein k |

| | | | | | | | | |
|---|---|---|---|---|---|---|---|---|
| 179 | 187 | 158 | 226 | 328 | 296 | AT3G01480 | ATCYP38,C | cyclophil |
| 2 | 1 | 2 | 7 | 18 | 16 | AT3G47800.1 | | Galactose |
| 98 | 96 | 90 | 168 | 218 | 188 | AT1G74160.1 | | |
| 32 | 32 | 25 | 156 | 224 | 176 | AT1G25330.1 | | basic hel |
| 45 | 58 | 48 | 98 | 59 | 123 | AT3G47570.1 | | Leucine-r |
| 80 | 51 | 66 | 38 | 55 | 17 | AT5G37690.1 | | SGNH hydr |
| 184 | 143 | 137 | 280 | 439 | 451 | AT5G37600 | ATGLN1;1, | glutamine |
| 272 | 310 | 319 | 507 | 582 | 478 | AT1G24625 | ZFP7 | zinc fing |
| 114 | 110 | 95 | 85 | 78 | 70 | AT1G65730 | YSL7 | YELLOW ST |
| 20 | 26 | 32 | 61 | 89 | 81 | AT3G57560 | NAGK | N-acetyl- |
| 393 | 334 | 367 | 205 | 219 | 45 | AT2G24430 | ANAC038,A | NAC domai |
| 128 | 146 | 103 | 53 | 59 | 21 | AT2G29110 | ATGLR2.8, | glutamate |
| 0 | 0 | 1 | 0 | 4 | 34 | AT3G63520 | ATCCD1,AT | carotenoi |
| 2013 | 2040 | 2066 | 659 | 678 | 222 | AT4G32810 | ATCCD8,CC | carotenoi |
| 78 | 92 | 88 | 278 | 322 | 289 | AT5G25880 | ATNADP-ME | NADP-mali |
| 2598 | 2508 | 3385 | 904 | 1387 | 1133 | AT5G07990 | CYP75B1,D | Cytochrom |
| 1520 | 1600 | 1346 | 851 | 1027 | 737 | AT5G11890.1 | | |
| 184 | 189 | 155 | 70 | 138 | 53 | AT1G74960 | ATKAS2,FA | fatty aci |
| 216 | 185 | 214 | 97 | 69 | 78 | AT3G26060 | ATPRX Q | Thioredox |
| 78 | 97 | 72 | 66 | 62 | 47 | AT3G42725.1 | | Putative |
| 111 | 111 | 65 | 204 | 226 | 185 | AT4G29060 | emb2726 | elongatio |
| 361 | 448 | 398 | 255 | 383 | 304 | AT5G38420.1 | | Ribulose |
| 3876 | 4376 | 3328 | 2872 | 3339 | 2864 | AT5G17420 | ATCESA7,C | Cellulose |
| 54 | 44 | 61 | 29 | 38 | 38 | AT5G48930 | HCT | hydroxyci |
| 152 | 103 | 143 | 89 | 134 | 79 | AT4G30610 | BRS1,SCPL | alpha/bet |
| 62 | 45 | 30 | 15 | 23 | 10 | AT5G20260.1 | | Exostosin |
| 118 | 113 | 122 | 182 | 197 | 160 | AT1G02630.1 | | Nucleosid |
| 957 | 904 | 880 | 289 | 286 | 258 | AT1G80130.1 | | Tetratric |
| 450 | 367 | 377 | 834 | 1264 | 742 | AT4G10310 | ATHKT1,HK | high-affi |
| 79 | 98 | 99 | 54 | 62 | 67 | AT2G22590.1 | | UDP-Glyco |
| 1924 | 1939 | 1490 | 980 | 1180 | 860 | AT2G03200.1 | | Eukaryoti |
| 7 | 15 | 12 | 19 | 33 | 49 | AT3G04290 | ATLTL1,LT | Li-tolera |
| 12 | 7 | 8 | 14 | 21 | 28 | AT5G33370.1 | | GDSL-like |
| 778 | 497 | 550 | 290 | 366 | 111 | AT3G05950.1 | | RmlC-like |
| 85 | 71 | 80 | 48 | 42 | 10 | AT3G05950.1 | | RmlC-like |
| 54 | 47 | 49 | 32 | 43 | 8 | AT5G16770 | AtMYB9,MY | myb domai |
| 500 | 613 | 433 | 720 | 846 | 691 | AT5G17020 | ATCRM1,AT | exportin |
| 3312 | 4317 | 3362 | 8986 | 11230 | 7828 | AT1G71380 | ATCEL3,AT | cellulase |
| 12 | 11 | 16 | 20 | 43 | 39 | AT4G32480.1 | | Protein o |
| 2905 | 2993 | 2578 | 3696 | 4505 | 3271 | AT1G22530 | PATL2 | PATELLIN |
| 1723 | 1747 | 1357 | 3044 | 4251 | 3118 | AT1G22710 | ATSUC2,SU | sucrose-p |
| 28 | 14 | 24 | 8 | 20 | 3 | AT2G42360.1 | | RING/U-bo |
| 601 | 612 | 582 | 197 | 274 | 64 | AT3G09390 | ATMT-1,AT | metalloth |
| 383 | 373 | 309 | 182 | 282 | 79 | AT1G03700.1 | | Uncharact |
| 327 | 292 | 294 | 754 | 993 | 671 | AT4G03510 | ATRMA1,RM | RING memb |
| 477 | 561 | 453 | 1670 | 1608 | 1036 | AT1G05710.5 | | basic hel |
| 446 | 494 | 434 | 375 | 487 | 306 | AT3G59690 | IQD13 | IQ-domain |
| 243 | 169 | 213 | 98 | 96 | 30 | AT2G30210 | LAC3 | laccase 3 |
| 126 | 123 | 104 | 362 | 509 | 242 | AT2G25150.1 | | HXXXD-typ |
| 2286 | 2112 | 1853 | 3564 | 5065 | 2852 | AT5G58350 | WNK4,ZIK2 | with no l |
| 187 | 103 | 192 | 235 | 282 | 333 | AT5G25610 | ATRD22,RD | BURP doma |
| 16 | 12 | 12 | 40 | 43 | 60 | AT4G27290.1 | | S-locus l |
| 1345 | 1822 | 1783 | 844 | 1646 | 997 | AT2G26560 | PLA IIA,P | phospholi |
| 943 | 1214 | 881 | 501 | 562 | 517 | AT5G37478.1 | | TPX2 (tar |

| | | | | | | | | |
|---|---|---|---|---|---|---|---|---|
| 30 | 27 | 27 | 78 | 83 | 113 | AT5G10180 | AST68,SUL | slufate t |
| 30 | 34 | 33 | 62 | 72 | 50 | AT4G19840 | ATPP2-A1, | phloem pr |
| 75 | 70 | 64 | 111 | 121 | 84 | AT4G04640 | ATPC1 | ATPase, F |
| 97 | 91 | 106 | 45 | 44 | 5 | AT2G26560 | PLA IIA,P | phospholi |
| 484 | 447 | 361 | 388 | 381 | 344 | AT4G21990 | APR3,ATAP | APS reduc |
| 239 | 185 | 224 | 129 | 157 | 49 | AT2G05990 | ENR1,MOD1 | NAD(P)-bi |
| 446 | 419 | 377 | 319 | 319 | 94 | AT1G24430.1 | | HXXXD-typ |
| 35 | 40 | 27 | 102 | 167 | 169 | AT3G01680.1 | | |
| 35 | 38 | 44 | 22 | 30 | 26 | AT1G79620.1 | | Leucine-r |
| 13 | 3 | 8 | 4 | 2 | 0 | AT3G13610.1 | | 2-oxoglut |

:line.y
:osidase 16
ıase 2
ıase 2
:ich repeat (LRR) family protein
ɔited Ca(2+)-ATPase, isoform 4
:esistance-responsive (dirigent-like protein) family protein
:al ion transporter 6
:e intrinsic protein 6;1
:opeptide repeat (TPR)-like superfamily protein
'ase-like superfamily protein
.7
ıle-associated protein 65-8
ıin-like superfamily protein
.ng (RRM/RBD/RNP motifs) family protein
:a-Hydrolases superfamily protein
ɔf unknown function, DUF538
hydrolase 9C2
ɔf unknown function (DUF604)
:amily protein
RNA polymerase D1A
.ike homeobox of Arabidopsis thaliana 7
: A
.n protein 42
ıolar processing enzyme
:opeptide repeat (TPR)-like superfamily protein

.ike 1
:thylesterase 3
:thylesterase 3
ɔha-trypsin inhibitor heavy chain-related
ıe P450 superfamily protein
:ransporter 3;4
ɔhotransmitter 4
:arate (2OG) and Fe(II)-dependent oxygenase superfamily protein
ıethanol-choline (GMC) oxidoreductase family protein
ılpha motif (SAM) domain-containing protein
'losidase 4
:vesting complex photosystem II subunit 6
ıscorbate oxidase
:sis-related thaumatin superfamily protein
:osidase 27
.transferase family protein
ıe P450, family 86, subfamily A, polypeptide 1
ɡlyoxylate aminotransferase
.in-regulated family protein
dehydrogenase 3F1
ɔtein of unknown function (DUF828)

.n-containing protein with a domain of unknown function (DUF21)
3;1
N-like arabinogalactan-protein 11

e-related gene 3
e acyl-transferase family protein
n repeat 6
f unknown function (DUF 3339)
hydratase/isomerase D
stidine transporter 1
ase-like superfamily protein
l-L-methionine-dependent methyltransferases superfamily protein
dine synthase family protein
ase-like superfamily protein
binding protein 28

x superfamily protein
cine zipper (bZIP) transcription factor family protein
alate dehydrogenase family protein

family protein
protein L13 family protein
e transporter family protein
reonine-protein kinase WNK (With No Lysine)-related
nine beta-synthase (CBS) family protein
n-containing protein
cleotide-gated cation channel 4
nosyltransferase 12
ne S-transferase TAU 19
em II subunit R
ectin protein kinase family protein
superfamily protein
family protein
effect embryo arrest 22
ke phosphatidylinositol transfer family protein
elated homeobox 13
lar 5
n-containing protein

hydrolase 9B5
homeodomain 7

ted kinase 2
ransporter 3;1
nal inhibitor/lipid-transfer protein/seed storage 2S albumin superfamily protein
ily protein
em II family protein
phosphate (PLP)-dependent transferases superfamily protein
n protein 93
x superfamily protein
ion and MADS-box transcription factor family protein
nding Rossmann-fold superfamily protein
o AP2 1
ng protein 47C
zinc finger family protein
ine peptidase 1
dent malic enzyme 2
binding protein 43

\pical Meristem) domain transcriptional regulator superfamily protein
:vesting chlorophyll-protein complex II subunit B1
)ic-responsive NPH3 family protein
lehyde 3-phosphate dehydrogenase A subunit 2
/ESR-RELATED 41

-acetic acid inducible 11
ıe P450, family 81, subfamily D, polypeptide 8
)inding EF-hand family protein
; resistance-associated protein 6
ıethanol-choline (GMC) oxidoreductase family protein

.ke protein 1
ıe P450 superfamily protein
ıe P450 superfamily protein
/brid motif superfamily protein
:otein 2
.pooxygenase, PLAT/LH2 family protein
.n protein 42
;e homolog E1B
'amily protein
)eta 8
ıbulin
/ase-like superfamily protein
.n protein 103
)rane amino acid transporter family protein
; synthase like G3
; synthase like G3
.n protein 67
.on protein SH3-like family protein
ın endotransglucosylase/hydrolase 5
ıin-like superfamily protein
.in 20-2

;ion and MADS-box transcription factor family protein
; protein dnaJ-related
. phosphate (PLP)-dependent transferases superfamily protein
ıle-associated protein 65-8
:opeptide repeat (TPR)-like superfamily protein
ıe gamma-lyase
ıe gamma-lyase
ıte:CoA ligase 2
:ansferase 1
:ich family protein
:oot primordium (LRP) protein-related
:ich repeat (LRR) family protein
lependent lipid-binding (CaLB domain) plant phosphoribosyltransferase family prote
.nding Rossmann-fold superfamily protein
:us rep interacting kinase 1
SYNTHASE
ı60 chaperonin family protein
:tase 3
le cyclase/dehydrase and lipid transport superfamily protein
ın endotransglucosylase/hydrolase 8

:em II subunit Q-2
ɔeptide/Phox/Bem1p family protein
ı induced protein with YGL and LRDR motifs
.n protein 12

ɔe acyl-transferase family protein
:al transport/detoxification superfamily protein
ɜ synthase family protein
ɜ Lipase/Acylhydrolase superfamily protein
ımily trypsin and protease inhibitor protein
ɔsyl transferase 88A1
ɛyglucosinolate 1
ɜ synthase clone R1
:olase-type esterase superfamily protein
.n like 23
:ich repeat protein kinase family protein
ɔnal inhibitor/lipid-transfer protein/seed storage 2S albumin superfamily protein

BIREFRINGENCE-LIKE 19
A1
: finger 2
synthase 1
ɛide synthase
:ome-interacting basic-helix-loop-helix 1

ıcine-zipper 44
-bisphosphate aldolase 2
:ich protein 4

ɔolymerizing factor 5
:yl CoA reductase-related male sterility protein
.ose/UDP-D-xylose synthase 2
ıoplast intrinsic protein
ɔf unknown function (DUF579)
:ilitator superfamily protein
ɜtylesterase family protein
.n fatty alcohol dehydrogenase family protein
ımine synthase 3
ɜ synthase 6
ıe P450, family 76, subfamily G, polypeptide 1
ɔntaining nucleoside triphosphate hydrolases superfamily protein
ɾcogenin-like starch initiation protein 5
ɡe-repair/toleration protein (DRT102)
ɾ element differentiation-related 7
ɾcogenin-like starch initiation protein 1
.n protein 93
ıe P450, family 86, subfamily B, polypeptide 1
:RIPE like 7
:actor Y, subunit C2
:elated homeobox 13
ɜmbrane intrinsic protein 2;8
ıcid 5-hydroxylase 1
.ng protein 1
ɔf unknown function (DUF579)

ox superfamily protein
in protein 93
-containing protein
transporter 1;3
transporter 1;3
MtN21 /EamA-like transporter family protein
superfamily protein
in protein 52
se superfamily protein
ne P450, family 78, subfamily A, polypeptide 7
tem II family protein
inked oxidoreductase superfamily protein
onal inhibitor/lipid-transfer protein/seed storage 2S albumin superfamily protein
in containing protein 74
Pase large subunit
be acyl-transferase family protein
binding lectin superfamily protein
actosidase 3
ptional factor B3 family protein / auxin-responsive factor AUX/IAA-related
se 3
anin 1
tosolic factor family protein / phosphoglyceride transfer family protein

dehyde-3-phosphate dehydrogenase B subunit
anscription factor family protein
transporter 4
transporter 4

osyltransferase superfamily protein
ase 9
ndent synthetase and ligase family protein
on defective 1 protein / ELD1 protein
protein S5 family protein
exchanger 20
onal inhibitor/lipid-transfer protein/seed storage 2S albumin superfamily protein
binding EF-hand family protein
in gene 2
MtN21 /EamA-like transporter family protein
inding Rossmann-fold superfamily protein
ike endoglucanases superfamily protein
17
inding Rossmann-fold superfamily protein

Diphenol oxidase family protein
-3-phosphate acyltransferase 6
embrane intrinsic protein 1;4
xchanger 3
id desaturase 8
xin superfamily protein
hydrolase superfamily protein
ctose transporter 3
protein L17 family protein

n family protein

ın endotransglucosylase/hydrolase 32
ɔmbrane intrinsic protein 2
N-like arabinogalactan-protein 11
ınsmembrane MLO family protein
se superfamily protein
one S-transferase family protein
xin superfamily protein

ate:CoA ligase 2
e synthase family protein
yl-CoA synthase 10
ed homeodomain-like superfamily protein
in protein 4

le cyclase/dehydrase and lipid transport superfamily protein
ain-containing protein
ain-containing protein
of unknown function, DUF642
-acetic acid inducible 29
in containing protein 38
e family protein
rich repeat (LRR) family protein
pe acyl-transferase family protein
-leucine zipper protein family
nolpyruvate carboxykinase 1
nain-containing protein kinase family protein
related NAC-domain protein 1
acid 5-hydroxylase 1
ta-Hydrolases superfamily protein

e homeodomain 4
e Lipase/Acylhydrolase superfamily protein
IC LEAVES 2-like 1
ipase A 2A

of unknown function (DUF579)
me P450, family 86, subfamily B, polypeptide 1
ain-containing protein / ABA-responsive protein-related
in protein 92
id amide hydrolase
id permease 2
in protein 69
ycogenin-like starch initiation protein 1
y element differentiation-related 7

roline-rich glycoprotein family protein
bonic anhydrase 5
in containing protein 73
rich repeat (LRR) family protein
de-diphospho-sugar transferases superfamily protein
nia lyase 1
ing (RRM/RBD/RNP motifs) family protein
pe transporter family protein
ck cognate protein 70-1

-3-phosphate acyltransferase 7
ictivase
:opeptide repeat (TPR)-like superfamily protein
kinase family protein
l-phosphate adenylyltransferase family protein
onal inhibitor/lipid-transfer protein/seed storage 2S albumin superfamily protein
ilose-bisphosphatase
otein of unknown function (DUF828)
BIREFRINGENCE-LIKE 33
5
l2
osylase superfamily protein
ln protein 67
orane amino acid transporter family protein
ease 3
n-like superfamily protein

osidase 1
rich protein family
cal transport/detoxification superfamily protein
le cyclase/dehydrase and lipid transport superfamily protein
vertase/pectin methylesterase inhibitor superfamily protein
hyde dehydrogenase family protein
an endotransglucosylase/hydrolase 28
onal inhibitor/lipid-transfer protein/seed storage 2S albumin superfamily protein
dehalogenase-like hydrolase (HAD) superfamily protein
yll A-B binding family protein
irylase 1
yl-CoA synthase 11
motif nuclear-localized protein 20
MtN21 /EamA-like transporter family protein
pane-fatty-acyl-phospholipid synthase
lated protein C2B
e S41 family protein
like superfamily protein
ne sulfoxide reductase B5
ated 1
BIREFRINGENCE-LIKE 11
on at the outer envelope membrane of chloroplasts 159
arboxypeptidase-like 51
of unknown function (DUF1635)
ln protein 36
kinase superfamily protein
le/sugar transporter family protein
le e 1 allergen and extensin family protein
onal inhibitor/lipid-transfer protein/seed storage 2S albumin superfamily protein
transcriptionally active 16

ne P450, family 86, subfamily A, polypeptide 1
P/DUF1795-like photosystem II reaction center PsbP family protein
nily protein
ne P450 superfamily protein
pe transporter family protein

lependent lipid-binding (CaLB domain) family protein
/uracil permease family protein
e zinc finger transcription factor family protein
f unknown function (DUF303)
l1
ca-Hydrolases superfamily protein
-prolyl cis-trans isomerase / cyclophilin-40 (CYP40) / rotamase
:arate (2OG) and Fe(II)-dependent oxygenase superfamily protein
in/WD40 repeat-like superfamily protein
e negative transcriptional regulator family protein
l-phosphate adenylyltransferase family protein

-bisphosphate aldolase 2
embrane intrinsic protein 1A
sis phospholipase-like protein (PEARLI 4) family
ltransferase family protein
e synthase-like A02
e Lipase/Acylhydrolase superfamily protein
2
ic aspartyl protease family protein
FtsZ family protein
nscription factor family protein
racting protein kinase 1
rfamily hydrolase, subfamily IG, 5\'-nucleotidase
etylesterase family protein
kinase 3

ble protein 1

be acyl-transferase family protein
lated protein C2B
of unknown function (DUF810)
transporter 3
ox superfamily protein
:arate (2OG) and Fe(II)-dependent oxygenase superfamily protein
e synthase like G1
yl-CoA synthase 2
prenylyltransferase superfamily protein
prin domain-containing protein
calcium-binding protein family

se superfamily protein
e protein htpG family protein
osylase superfamily protein
N-acyltransferases (NAT) superfamily protein
vase-like superfamily protein
of unknown function (DUF620)
in protein 67
osylase superfamily protein
nase family protein

l2
BIREFRINGENCE-LIKE 33
ne P450 superfamily protein

)rane amino acid transporter family protein
:d homeodomain-like superfamily protein
)nal inhibitor/lipid-transfer protein/seed storage 2S albumin superfamily protein

-3-phosphate acyltransferase 5
:erised protein family (UPF0497)
C2HC zinc fingers superfamily protein
)e transporter family protein
le-diphospho-sugar transferases superfamily protein
:a-Hydrolases superfamily protein

subunit exo70 family protein G1
1-specific protease family C19-related protein
.ux family protein
receptor kinase
:epeat family protein
:epeat family protein
:epeat family protein
:epeat family protein
:em II subunit Q-2
.ix-loop-helix (bHLH) DNA-binding superfamily protein
repeat cysteine protease family protein
:k cysteine peptidase 3
: Lipase/Acylhydrolase superfamily protein
BIREFRINGENCE-LIKE 38
)e transporter family protein
)ne S-transferase TAU 19
ılin A-like lectin protein kinase family protein
\pical Meristem) domain transcriptional regulator superfamily protein
.ng Berberine family protein
.ng Berberine family protein
7ase-like superfamily protein
:ransferase 1
/ESR-RELATED 41
ısferase 16

)xygenase
.n containing protein 58
: Lipase/Acylhydrolase superfamily protein
ıase superfamily protein
1
)-xylulose 5-phosphate reductoisomerase
:thionine sulfoxide reductase 1
le diphosphate kinase 2
)f unknown function (DUF 3339)
family protein
hydrolase family 38 protein
1-protoporphyrin IX methyltransferase

ıin-like superfamily protein
.ike protein 6
7ertase/pectin methylesterase inhibitor superfamily protein
7ertase/pectin methylesterase inhibitor superfamily protein
:amily protein

ın endotransglycosylase 6
›f unknown function, DUF538

›f unknown function (DUF1645)
ıe P450, family 93, subfamily D, polypeptide 1

wnregulated-like 1
-phosphate isomerase, type A protein
ıolpyruvate carboxylase kinase 2
copeptide repeat (PPR-like) superfamily protein
e-5\'-phosphosulfate (APS) kinase 3
e dehydrogenase 1
ox superfamily protein
A15
embrane choline transporter family protein
ıe P450 superfamily protein
pe acyl-transferase family protein
ylase superfamily protein
ylase superfamily protein
›f unknown function (DUF579)
protein 33
v-like 3
transferase family protein
3
ı 13

ix-loop-helix (bHLH) DNA-binding superfamily protein
6;1
n-containing protein 15
egulating factor 1
n-containing protein 38
nding Rossmann-fold superfamily protein
in-regulated family protein
ox superfamily protein
n protein 73
ıreonine-protein kinase WNK (With No Lysine)-related
n-like serine endopeptidase family protein
n-like serine endopeptidase family protein
n like 37

de-diphospho-sugar transferases superfamily protein
›f unknown function (DUF607)
osyltransferase superfamily protein
ıe P450, family 721, subfamily A, polypeptide 1
yl-L-methionine-dependent methyltransferases superfamily protein
pe transporter family protein
e zinc finger transcription factor family protein
-binding protein 43
ta-Hydrolases superfamily protein
ome interacting factor 3
r Glassy Yellow 1
vase-like superfamily protein
ontaining nucleoside triphosphate hydrolases superfamily protein
like 2

hydrolase 9B13
:in superfamily protein
:a-Hydrolases superfamily protein
:ilitator superfamily protein
:e to phytophthora 1
elix DNA-binding transcription factor family protein
of unknown function (DUF640)
kinase family protein
ix-loop-helix (bHLH) DNA-binding superfamily protein
ethylesterase 61
protein L6 family

:al transport/detoxification superfamily protein
l hydrolases family 17 protein
protein 34
n protein 52
ransferase family 61 protein
n containing protein 58

it superfamily protein
anhydrase 2
-binding family protein
l-ACP thioesterases B
defective 3 GTP-binding protein (RHD3)
dehalogenase-like hydrolase (HAD) superfamily protein
ertase/pectin methylesterase inhibitor superfamily protein
ux family protein
e e 1 allergen and extensin family protein
le-associated proteins 70-5
ase 9
monellin family protein
nding Rossmann-fold superfamily protein
k transcription factor B3
c aspartyl protease family protein
e peroxiredoxin B
in-like superfamily protein
le-diphospho-sugar transferases superfamily protein

onal inhibitor/lipid-transfer protein/seed storage 2S albumin superfamily protein
of unknown function (DUF962)
transporter 4;2
brane amino acid transporter family protein
otein of unknown function (DUF828)
of unknown function (DUF567)
hydrolase superfamily protein
etetrahydrofolate reductase 2
ase-like superfamily protein
peptidyl-prolyl cis-trans isomerase family protein
e P450, family 81, subfamily K, polypeptide 1
ase 2

protein 31
own complex homolog protein 11
inase family protein

.in 38
: mutarotase-like superfamily protein

.ix-loop-helix (bHLH) DNA-binding superfamily protein
:ich repeat protein kinase family protein
:olase-type esterase superfamily protein
: synthase clone R1
ger protein 7
RIPE like 7
-l-glutamate kinase
.n containing protein 38
: receptor 2.8
.d cleavage dioxygenase 1
.d cleavage dioxygenase 8
.c enzyme 3
ne P450 superfamily protein

.d biosynthesis 1
kin superfamily protein
membrane lipoprotein
on factor Ts family protein
bisphosphate carboxylase (small chain) family protein
: synthase family protein
.nnamoyl-CoA shikimate/quinate hydroxycinnamoyl transferase
:a-Hydrolases superfamily protein
1 family protein
le transporter family protein
:opeptide repeat (TPR)-like superfamily protein
.nity K+ transporter 1
osyltransferase superfamily protein
.c aspartyl protease family protein
nt lipase 1
: Lipase/Acylhydrolase superfamily protein
: cupins superfamily protein
: cupins superfamily protein
.n protein 9
1A
: 3
of unknown function (DUF506)
2
proton symporter 2
ox superfamily protein
nionein 2A
:erised protein family (UPF0497)
orane-anchor 1
.ix-loop-helix (bHLH) DNA-binding superfamily protein
1 13
3
oe acyl-transferase family protein
.ysine (K) kinase 4
ain-containing protein
.ectin protein kinase family protein
.pase A 2A
:geting protein for Xklp2) protein family

:ransporter 2;1
:otein 2-A1
'1 complex, gamma subunit protein
.pase A 2A
:tase 3
.nding Rossmann-fold superfamily protein
)e acyl-transferase family protein

:ich repeat protein kinase family protein
:arate (2OG) and Fe(II)-dependent oxygenase superfamily protein

ein
