# Supplementary material for: Transcription factor PagLBD21 functions as a repressor of secondary xylem development in Populus
Source: For Res (Fayettev). 2022 Dec 21;2:19. doi: 10.48130/FR-2022-0019 (PMC11524276; doi:10.48130/FR-2022-0019)
Supplement: Supplementary file 1 — Supplementary data to this article can be found online. [file FR-2022-0019-S1.zip › 10.48130_FR-2022-0019-Suppl-FigureS1.pdf]

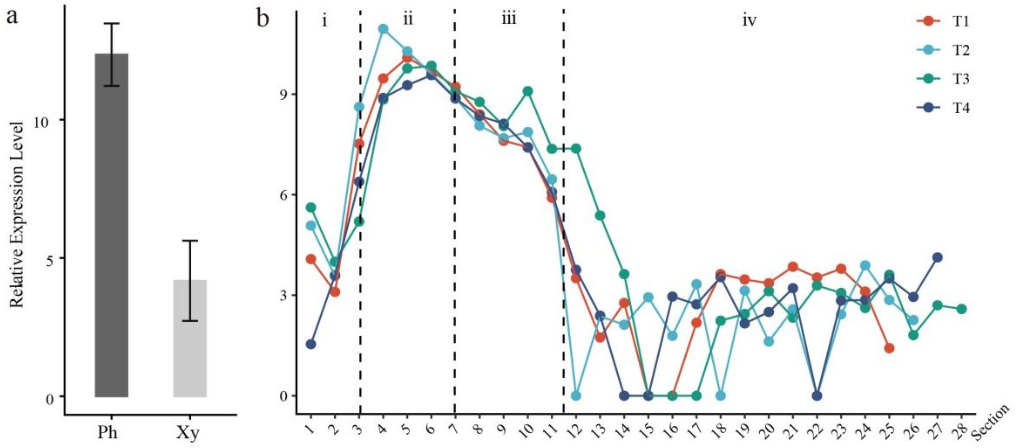

Supplemental Fig. S1. The expression levels of *PagLBD21* in *Populus trichocarpa*. (a) RNA-seq expression data in poplar phloem and xylem. Ph, phloem; Xy, xylem. (b) Expression profiles of the section of poplar cryosection from differentiated phloem to mature xylem in four trees (T1–T4). T, Tree. ⅰ, ⅱ, ⅲ, ⅳ indicate phloem, cambium, expanding xylem, and maturing xylem respectively. Each value is the mean ± standard error(SEM) of three replicates (n=3 technical repetitions).
